# Design – a new way to look at old molecules

**DOI:** 10.1515/jib-2022-0020

**Published:** 2022-07-01

**Authors:** Davide Spalvieri, Anne-Marine Mauviel, Matthieu Lambert, Nicolas Férey, Sophie Sacquin-Mora, Matthieu Chavent, Marc Baaden

**Affiliations:** Laboratoire de Biochimie Théorique, CNRS, Université Paris Cité, UPR 9080, 13 rue Pierre et Marie Curie, F-75005, Paris, France; Institut de Biologie Physico-Chimique - Fondation Edmond de Rothschild, Paris, France; Ecole Estienne, Paris, France; Université Paris-Saclay, CNRS, Laboratoire Interdisciplinaire des Sciences du Numérique, 91405, Orsay, France; Institut de Pharmacologie et de Biologie Structurale, Université de Toulouse, CNRS, Université Paul Sabatier, 31400, Toulouse, France

**Keywords:** exploded views, illustration, ion channels, molecular design, outreach

## Abstract

We discuss how design enriches molecular science, particularly structural biology and bioinformatics. We present two use cases, one in academic practice and the other to design for outreach. The first case targets the representation of ion channels and their dynamic properties. In the second, we document a transition process from a research environment to general-purpose designs. Several testimonials from practitioners are given. By describing the design process of abstracted shapes, exploded views of molecular structures, motion-averaged slices, 360-degree panoramic projections, and experiments with lit sphere shading, we document how designers help make scientific data accessible without betraying its meaning, and how a creative mind adds value over purely data-driven visualizations. A similar conclusion was drawn for public outreach, as we found that comic-book-style drawings are better suited for communicating science to a broad audience.

## 1 Introduction

The connections between art and science can be traced back to the history of science. From Leonardo Da Vinci’s drawings to Ernst Haeckel’s illustrations, the way man depicts nature can be considered a work of art. Conversely, the depiction of the beauty of nature is an eternal source of inspiration for artists such as painters, sculptors, etc. However, in the case of science, it is necessary to accurately represent what is seen in order to inform the scientific community correctly. This is straight forward if one can see the object of study – with various tools, from telescopes to microscopes – but how can this be achieved if one cannot see the object of research? How can you make the invisible visible? How can new findings be illustrated when there is no standard? In this case, we are no longer in the simple cases of visualisation, but beyond. In this twilight, the use of design can be a solution. If we search for the definition of the noun design, we find, “*The art of making plans or drawings for something*” (https://dictionary.cambridge.org/dictionary/english/design). Consistent with this description, we define design in the context of this article as the process or result of creating an effective plan, drawing, or model as a solution to a molecular visualization or analysis problem. This includes the process of developing conceptual ideas into scientific visualizations and representations. It comprises the creative processes of developing such ideas and communicating them visually.

**Figure 1:**
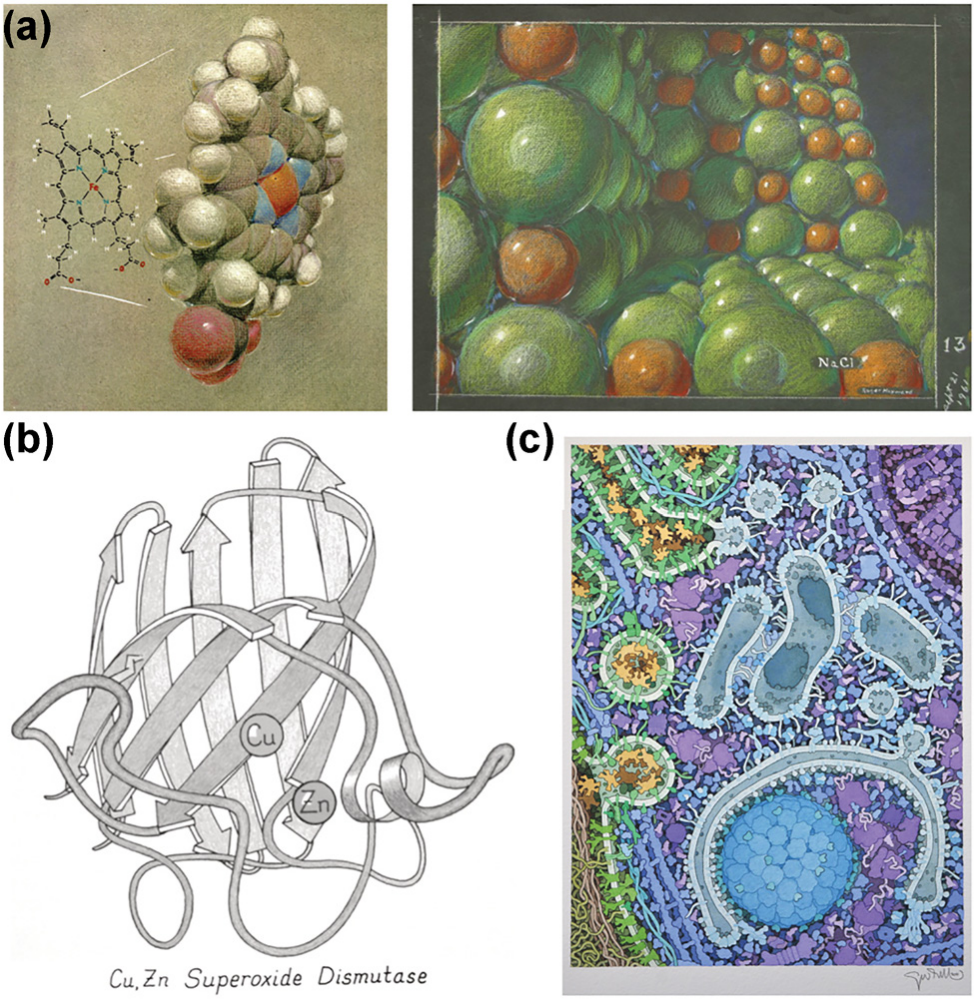
Molecular drawings. (a) Pastel drawings by Roger Hayward of a hemoglobin heme group (1964) or a NaCl crystal (1961) made during his collaboration with Linus Pauling (Special collections & Archives research Center, Oregon state College). (b) Ribbon drawing by Jane Richardson highlighting the beta strands in a protein (1981) [49, 50]. (c) Formation of an autophagosome illustrated by David S. Goodsell, in collaboration with Daniel Klionsky (2011).

When we turn to molecules, a collection of atoms, it has always been very difficult to “see” such small objects. Researchers have tried to find new ways to understand their behaviour and structures. Linus Pauling’s collaboration with Roger Haywards, “*architect, artist and illustrator*”, as presented in his online bibliography Renaissance Man [1], is a rather striking example of improving the understanding of molecular assemblies (Figure 1(a)).

Protein crystallography itself can serve as an example. In the early 1960s, the Nobel laureate Sir John Kendrew began “designing” molecular structures with wire and modelling clay. He had become interested in the crystallographic determination of protein structures. Thanks to the incorporation of heavy atoms into the relatively small protein myoglobin, diffraction spots were obtained that made it possible to resolve its three-dimensional structure. Using the computing facilities of the College of Cambridge, Kendrew succeeded in visualizing the structure of myoglobin in 1958. Some time later, he inspired Cyrus Levinthal and his colleagues at MIT to reproduce the same scientific structures using an oscilloscope.

Hence, the first computer representations of molecular structures used lines to represent bonds between atoms [2], followed later by computer visualisations of spheres or cylinders to represent atoms and bonds [3, 4]. In the early 1980s, the molecular surface was introduced to represent parts of molecules accessible to water by combining parts of spheres and torus to form a relatively smooth surface [5]. More recently, parts of hyperboloids have been used to render molecular surfaces and other representations [6, 7]. Rendering of all these representations is now accelerated by GPU functions [8]. While the recent trend has been toward increasingly smooth molecular representations, tetrahedra have been proposed as the basis for representing molecules at various scales [9]. Unfortunately, the primitive geometric shapes described so far are quite limited for complex molecular systems. This problem is overcome by involving designers, illustrators and artists who can offer new perspectives on molecular objects and expand the repertoire of shapes to be considered.

Art is a valuable source of new designs for representing and understanding proteins, as illustrated by the beta-sheet topology designed by Dr. Jane Richardson (Figure 1(b)), inspired by Greek and Native American symbols on pottery [10]. Several works by illustrators, animators, and artists such as Drew Berry [11], Gaël McGill [12], Janet Iwasa [13], and Graham Johnson [14] at the intersection of art and science are redefining the way we see the molecular world. The molecular paintings of David Goodsell (Figure 1(c)) [15] have been widely disseminated outside the scientific community [16, 17]. These and other works are helping to rehabilitate hand-drawing techniques [18] in an academic context [19]. A recent review article describes the process of developing and applying visualization and analysis methods for molecular structures and provides some thoughts on the current challenges in the field of biomolecular visualization [20].

The collaboration of designers, illustrators and artists with scientists has been around for many decades. The application of concepts from contemporary design is a more recent development, as this discipline has made tremendous strides recently. A specific example of the use of design to improve the representation of molecular structures is the Aquaria software, which explicitly incorporated these concepts into its development [21, 22]. Some related studies are briefly described to provide a framework for the present work. Traditionally, the influence of visual design and illustration has been most pronounced. This is true for the representation of complex biological objects, e.g., membranes [23], cells [24] and pathogens [25].

A recurring theme related to the visualization of complex biological objects is the development of appropriate abstractions. Valuable concepts have emerged in this context, such as continuous abstraction [26] and simplification of the shapes of molecular surfaces [27]. Work on the concept of abstracted molecular surfaces continues in various scientific projects. A recent example are the resources for peripheral protein-membrane interactions developed by the Reuter group in Norway (https://reuter-group.github.io/peprmint/).

A final example of the transfer of design concepts to biology is exploded views. As described below, one designer inspired us to apply the concept of exploded views to molecular objects, which we then described and implemented in 2014 [28, 29]. This concept was subsequently expanded and generalized by other groups [30].

Despite all this previous work illustrating the added value of design considerations, such a cross-disciplinary approach remains rare. Here we provide some examples of how it has been possible to make fruitful connections with illustrators or designers to present research developed in scientific laboratories in new ways. Through design approaches, molecular scientists can find new ways to view their favourite objects and better connect with the broader community. We have had conversations with designers at different levels and will first present their views and then discuss two use cases, one related to academic practice and the other to design for outreach. In the first case, we address the representation of ion channels and their dynamic properties, as this family of proteins is an important target of current research and a fruitful source for design improvements. In the second case, we document part of the process of transitioning from a research setting to more widely accessible designs. Throughout the manuscript, we discuss examples of how computer software can help designers represent the molecular world.

## 2 The designer in the lab

Only in a few scientific disciplines is it common to employ an illustrator or designer in the laboratory. In bioinformatics, and more generally in biology, this is unfortunately a rare opportunity. Nevertheless, there are some bridges, for example through internships in collaboration with design schools, when preparing an illustrated book for public relations, or when a designer decides to work in an academic environment for a while. We have collected five testimonials from practitioners in these circles who have had the opportunity to observe or directly participate in such interactions. To this end, we have analyzed the contributions of Matthieu Lambert, lecturer at the Ecole Estienne of Applied Arts in Paris; Alain Bade, pedagogical director of EPSAA, the School of Visual Communication of the City of Paris; Davide Spalvieri, art director, graphic designer, videomaker, and photographer; Carsten Janke, biologist at the Institut Curie in Orsay; and Renaud Chabrier, writer, designer, film director and researcher at the Ecole Polytechnique – IP Paris. Their complete original texts can be found in the supplementary material, while we place excerpts from their contributions in context below.

### 2.1 Bringing designers to the lab: benefits and motivation

In the interviews it was pointed out by Carsten Janke that *the representation of scientific concepts across size and time scales is a difficult task and requires collaboration with graphic designers or artists*. Thereby, *research work, scientific concepts and their greater impact are communicated in a way that is comprehensible to a large public* – *both scientific and lay*. Alain Bade emphasized that *the goal is to make science accessible to the greatest number of people without betraying its meaning, an ideal challenge for a visual communication student.* This process implies a confrontation of visual communication tasks with realistic expectations in terms of constraints and goals. It represents *an opportunity for the lab to see the fruits of its research through a different prism*. As Carsten Janke further points out, this approach *stimulates both, scientists and graphic artists, to go beyond their comfort zones. The interactions with scientists and artists is a fruitful way of fostering scientific thinking and goes beyond the pure illustration of science* – *it also helps the scientists to think further about their own work, and discover greater concepts that crystallise during their work with the artist.* This idea is further echoed by Renaud Chabrier’s statement that *the work of a scientific illustrator can be very close to the work of a consultant: through addressing the question* “*how can we visualize this idea*?”, *it brings researchers the occasion to identify problems like blind spots, contradictions or confusion in the proportions and scales.*

### 2.2 The art of designing for research

As Matthieu Lambert explains, *design research consists of the articulation between hypotheses from collected data or formulated considerations and concrete experiments with different tools. The connection between the different actors of the project* – *designer, scientific sender and receiving audience* – *is fundamental to ensure the effectiveness of the resulting mediation system*. *The scientific illustrator must also know how to adapt to different contexts, taking care at each step to enter into a dialogue with his interlocutors*. *The key to the success of an information design project in this field is collaboration*. Collaboration is emphasized in several testimonies, for instance in terms of pedagogical considerations and the variety of produced output. When reflecting on his own projects, Renaud Chabrier summarizes that *each of those experiences confirmed the benefit of a close collaboration between designers and researchers*. Matthieu Lambert points out that *scientific illustration is a deeply transdisciplinary activity*. Another recurring aspect concerns the creative approach and its inherent link to art, which is aptly expressed by Davide Spalvieri: *It’s interesting how many scientists are involved in art or simply art lovers. This means that art needs understanding and cognition. In a world where CG, imagery and scan technology are extremely advanced, a creative mind still adds value with his work to science*.

### 2.3 We need creative humans for design tasks

Davide Spalvieri further states that *if in the past illustration was the only way to represent reality, today creatives aim to represent what is to tell in the fastest and clearest way our brain can digest*. According to Alain Bade, such works *require a synthetic mind, the ability to understand complex processes, analyze them and translate them into a visual language that can be understood by all audiences*. The creative aspect may set an illustrator apart from a designer according to Davide Spalvieri: *we can differentiate the illustrator and the designer: an illustrator is asked to realise images by more or less traditional techniques such as hand and digital drawing, 3d modeling and rendering. He could employ imagery as part of his work. A designer is asked to find a creative solution for a specific need*. Renaud Chabrier further mentions that *the designer is here to orient the team towards the best approach for the problem to be addressed, and the kind of public in view*. This finds some echo in Davide Spalvieri’s comment that *a faithful image is not always what we need to understand something: too much information (detail), extremely big or small scales in space (such as in astronomy opposed to microbiology) and the lack of codes lead the viewer to confusion. The illustrator/designer understands what is to hide and what is to visually highlight to make an artifact clear, to make it speak*. The accountability for such decisions is explicitly stressed by Matthieu Lambert expecting a designer to *present his progress in design in a didactic way and to justify each of his choices*. It also sets the human apart from a machine as Davide Spalvieri points out: *simplification is also something that machines can do, but they need parameters set by a human and they are not able to work it through to find a path, a scheme, a structure and links between the elements in the picture. An illustrator/designer can use the conventional visual codes to speak through images, and can even conceive new visual codes*. As elegantly summarized by Alain Bade, *the goal is to make science accessible to the greatest number of people without betraying its meaning*.

### 2.4 Challenges facing designers in the lab

How can designers efficiently be integrated into a research lab setting? Carsten Janke points out an interesting approach, as he has *organised several scientific meetings in which artists were documenting the science presented in the talks by drawings, which built the groundwork for future scientific communication and outreach*. Alain Bade describes how his team *integrated an art-science interface into the teaching program, which sends all their students into different research laboratories to directly interact with researchers and present their scientific work in a graphic format that is then presented to the lay public*. Renaud Chabrier makes an important point about sustainability: *Efficient scientific design is more like teamwork that develops in the long term. It can be very comparable to science laboratories, with less expensive equipment and more diverse collaborations, and I think it should be funded and organized as such*. “*Design*” *has recently become a generic word that can gather many activities beyond its classical meaning: author, film director, illustrator, and so on. Today the collaboration between designers and scientific researchers is probably mandatory for a good coordination between science and society.*

### 2.5 Discussion

The testimonials were very valuable for our own research, which we present below. They guided us in our approach to interact directly with designers, especially to benefit from their creative input, as mentioned earlier. The research objectives we faced aligned perfectly with the feedback we had received on expected benefits and initial motivations. We followed the collaborative approach mentioned above, and the designers’ decisions were discussed and justified.

## 3 Establishing the link between science and design

This section focuses on two use cases for designing molecular visualizations for research and for outreach. In the case of research, our main goal was to illustrate molecular processes related to allostery by applying the techniques of scientific and medical illustration to ligand-gated ion channels. As for public relations, the goal was to design graphic representations of proteins for a wide audience to illustrate a scientific book and make it more accessible.

### 3.1 Designing information visualization for ion channels

The research interaction on ion channels was designed during a 10-month internship by Mr. Davide Spalvieri, who was then a student at the Ecole Estienne of Applied Arts in Paris. The design object is a transmembrane protein fundamental for communication between cells, especially at the level of neuronal synapses: the pentameric ion channel GLIC [31]. Academic laboratories around the world are trying to understand the properties and mechanisms that control this family of channels by analyzing the results of molecular dynamics simulations. The design approach focuses on the clarity with which this information of interest is presented.

#### 3.1.1 Preliminary considerations and project settings

The first phase involved an analysis of realistic 3D representations of raw molecular data that is commonly used to focus on precision, computer friendliness, interactivity, and realism. The researchers will not question their validity, while the designer would analyze fundamental questions about communication: What visual codes should be used? What support and context? What detail should be focused on? What should be hidden and what should be highlighted? What perspective and strategy should be chosen? The goal of an illustrator’s sketch is to be more direct and effective than precise but overly detailed images. It is necessary to simplify shapes to schematically represent what a protein or part of it looks like; it may be necessary to exaggerate certain movements or phenomena to emphasize their importance. This kind of manipulation cannot be done automatically by computers, because it requires intuition and the ability to judge on a case-by-case basis whether an expression is necessary. Our goal is to develop a method to represent selected properties derived from molecular dynamics simulations in a graphically appealing manner to facilitate understanding of the data. Experiments were performed with graphical solutions to simplify the shapes and divide the phenomena into stages. Homogeneous graphical codes were developed. Supplementary Figure S1 shows the choice of colour scale for the project, while Supplementary Figure S2 shows initial drawings related to the scientific domain and context.

#### 3.1.2 Learning the system – first sketches

How would we consider the GLIC ion channel from a design perspective? This protein allows the selective passage of ions across a cell membrane. It forms a tunnel through the membrane barrier and only allows ions with a certain charge or size to pass. The homo-pentameric protein consists of five identical substructures, as shown in the initial sketches for this system in Supplementary Figure S3. The conformation of the channel pore changes by opening and closing the passage of ions. This so-called gating process is associated with a twisting of the extracellular part of the membrane protein, with the transmembrane part adapting and at the same time reducing the internal space. From a design perspective, the analogy to a twisted beverage can was explored to convey the concept in an intuitive way (Supplementary Figure S3a).

#### 3.1.3 From simplifying the complexity of structure to exploded views

**Figure 2:**
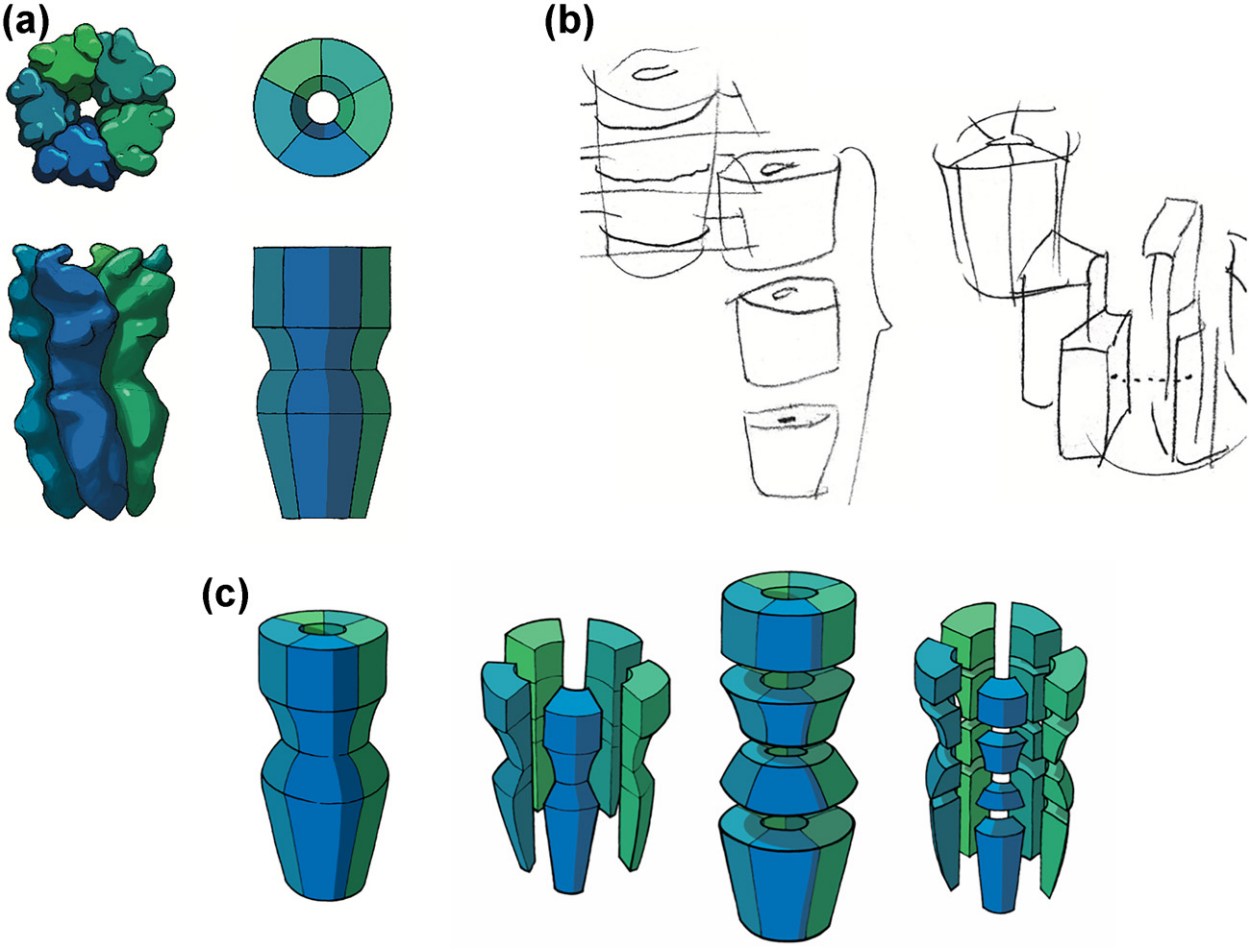
Simplification and decomposition of molecular structure. (a) Grasping the essential features of the molecular surface, followed by geometric abstraction. (b) First sketches applying the idea of exploded view to molecules. (c) Explosion of the geometrically abstracted protein shape according to its division into 5 monomers with respect to the symmetry axis or vertically with respect to the membrane or a combination of both.

The first realization regarding the commonly used surface representation of the receptor protein was that it is very complex and visually confusing. After a series of experiments, some of which are shown in Supplementary Figure S3, we agreed on the two levels of representation shown in Figure 2(a): a simplified but still “realistic” representation of the shape of the molecular surface that can be easily interpreted by expert scientists, or a very abstracted representation with simple geometric elements that requires rethinking the usual visual codes. In both cases, the colour emphasizes the substructure associated with the five monomers.

Another shortcoming is that the surface representation hides the interior of the molecule, and in particular the interfaces between subunits. Thus, we came up with the idea of applying the common design concept of exploded view to our molecular objects. Initial conceptual sketches of this idea can be found in Figure 2(b). The application to a simplified abstracted protein representation is shown in Figure 2(c). Such a systematic decomposition of molecular architecture is very insightful, and to our knowledge an unprecedented way to decompose molecular complexity. We published a first conceptual implementation in two articles in 2014 and 2015 [28, 29].

#### 3.1.4 Unrolling the 3D structure for an overview

**Figure 3:**
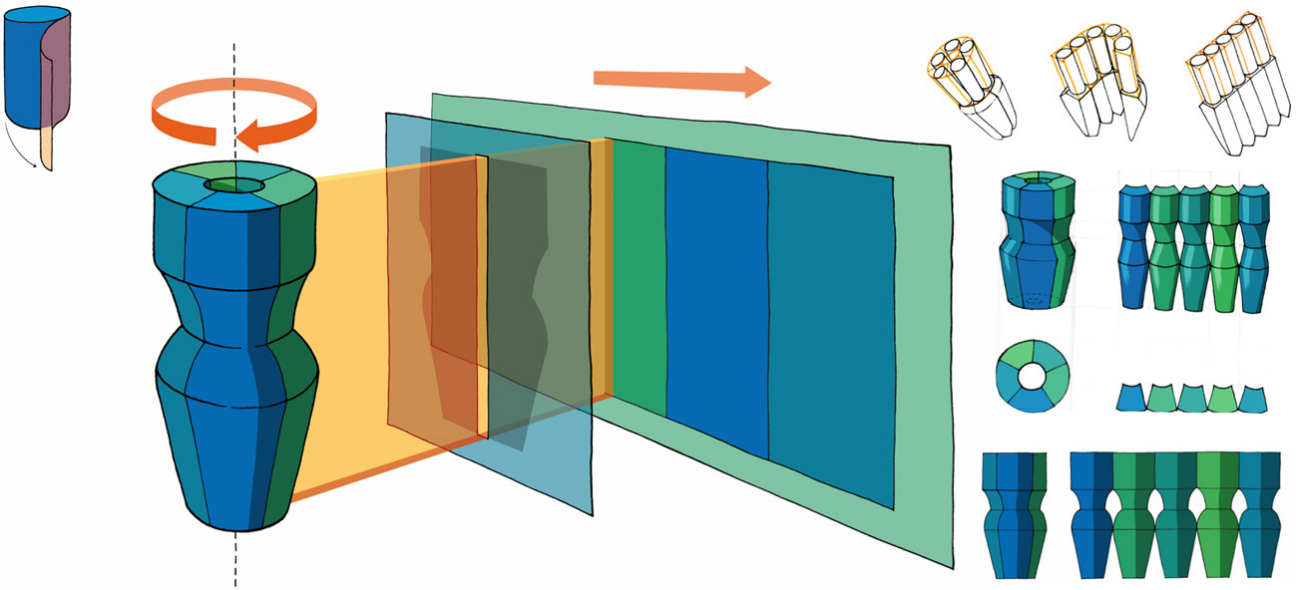
Unrolling of the surface. Transfer of the photographic principle of 360-degree rotation of the camera to the case of taking a panorama of a protein surface. The final concept is drawn in the centre of the image, the first studies are shown smaller on the right side.

Given the practice of scientific publications in 2D paper format, we have looked for ways to create a comprehensive 2D-compatible overview by “flattening” the third dimension. The concept is not new and has been used in the past [32] and more recently [33, 34] for scientific illustrations. Our inspiration for a new way to design it came from photography, more specifically from the Lomography Spinner 360°, a free-moving 360° panoramic camera that rotates 360° on its axis and captures everything around it in a single image. We have developed a similar approach, shown in Figure 3. The approach unrolls the protein surface onto a flat 2D projection. This method is suitable for the pentameric channels because they have a roughly cylindrical shape with a well-defined central axis. When the surface is graphically labelled with specific properties, especially those calculated during a molecular dynamics simulation, such a 2D map allows analysis of time evolution or easy comparison of different situations, since the entire surface is shown in a single view without hidden segments.

#### 3.1.5 Slicing, contours and dynamics to reveal function

**Figure 4:**
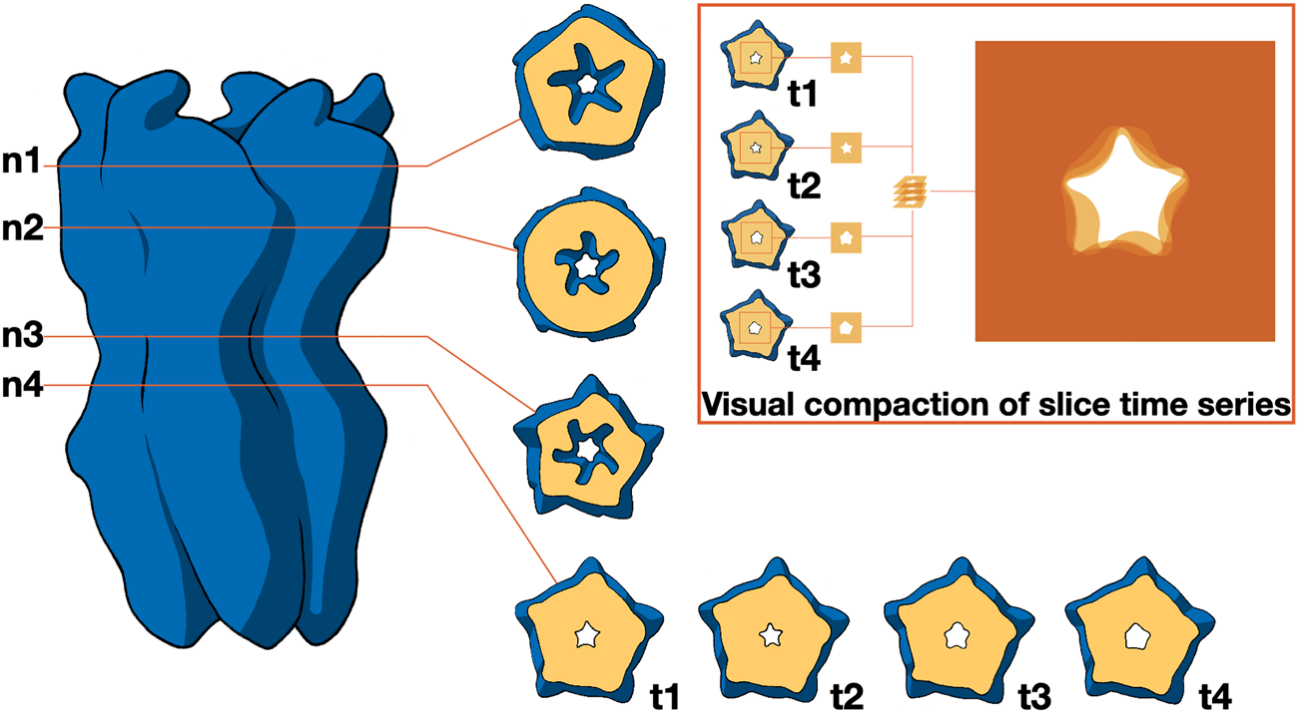
Temporal distribution of the ionic passages. It is shown how the contour of the ionic permeation pores can be detected by sections at different heights (n1 to n4) with respect to the membrane. The main constriction n4 is detected in a time series (t1 to t4) that can be visually condensed by combining transparent images, as shown in the inset.

By slicing the channel, as shown in Supplemental Figure S4, we can identify the presence of a passage through the protein that allows ion permeation. A lateral contour helps locate key channel constrictions used as reference points to create a contour slice, as shown in Figure 4. The key constriction (n4) was selected to be tracked over time during a molecular dynamics simulation through a time series of contours that can be visually condensed by combining the individual images with an appropriate transparency level. The resulting 2D image summarizes the temporal evolution, which in general remains a challenging task as pointed out in [35].

#### 3.1.6 Critical discussion

In this critical discussion, we will compare our findings with the existing literature. For exploded views following the corresponding approach in mechanical drawing, we are only aware of the recent work of Sbardellati et al. [30], which provides a very thorough and detailed analysis, while our focus is on abstracting the hull and generalizing the approach to large structural databases. The 2D imaging of the surface by unrolling that we present complements the numerous works on synthetic 2D map-like representations of molecules such as [36] or [34] by its unconventional implementation that mimics the physical process of a 360-degree photo camera. It is very simple compared to what has been described in the literature for more than 35 years [32] and serves more to illustrate how a design idea can be directly translated into a simple visualization tool than to offer new insights into such a mapping. The contour slicing we propose is a standard representation in the field of ion channels [37]. Here, it serves as an illustration of how a designer achieves a goal (“represent dynamics in a still image”) using a repertoire of illustration techniques, such as, in this case, combining transparent contour planes.

### 3.2 Designing proteins for a broad audience, the *Top of the Prots* experiment

**Figure 5:**
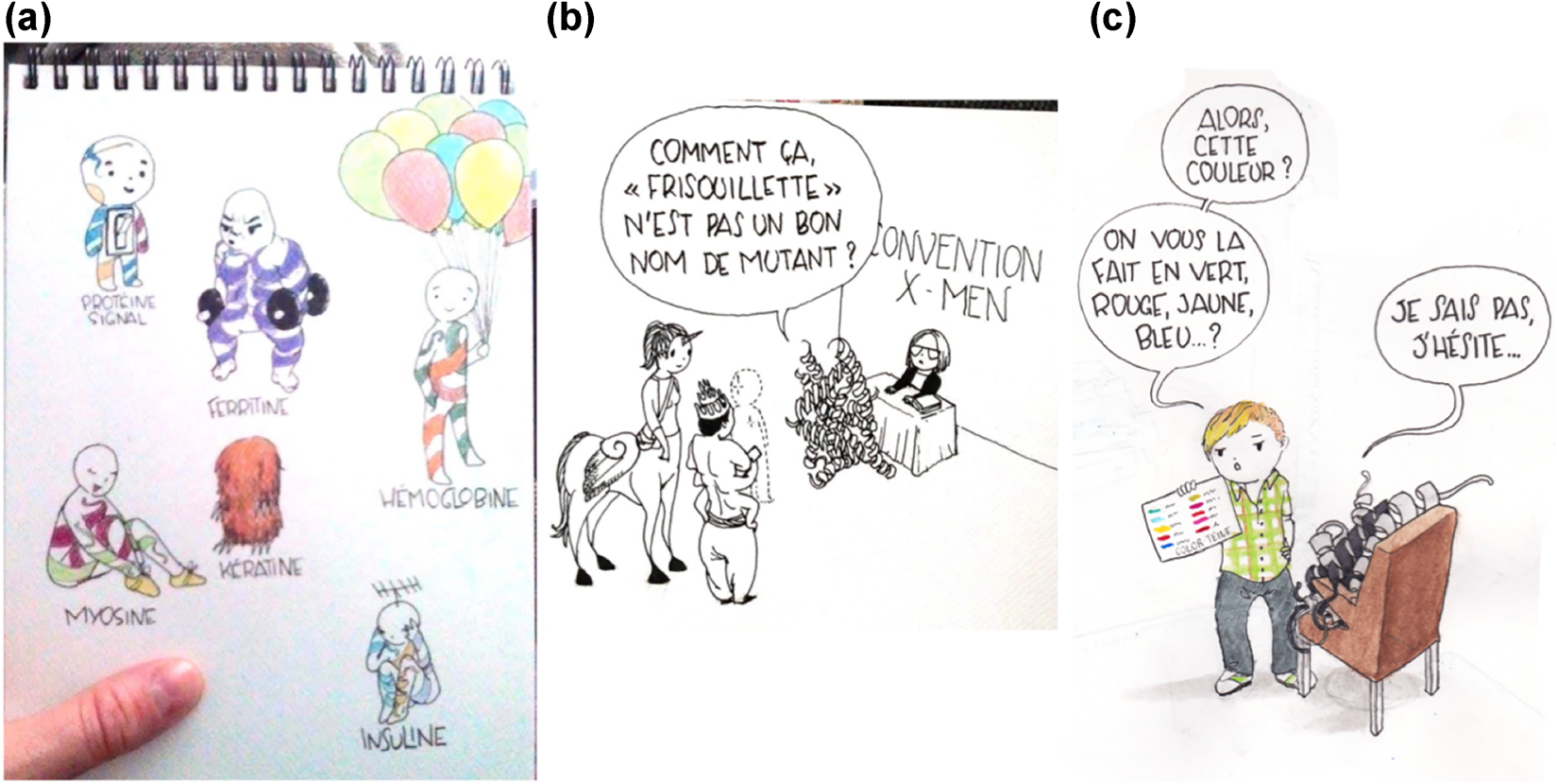
Early sketches by Anmryn for the protein figures. (a) Humanoid figures showing the function of proteins (e.g., hemoglobin, right, carries around a couple of balloons). (b) A mutant protein trying to join the X-Men team. (c) A protein that chooses the colour of its GFP tags at the hairdresser.

**Figure 6:**
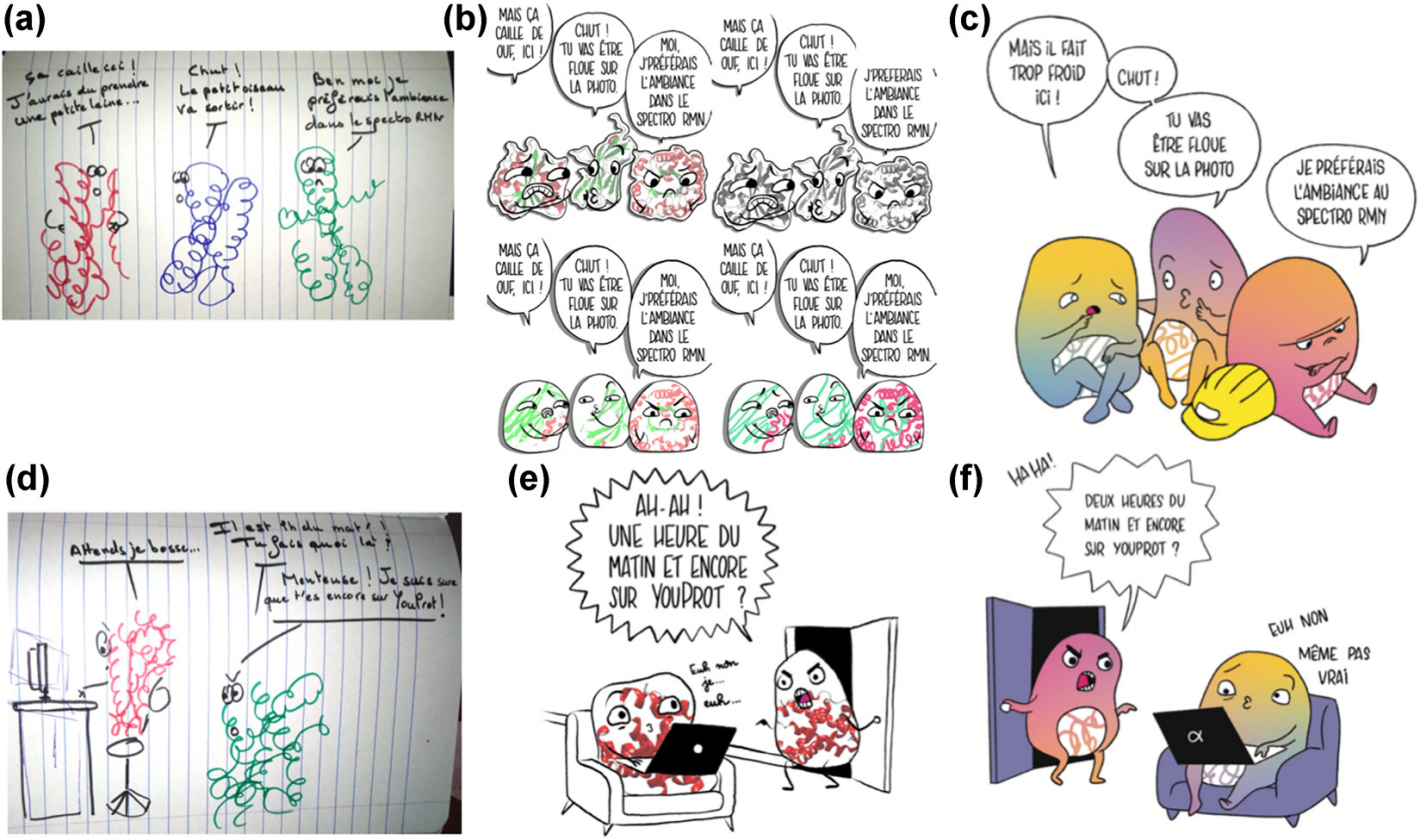
From scribble to publication. (a–c) Three proteins in the Cryo-EM spectrometer complain about the cold. (a) Original drawing by S. Sacquin-Mora (b) Various tests by Anmryn. (c) Final published figure by Anmryn. (d–f) some proteins spend too much time on the Internet. (d) Original drawing by S. Sacquin-Mora. (e) Test by Anmryn. (f) Final published figure of Anmryn. (Figures (c) and (f) reproduced with permission from EDP-Sciences).

Top of the Prots (https://topoftheprots.com/) is a French blog that publishes a short post (about 600 words) each week about a particular protein of interest. Each post includes both scientific, “serious” images of proteins created with classic VMD software [38] (see Supplementary Figure S5a) and comic-style illustrations showing proteins as humorous characters, according to rule #4 for drawing scientific comics [39]: *characters can improve engagement*. Relying on rule No. 1 (i.e., *you do not have to be good at art*), the authors optimistically provided their own drawings to illustrate the blog (see Supplementary Fig. S5b). When the blog was to be turned into a popular science book, it became clear that the illustrations should be redesigned by a more professional hand. Illustrator Anne–Marine Mauviel (who works under the name Anmryn) joined the team to design the illustrations in the style of a comic book. Having no scientific training herself, deciding how to depict the protein characters was a long process of trial and error involving the author team and the book’s editor. It started with human-like figures (see Figure 5(a)) and then progressed to figures shaped like “real” proteins, i.e., showing secondary structures (such as helices) (Figure 5(b) and (c)). Since these were too complex for a comic-style illustration, she tried to simplify this representation by using rough envelopes with protein drawings (Figure 6(b) and (e)). The final step (and choice) was to depict proteins as tiny figures with scribbled helices on their bellies (Figure 6(c) and (f)). These were often depicted with a construction helmet to emphasize the central role of proteins in cell function. The cartoon style of the proteins was a long elaboration to settle on body proportions, round shapes and anime eyes. Anmryn suggested a more attractive colour palette to match her own drawings (see Supplementary Figure S6a). Only the primary colors (yellow, magenta and cyan) were used, with the tacit message that proteins are the basic building blocks of life. Gradients were used to add more variety. Since there are no shadows, this also provides more texture. Overall, colors were an important point of discussion because, although expensive to print, they are a good way to give molecular objects more expressiveness: angry proteins are usually red (see Figure 6(c) and (f)), peaceful proteins blue, and so on. To distinguish them from the other drawings of “real things” of the macro world (people, animals …), these are shown only in black and white and not in a cartoonish way (see Supplementary Figure S7, an example of the “blue fulgates”). Colour appears only when proteins are involved. Because a realistic style was necessary to give uniformity to the book, everything was redrawn to maintain the same line thickness and overall look, drastically limiting the colour palette. Anmryn completely redesigned and redrew the original table of natural amino acids in the introductory chapters (Supplementary Figure S6b and c).

To position our approach against the existing literature and to provide a critical discussion, the works of Farinella et al. [40], McDermott et al. [39], Tigges et al. [41] and Kearns et al. [42] are particularly relevant. Our own results are consistent with their observations and can be summarized as follows: while precision is a key element in illustrating scientific publications, comic-style drawings are better suited to communicate with a broad audience.

## 4 Examples of implementing design ideas into a scientific workflow

**Figure 7:**
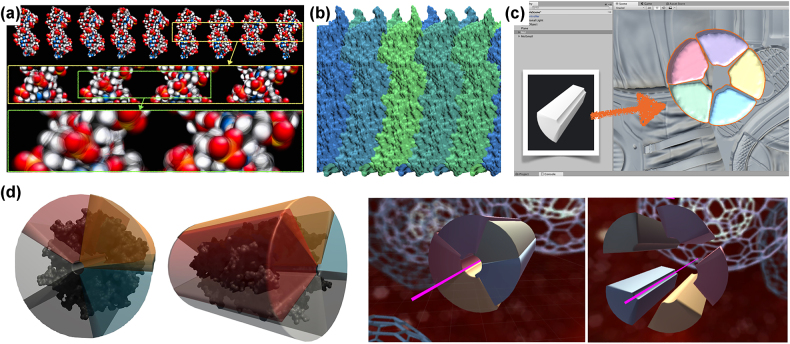
Design implementations. (a) is an experiment to visually capture motion in a static image of a DNA strand using motion blur. (b) is an implementation of the surface panorama. (c) is a geometric abstraction of the GLIC ion channel. (d) shows exploded views of the abstracted channel model.

A fundamental question that arose in the experiments described in the previous section is how to capture the creative know-how of the designer. It cannot simply be digitized to make it available in computerized tools and software. Nevertheless, some examples are documented in the literature, and without claiming to be exhaustive, we would like to mention here, for example, the tools MolScript [43] for detailed and schematic representations of protein structures, ProteinShader [44] for illustrative rendering of macromolecules, the software Illustrate for biomolecular illustrations [45] and Cellpaint [46] (https://ccsb.scripps.edu/cellpaint/), which enables to draw cellular landscapes using Goodsell-like biomolecular representations. For our own experiments, we have implemented prototypes in UnityMol [47], or developed simple generic scripts that work with a variety of programs. As an example, Figure 7(a) shows an experiment to represent molecular motion in still images.

With this in mind, we turned some of the ideas from Chapter 3 into tool prototype implementations. For example, rolling out the 3D structure for an overview is relatively easy to implement as a camera motion that captures the corresponding images as they are rotated and stitched together as a panorama. The resulting 2D view of the GLIC ion channel surface can be seen in Figure 7(b).

In the second example, we extended the previous prototype for exploded views implemented in UnityMol [28, 29] by abstracting the molecular objects in a systematic way and generalizing criteria for their dissection, which is detailed in Supplementary material. The final result is shown in Figure 7(d). We are continuing the experimentation with the generalization of the enclosing shapes. A few early prototypes are depicted in Supplementary Figure S9.

To capture the added value of artistic renderings of molecular objects, we used a well-documented approach for our third experiment on lit sphere shading, which is described in more detail in supplementary material. We detail how a student project was set up to experiment with this approach with the aim to intuitively visualize molecular properties. Here, we only mention one solution, based on paper-cut and assembled spheres (Supplementary Figure S10c), with the example of representing the flexibility concept through a sort of elastomeric pattern (Supplementary Figure S10d), or the toxicity property through a combination of colour and texturing (Supplementary Figure S10e).

In these three implementations, domain scientists have tried to translate the designers’ ideas into tools or to capture artistic originality through specific procedures. Ideally, if the designer himself could make this transfer, one might expect higher fidelity to the original idea. To facilitate such self-implementation by designers and illustrators, generative design can be an excellent approach. In generative design, the visual idea is not implemented by hand or with the digital processes that now replace the earlier Illustrator tools. Instead, it is rewritten into a set of rules and then programmed. Processing, a fairly simple graphics-oriented programming software [48], is an established framework for such an approach. With a few lines of code, you can obtain interactive images, modify them, and create visual effects based on “rules” established by the designer that determine the behaviour of the software.

## 5 Conclusions

In order to develop or better communicate a new scientific visualisation strategy to a large audience, a collaboration between scientists and designers/illustrators has proven to be very successful. Interestingly, while the precision of the illustrations is a key element in scientific publications dealing with complex systems such as biomolecules, we found that “comics” style drawings are better adapted for scientific communication for a broad audience, as these will ensure a wider outreach [40–42]. Although this collaboration has mainly come about by bringing designers into a scientific environment, it would be interesting to see if scientists could join a team of designers or work on a team of illustrators for a few months to help them think outside the box of science and develop new communication skills or even develop new designs to solve scientific problems. In addition to the actual design process and the different perspectives in approaching a particular problem, software tools are also an important consideration. In fact, a number of programmes such as Blender (https://www.blender.org) are commonly used to create animations or illustrations. It is now possible to use such programmes for rendering molecular systems (http://www.bioblender.org) and their properties, paving the way to even more insightful renderings. In this case, the choice of colouring and lighting is critical to our understanding of these systems (Supplementary Figure S11) [51]. Again, collaboration with designers, illustrators, and artists is essential to harness the power of these new tools. Our plans for the future include strengthening such collaborations, with the explicit goal of developing useable software implementations that translate the design concept into actionable tools. This would allow subsequent evaluation studies to objectively quantify the benefits of a particular design.

## Supplementary Material

Supplementary Material Details

Supplementary Material Details
