# Supplementary material for: Design – a new way to look at old molecules
Source: J Integr Bioinform. 2022 Jul 1;19(2):20220020. doi: 10.1515/jib-2022-0020 (PMC9377703; doi:10.1515/jib-2022-0020)
Supplement: Supplementary file 1 — Supplementary Material Details [file j_jib-2022-0020_suppl_001.pdf]

Rendu artistique de propriétés moléculaires par l’approche dite «Lit-sphère»

Julie Borgese

JULIE BORGESE

La dernière fois...

Rendu artistique de propriétés moléculaires par l'approche dite « lit-sphère »

Visuel abouti

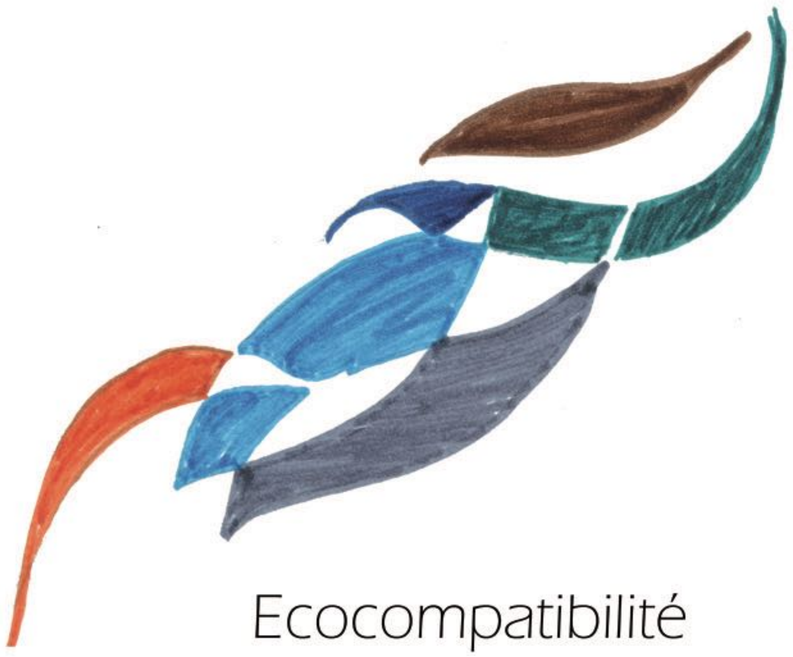

Ecocompatibilité

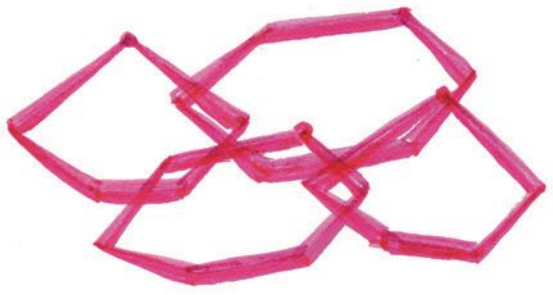

Sucre

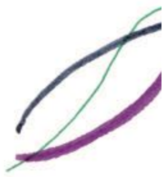

JULIE BORGESE

Flexibilité/rigidité

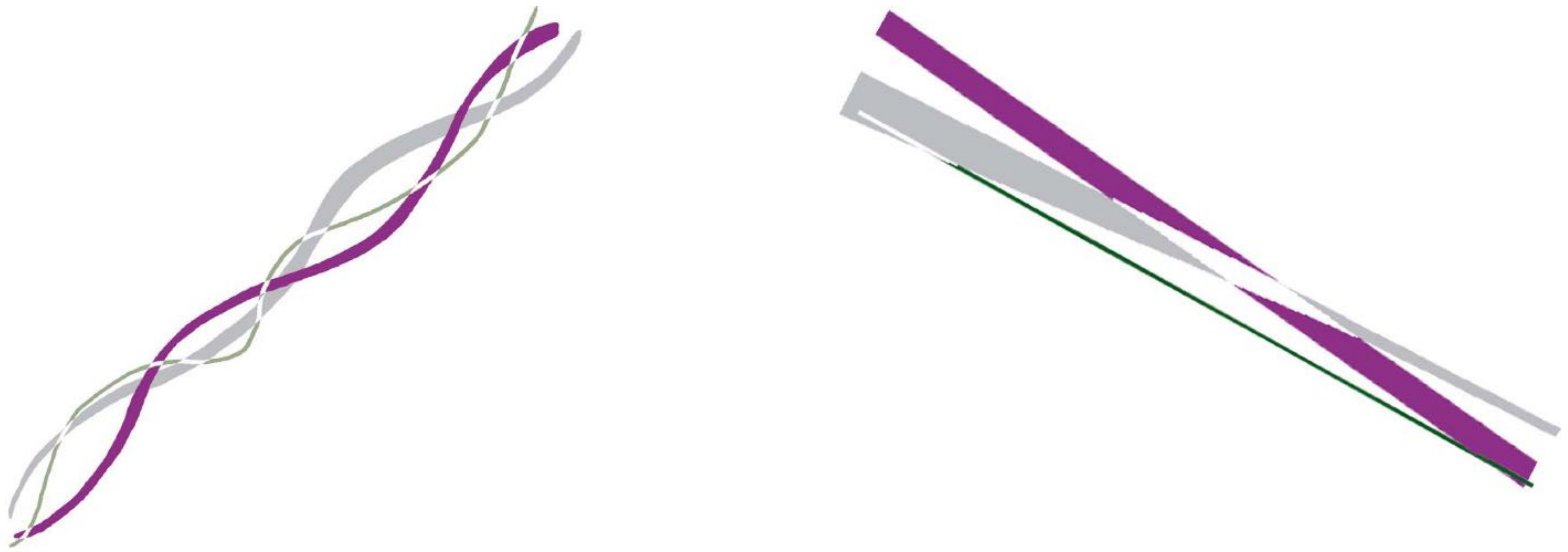

JULIE BORGESE

Visuel

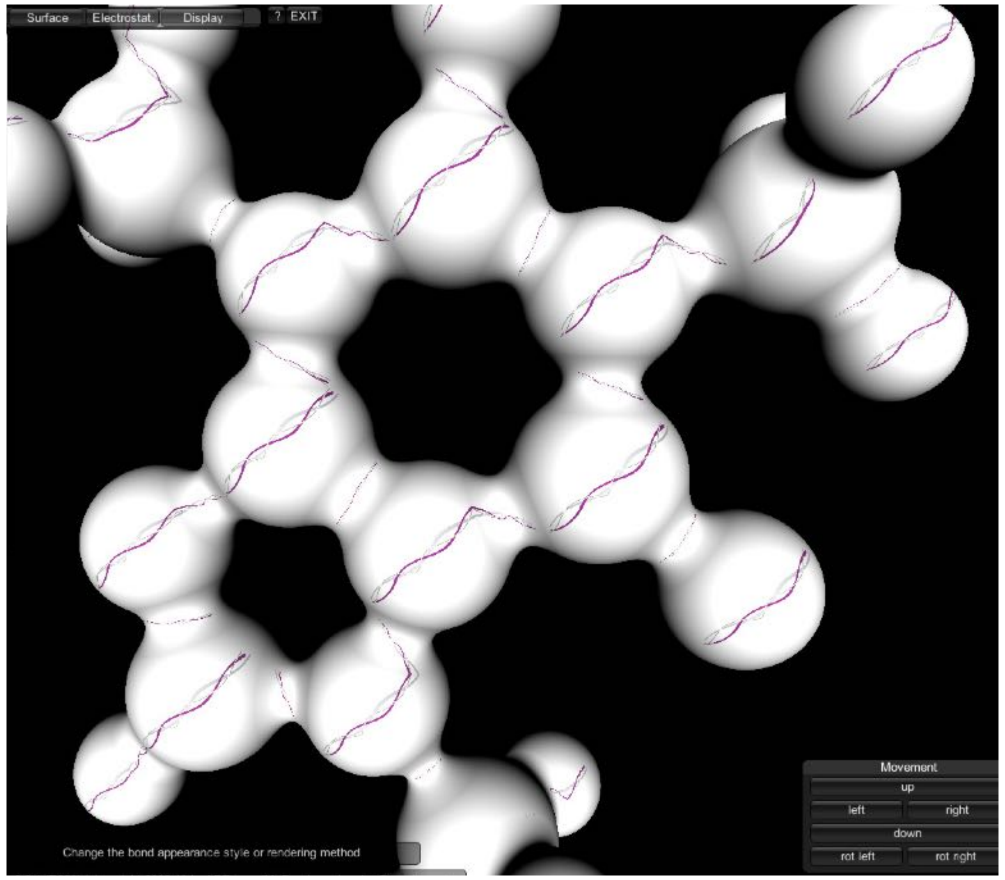

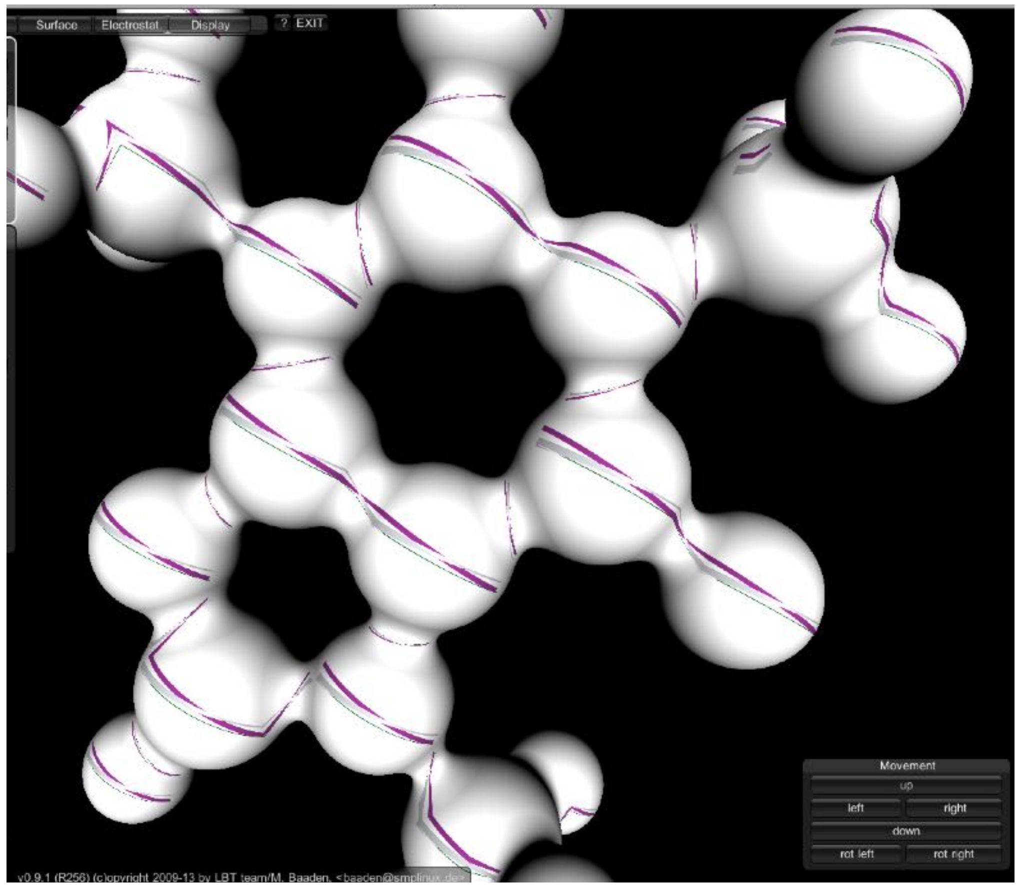

JULIE BORGESE

Charge/électronégativité

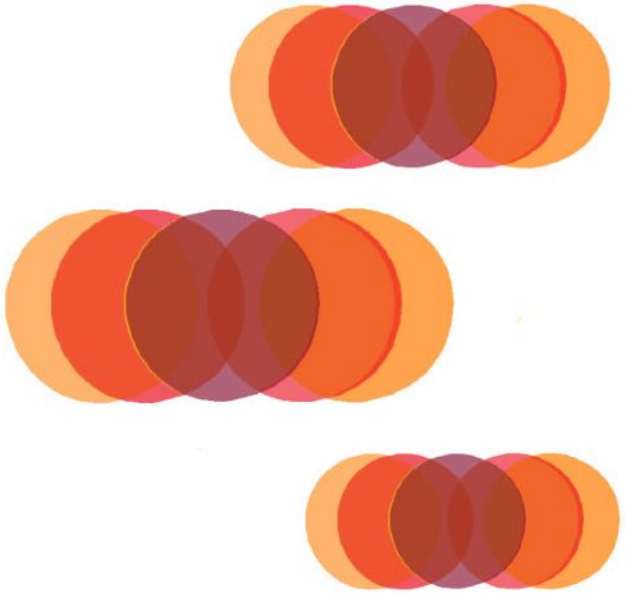

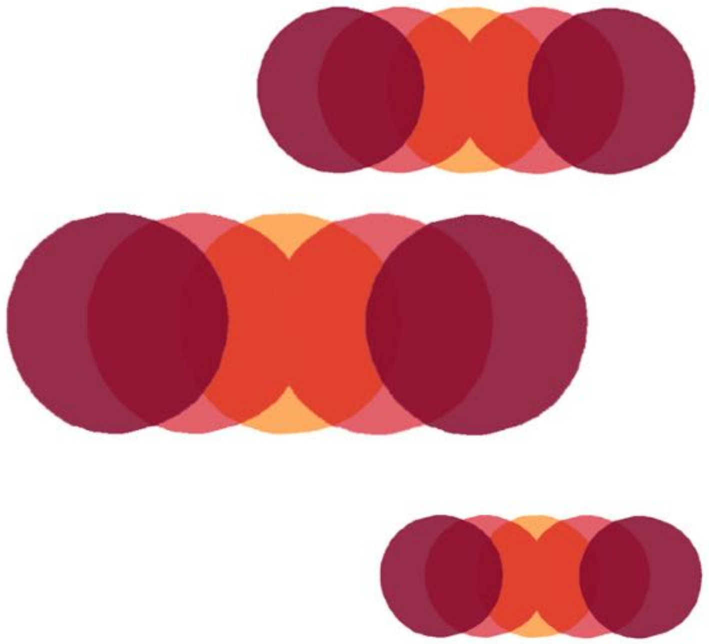

JULIE BORGESE

Visuel

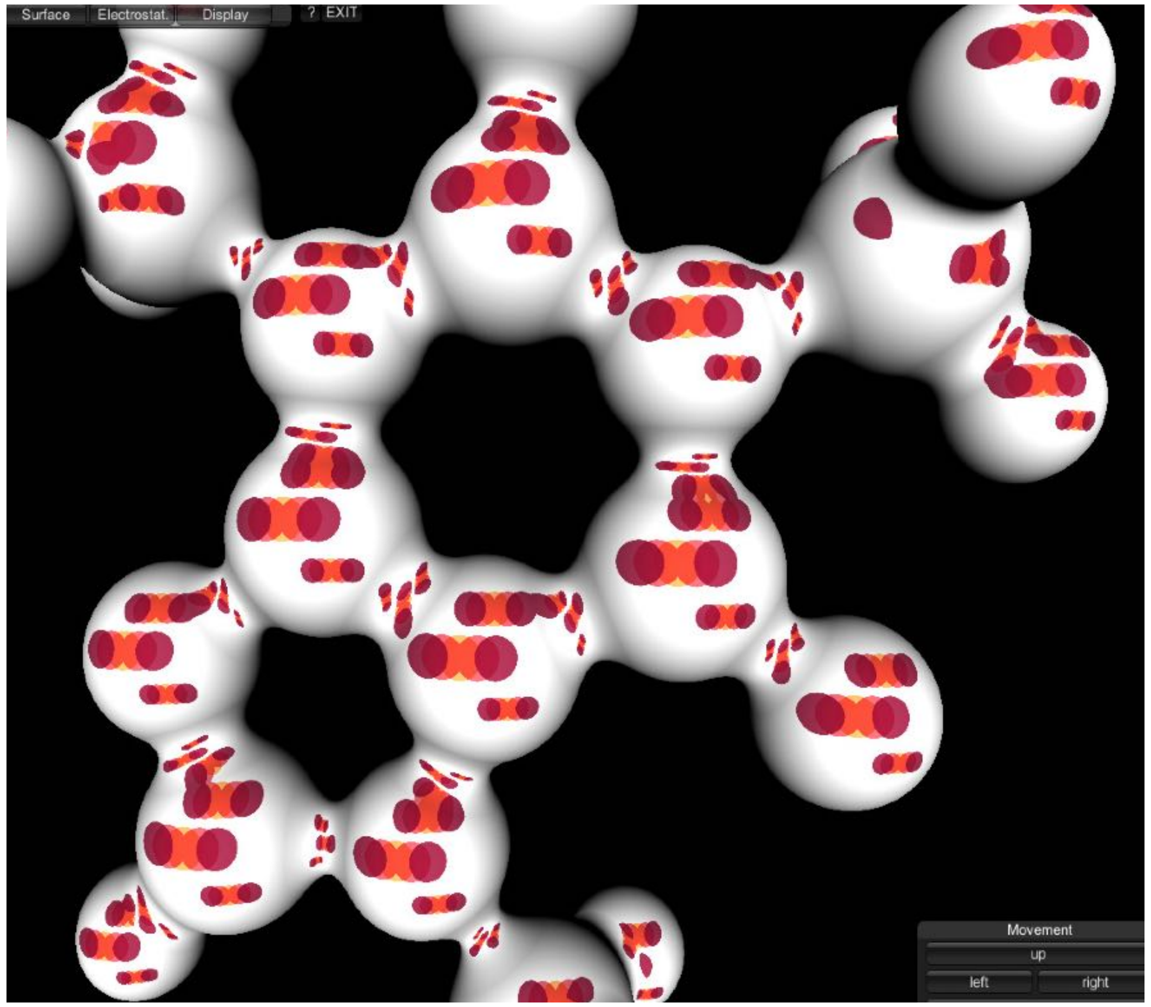

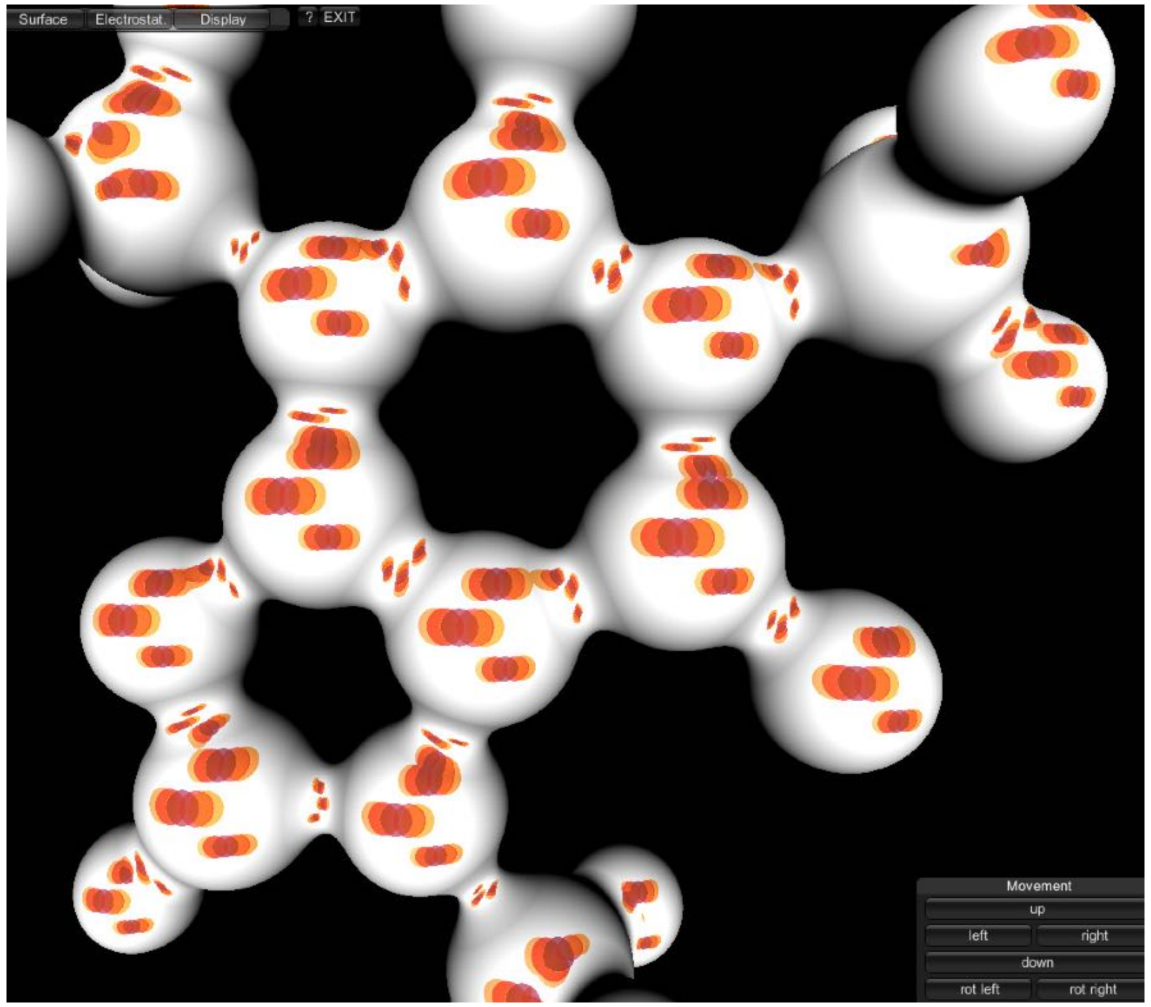

JULIE BORGESE

Hydrophobie/hydrophilie

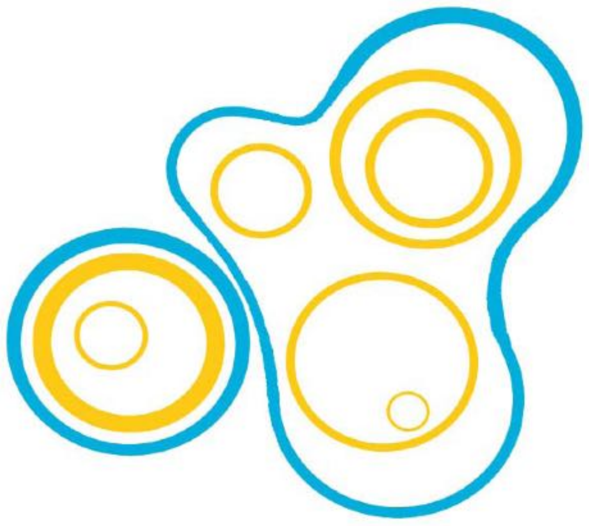

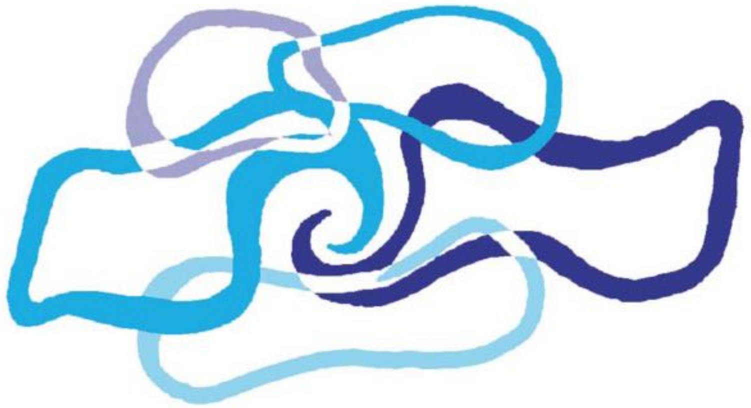

JULIE BORGESE

Visuel

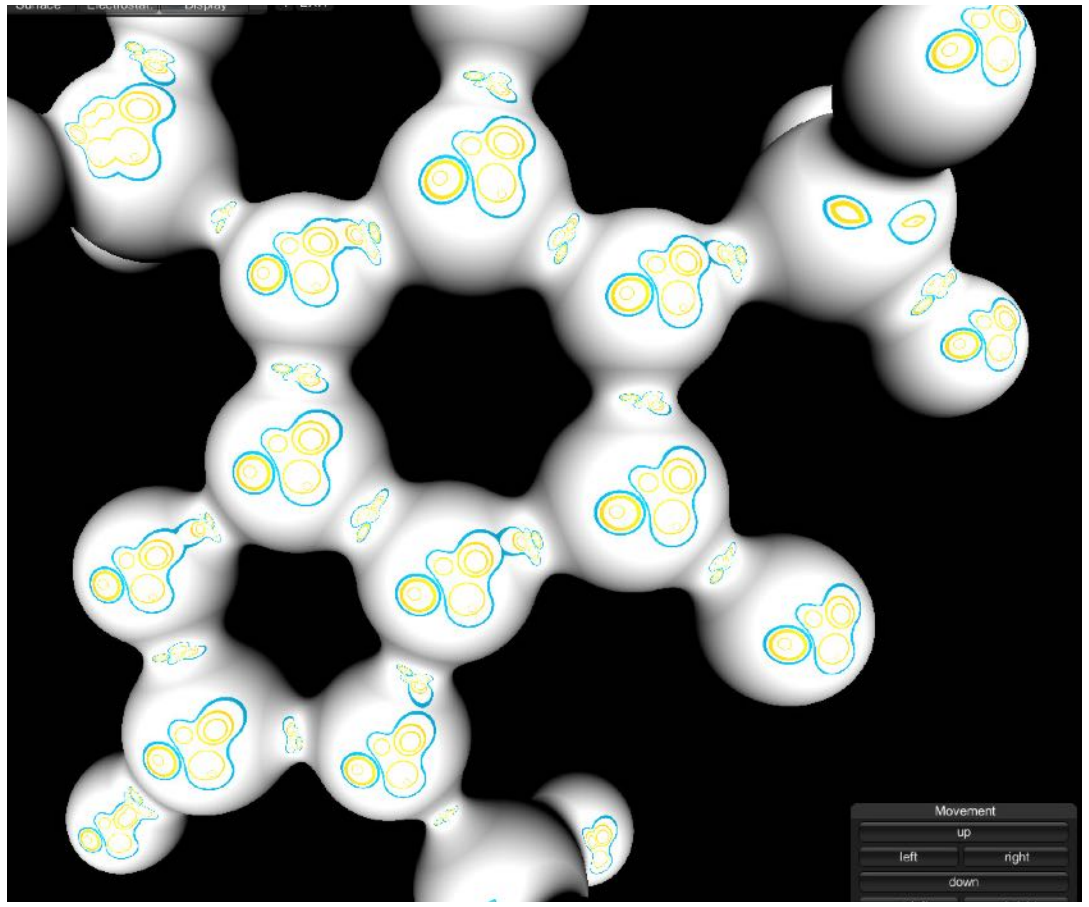

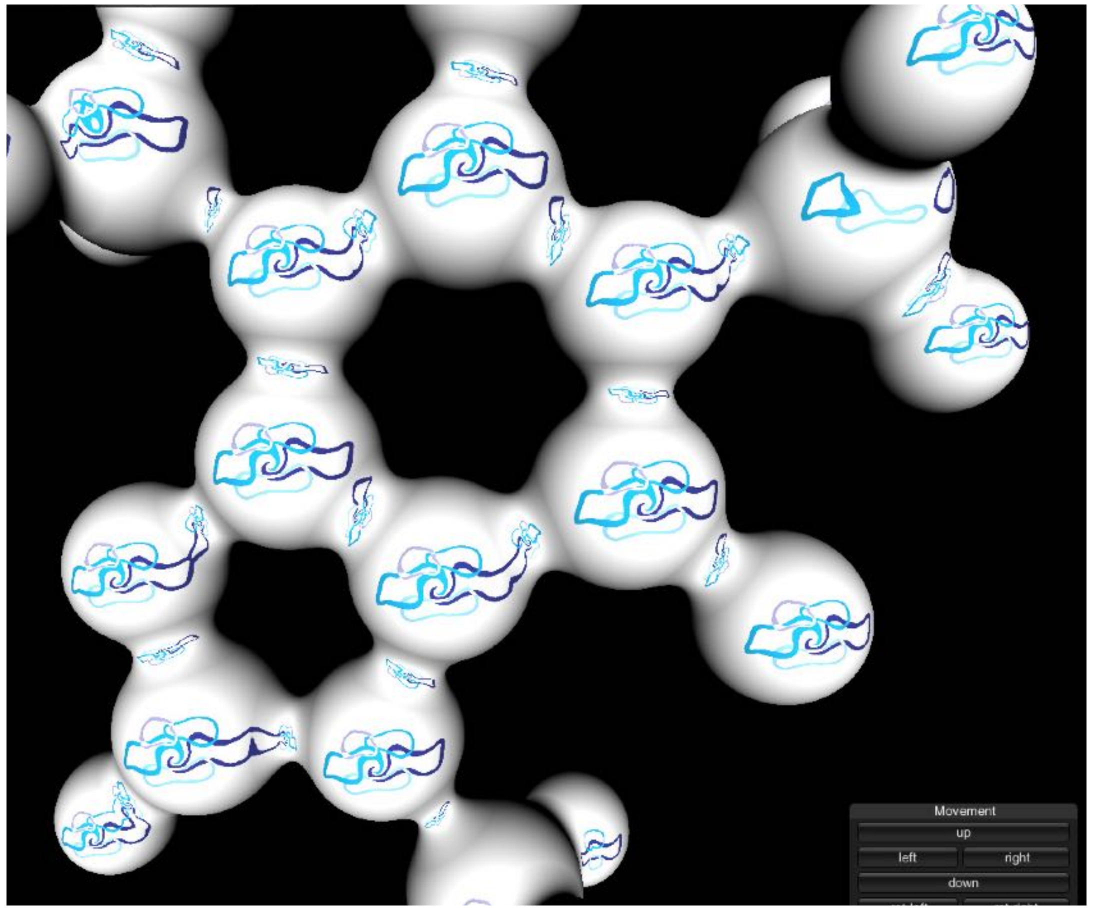

JULIE BORGESE

Bon/mauvais

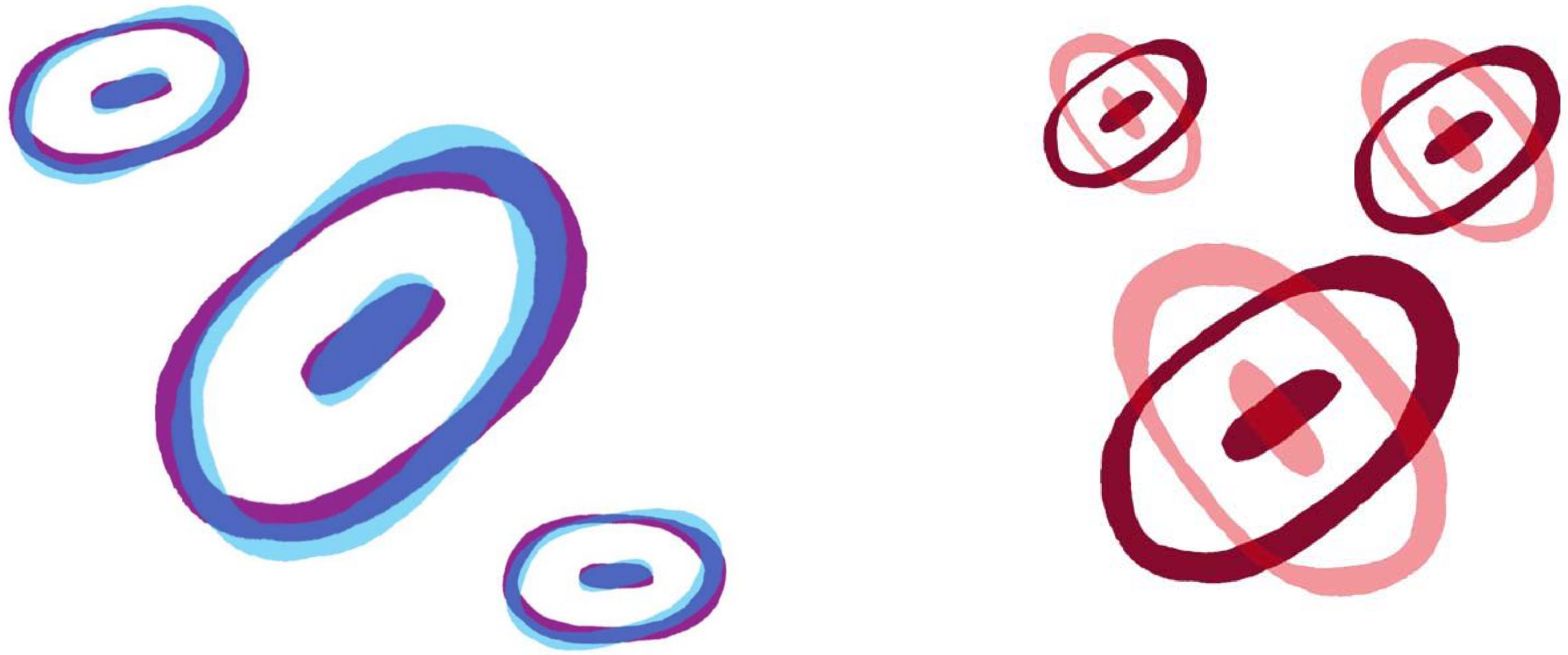

JULIE BORGESE

Visuel

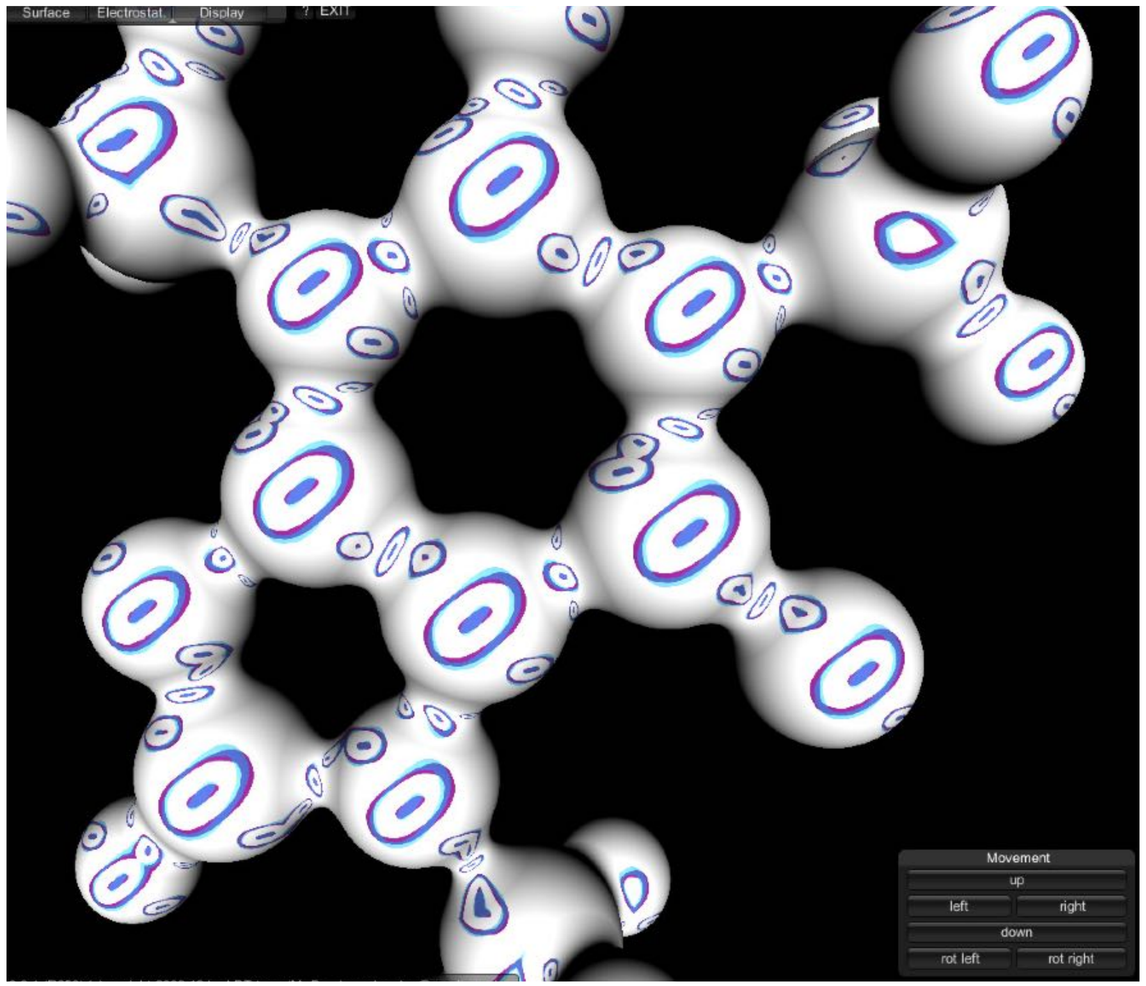

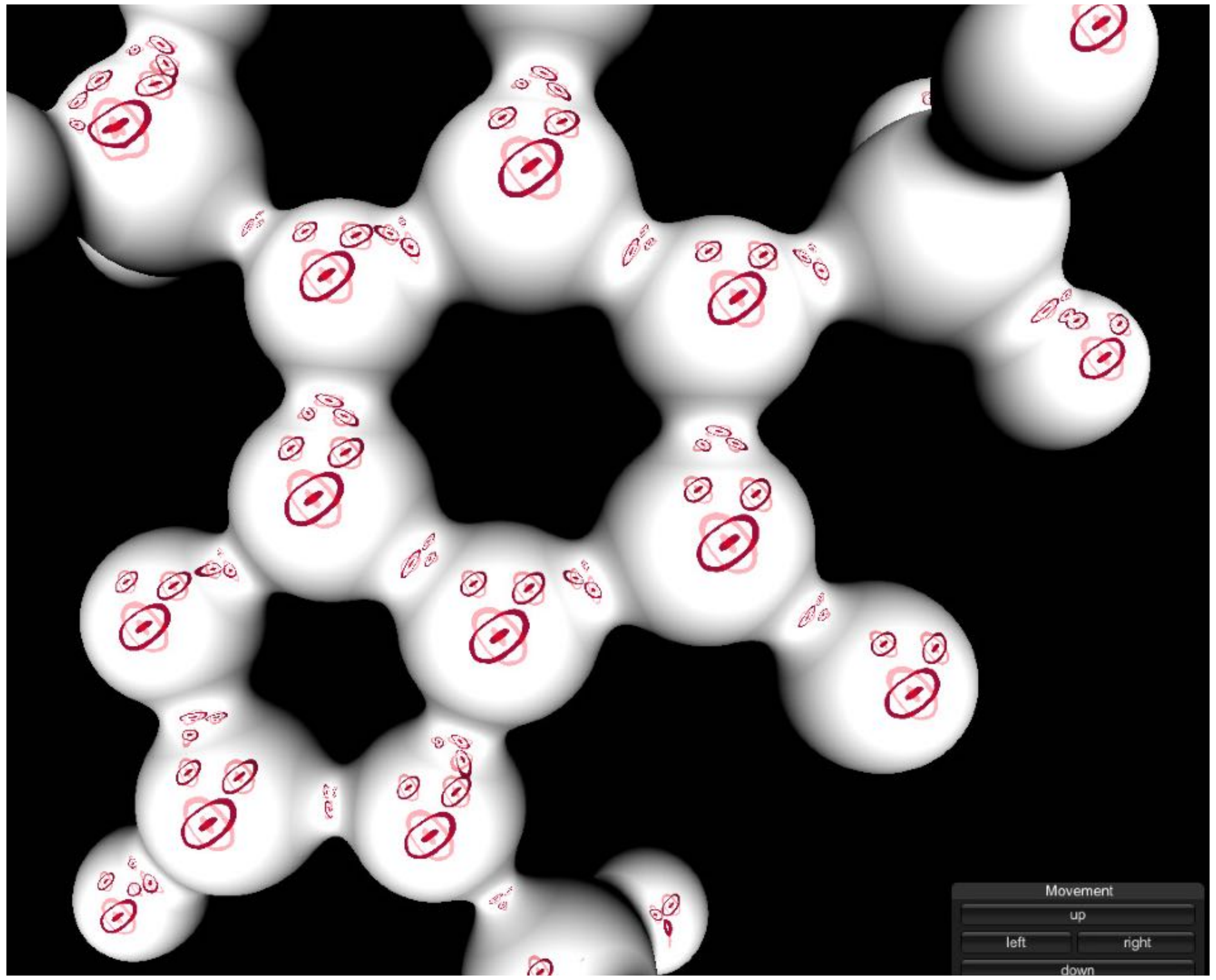

JULIE BORGESE

Site actif/structure en dehors du site

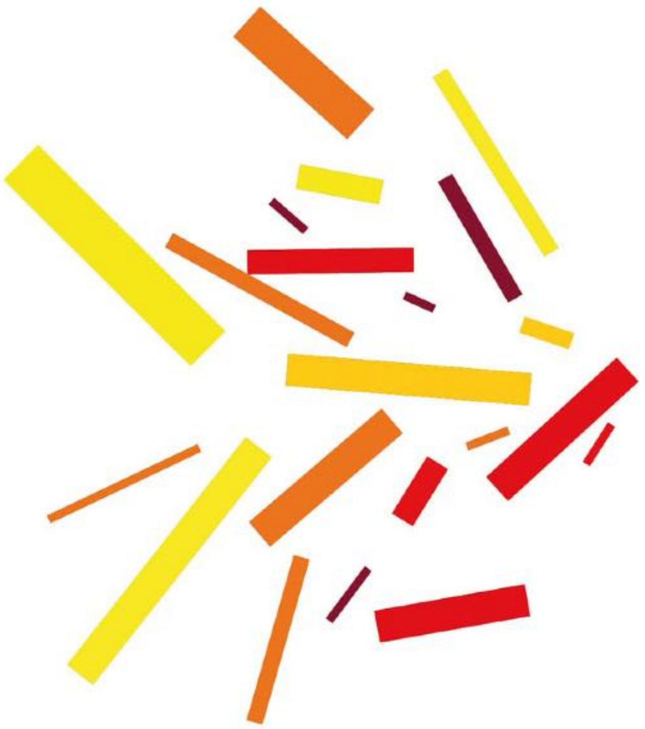

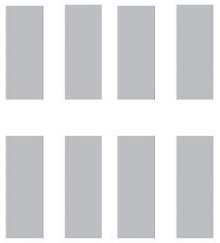

JULIE BORGESE

Visuel

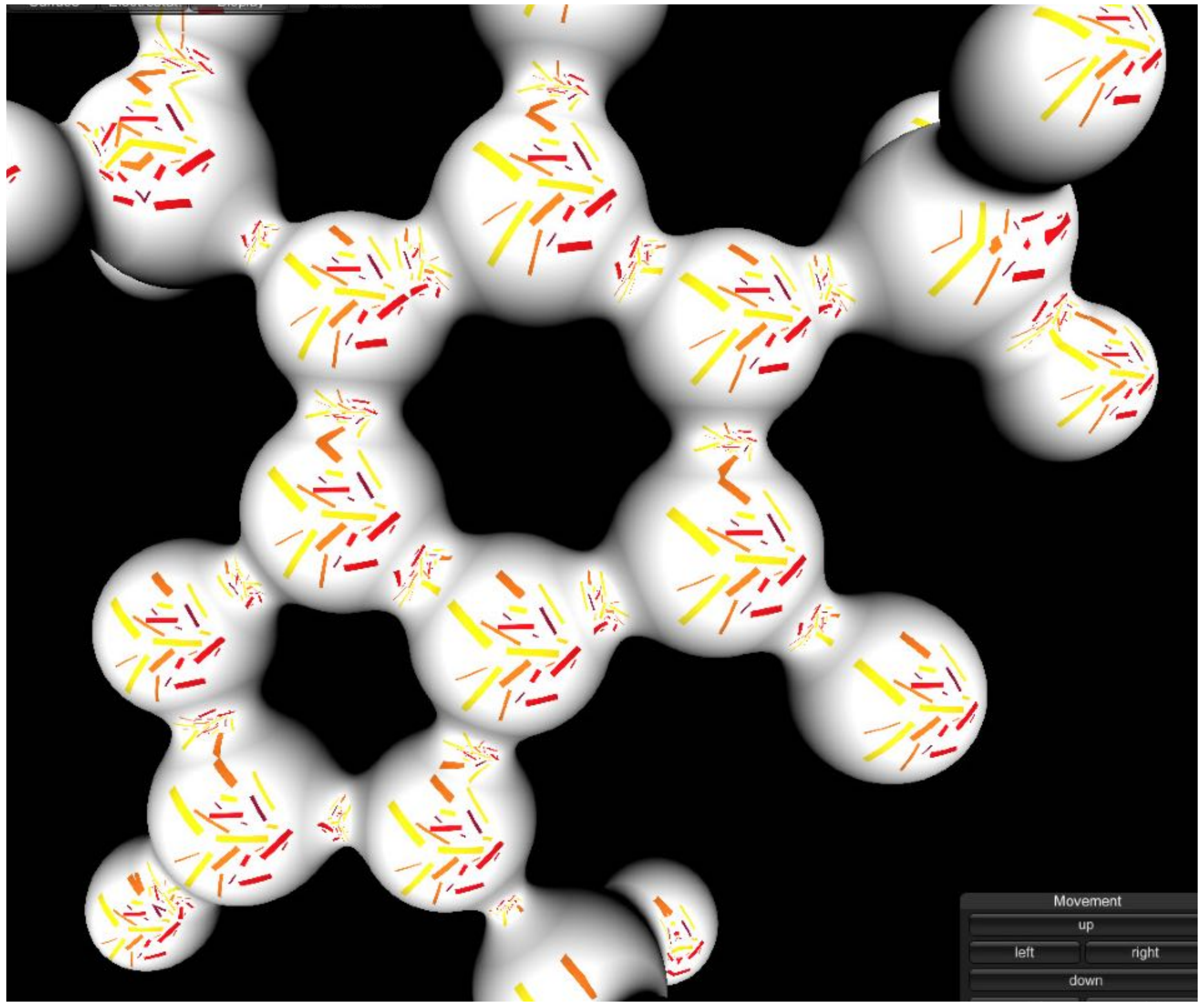

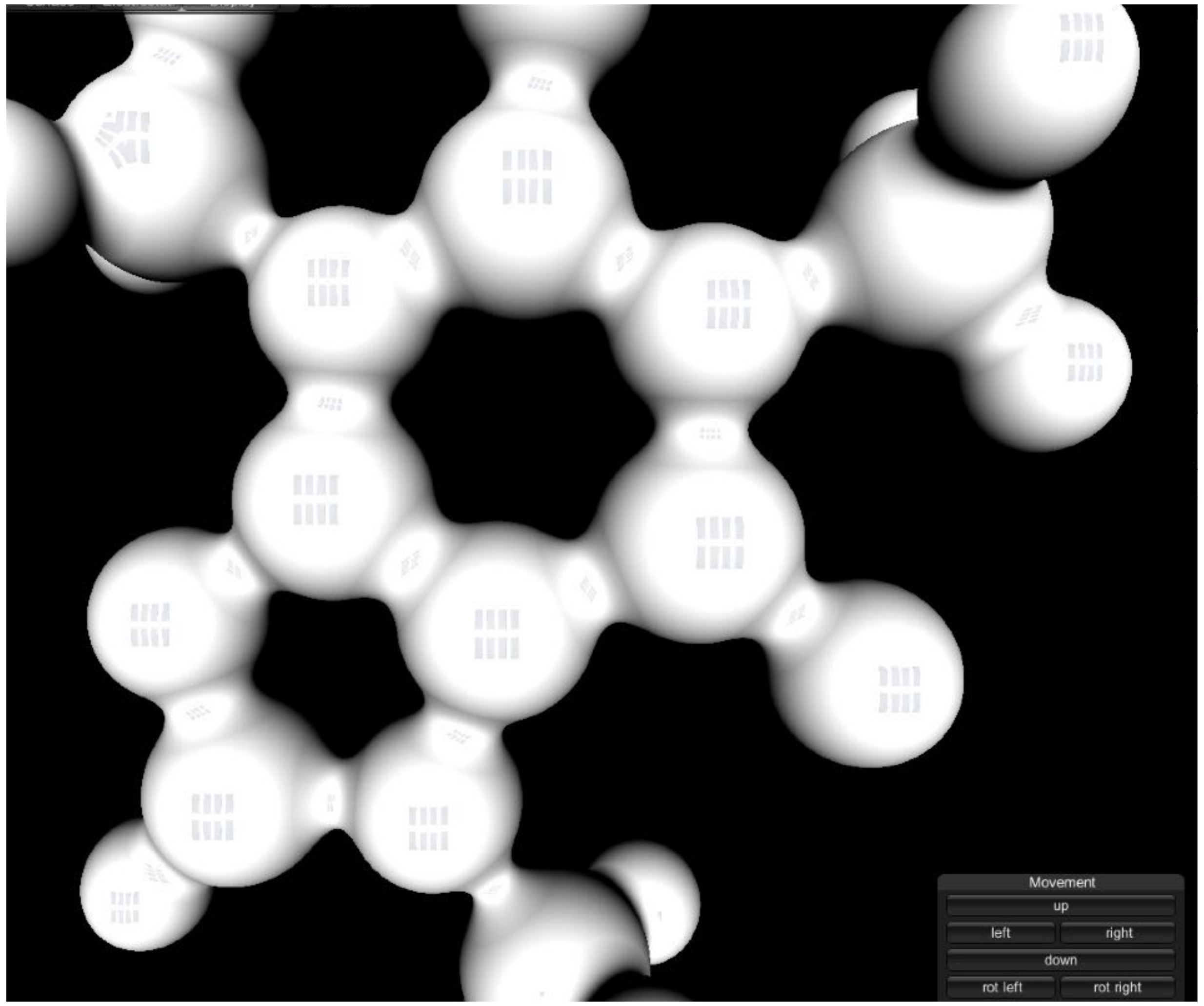

JULIE BORGESE

Toxicité/écocompatibilité

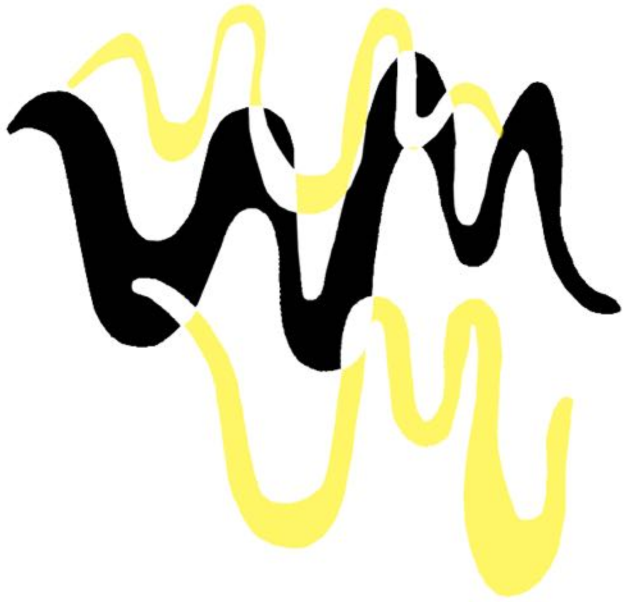

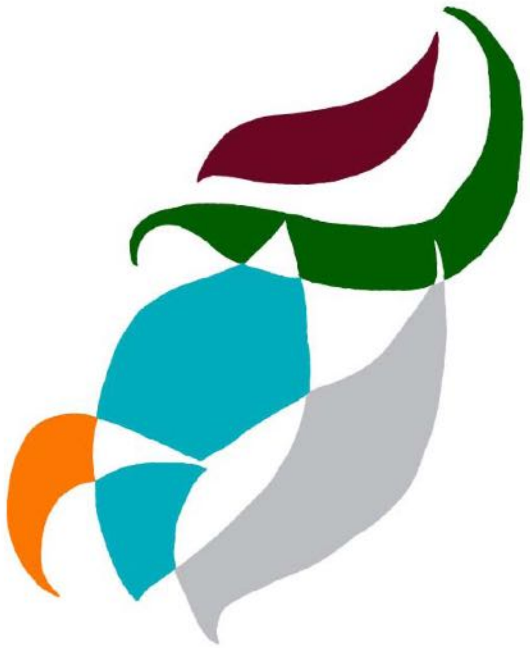

JULIE BORGESE

Visuel

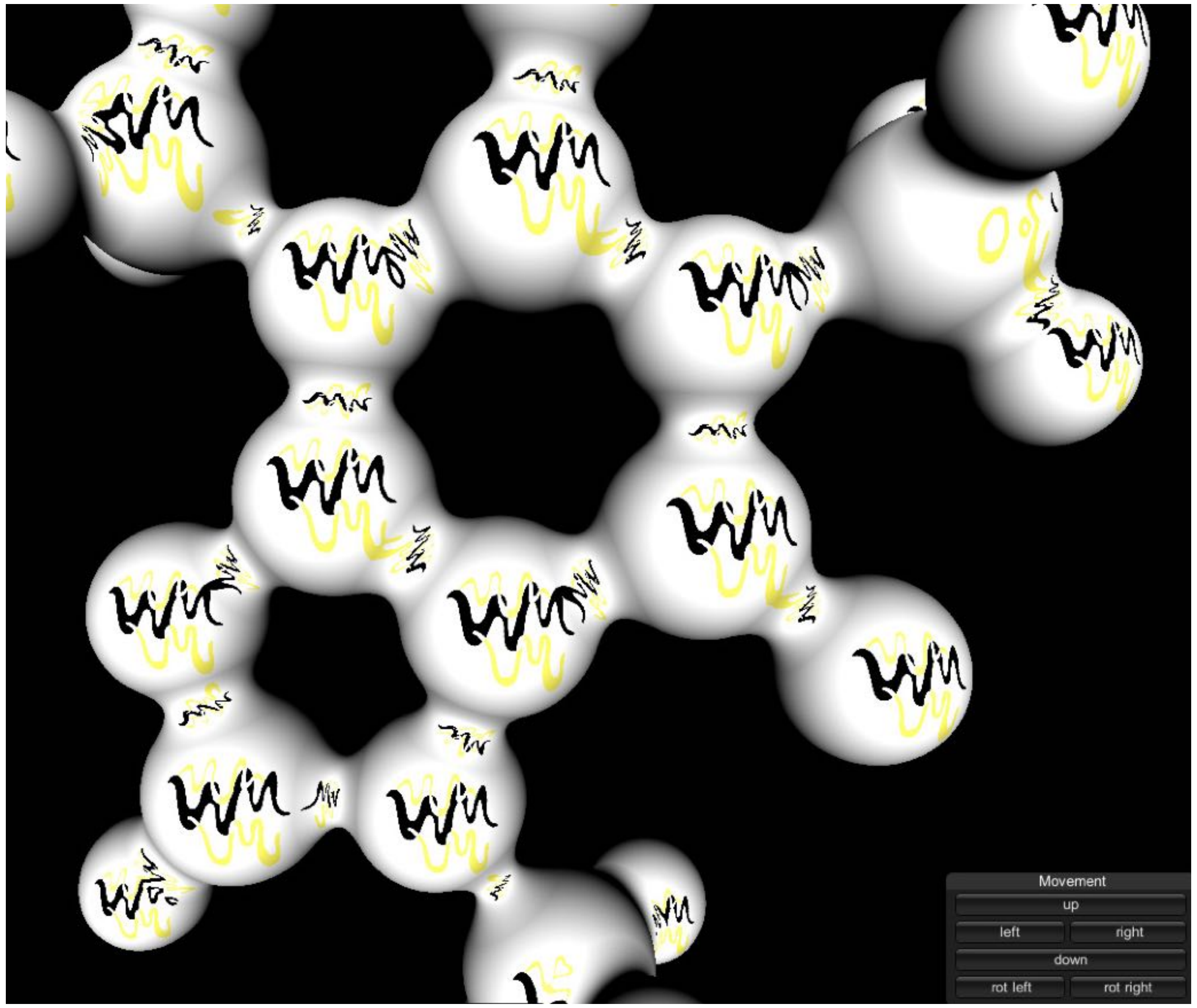

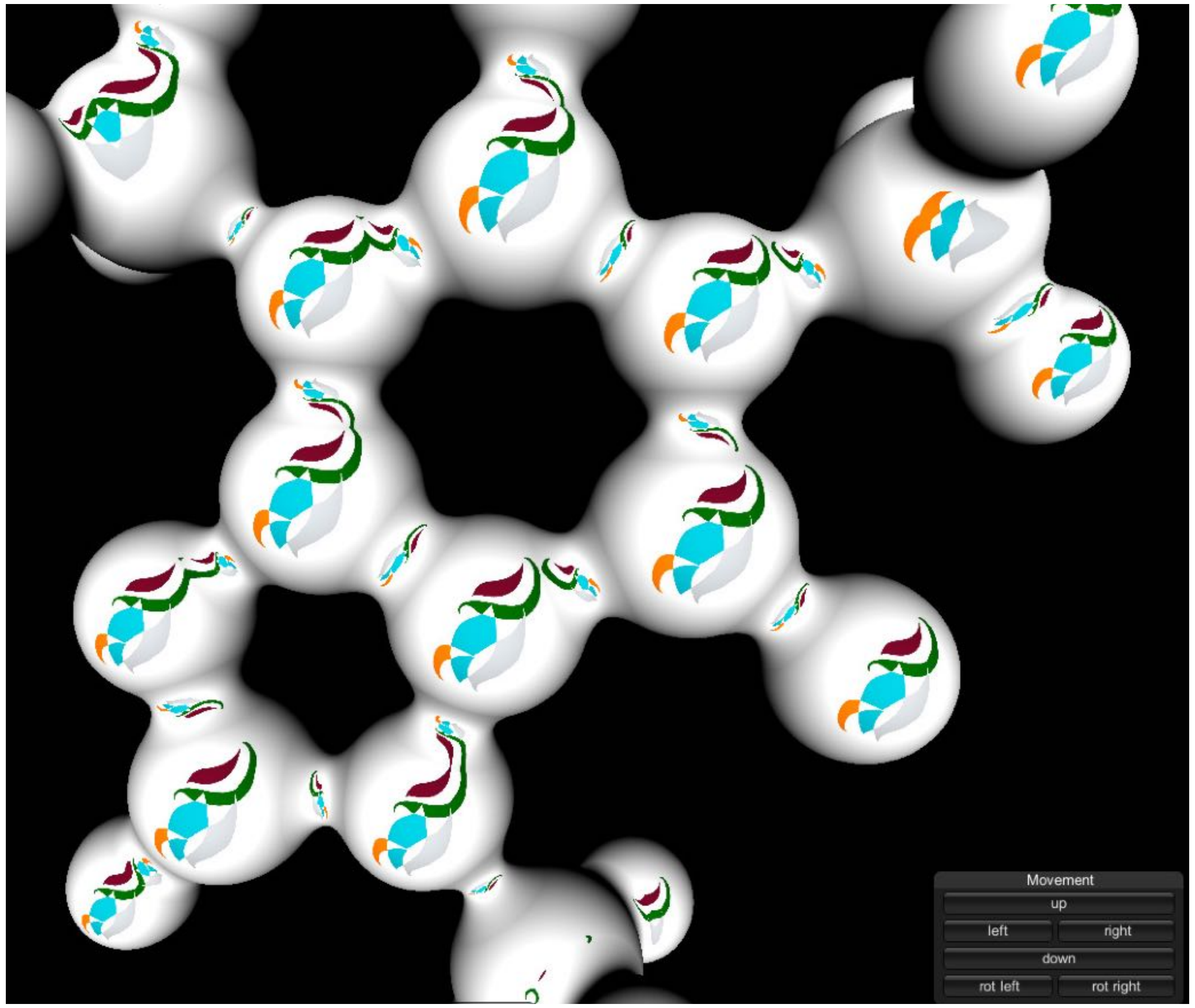

JULIE BORGESE

Sucre/Graisse

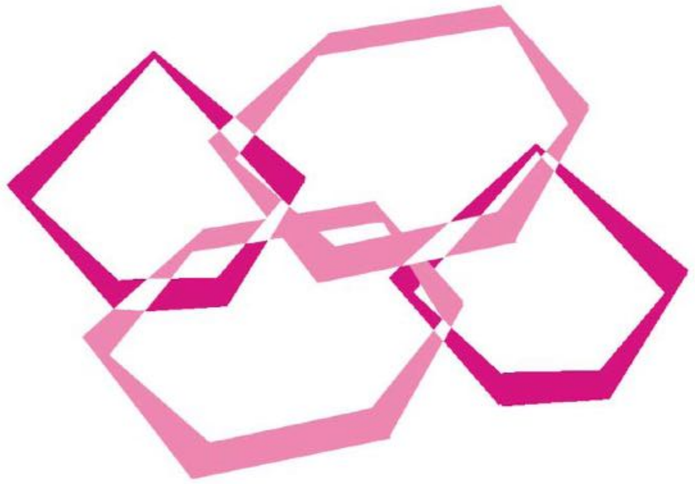

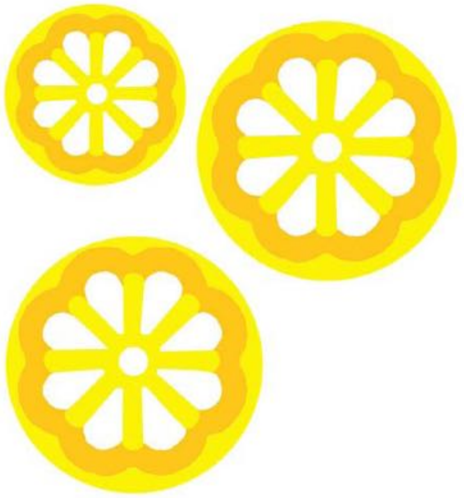

JULIE BORGESE

Visuel

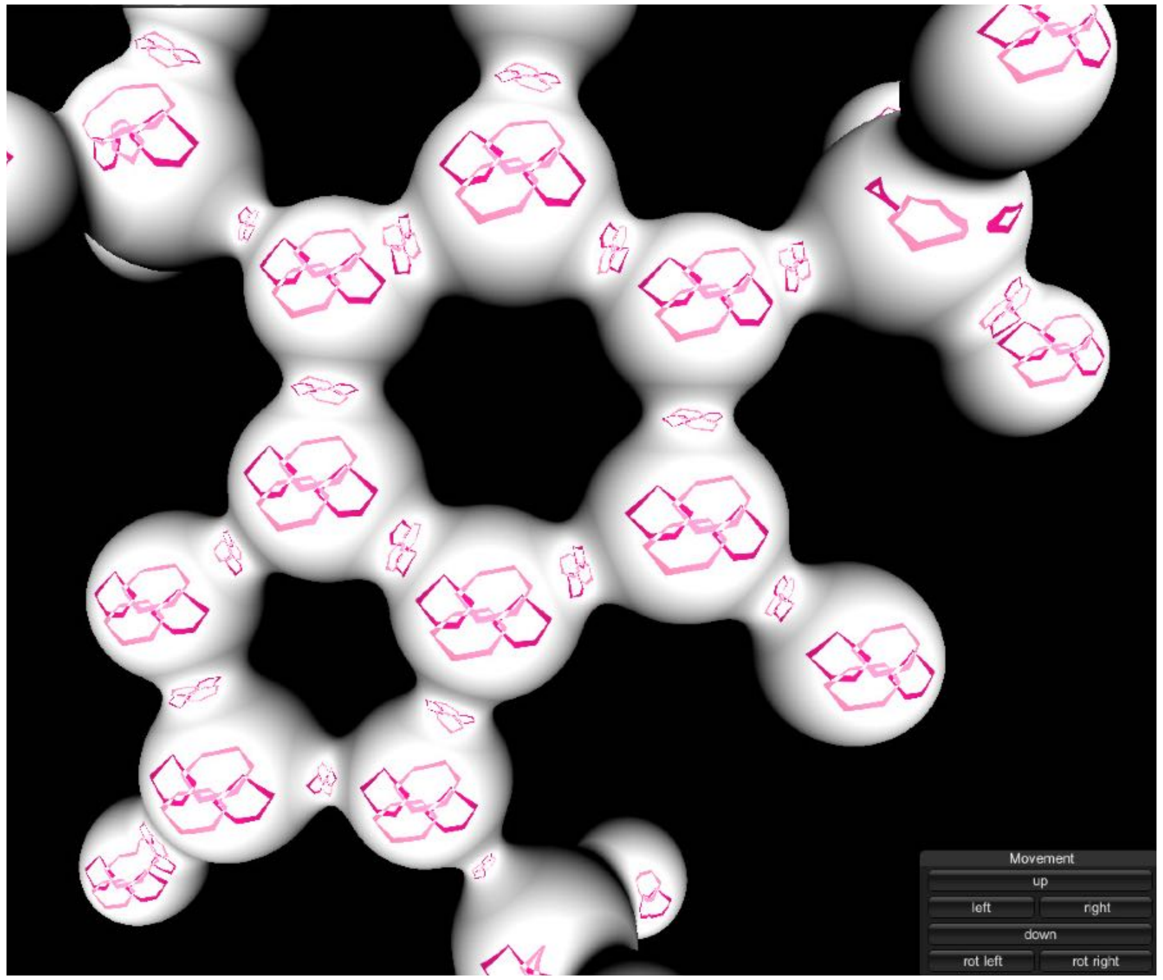

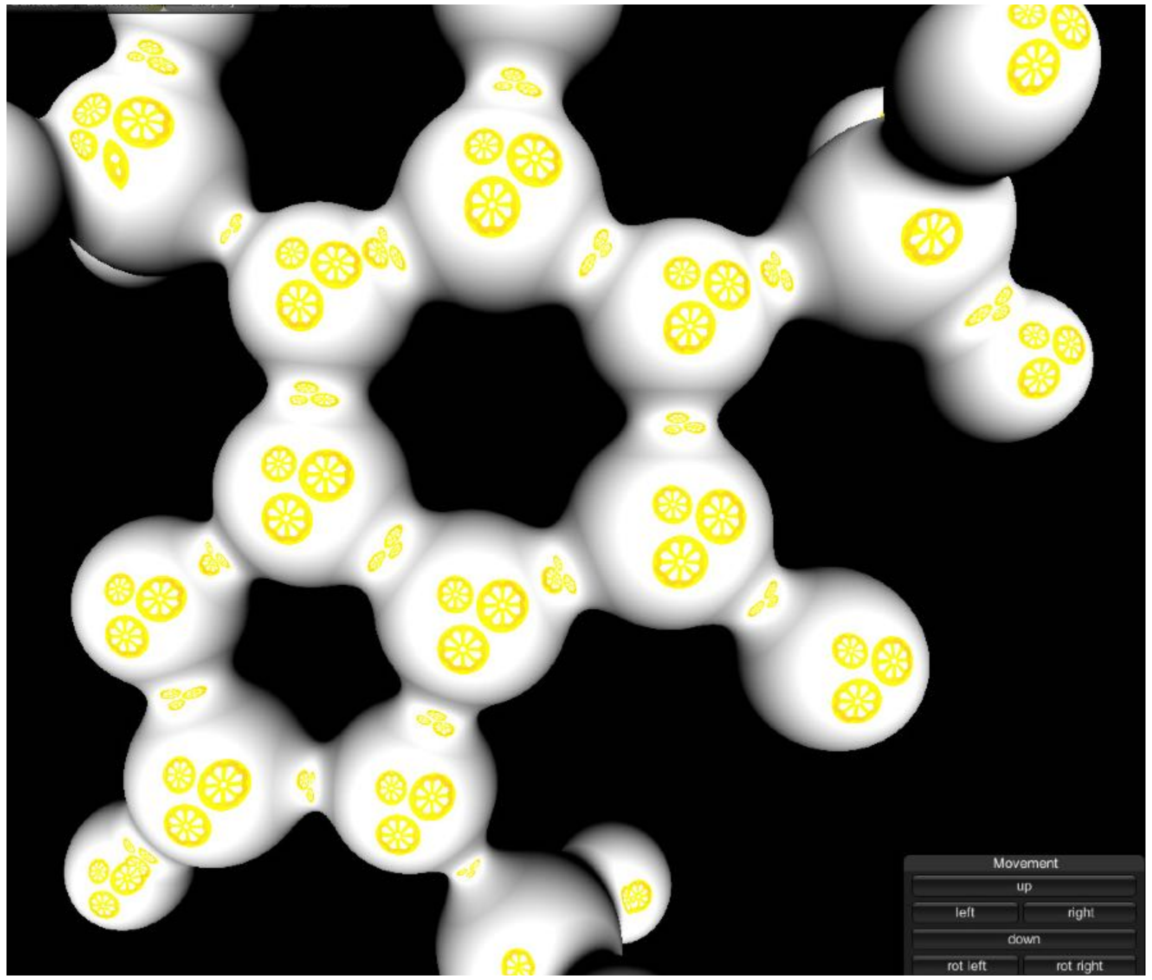

JULIE BORGESE

Azote/Titane

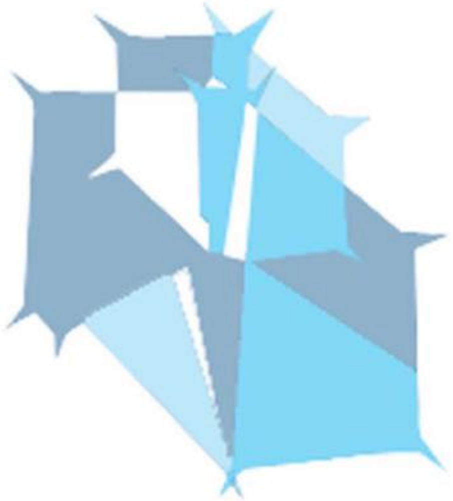

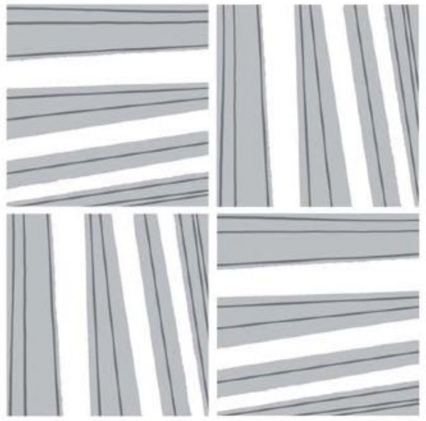

JULIE BORGESE

Visuel

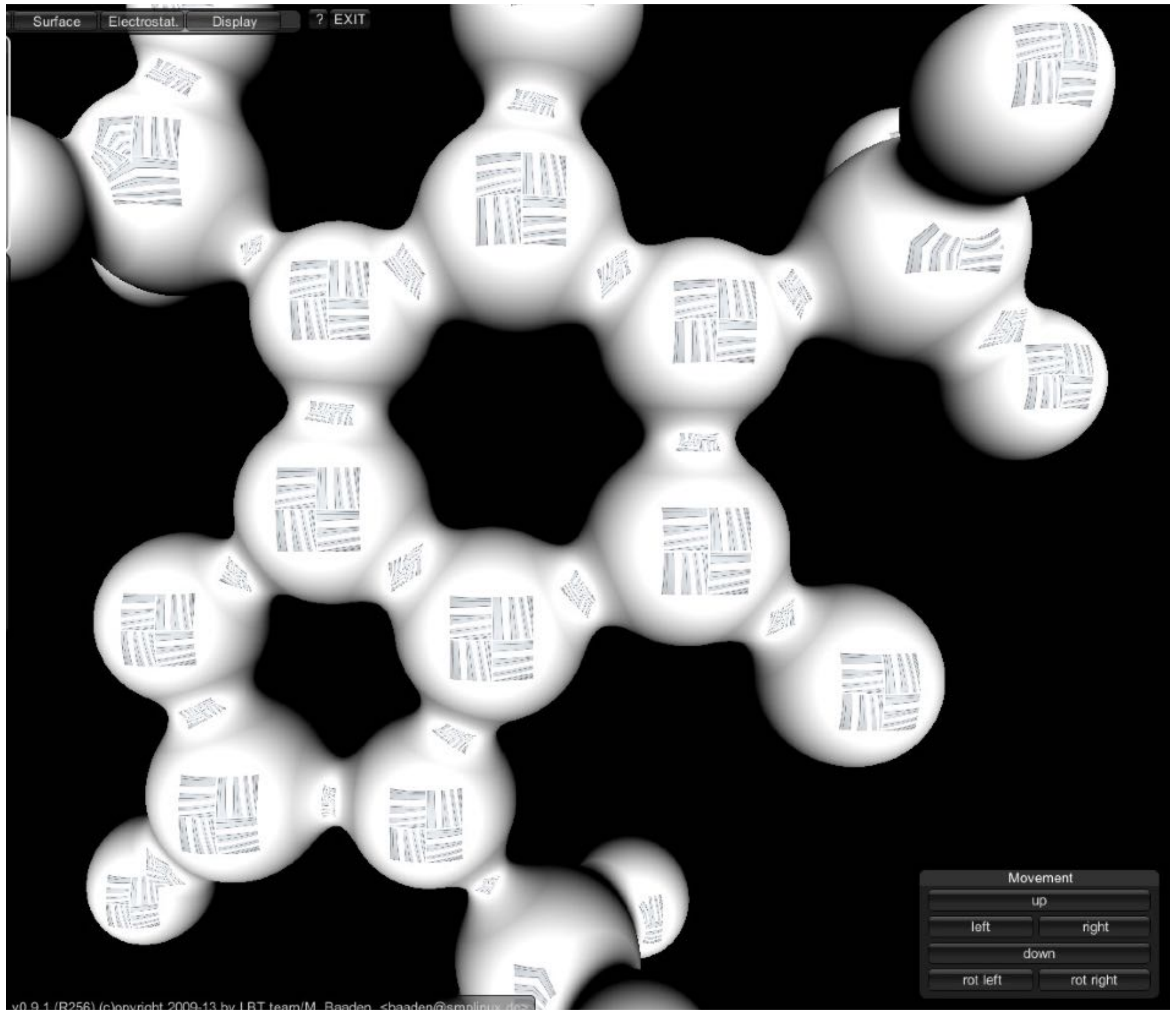

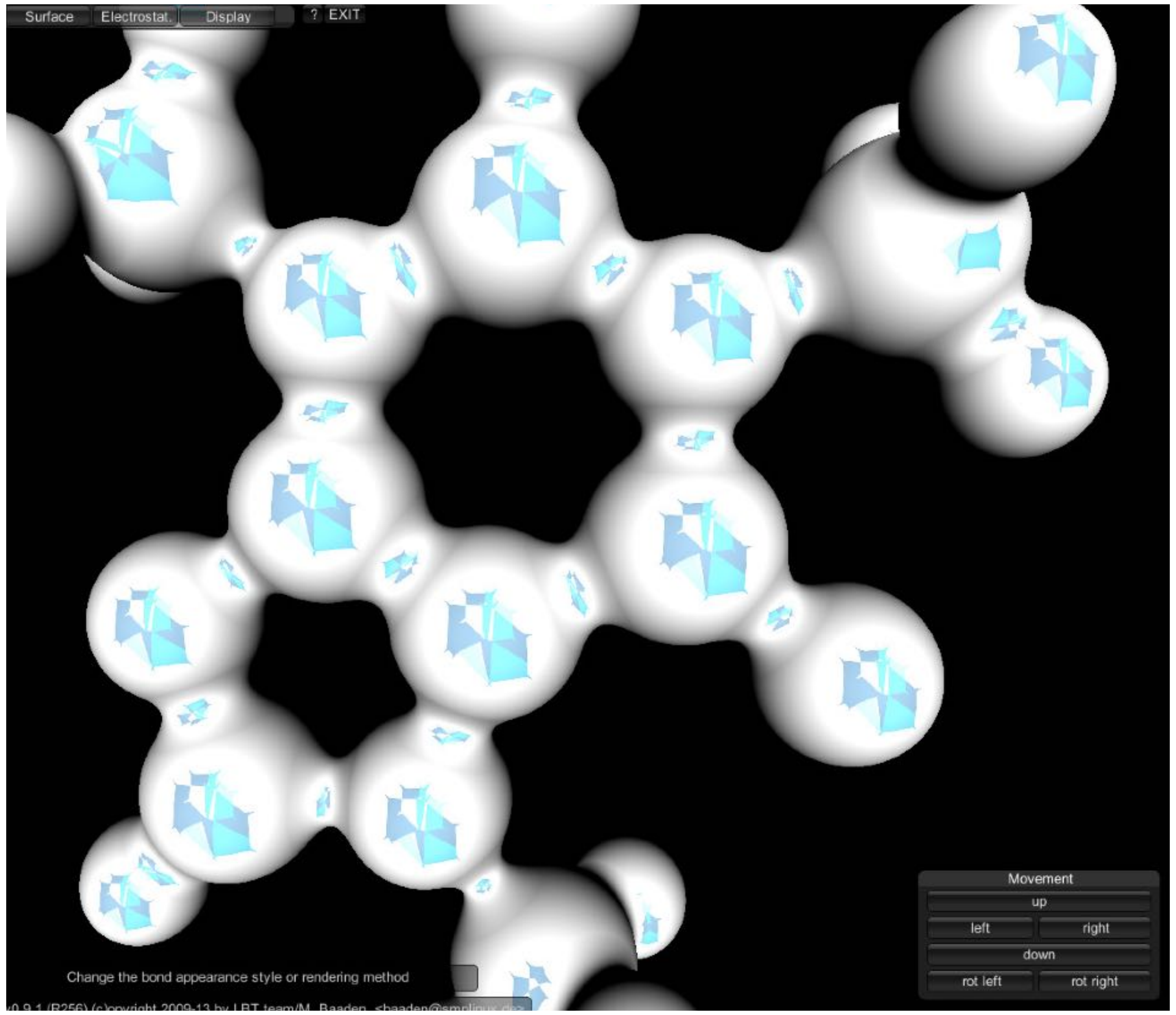

JULIE BORGESE

Principe de gradation

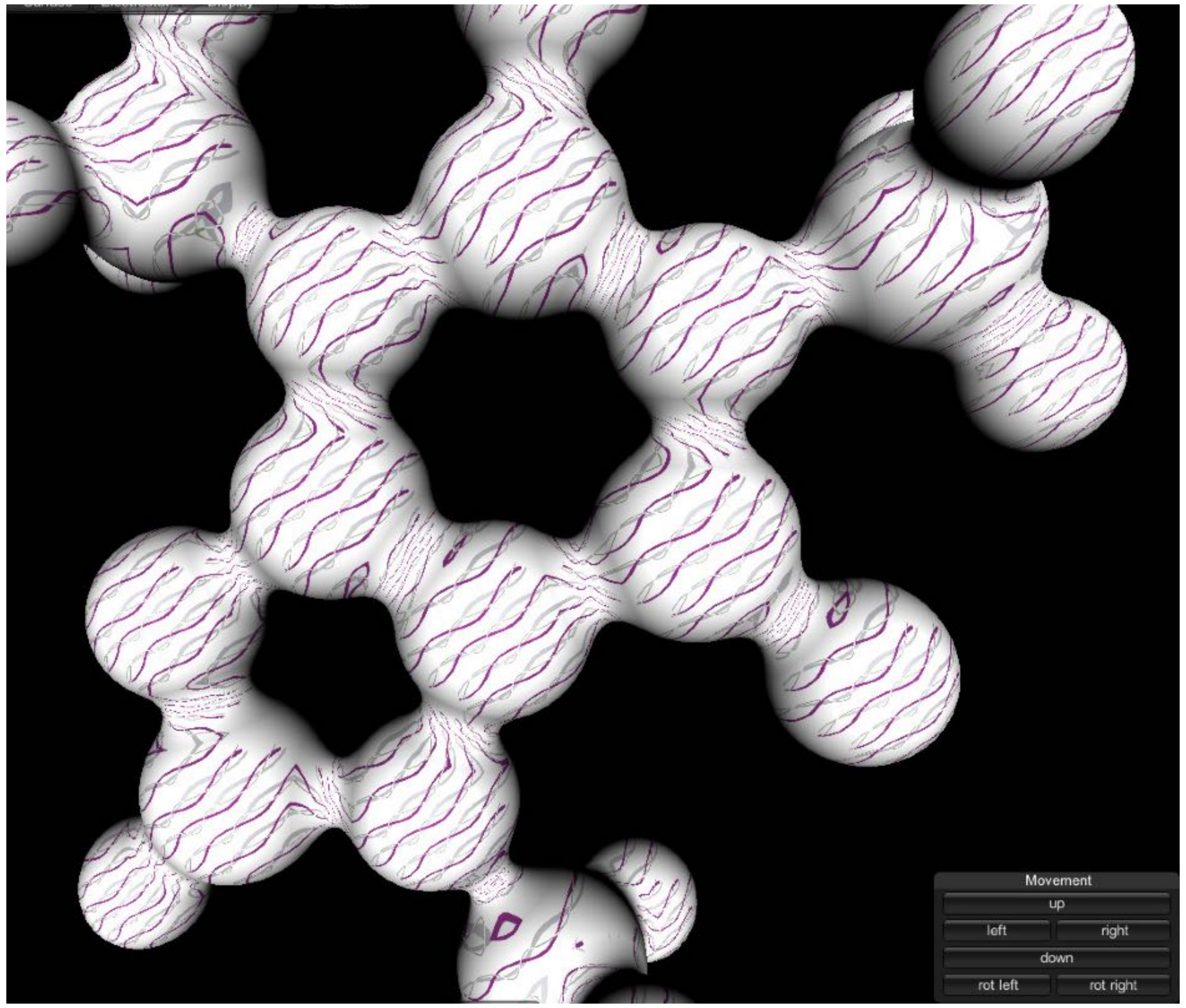

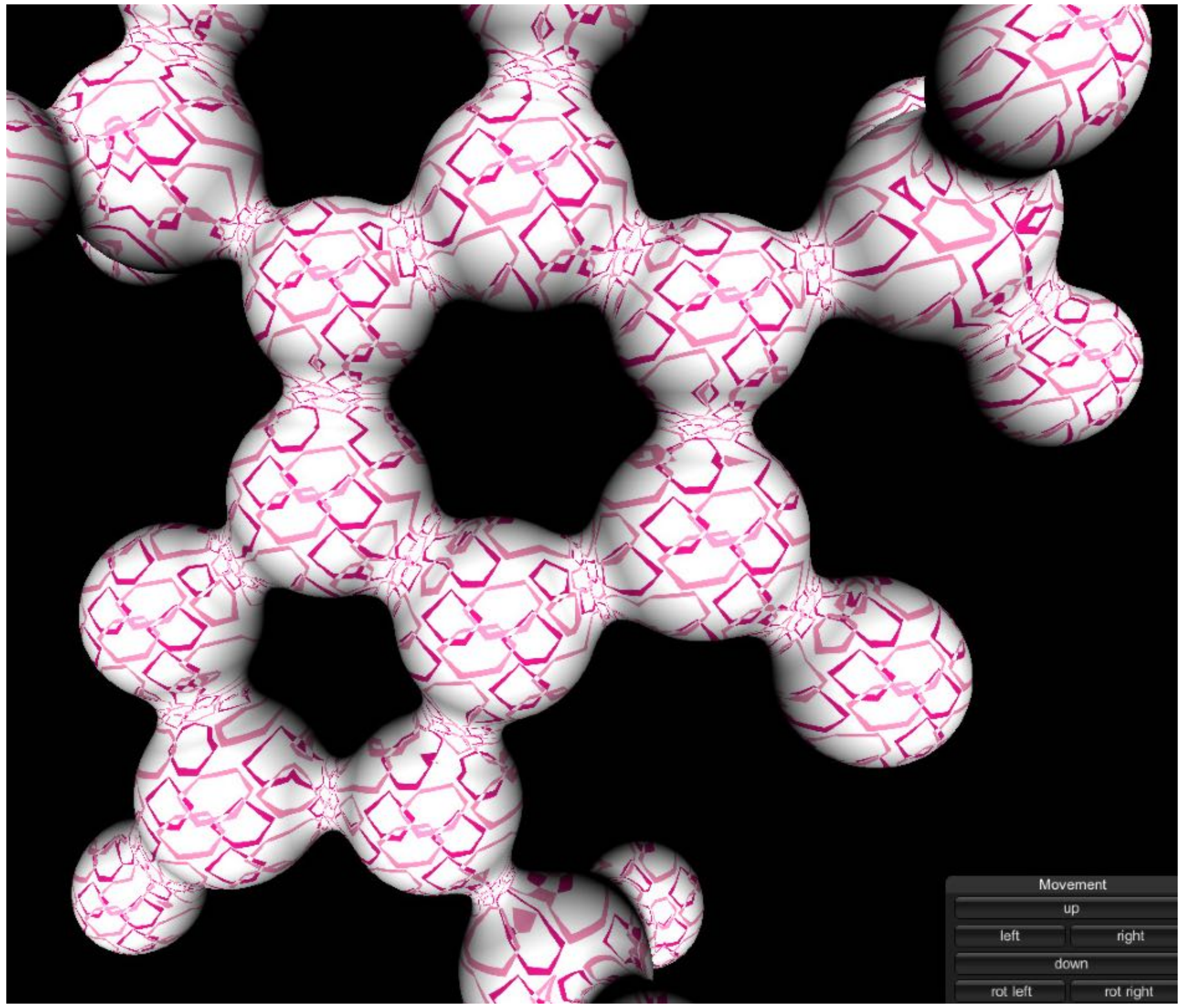

Merci !

Bonnes Fetes !

JULIE BORGESE

Flexibilité/rigidité

LIT-SPHÈRES

RENDU ARTISTIQUE DE PROPRIÉTÉS MOLÉCULAIRES

Elsa DEPONT

ELSA DEPONT

DATAVIZ

LEXIQUE DE FORMES

HYDROPHOBIE :

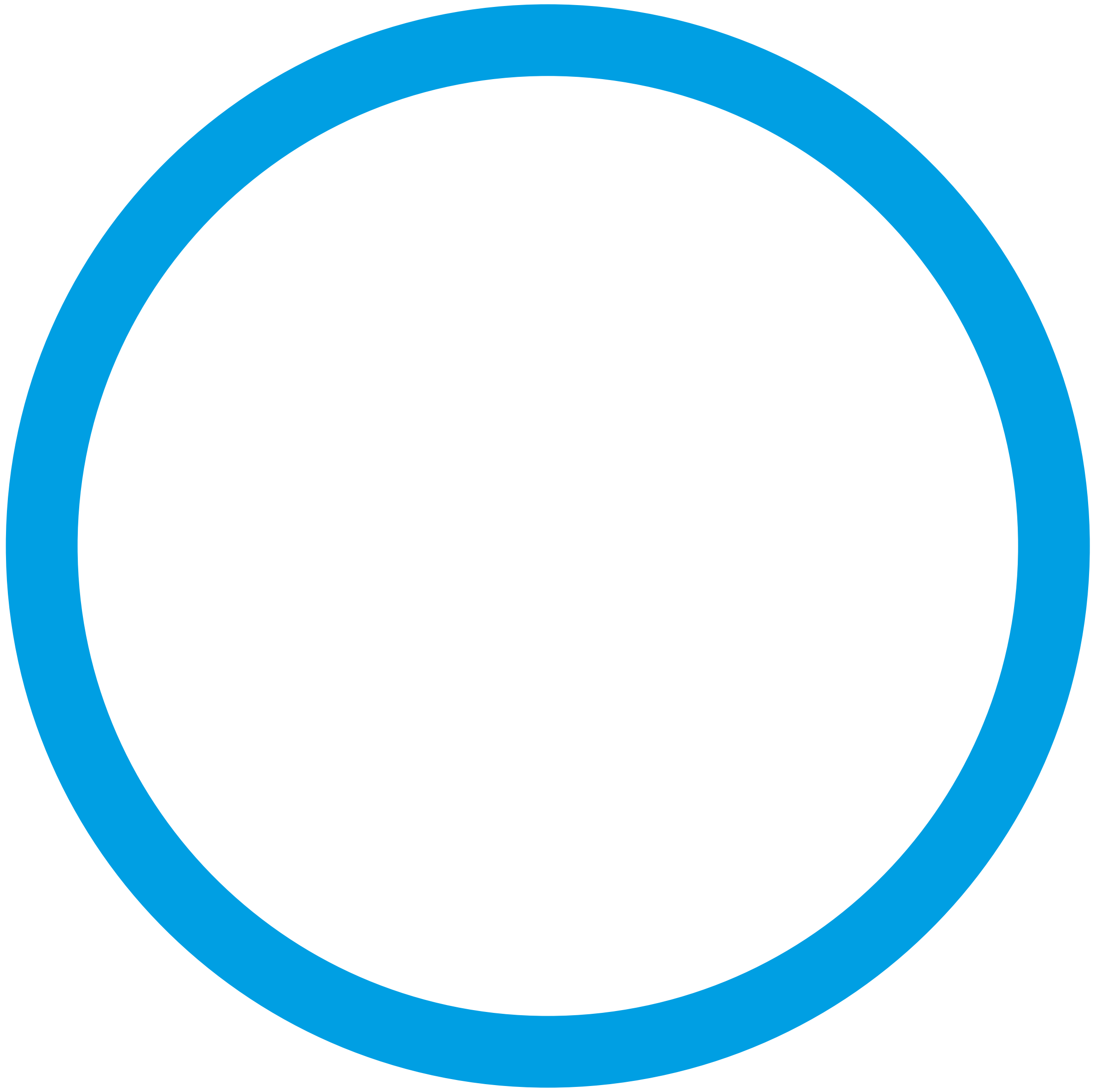

LIT-SPHÈRES
RENDU ARTISTIQUE DE PROPRIÉTÉS MOLÉCULAIRES

ELSA DEPONT

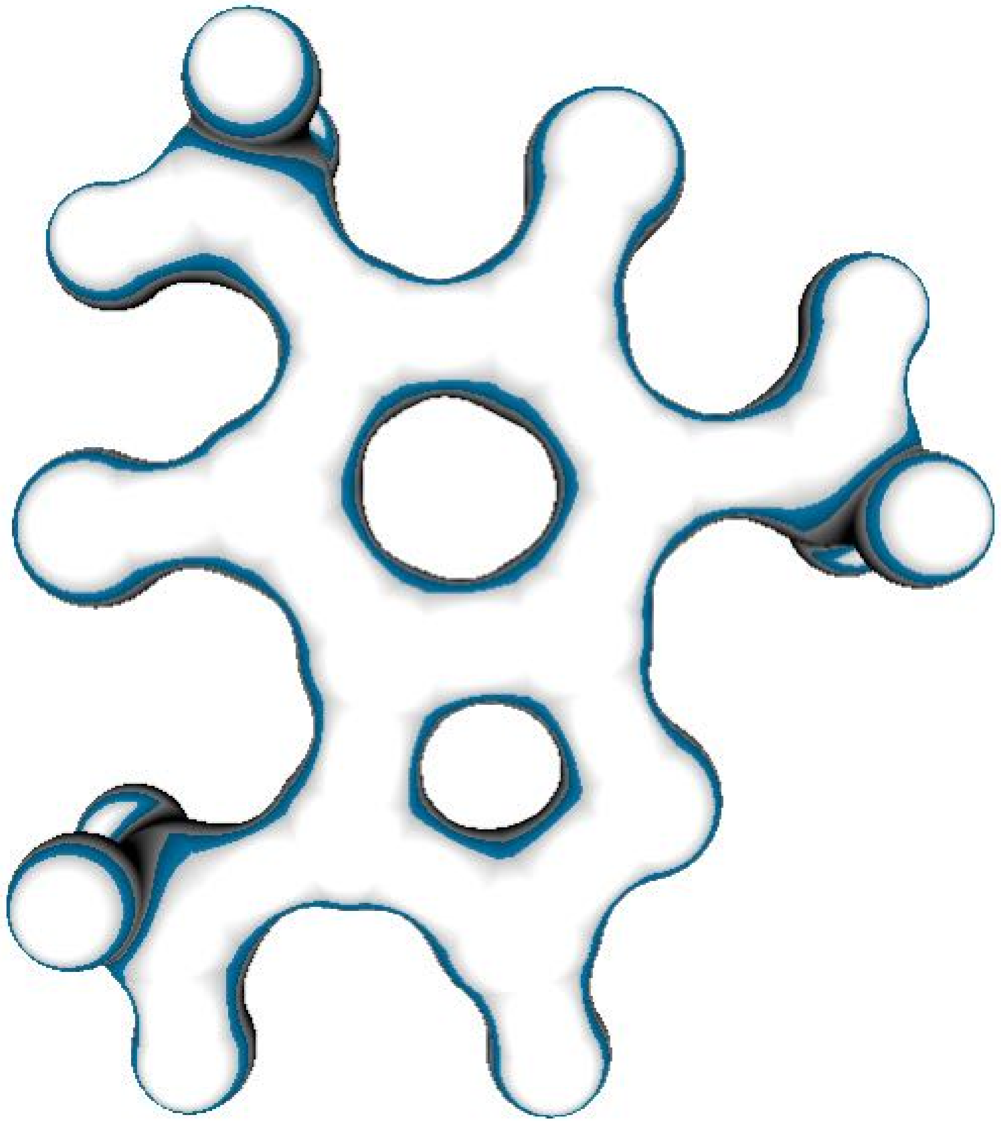

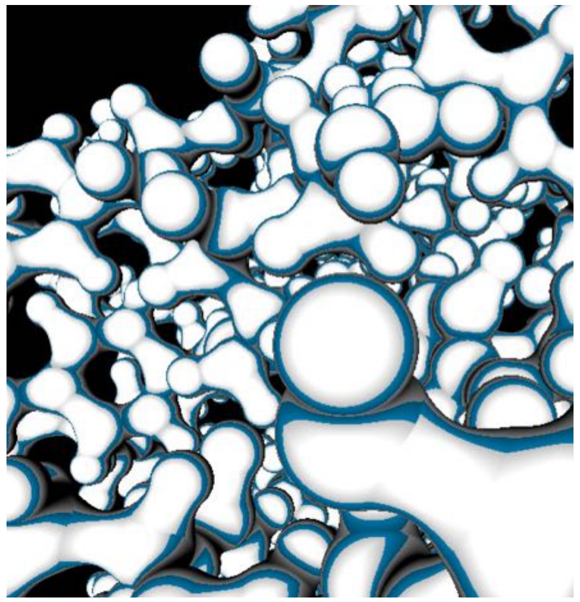

LIT-SPHÈRES
RENDU ARTISTIQUE DE
PROPRIÉTÉS MOLÉCULAIRES

ELSA DEPONT

HYDROPHILIE :

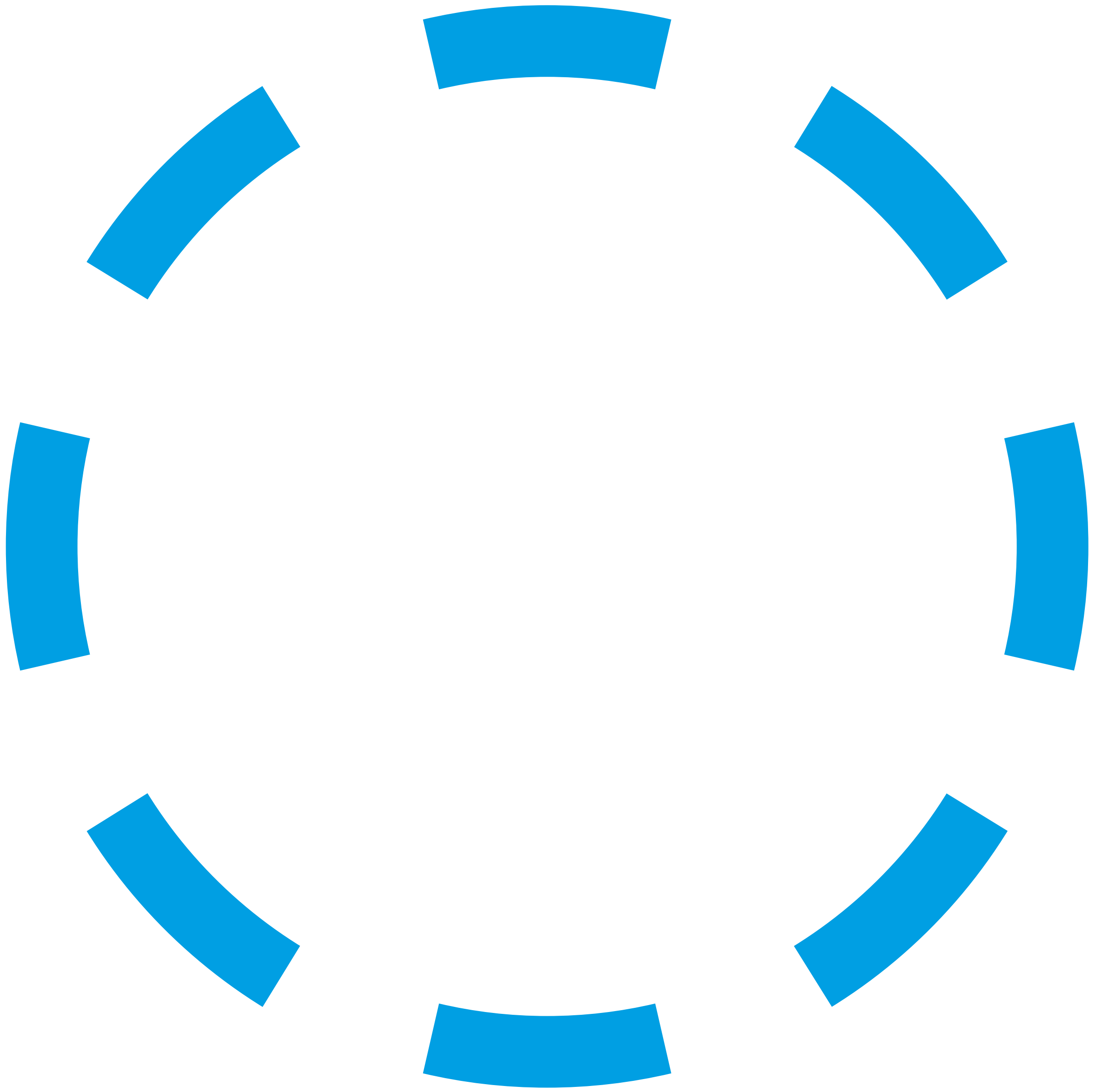

ELSA DEPONT

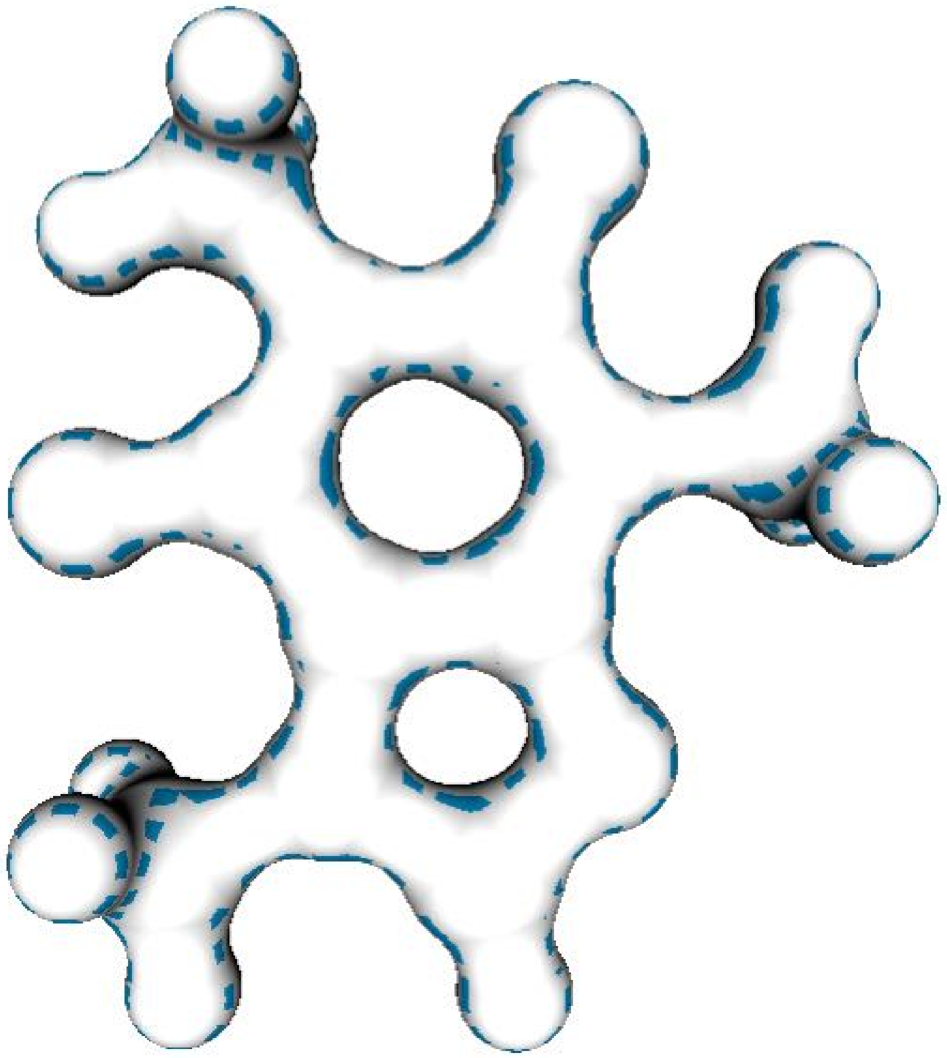

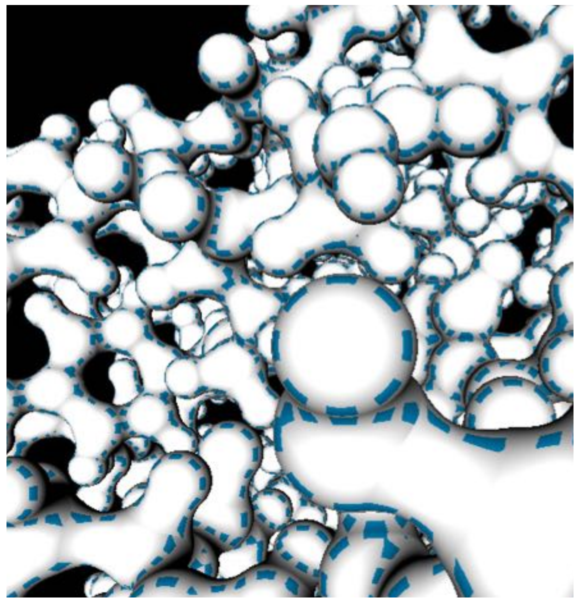

LIT-SPHÈRES
RENDU ARTISTIQUE DE
PROPRIÉTÉS MOLÉCULAIRES

ELSA DEPONT

RIGIDITÉ :

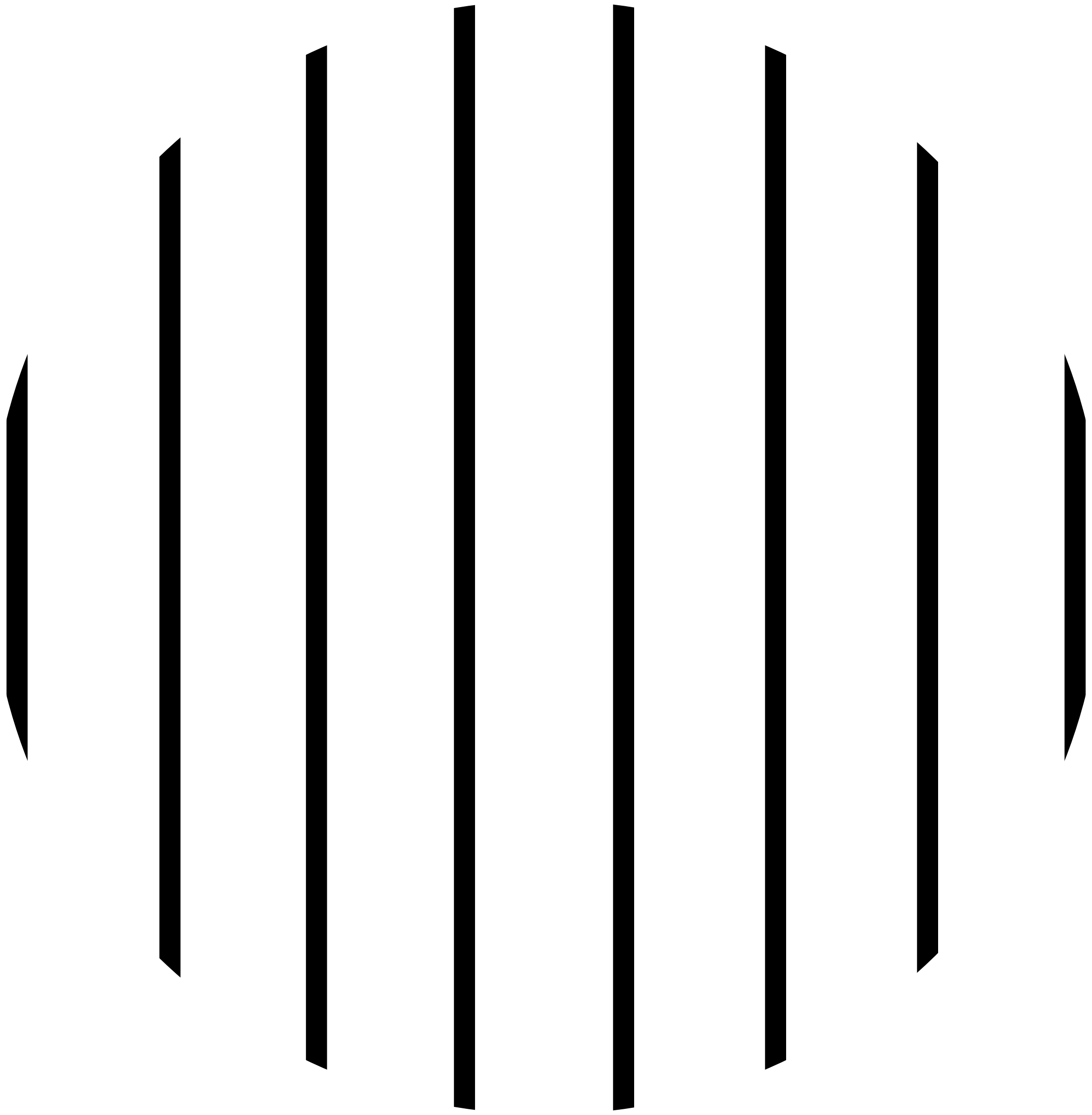

LIT-SPHÈRES
RENDU ARTISTIQUE DE PROPRIÉTÉS MOLÉCULAIRES

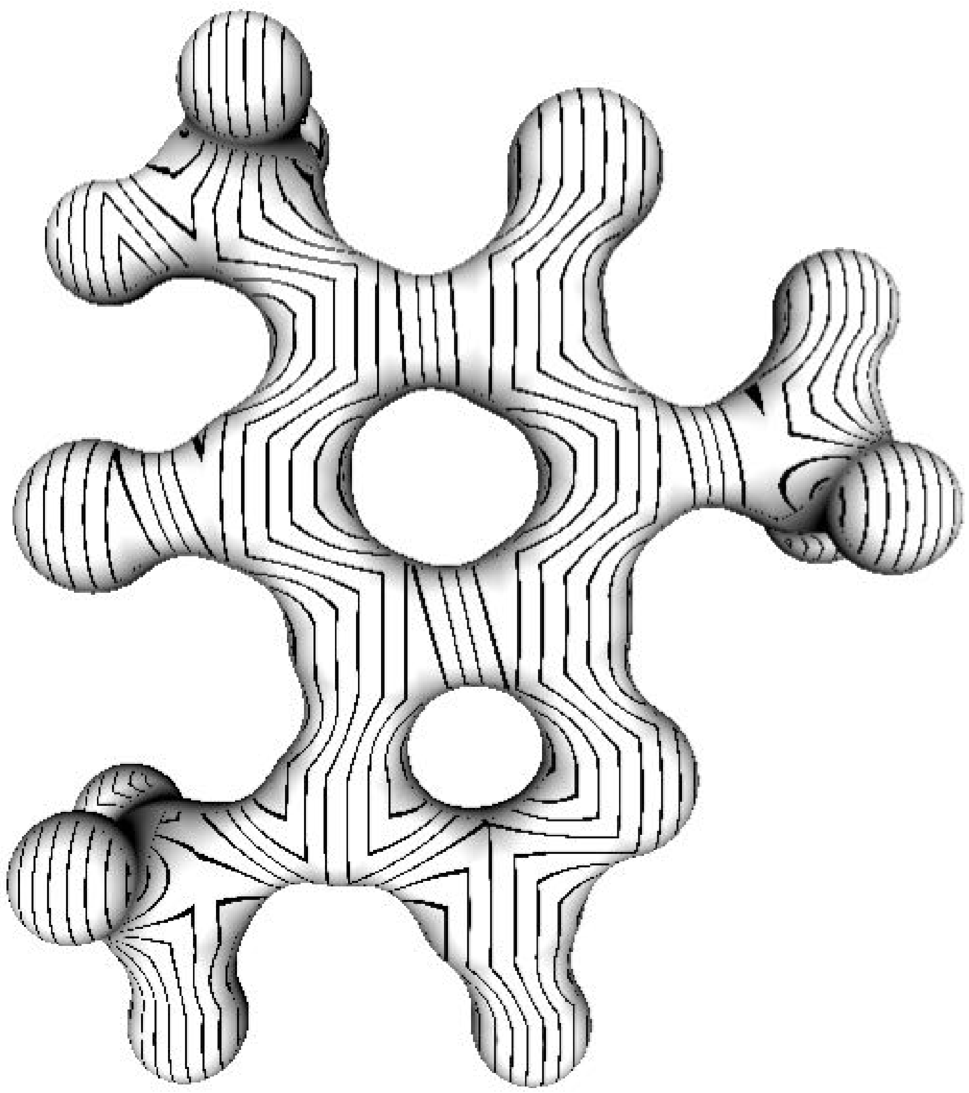

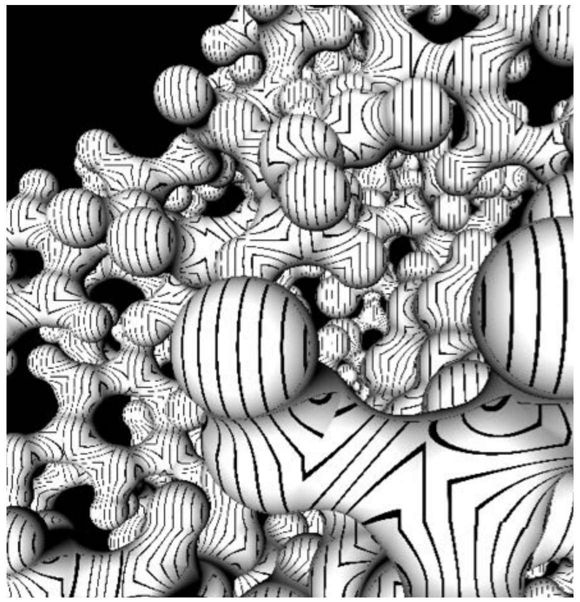

FLEXIBILITÉ :

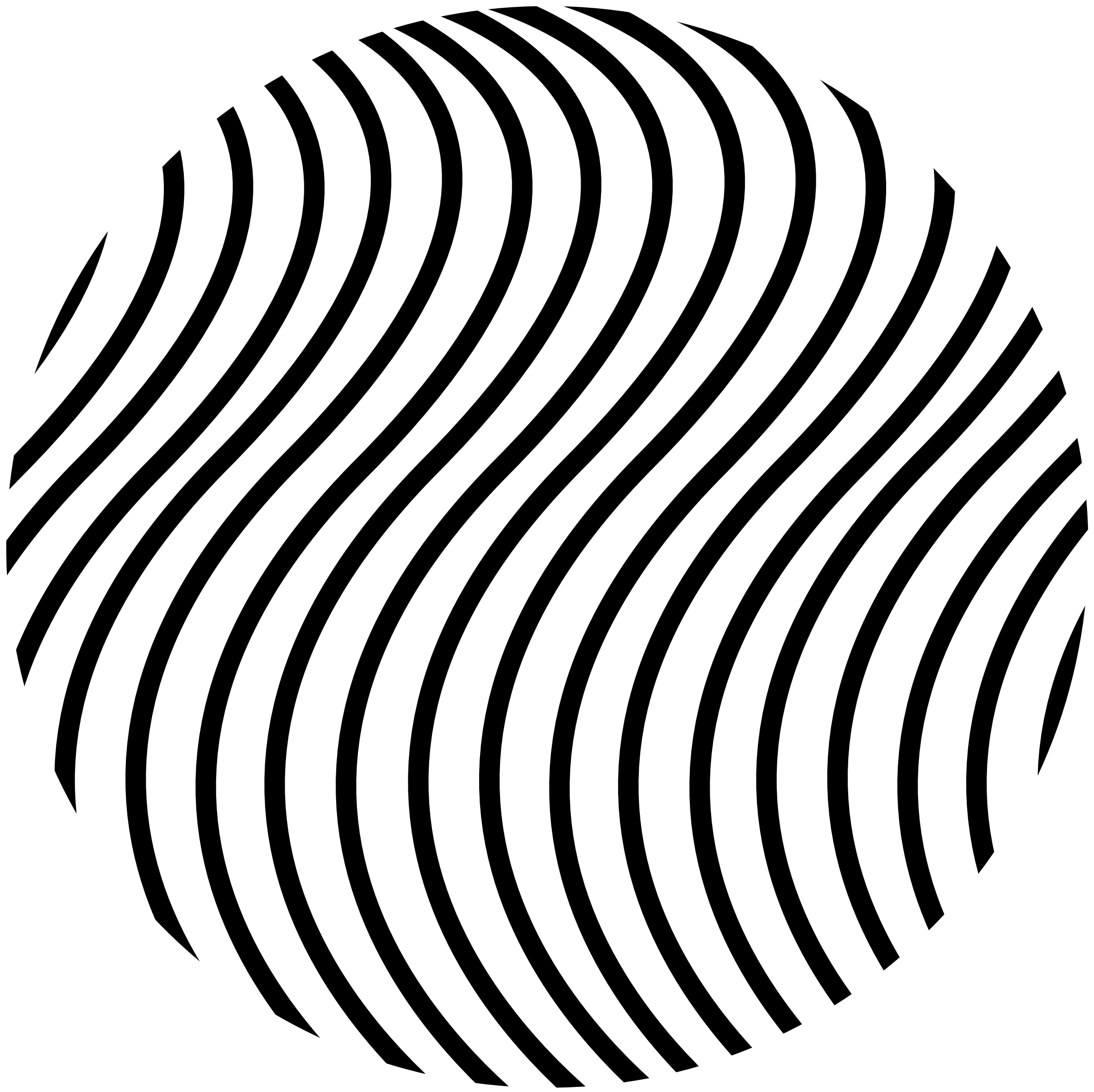

ELSA DEPONT

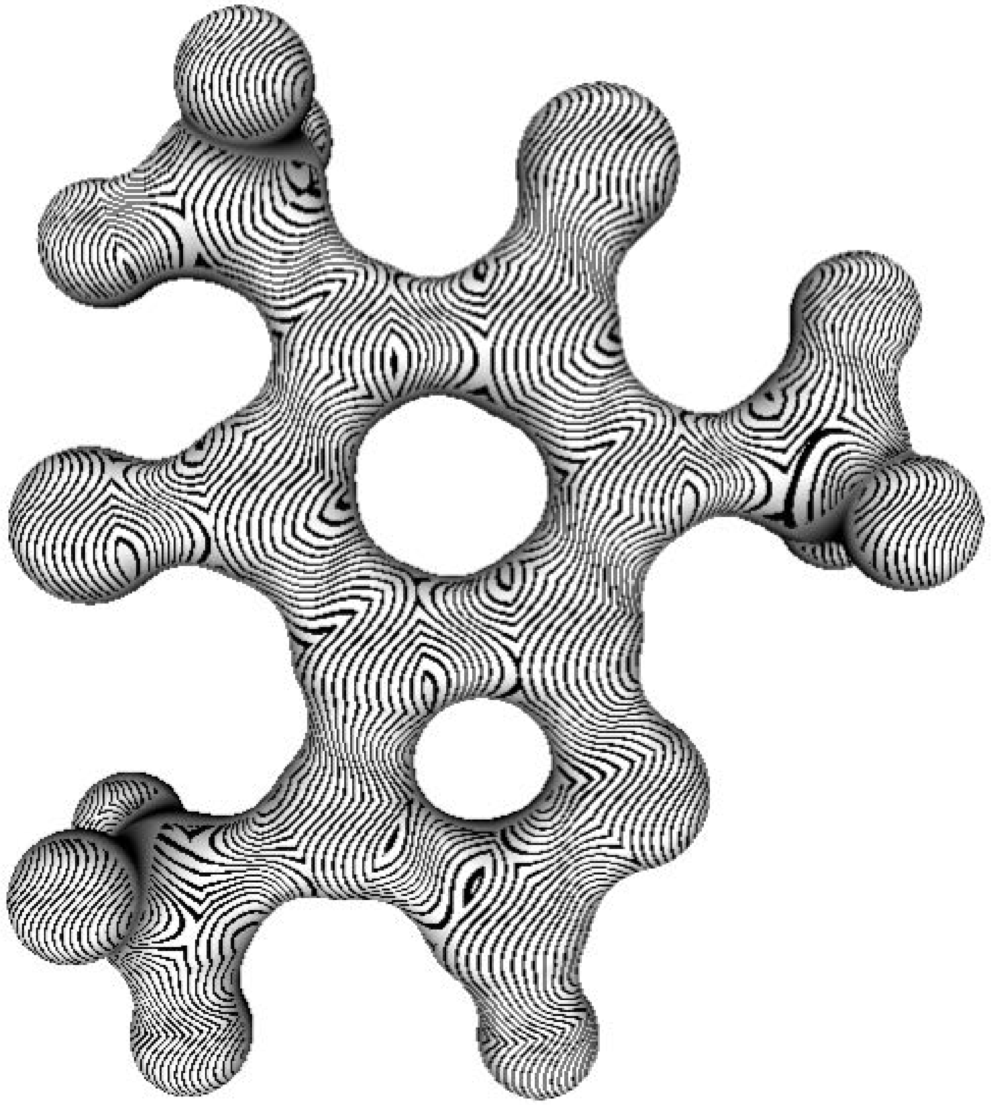

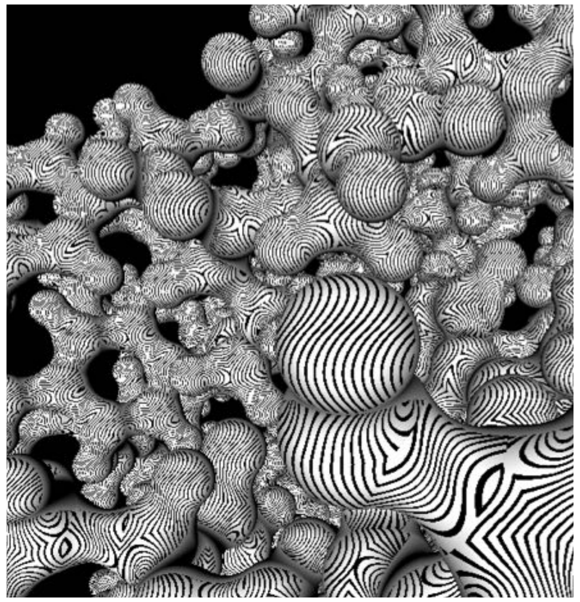

LIT-SPHÈRES
RENDU ARTISTIQUE DE
PROPRIÉTÉS MOLÉCULAIRES

ELSA DEPONT

ACTIVITÉ :

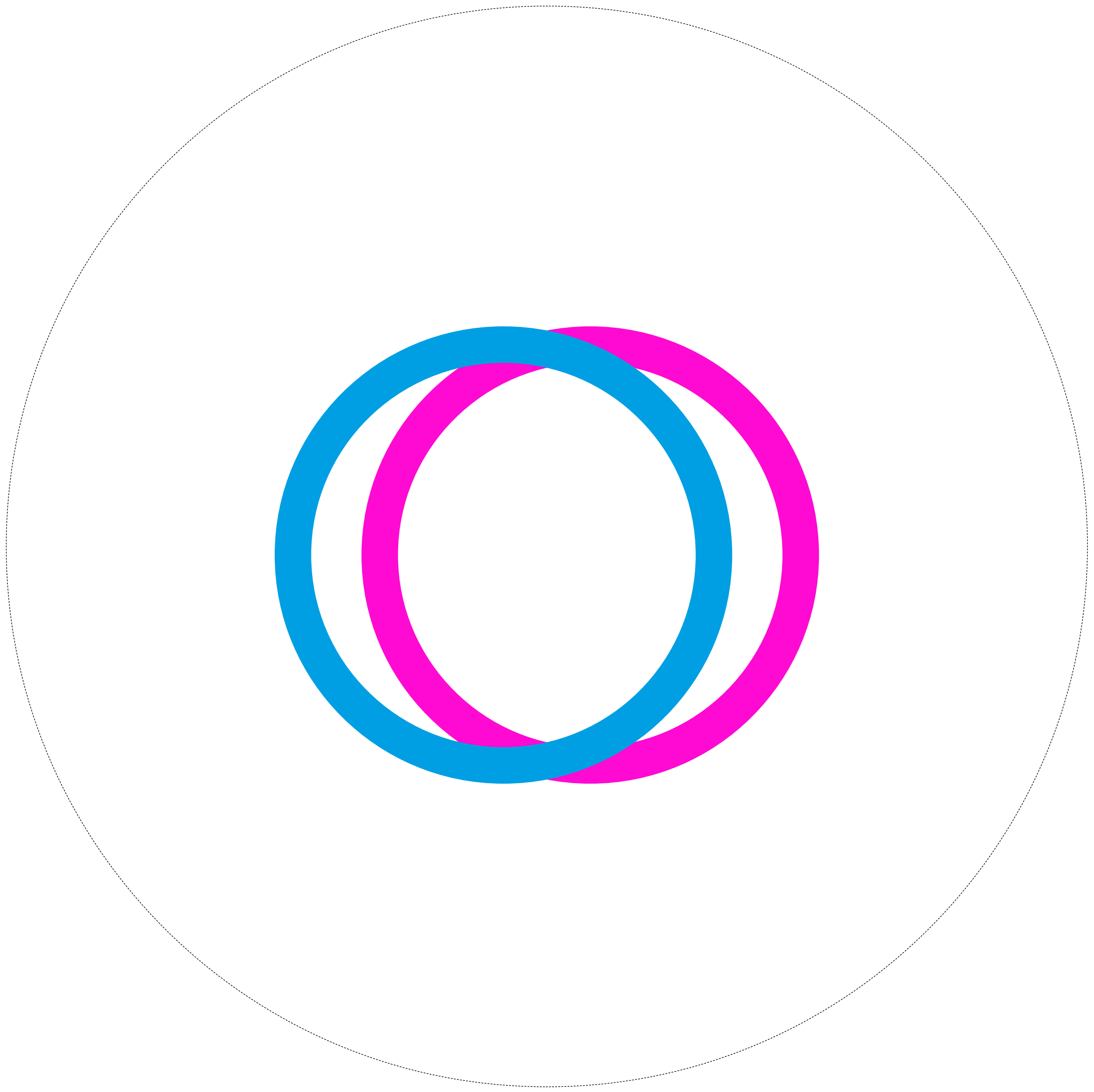

LIT-SPHÈRES
RENDU ARTISTIQUE DE PROPRIÉTÉS MOLÉCULAIRES

ELSA DEPONT

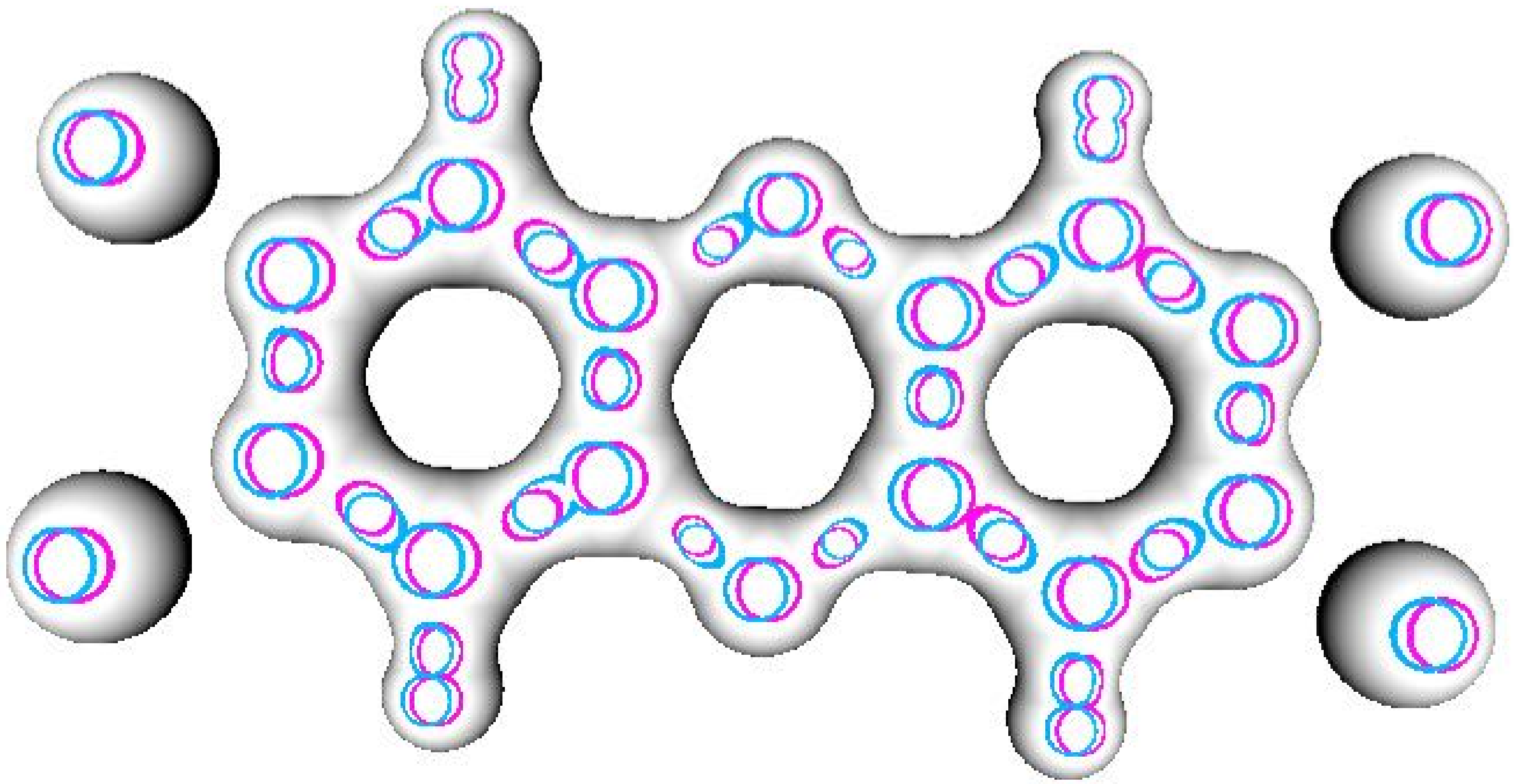

ELSA DEPONT

INACTIVITÉ :

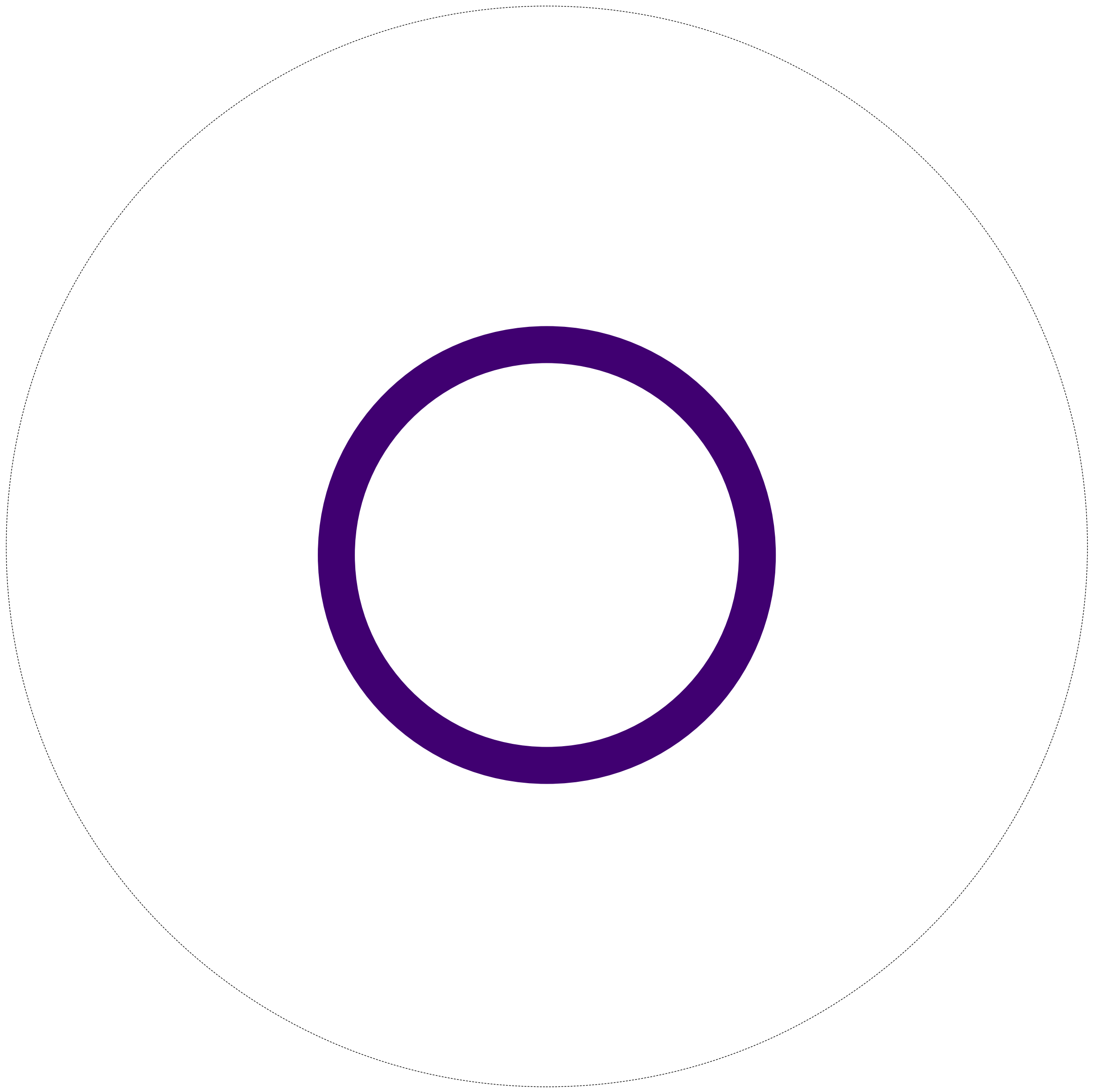

LIT-SPHÈRES
RENDU ARTISTIQUE DE
PROPRIÉTÉS MOLÉCULAIRES

ELSA DEPONT

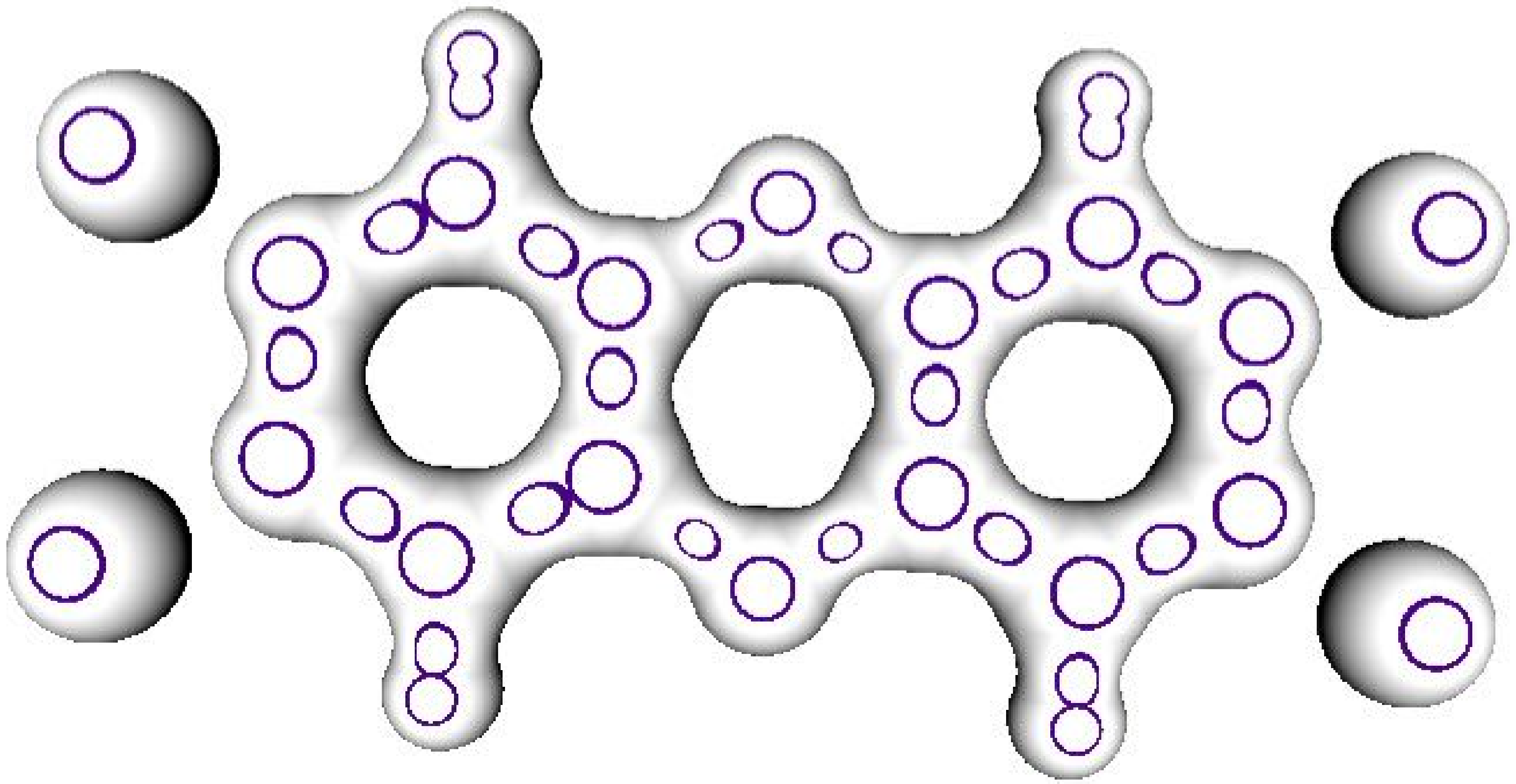

LIT-SPHÈRES
RENDU ARTISTIQUE DE
PROPRIÉTÉS MOLÉCULAIRES

ELSA DEPONT

ÉCOCOMPATIBILITÉ :

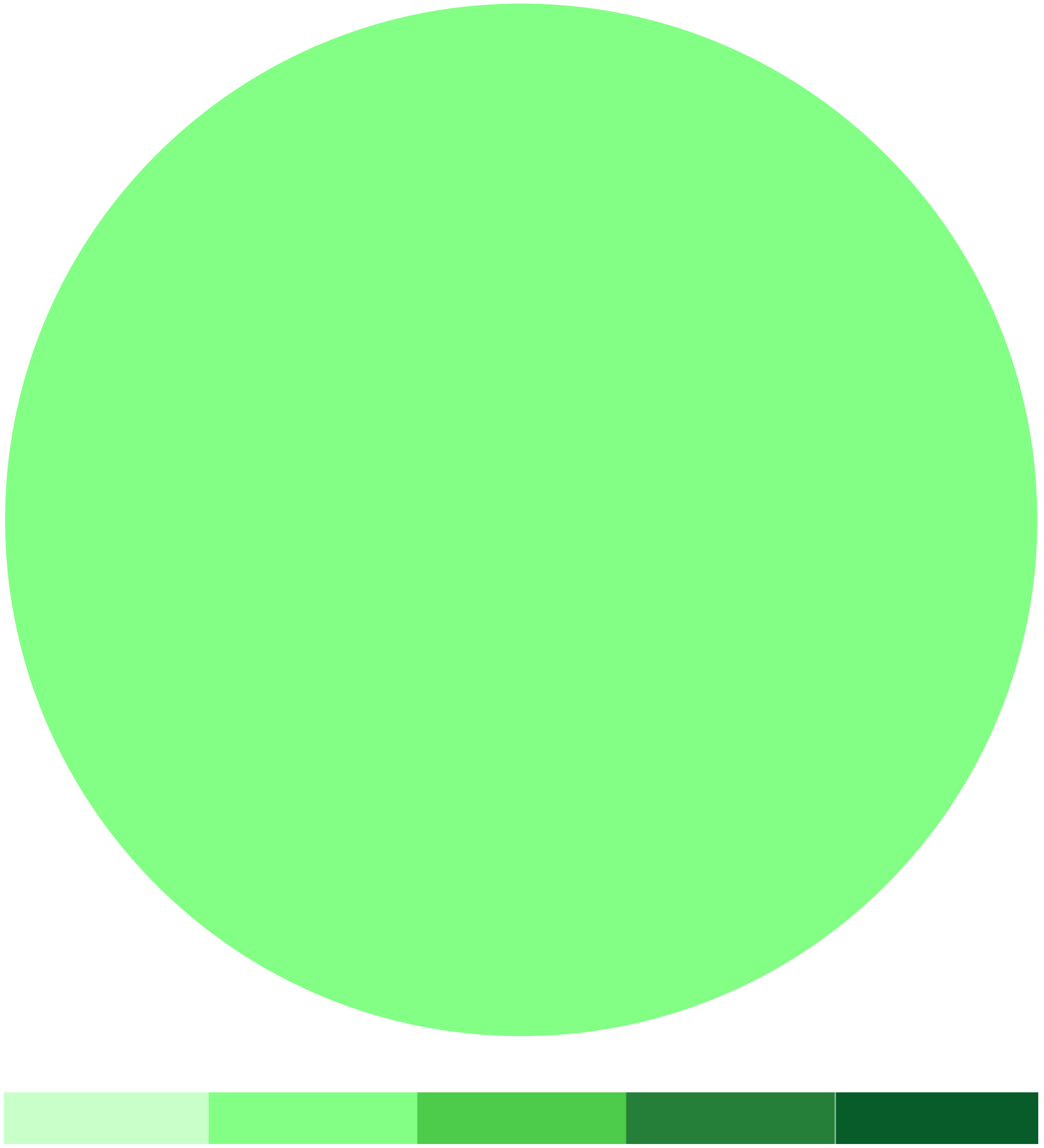

LIT-SPHÈRES
RENDU ARTISTIQUE DE
PROPRIÉTÉS MOLÉCULAIRES

ELSA DEPONT

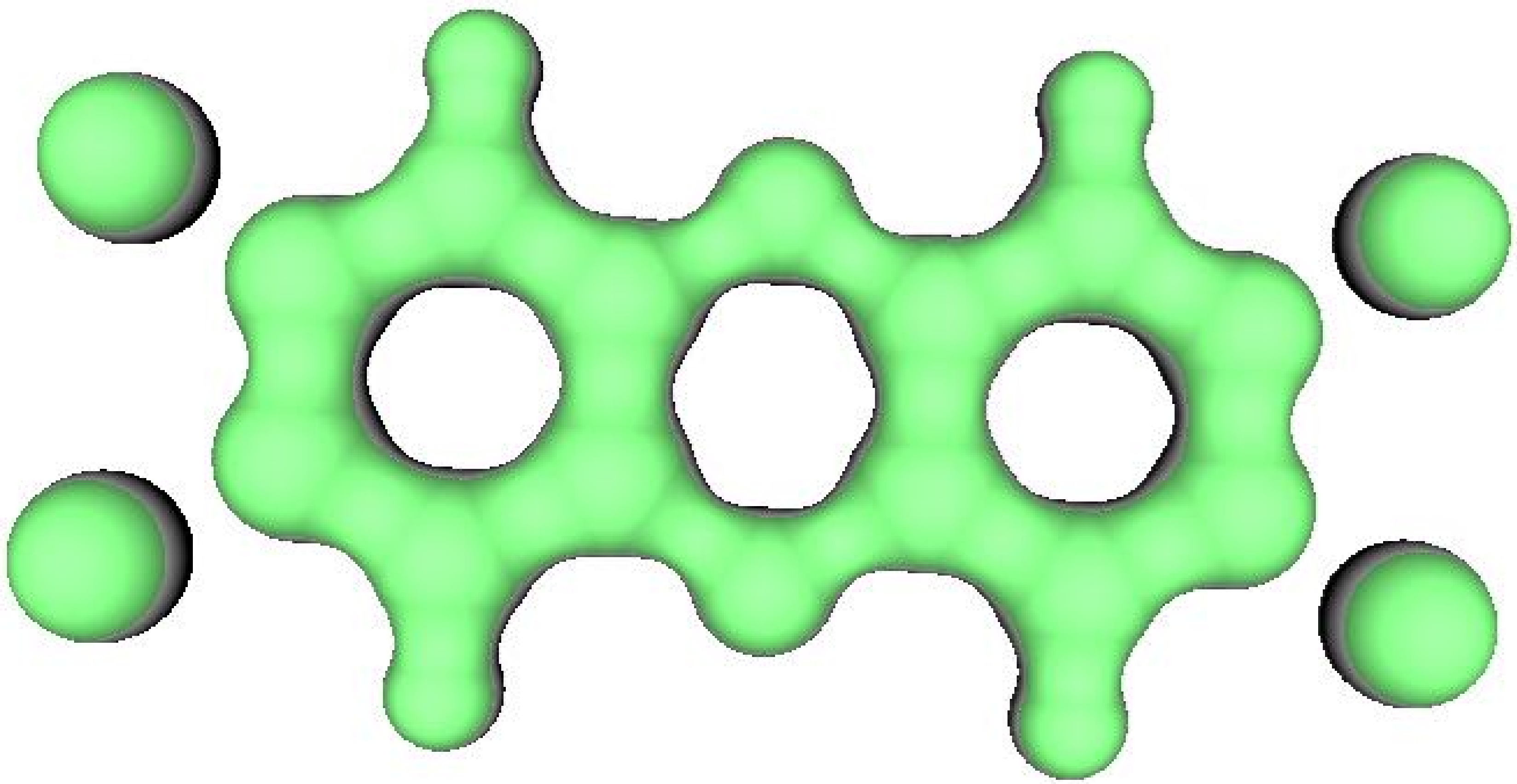

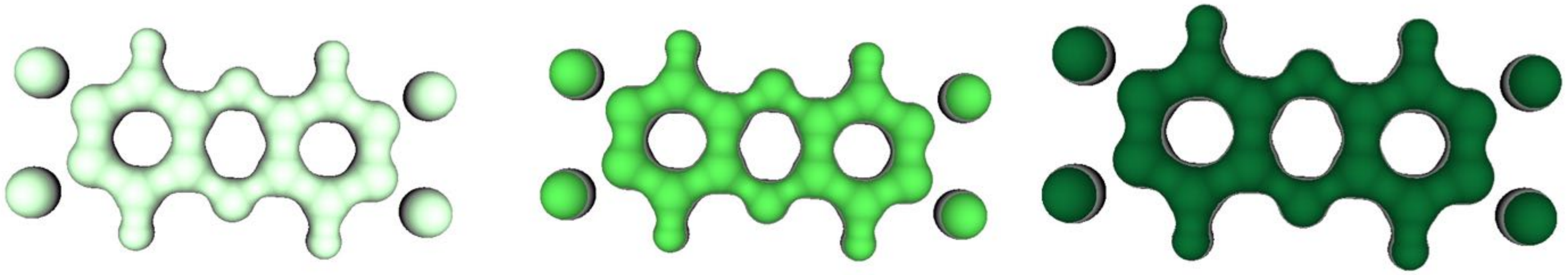

LIT-SPHÈRES
RENDU ARTISTIQUE DE
PROPRIÉTÉS MOLÉCULAIRES

ELSA DEPONT

TOXICITÉ :

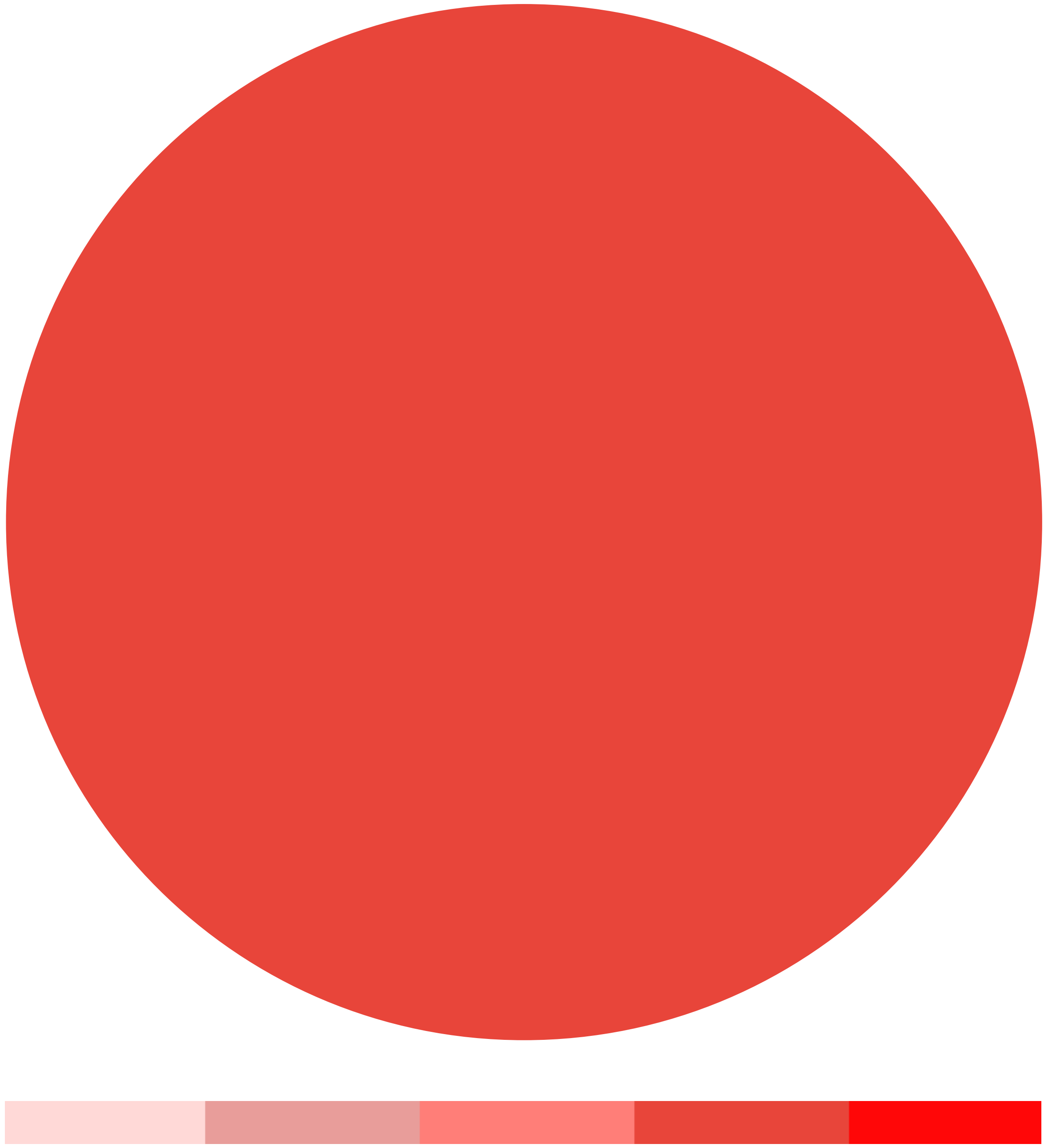

LIT-SPHÈRES
RENDU ARTISTIQUE DE
PROPRIÉTÉS MOLÉCULAIRES

ELSA DEPONT

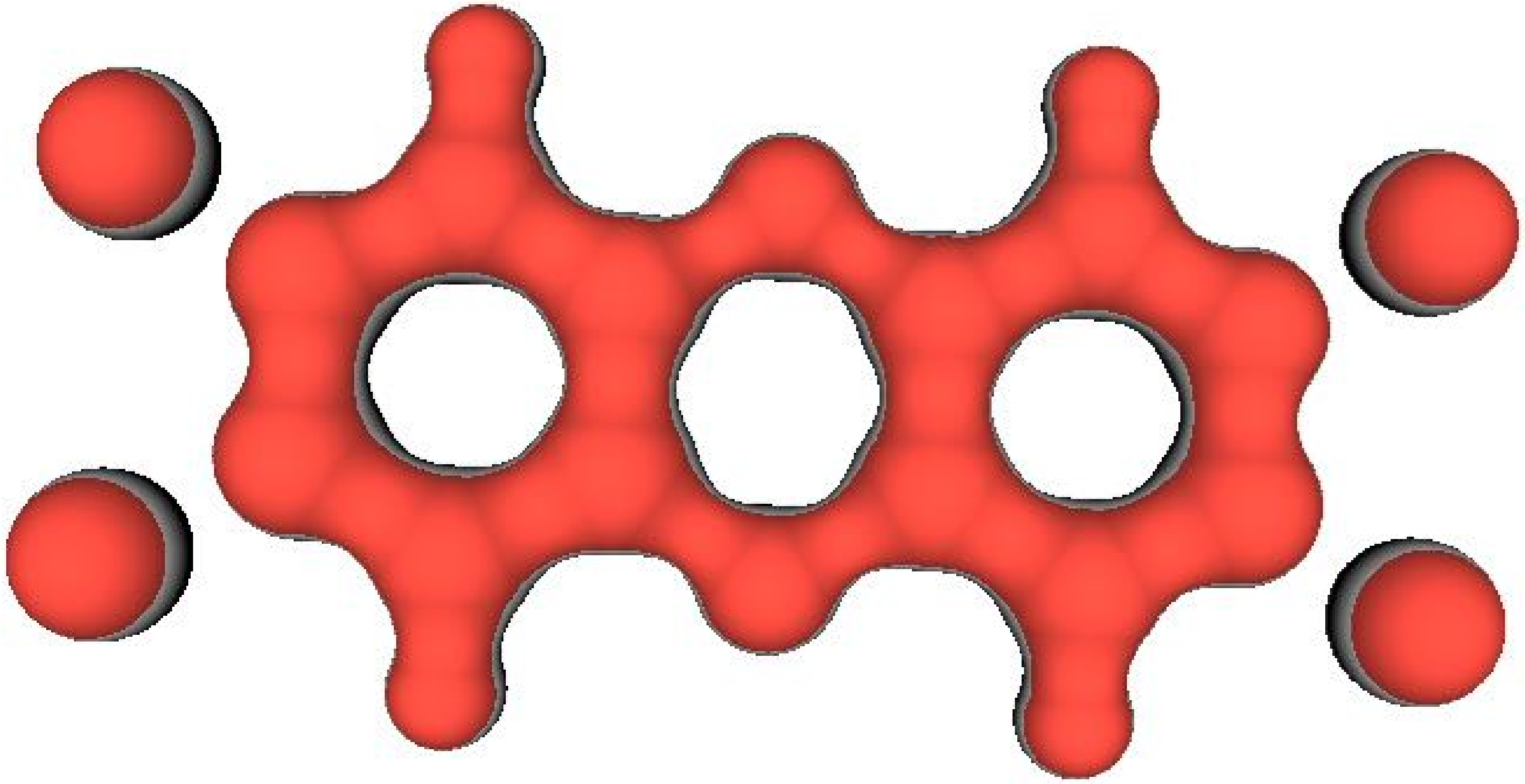

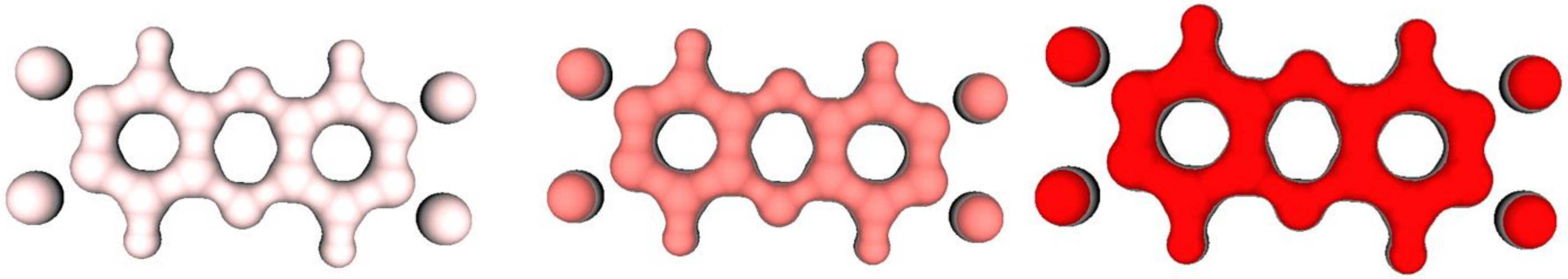

LIT-SPHÈRES
RENDU ARTISTIQUE DE
PROPRIÉTÉS MOLÉCULAIRES

CHARGE NÉGATIVE:

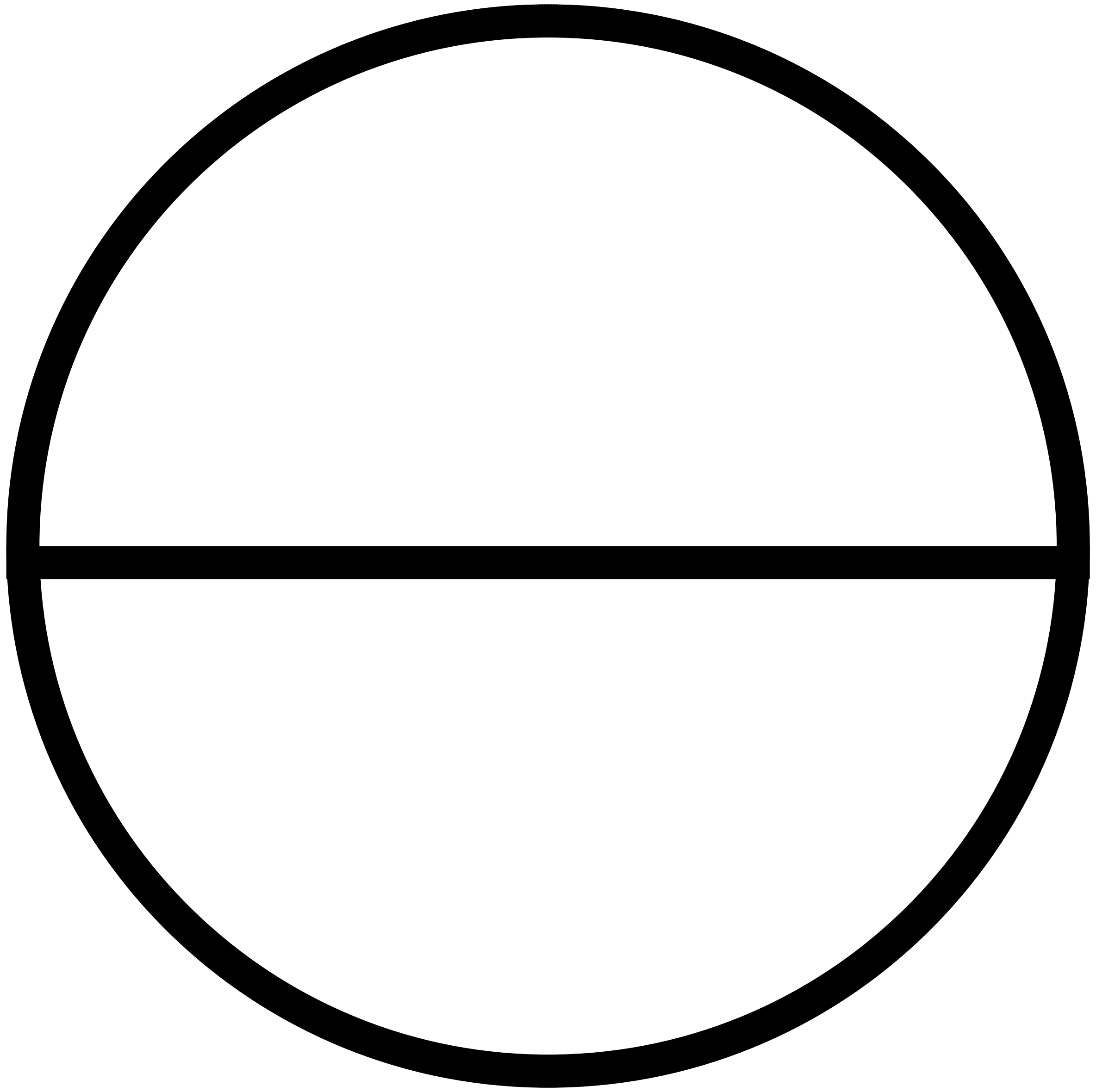

ELSA DEPONT

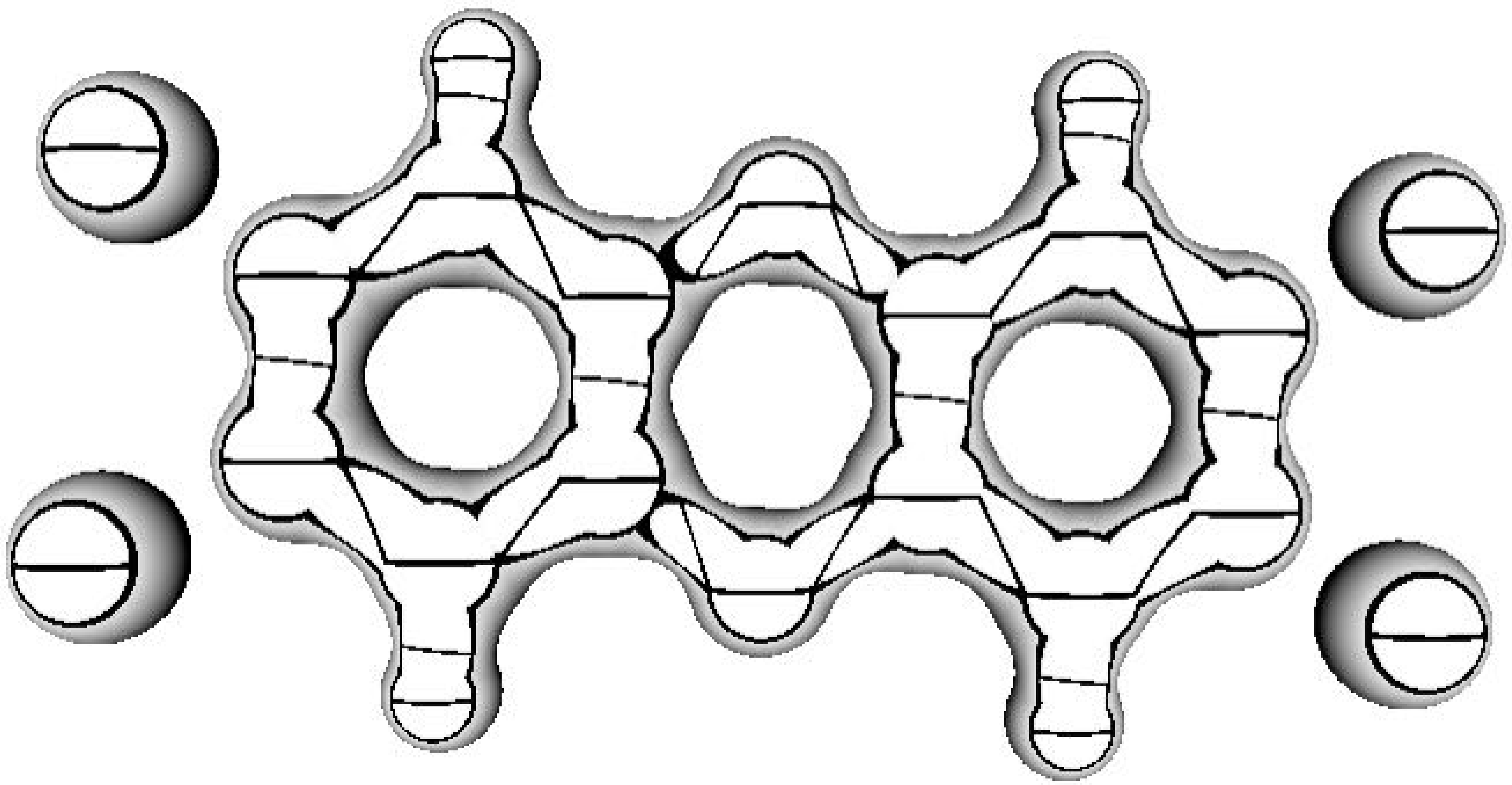

LIT-SPHÈRES

RENDU ARTISTIQUE DE PROPRIÉTÉS MOLÉCULAIRES

ELSA DEPONT

CHARGE POSITIVE:

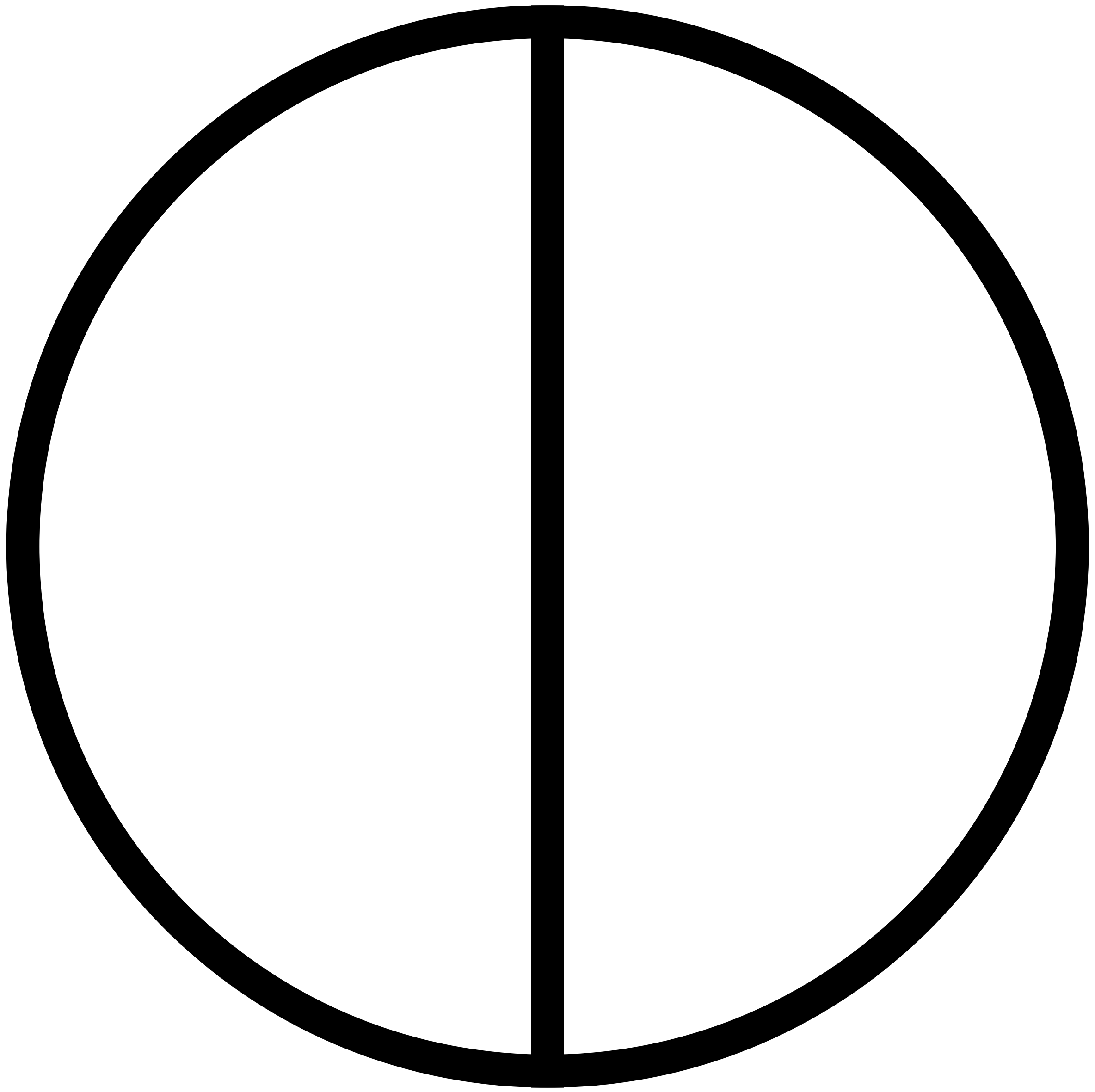

ELSA DEPONT

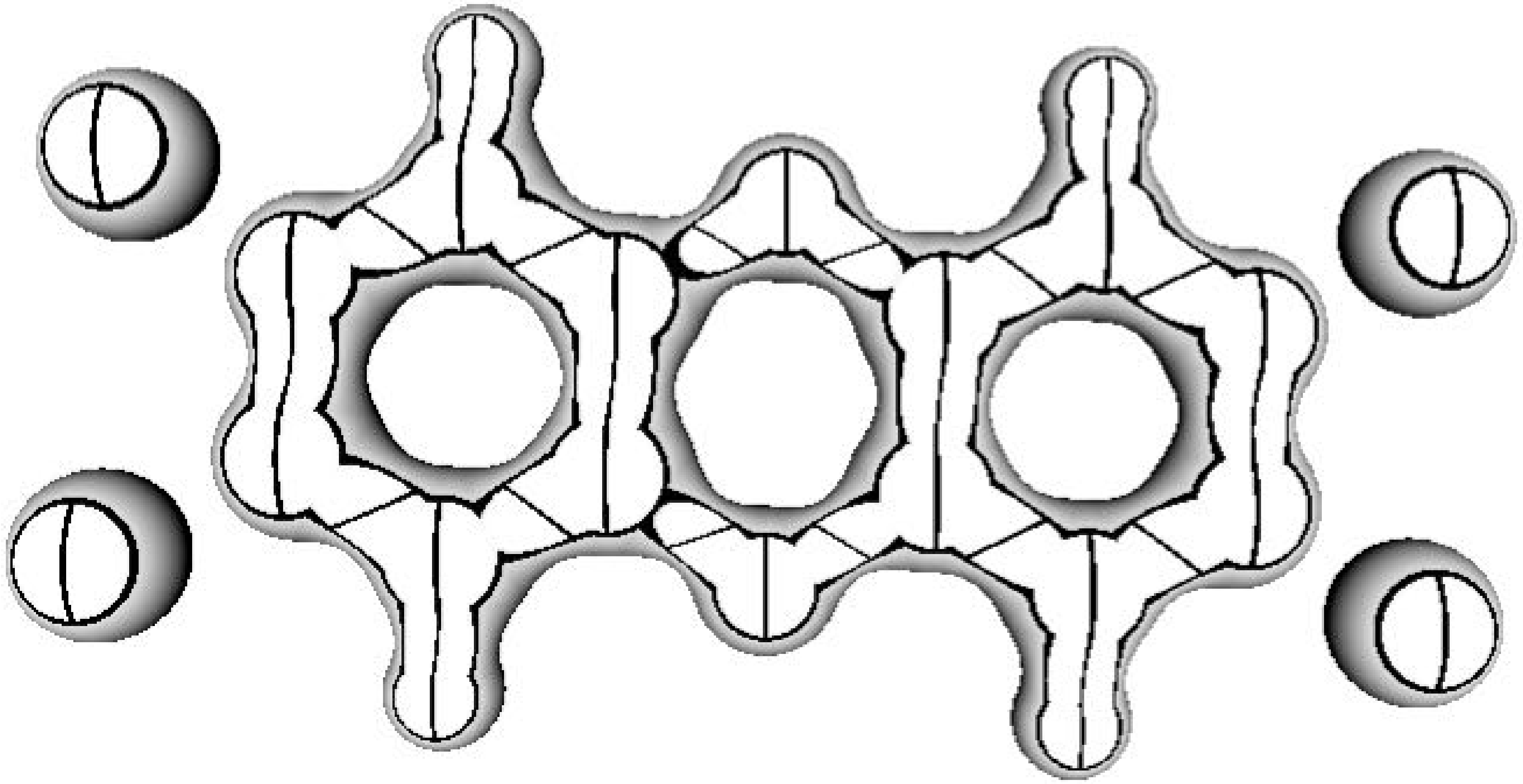

LIT-SPHÈRES
RENDU ARTISTIQUE DE
PROPRIÉTÉS MOLÉCULAIRES

ELSA DEPONT

BON:

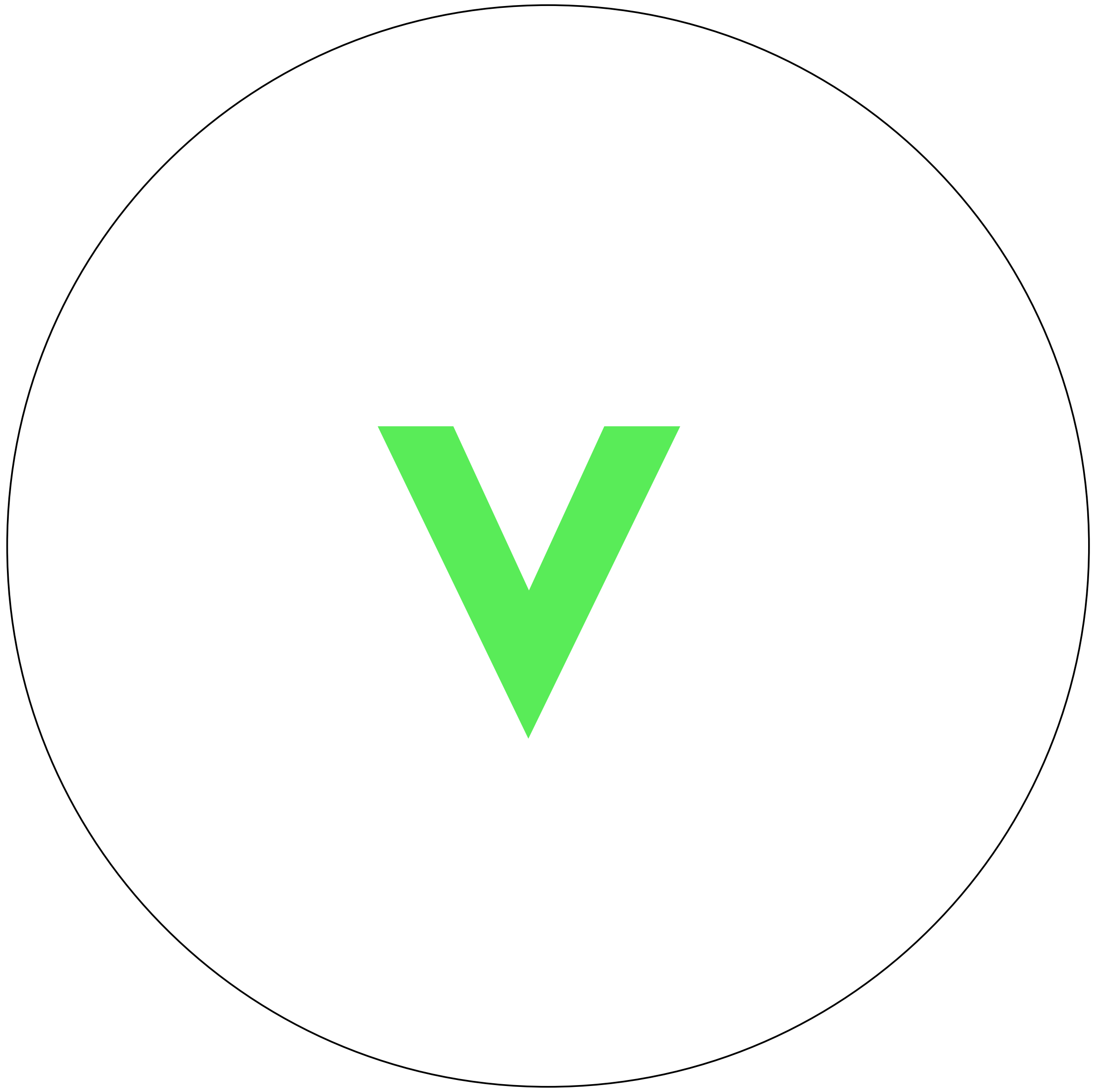

LIT-SPHÈRES
RENDU ARTISTIQUE DE
PROPRIÉTÉS MOLÉCULAIRES

ELSA DEPONT

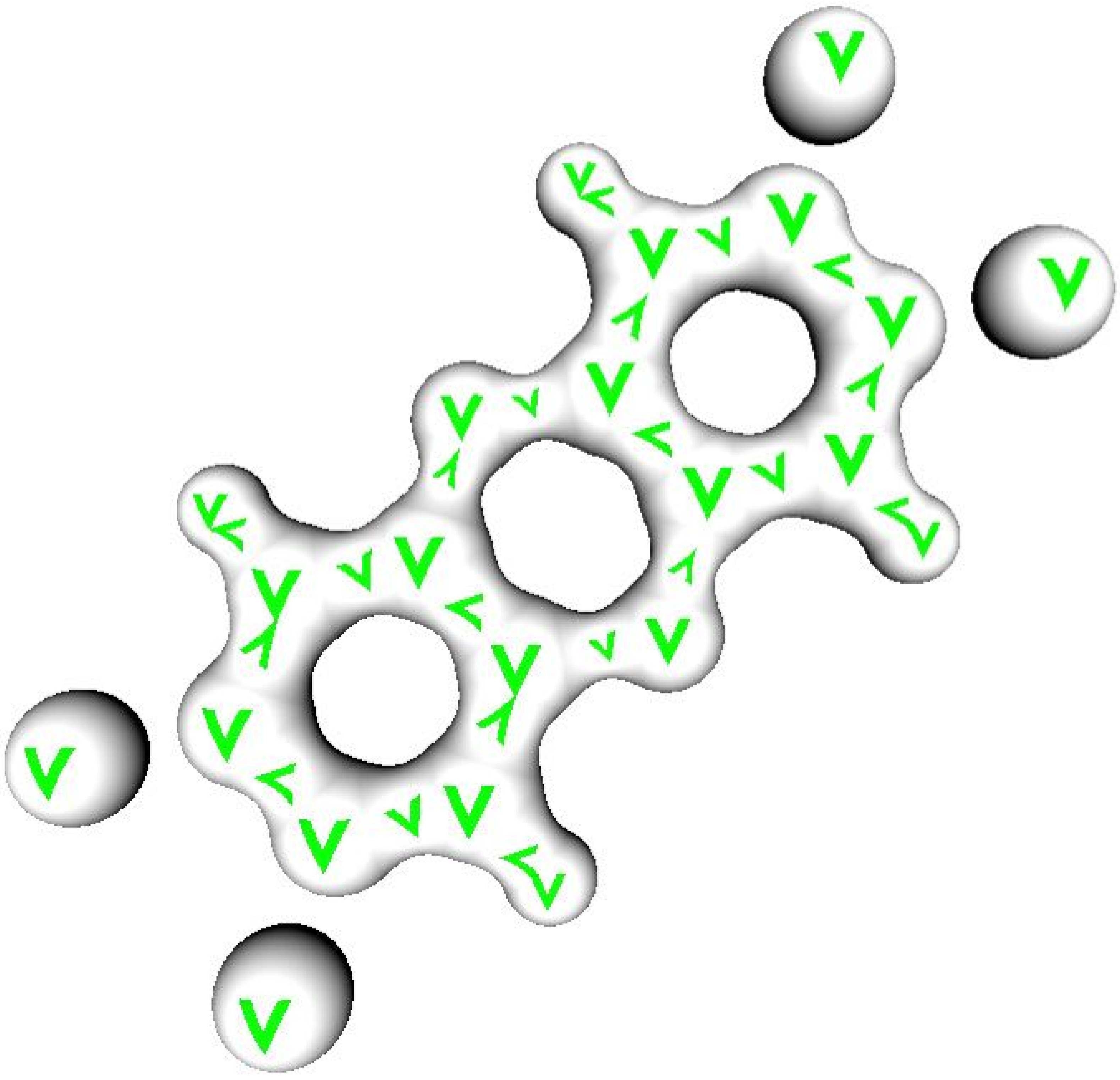

LIT-SPHÈRES
RENDU ARTISTIQUE DE
PROPRIÉTÉS MOLÉCULAIRES

MAUVAIS:

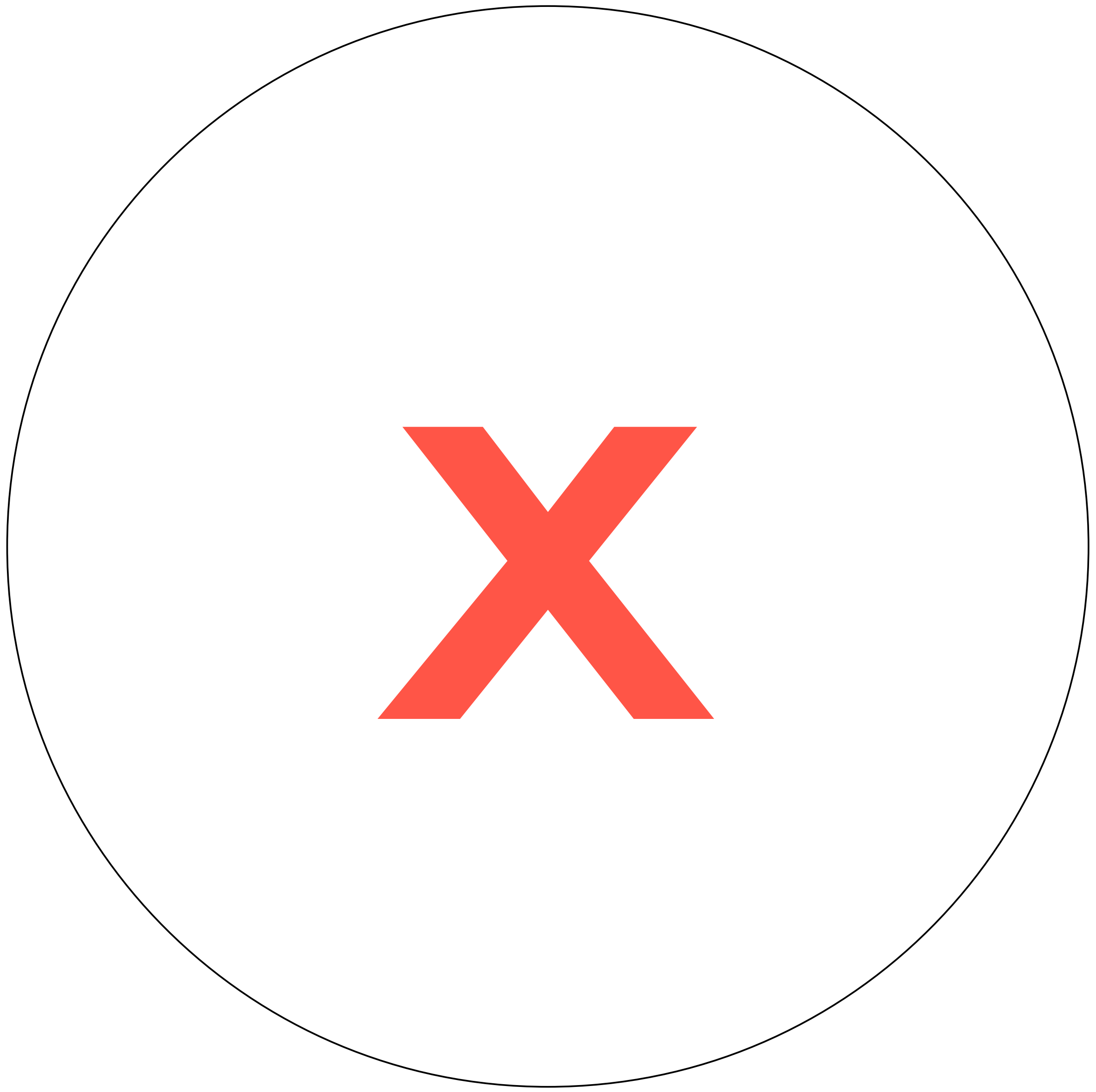

ELSA DEPONT

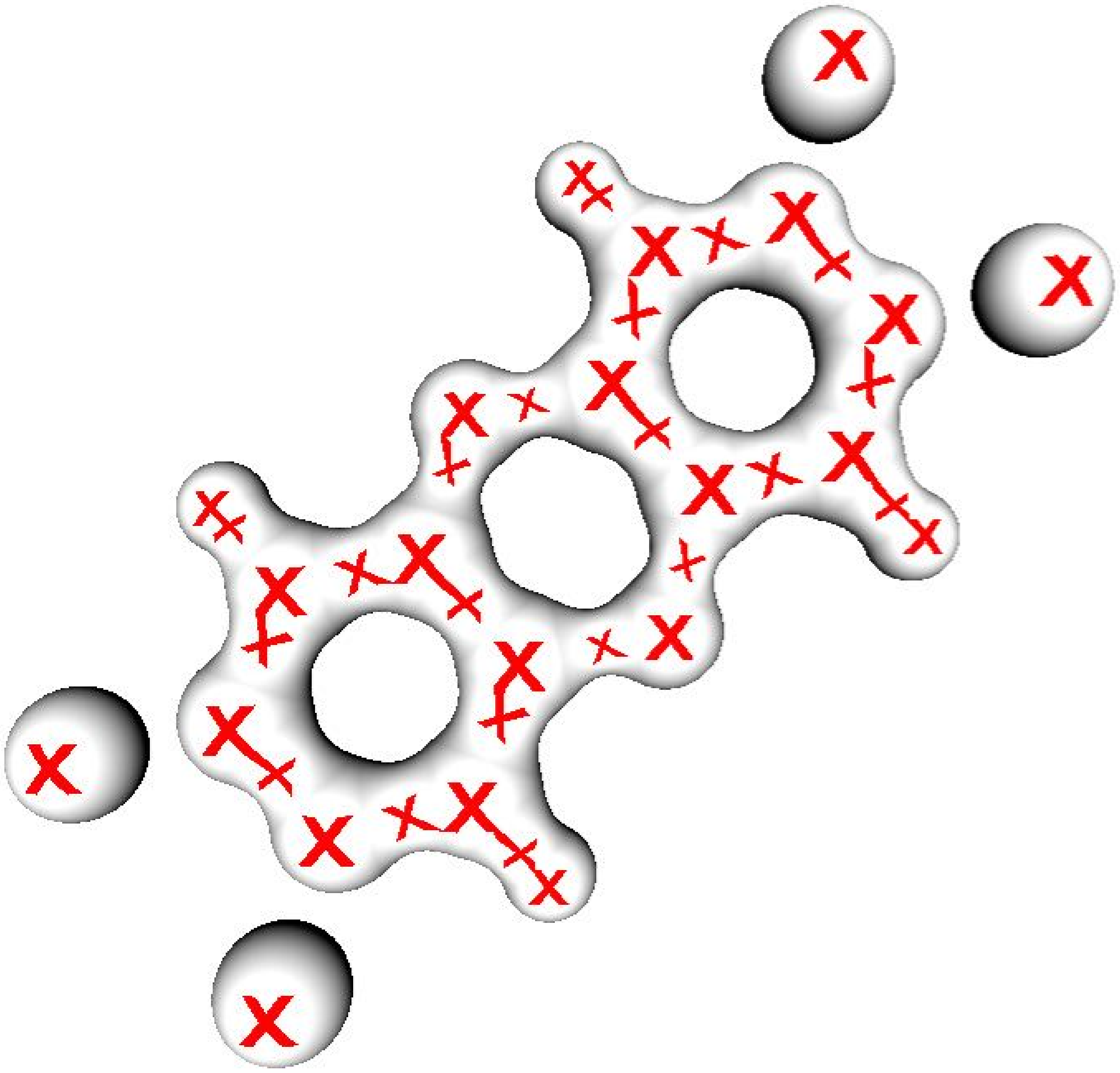

LIT-SPHÈRES
RENDU ARTISTIQUE DE PROPRIÉTÉS MOLÉCULAIRES

ELSA DEPONT

NATURE DE L'ATOME:
CARBONE

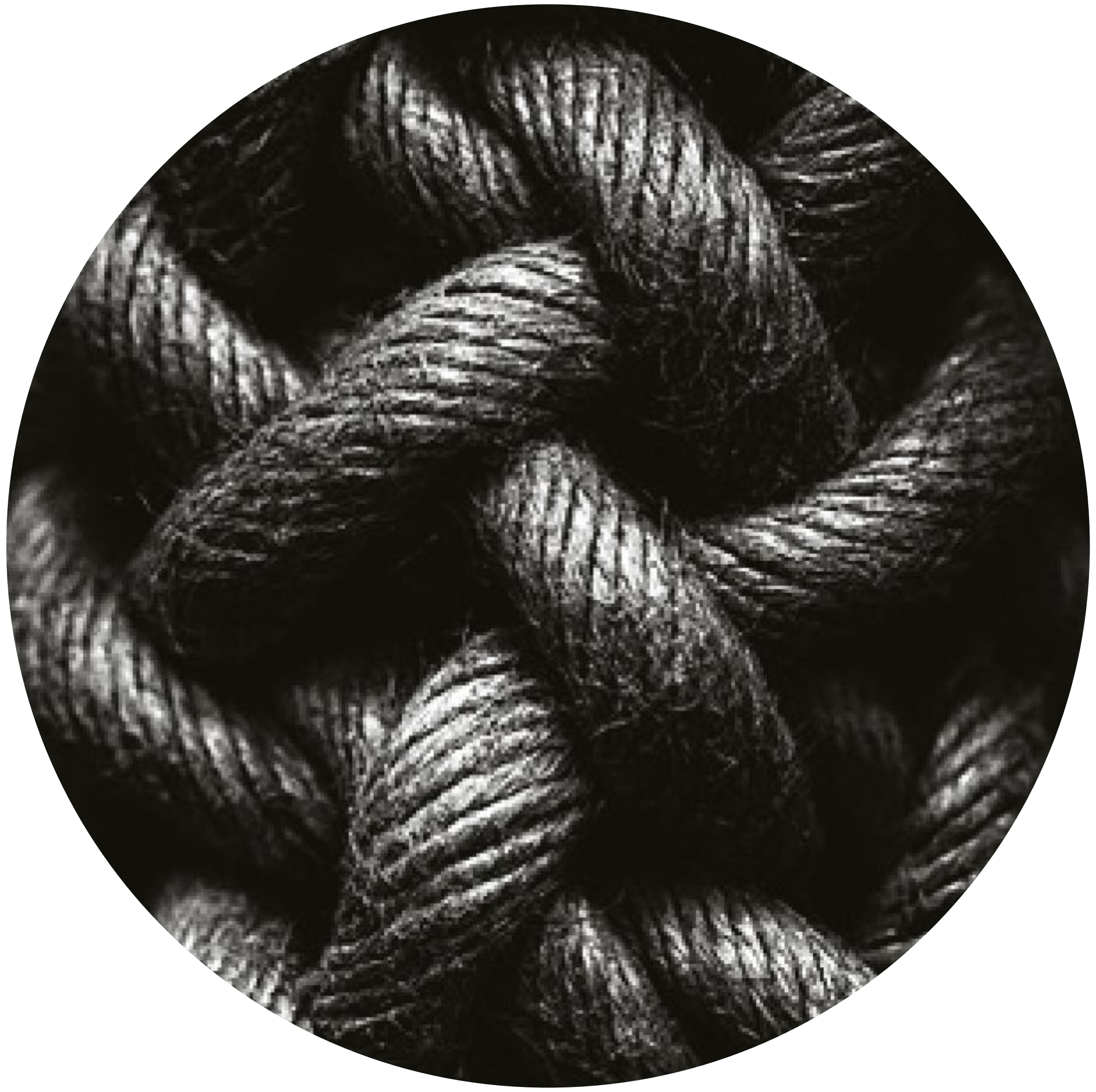

LIT-SPHÈRES
RENDU ARTISTIQUE DE
PROPRIÉTÉS MOLÉCULAIRES

ELSA DEPONT

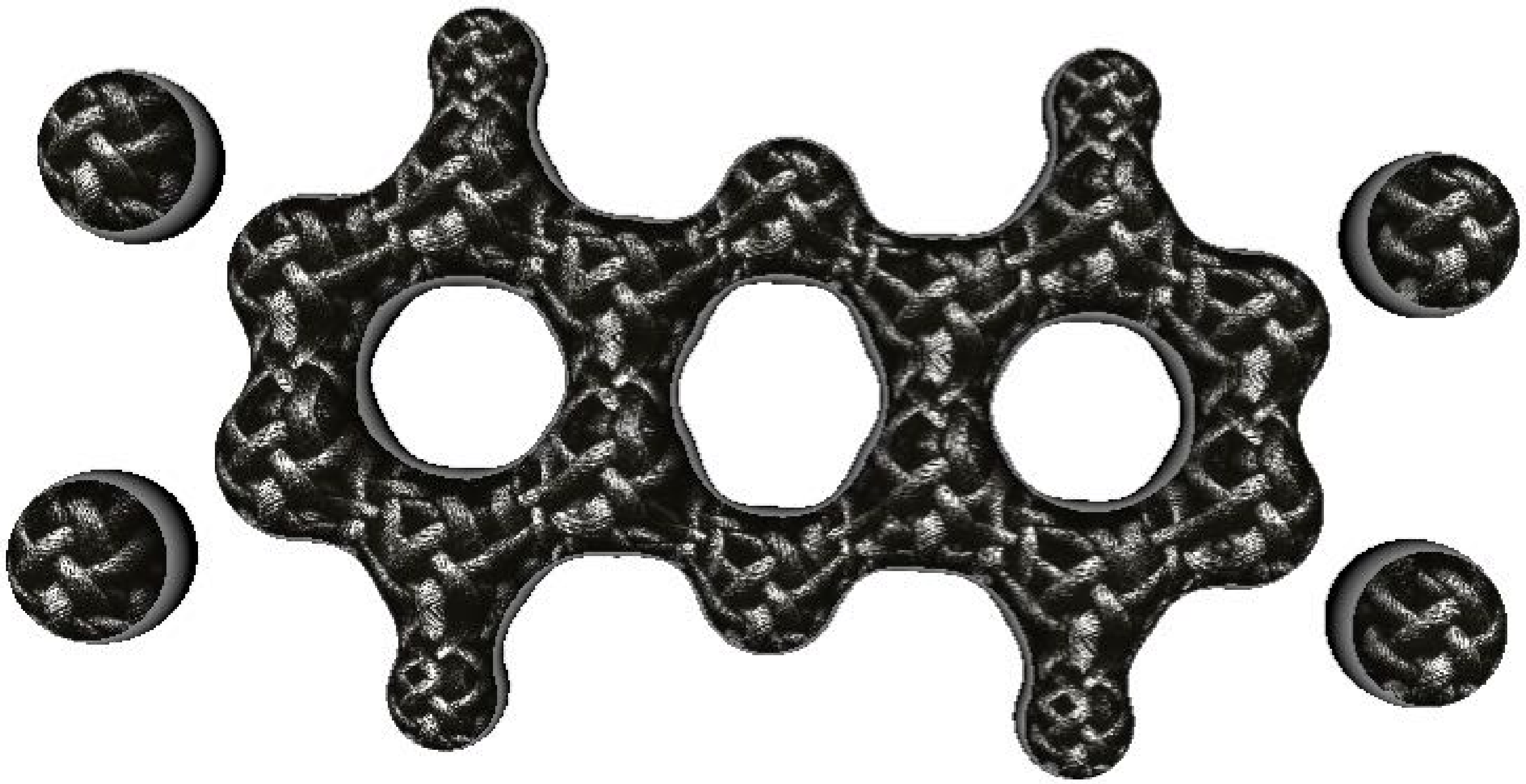

LIT-SPHÈRES
RENDU ARTISTIQUE DE
PROPRIÉTÉS MOLÉCULAIRES

ELSA DEPONT

NATURE DE L'ATOME:
OXYGÈNE

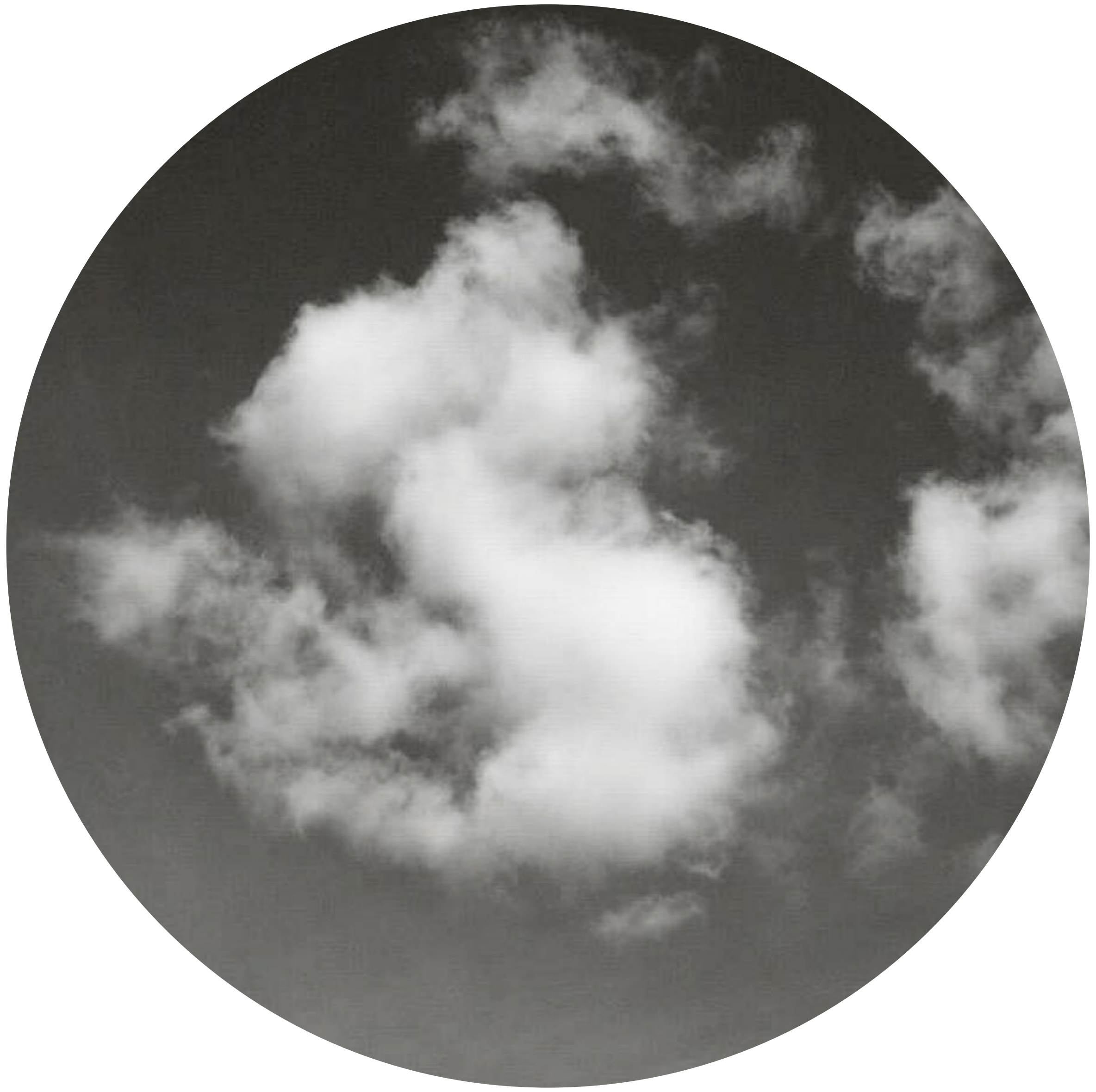

LIT-SPHÈRES
RENDU ARTISTIQUE DE
PROPRIÉTÉS MOLÉCULAIRES

ELSA DEPONT

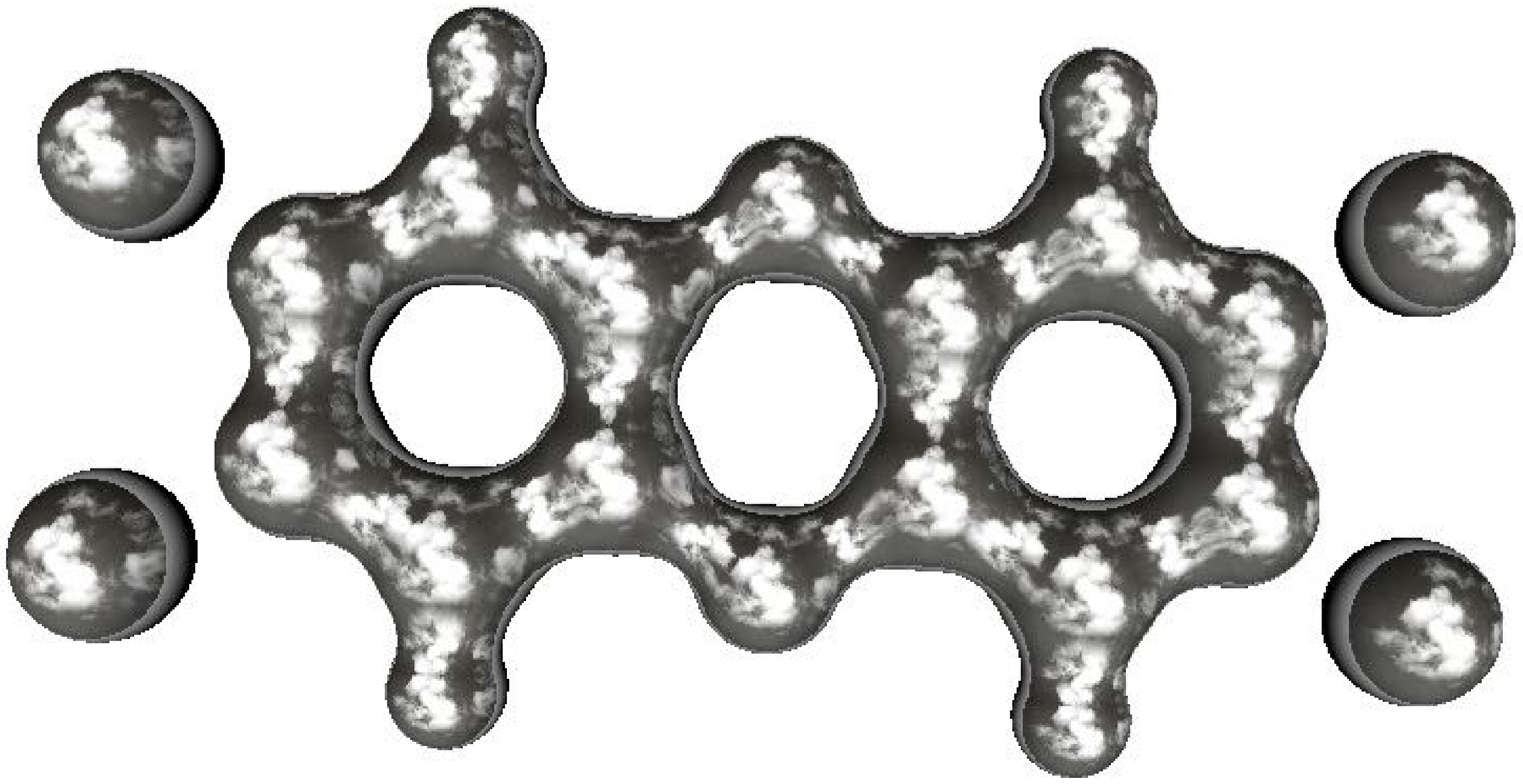

LIT-SPHÈRES
RENDU ARTISTIQUE DE
PROPRIÉTÉS MOLÉCULAIRES

ELSA DEPONT

NATURE DE LA MOLÉCULE:
GRAISSE

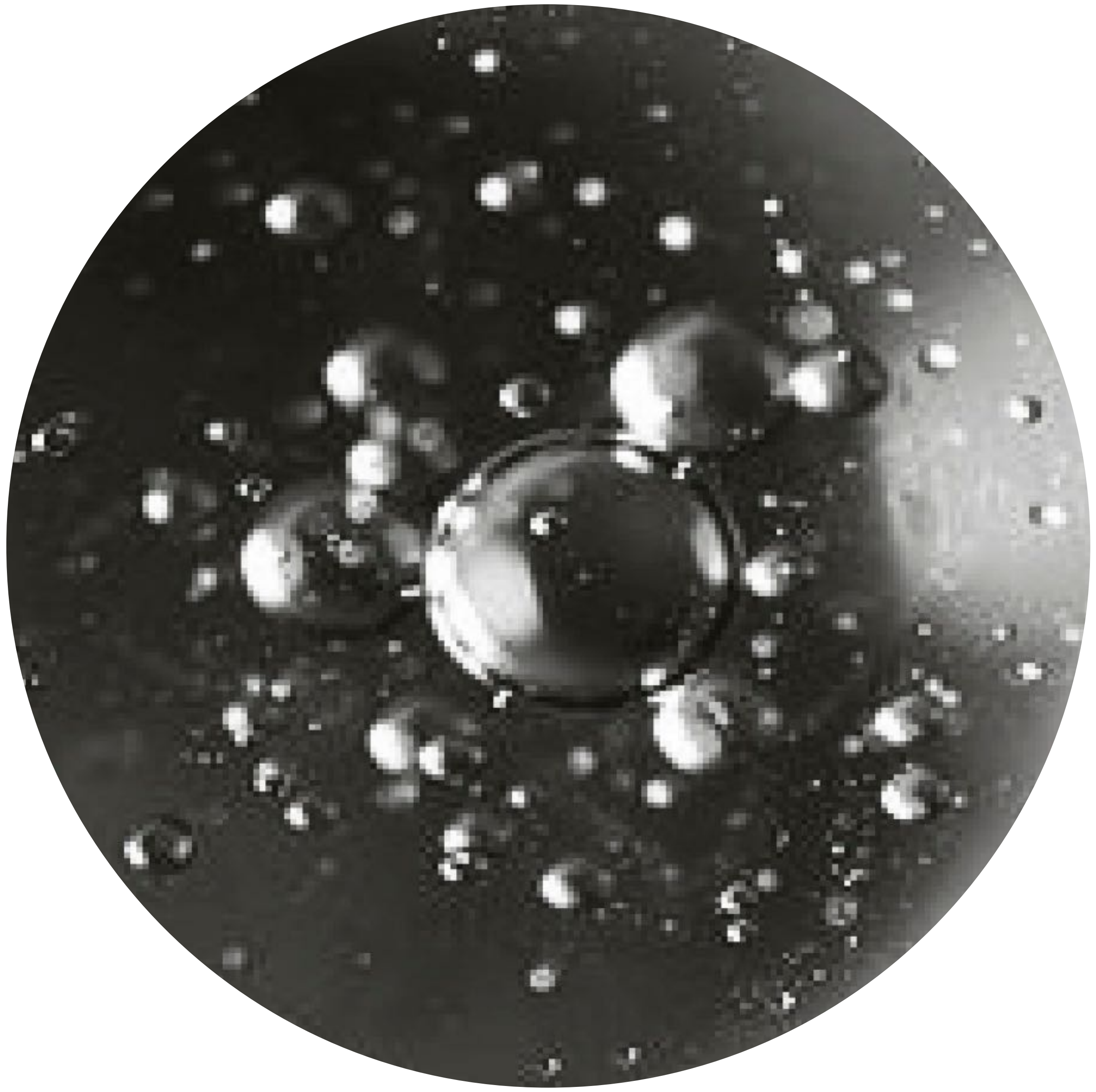

LIT-SPHÈRES
RENDU ARTISTIQUE DE
PROPRIÉTÉS MOLÉCULAIRES

ELSA DEPONT

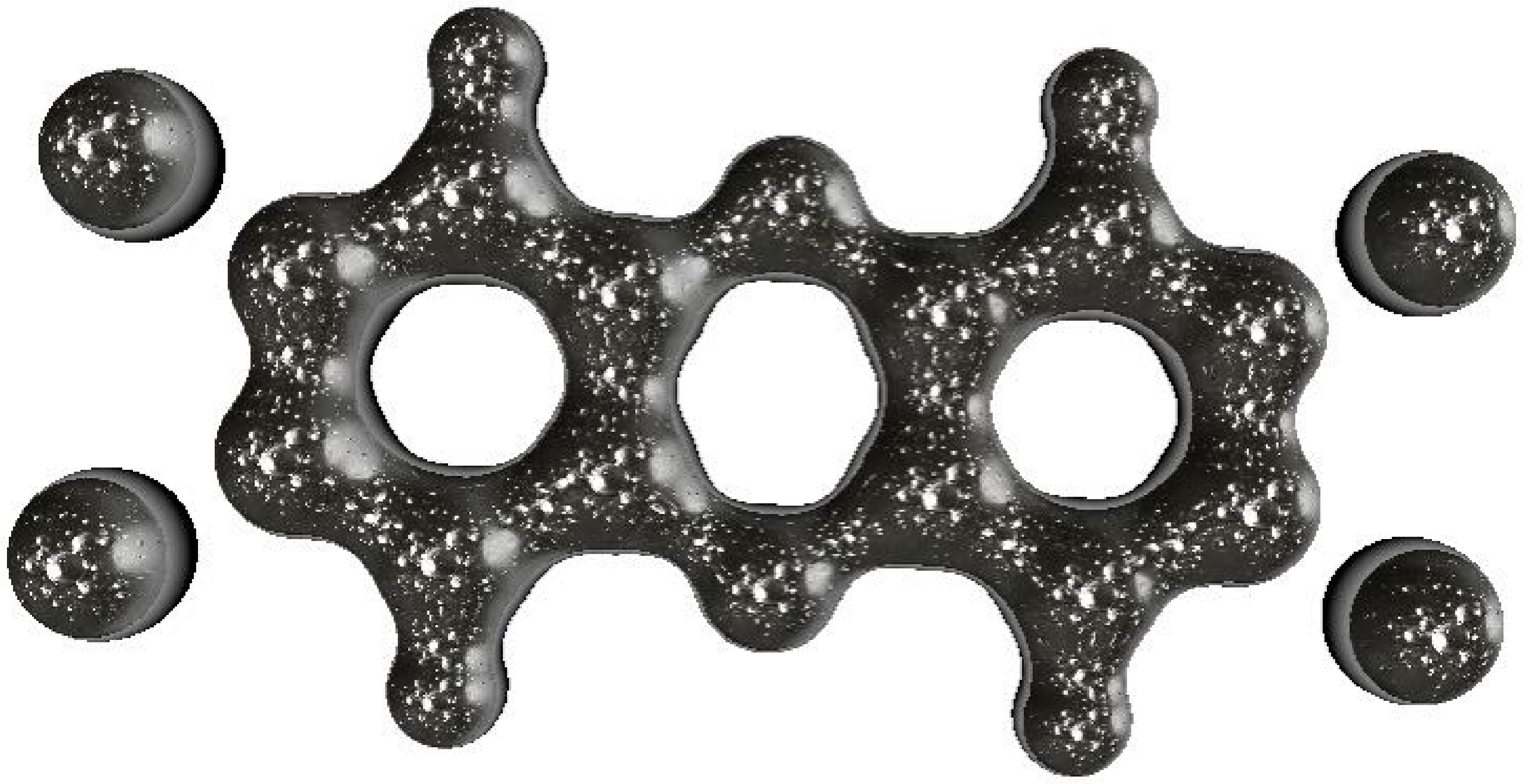

LIT-SPHÈRES
RENDU ARTISTIQUE DE
PROPRIÉTÉS MOLÉCULAIRES

ELSA DEPONT

NATURE DE LA MOLÉCULE:
EAU

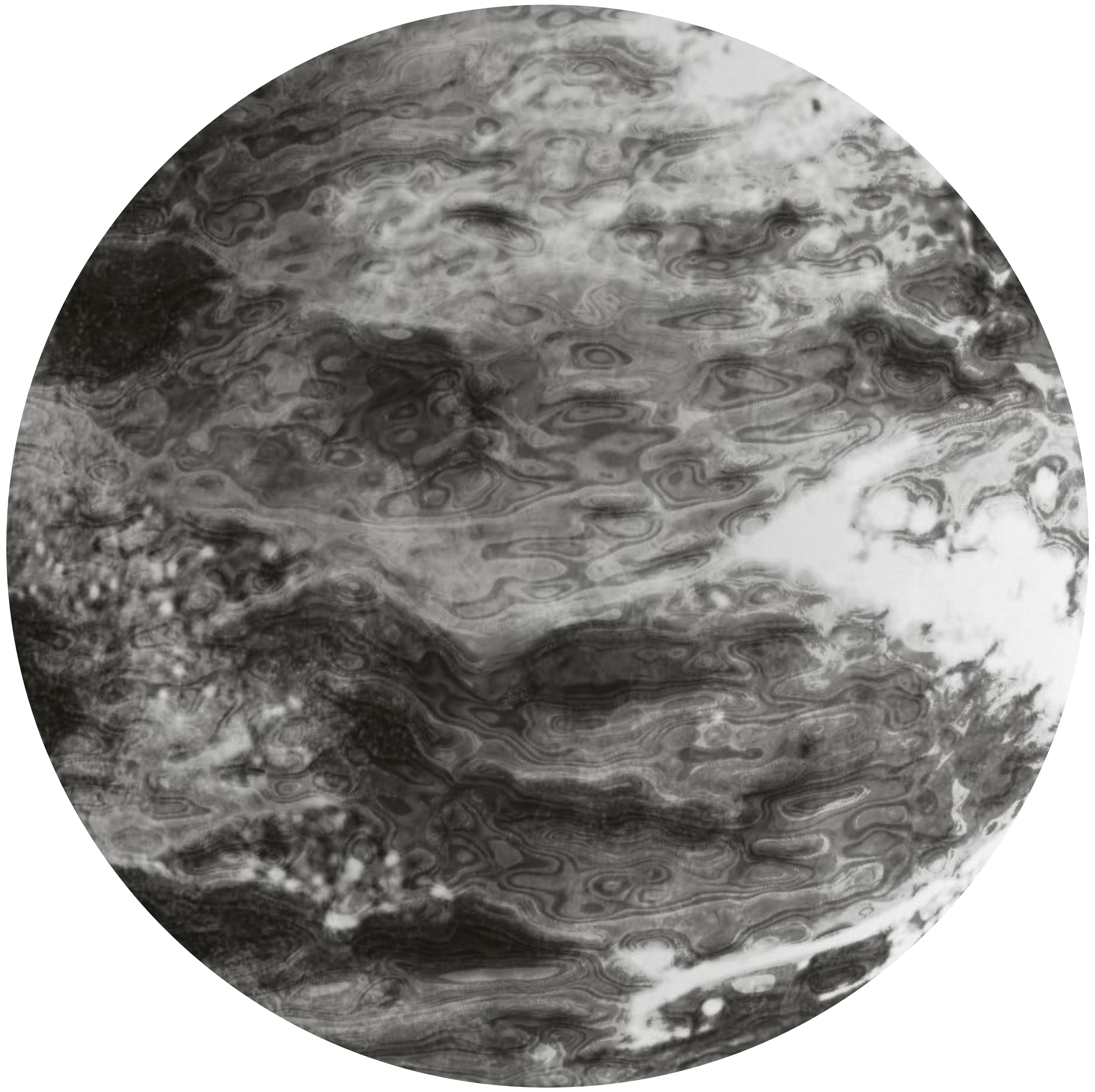

LIT-SPHÈRES
RENDU ARTISTIQUE DE
PROPRIÉTÉS MOLÉCULAIRES

ELSA DEPONT

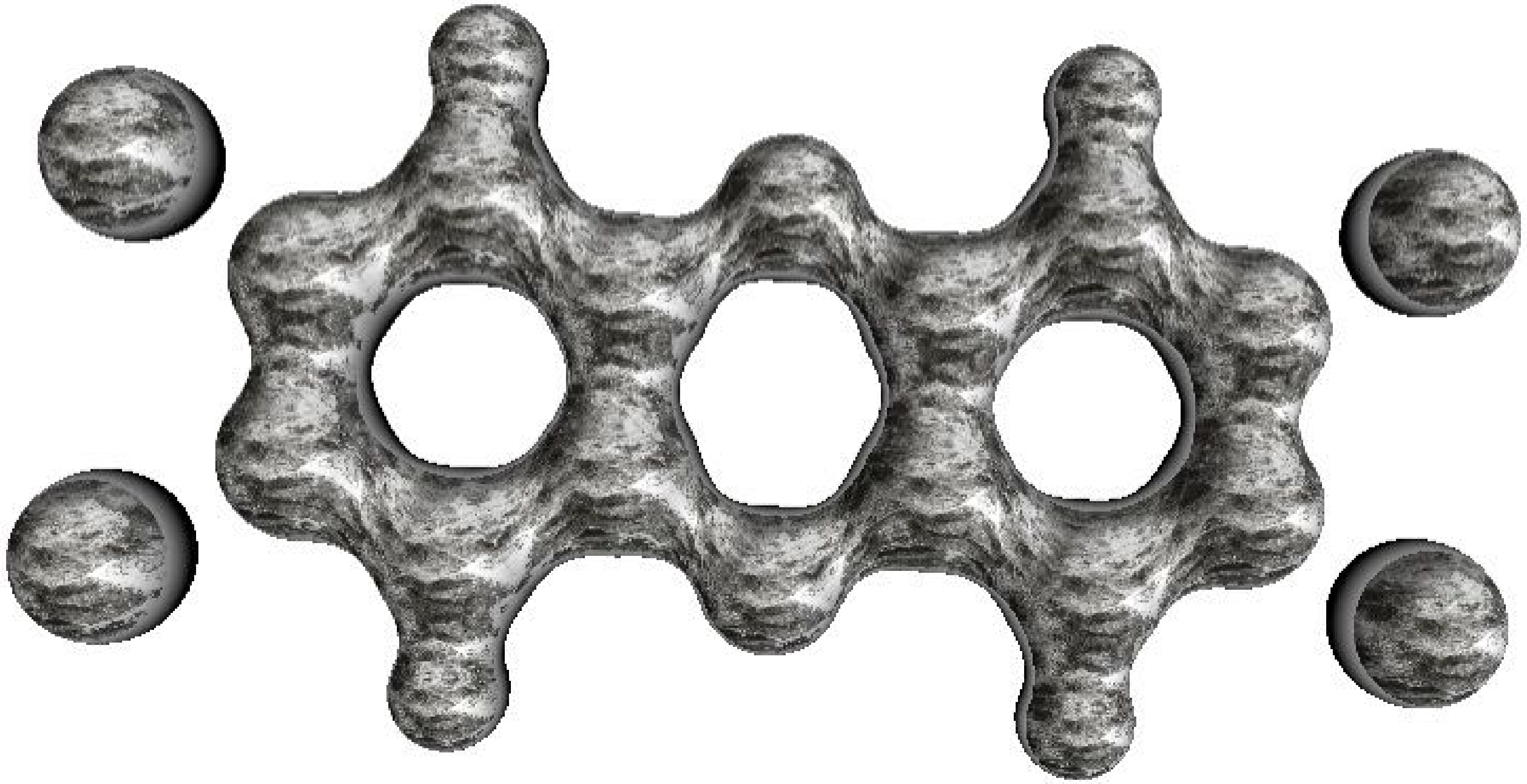

LIT-SPHÈRES
RENDU ARTISTIQUE DE
PROPRIÉTÉS MOLÉCULAIRES

ELSA DEPONT

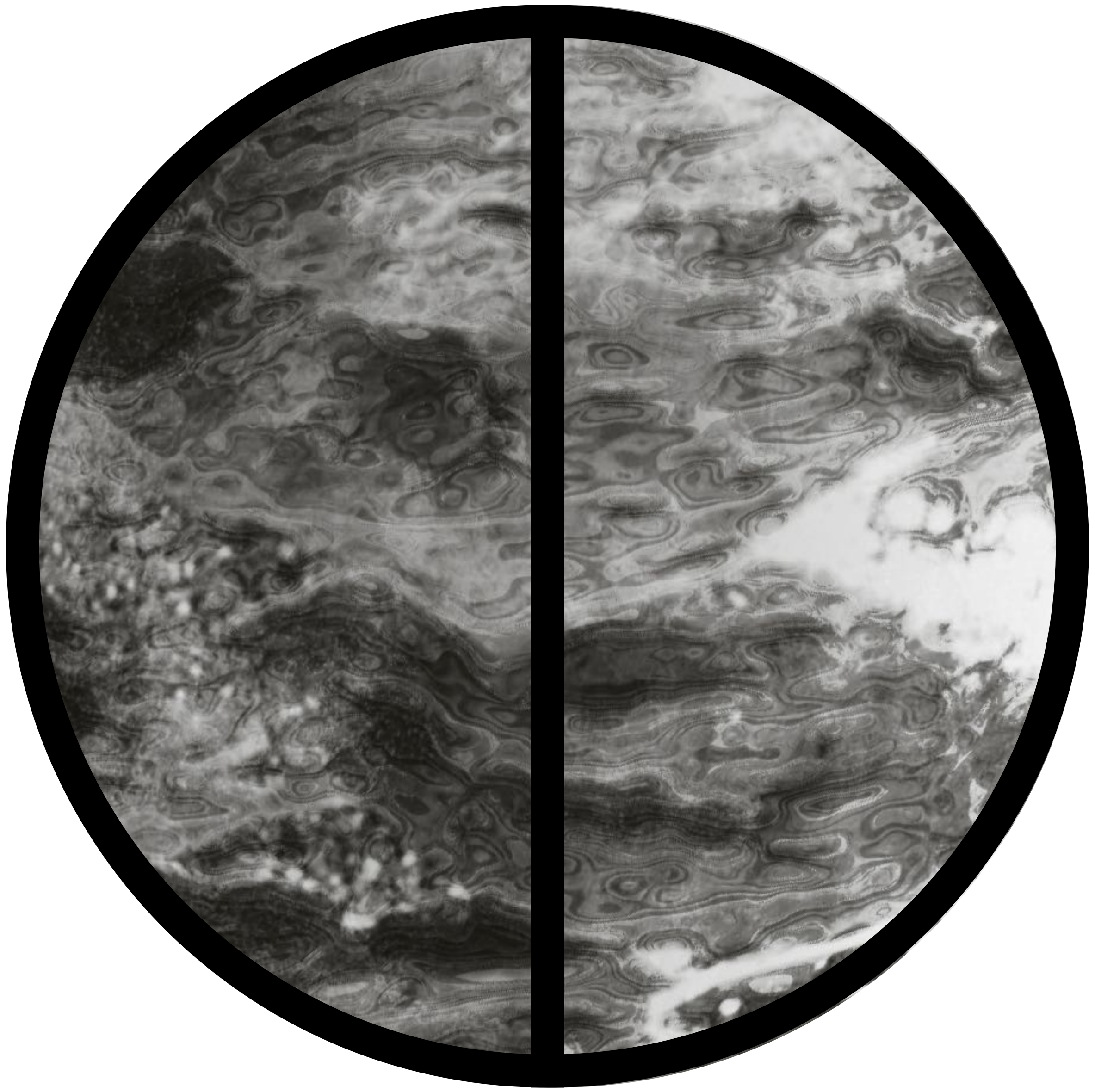

LIT-SPHÈRES
RENDU ARTISTIQUE DE
PROPRIÉTÉS MOLÉCULAIRES

ELSA DEPONT

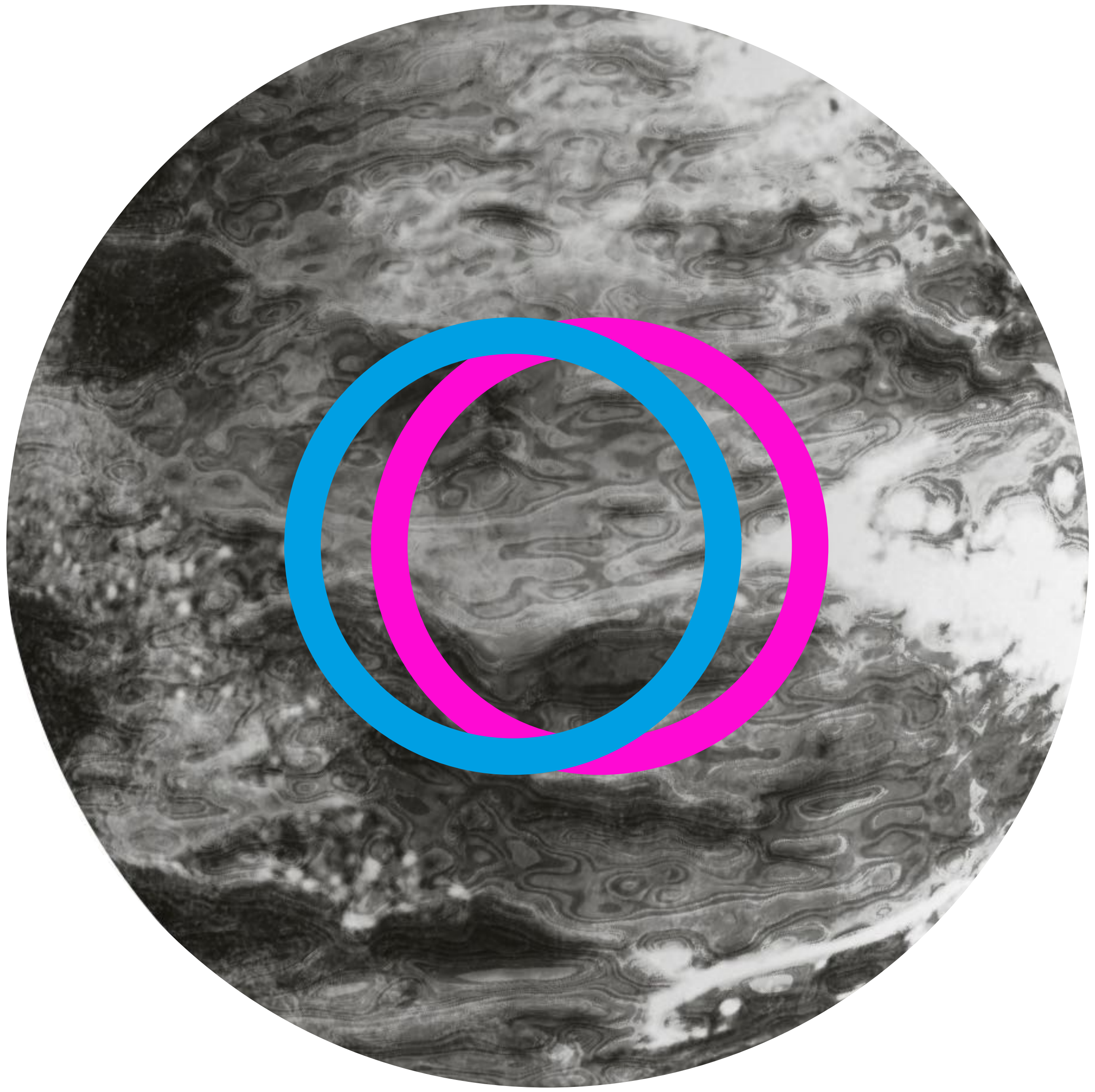

LIT-SPHÈRES
RENDU ARTISTIQUE DE
PROPRIÉTÉS MOLÉCULAIRES

MERCI !

JOANA GOUIN

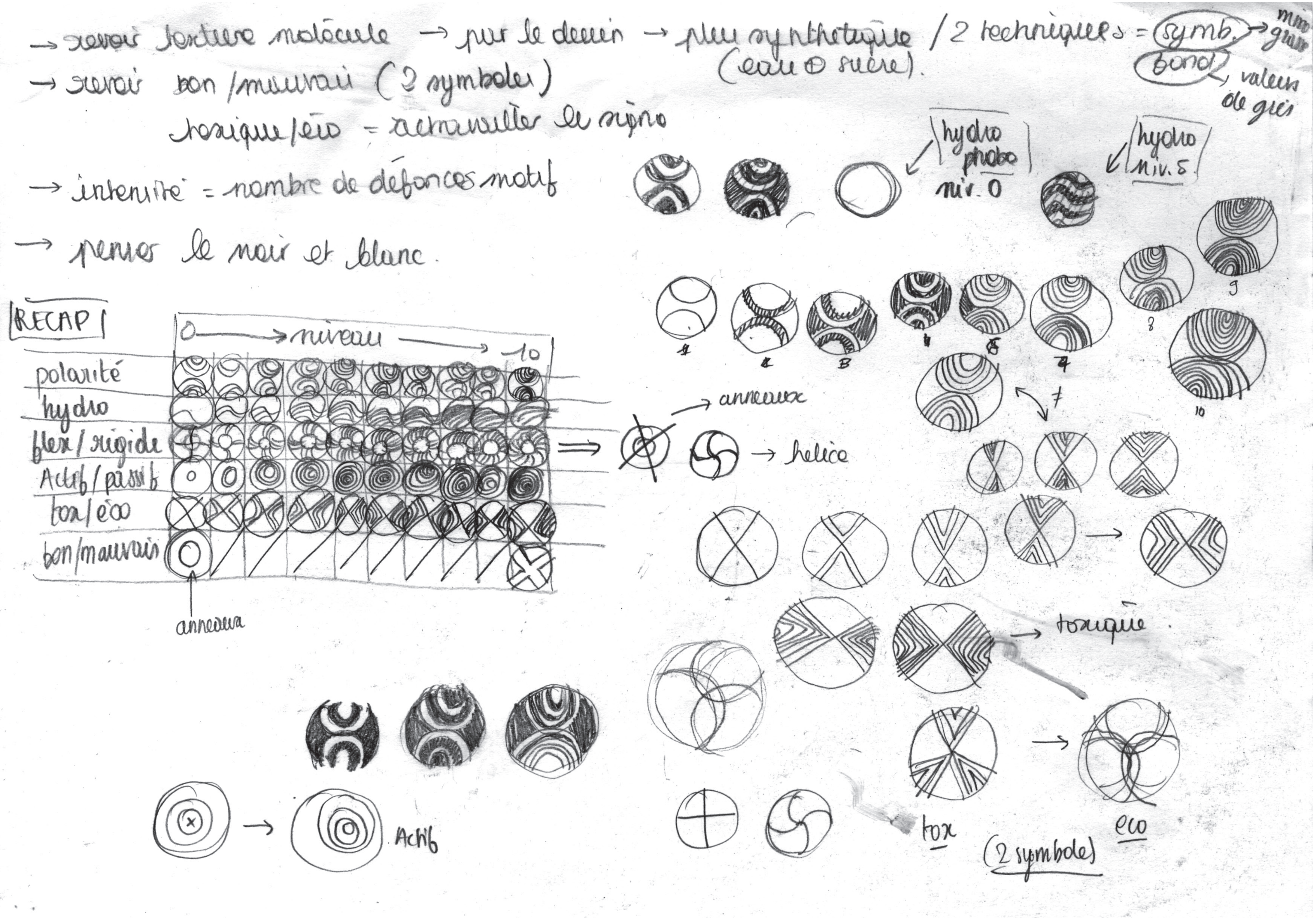
→ revoir texture molécule → par le dessin → plus synthétique (eau ⊕ sucre) / 2 techniques = symb. → mini gris
bond ← valeur de gris
→ revoir bon / mauvais (2 symboles)
toxique / éco = retravailler le signe
→ intensité = nombre de
→ penser le noir et blanc.
hydro phobo
niv. 0
hydro niv. 5
RECAP
0 → niveau → 10
polarité
hydro
flex / rigide
Actif / passif
tox / éco
bon / mauvais
anneaux
→ anneaux
→ hélice
→ toxique
Actif
tox
eco
(2 symbole)

JOANA GOUIN

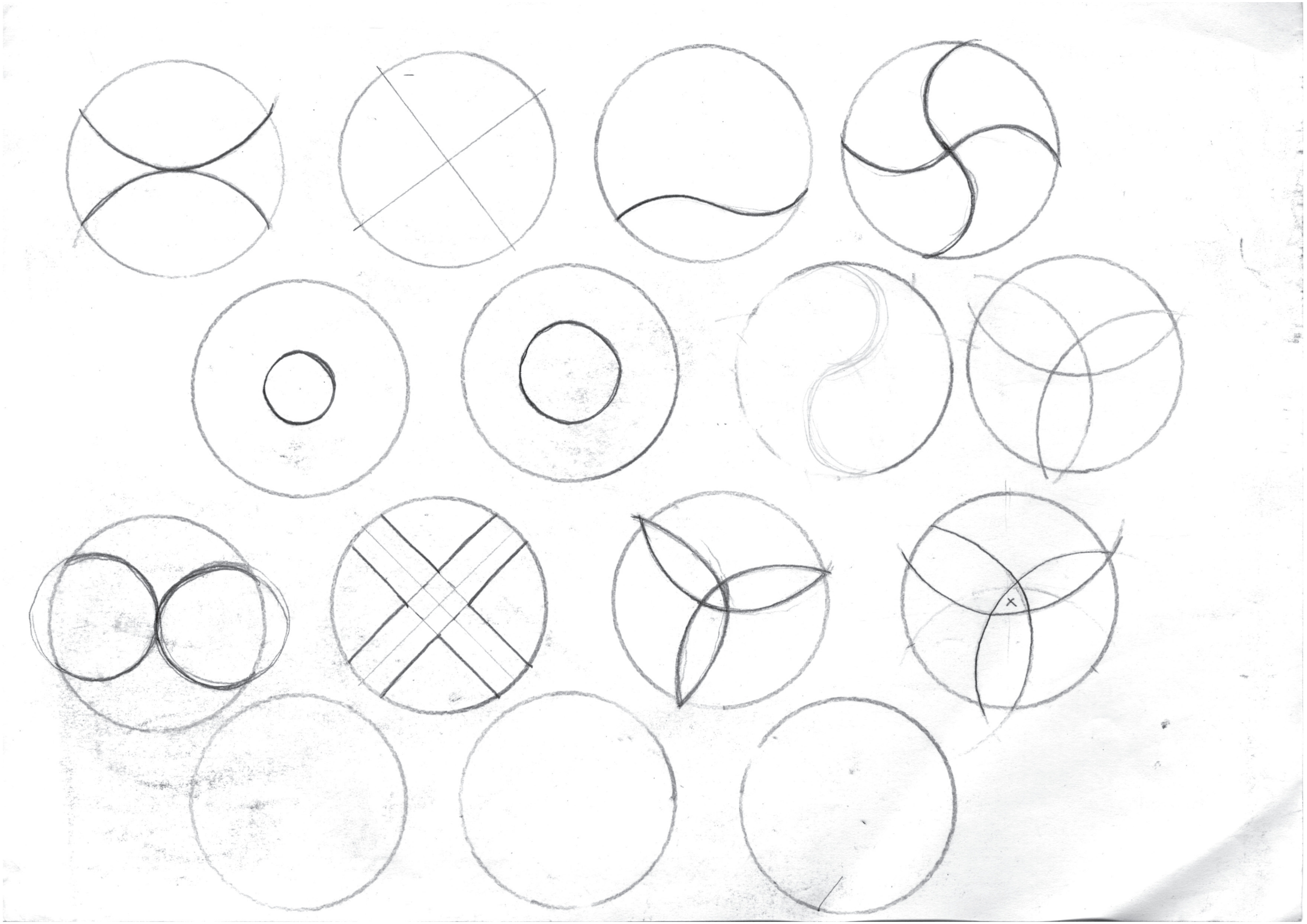

JOANA GOUIN

>> POSITIF / NÉGATIF

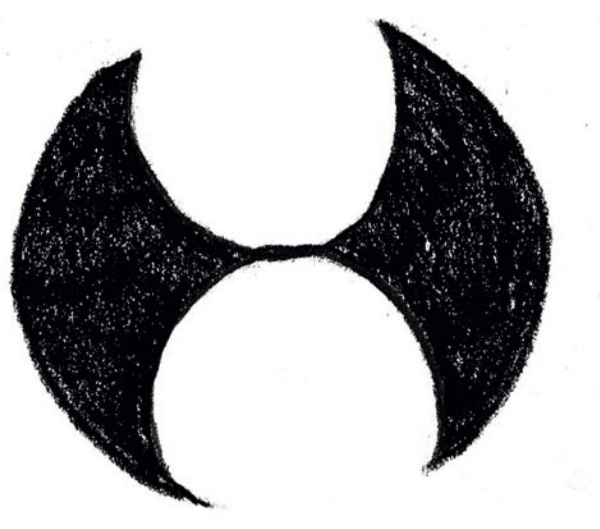

niveau 1

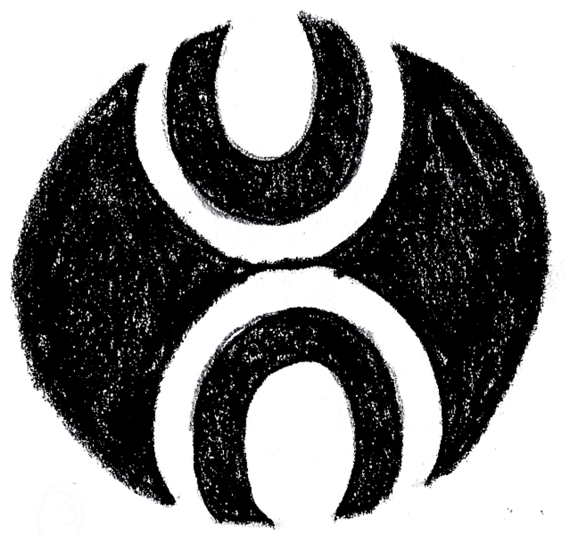

niveau 3

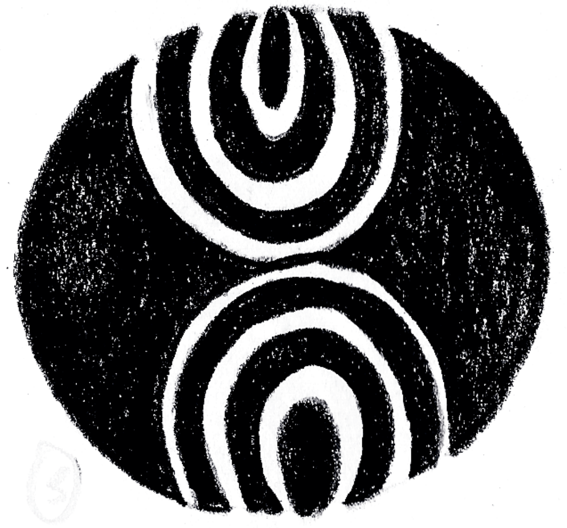

niveau 5

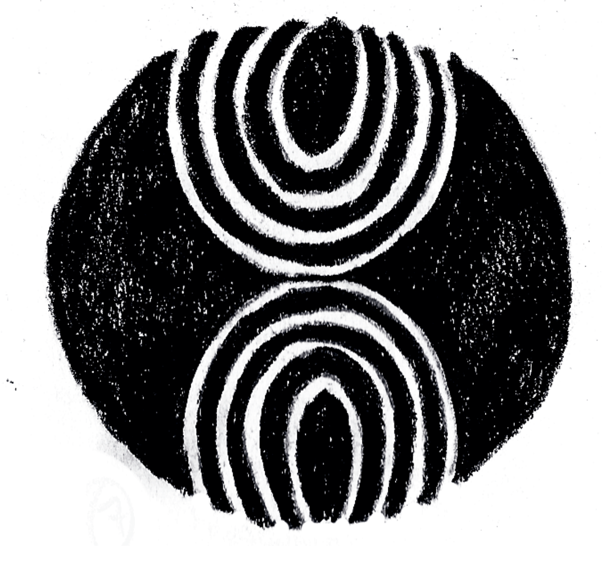

niveau 7

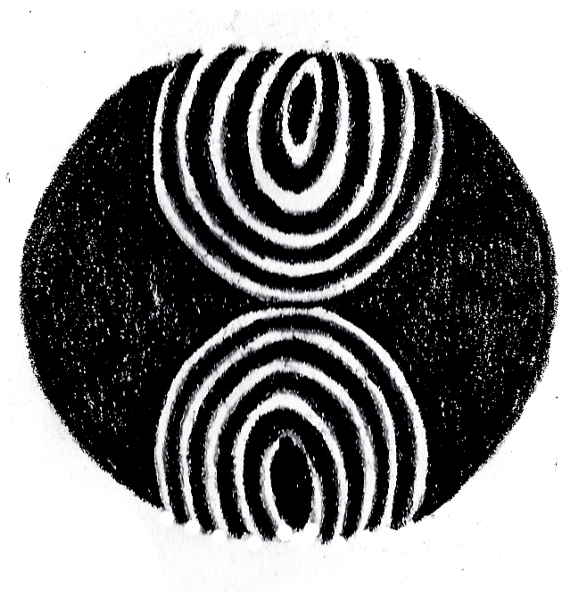

niveau 10

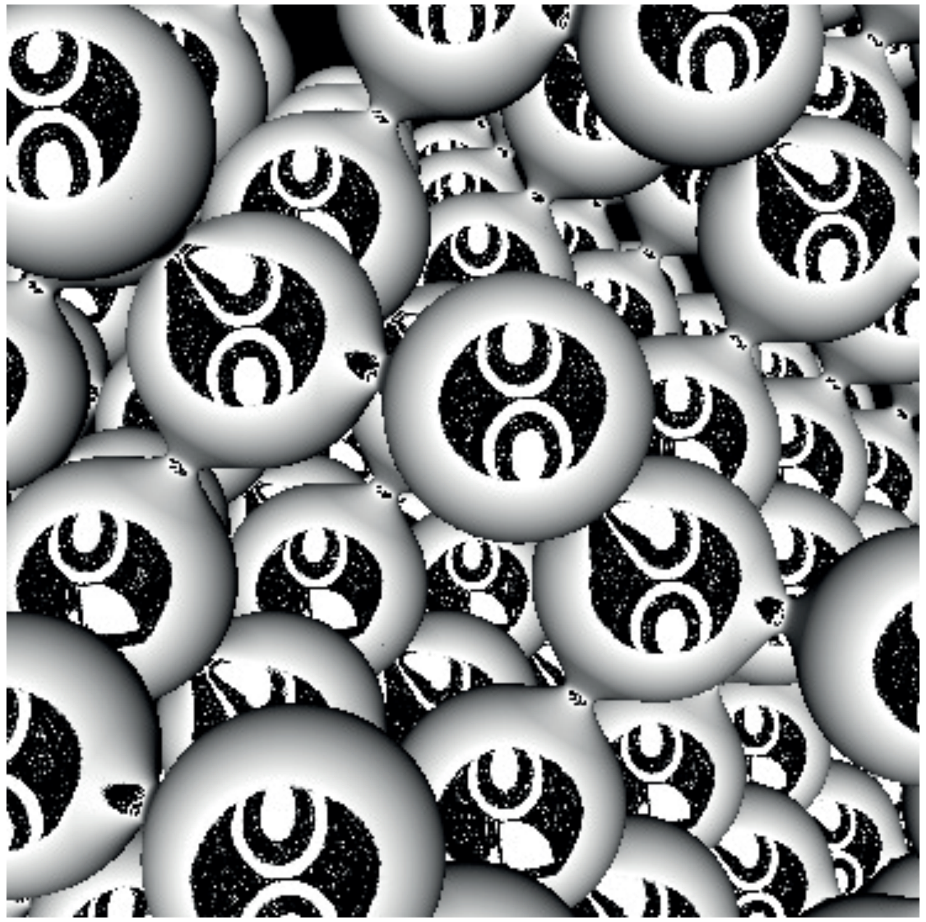

JOANA GOUIN

>> HYDROPHILIE / HYDROPHOBIE

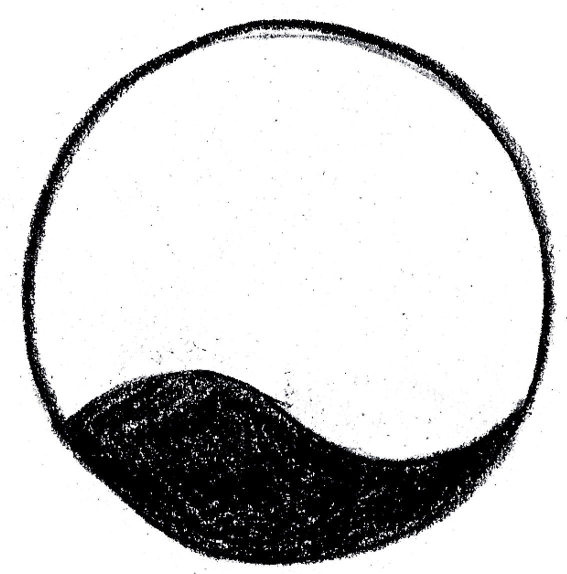
niveau 1

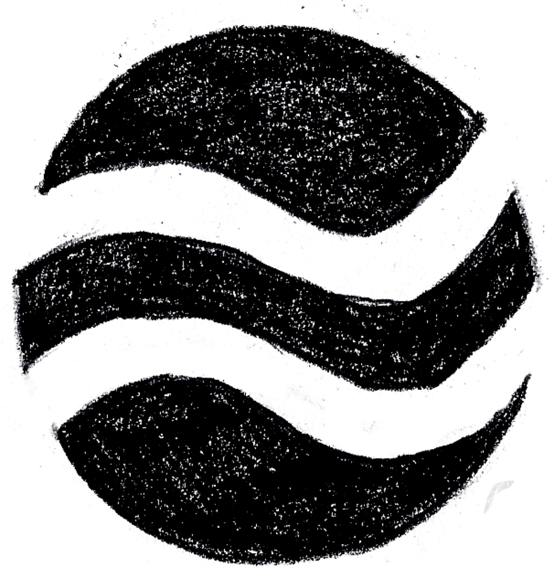
niveau 3

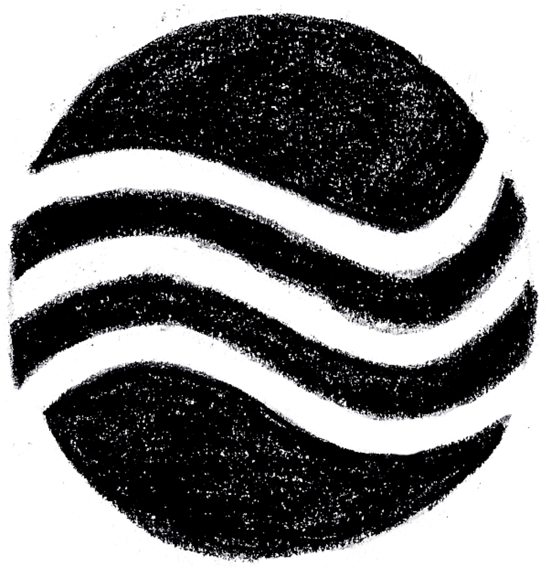
niveau 5

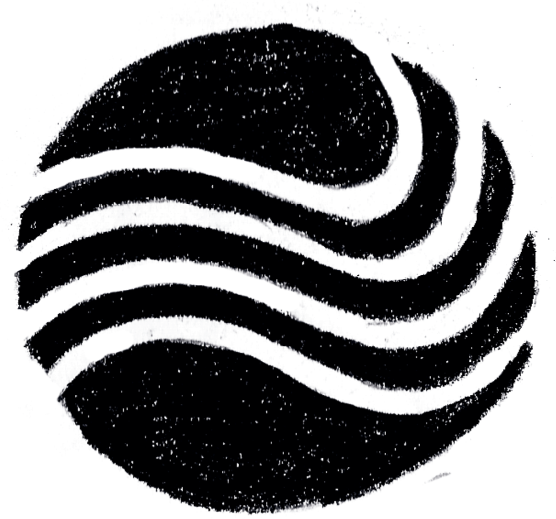
niveau 7

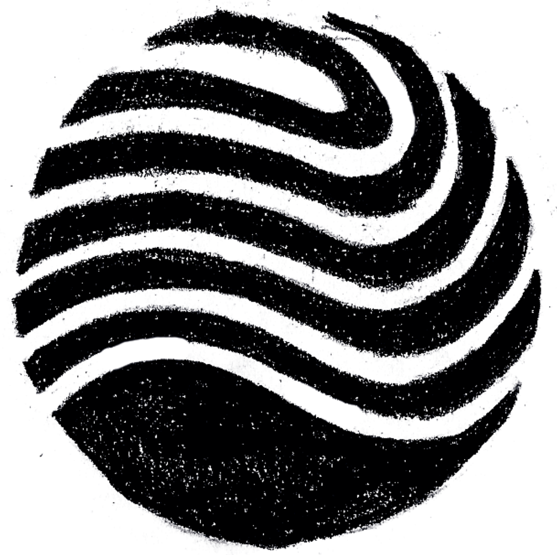
niveau 10

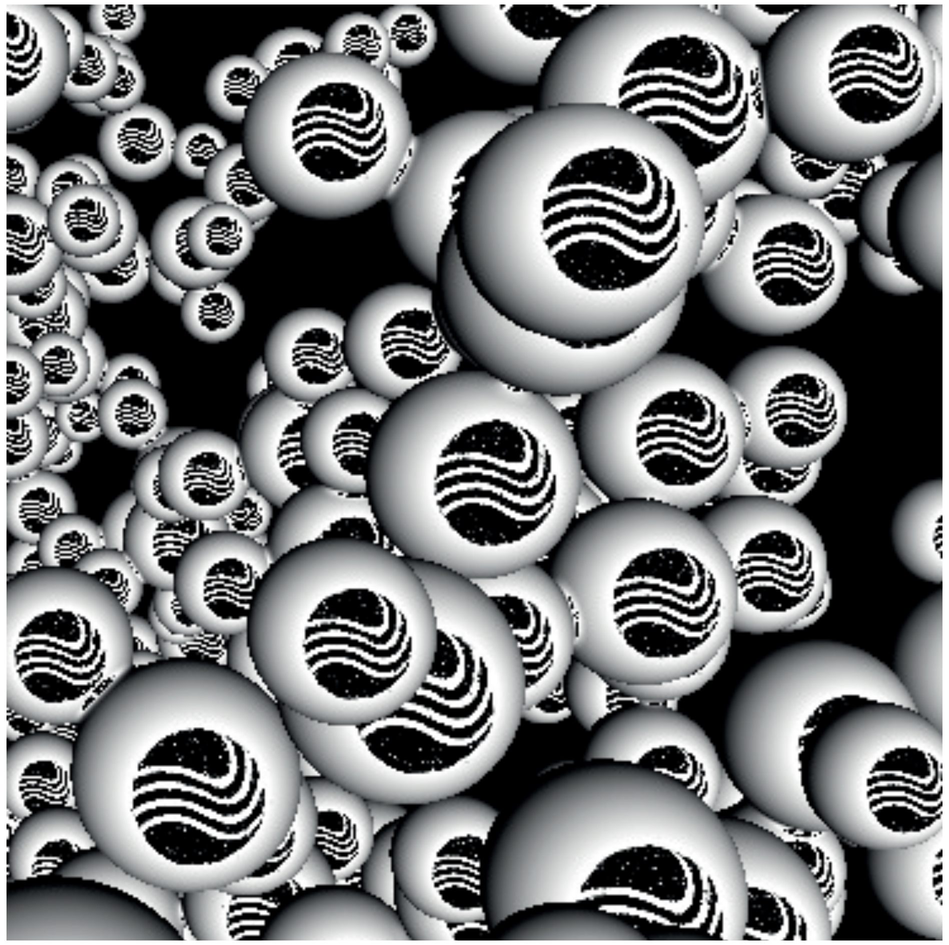

JOANA GOUIN

>> ÉCOCOMPATIBILITÉ

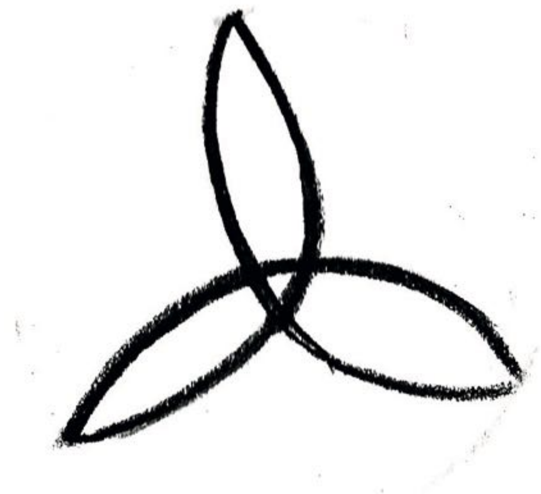

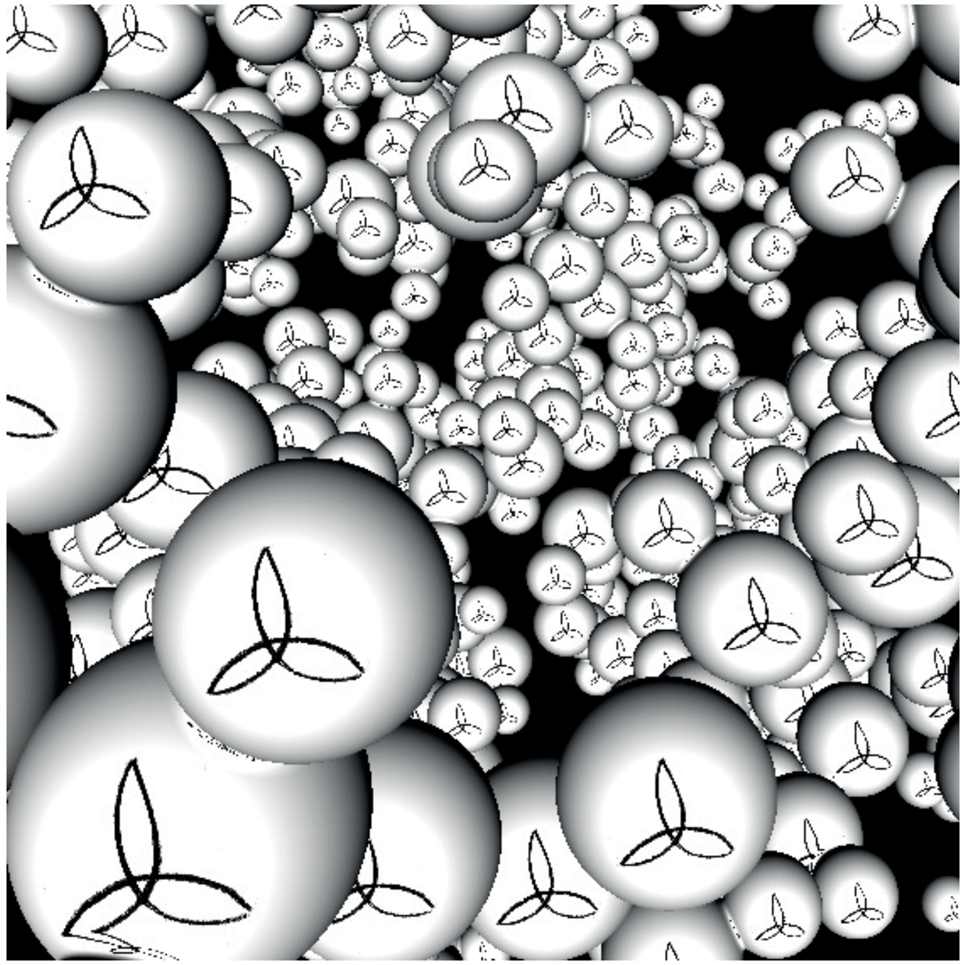

>> TOXICITÉ

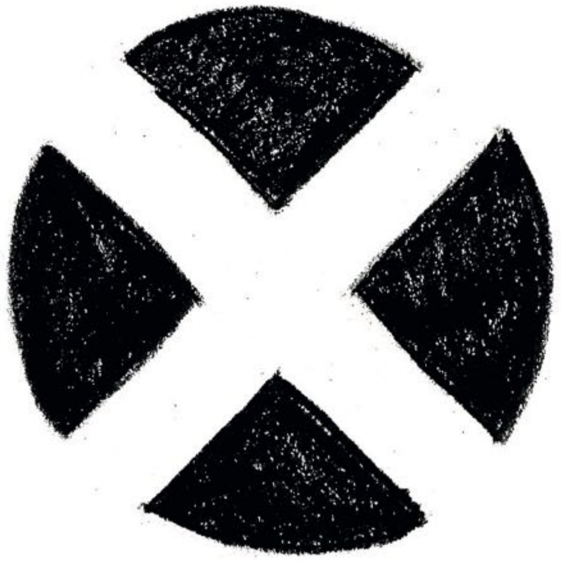

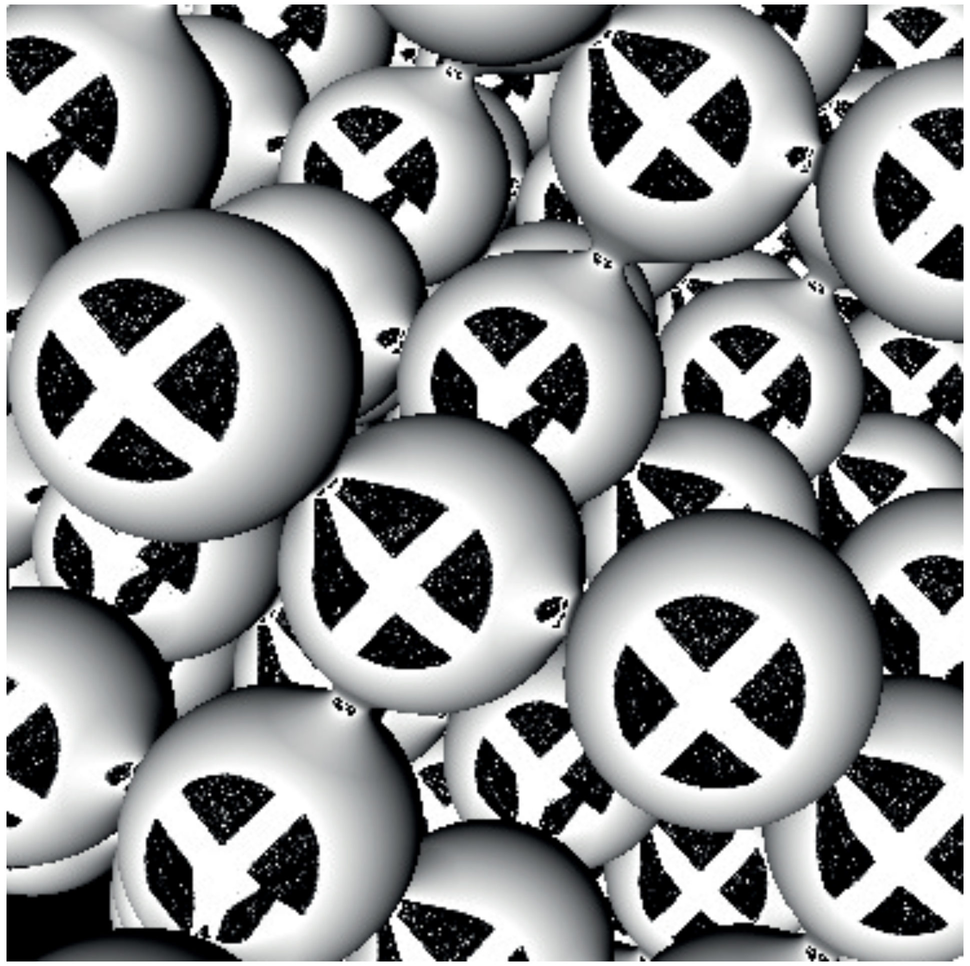

>> FLEXIBILITÉ / RIGIDITÉ

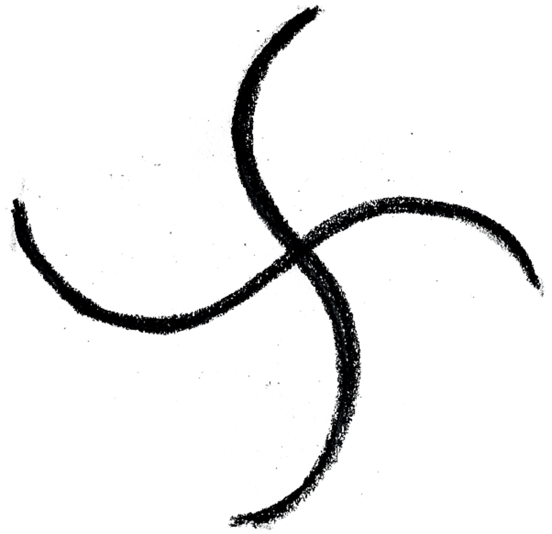

niveau 1

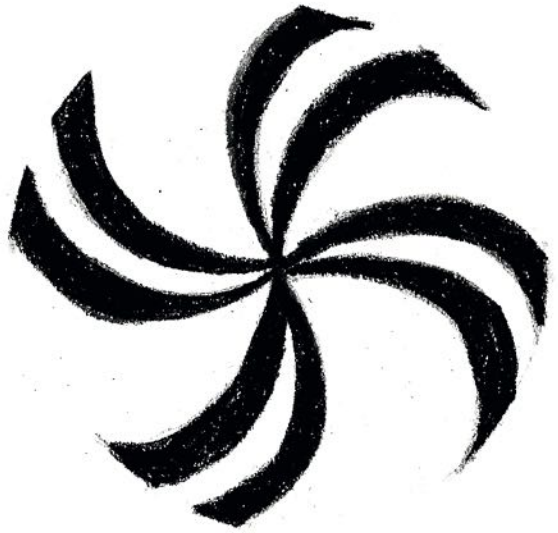

niveau 3

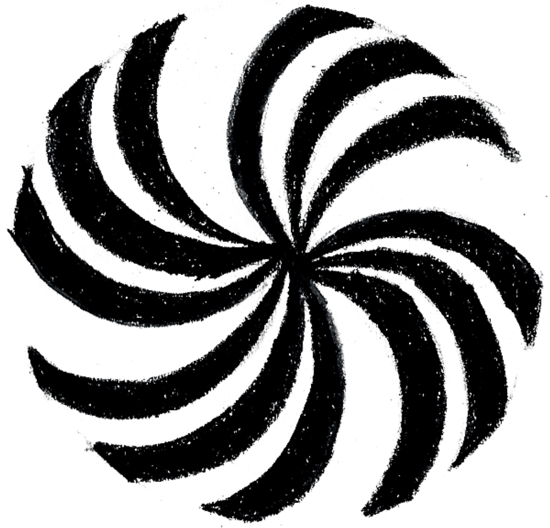

niveau 5

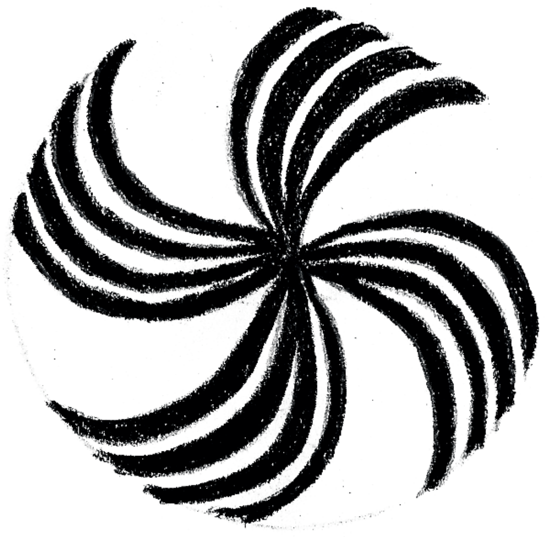

niveau 7

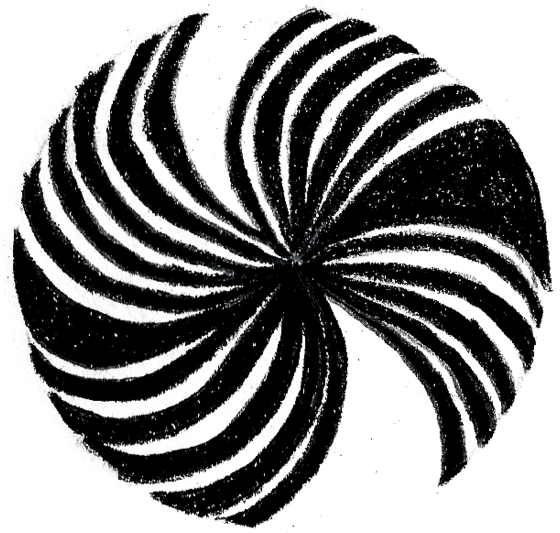

niveau 10

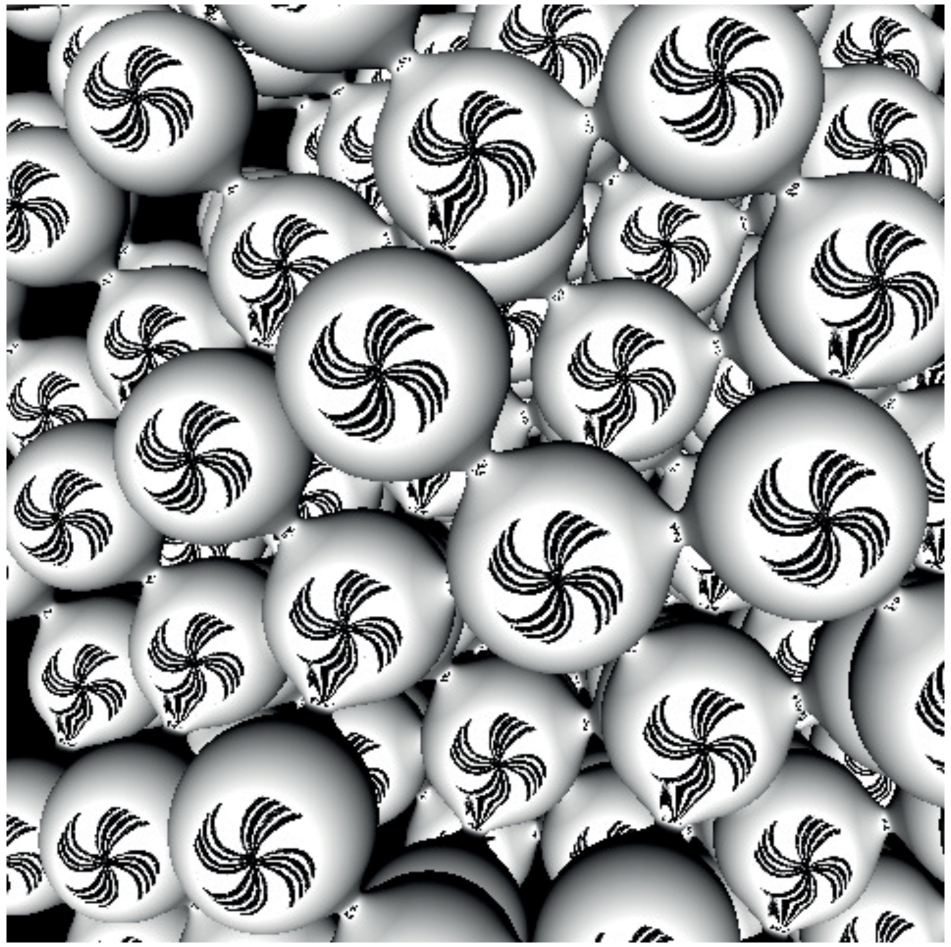

>> ACTIF / PASSIF

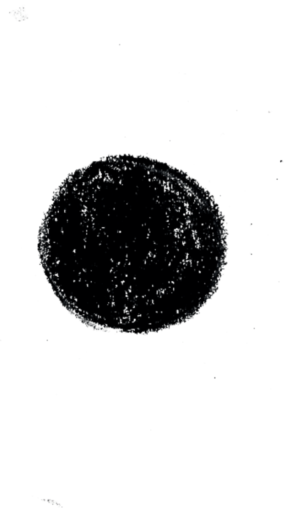

niveau 1

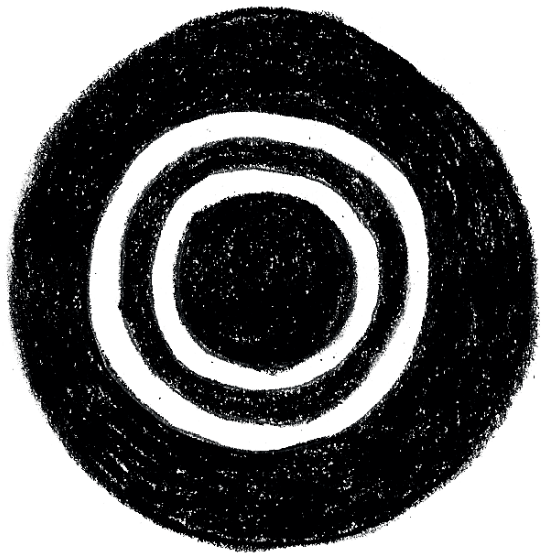

niveau 3

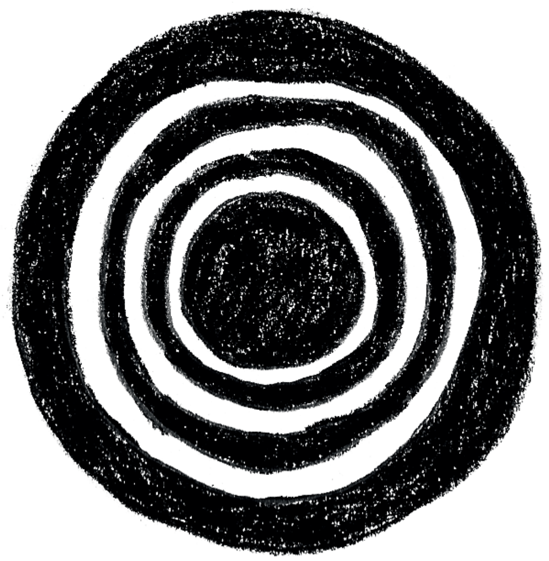

niveau 5

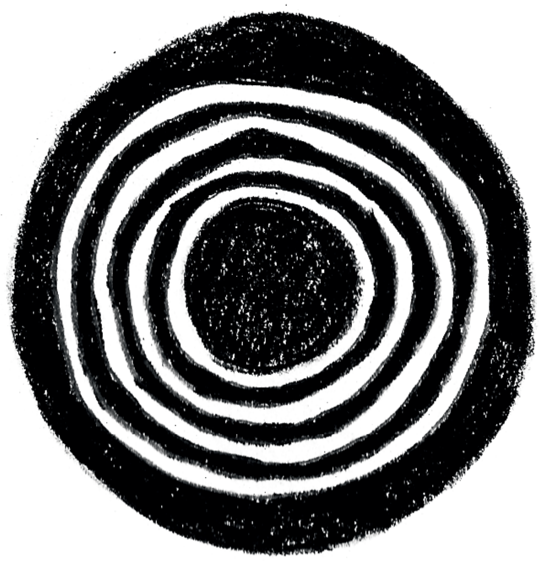

niveau 7

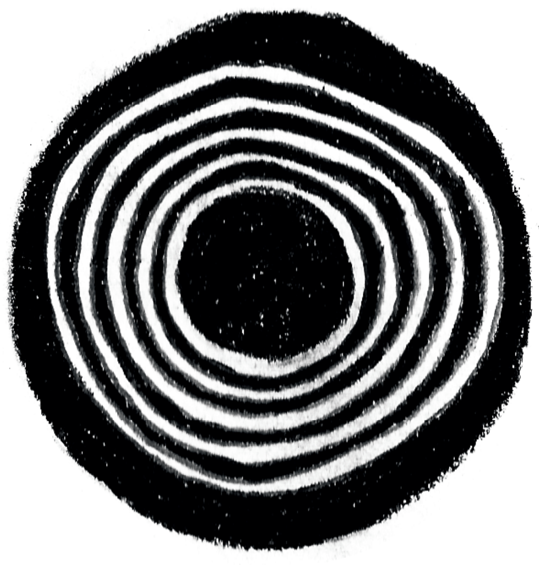

niveau 10

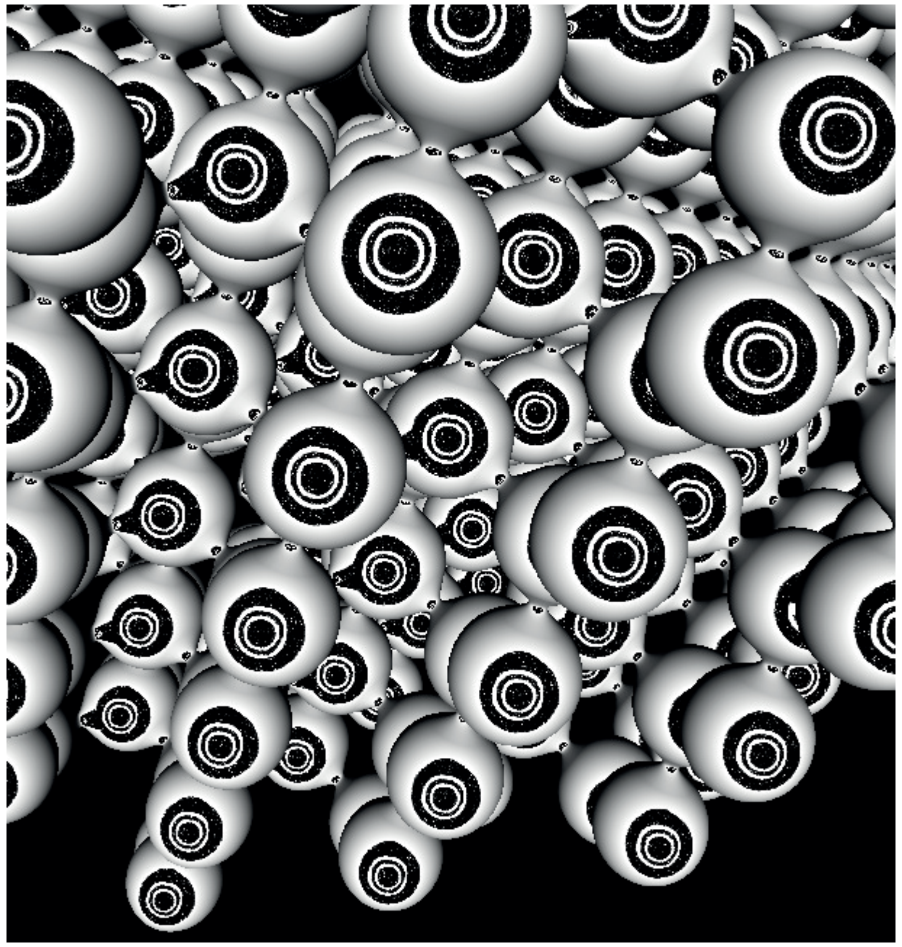

>> QUALITÉ

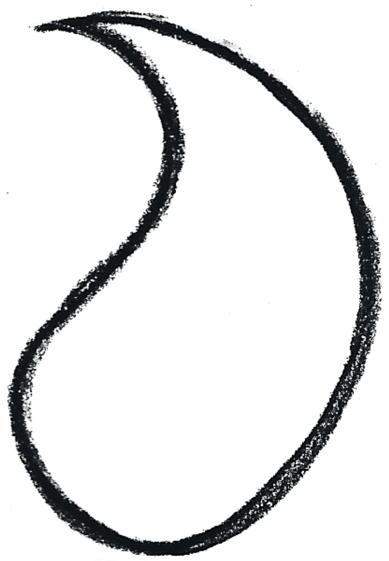

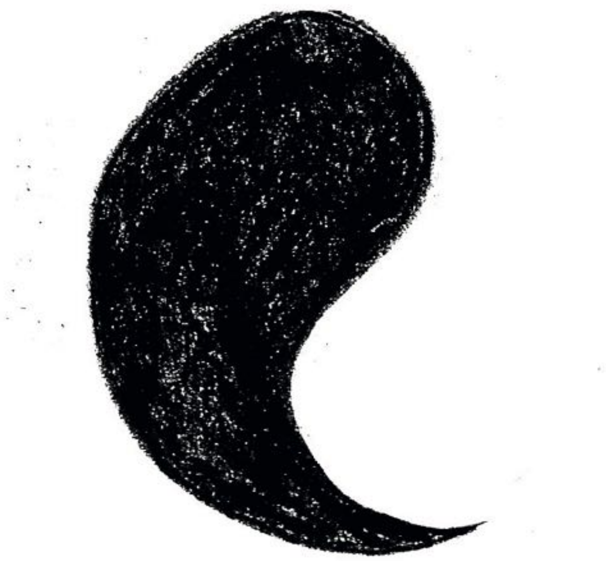

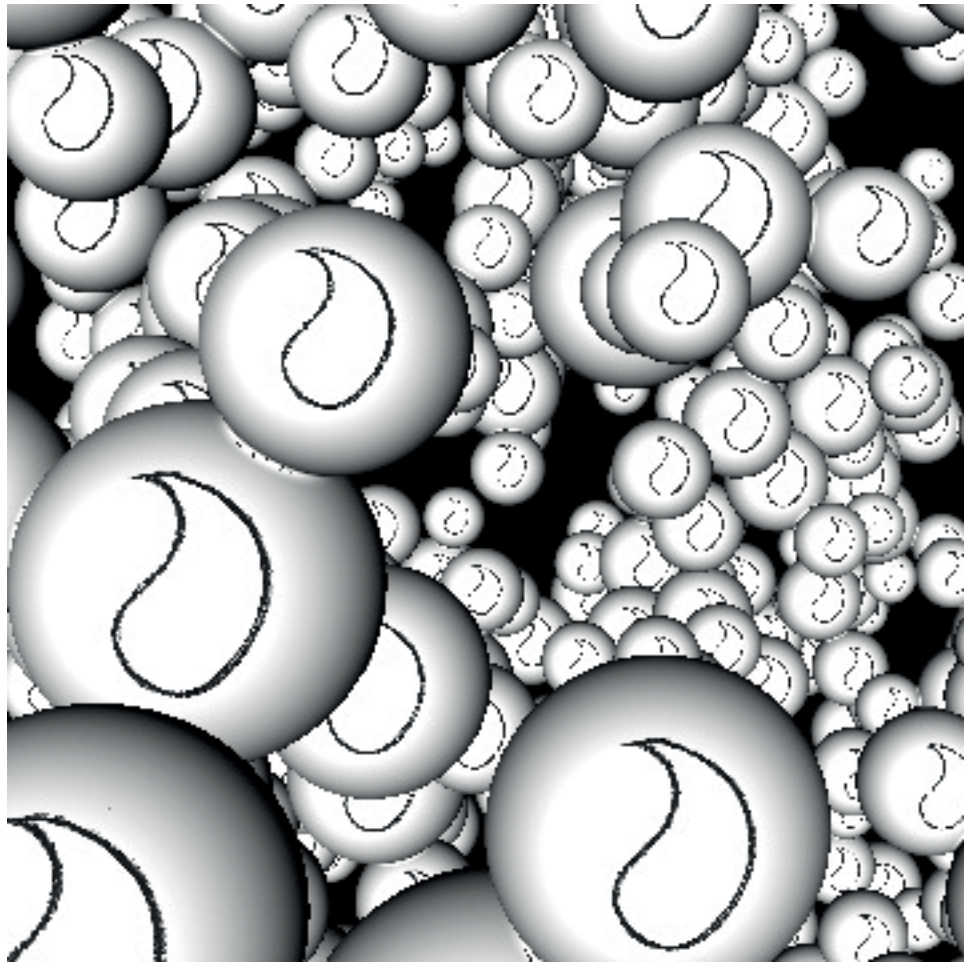

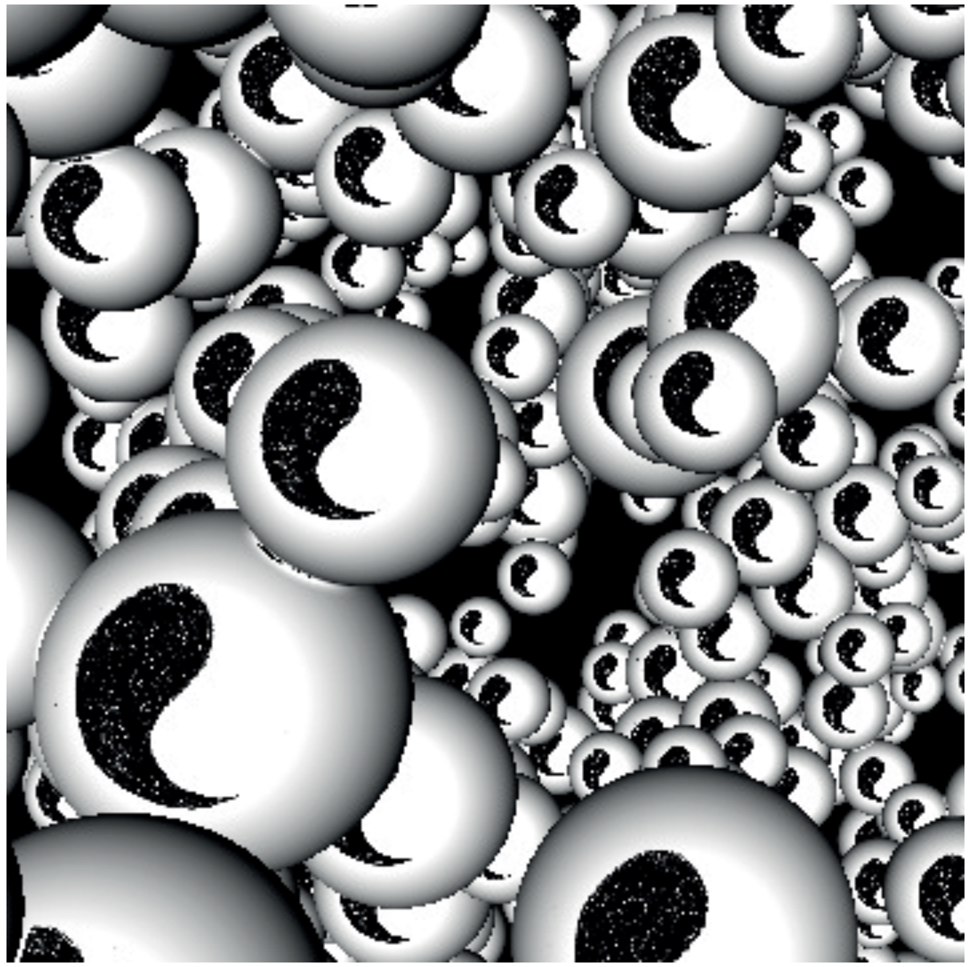

JOANA GOUIN

>> NATURE DE L'ATOME

Souffre

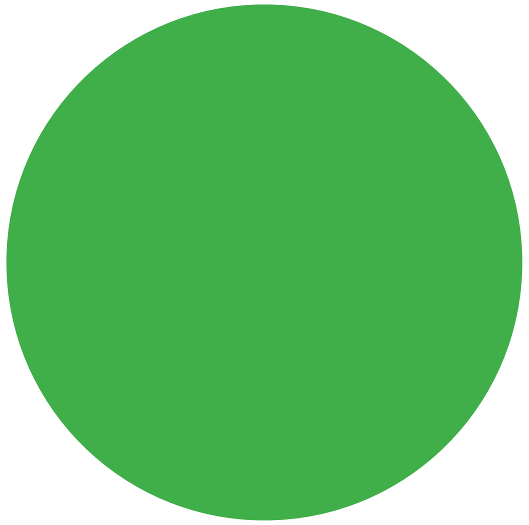

Nitrogène

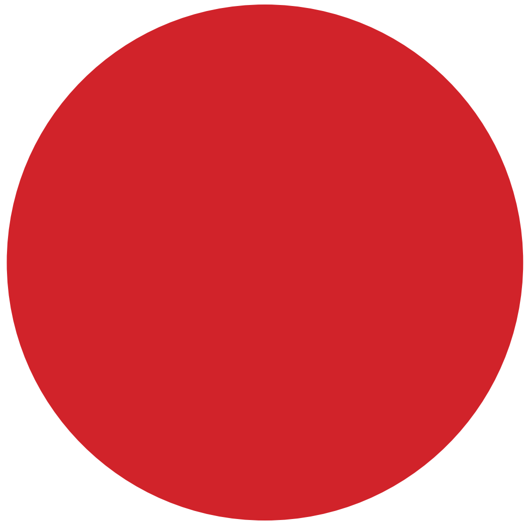

Oxygène

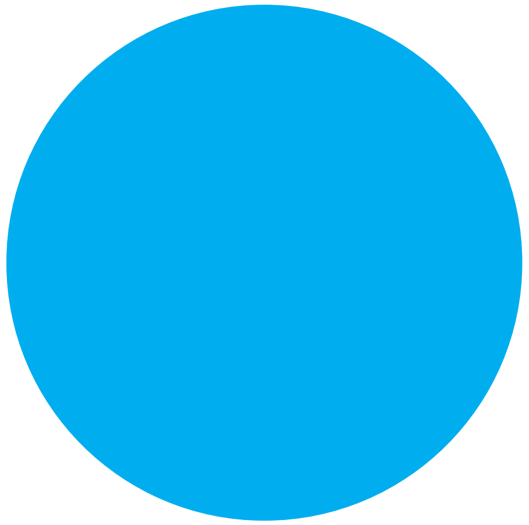

Azote

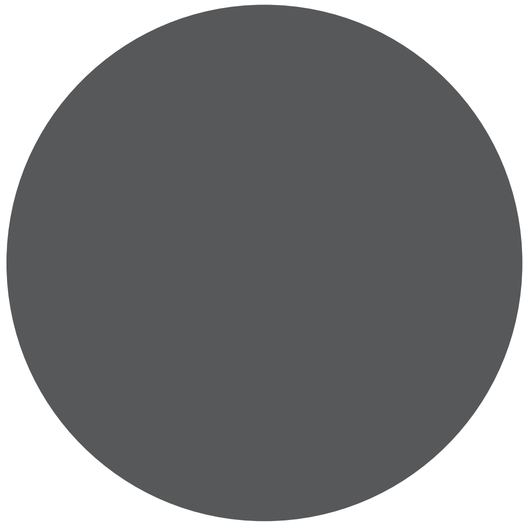

Carbone

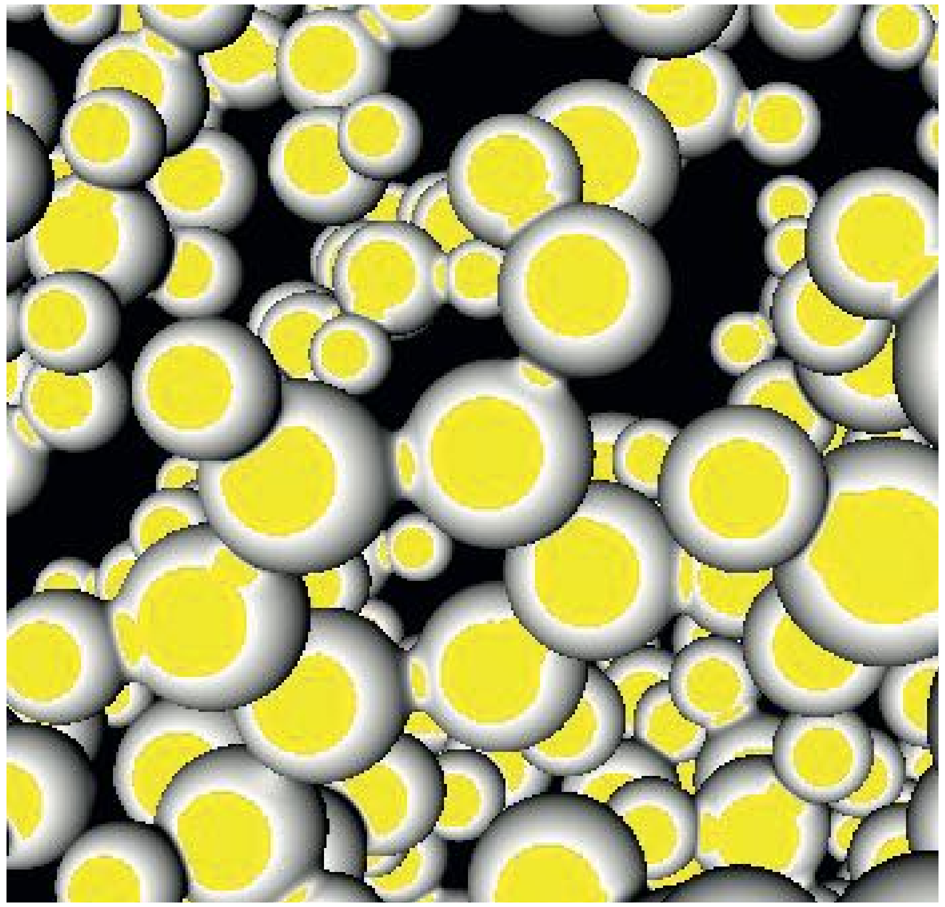

JOANA GOUIN

>> NATURE DE LA MOLÉCULE

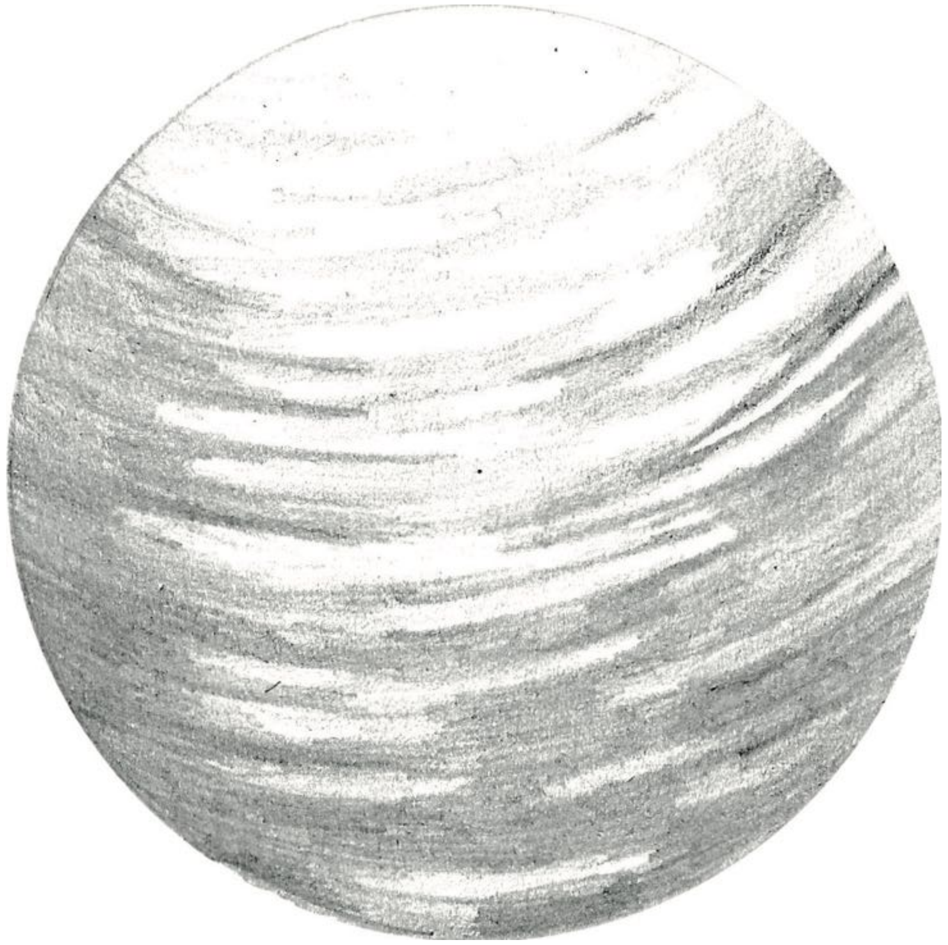

eau

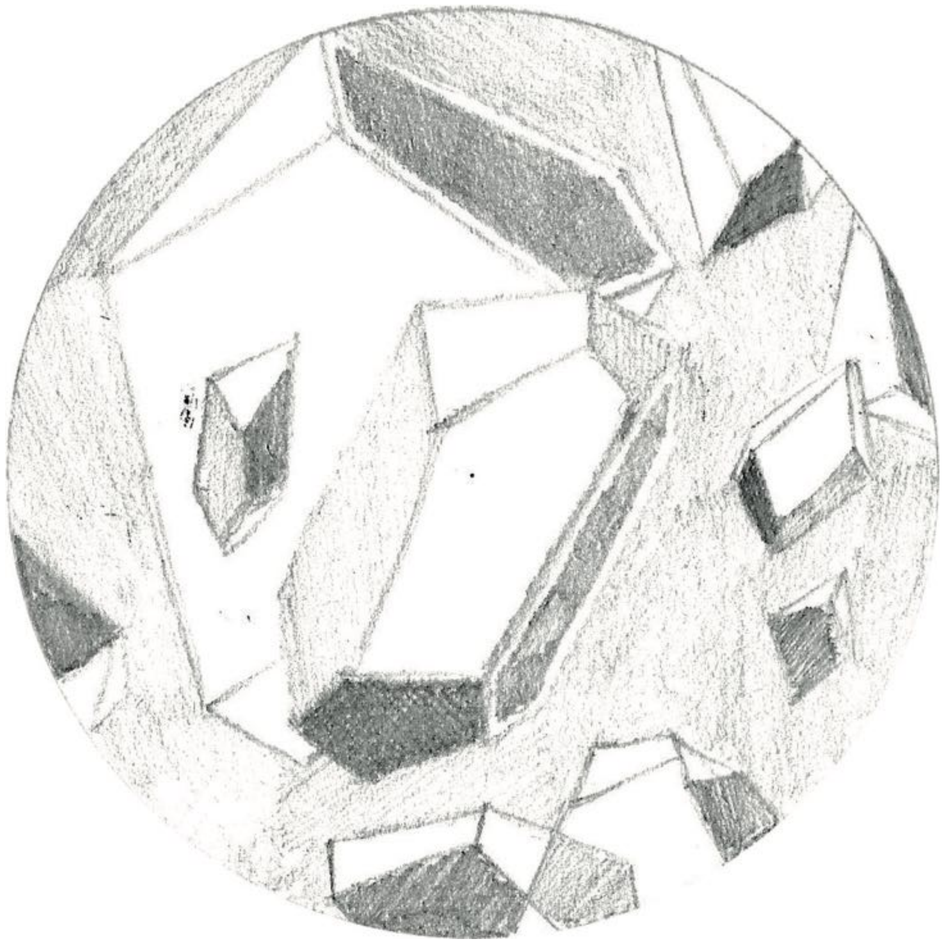

sucre

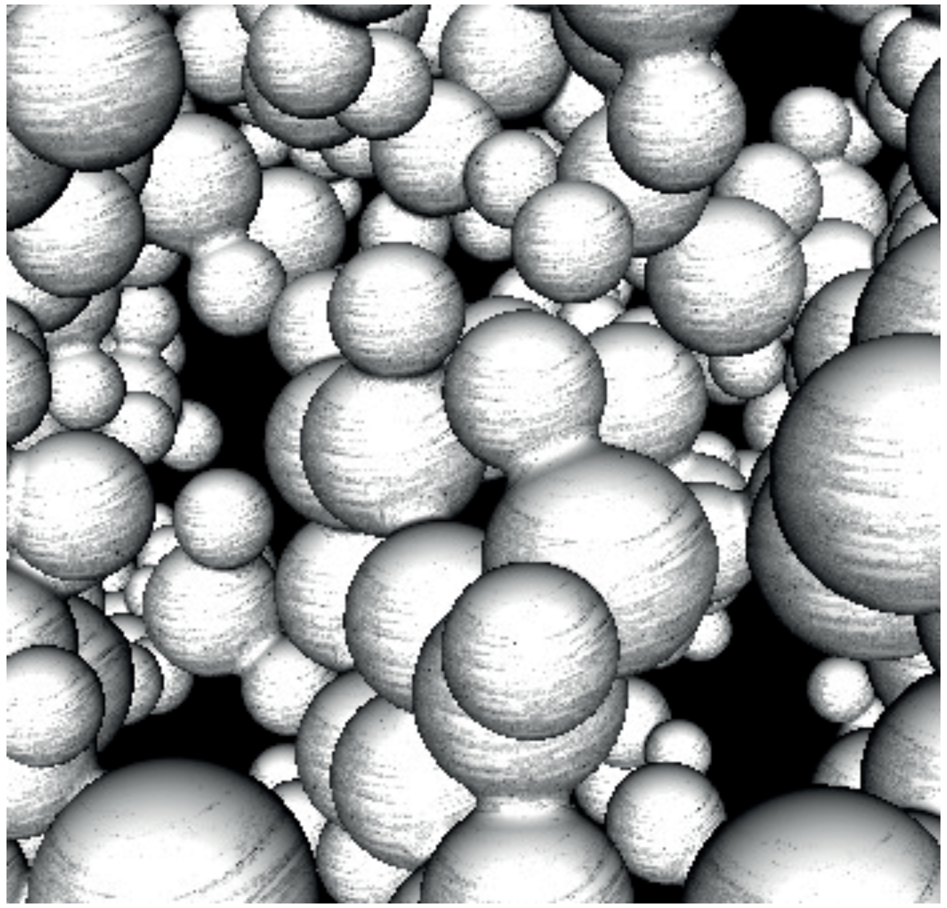

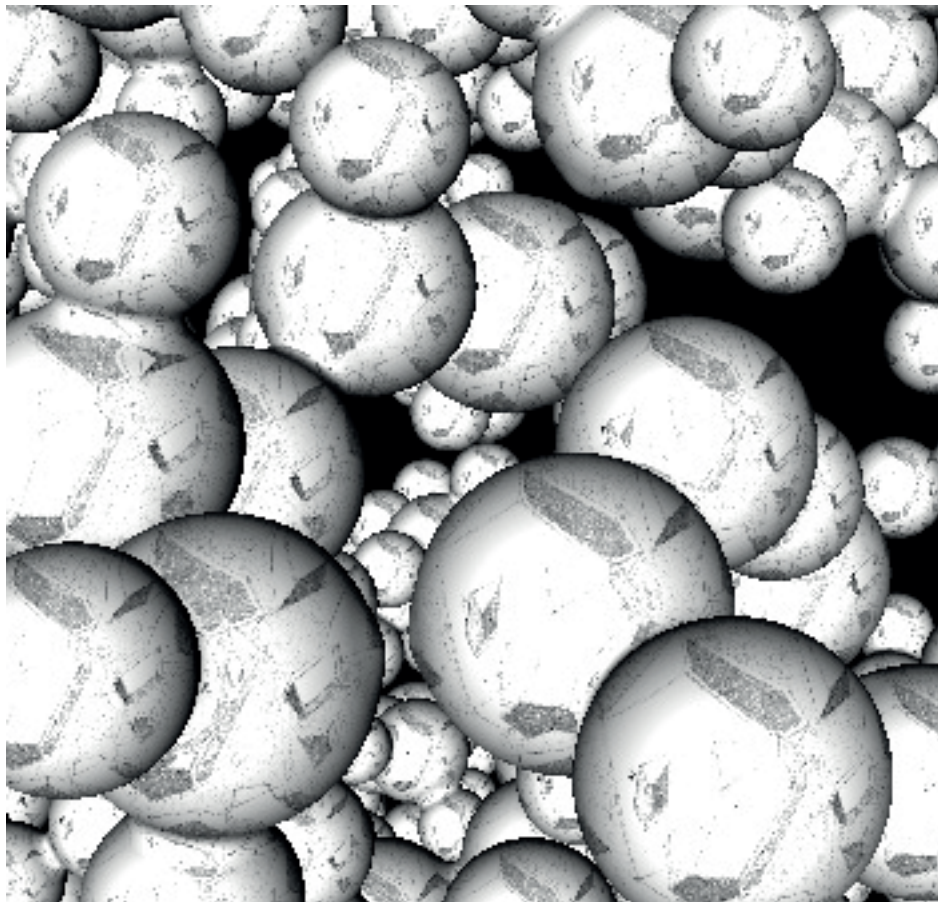

>> EXEMPLE D'INTÉGRATION

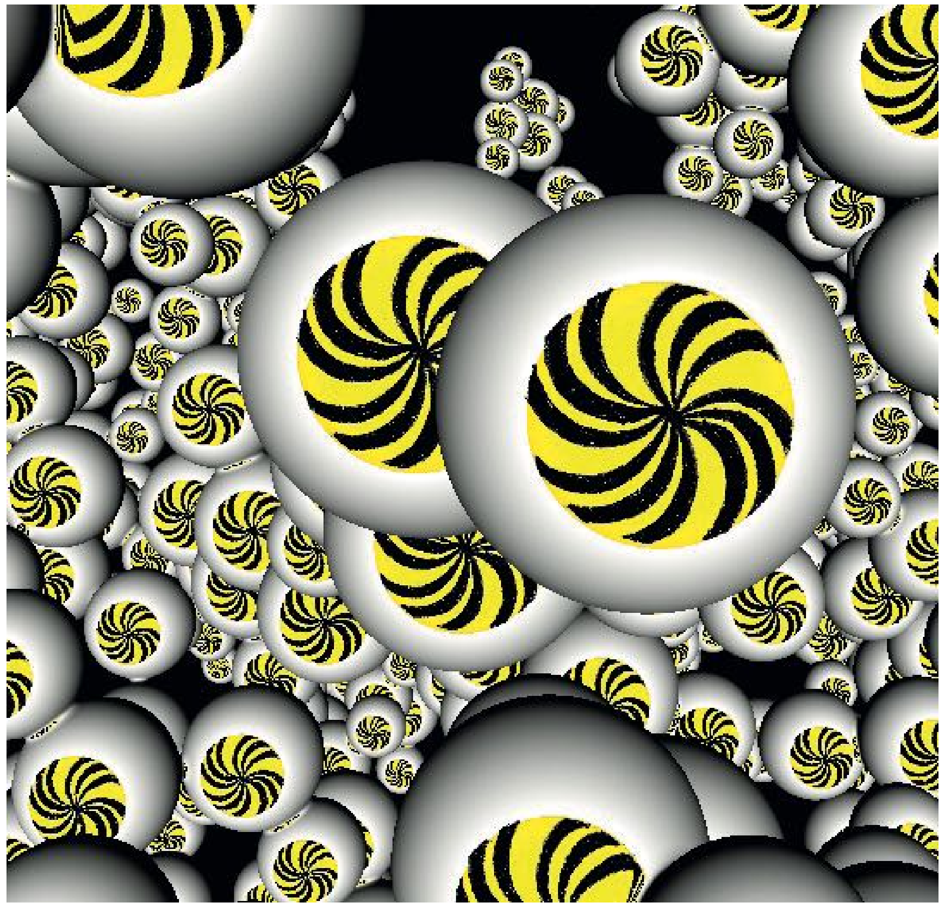
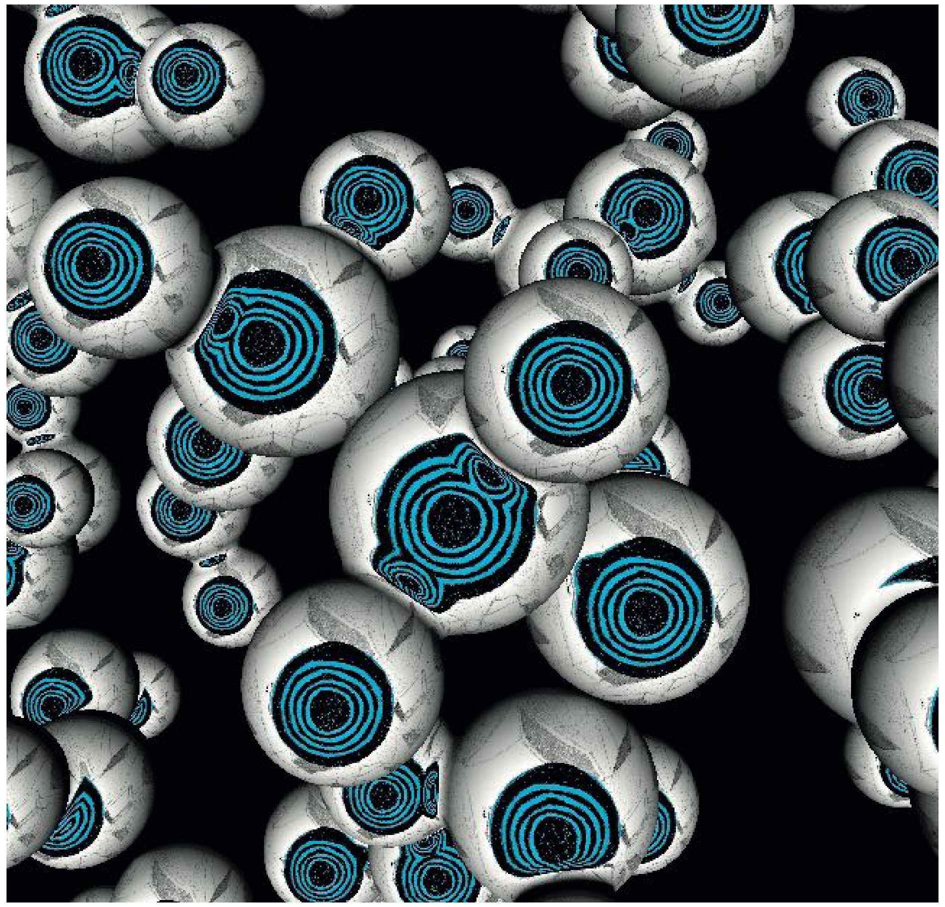
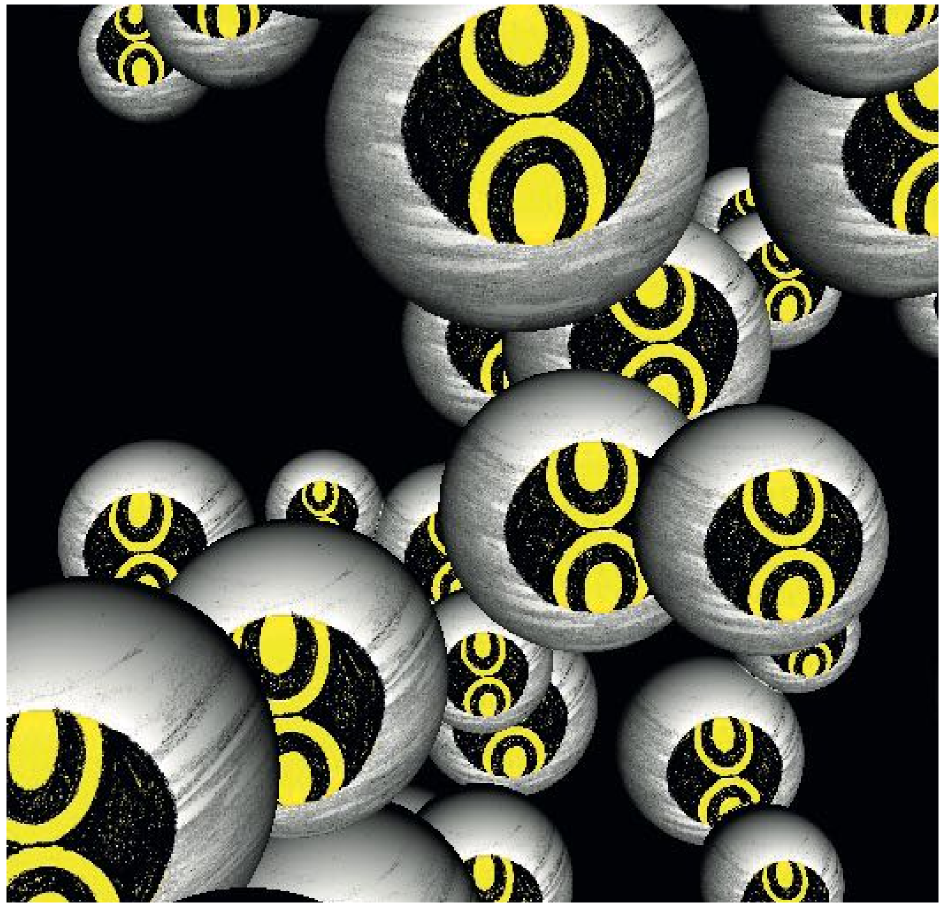
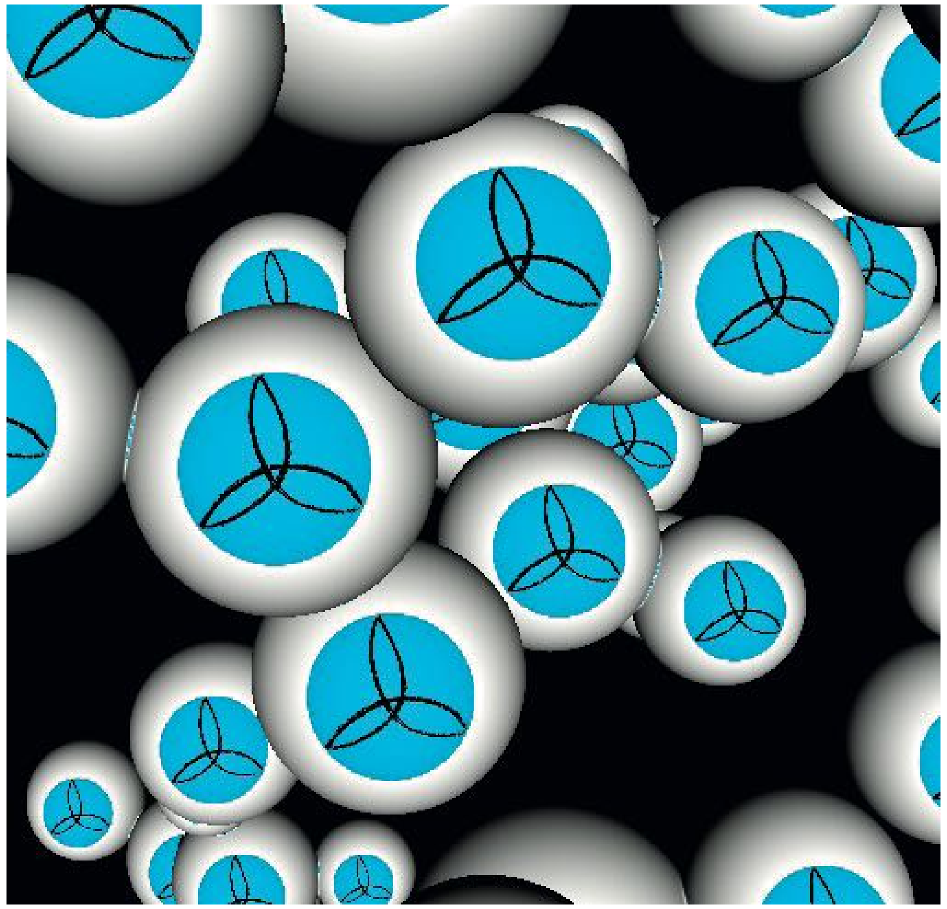
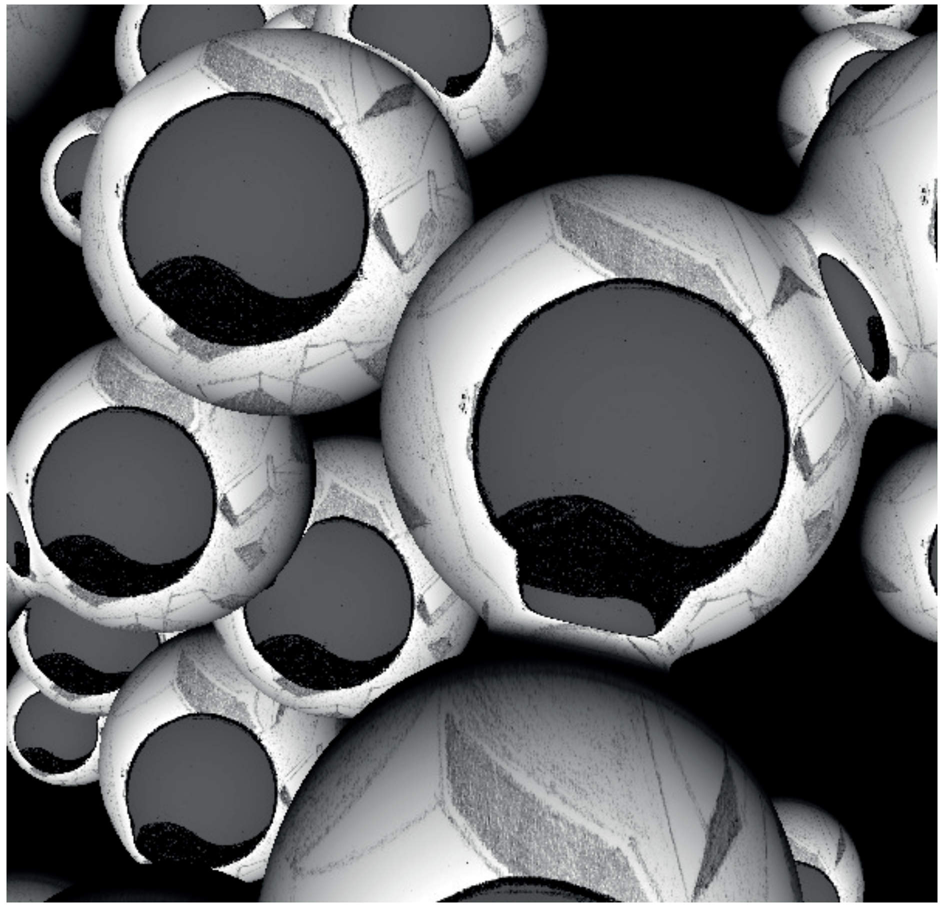
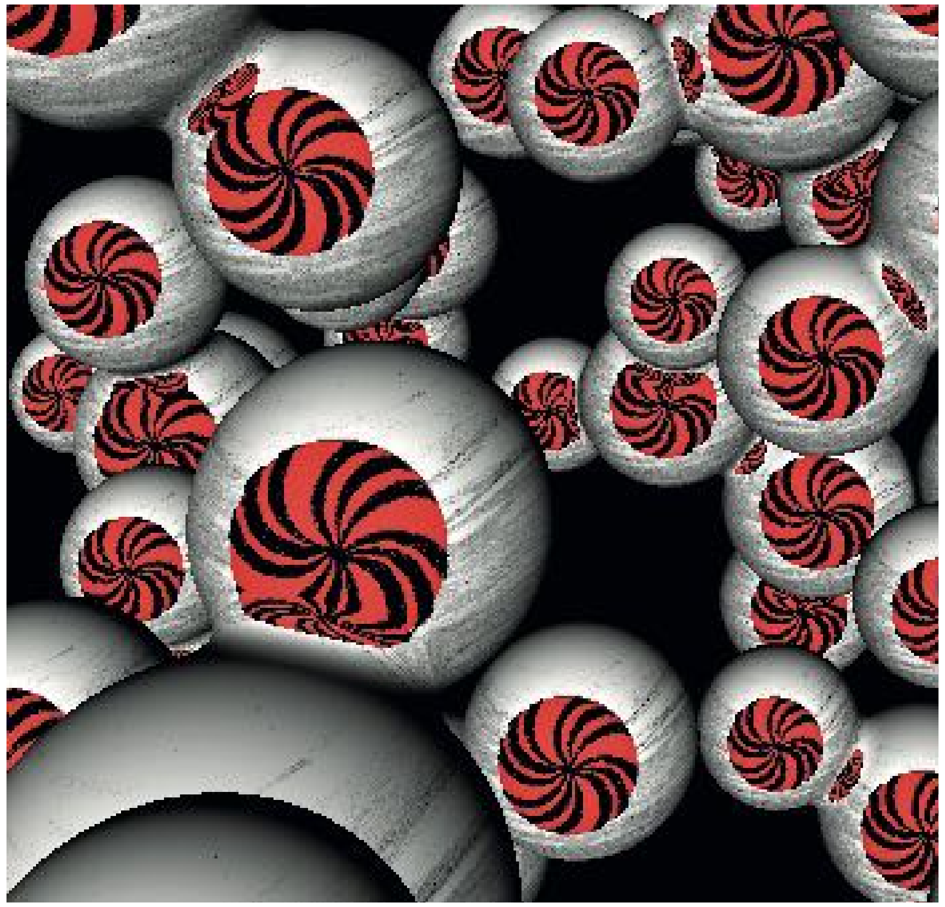

BILAN DE LA PHASE PRÉCÉDENTE

- resserer l'axe graphique
- définir la gamme d'icônes
- insérer les propositions dans le réel

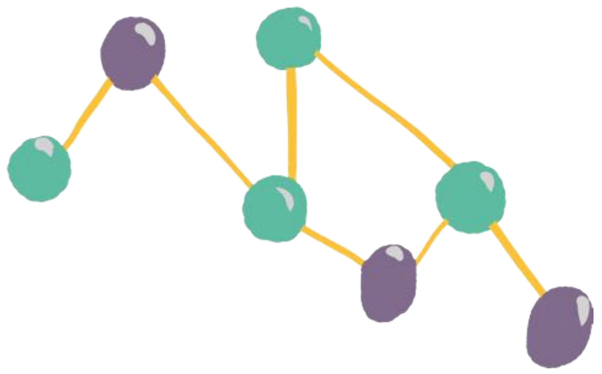

MARGAUX KHALIL

CHOIX D’APPLICATION GRAPHIQUE

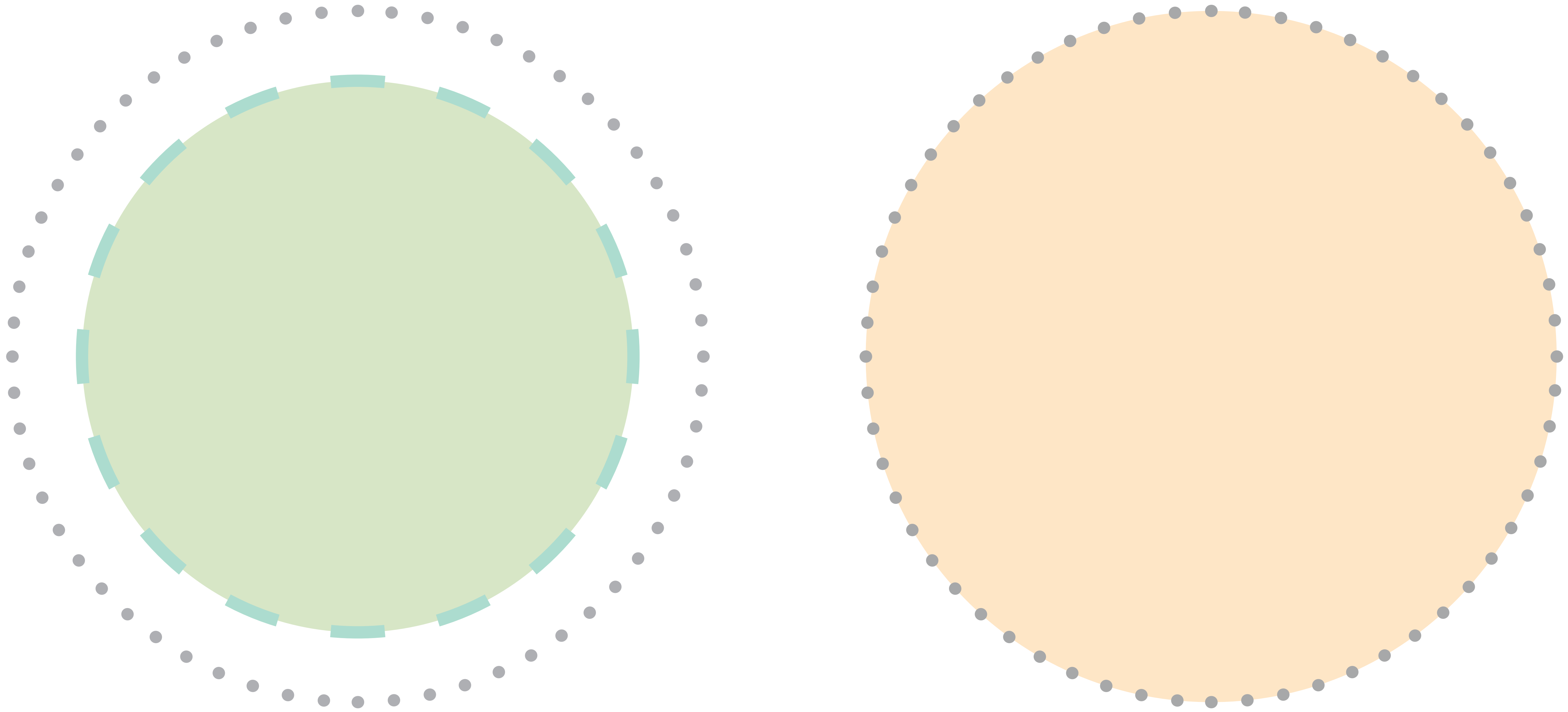

MARGAUX KHALIL

CHOIX D'APPLICATION GRAPHIQUE

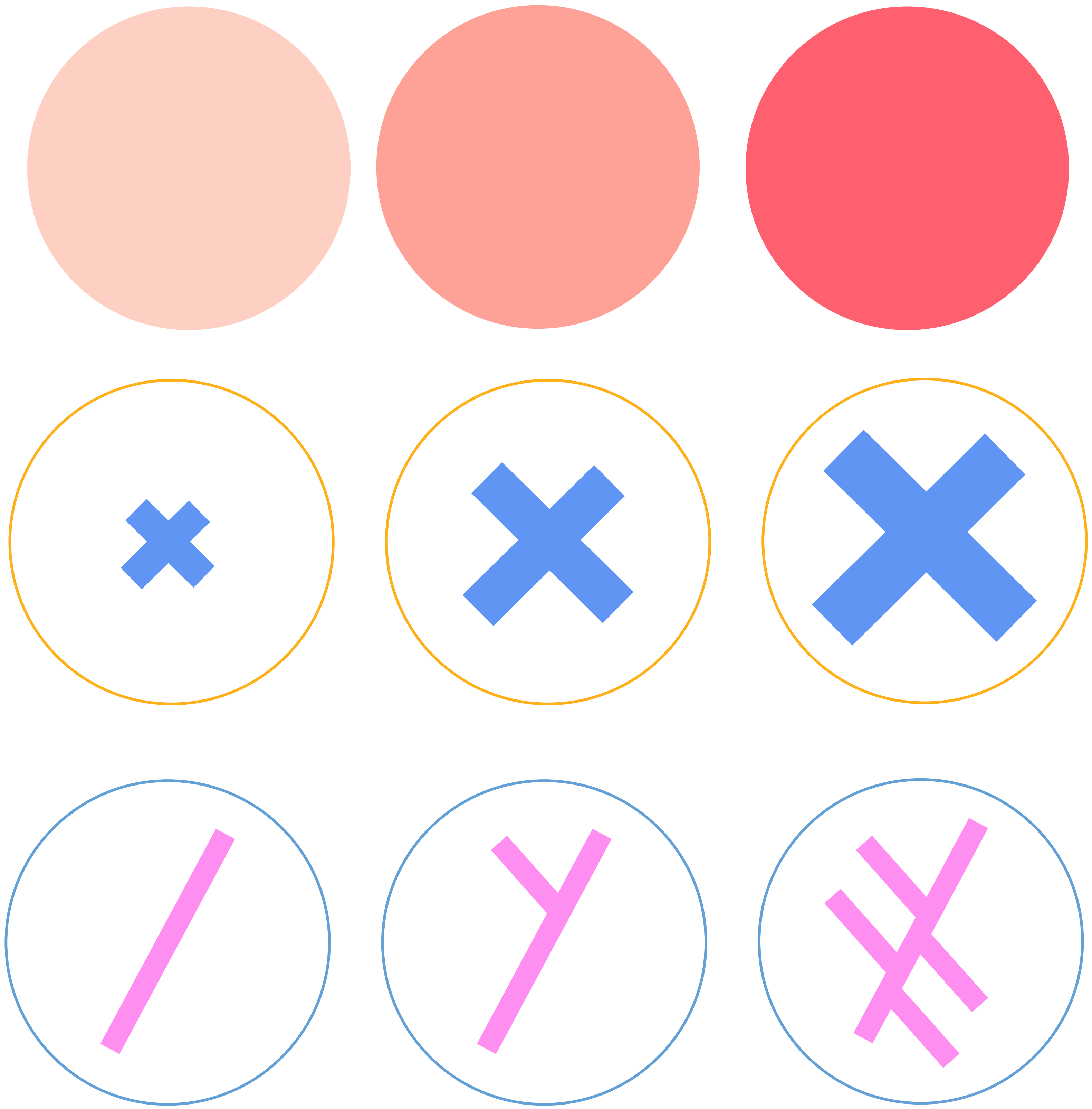

NATURE DE L'ATOME

carbone / carbon

C

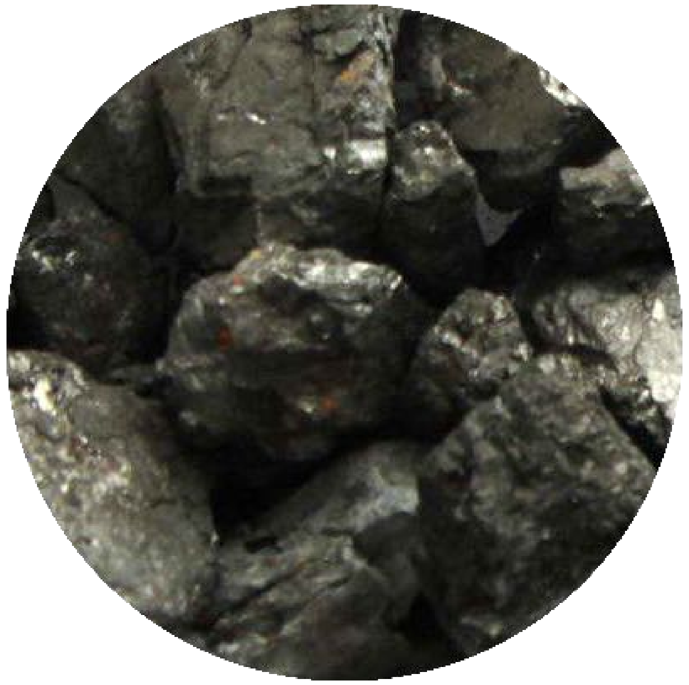

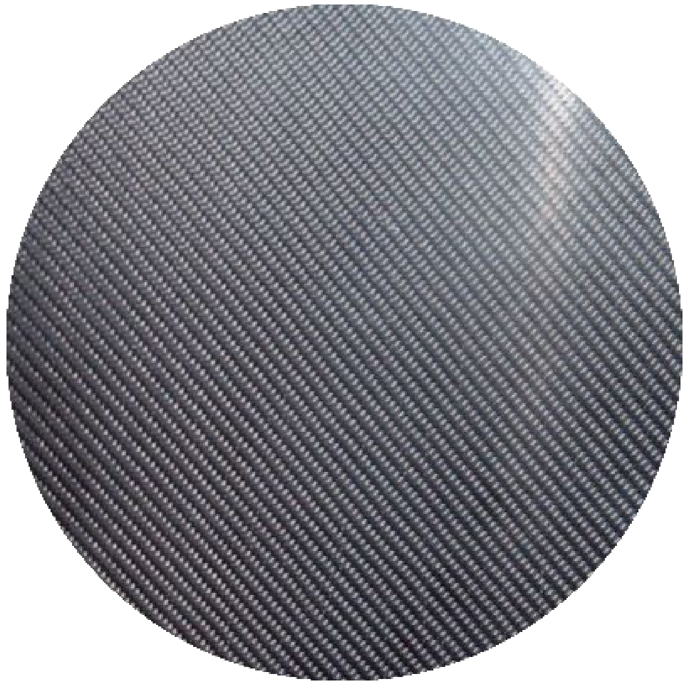

6

S

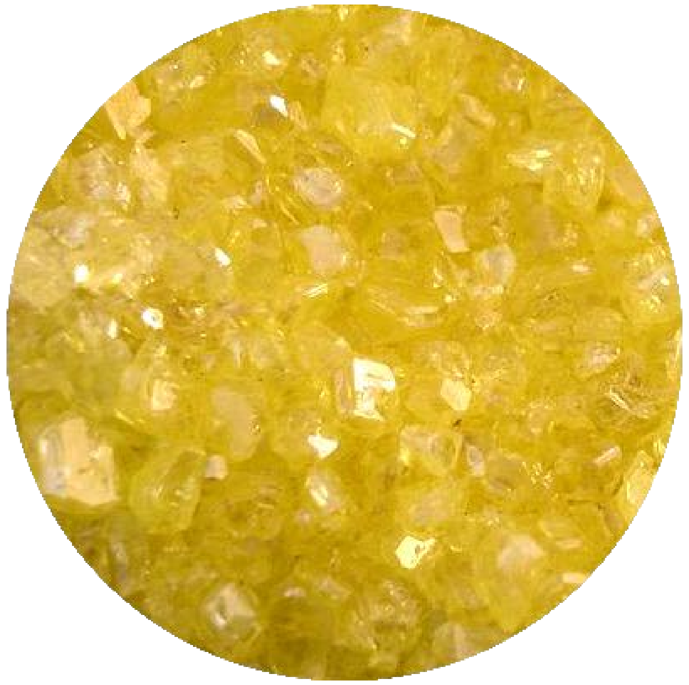

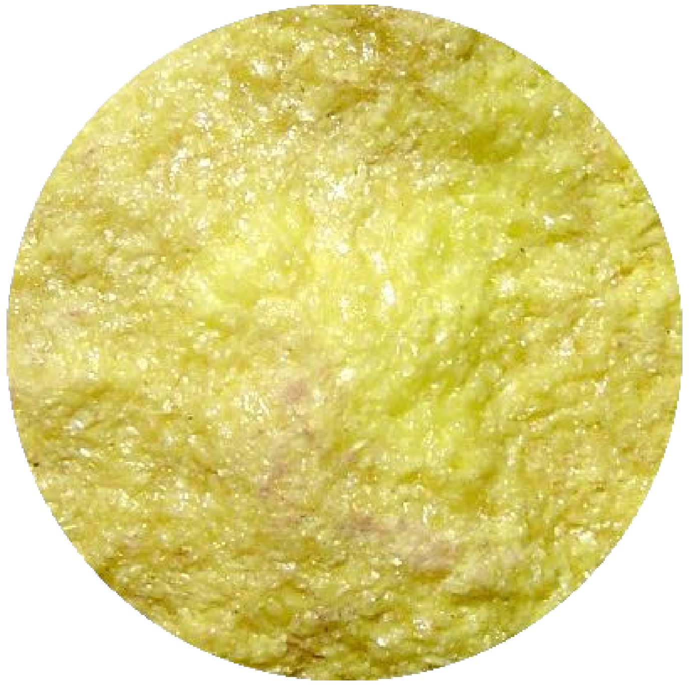

16

soufre / sulfur

MARGAUX KHALIL

NATURE DE L'ATOME

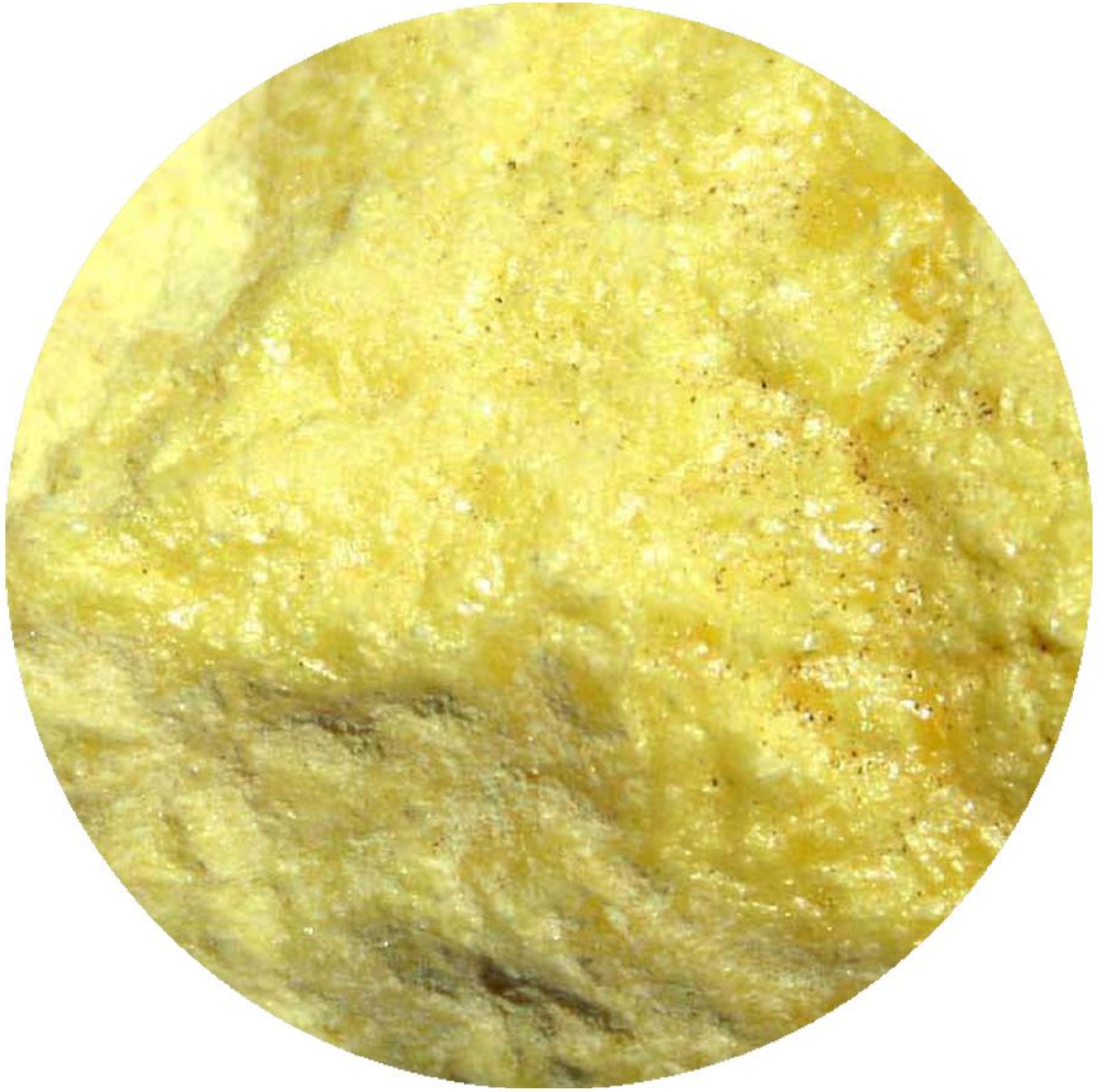

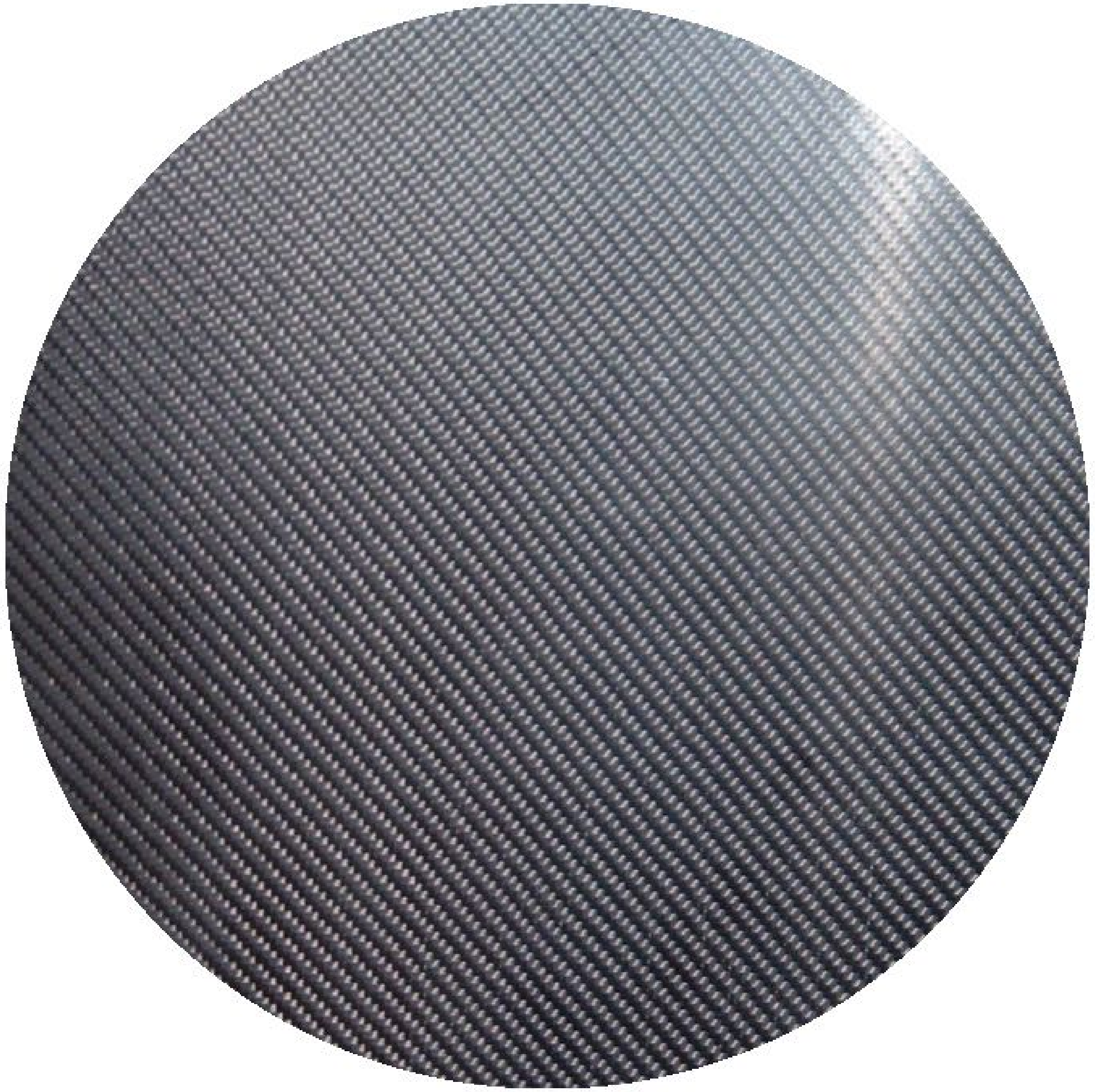

MARGAUX KHALIL

NATURE DE L'ATOME

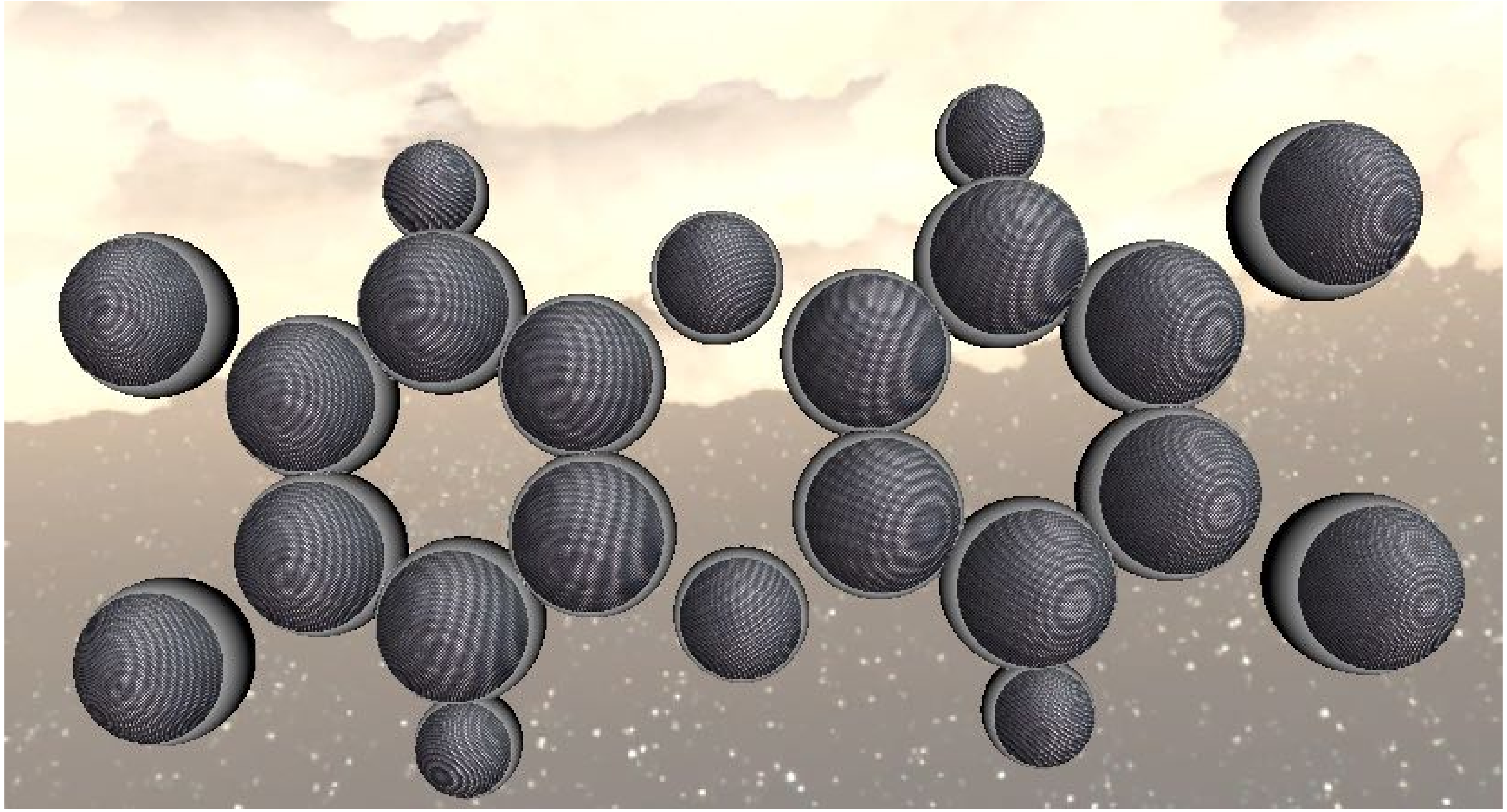

MARGAUX KHALIL

NATURE DE L'ATOME

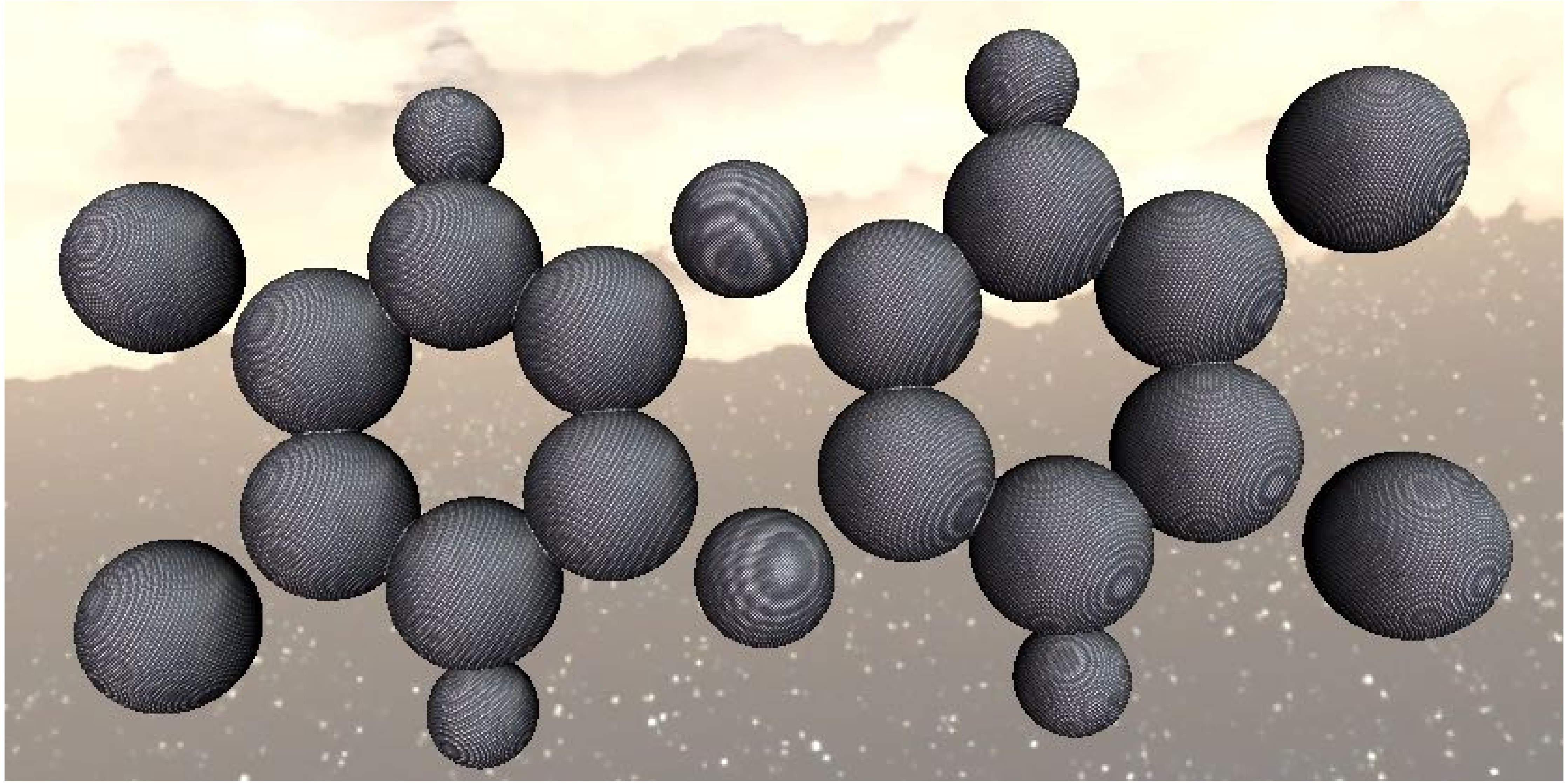

MARGAUX KHALIL

NATURE DE L’ATOME

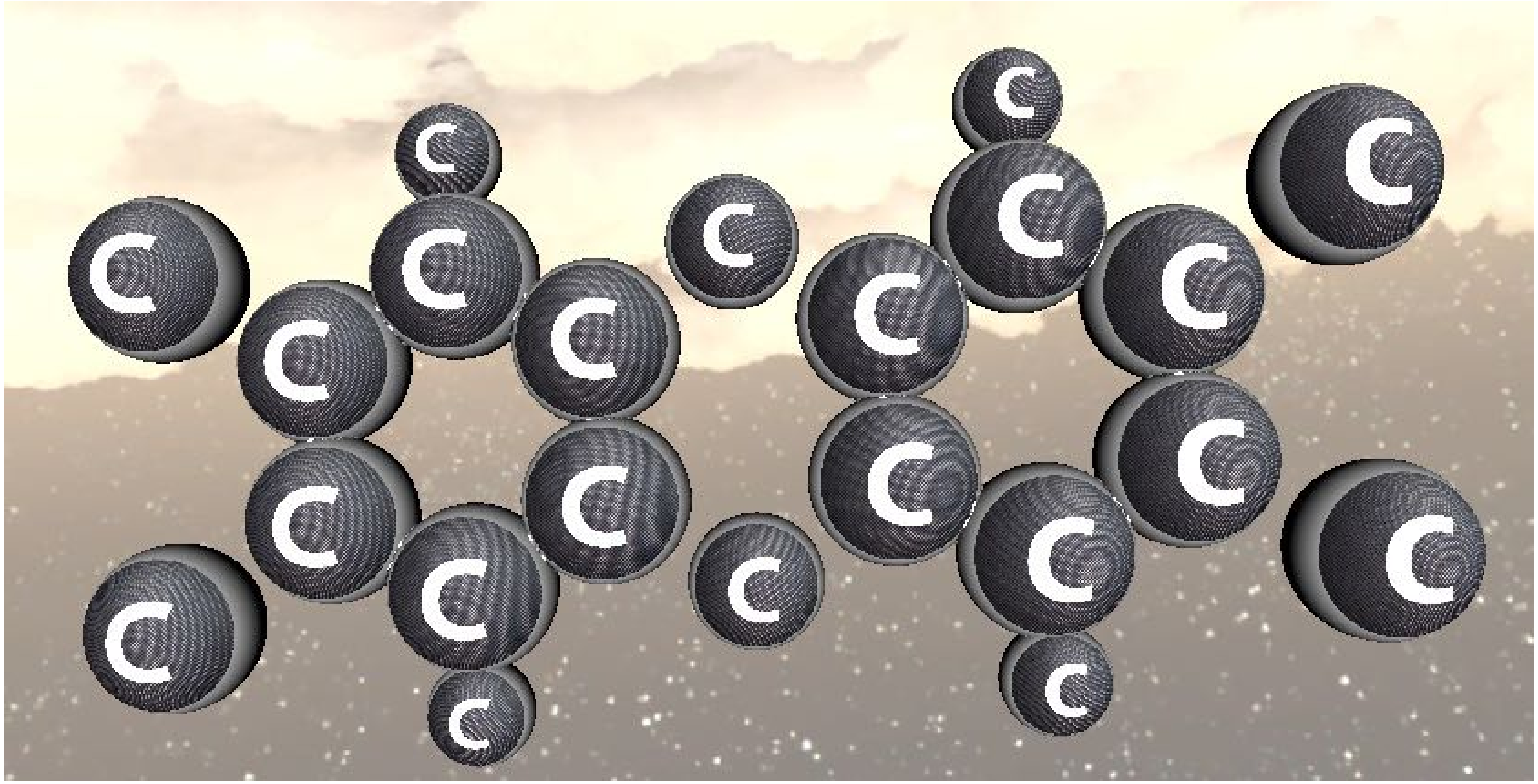

MARGAUX KHALIL

NATURE DE L'ATOME

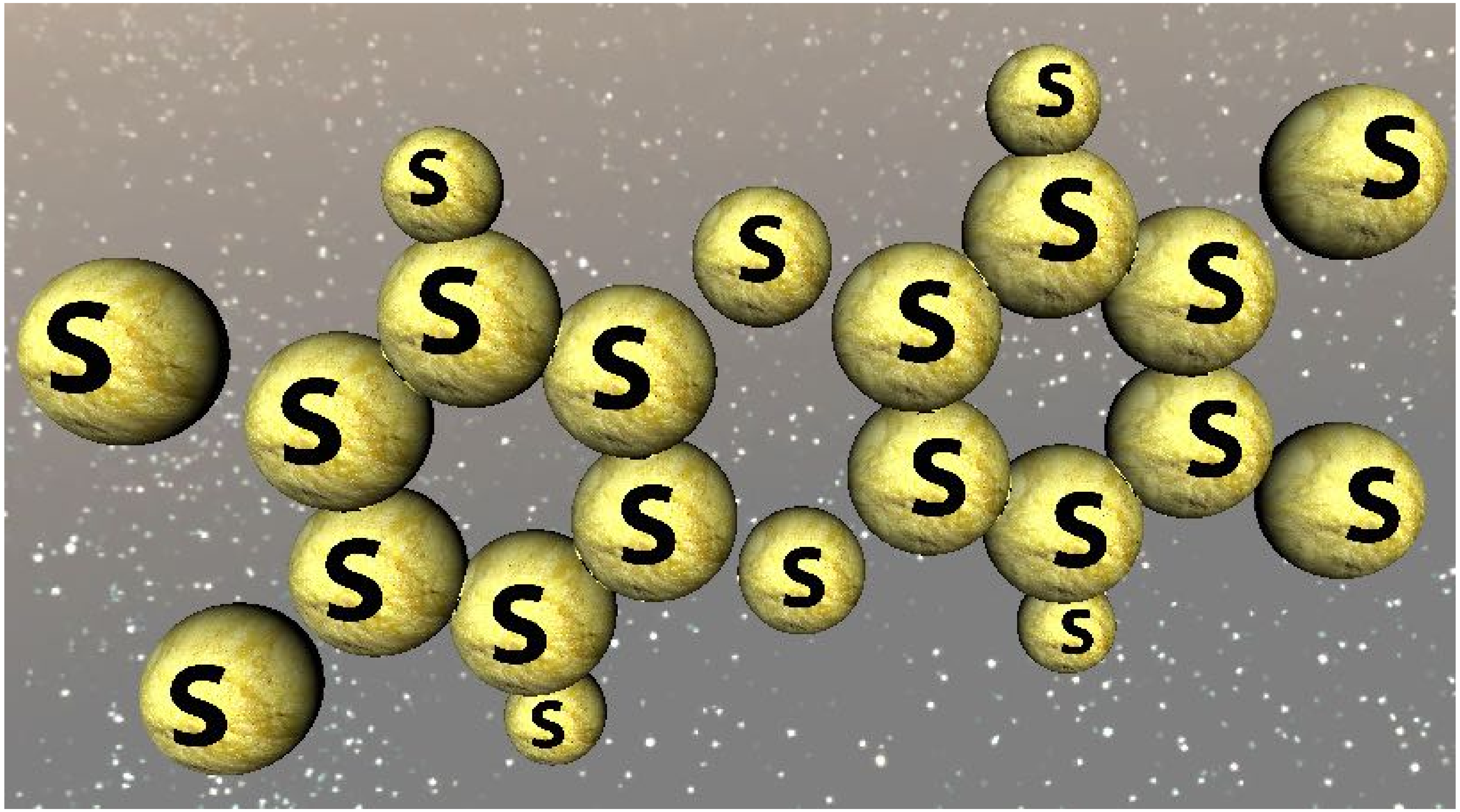

NATURE DE LA MOLÉCULE

soluté

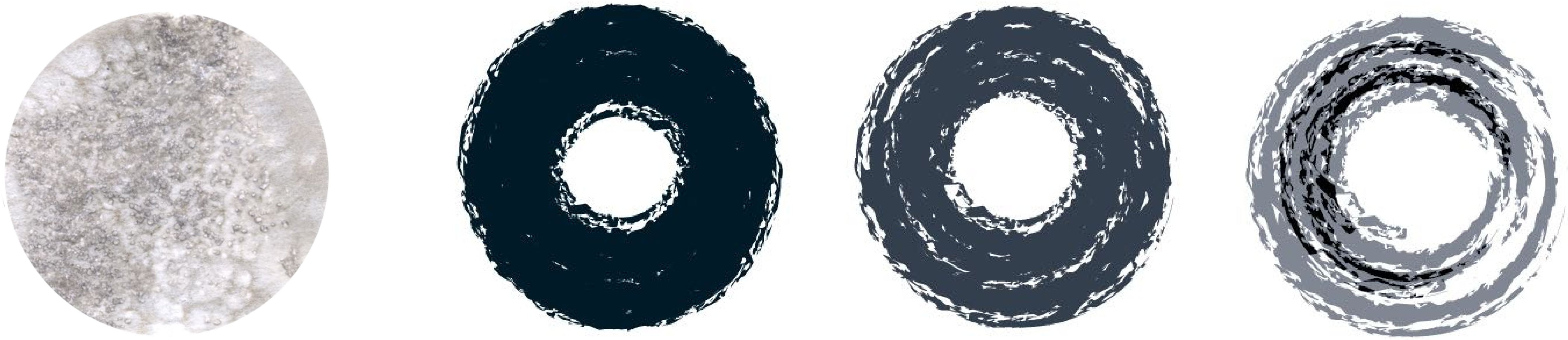

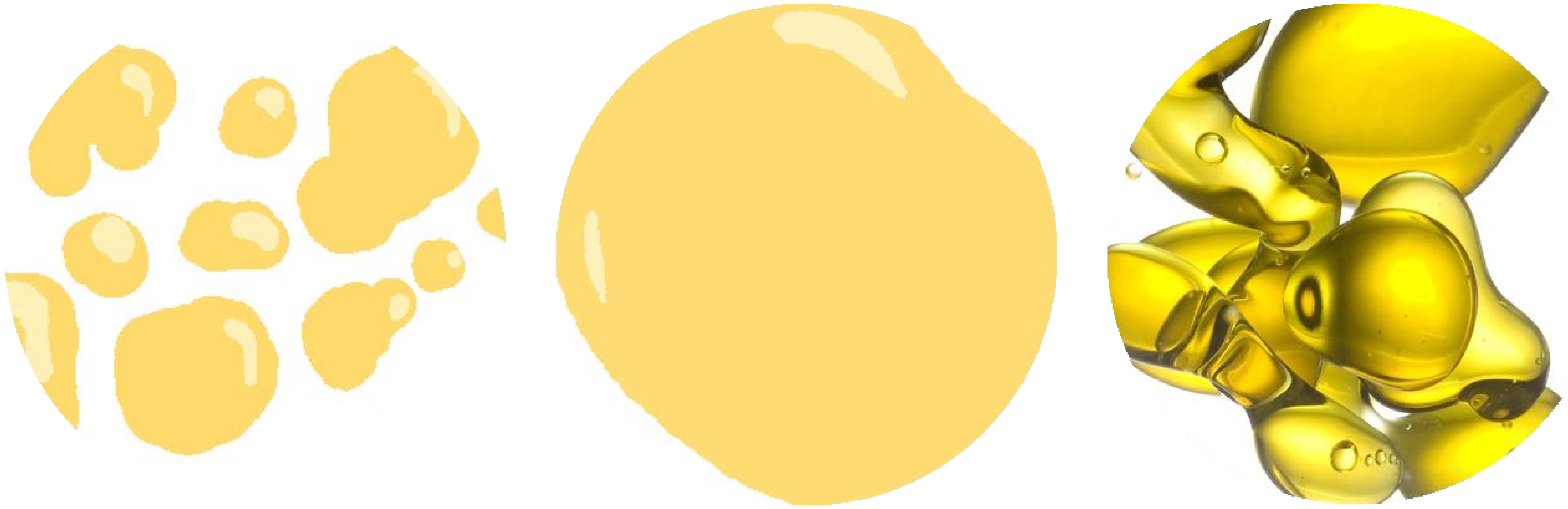

graisse

MARGAUX KHALIL

NATURE DE LA MOLÉCULE

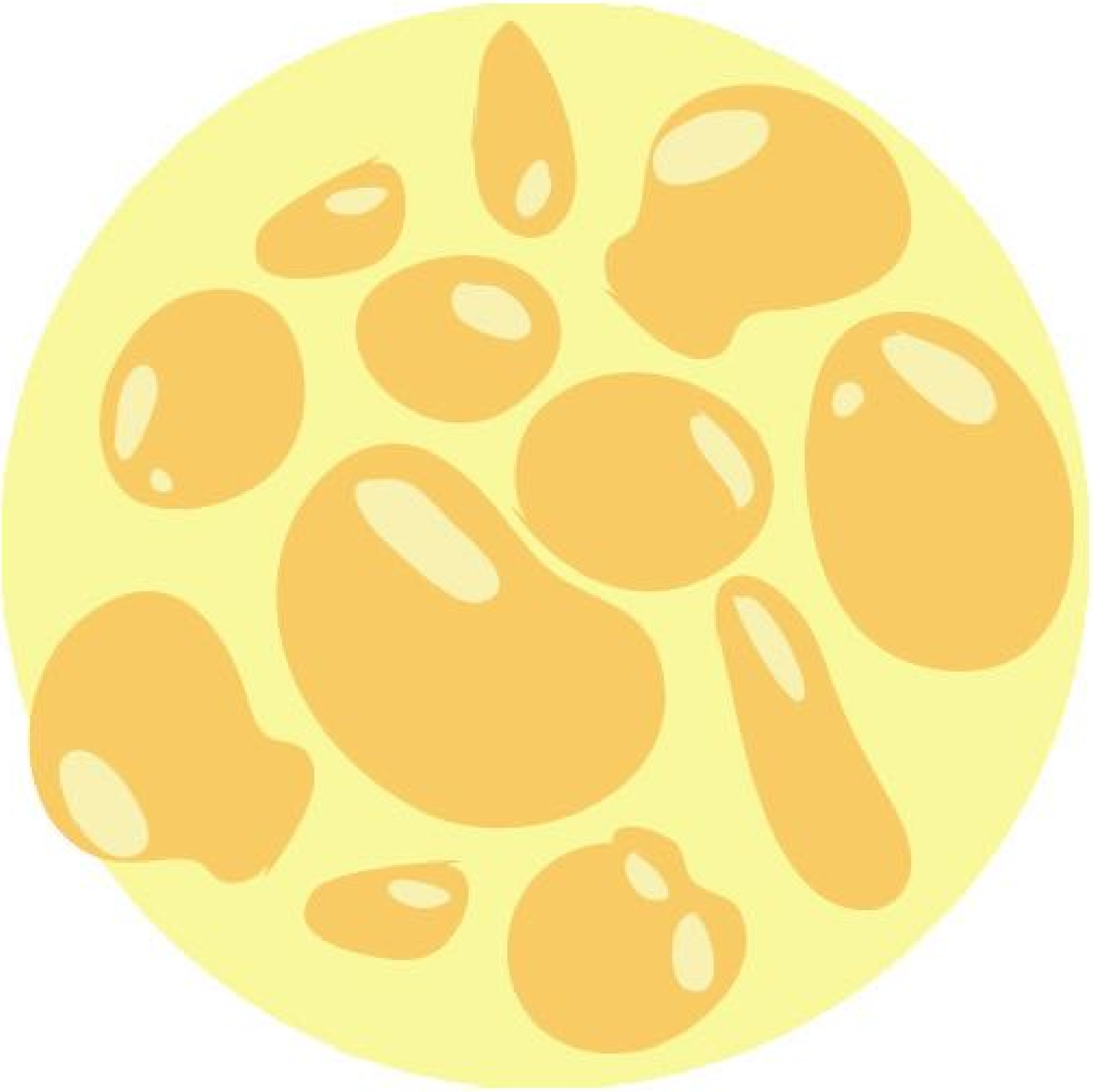

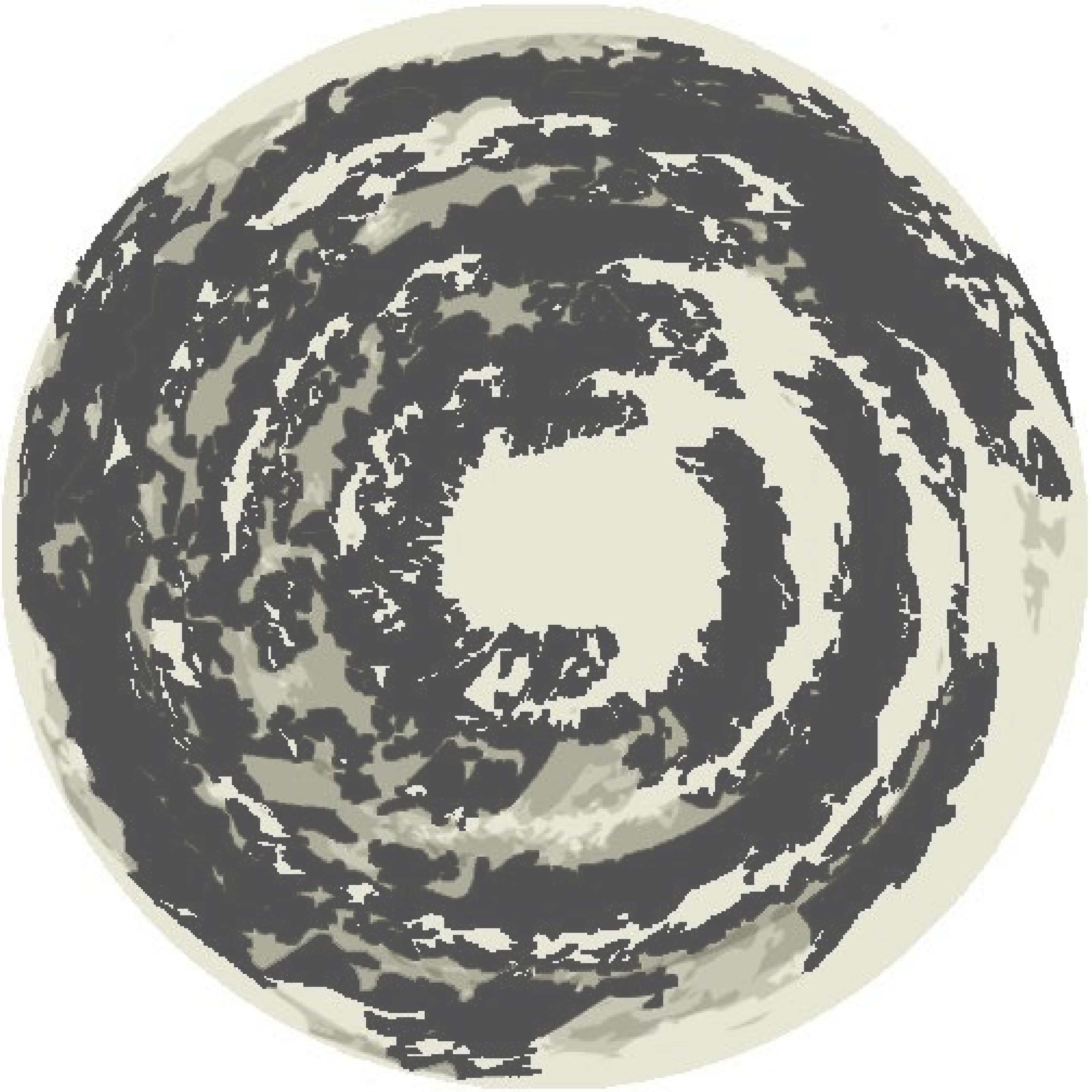

MARGAUX KHALIL

NATURE DE LA MOLÉCULE

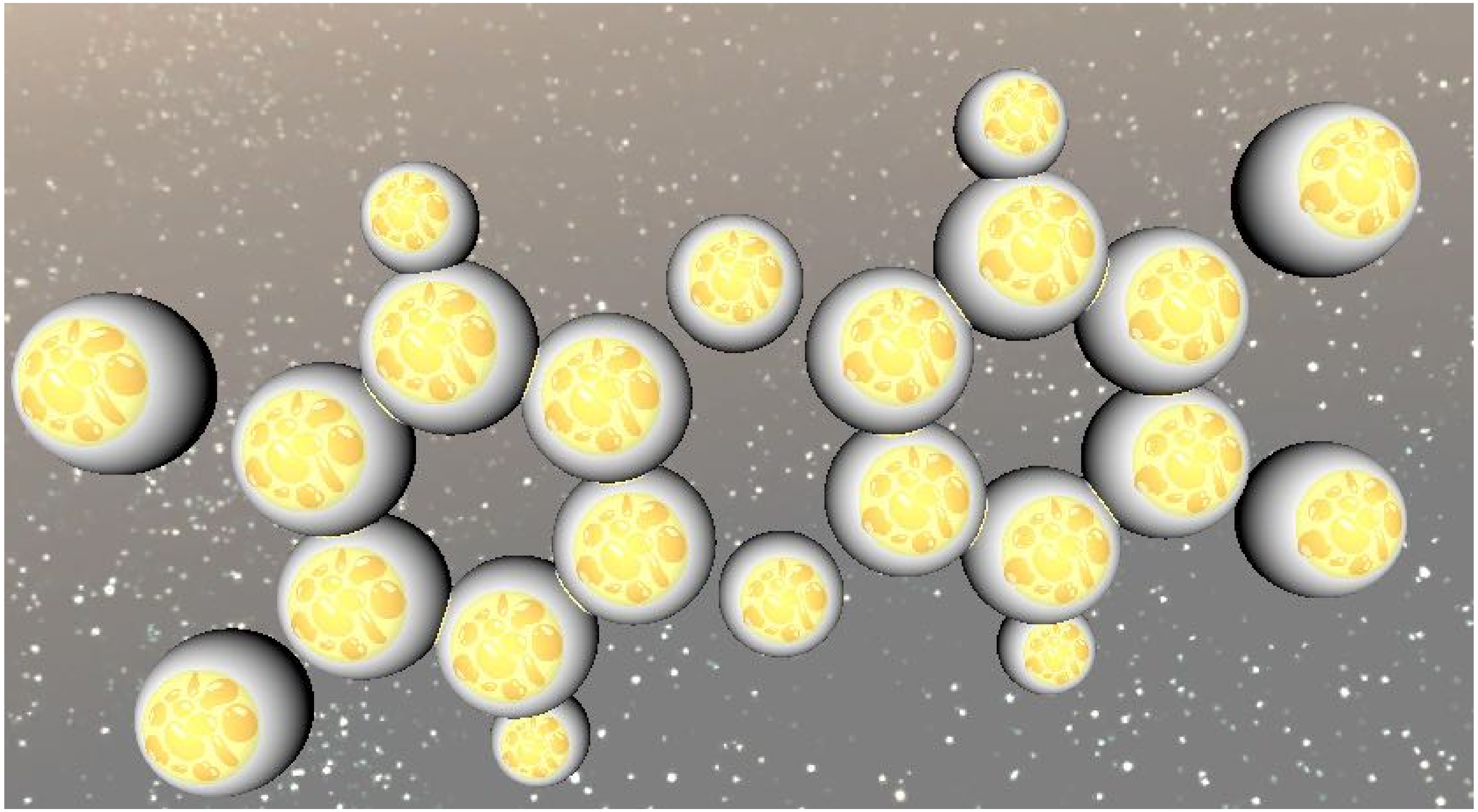

MARGAUX KHALIL

NATURE DE LA MOLÉCULE

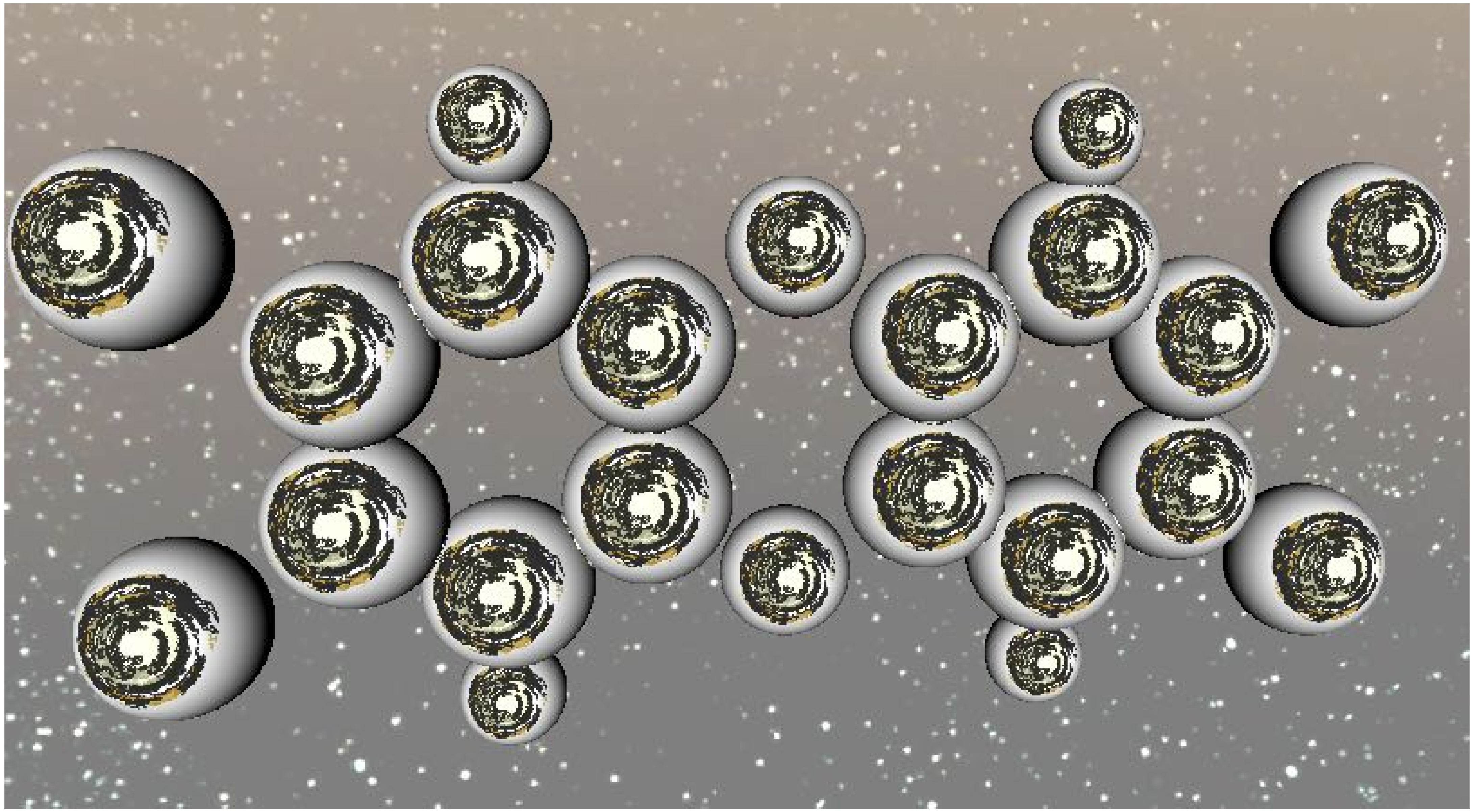

MARGAUX KHALIL

HYDROPHILIE / HYDROPHOBIE

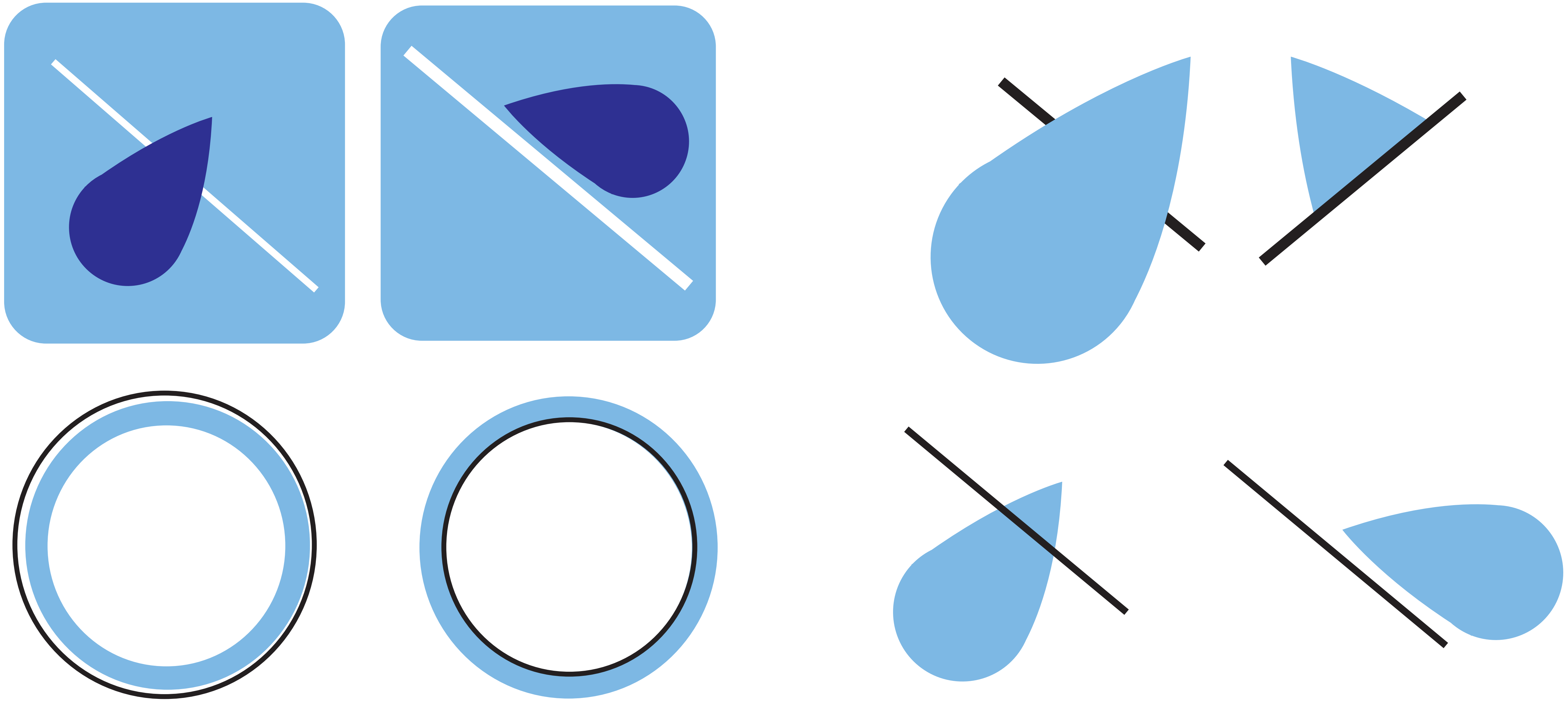

MARGAUX KHALIL

HYDROPHILIE / HYDROPHOBIE

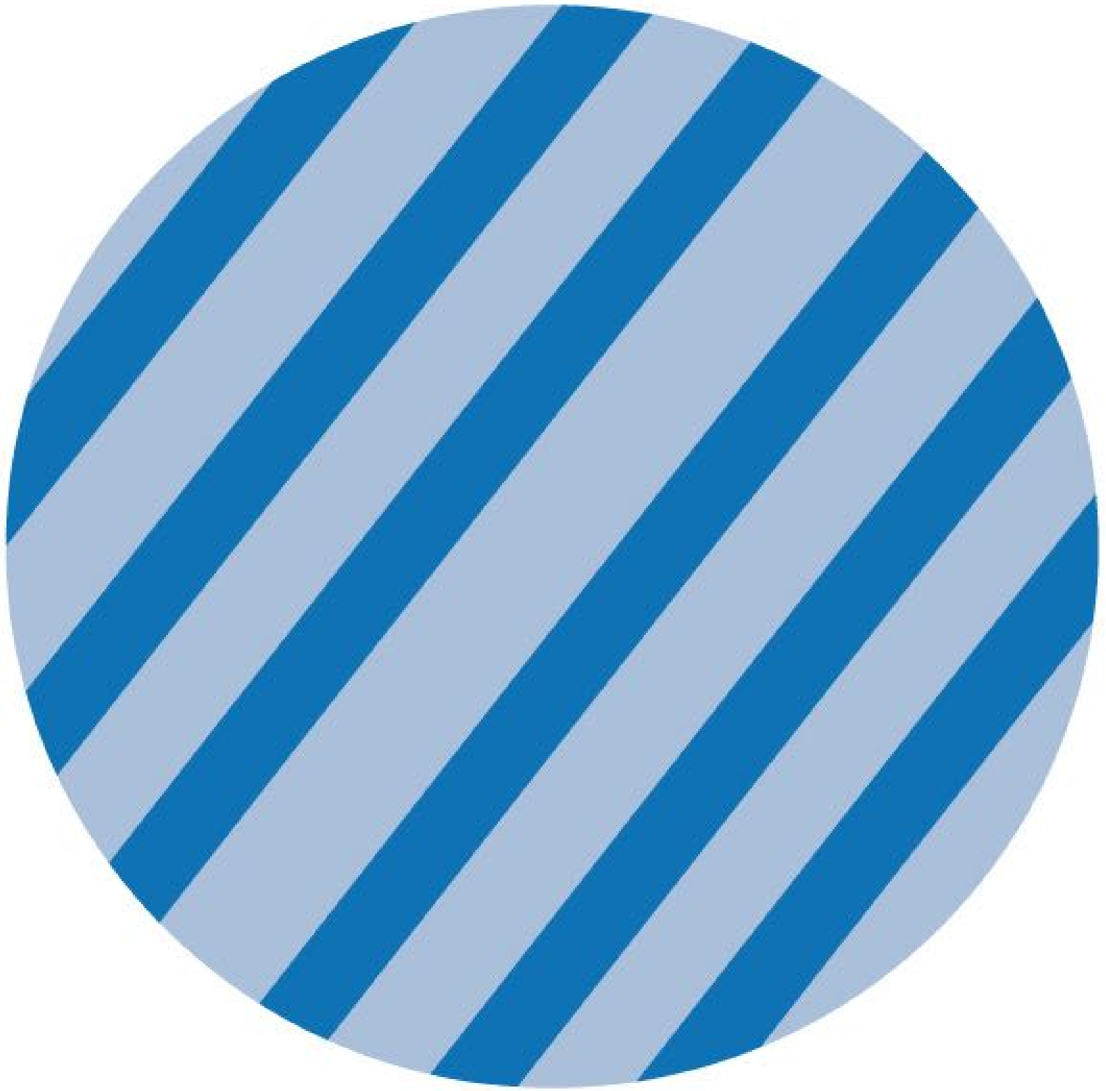

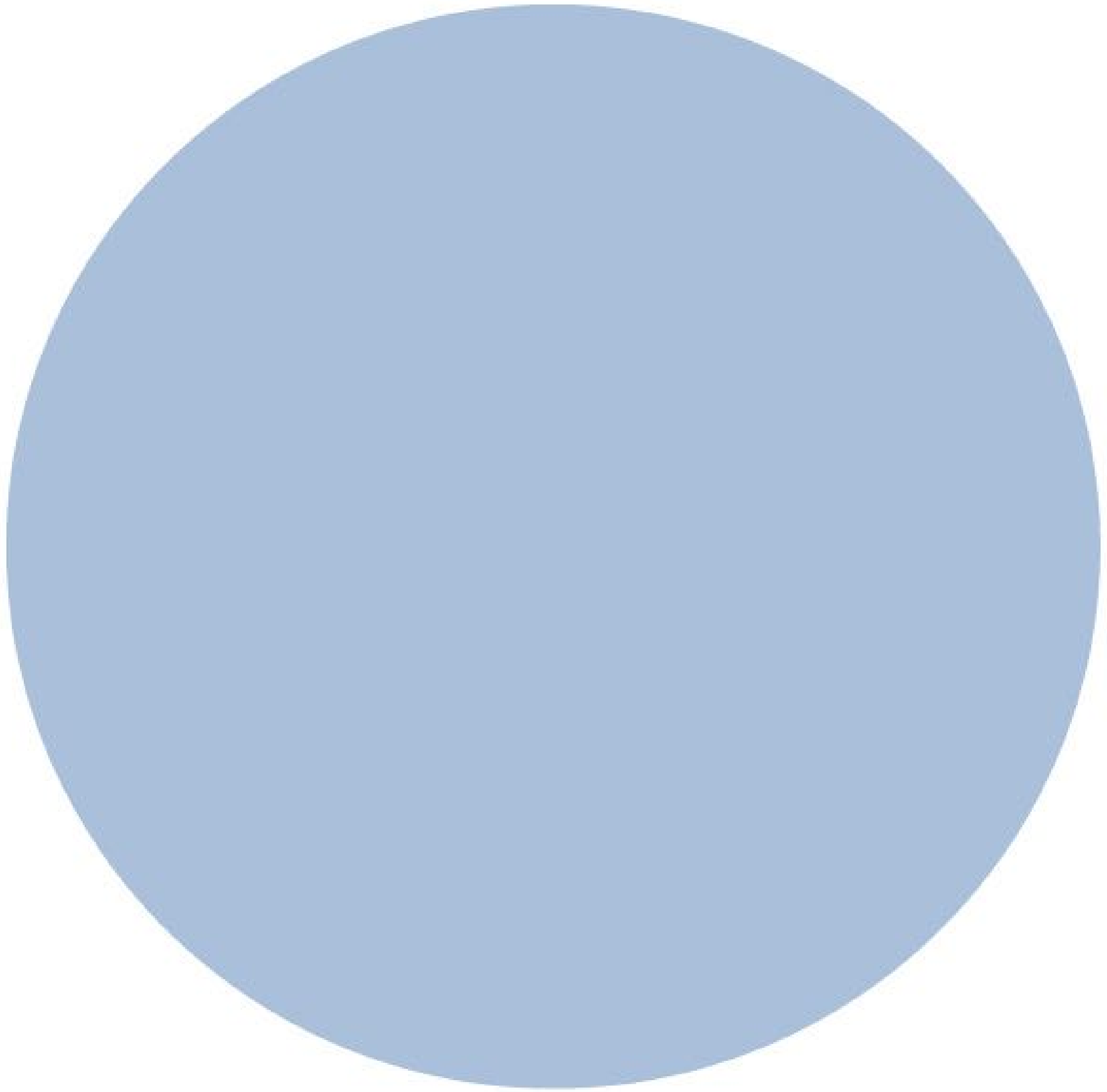

MARGAUX KHALIL

HYDROPHILIE / HYDROPHOBIE

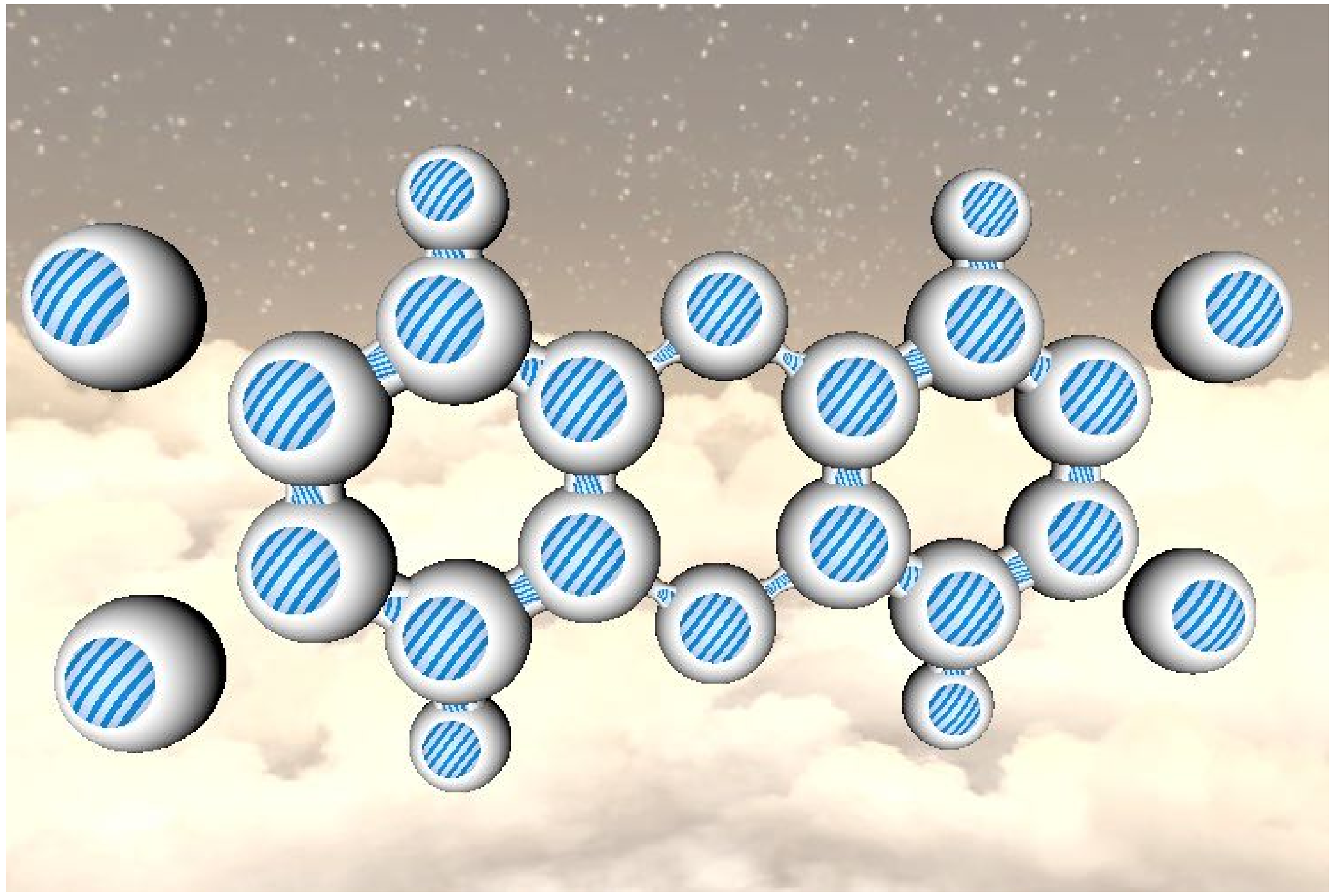

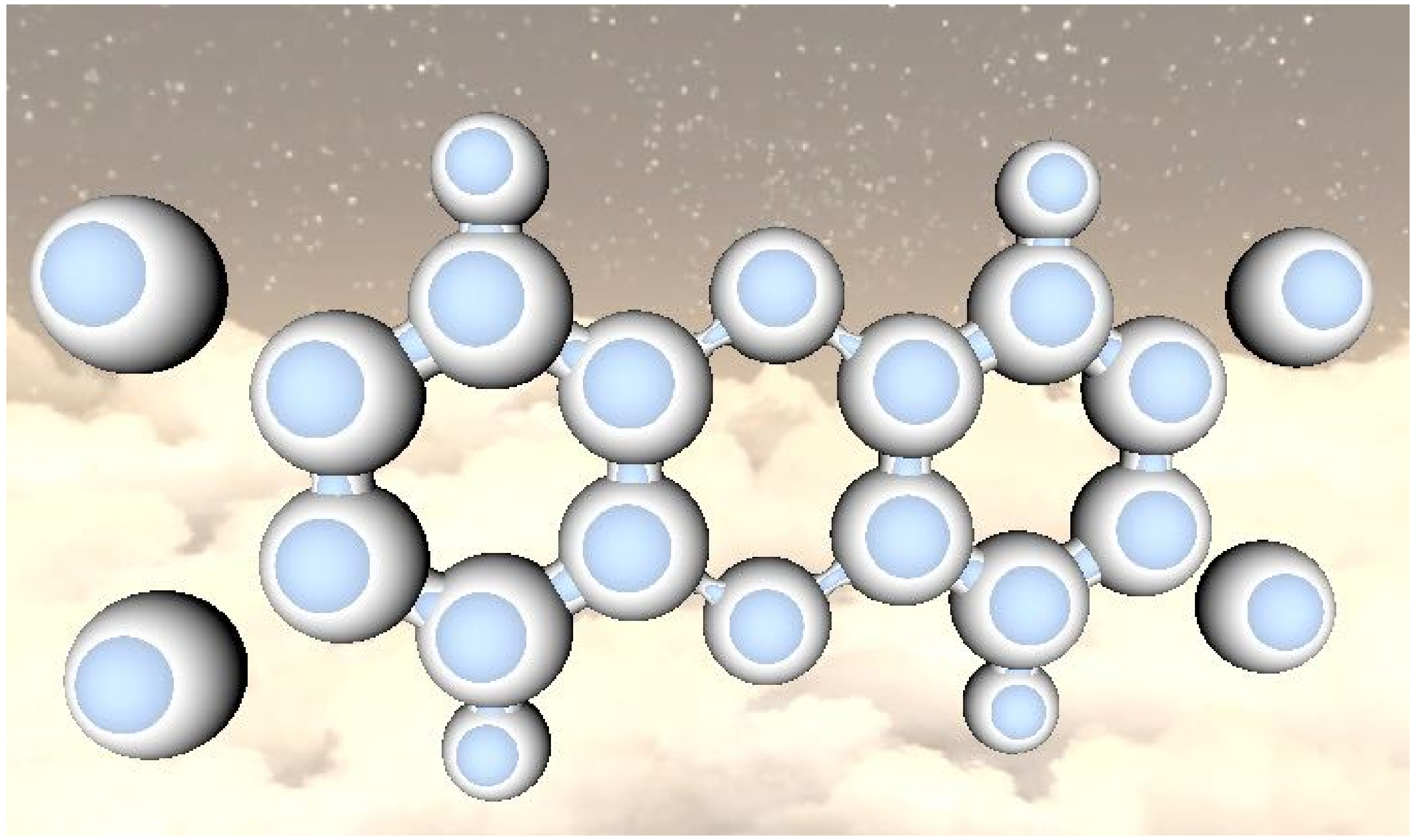

MARGAUX KHALIL

HYDROPHILIE / HYDROPHOBIE

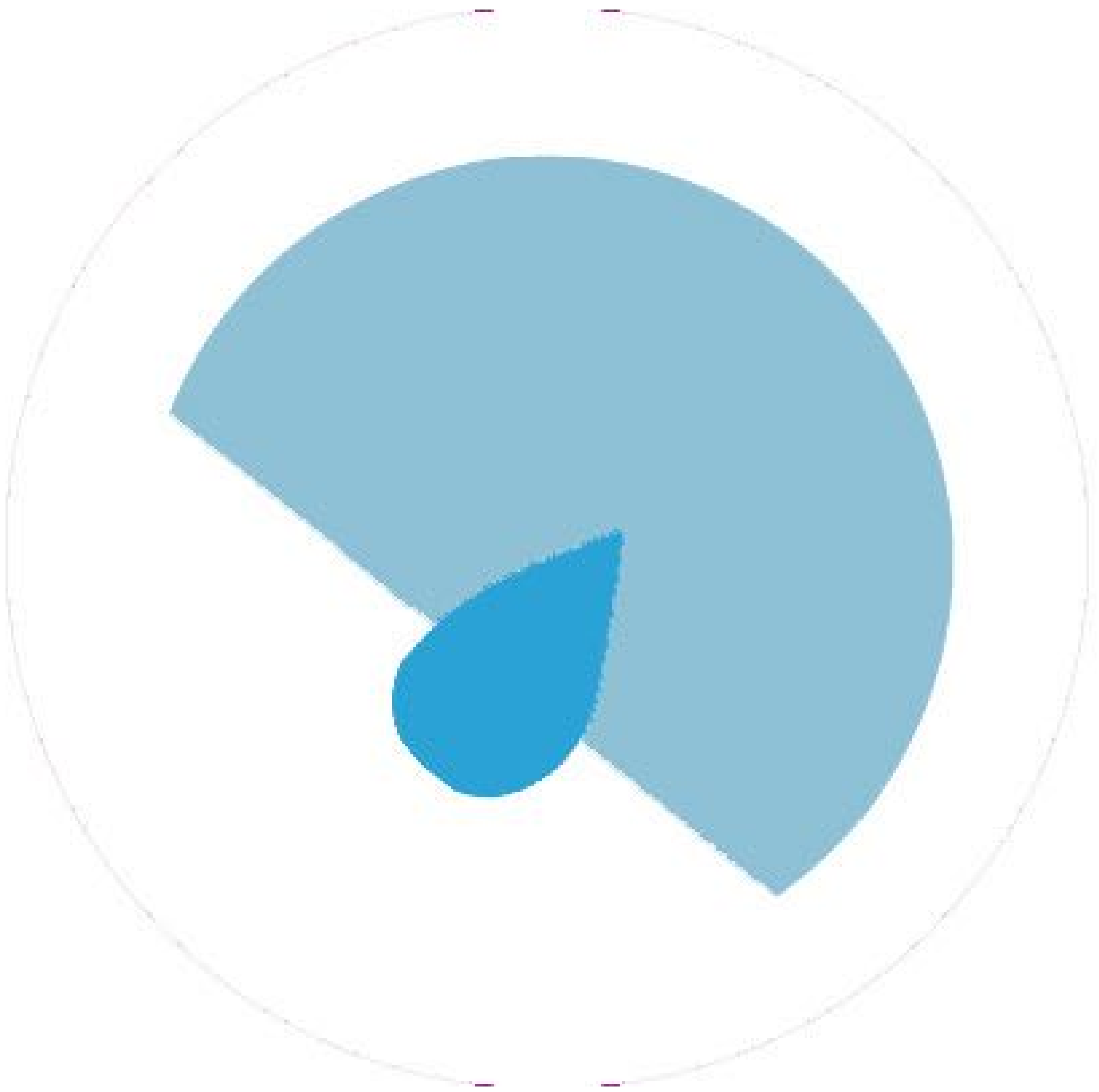

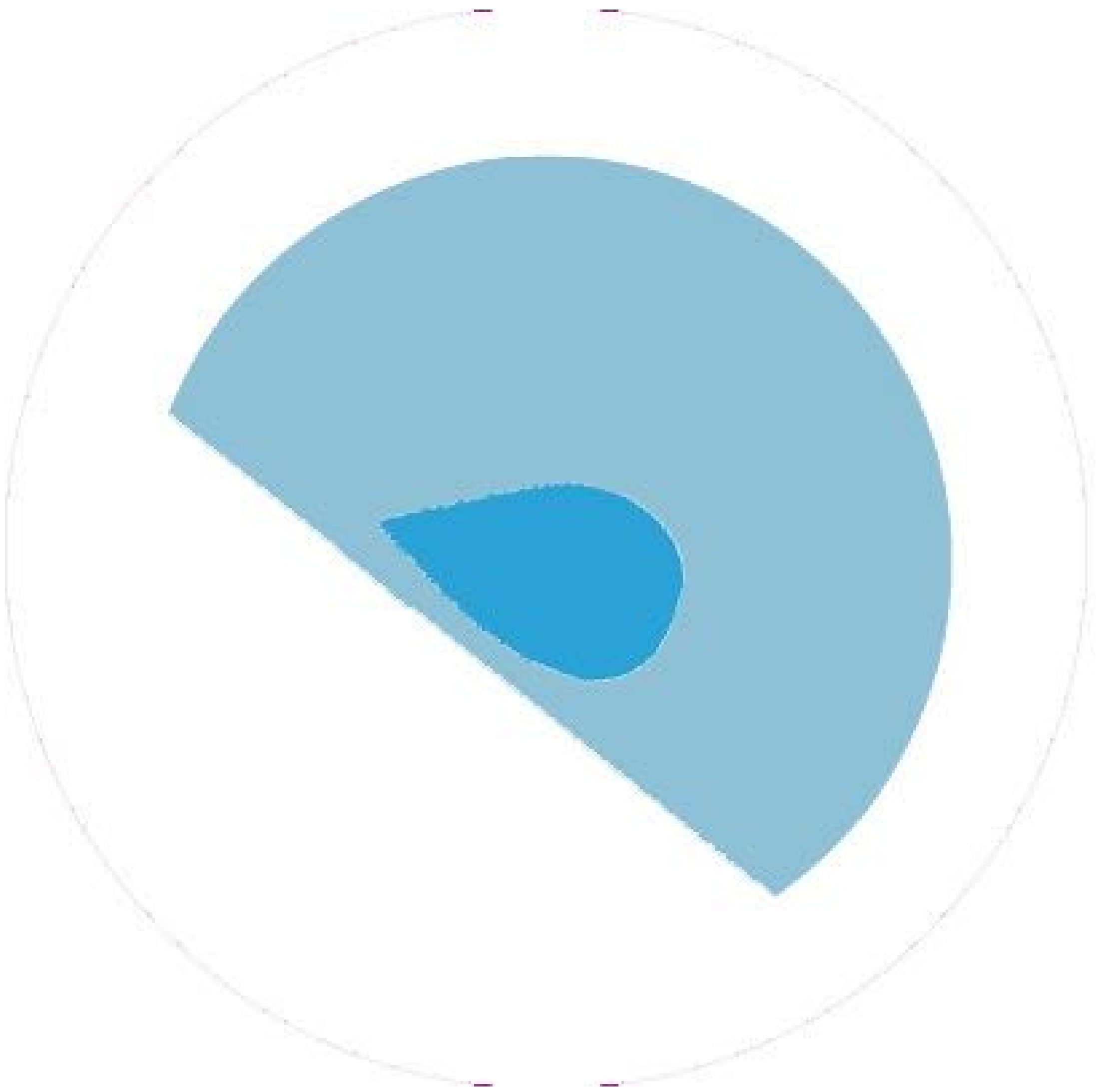

MARGAUX KHALIL

HYDROPHILIE / HYDROPHOBIE

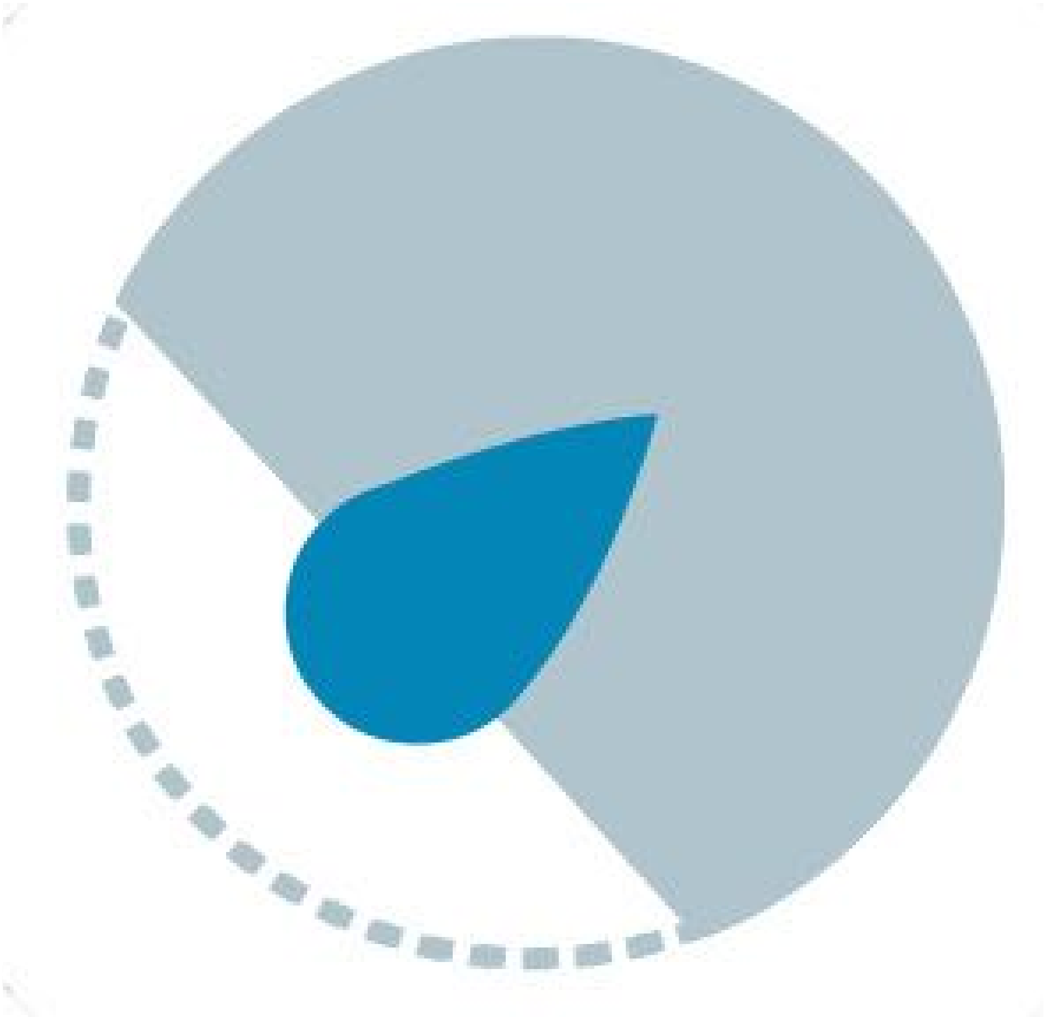

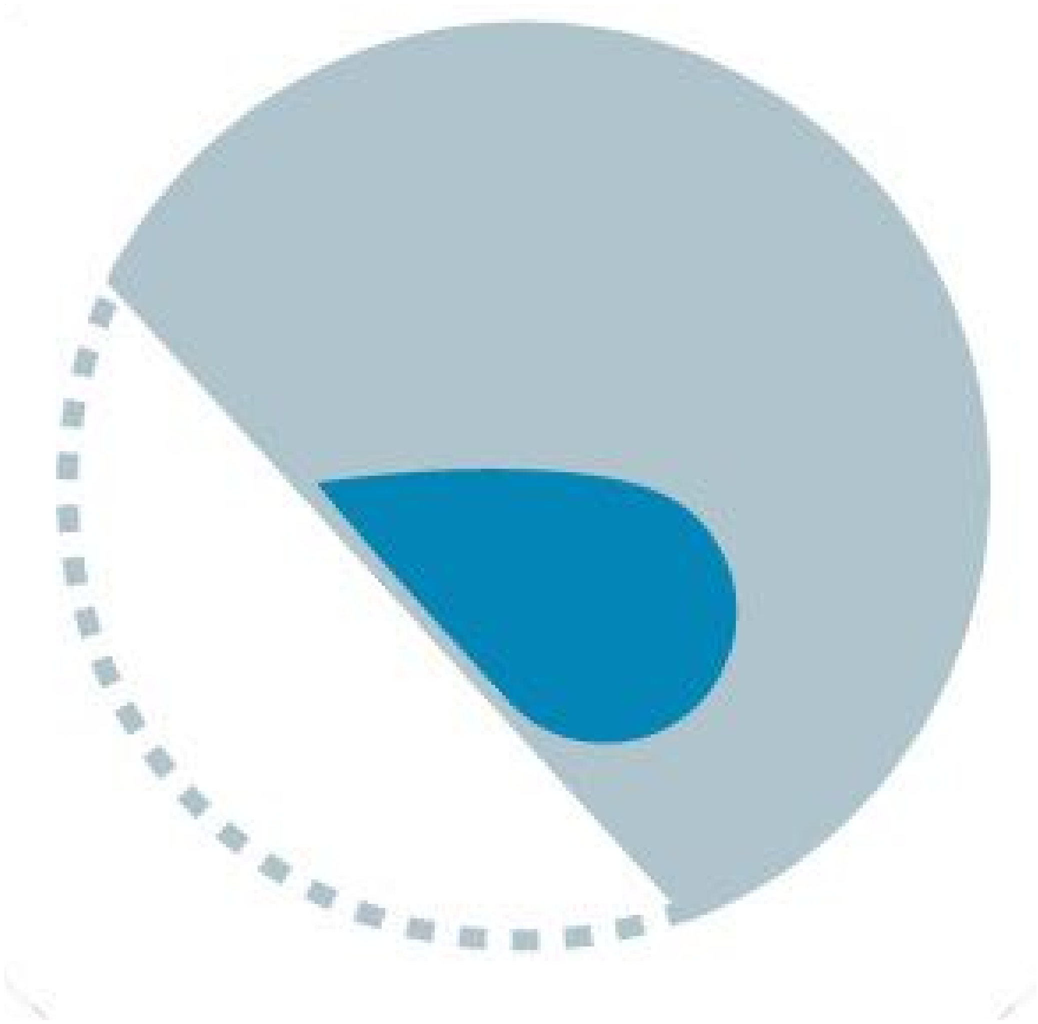

MARGAUX KHALIL

HYDROPHILIE / HYDROPHOBIE

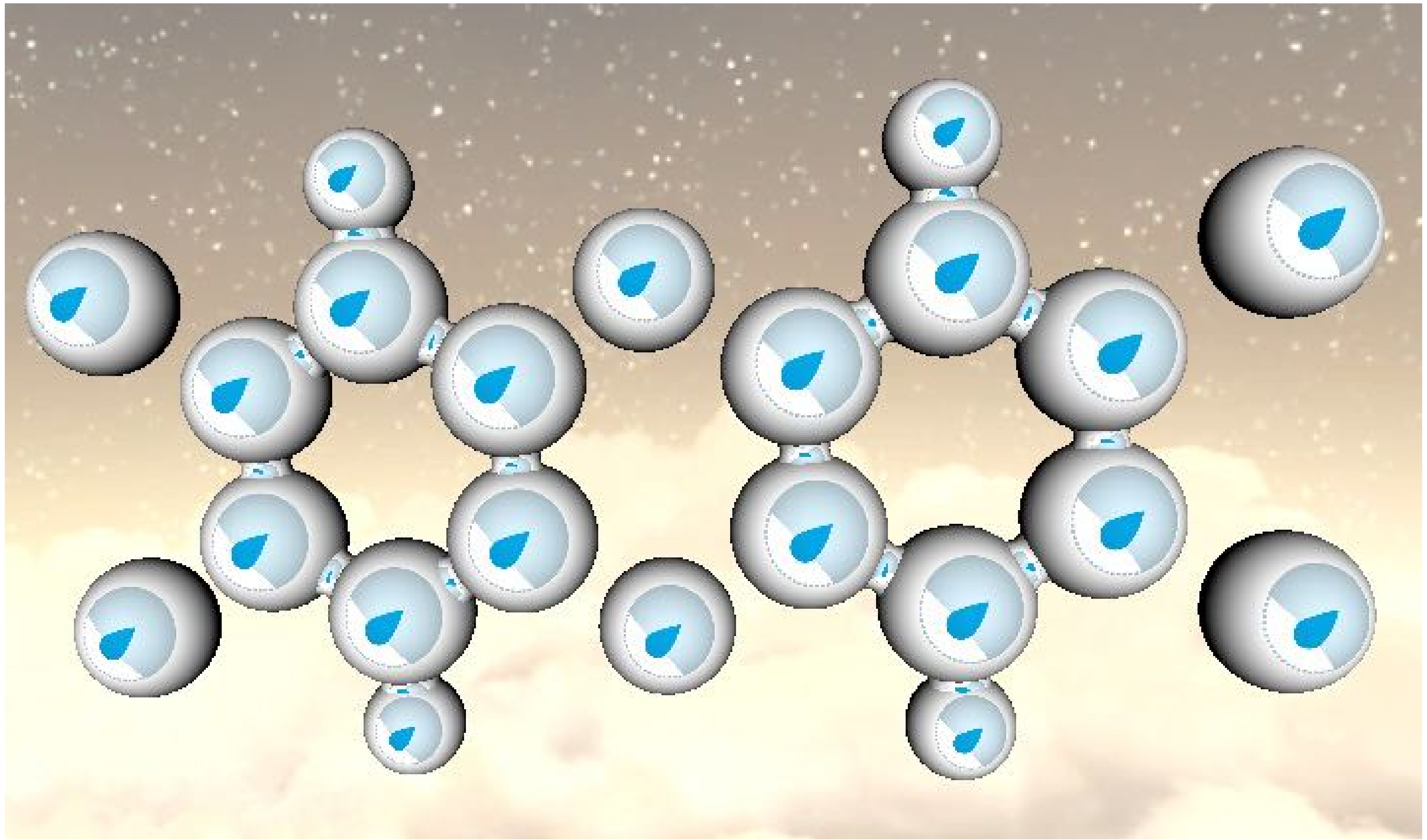

MARGAUX KHALIL

HYDROPHILIE / HYDROPHOBIE

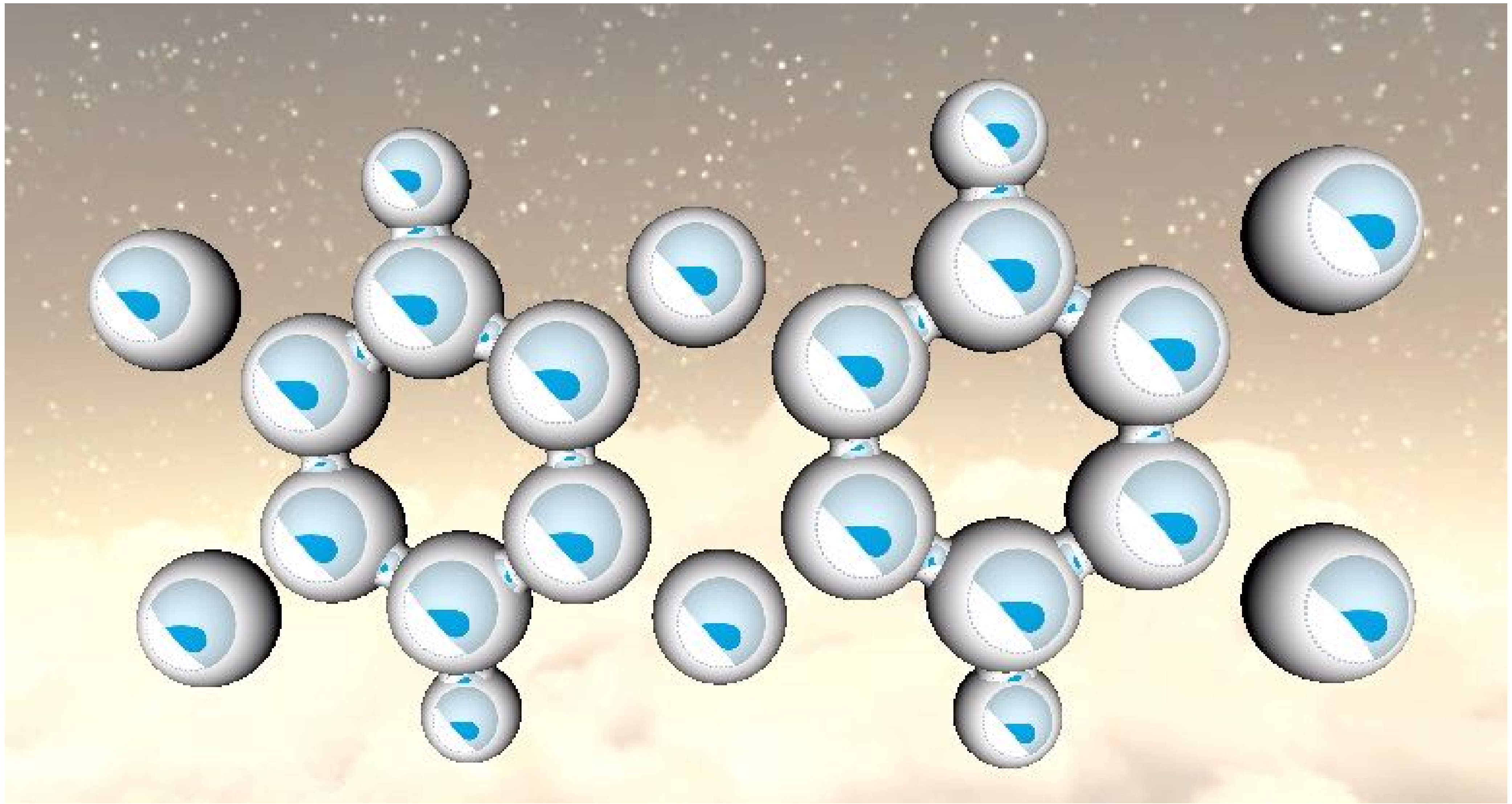

MARGAUX KHALIL

ÉCOCOMPATIBILITÉ

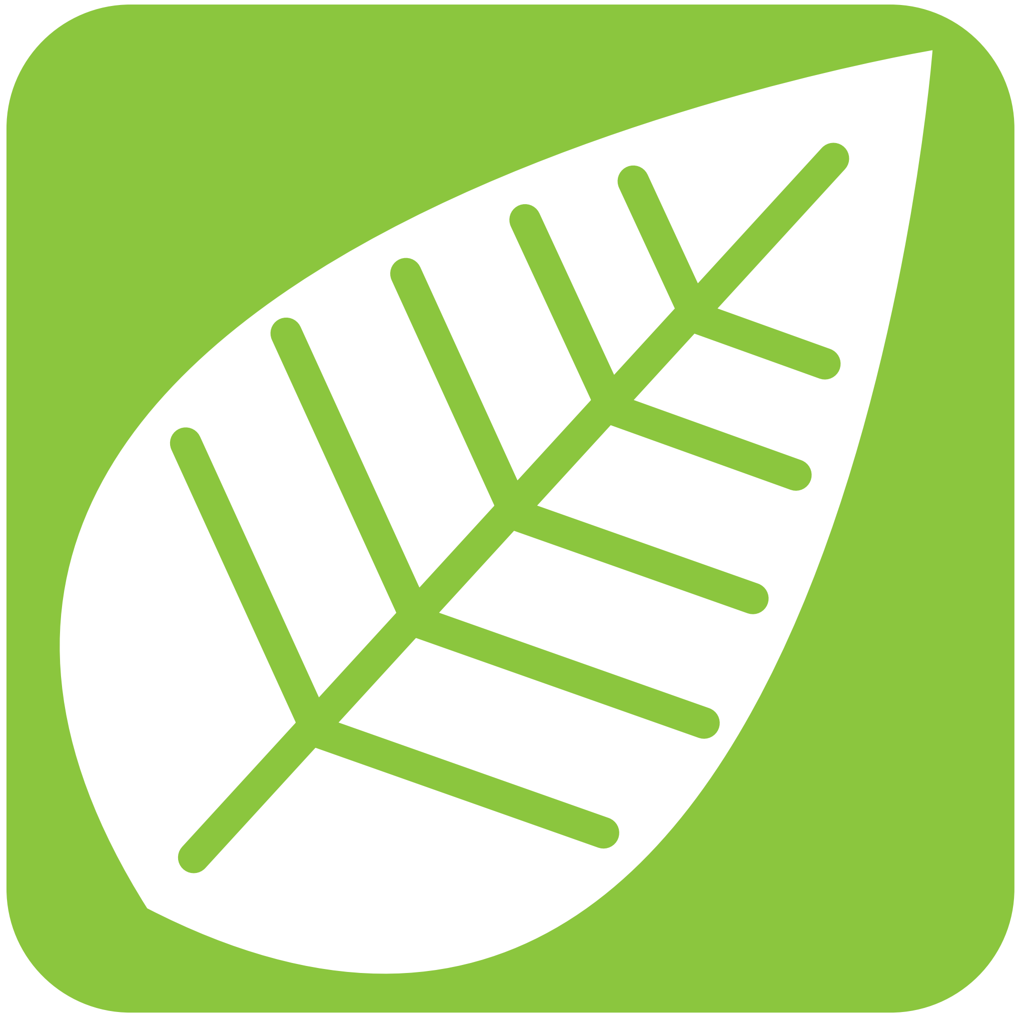

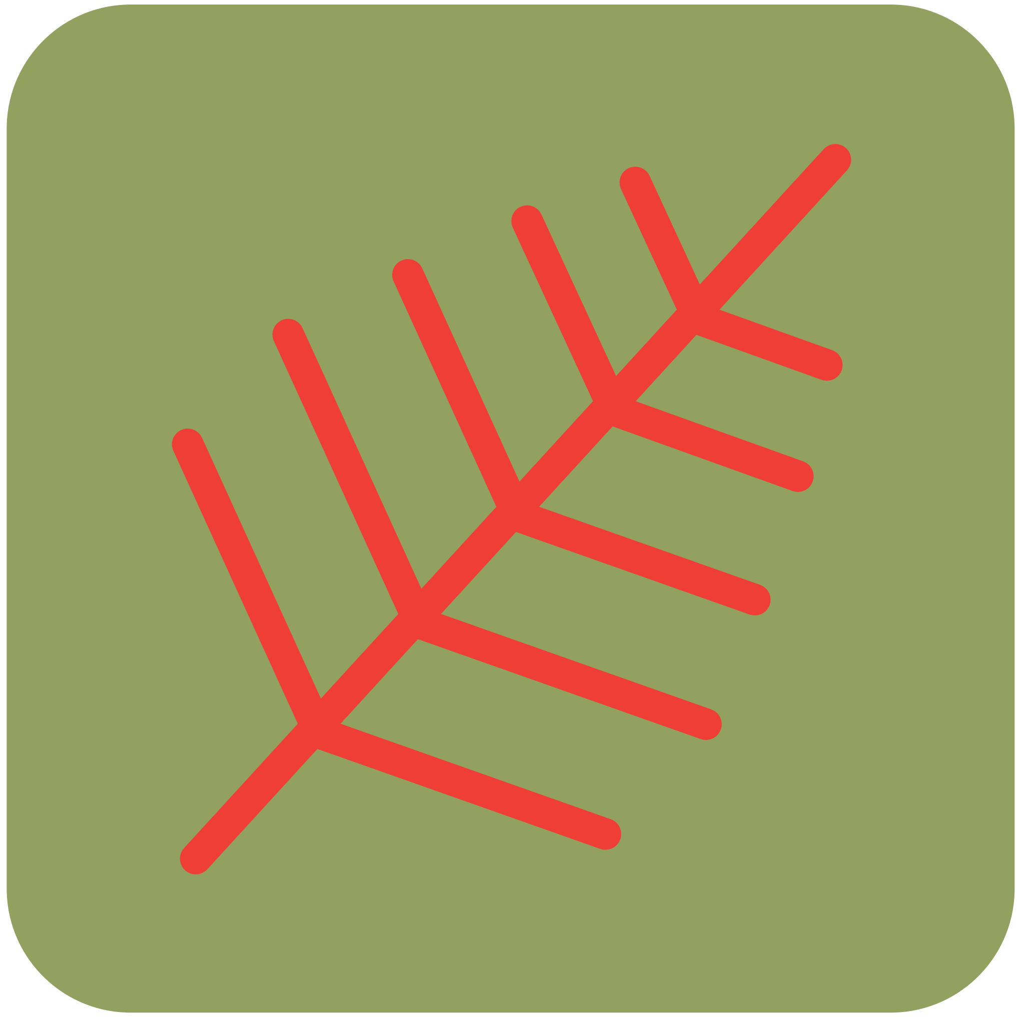

ÉCOCOMPATIBILITÉ

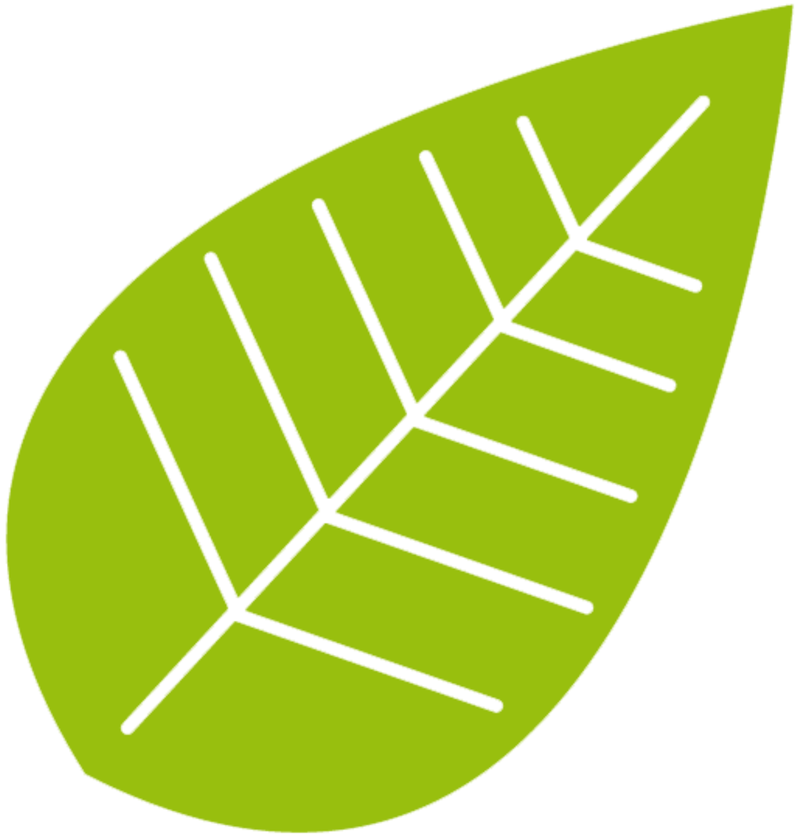

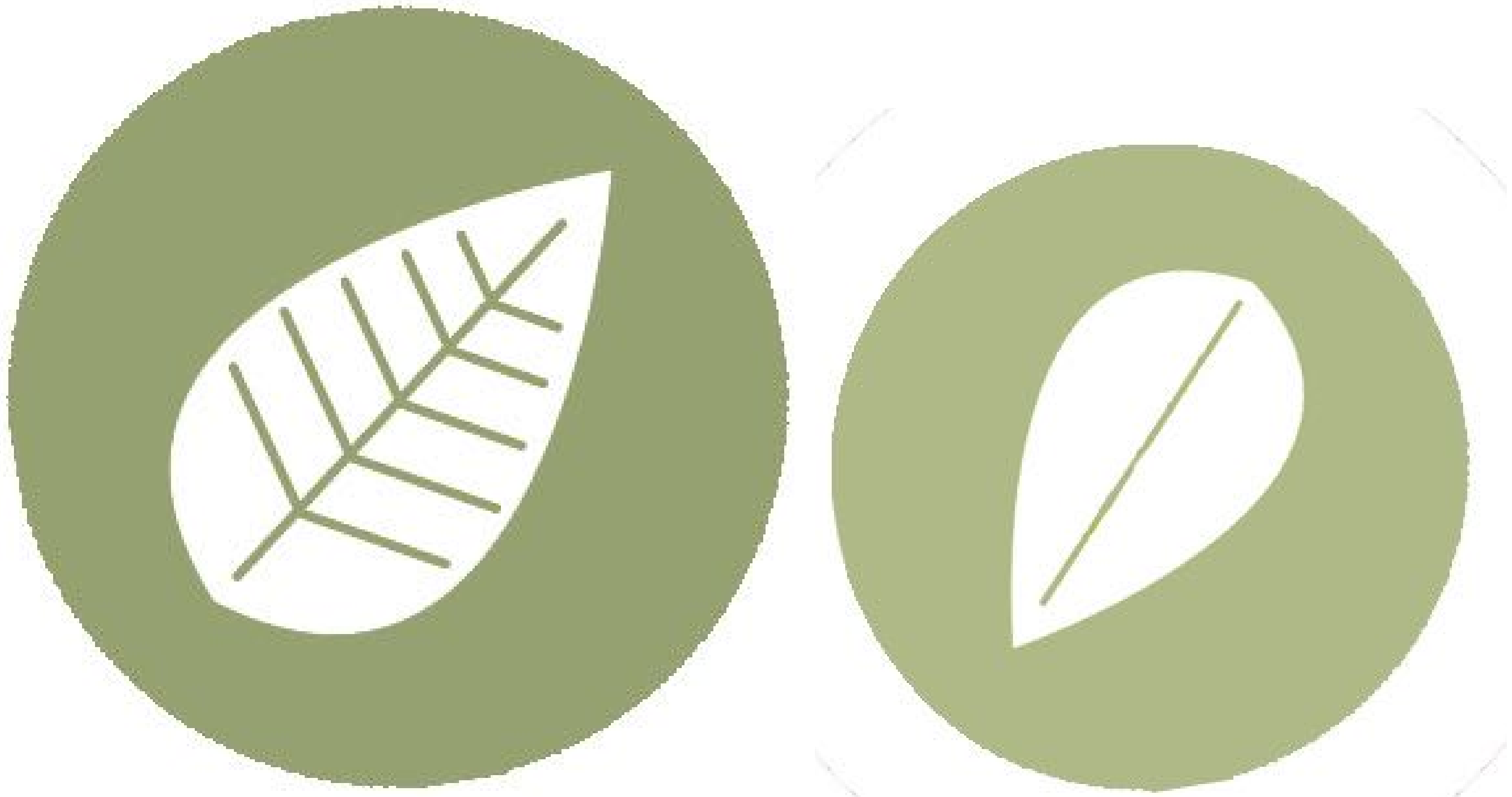

MARGAUX KHALIL

ÉCOCOMPATIBILITÉ

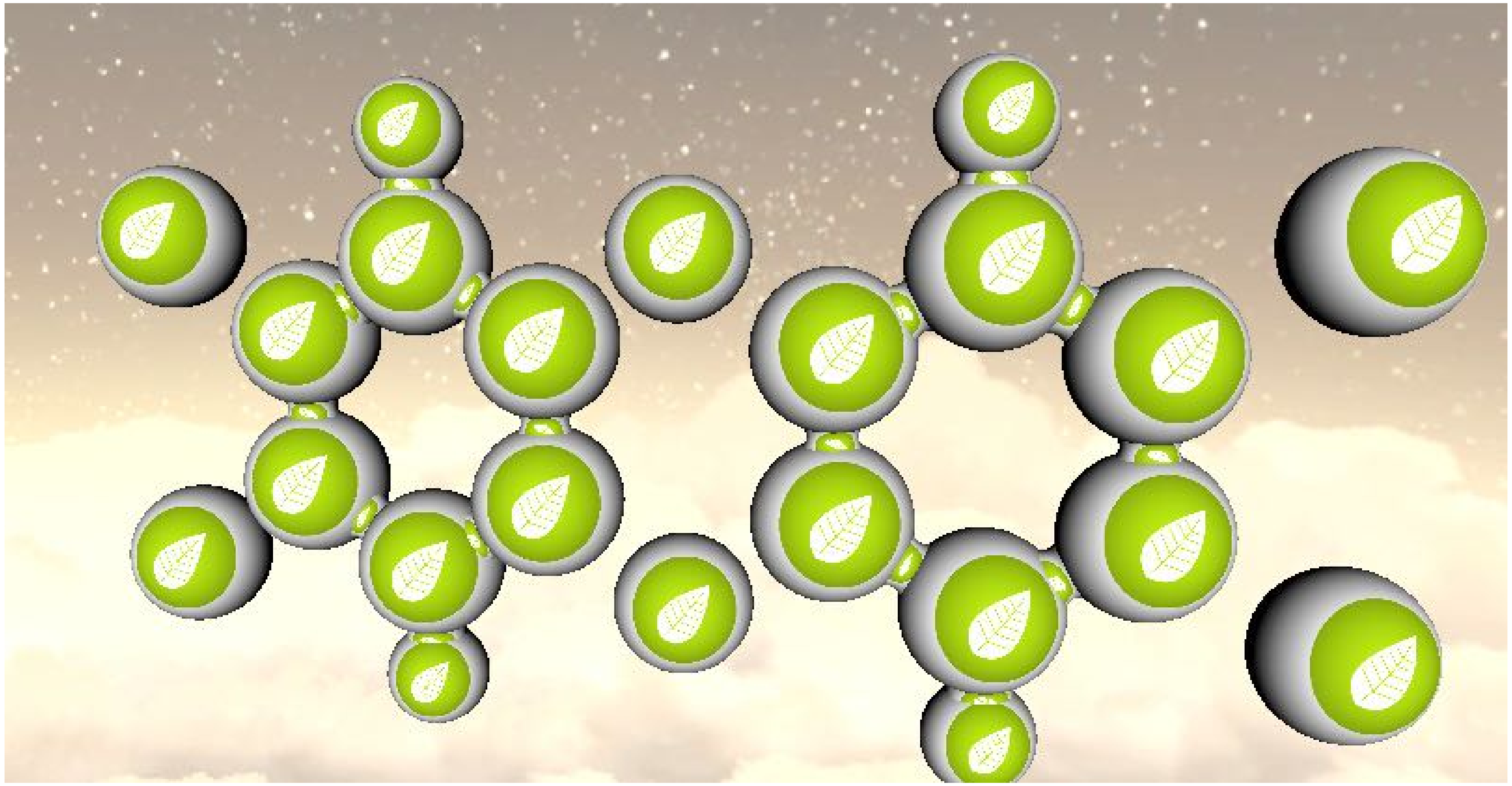

MARGAUX KHALIL

ÉCOCOMPATIBILITÉ

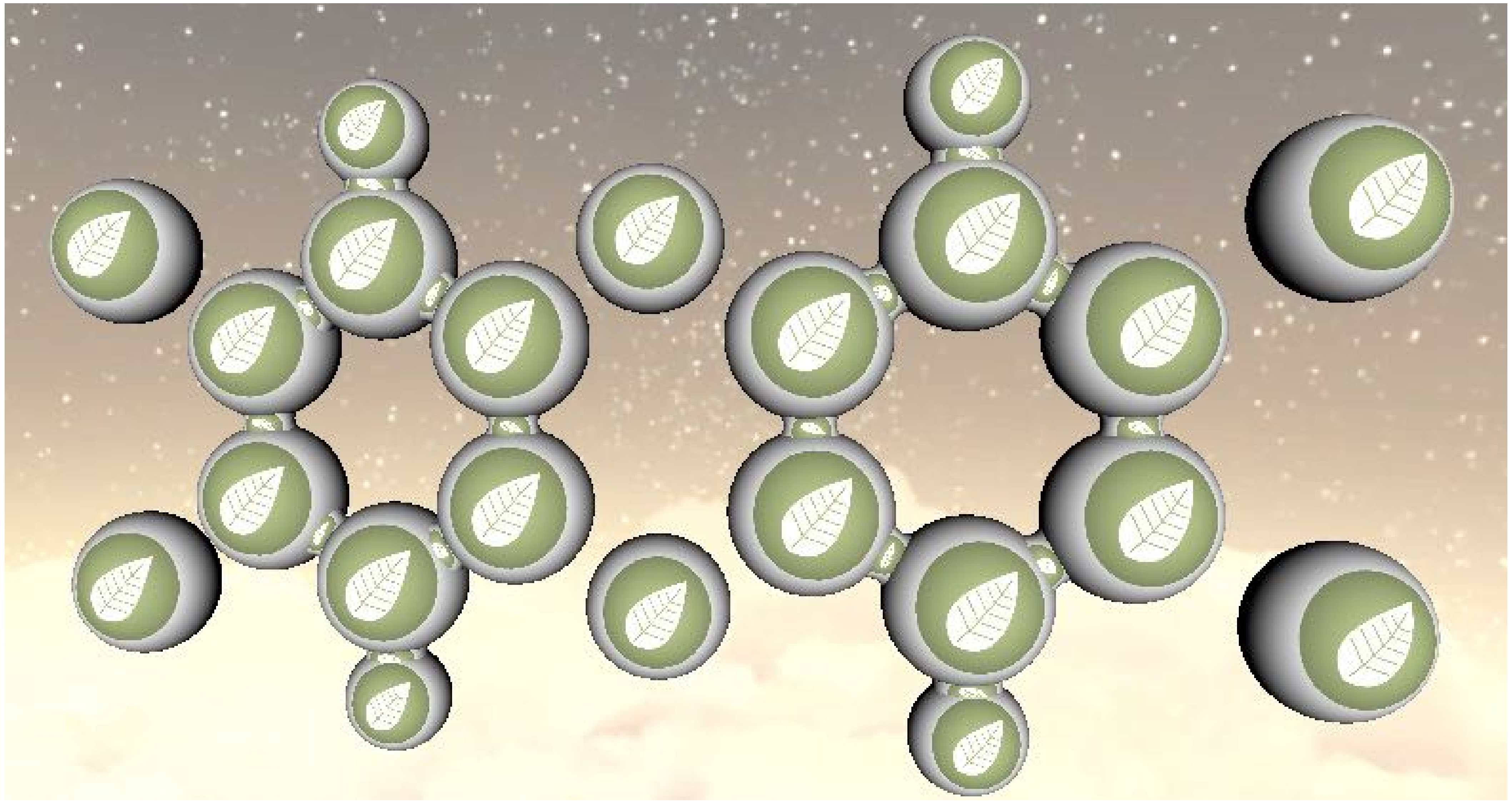

MARGAUX KHALIL

ÉCOCOMPATIBILITÉ

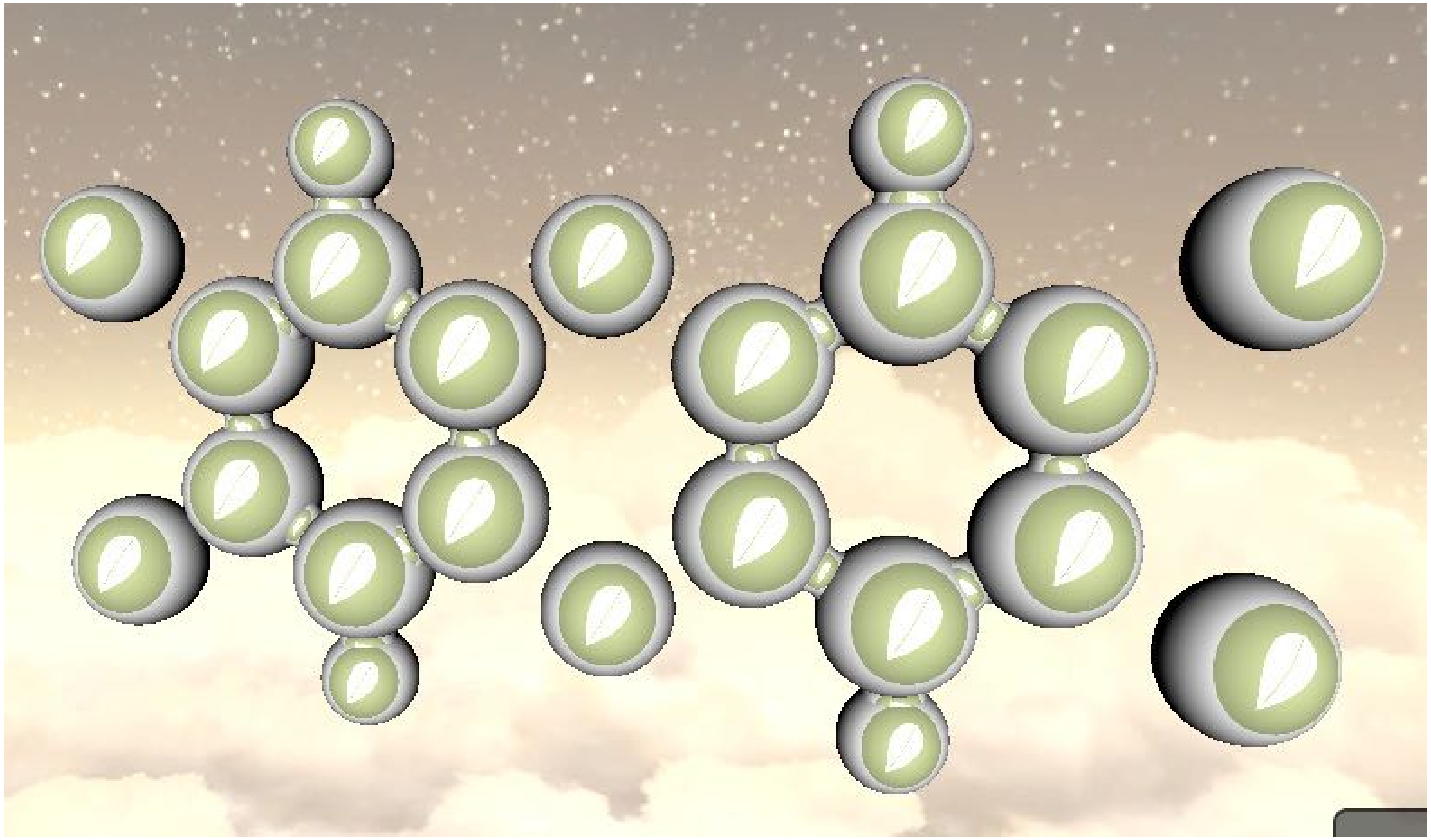

MARGAUX KHALIL

TOXICITE

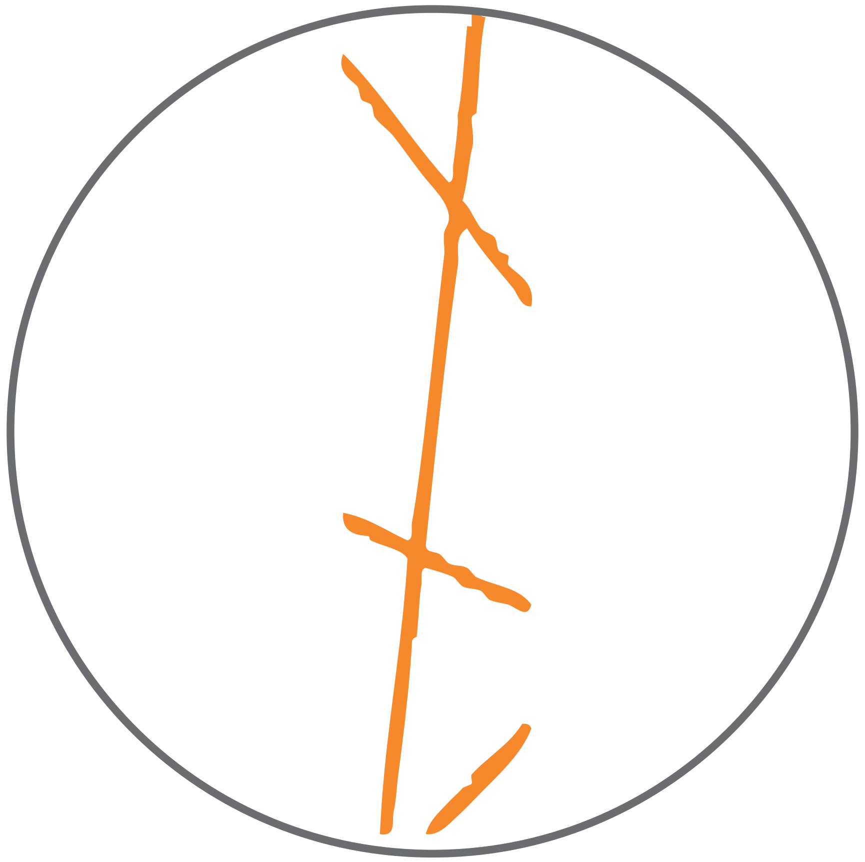

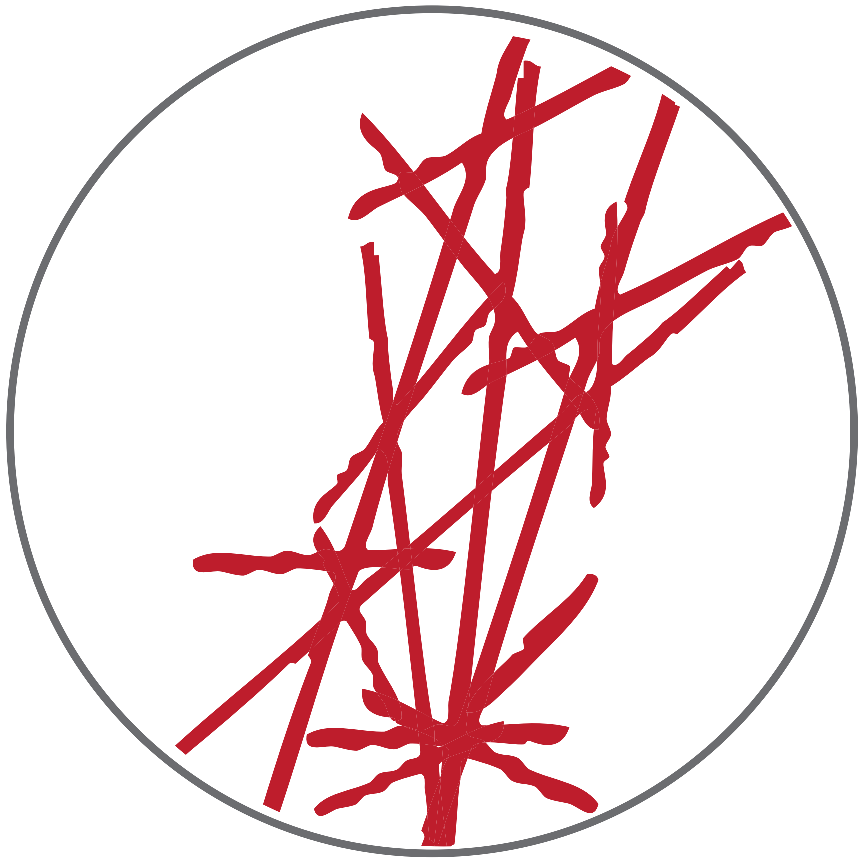

MARGAUX KHALIL

TOXICITE

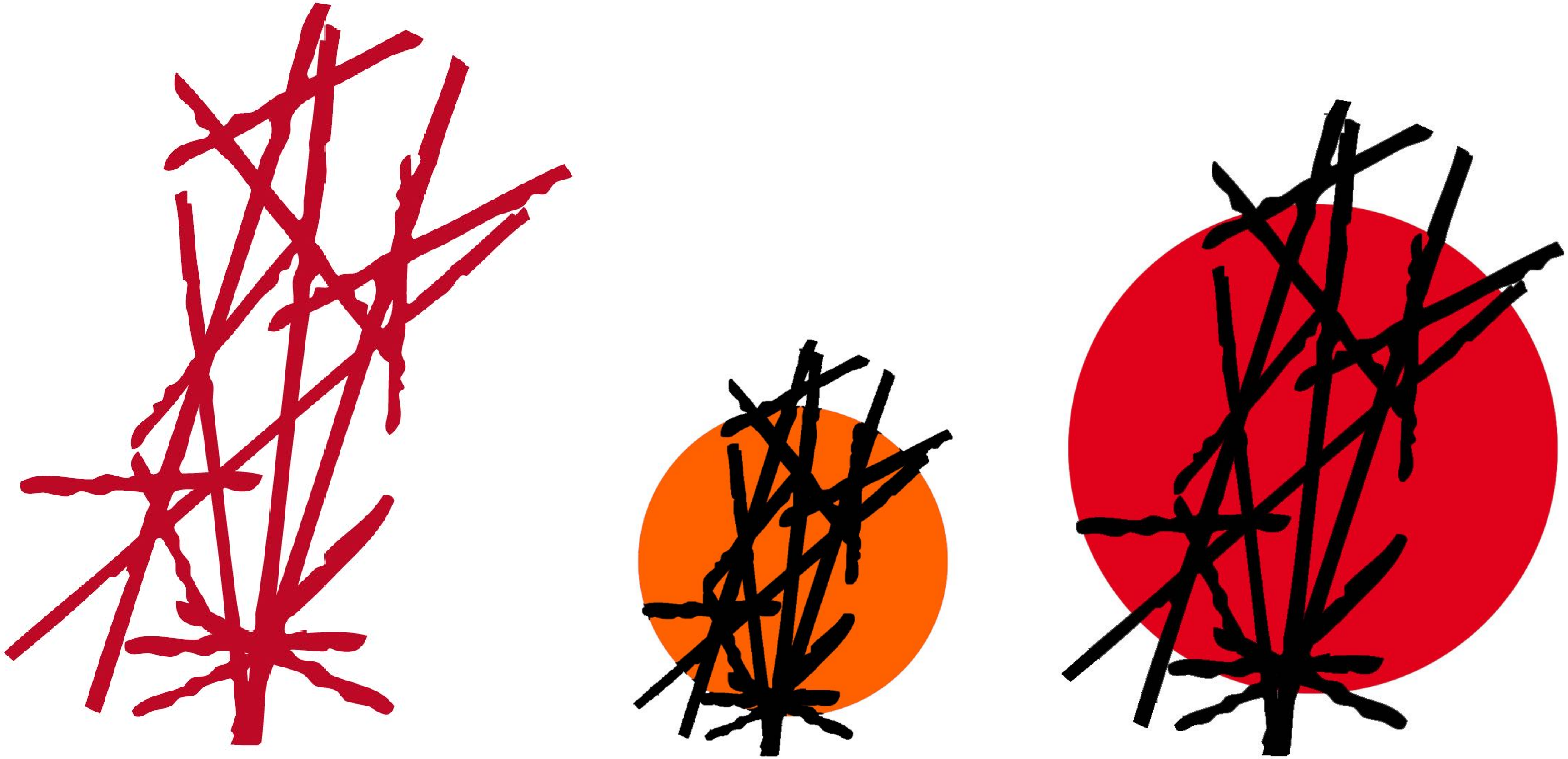

MARGAUX KHALIL

TOXICITE

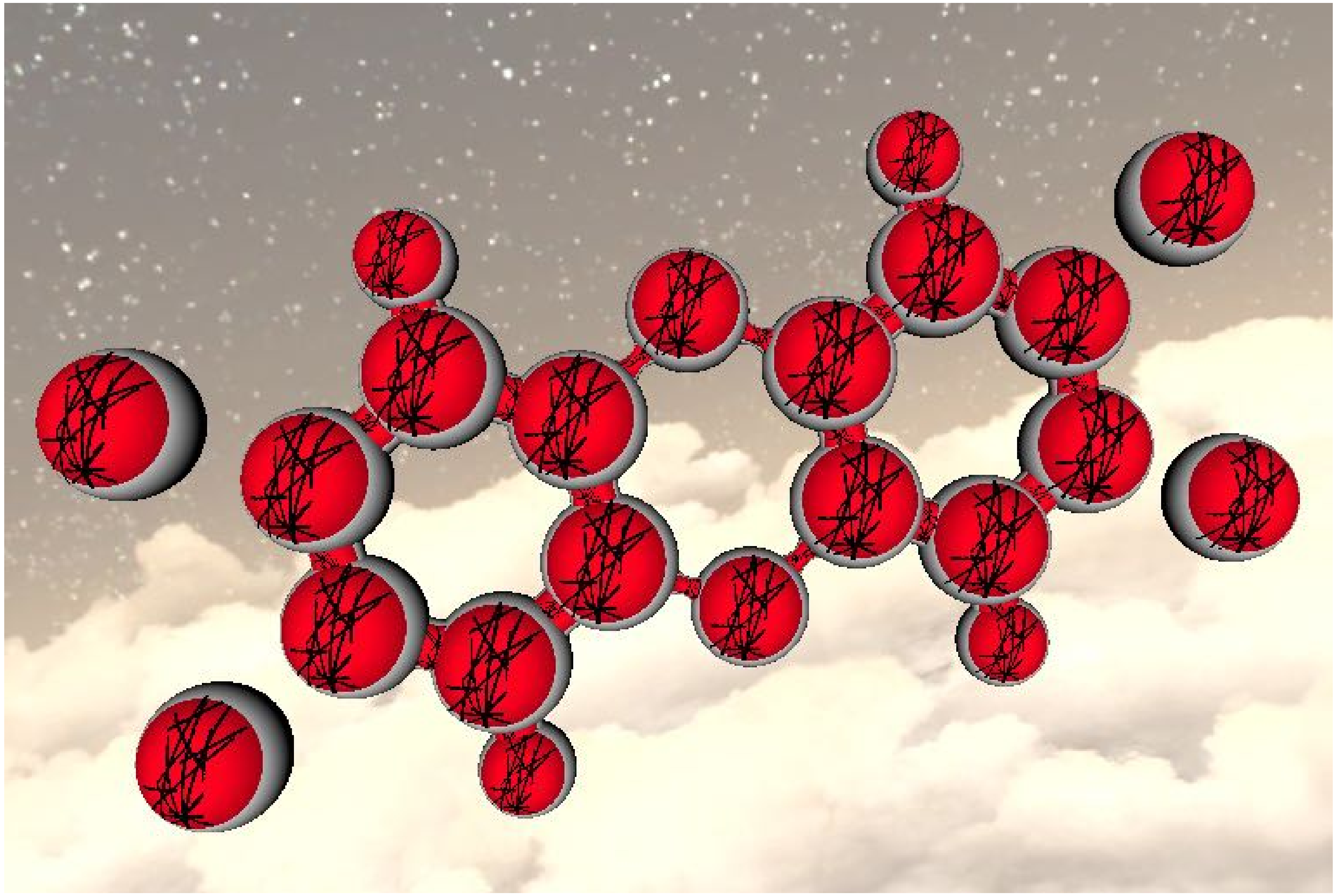

MARGAUX KHALIL

TOXICITE

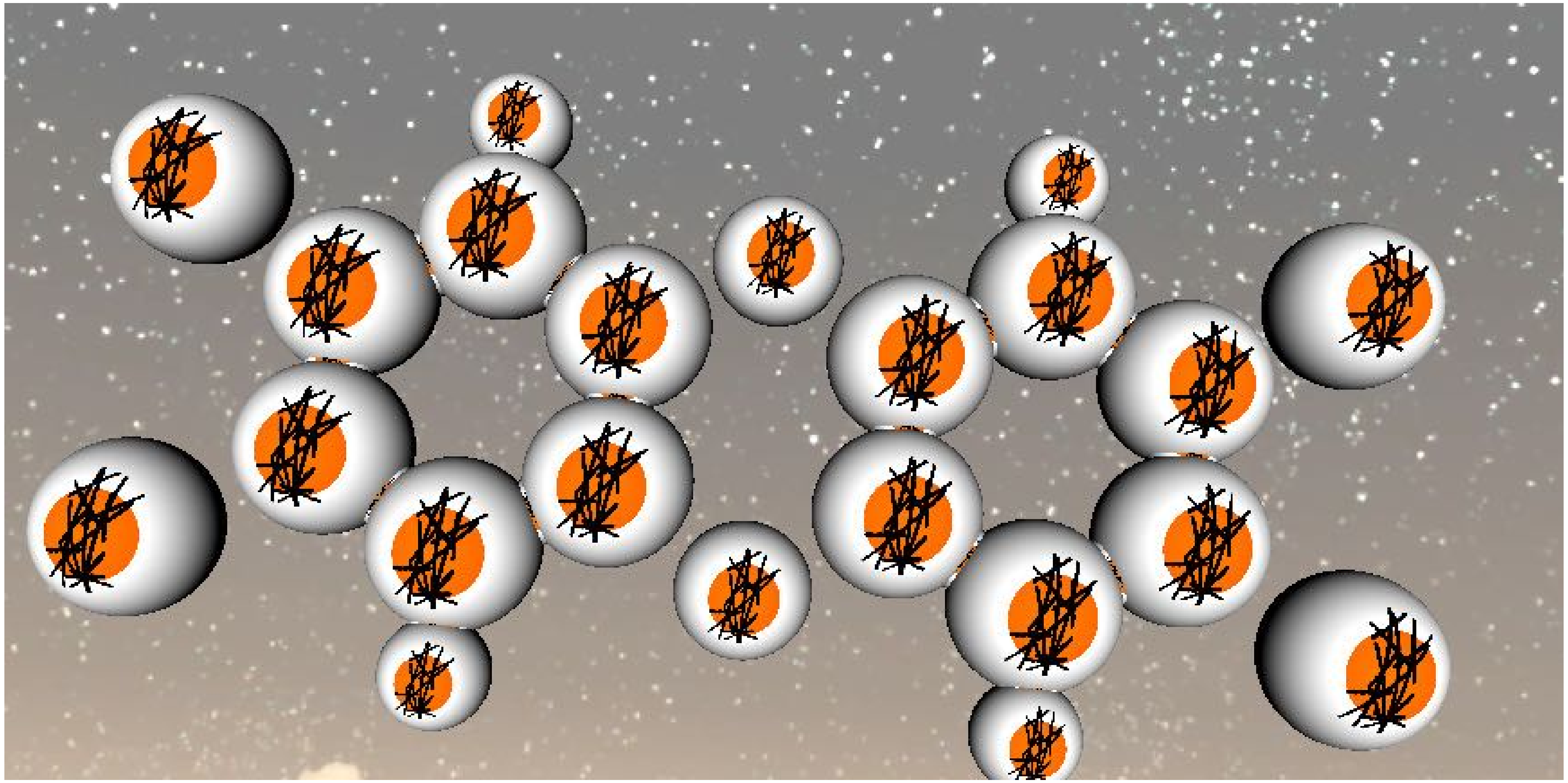

MARGAUX KHALIL

POLARITE

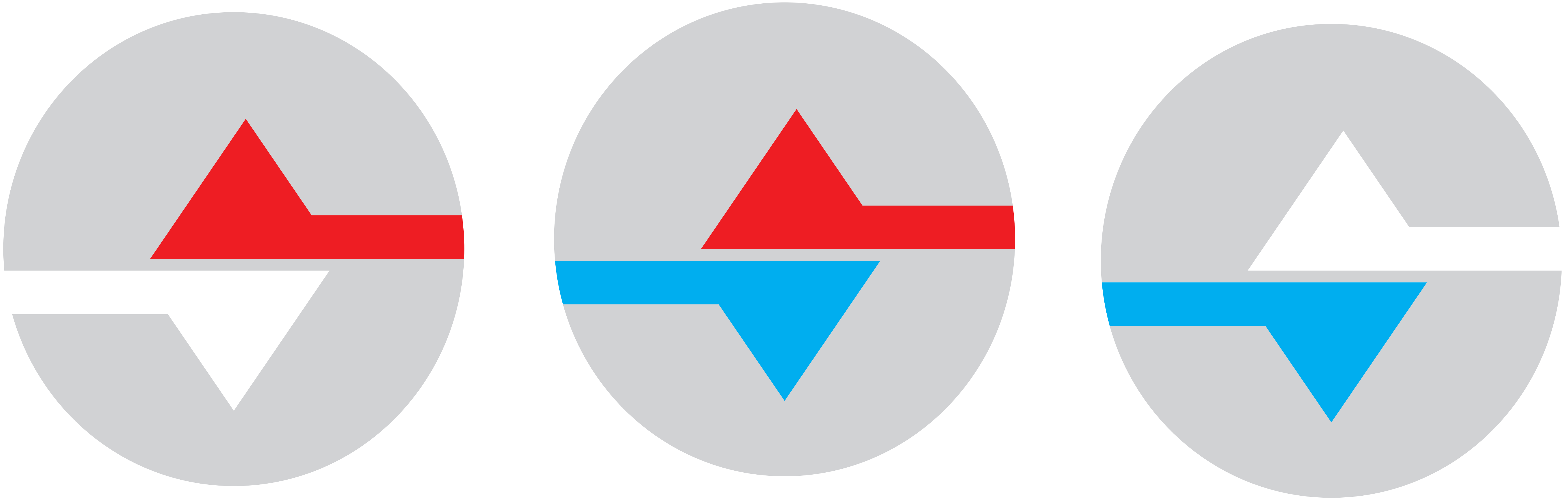

MARGAUX KHALIL

POLARITE

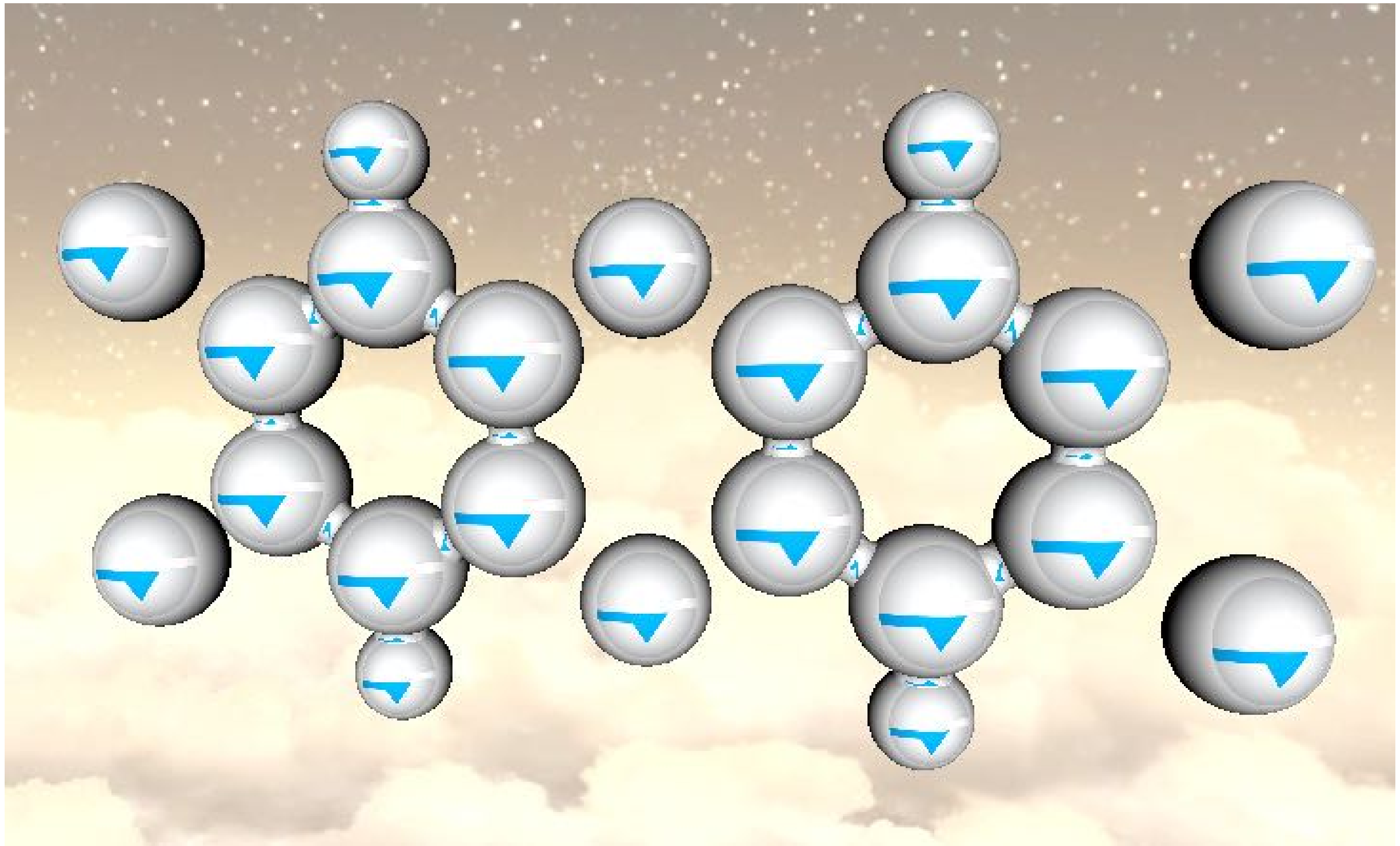

MARGAUX KHALIL

POLARITE

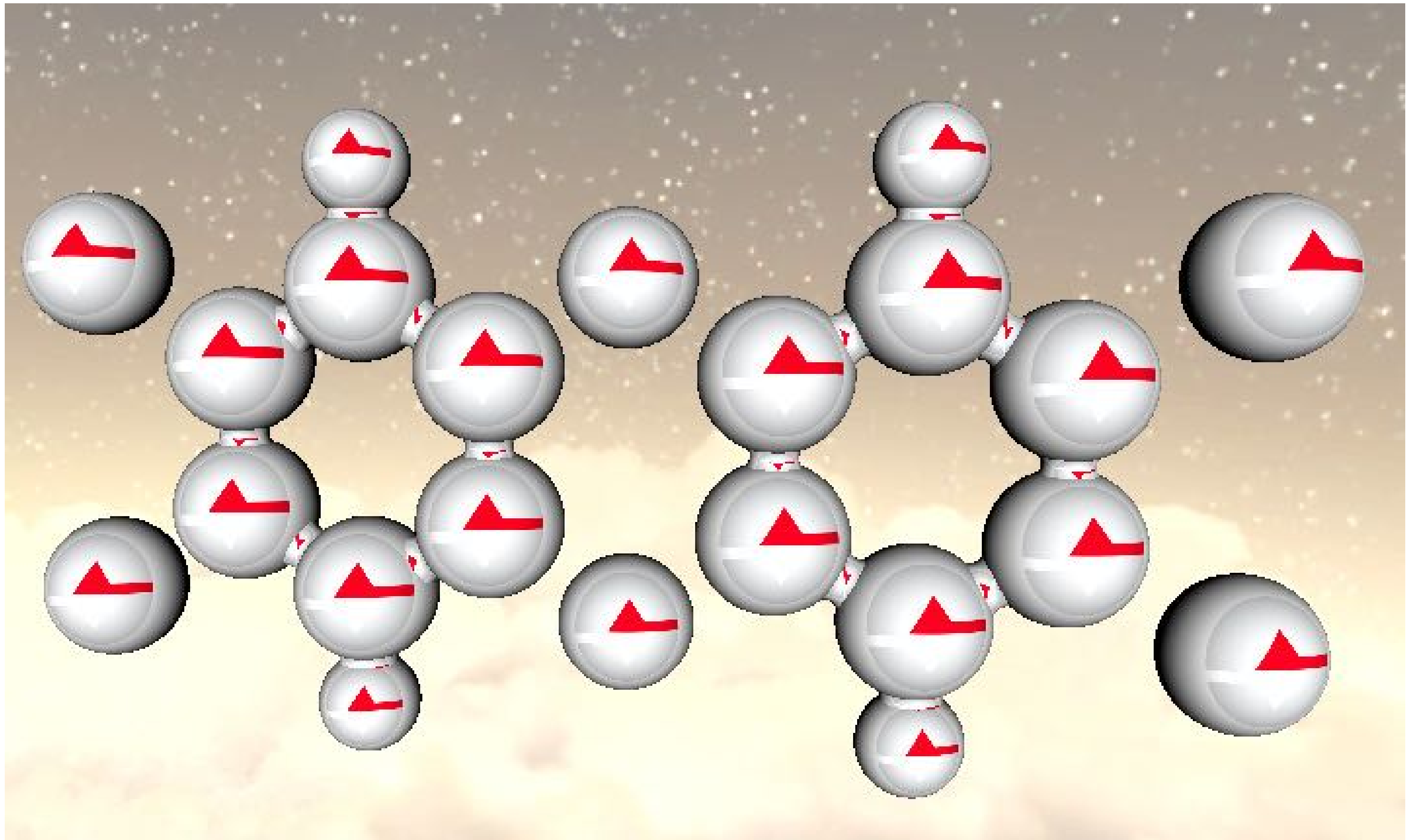

RIGIDITÉ / FLEXIBILITÉ

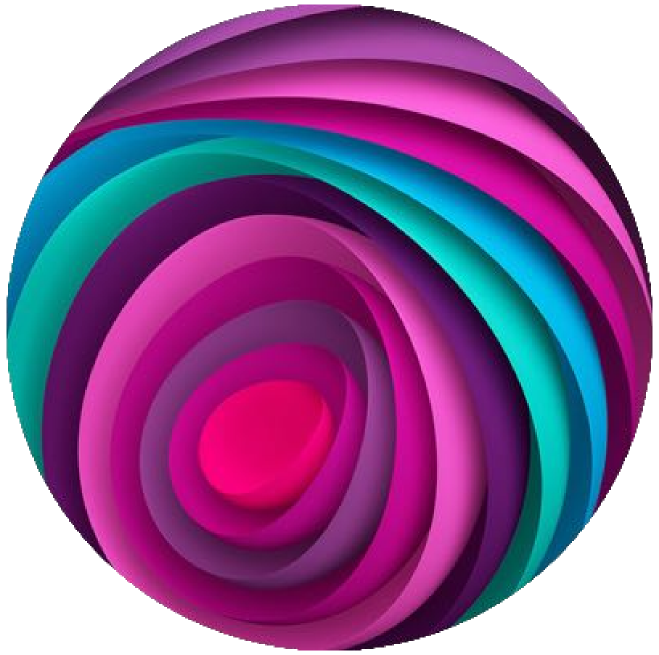

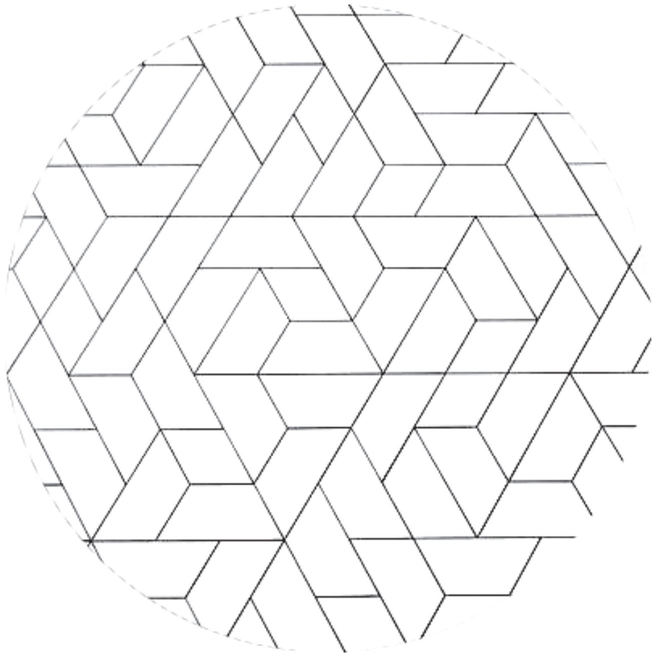

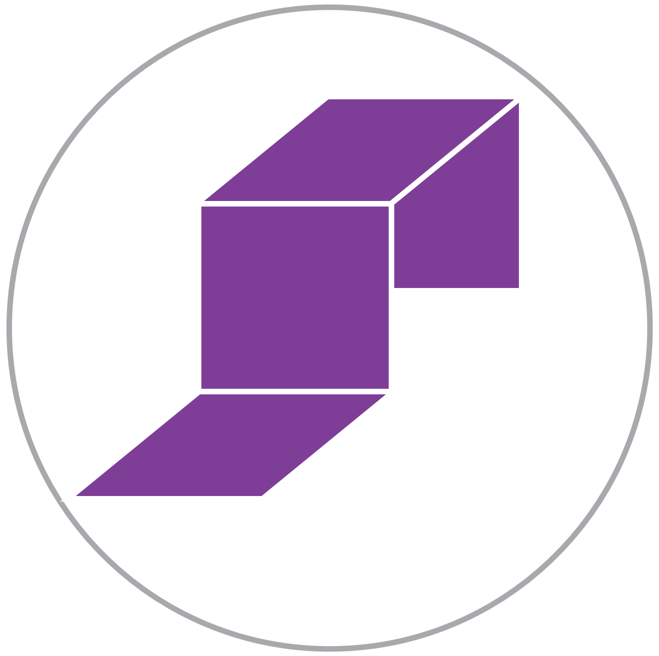

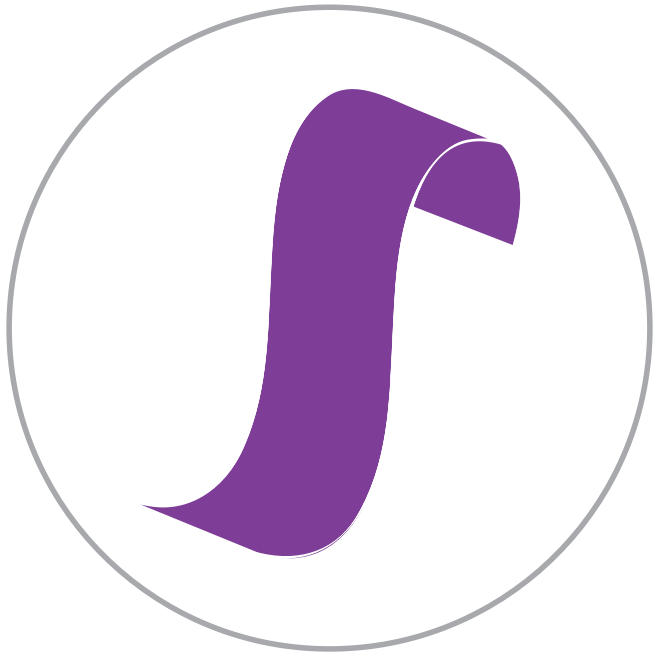

MARGAUX KHALIL

RIGIDITÉ / FLEXIBILITÉ

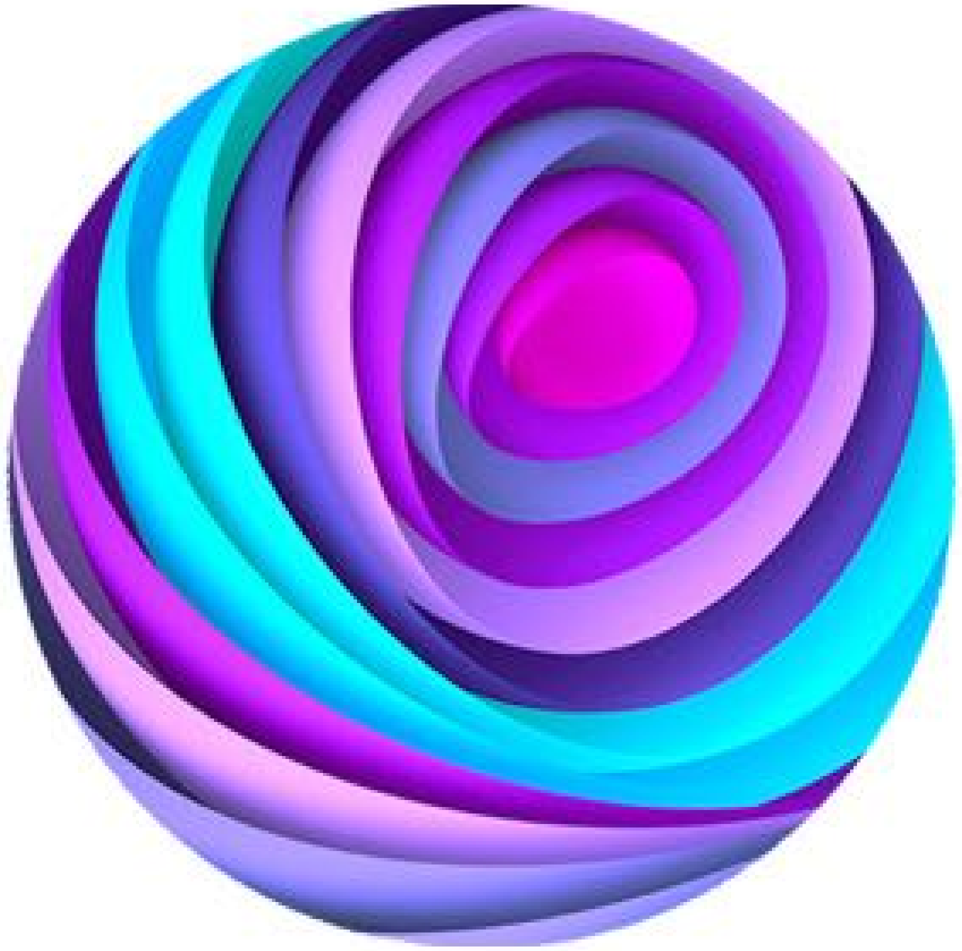

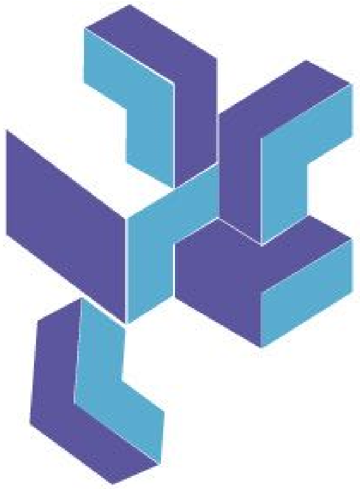

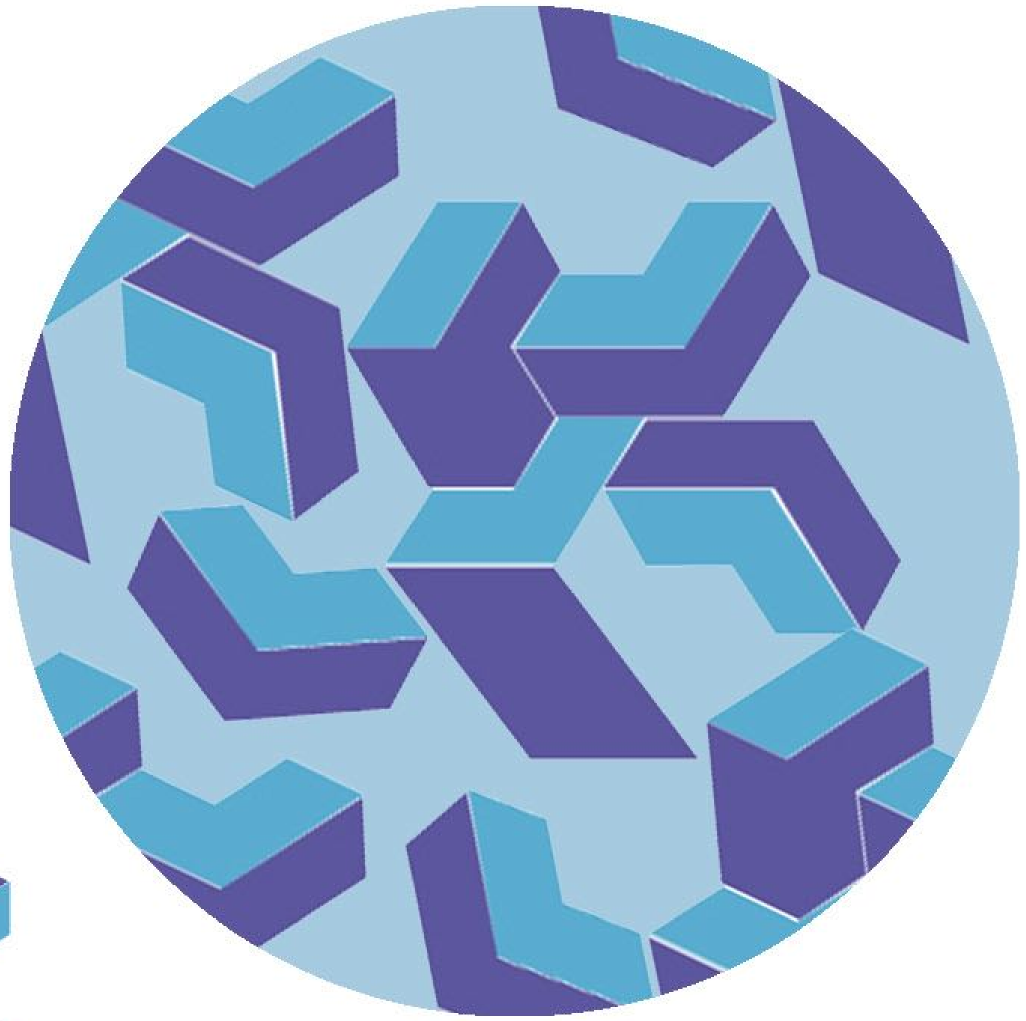

MARGAUX KHALIL

RIGIDITÉ / FLEXIBILITÉ

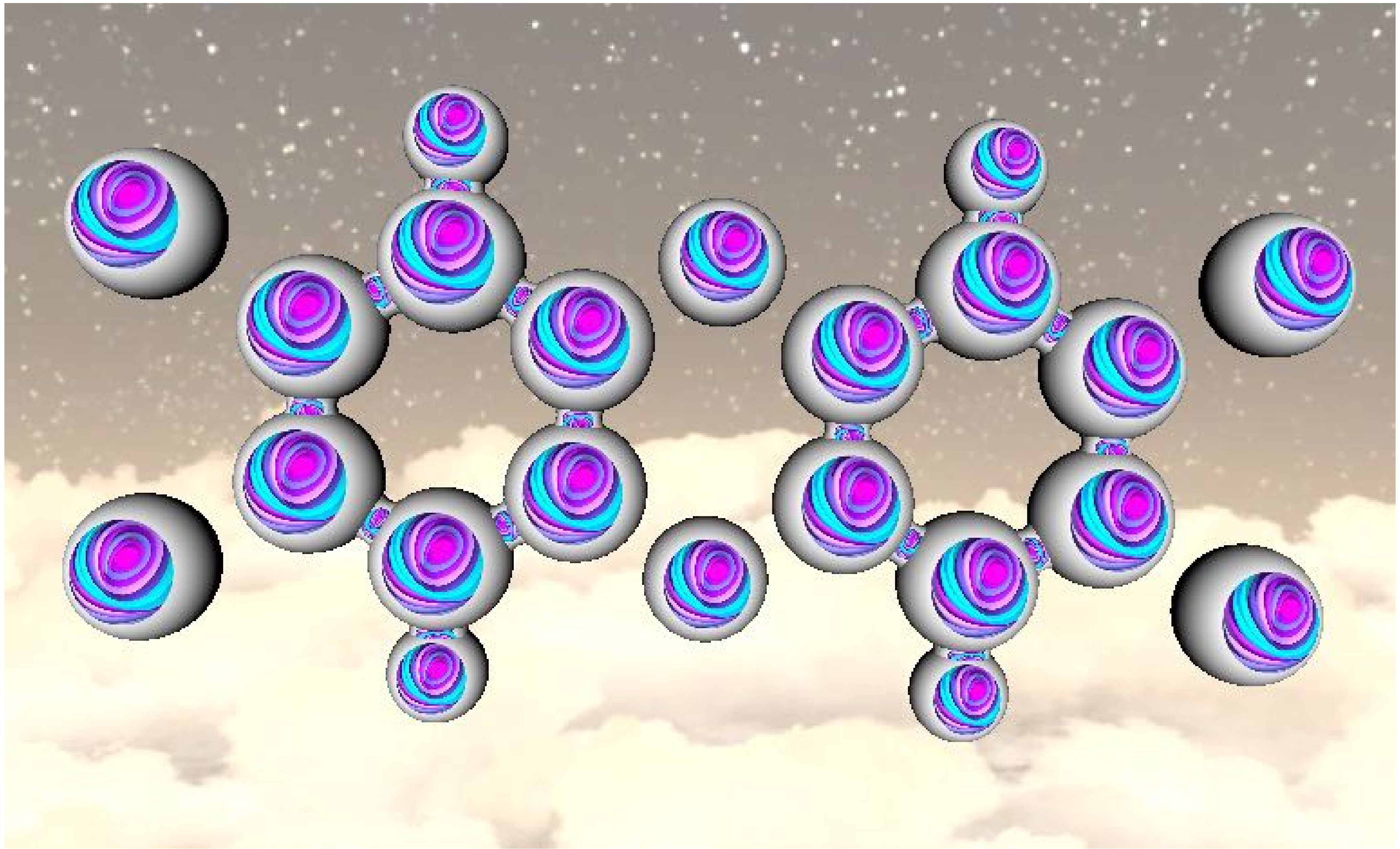

MARGAUX KHALIL

RIGIDITÉ / FLEXIBILITÉ

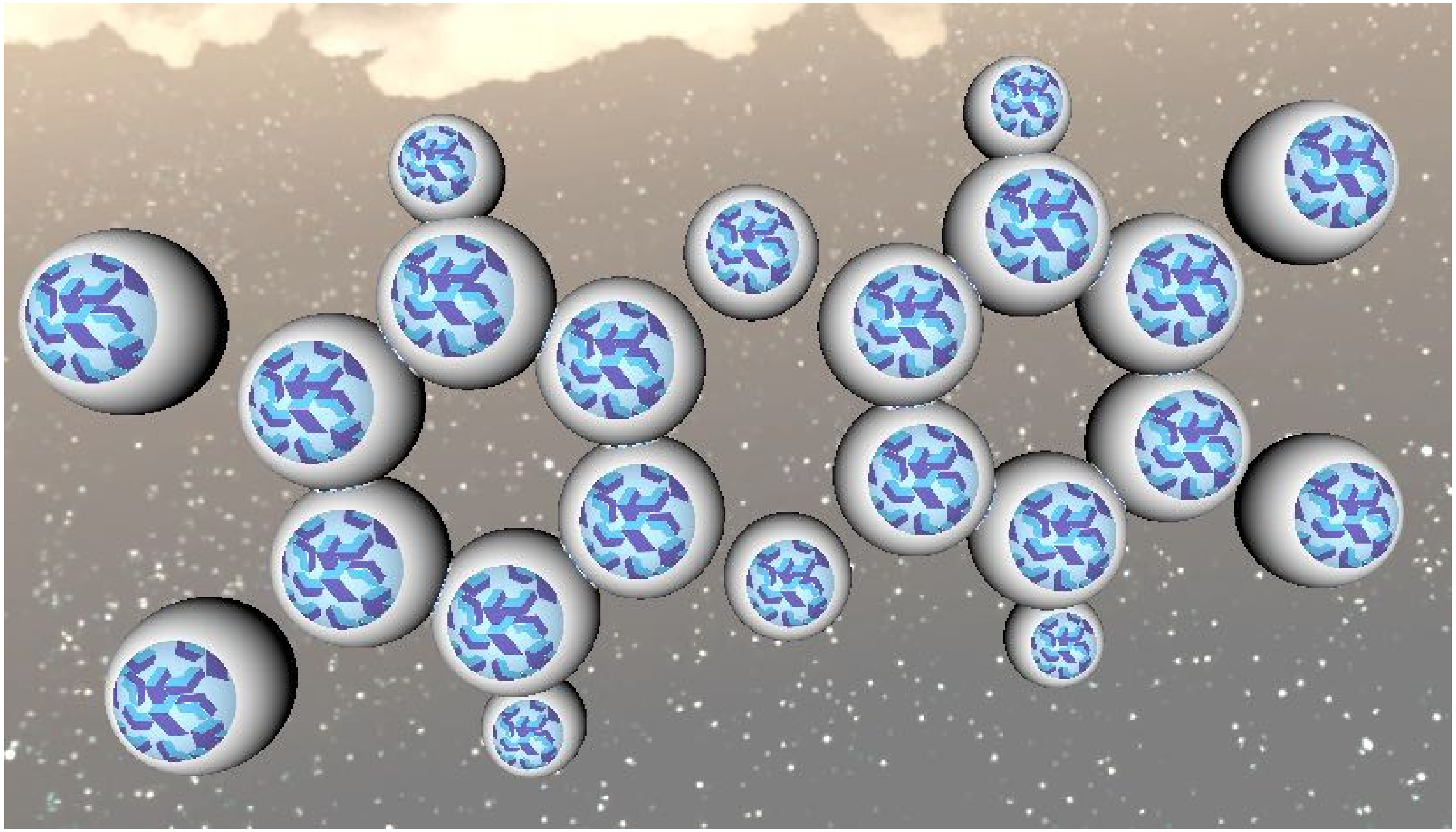

MARGAUX KHALIL

ACTIVITÉ / INACTIVITÉ

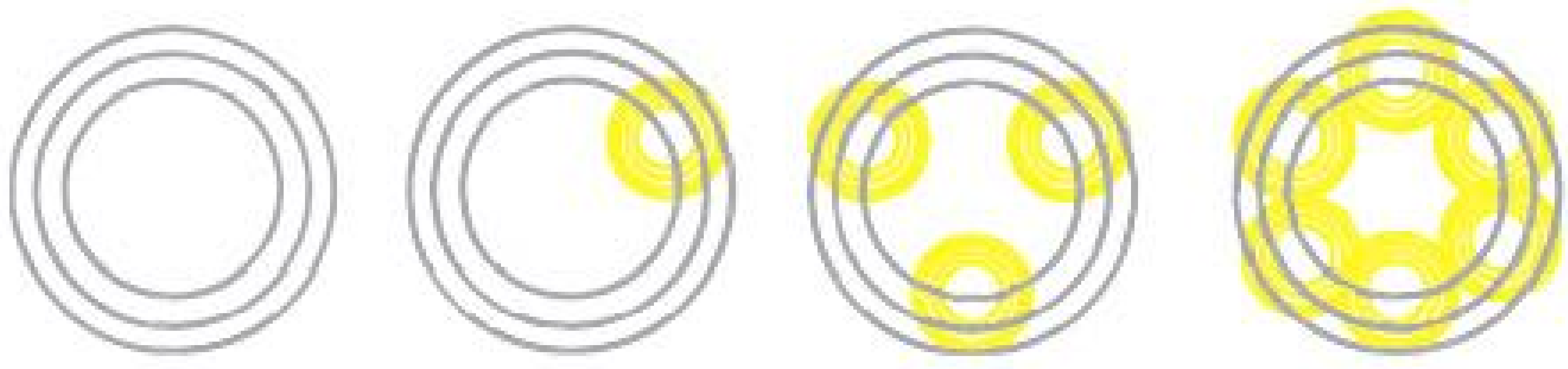

MARGAUX KHALIL

ACTIVITÉ / INACTIVITÉ

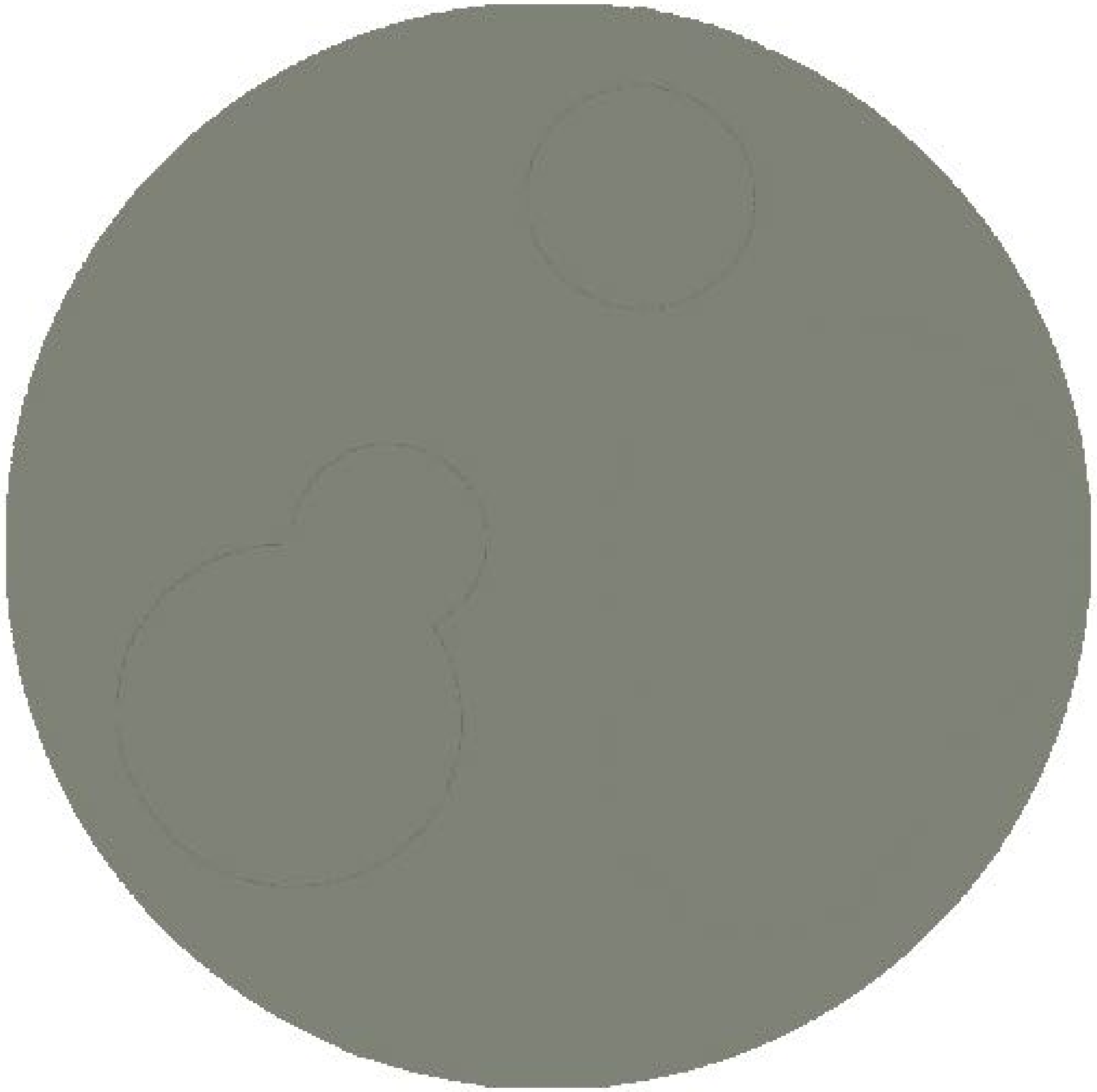

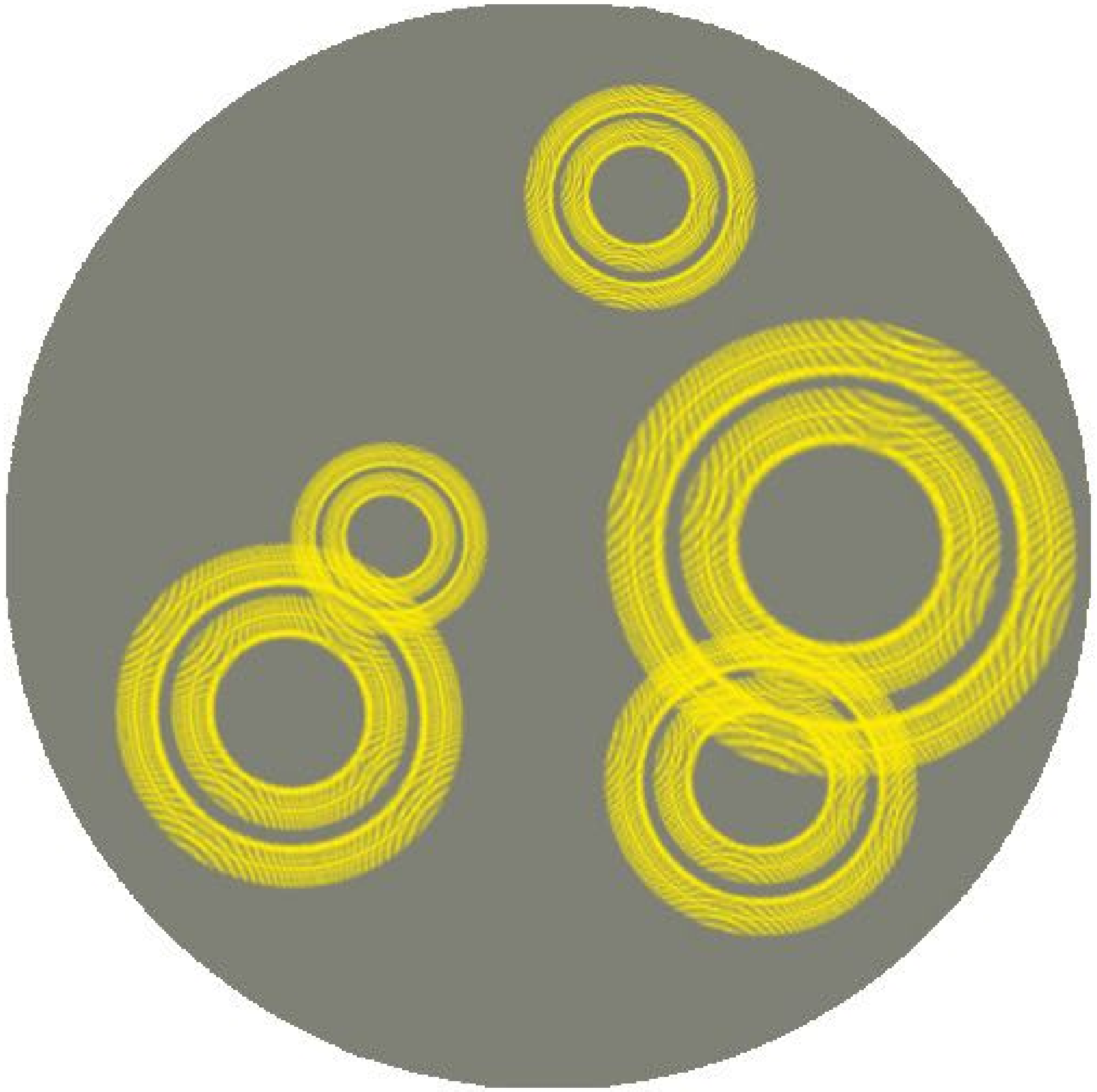

MARGAUX KHALIL

ACTIVITÉ / INACTIVITÉ

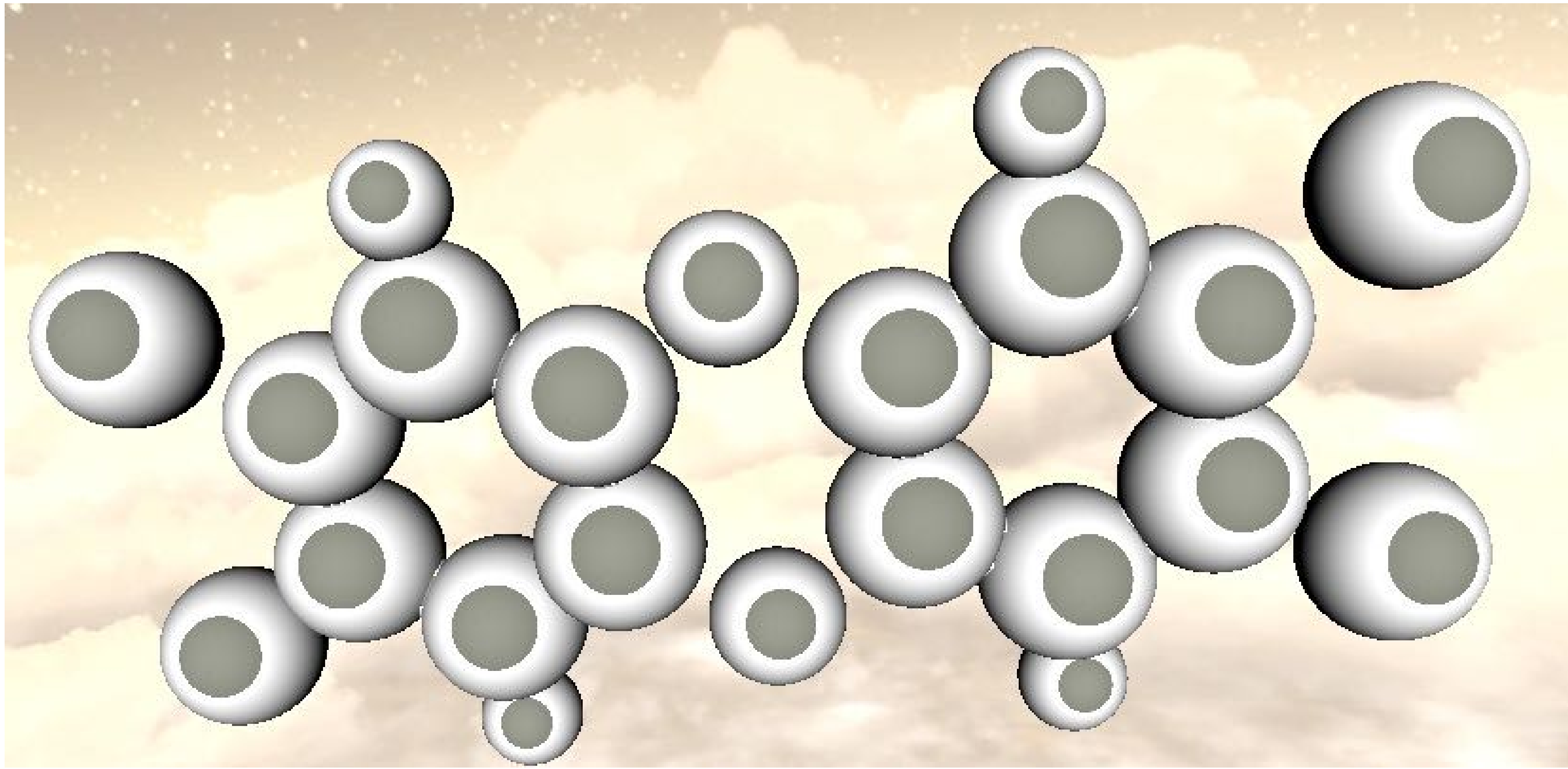

MARGAUX KHALIL

ACTIVITÉ / INACTIVITÉ

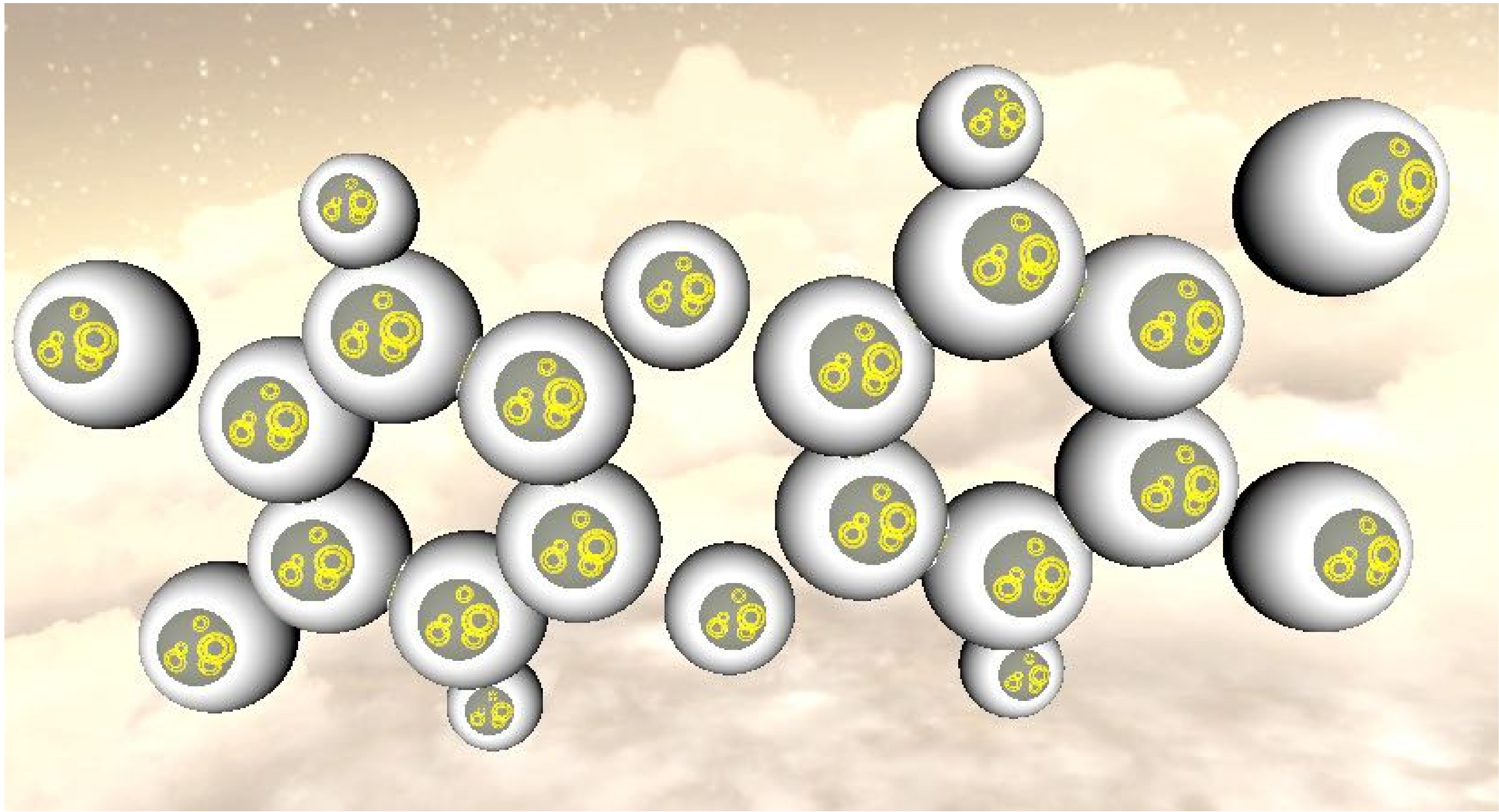

MARGAUX KHALIL

BON / MAUVAIS

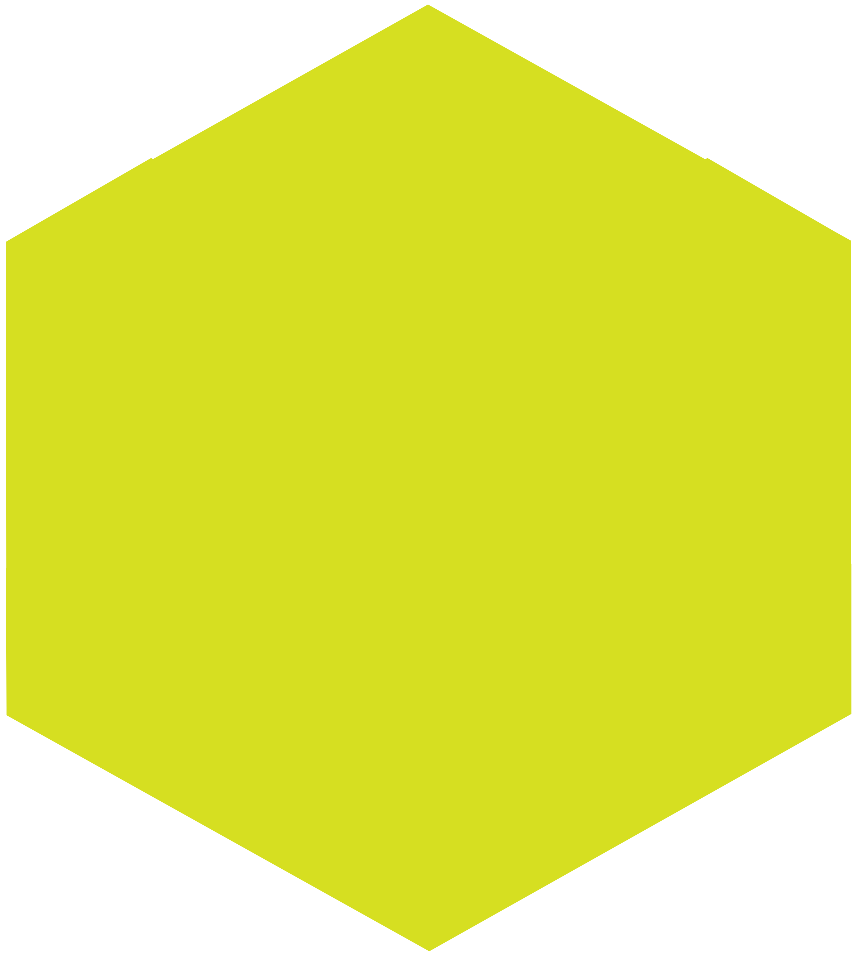

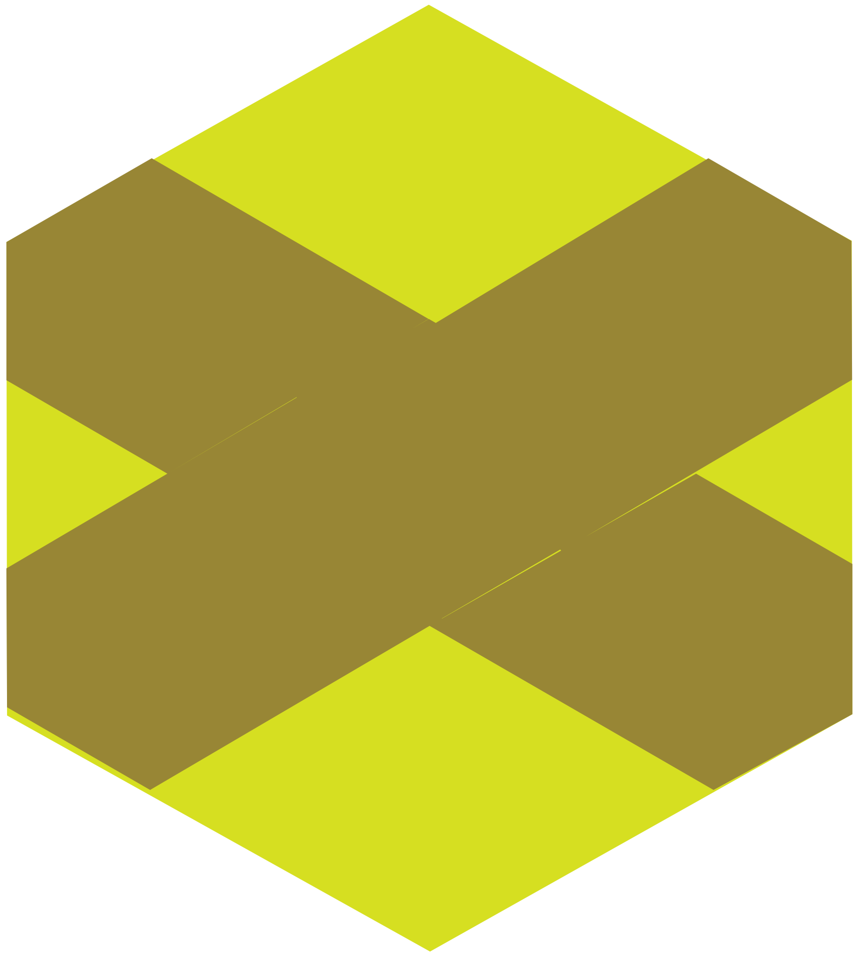

MARGAUX KHALIL

BON / MAUVAIS

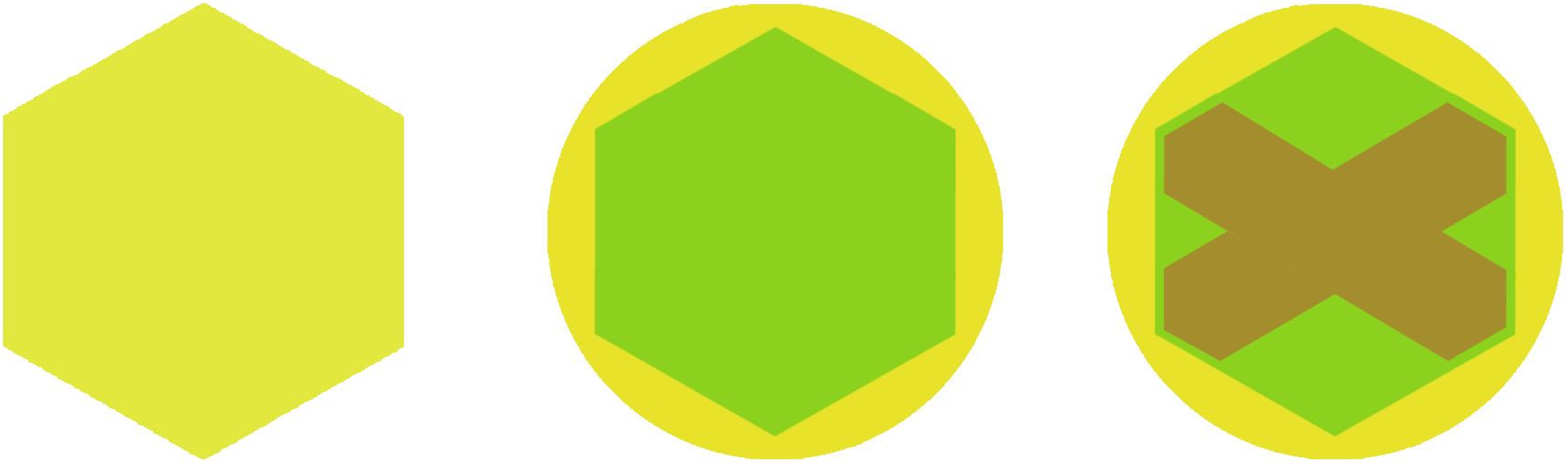

MARGAUX KHALIL

BON / MAUVAIS

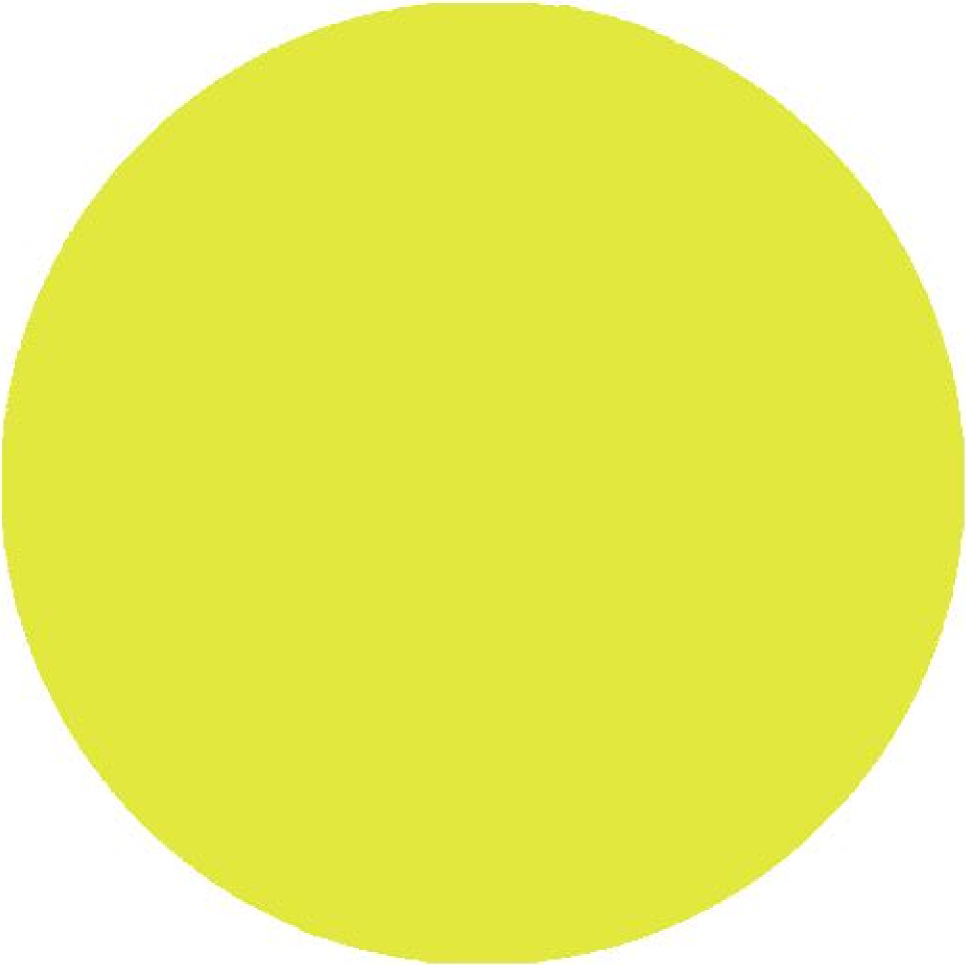

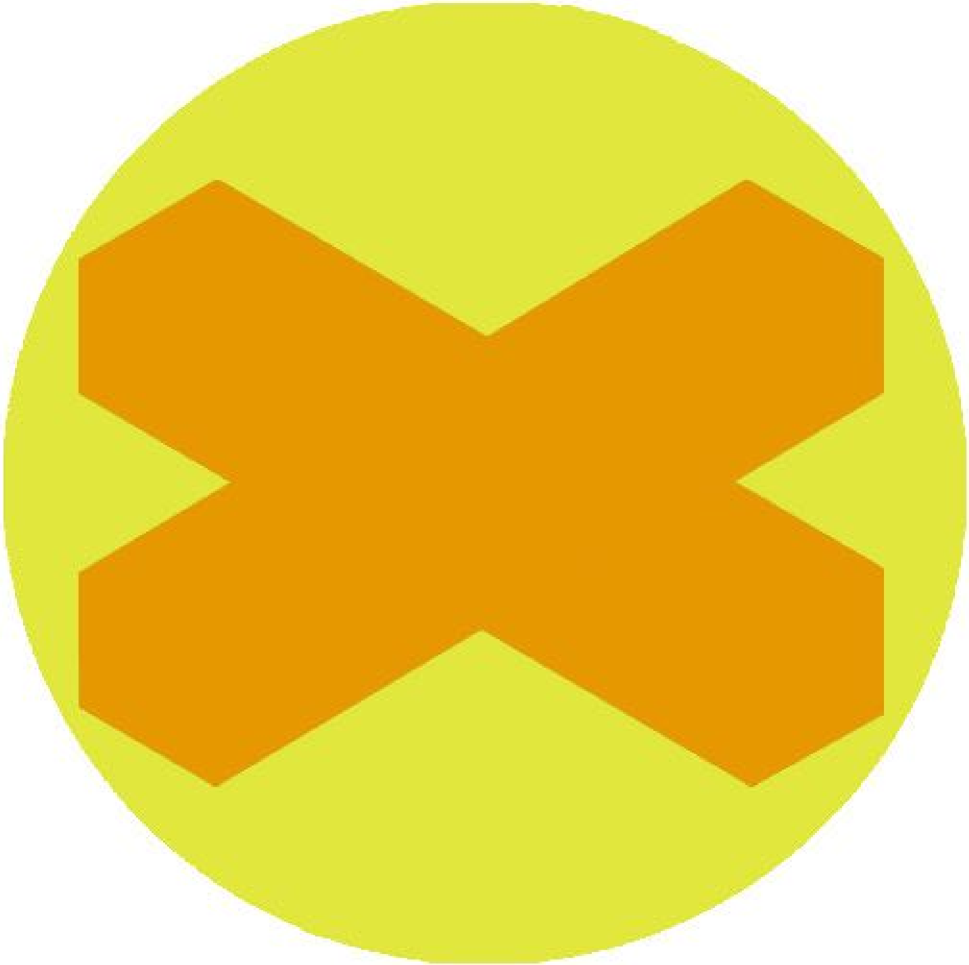

MARGAUX KHALIL

BON / MAUVAIS

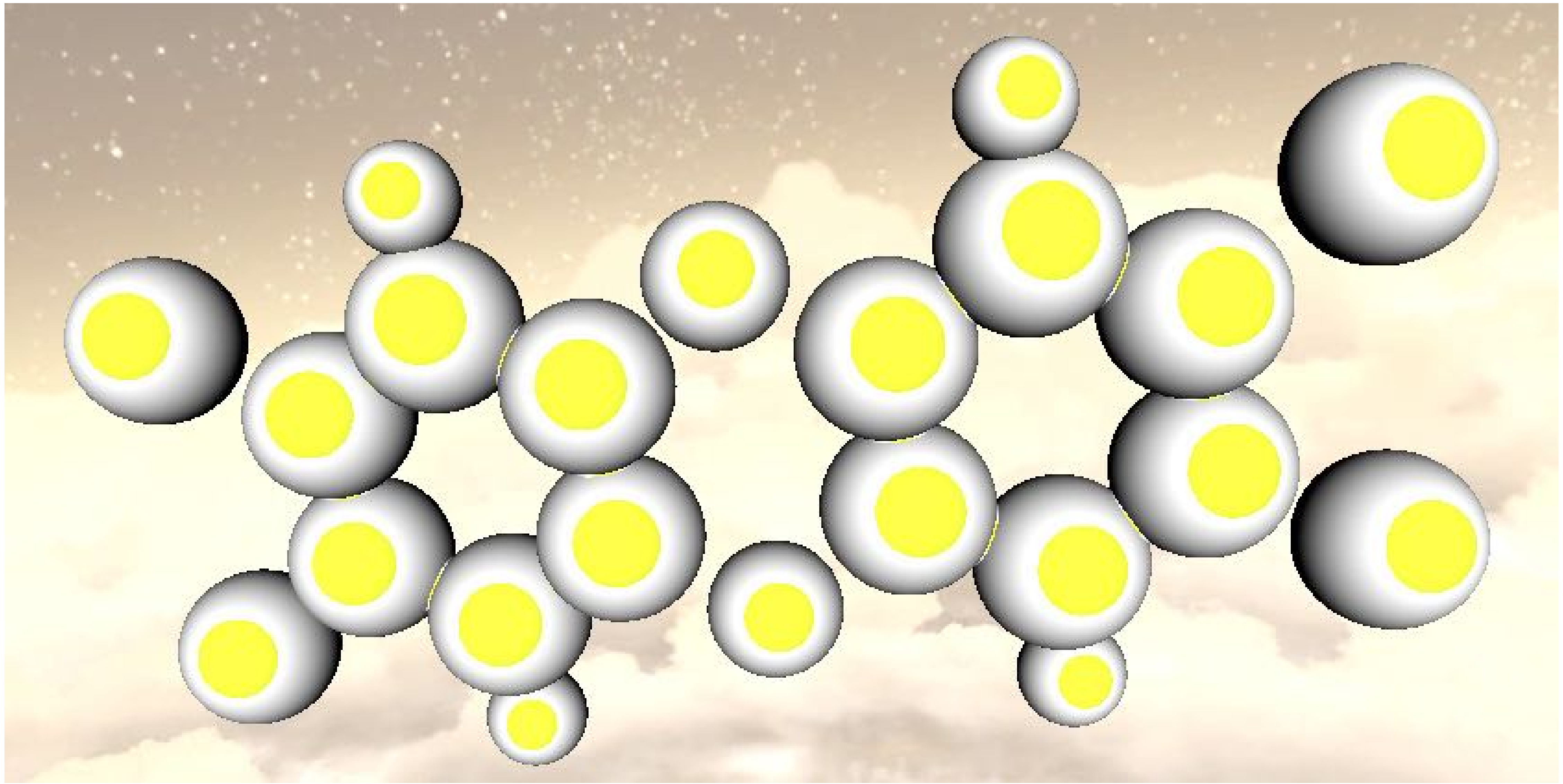

MARGAUX KHALIL

BON / MAUVAIS

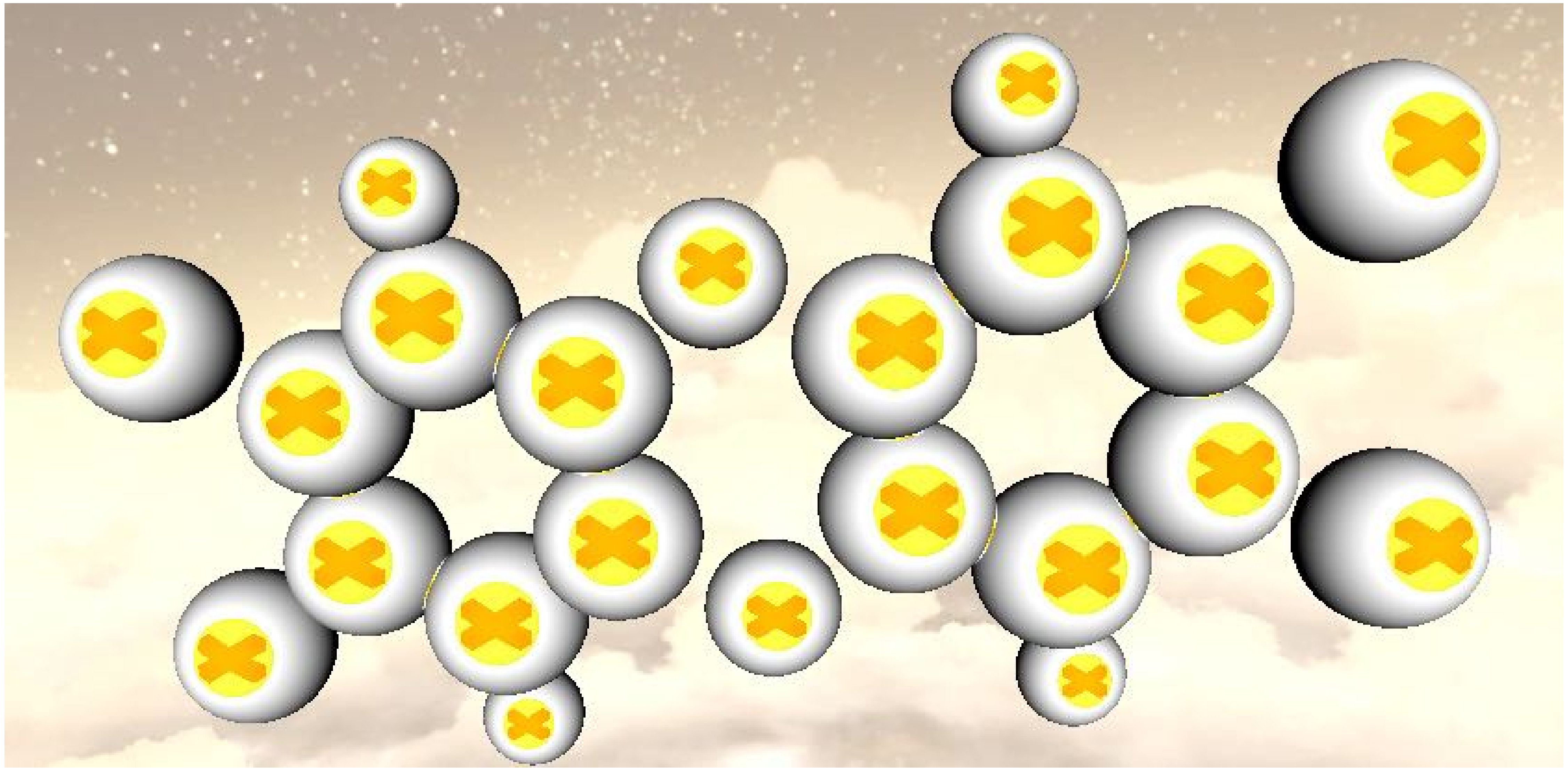

QUENTIN LEBEAU

Lit-sphère

Les pyctogrammes

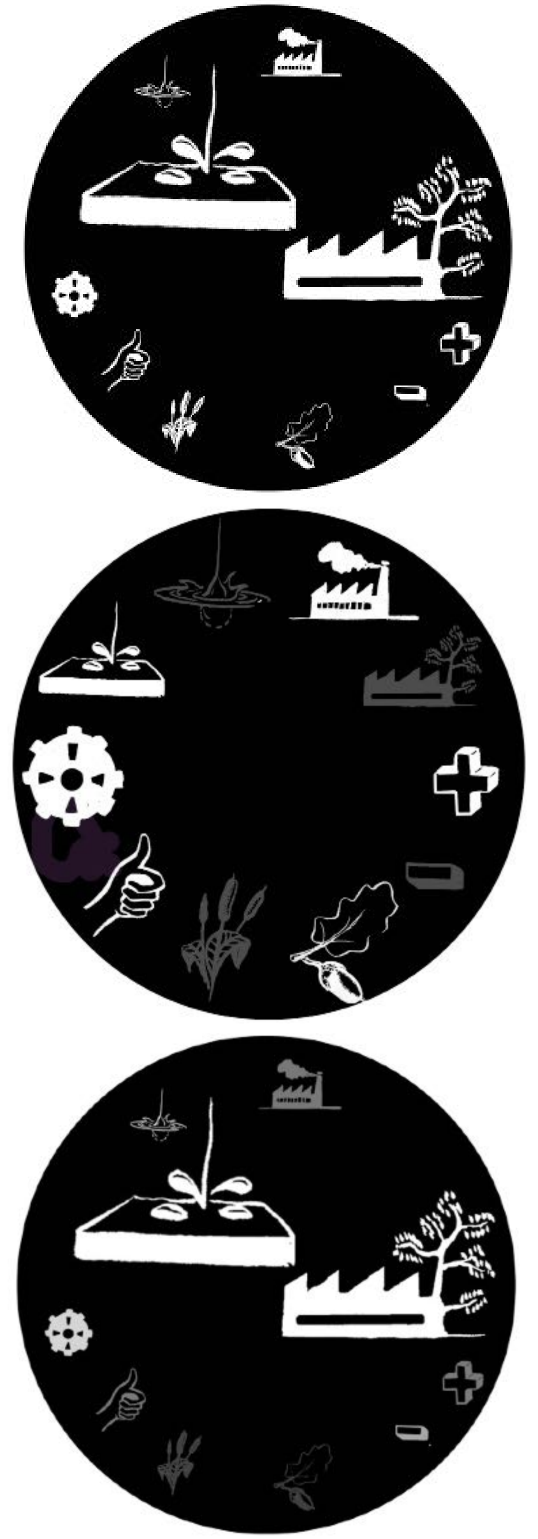

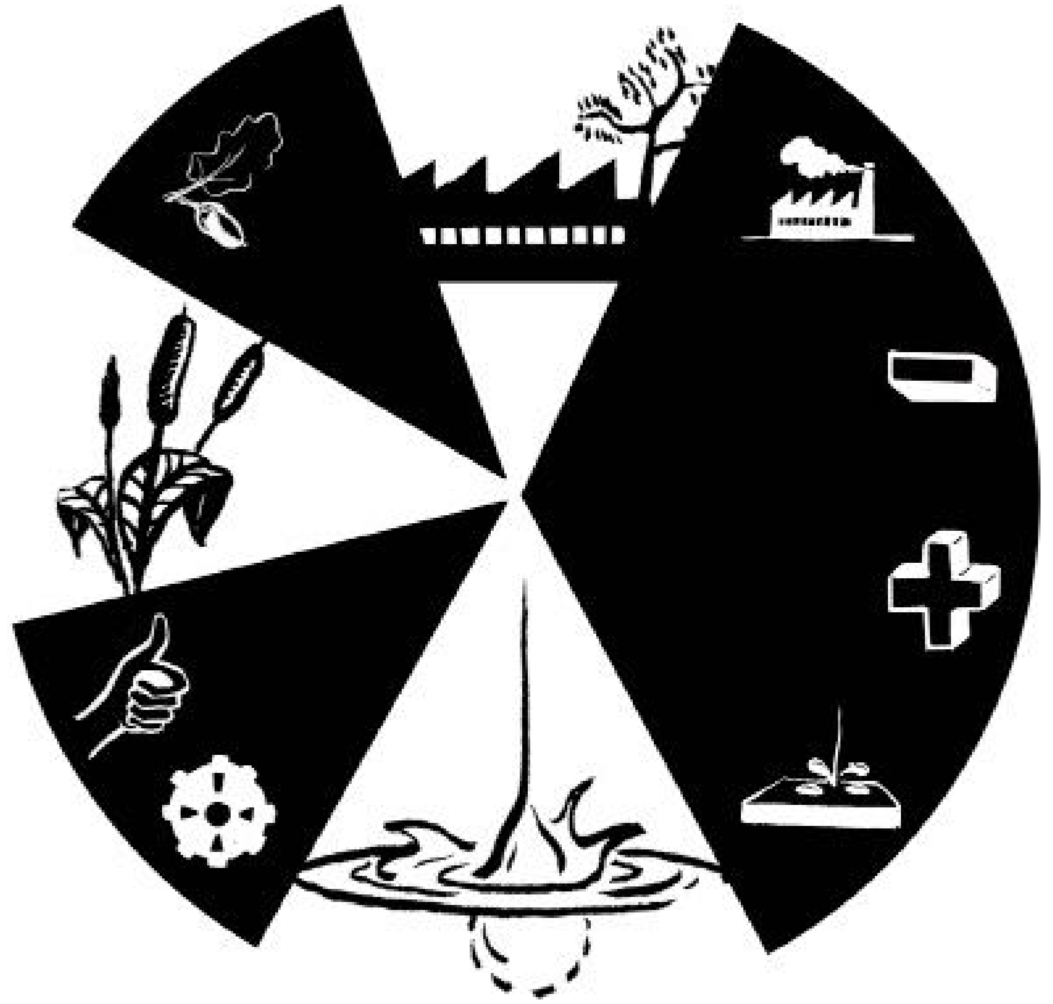

Atome hydrophile, bon pour la planète et assez souple. Ce pourrais être un atome qui entre dans la composition de l'eau.

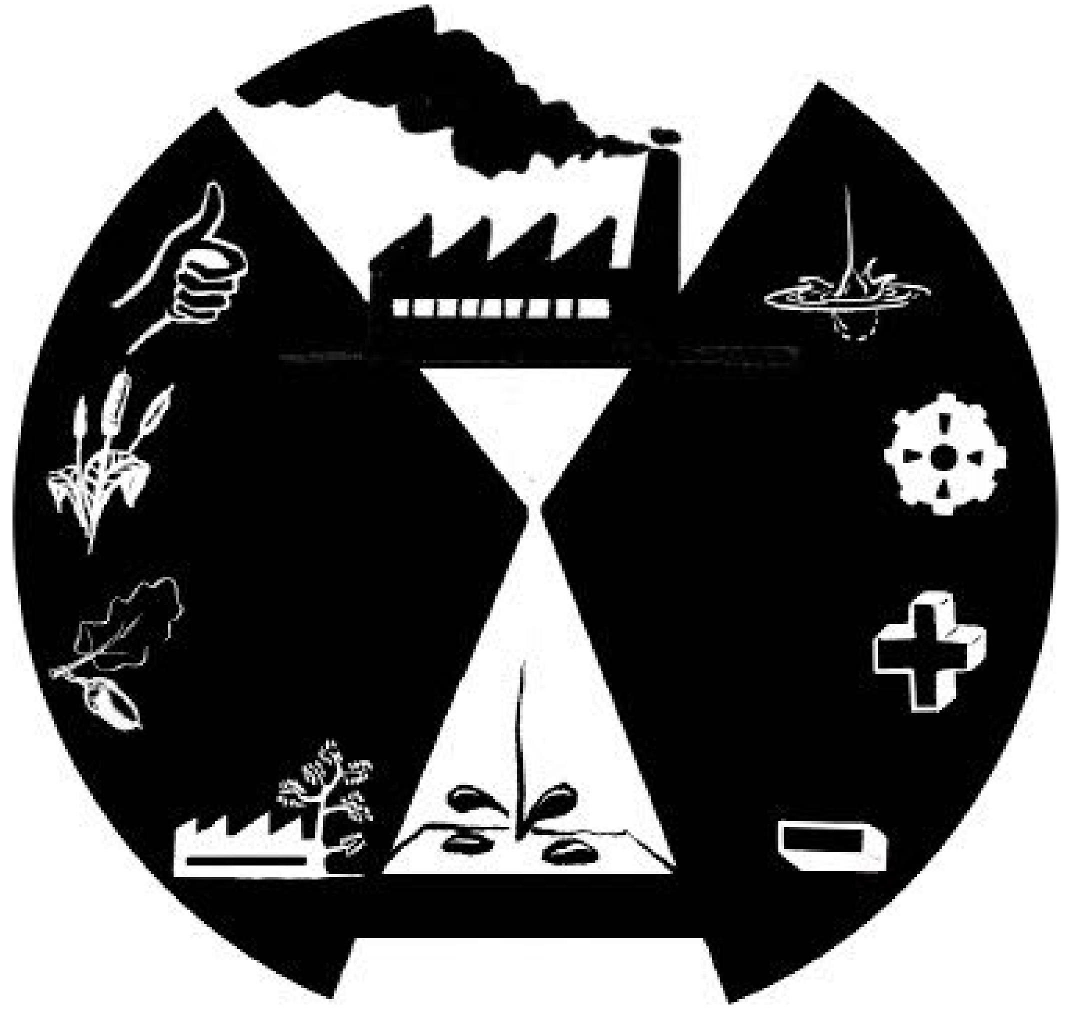

Atome hydrophobe et nocif pour l'environement.
Cela pourrais être un atome d'huile de vidance dans une prairie.

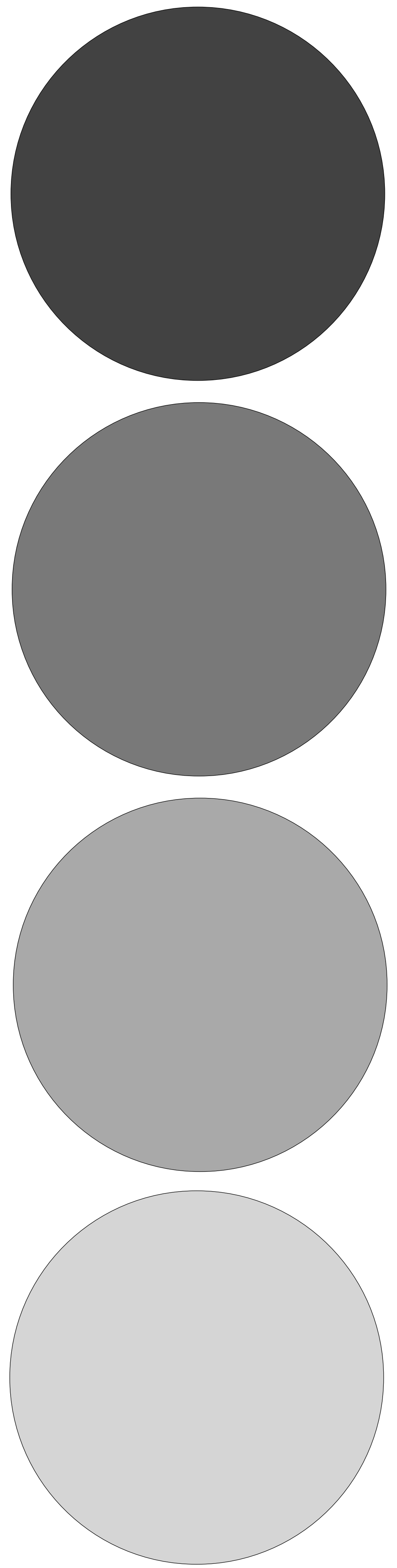

Lit-sphère

Les cercles chromatiques

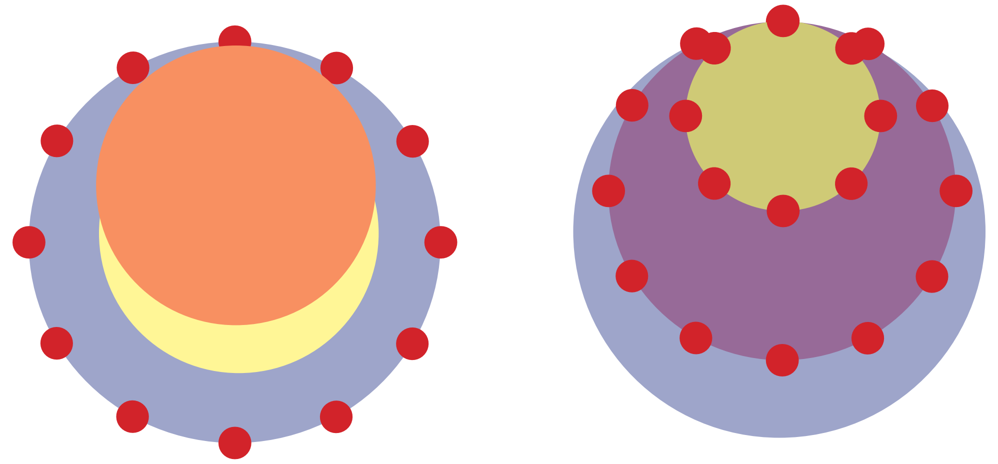

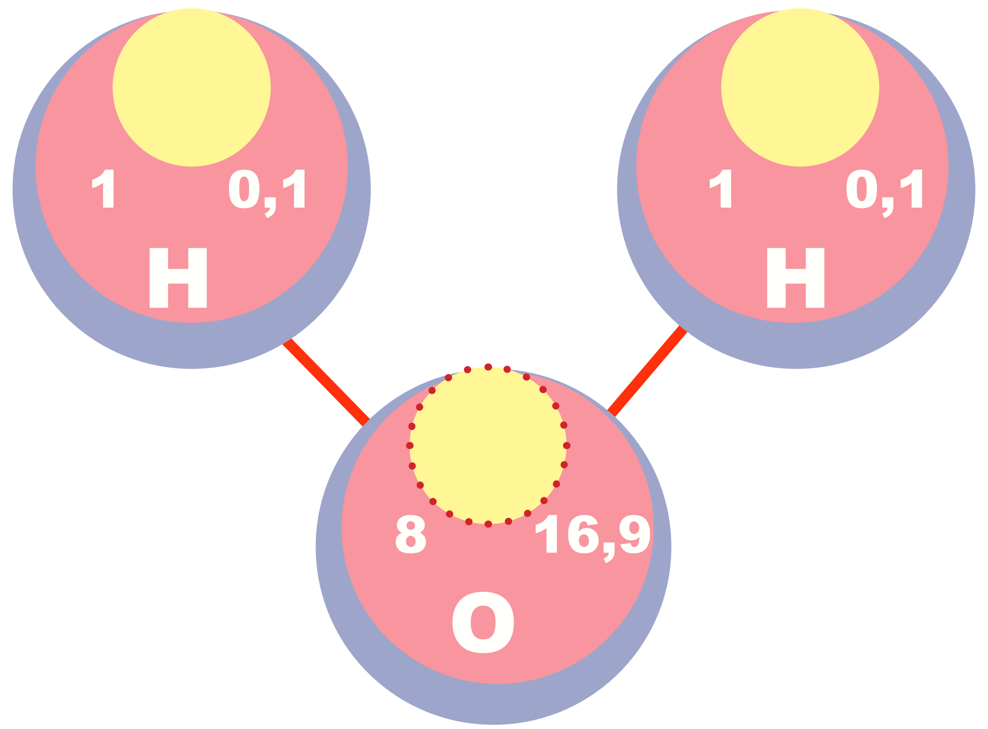

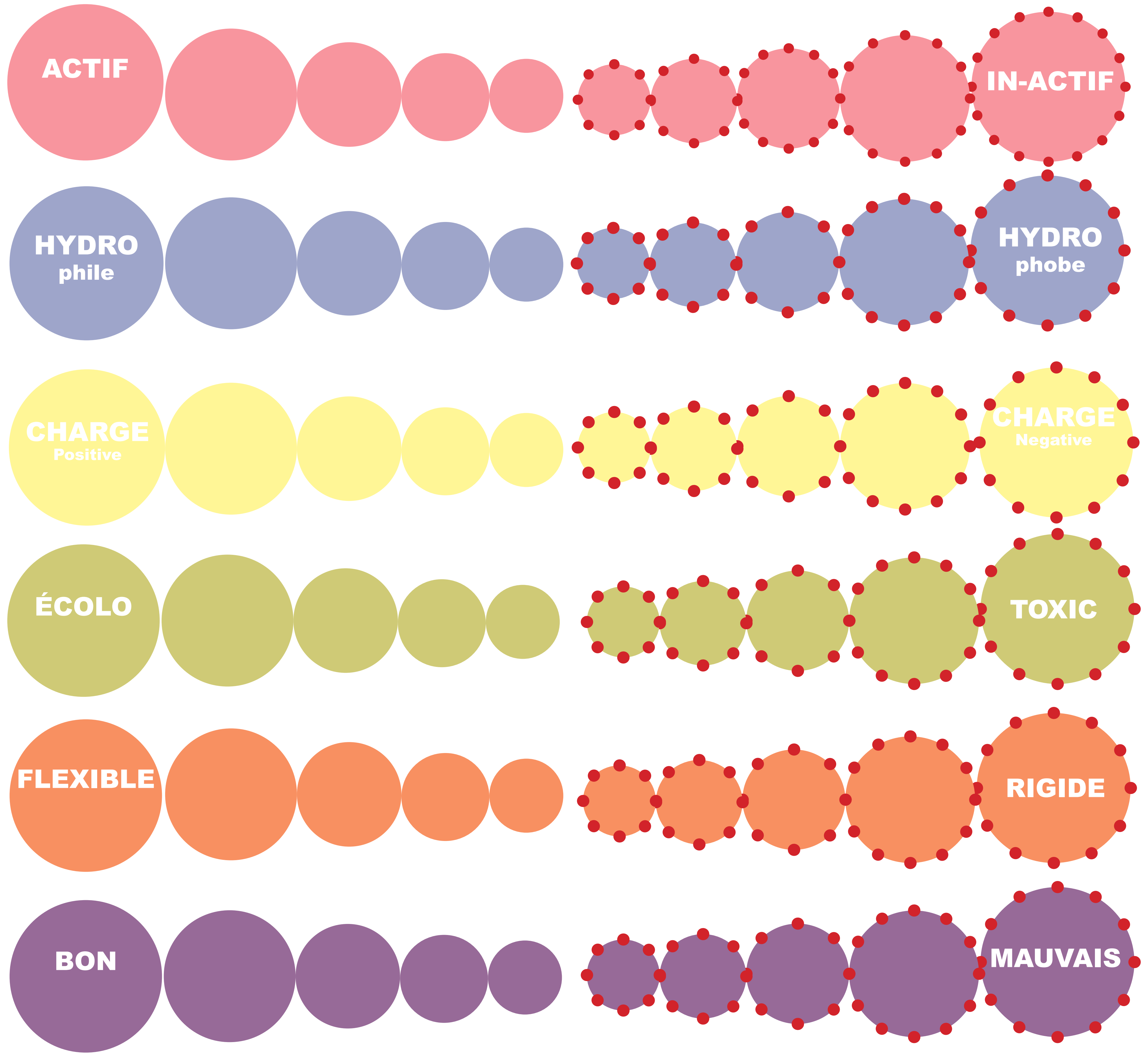

QUENTIN LEBEAU

LIT-SPHÈRE

RECHERCHES

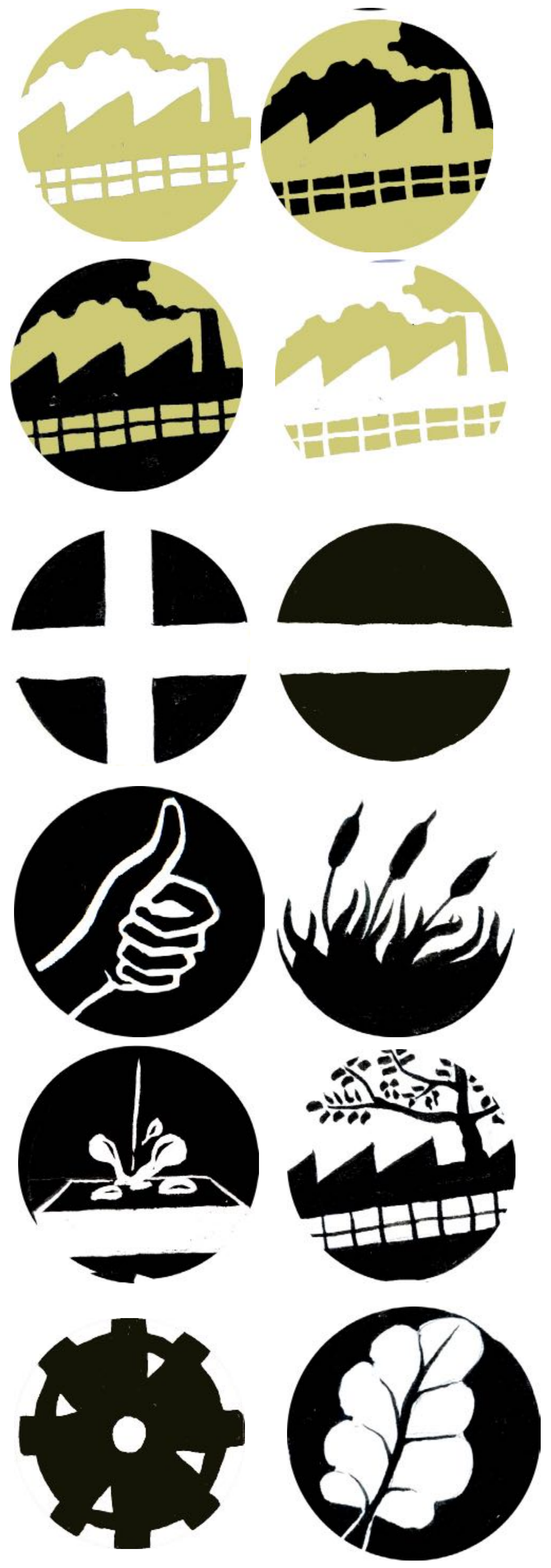

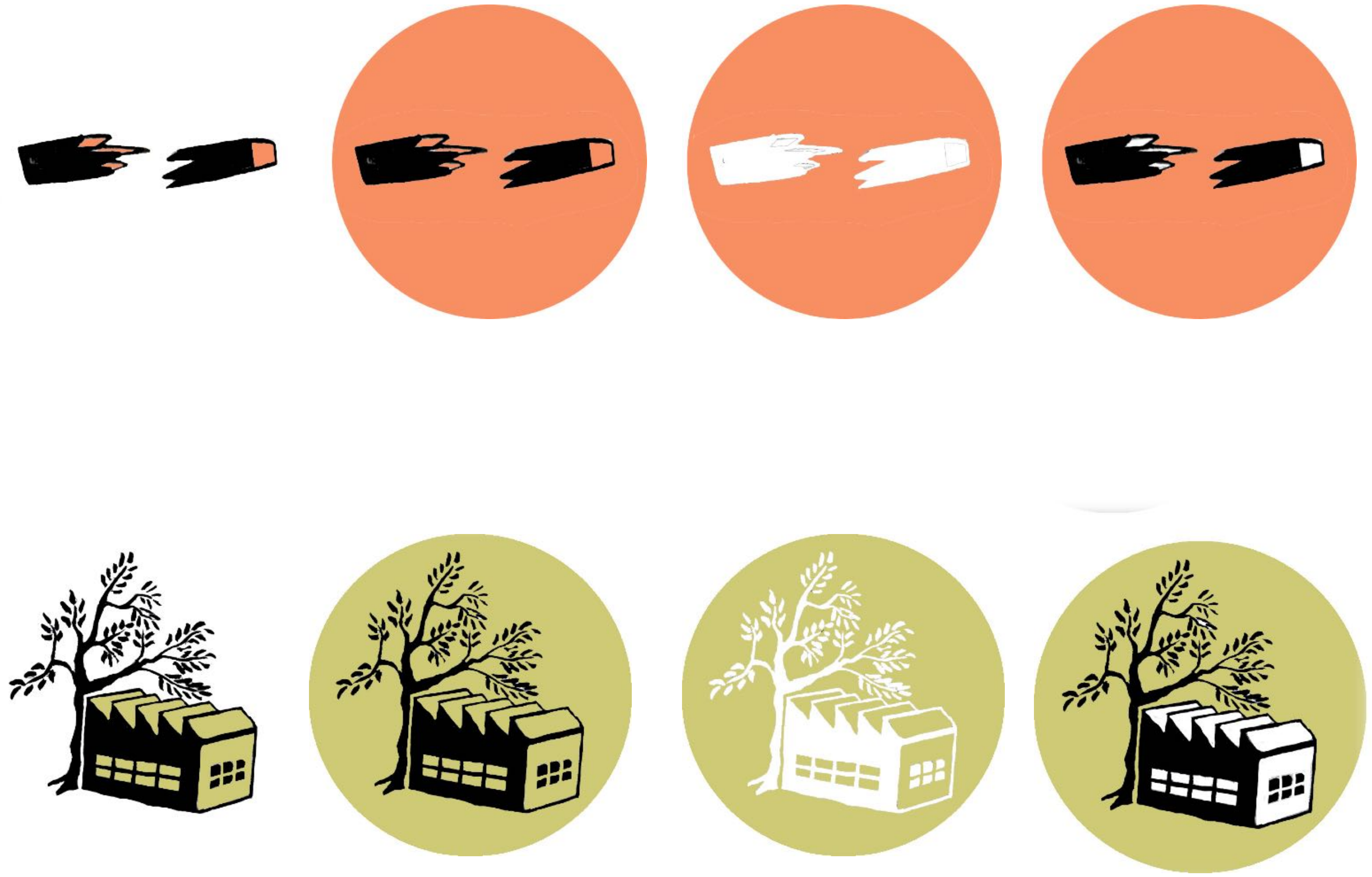

LIT-SPHÈRE

RECHERCHES

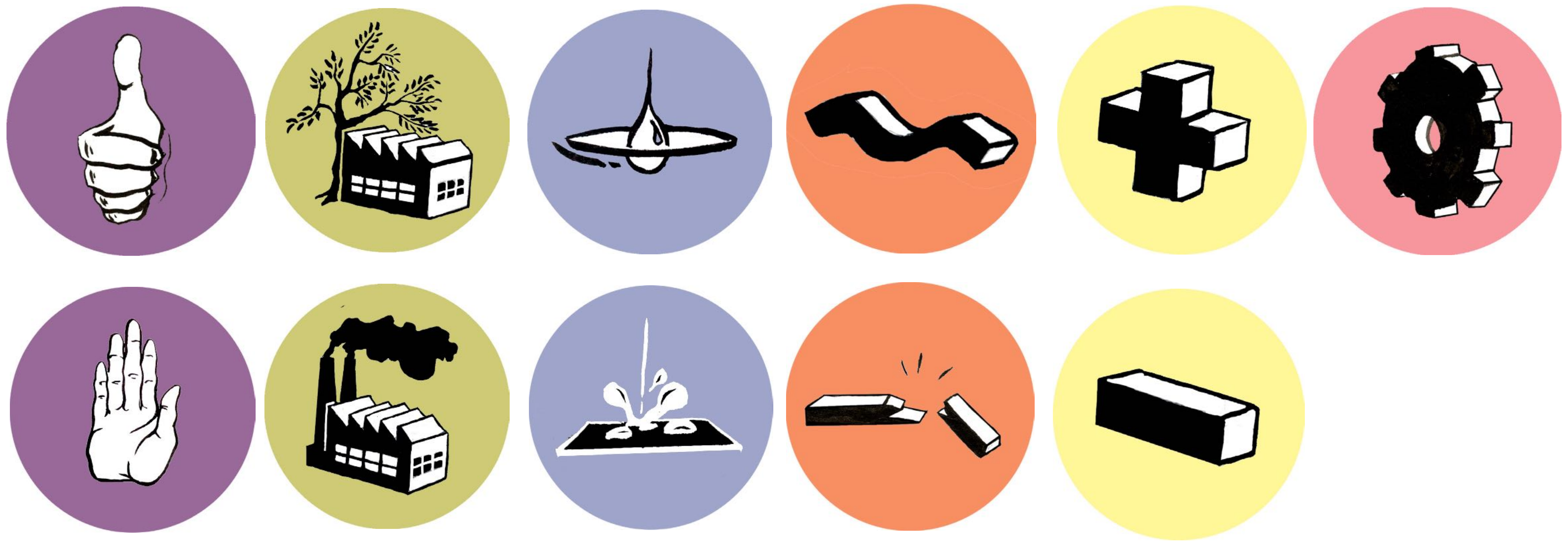

QUENTIN LEBEAU

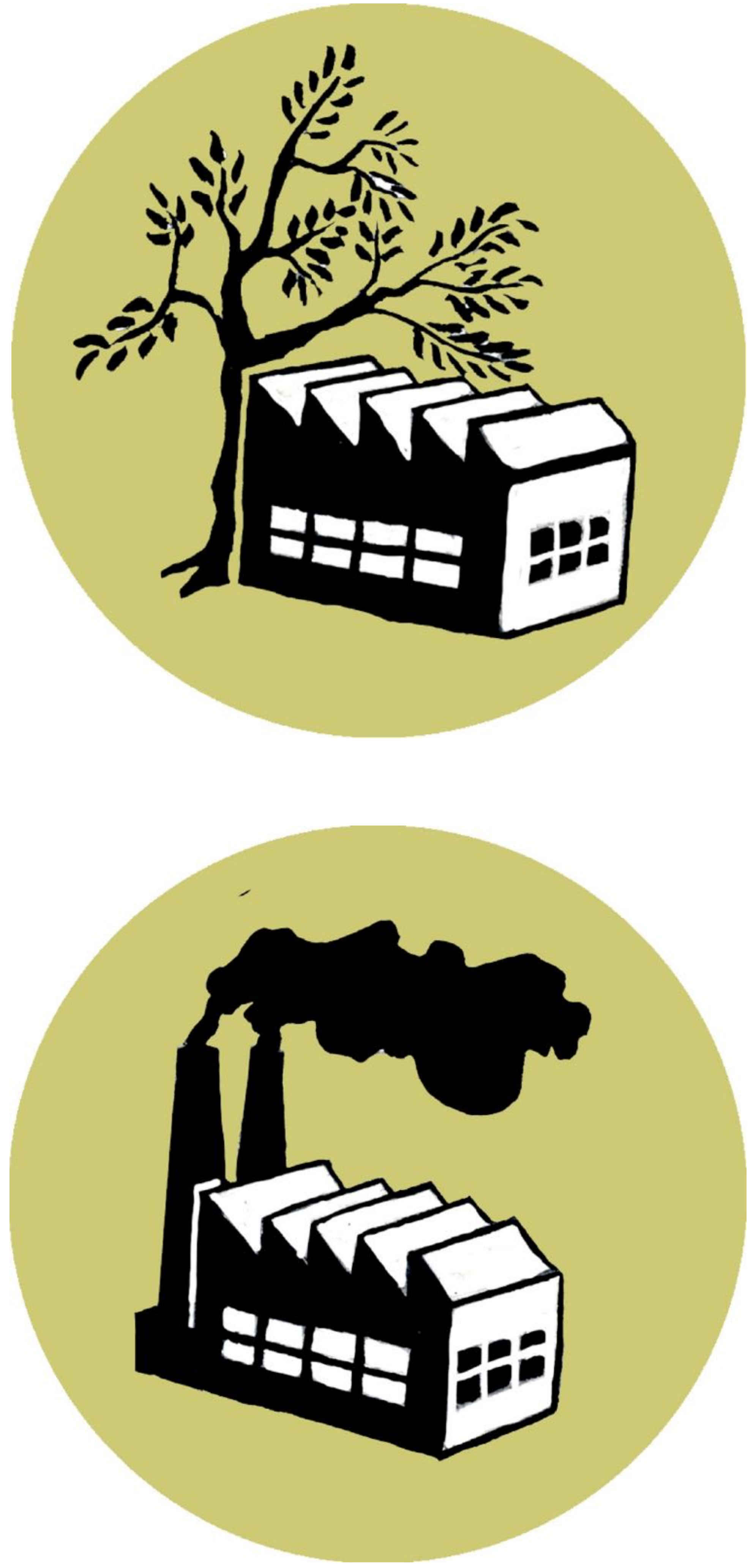

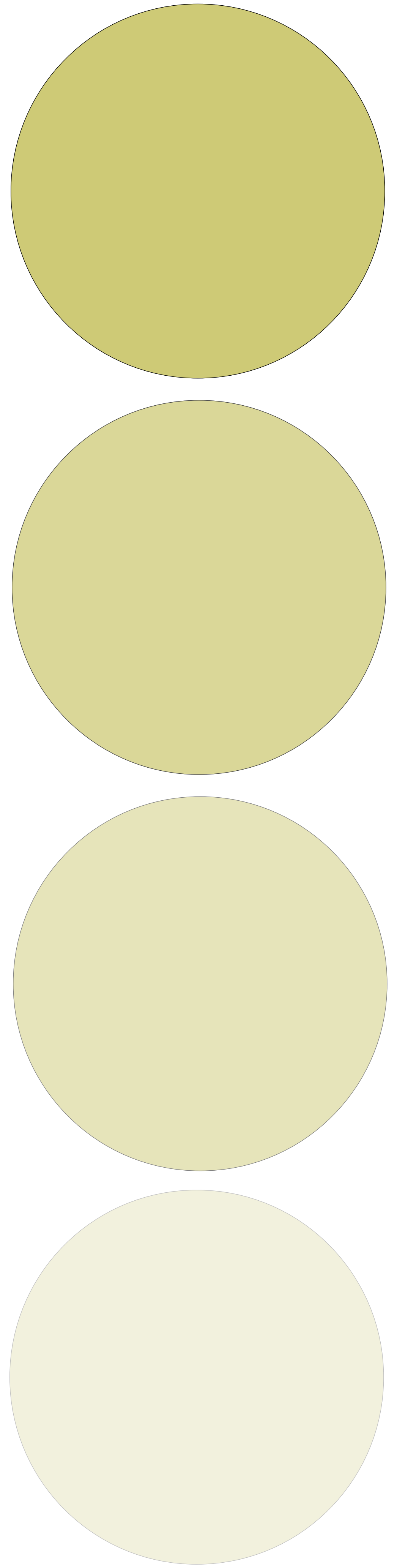

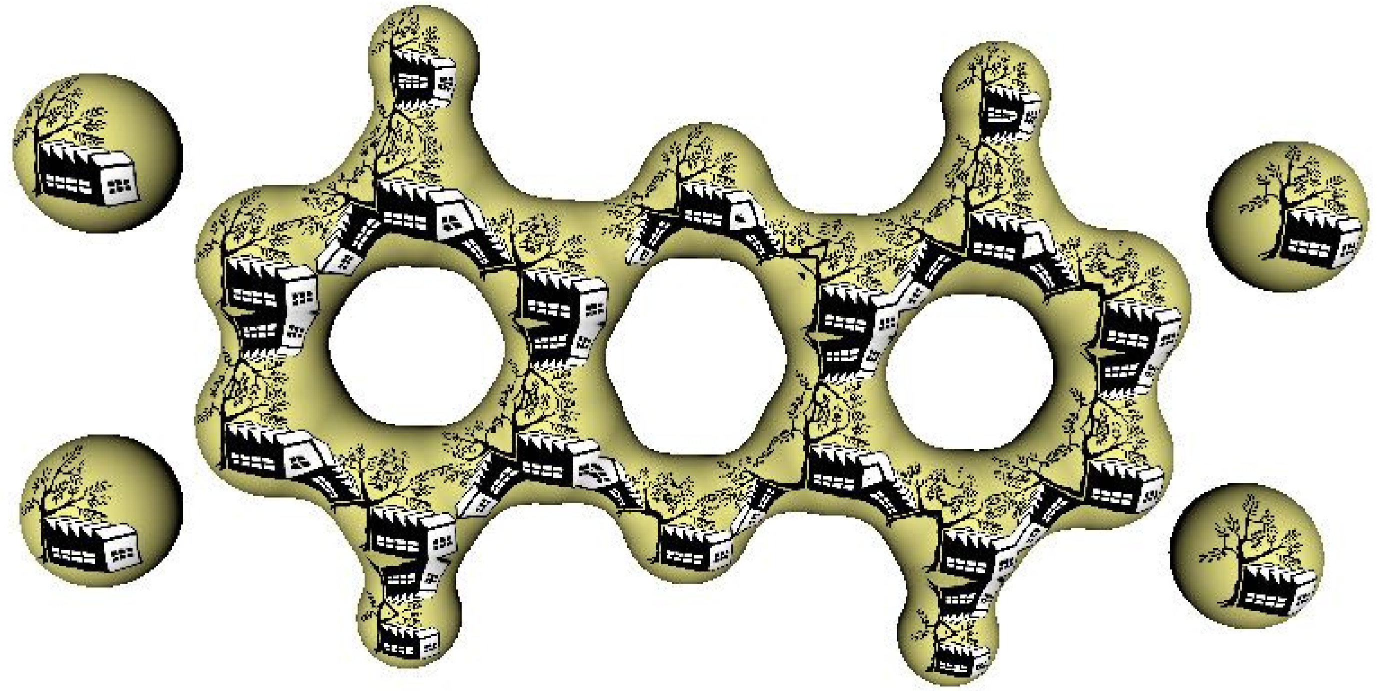

Écologique/Toxic

Lit-sphère

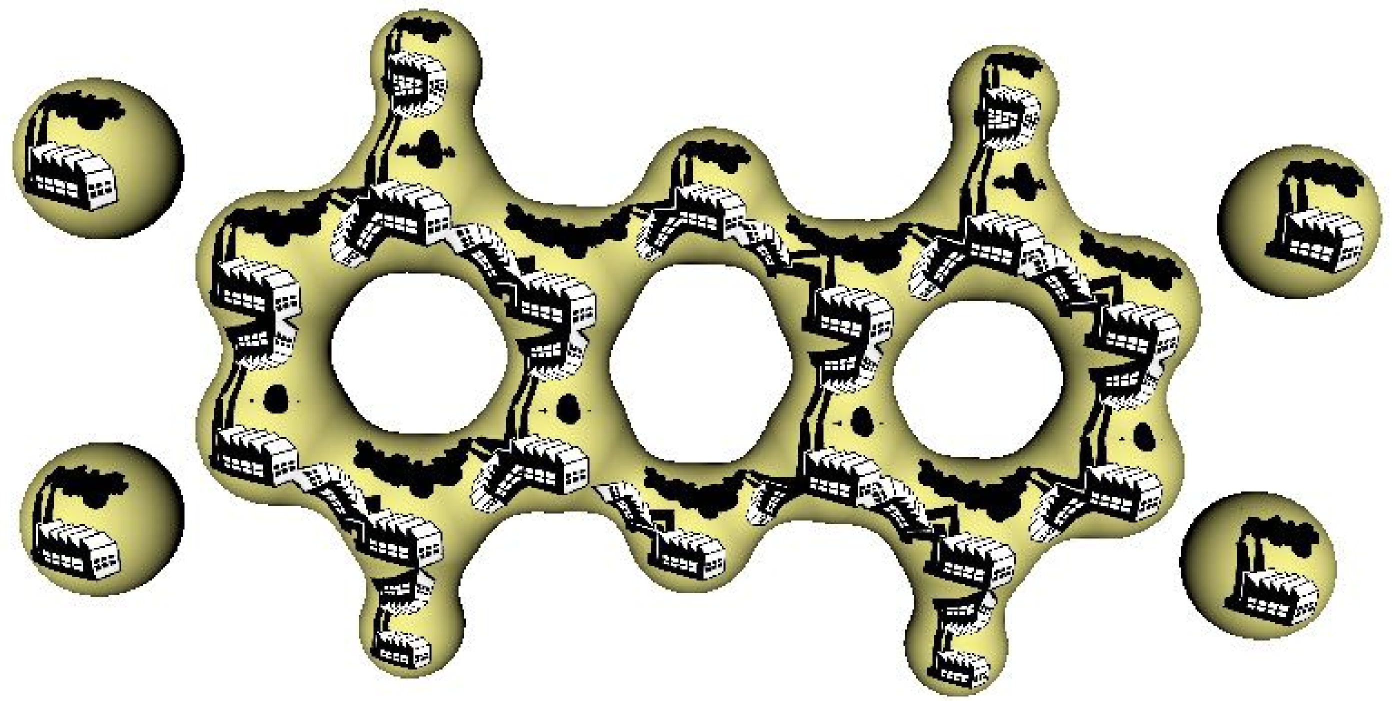

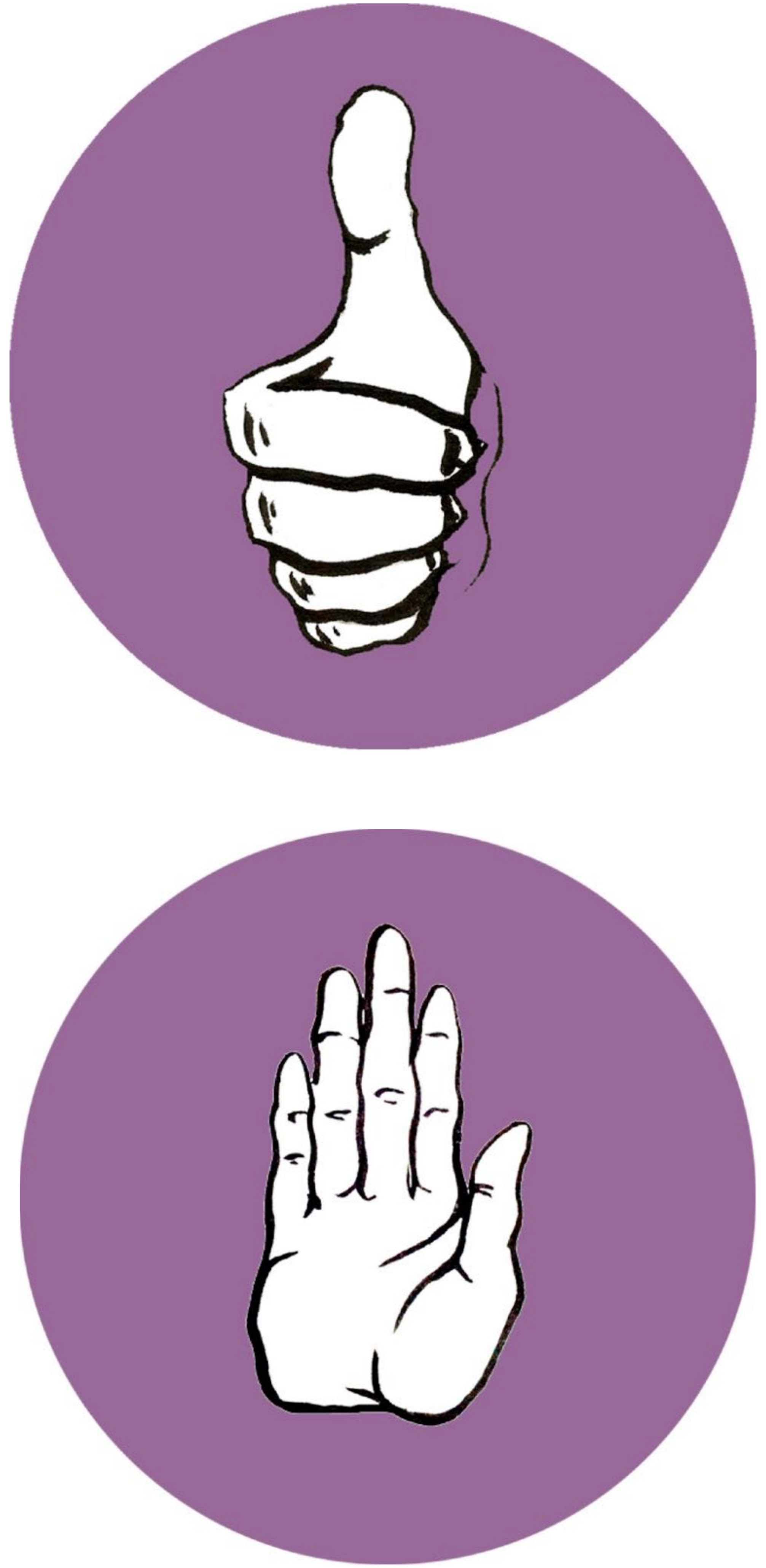

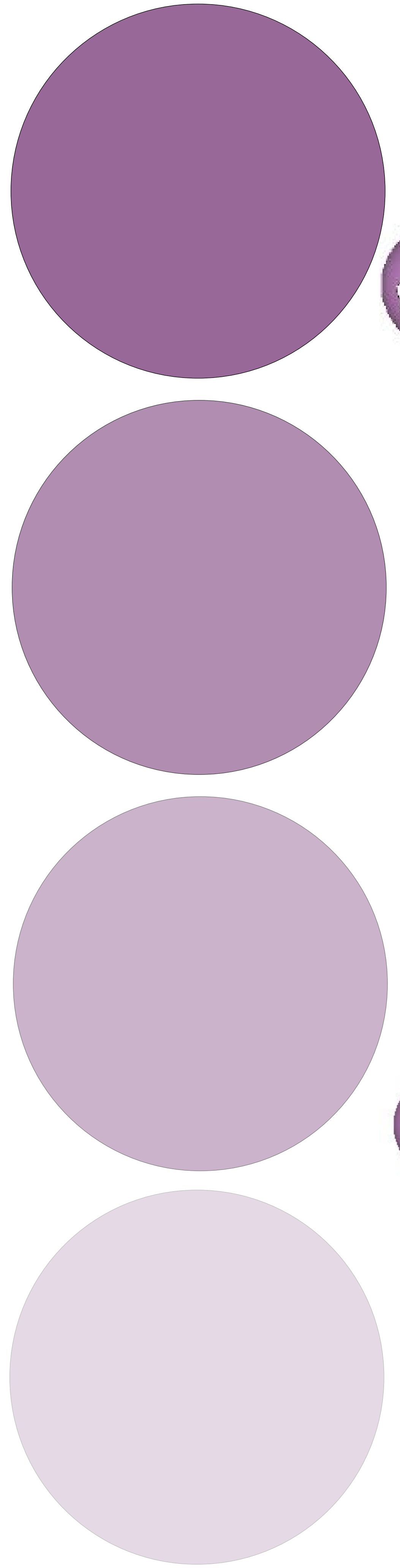

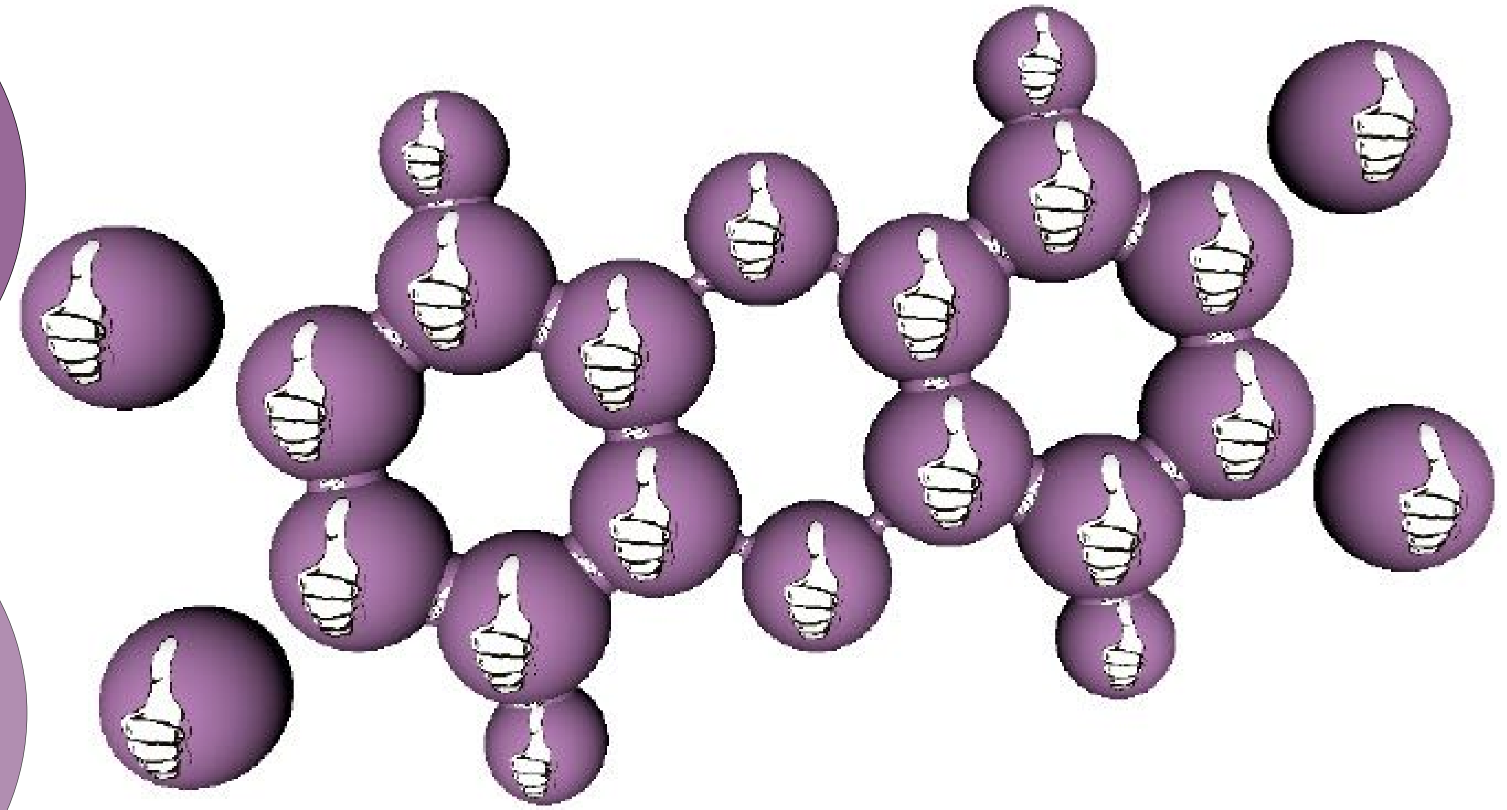

Bon/Mauvais

Lit-sphère

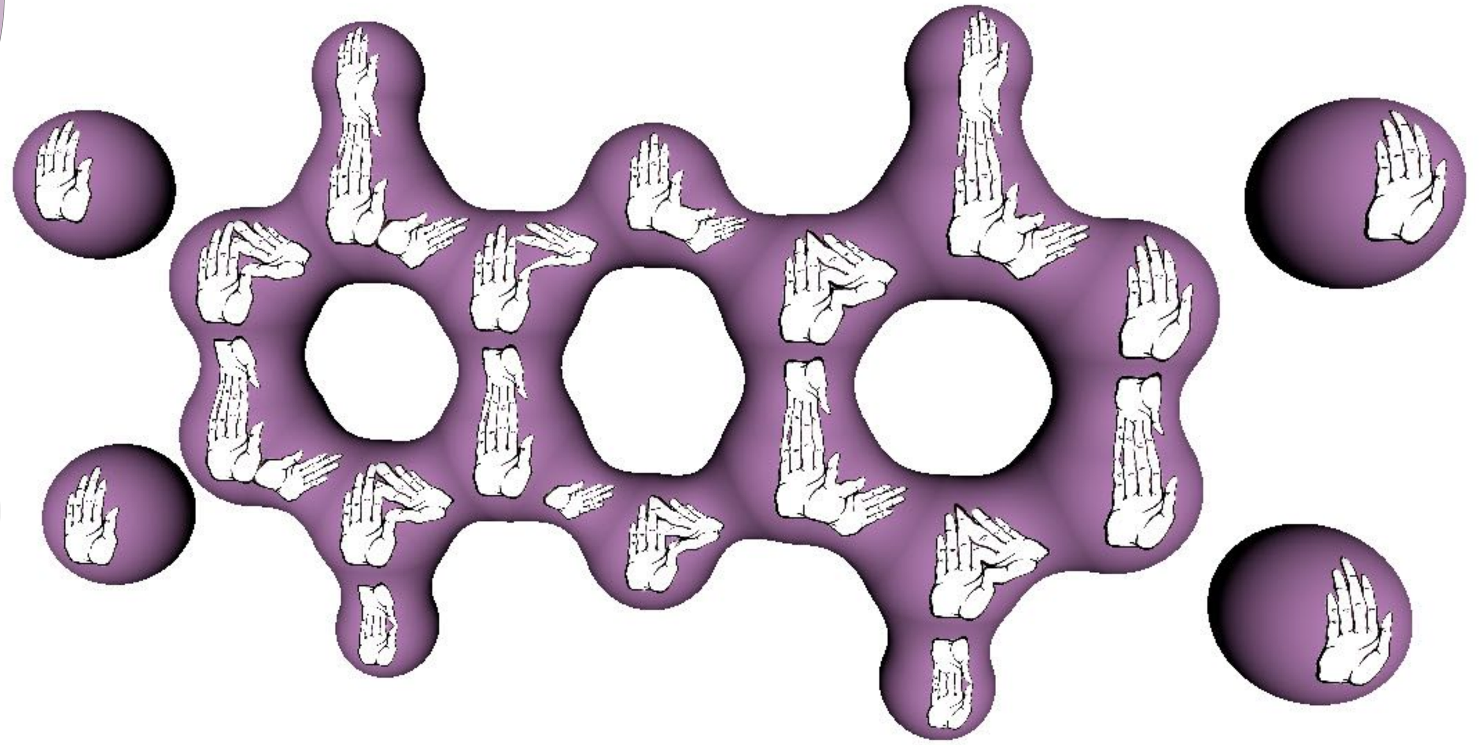

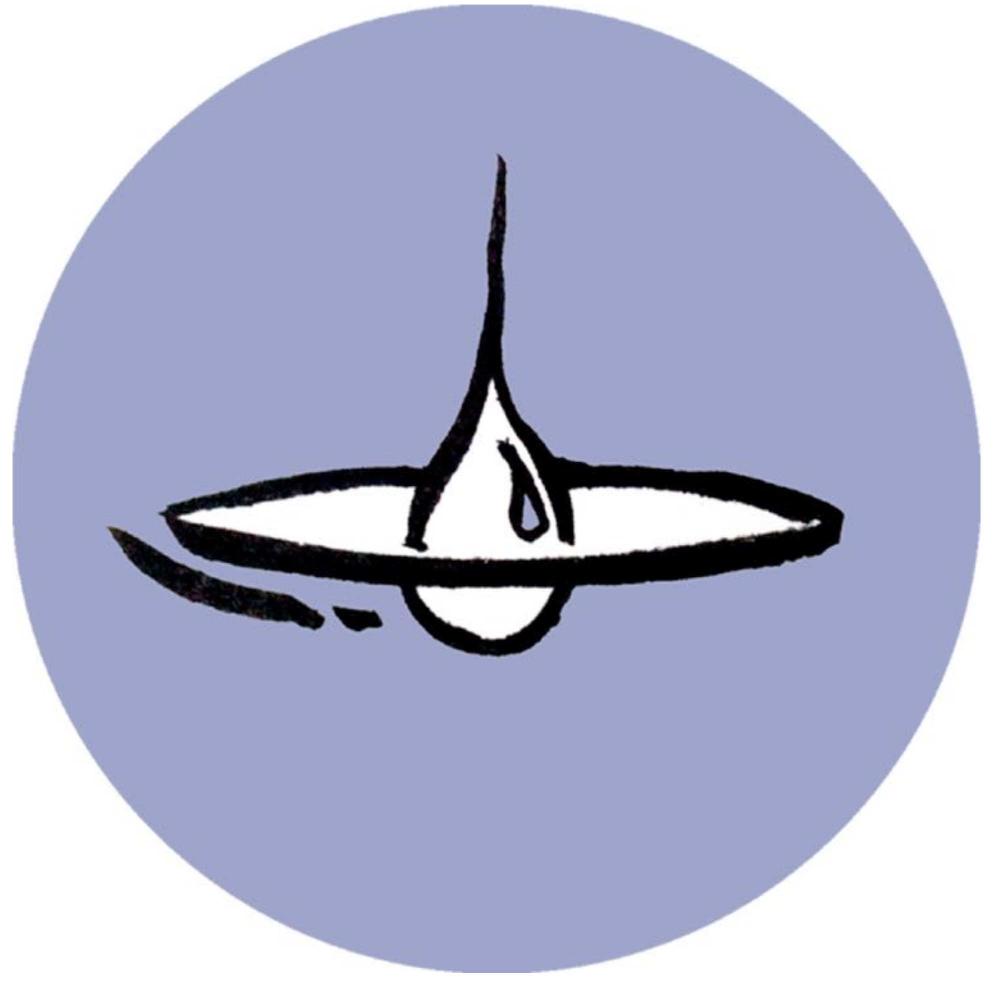

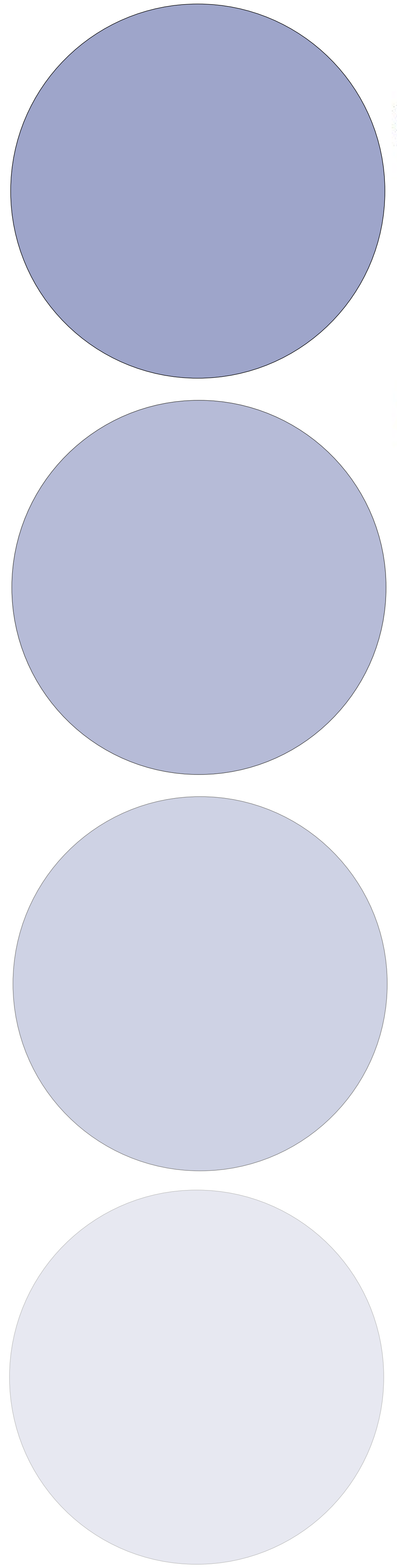

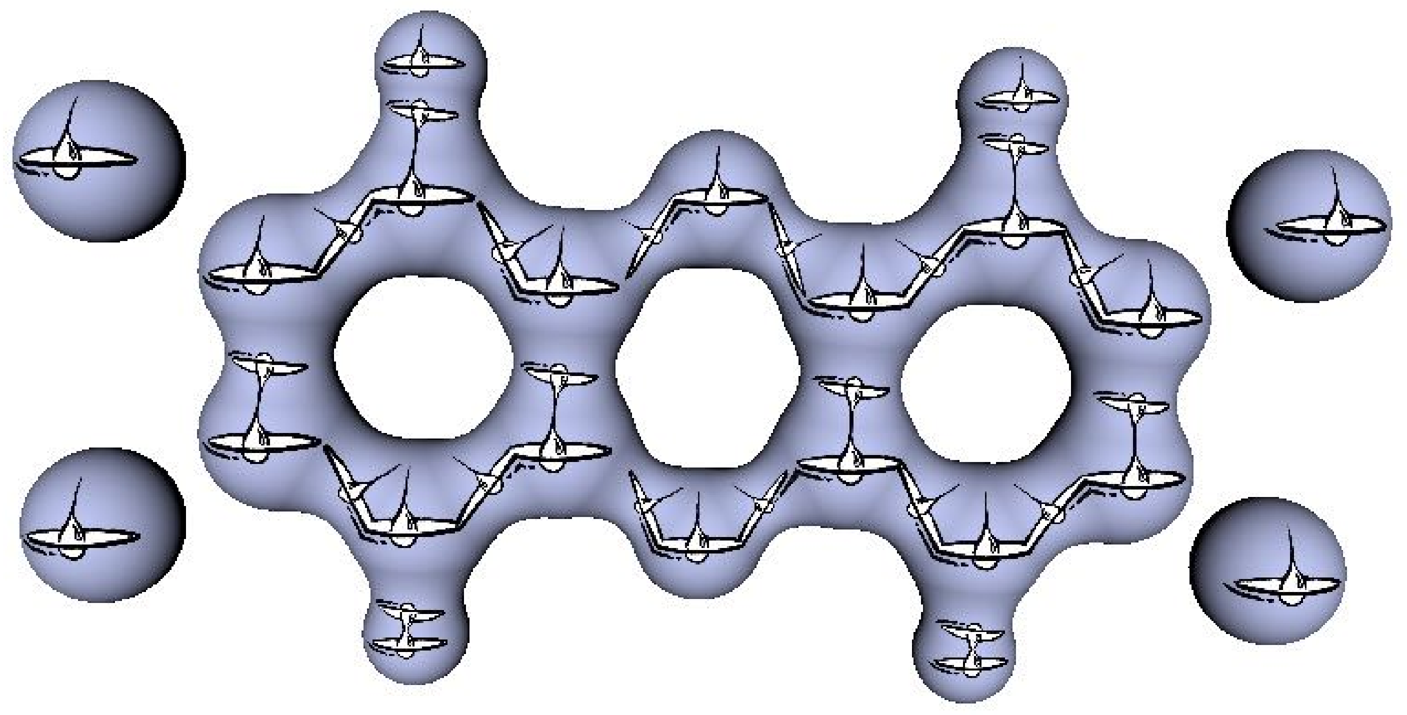

Hydrophile/phobe

Lit-sphère

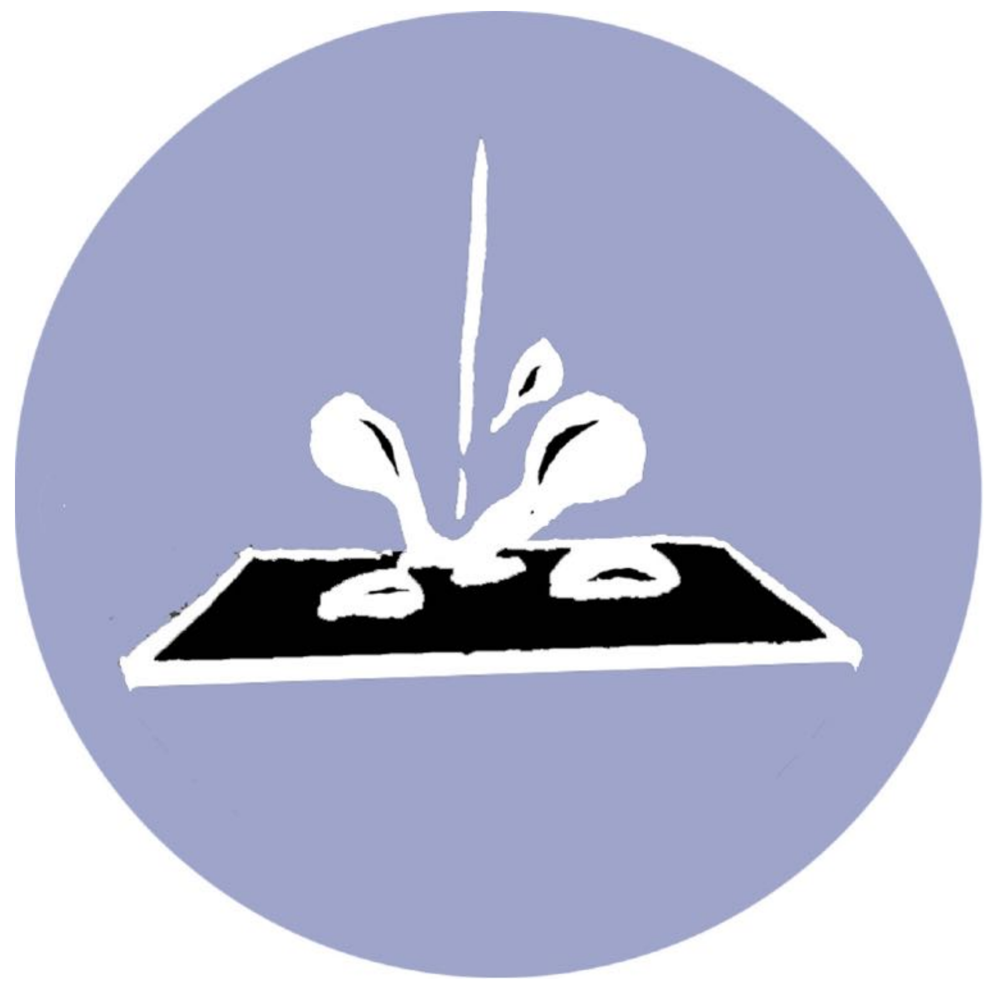

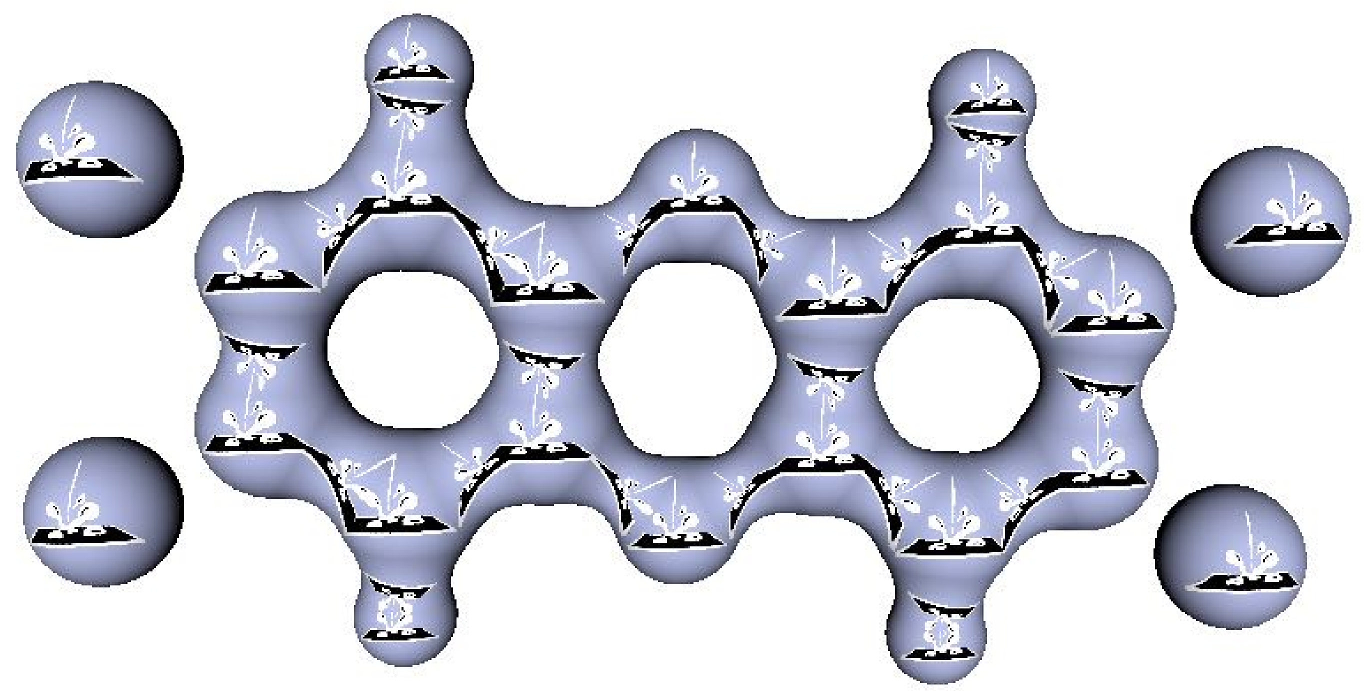

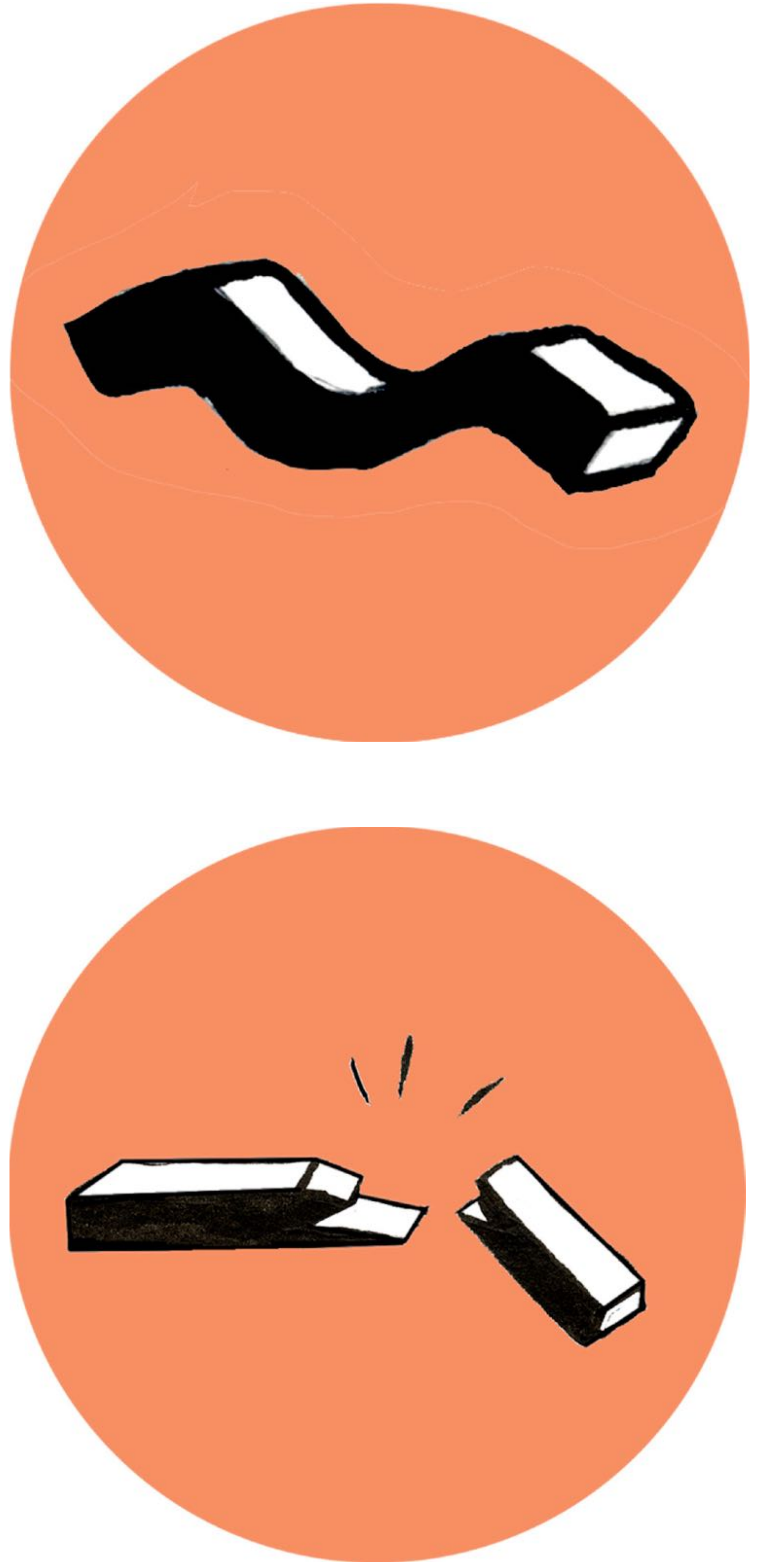

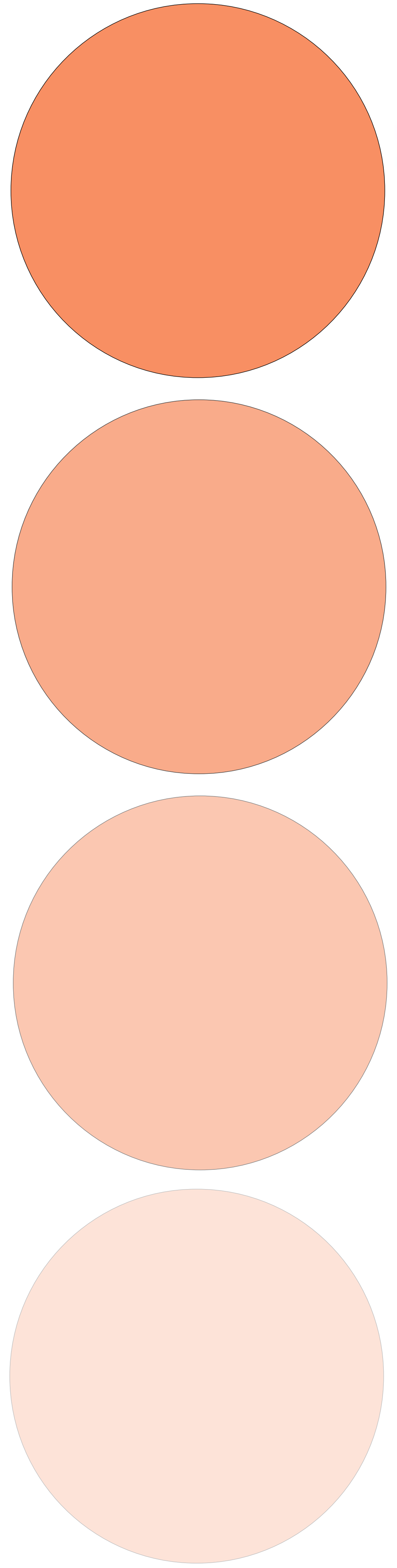

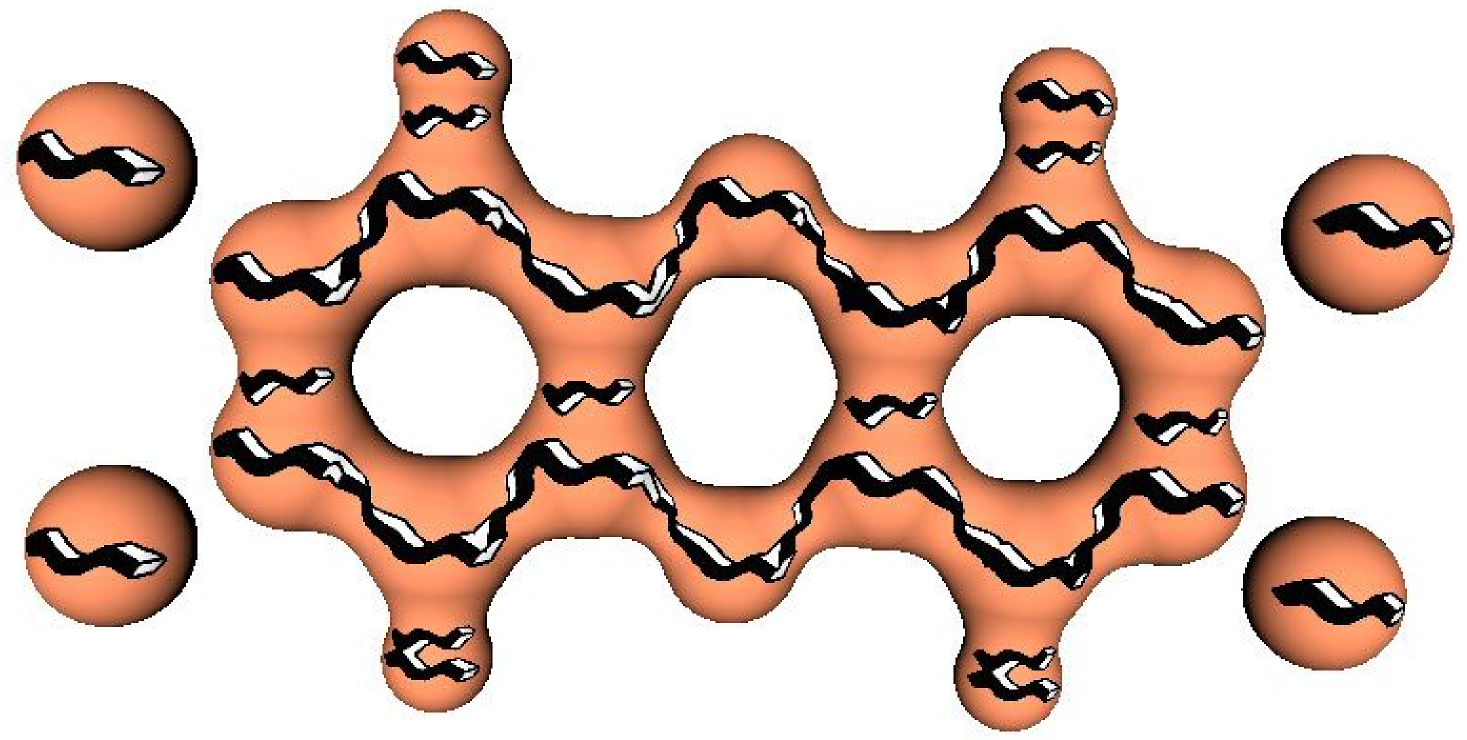

Flexible/Rigide

Lit-sphère

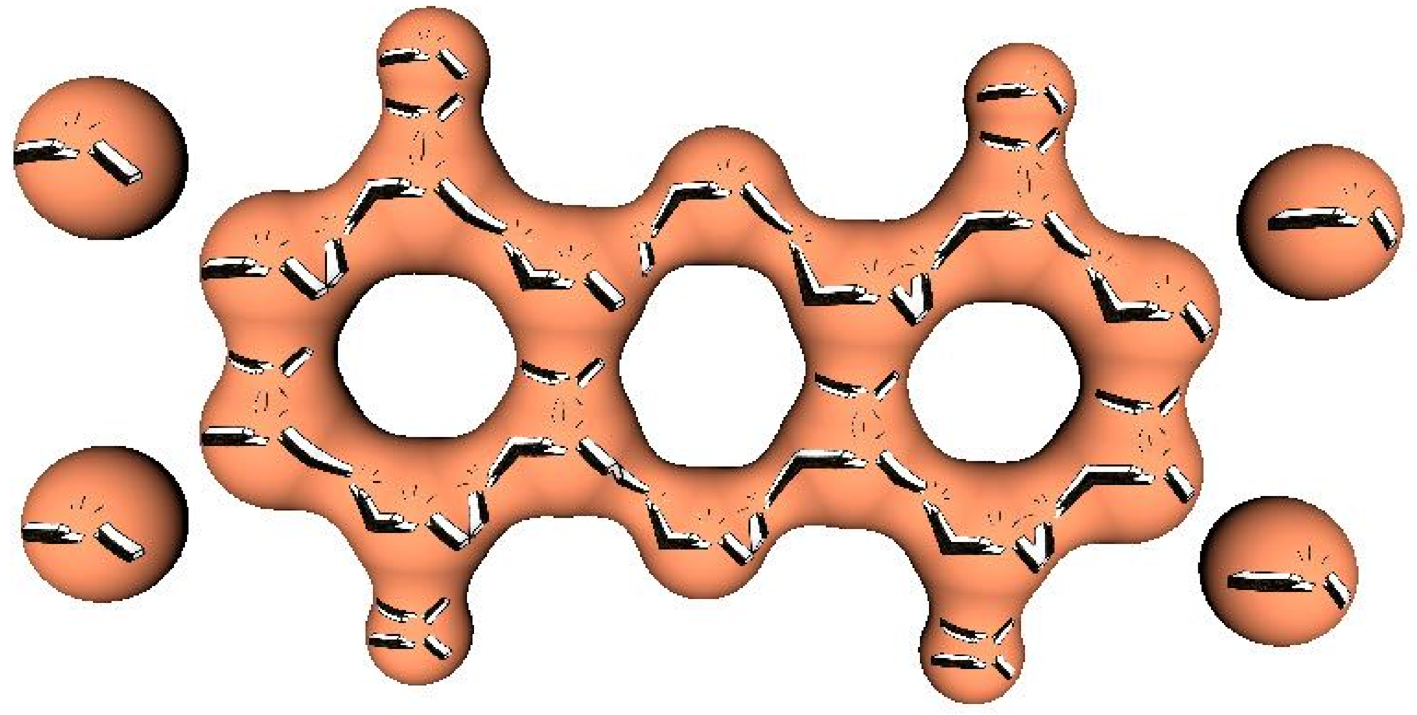

QUENTIN LEBEAU

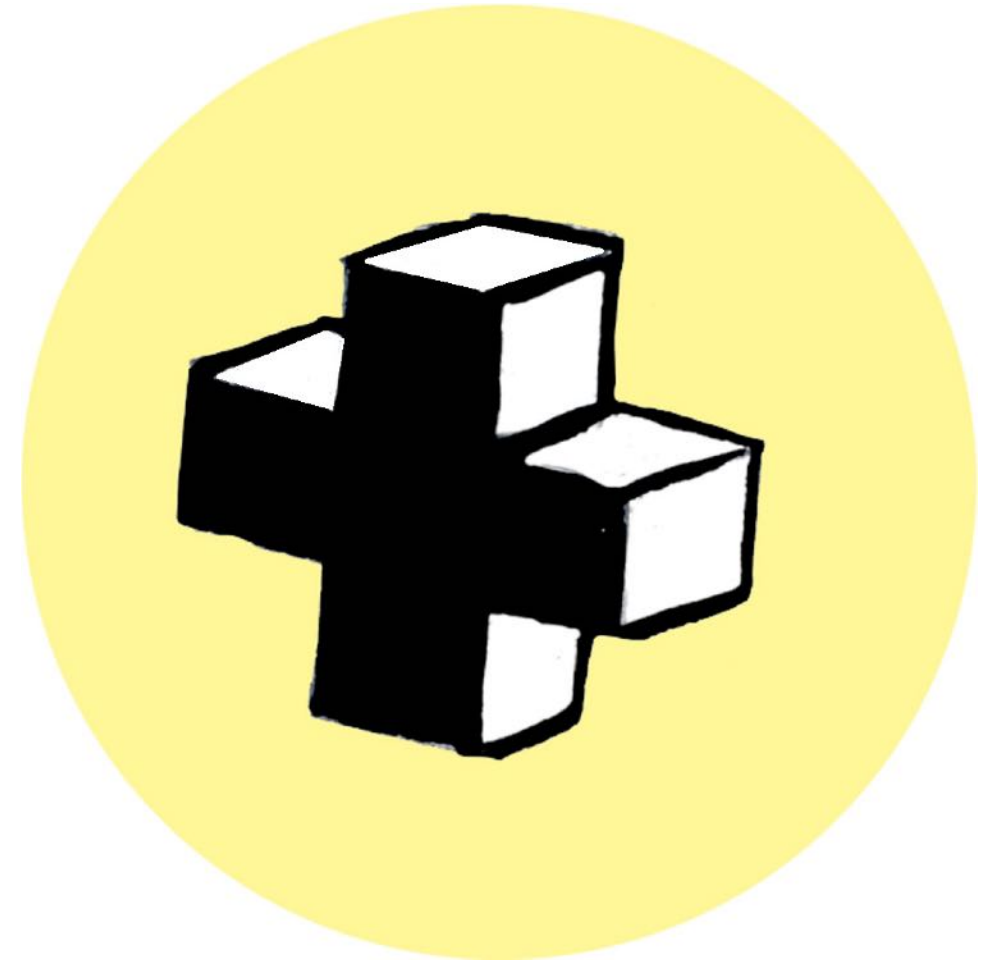

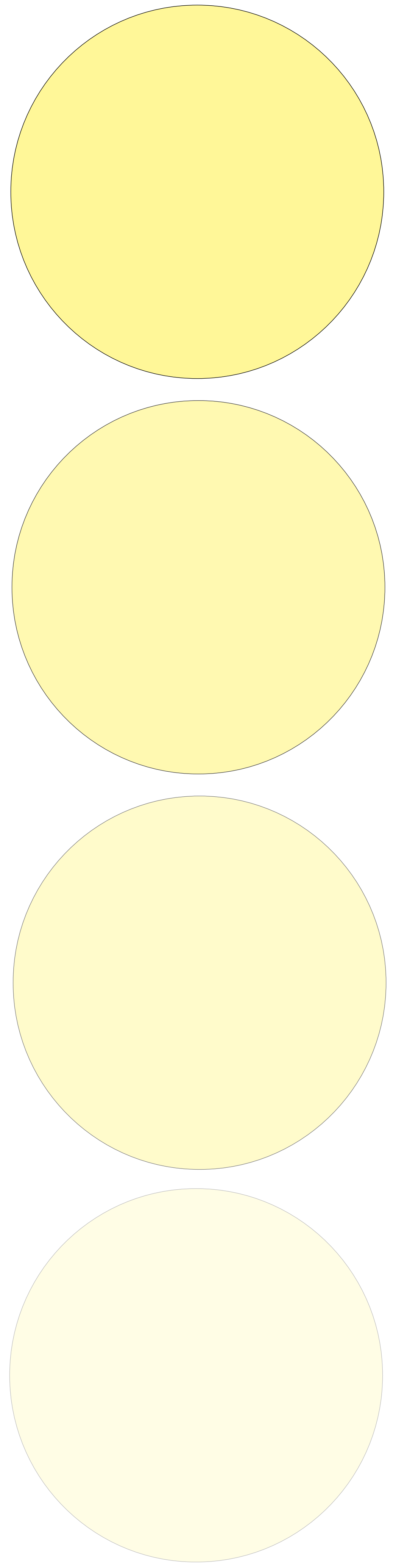

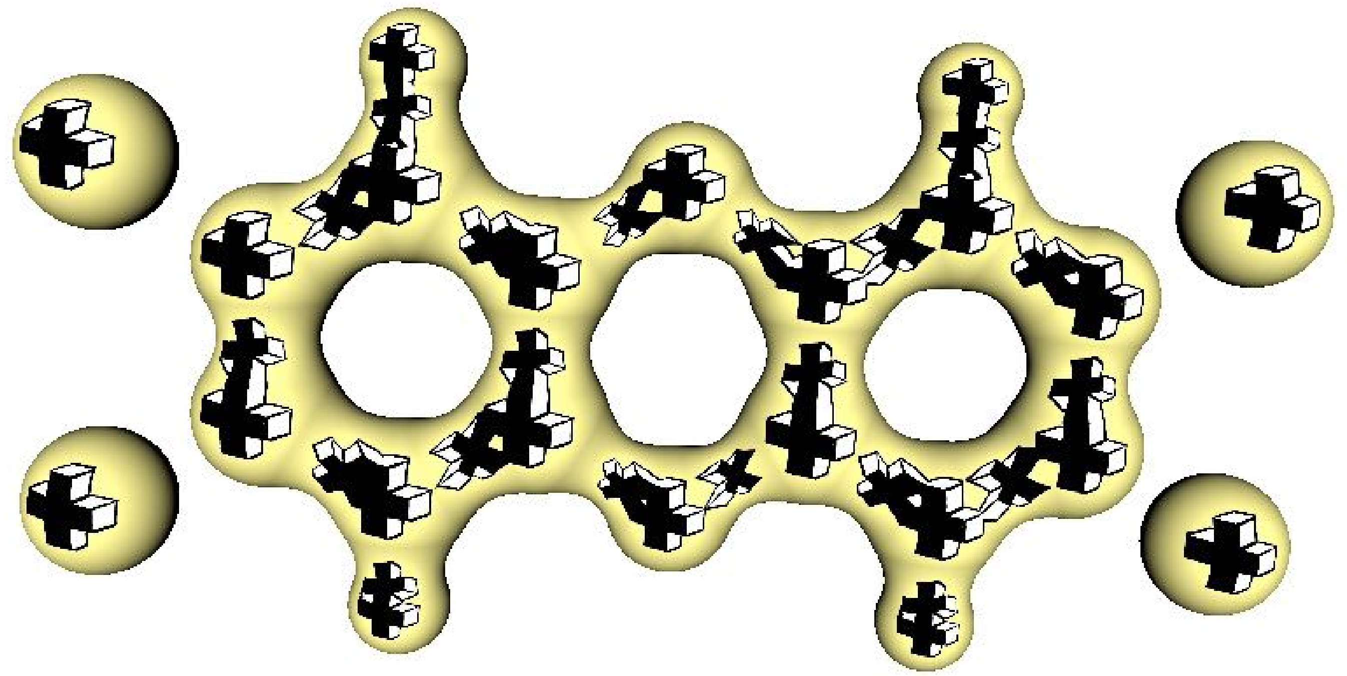

Charge +/-

Lit-sphère

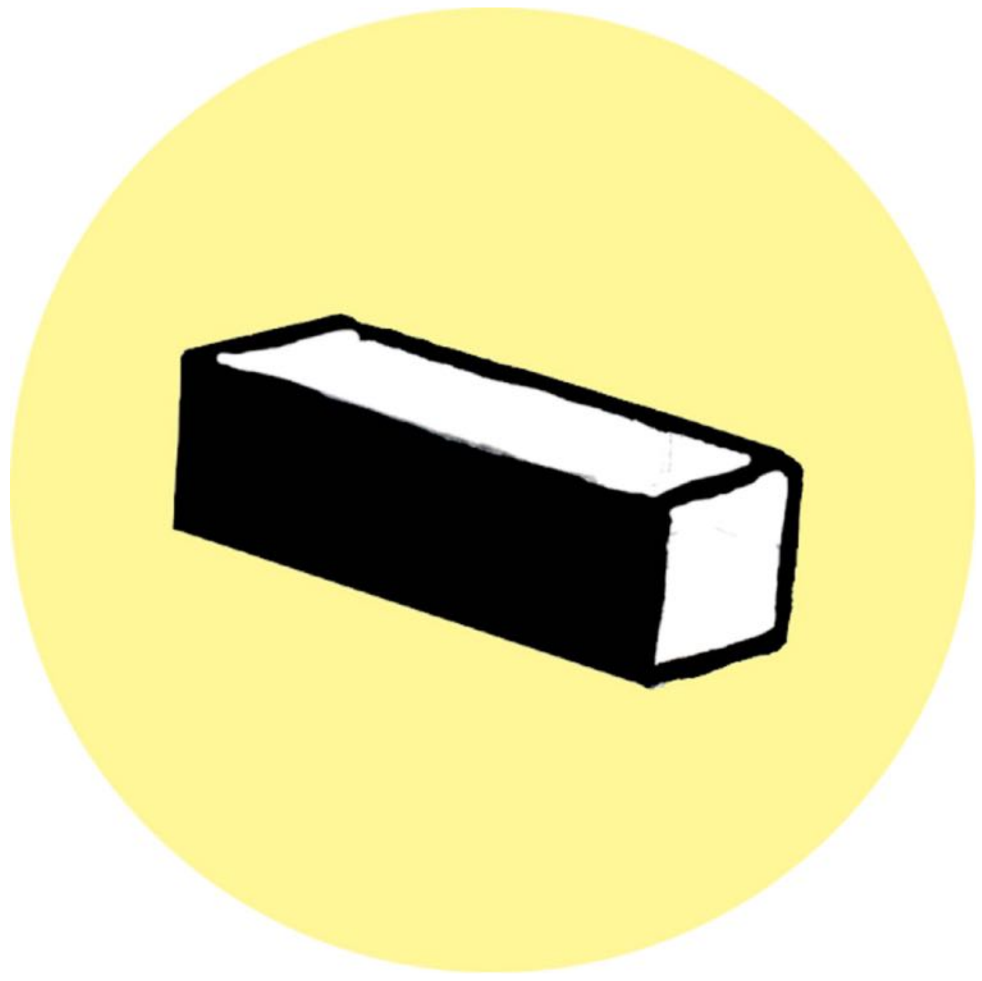

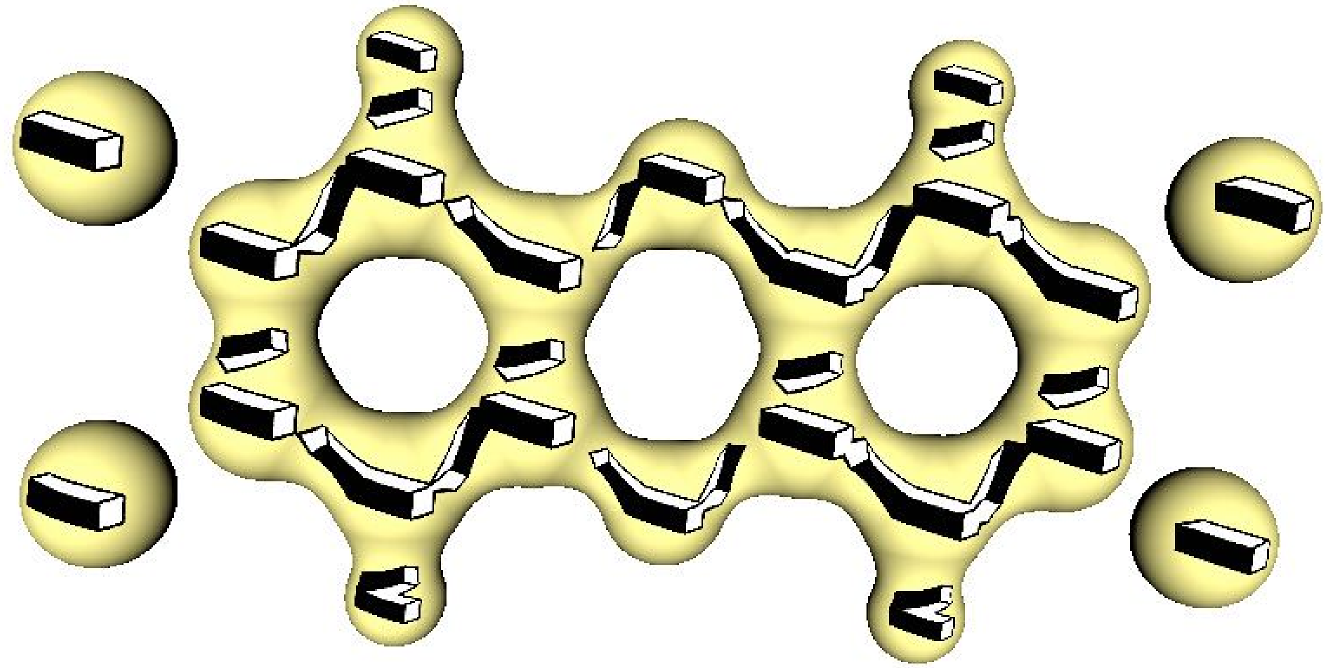

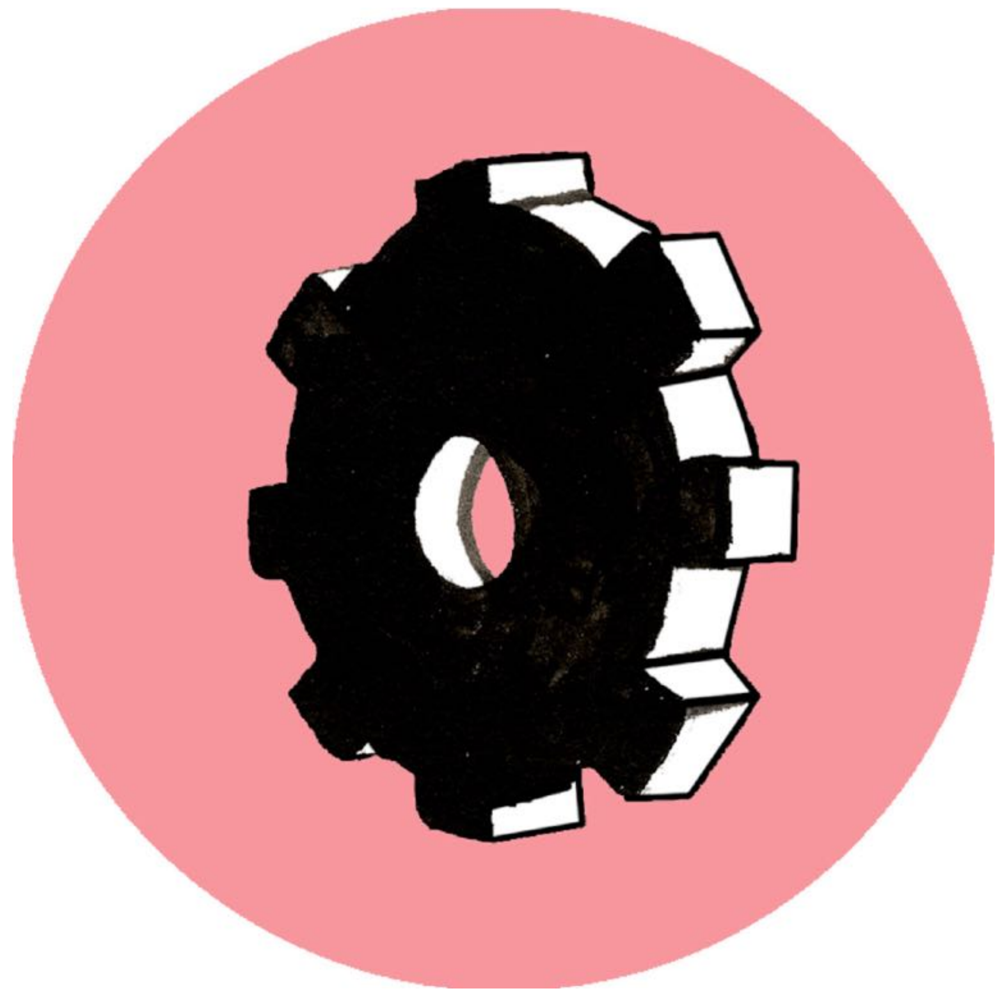

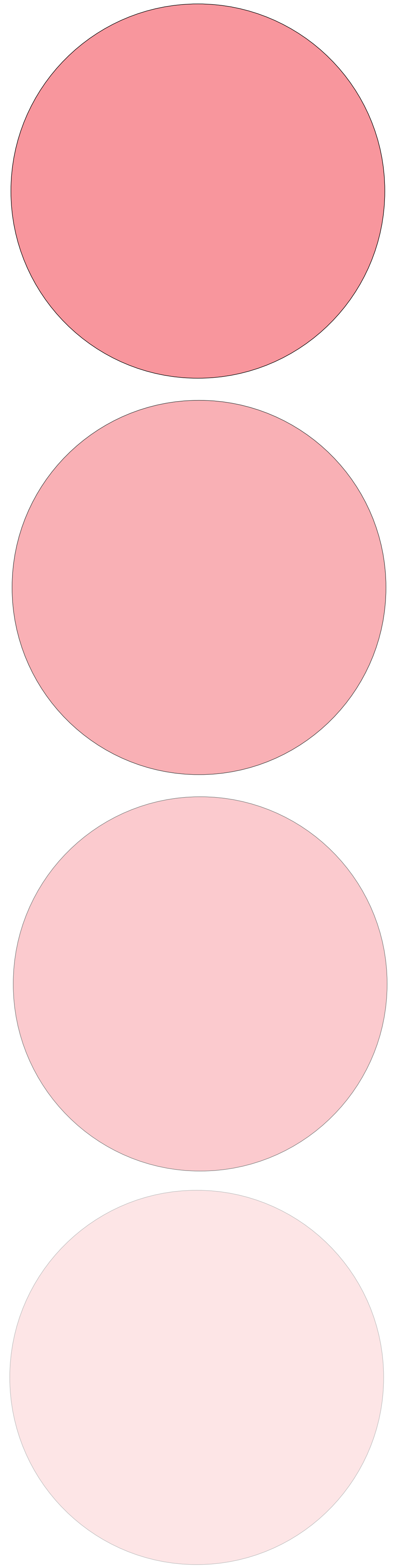

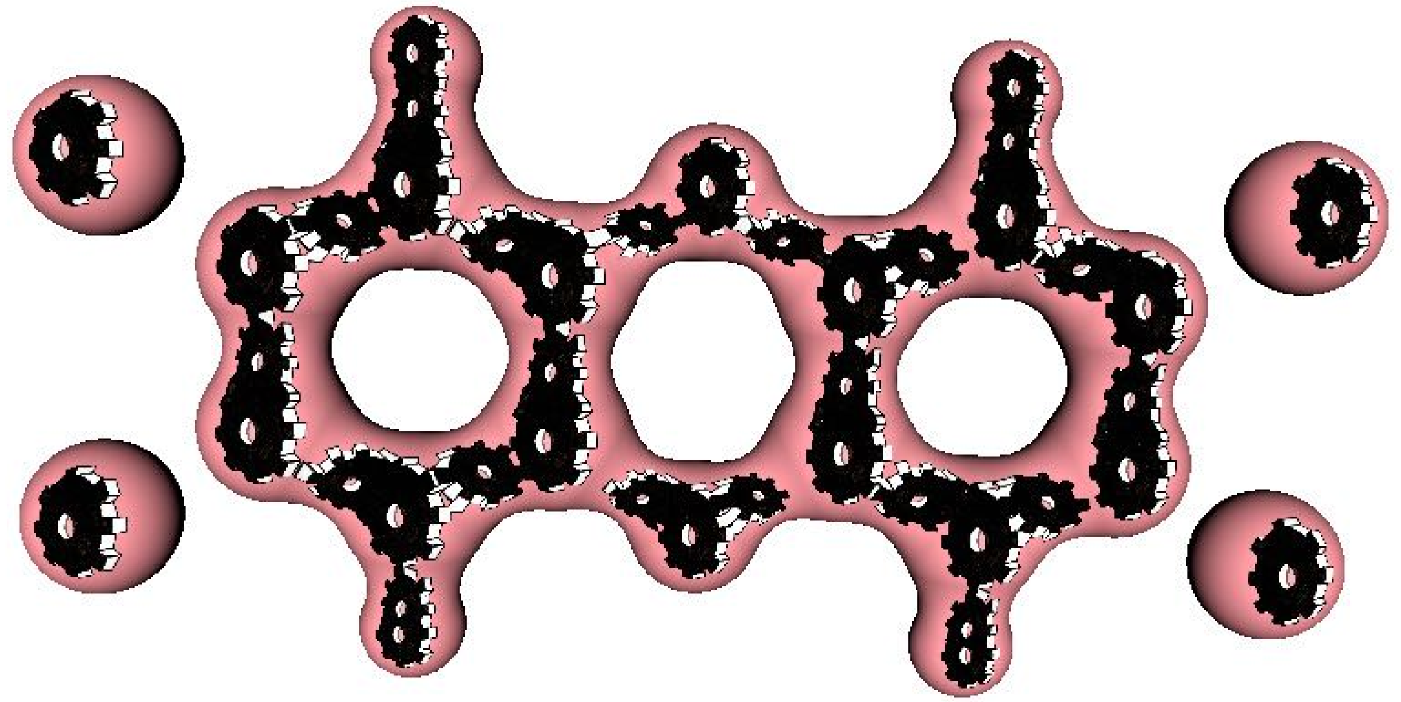

Actif/Inactif

Lit-sphère

QUENTIN LEBEAU

Lit-sphère

Nature des atomes et molécules

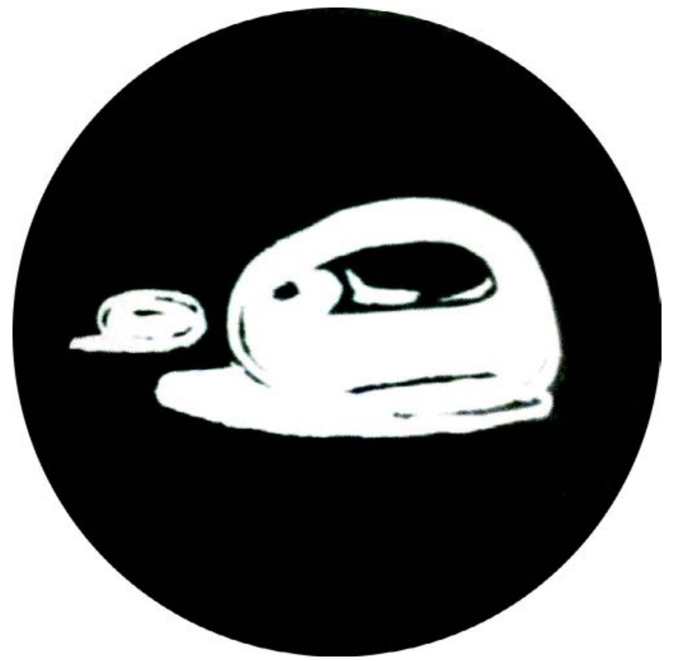
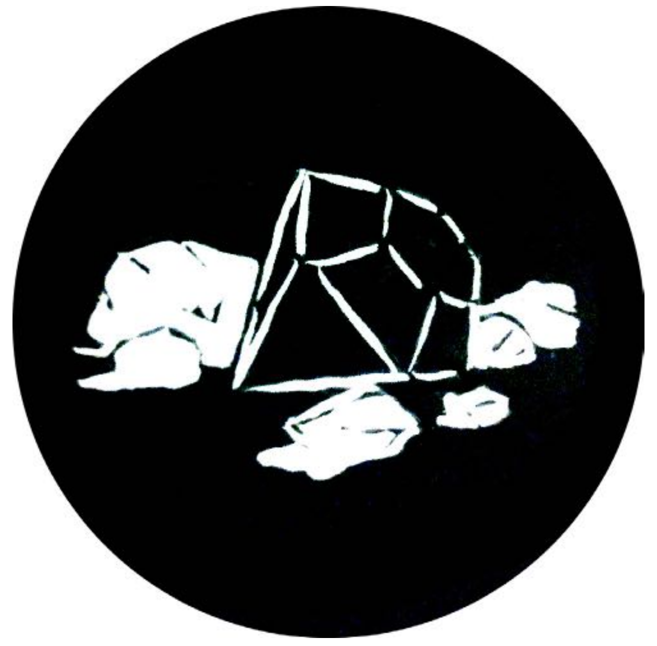
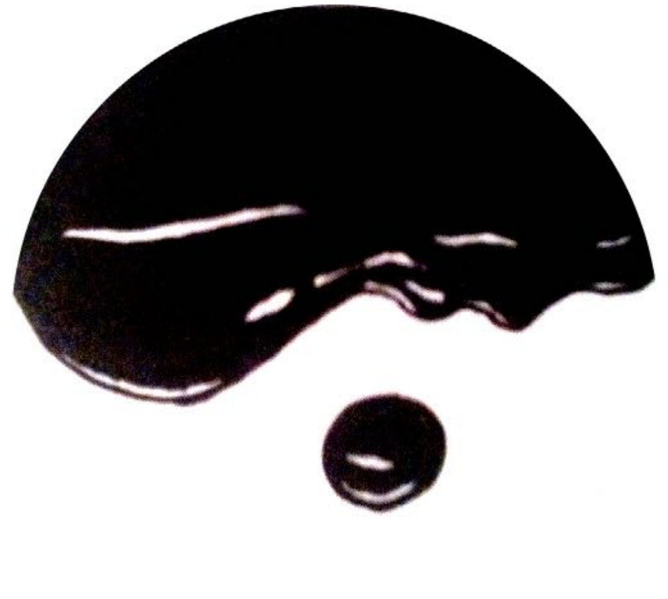
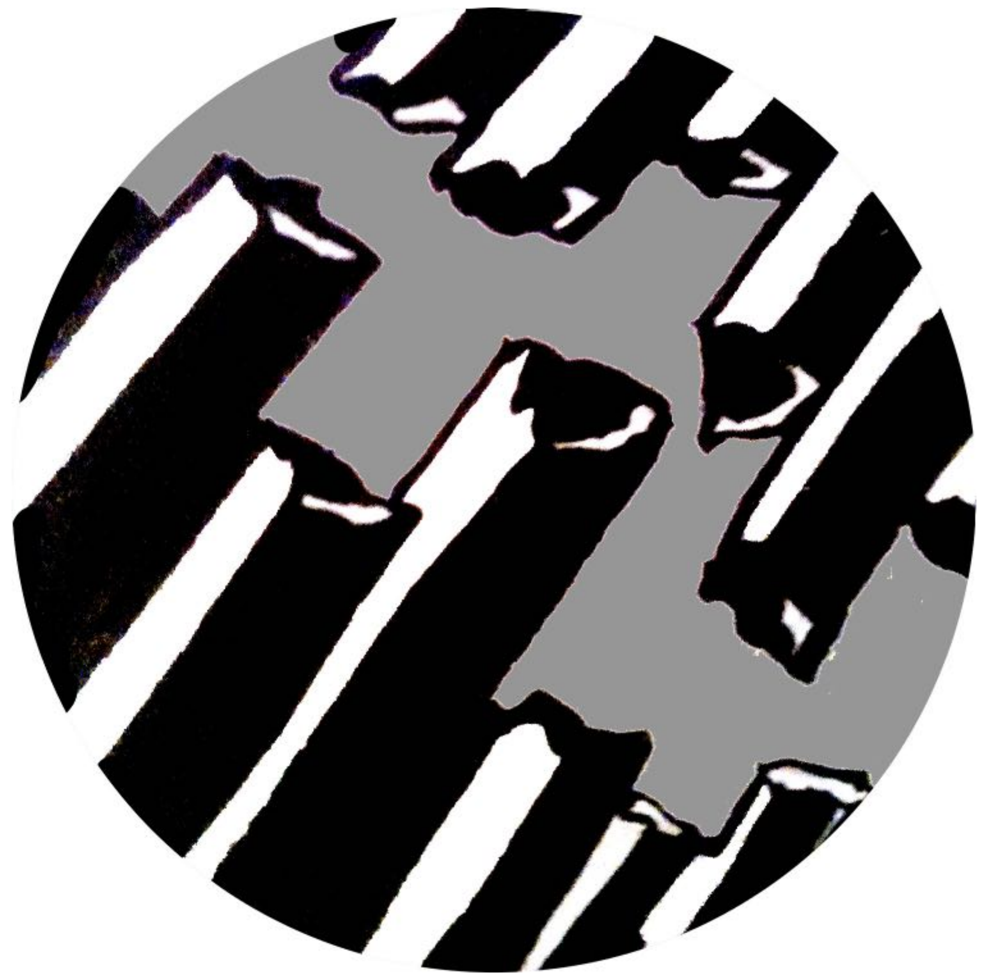
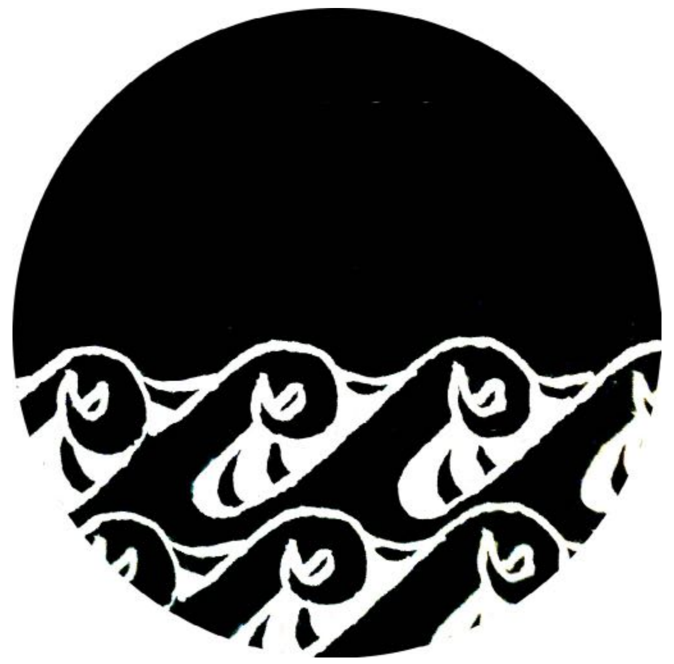
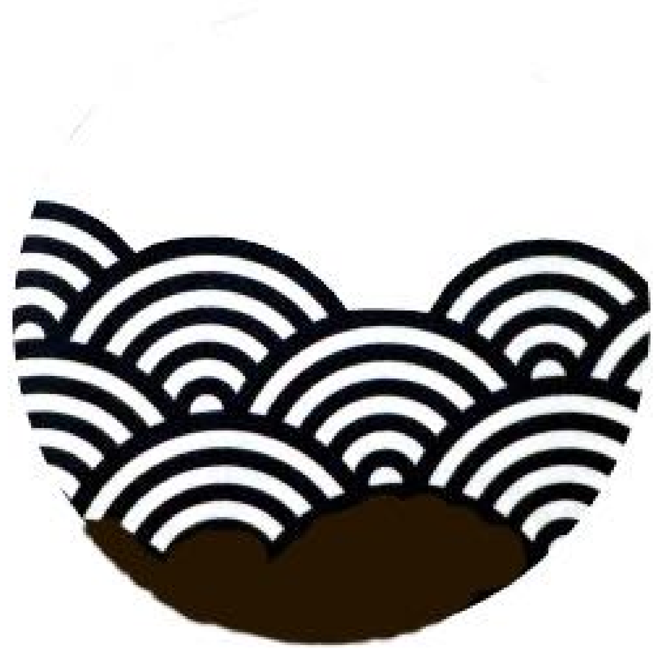
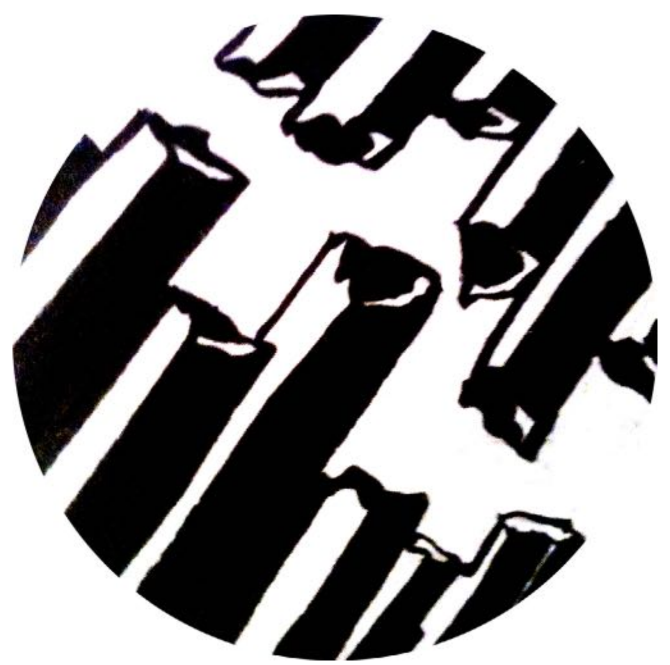
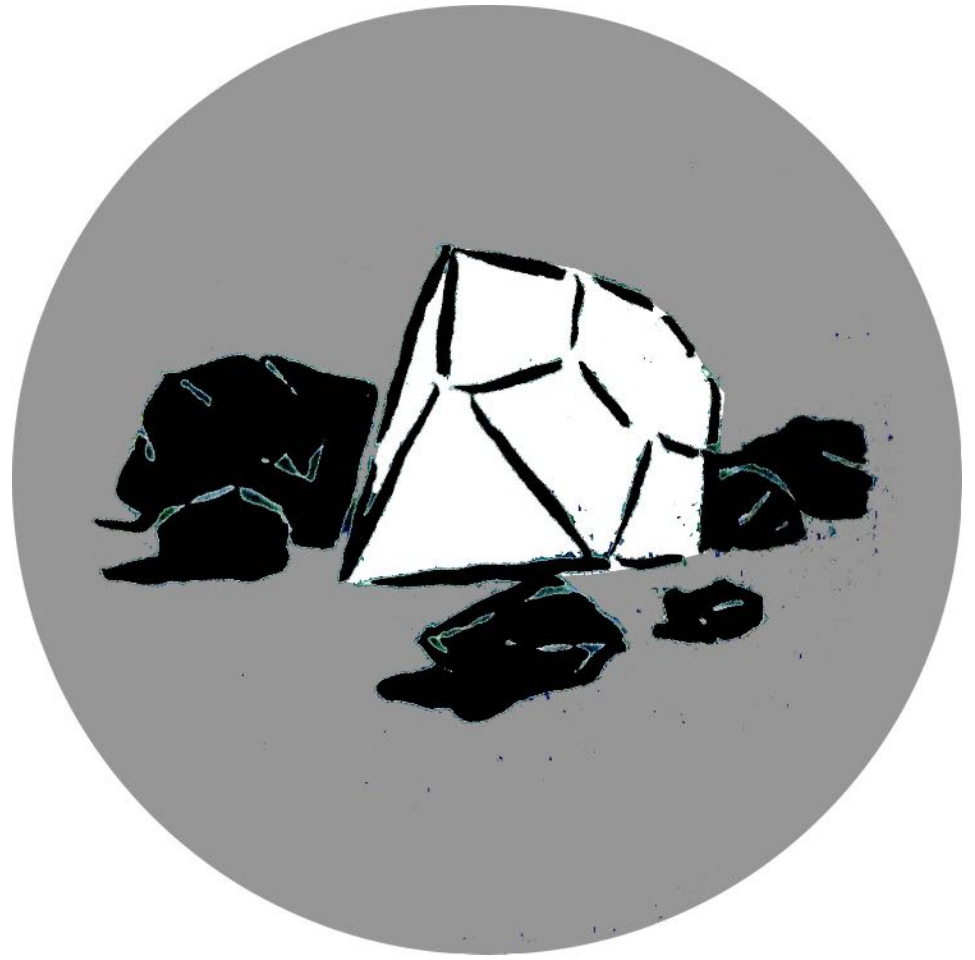
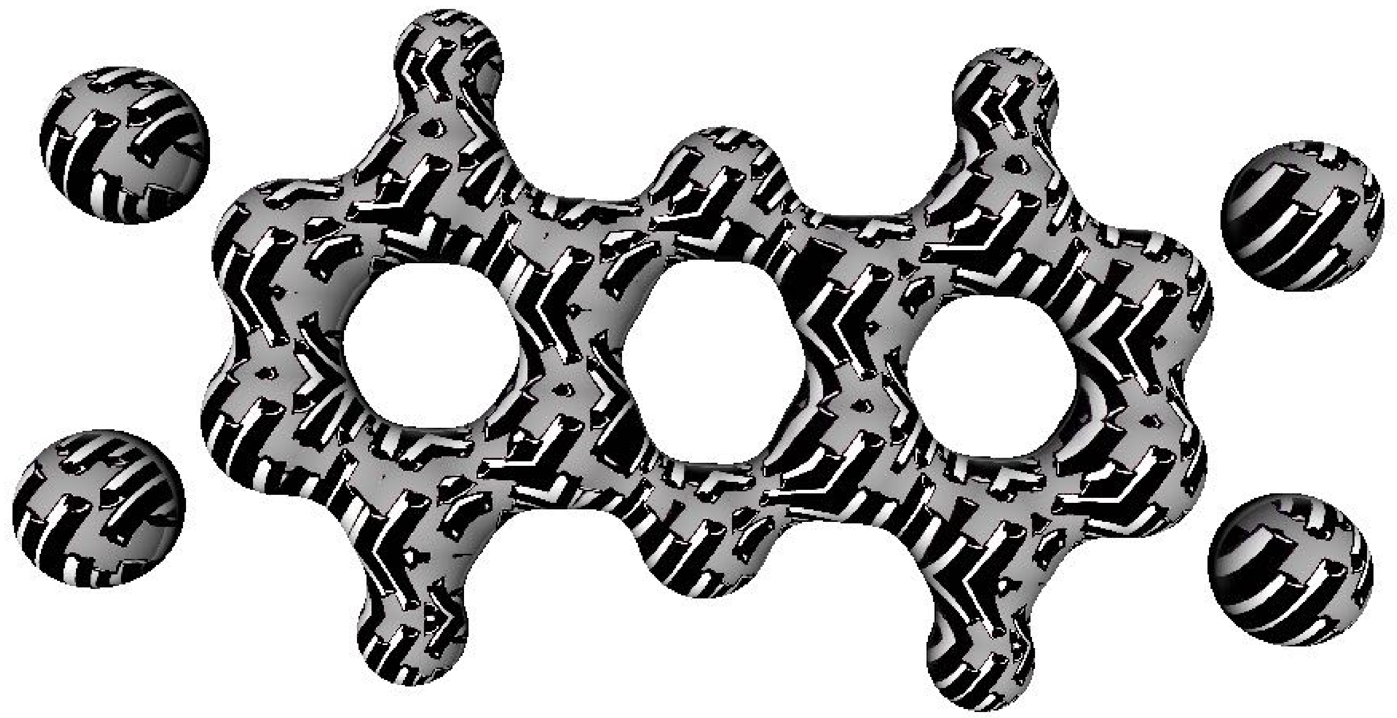

Carbone

Lit-sphère

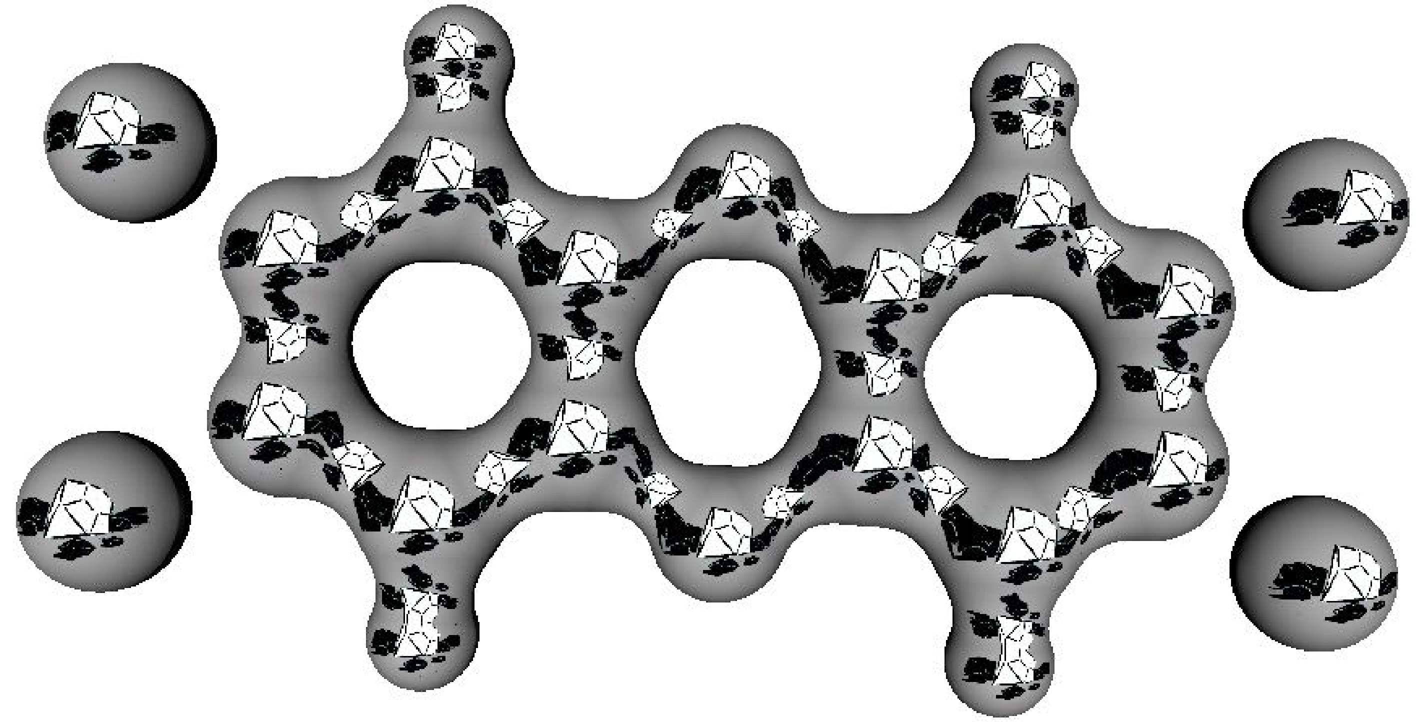

QUENTIN LEBEAU

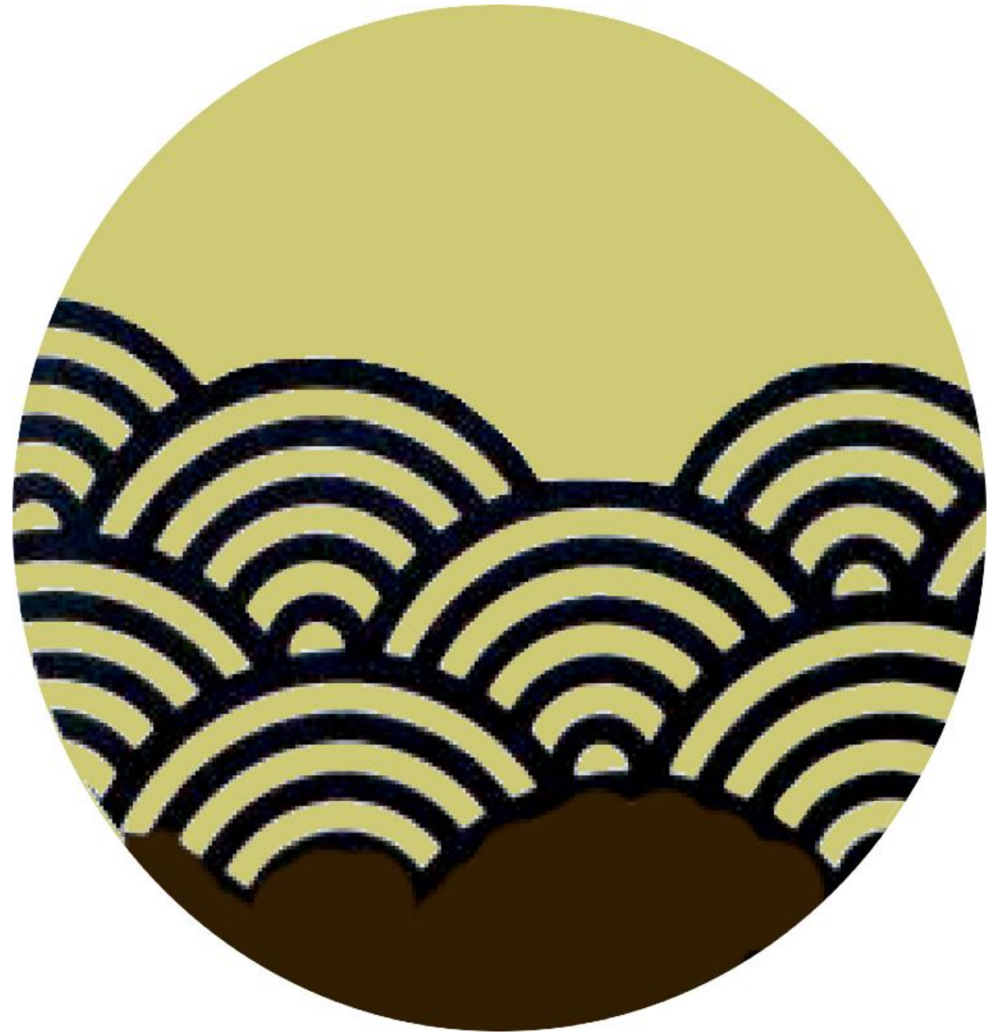

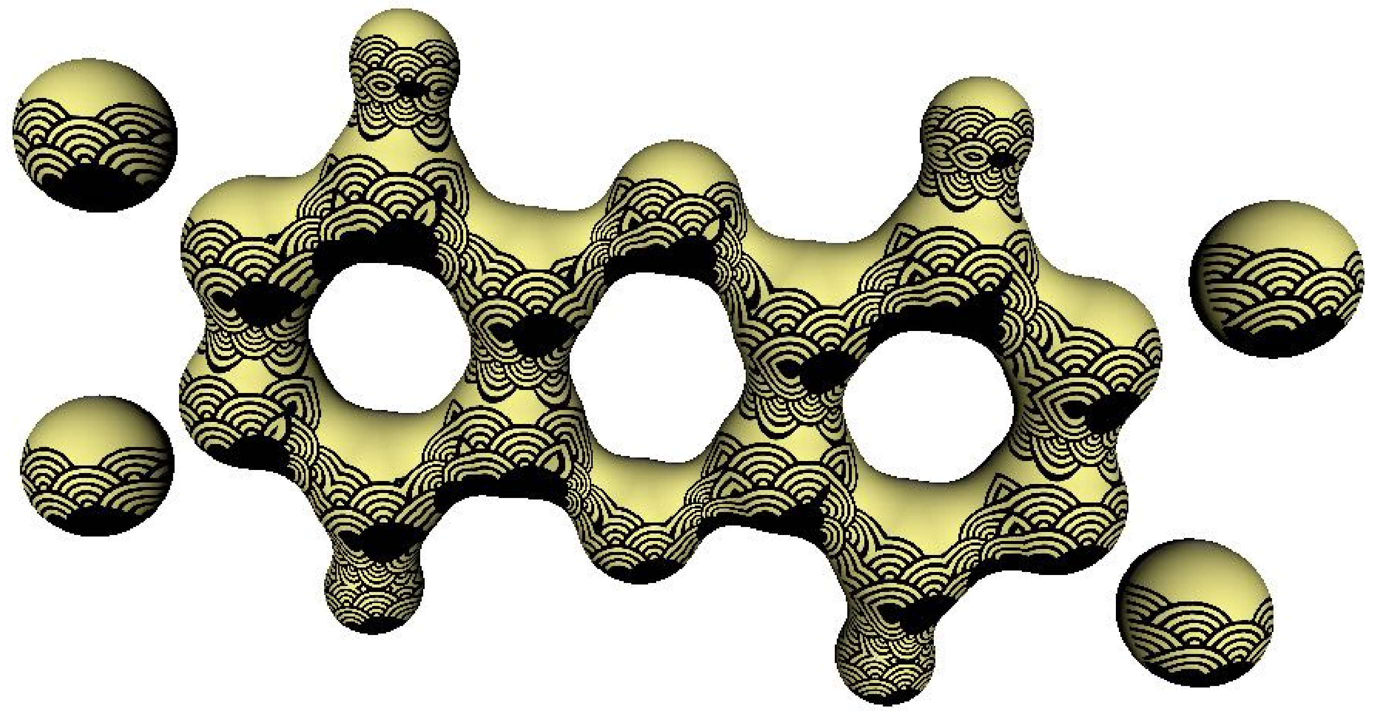

Oxygène

Lit-sphère

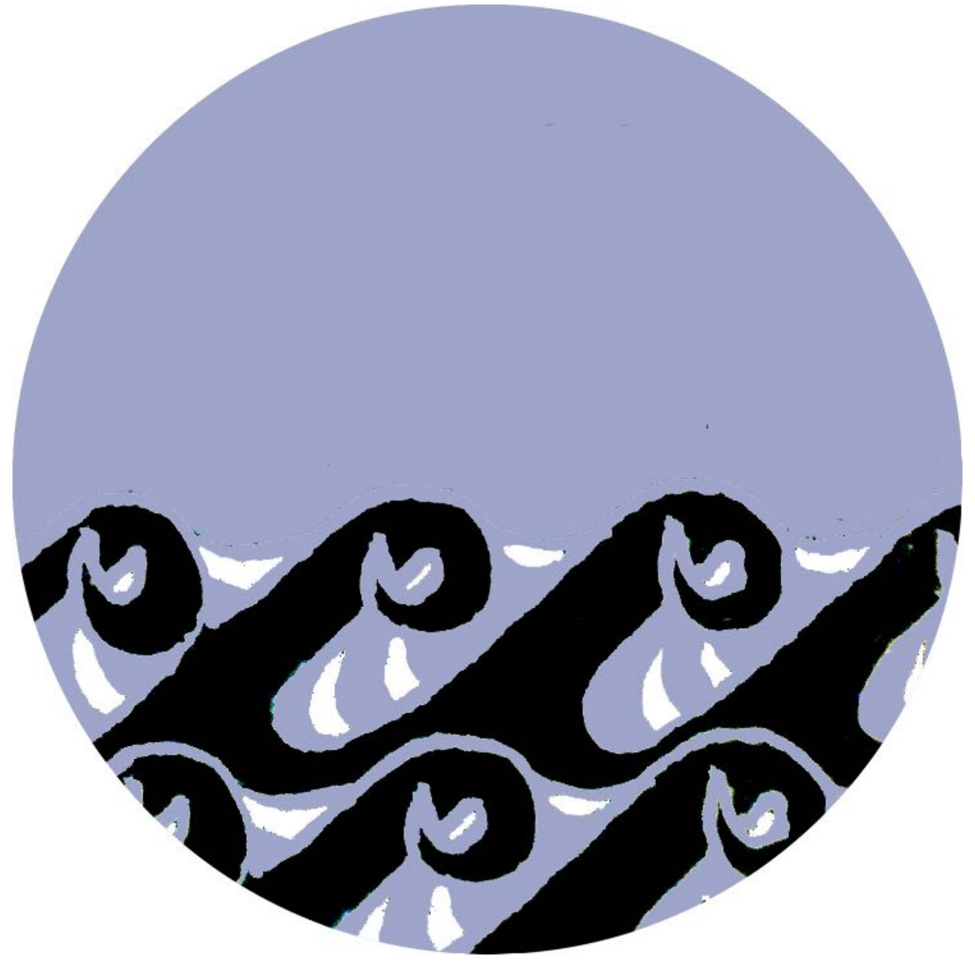

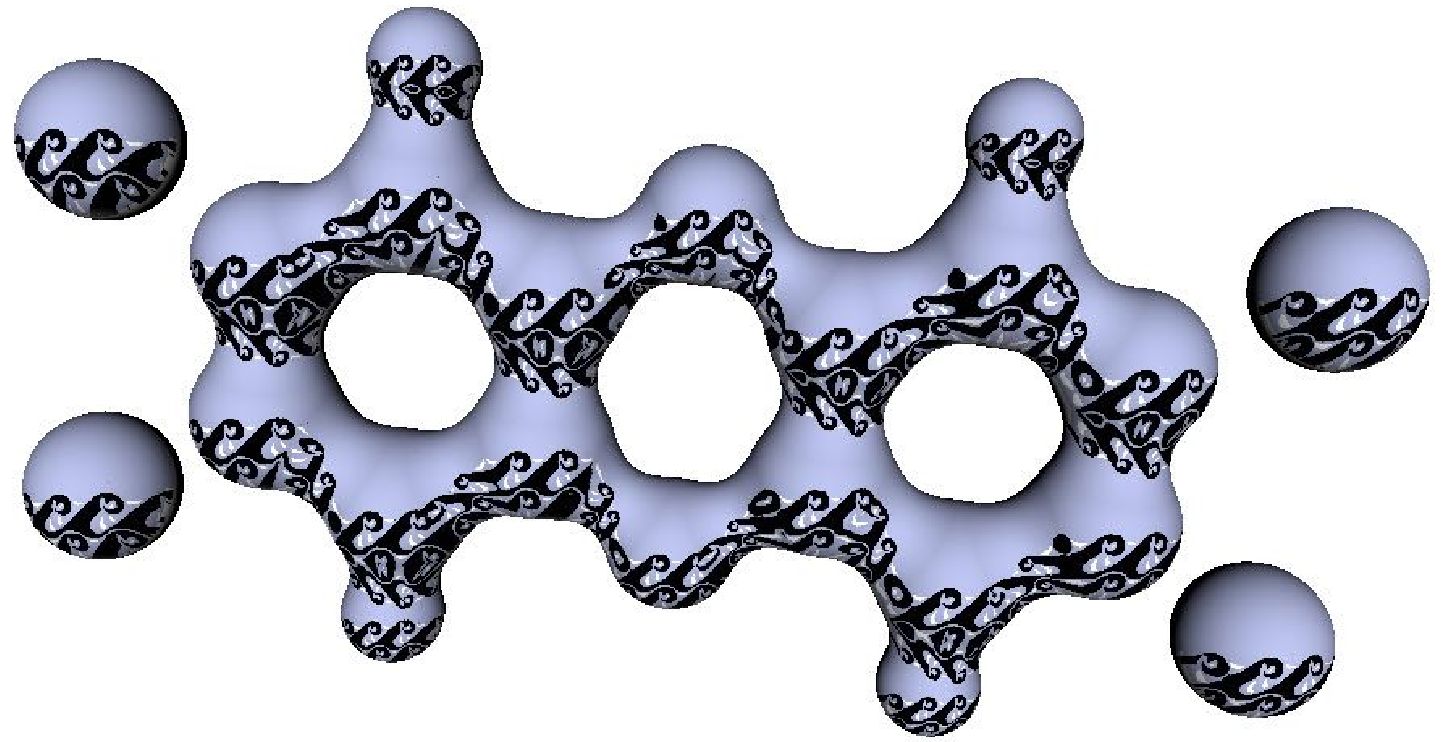

Eau

Lit-sphère

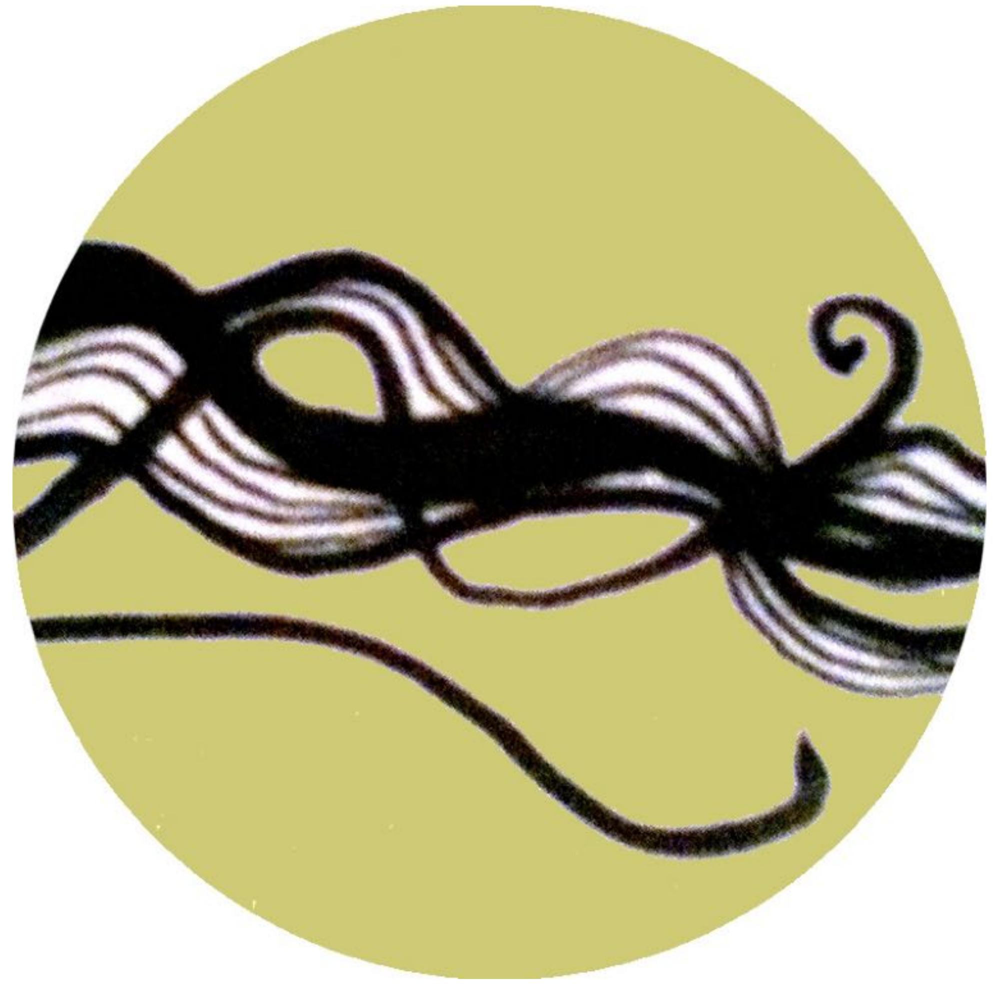

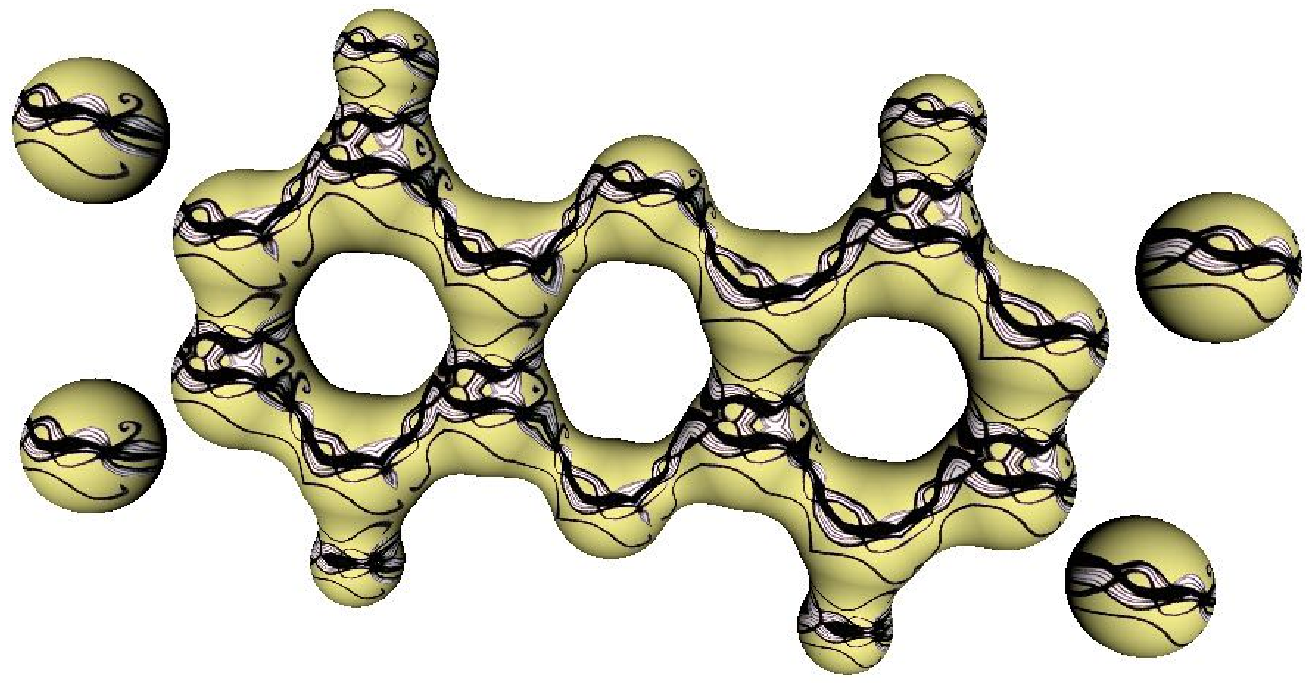

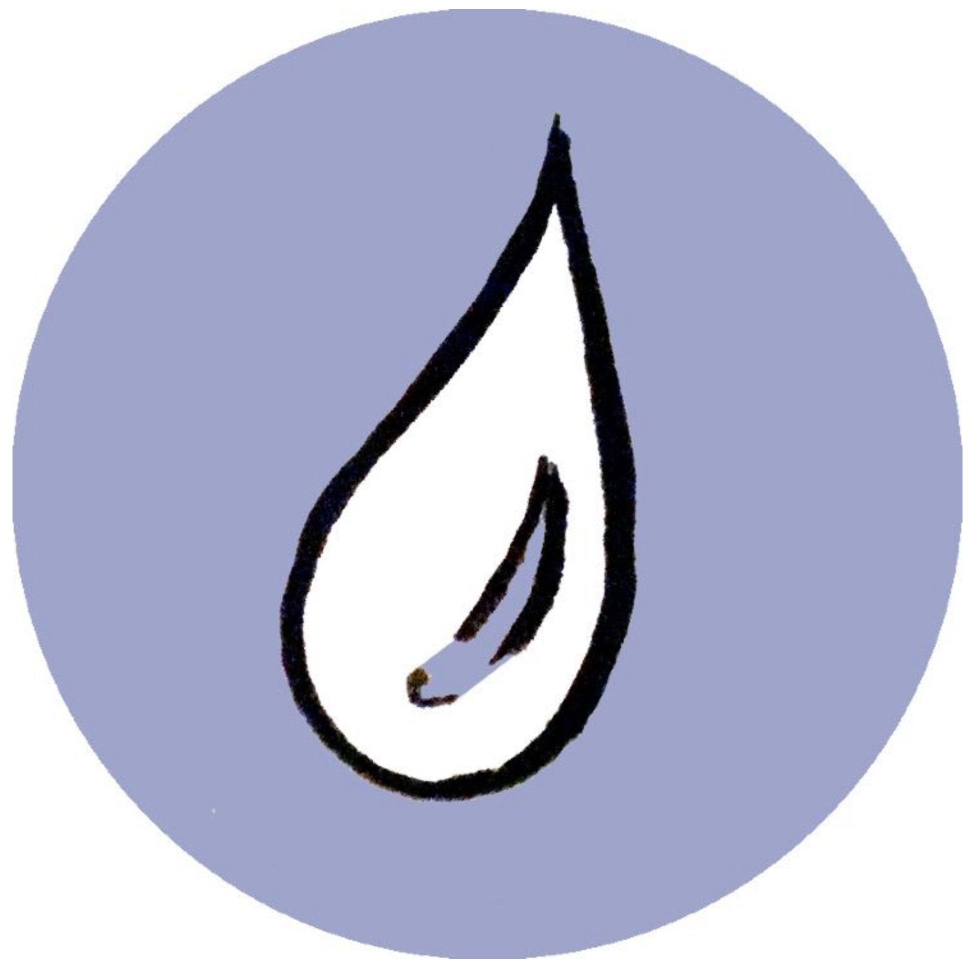

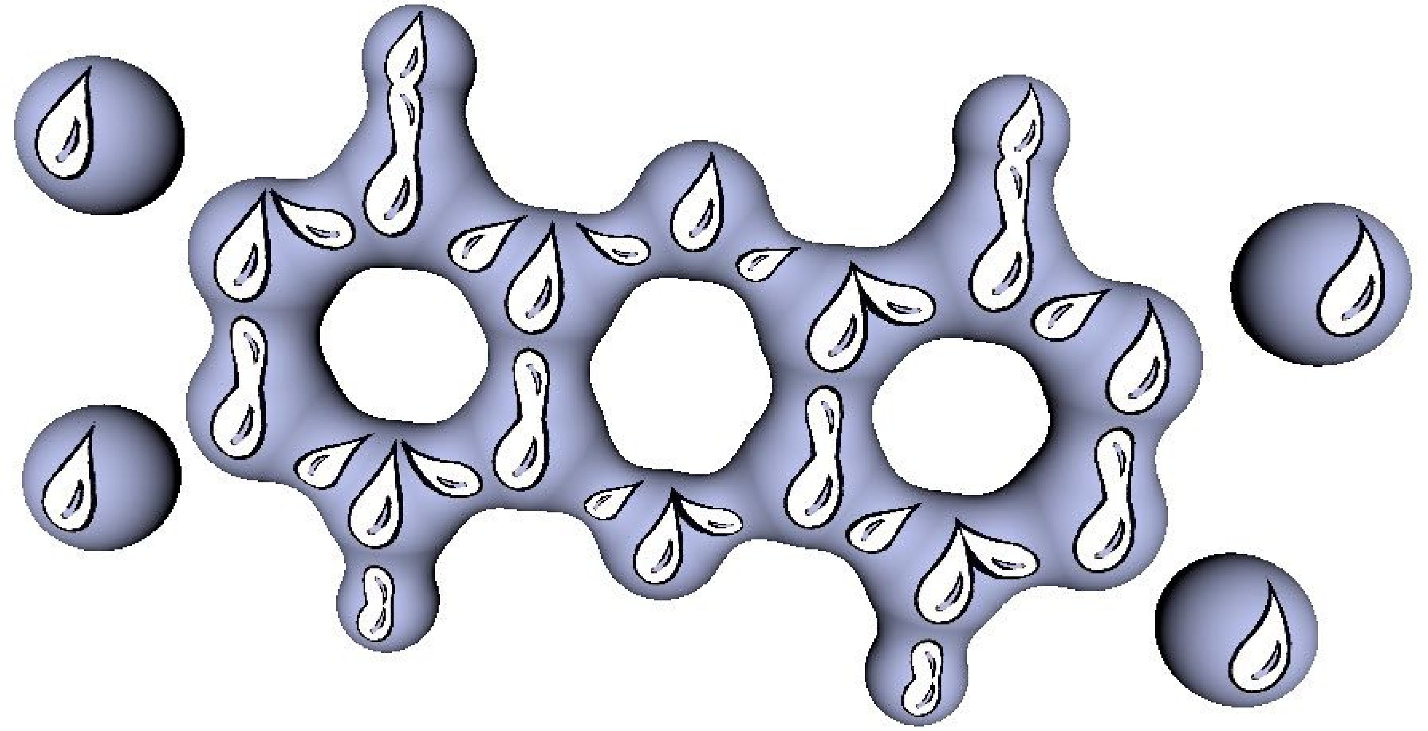

QUENTIN LEBEAU

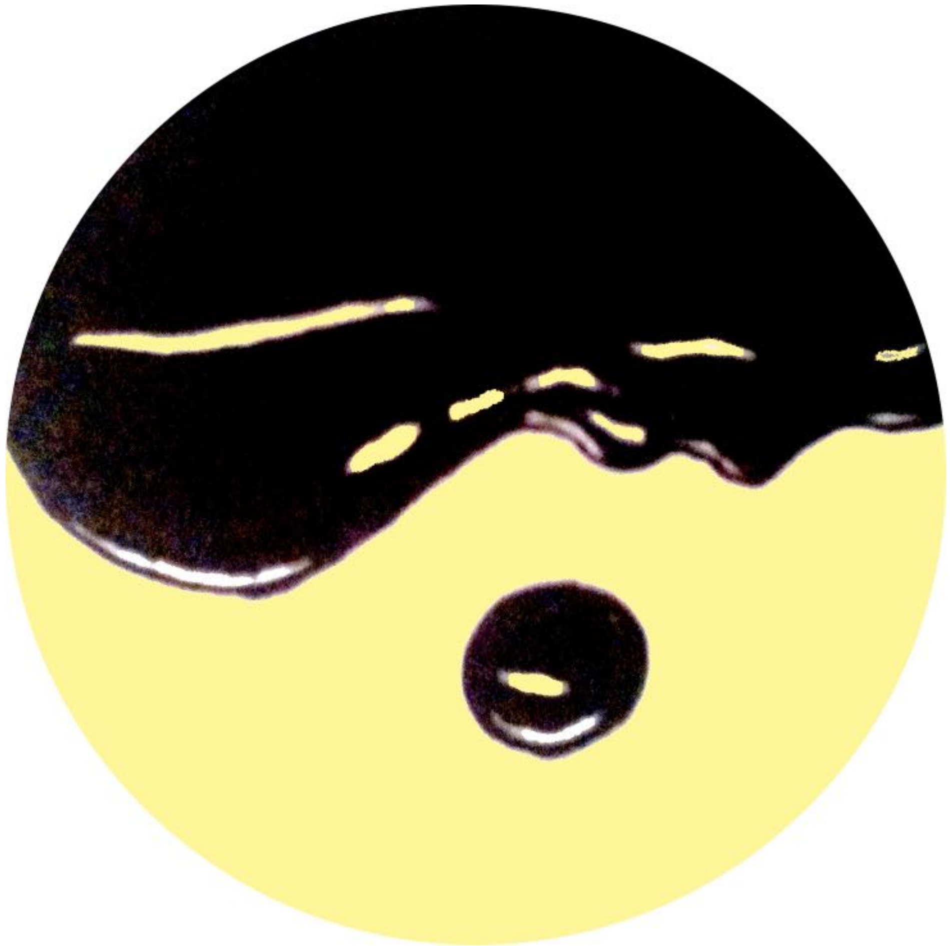

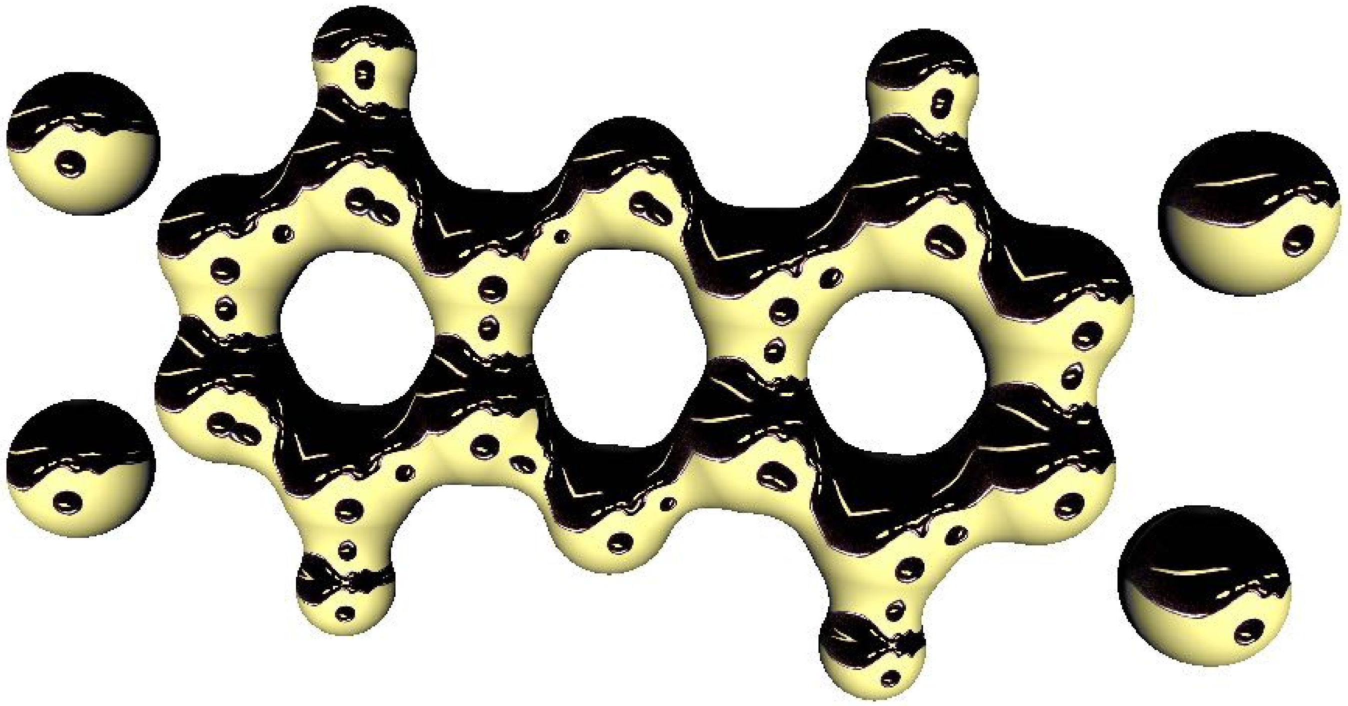

Graisse

Lit-sphère

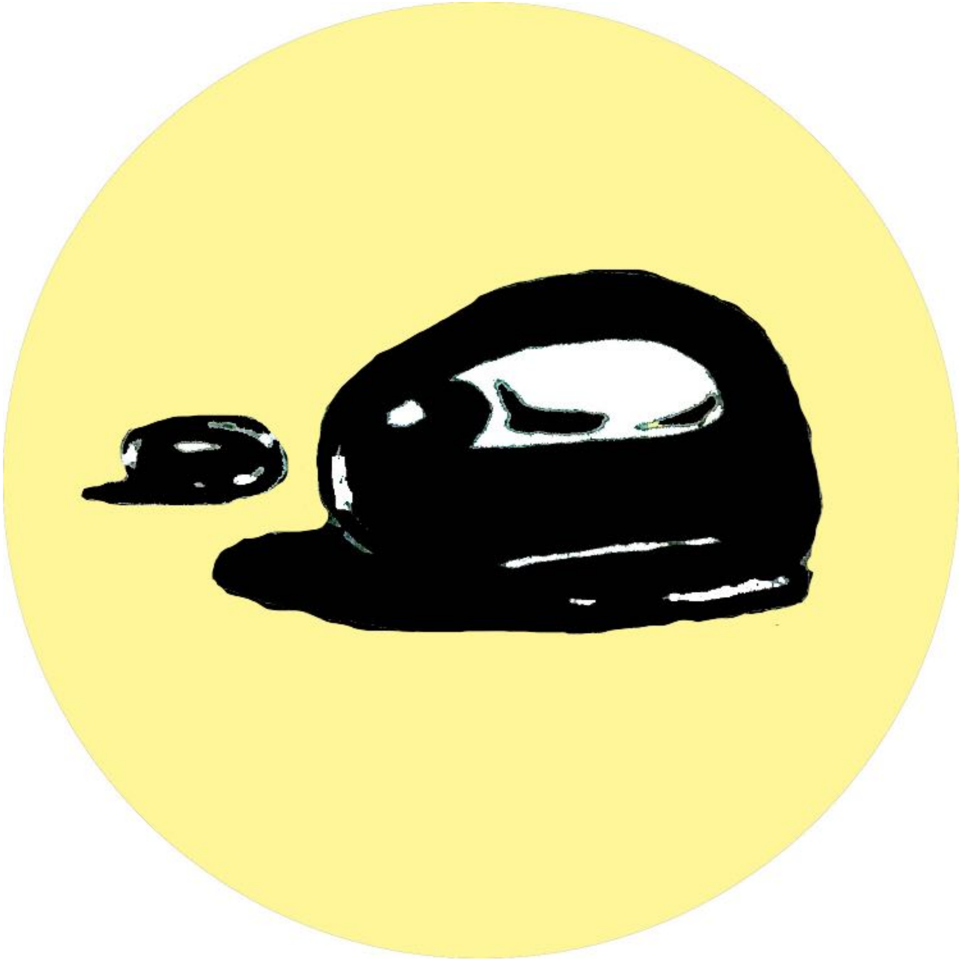

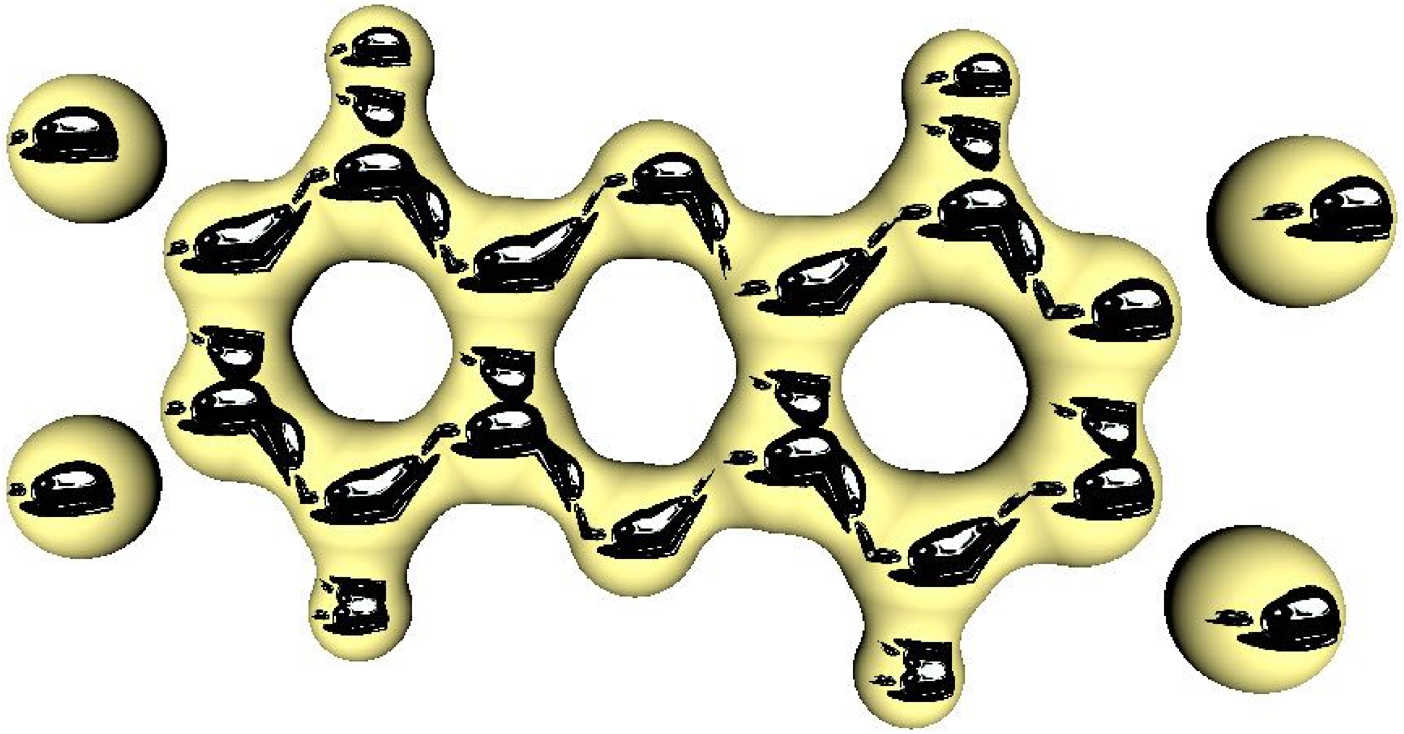

LIT-SPHÈRES

EVE NAGY

LIT-SPHÈRES

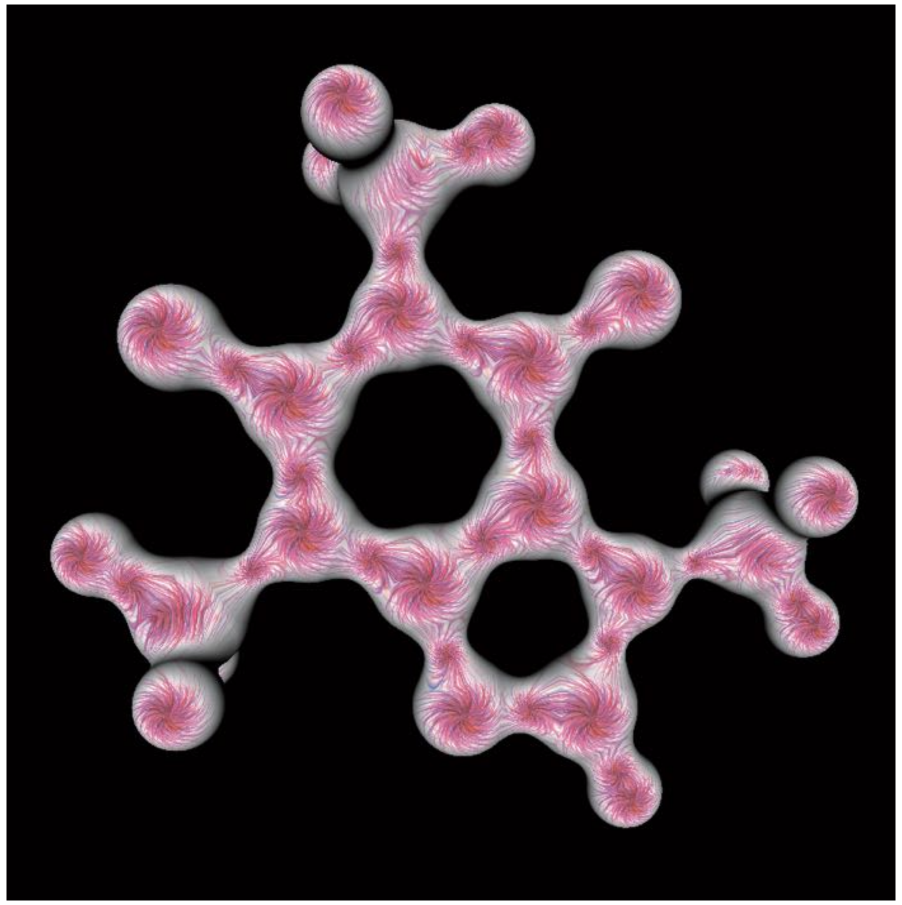

SITE ACTIF

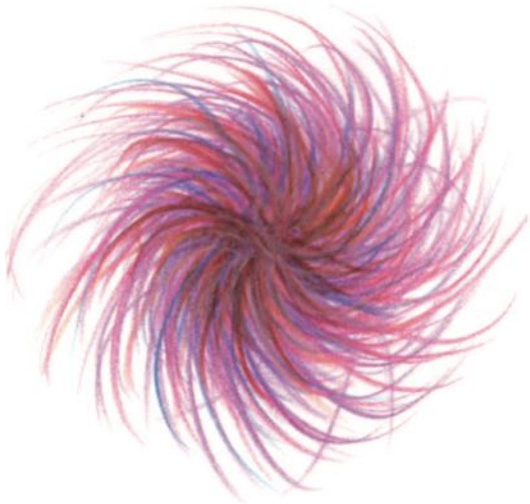

EVE NAGY

LIT-SPHÈRES

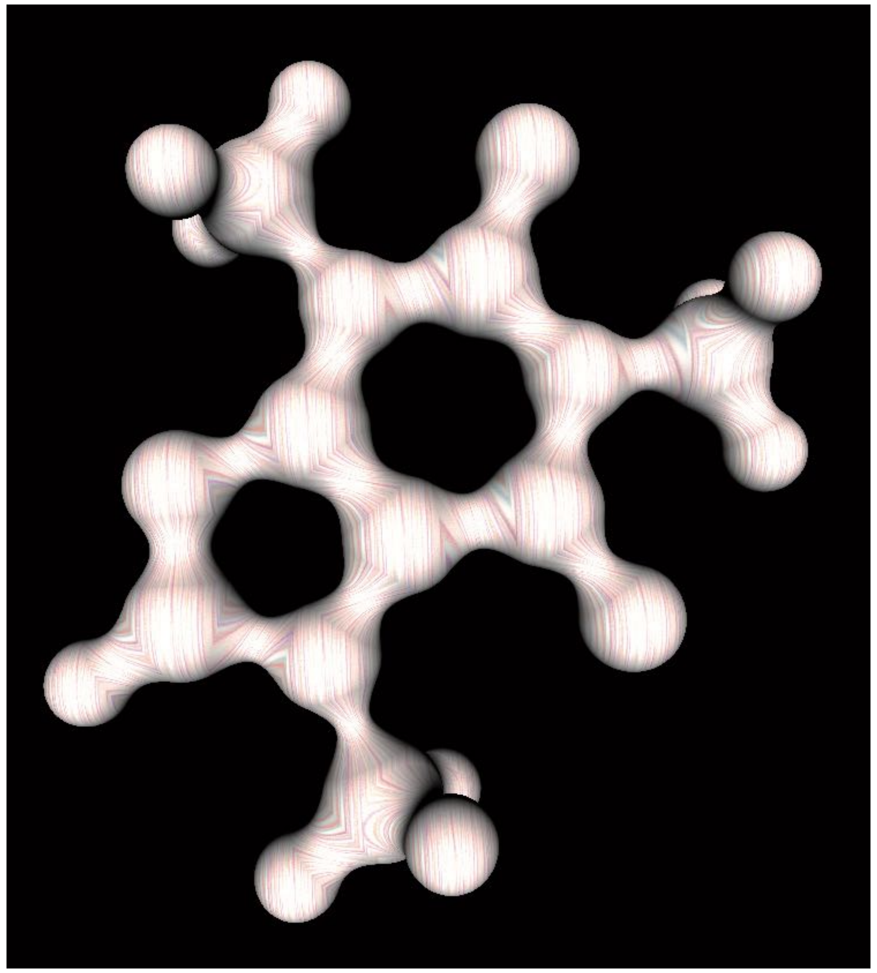

SITE PASSIF

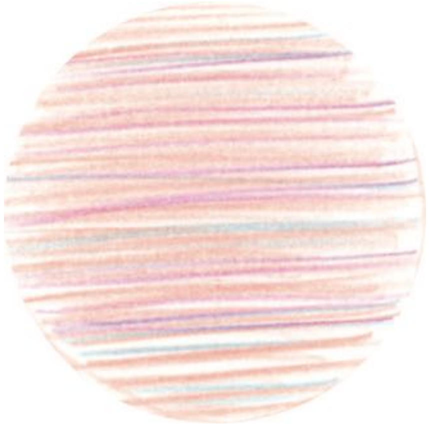

EVE NAGY

LIT-SPHÈRES

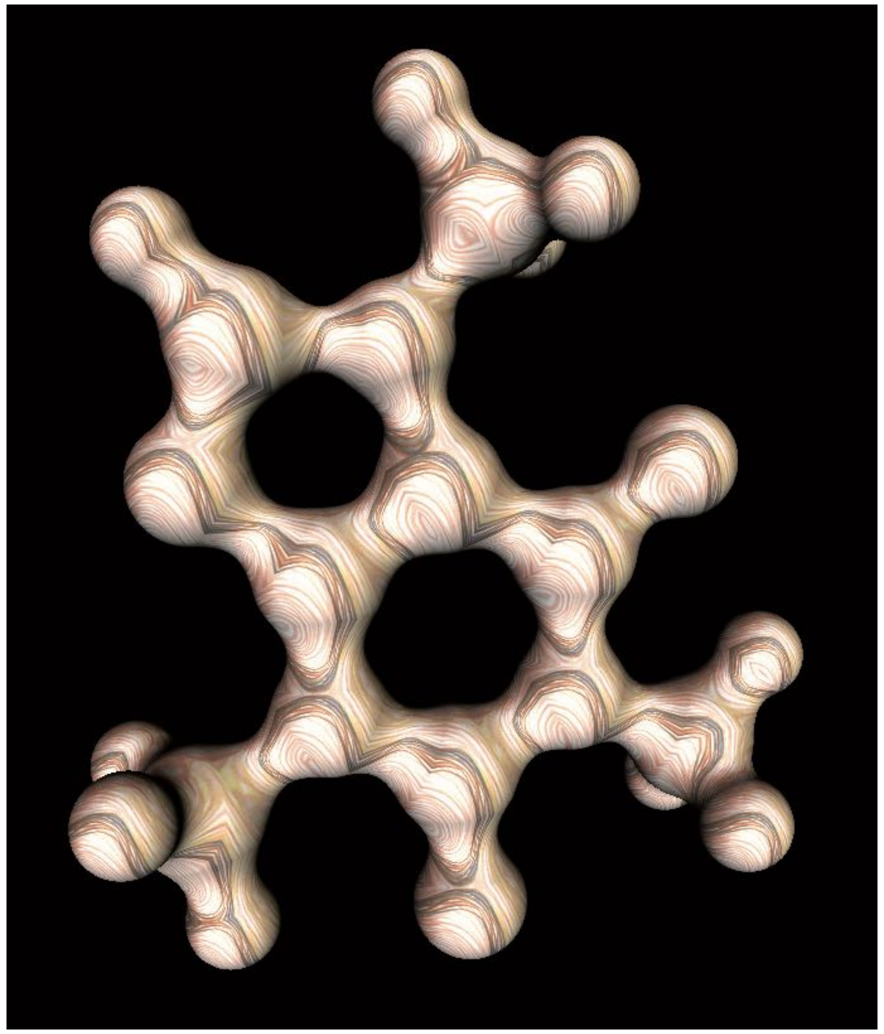

RIGIDE

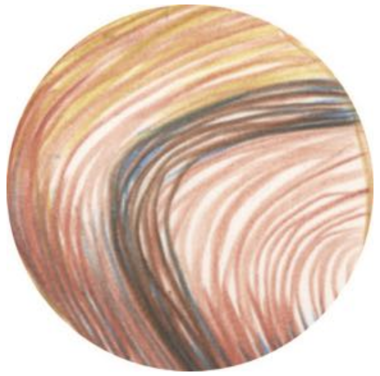

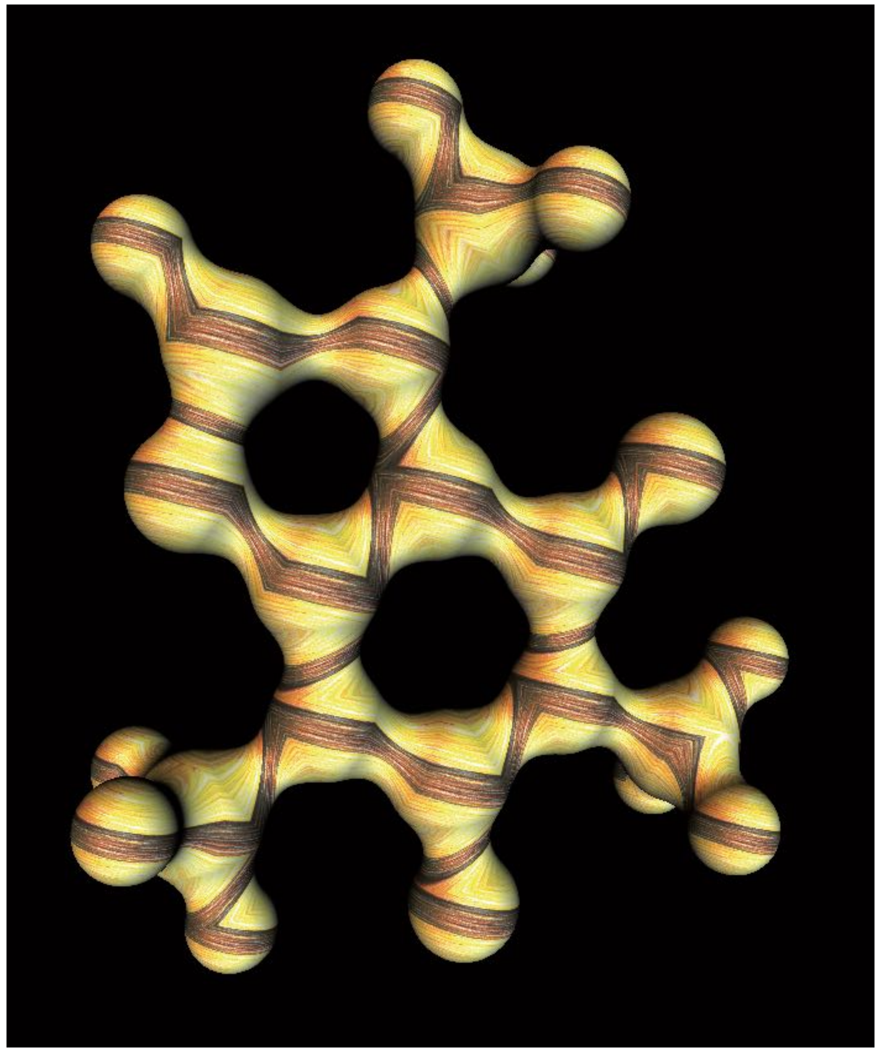

FLEXIBLE

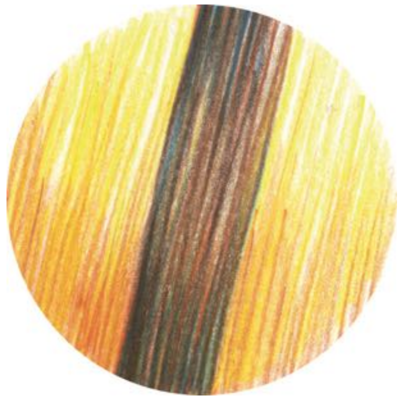

EVE NAGY

LIT-SPHÈRES

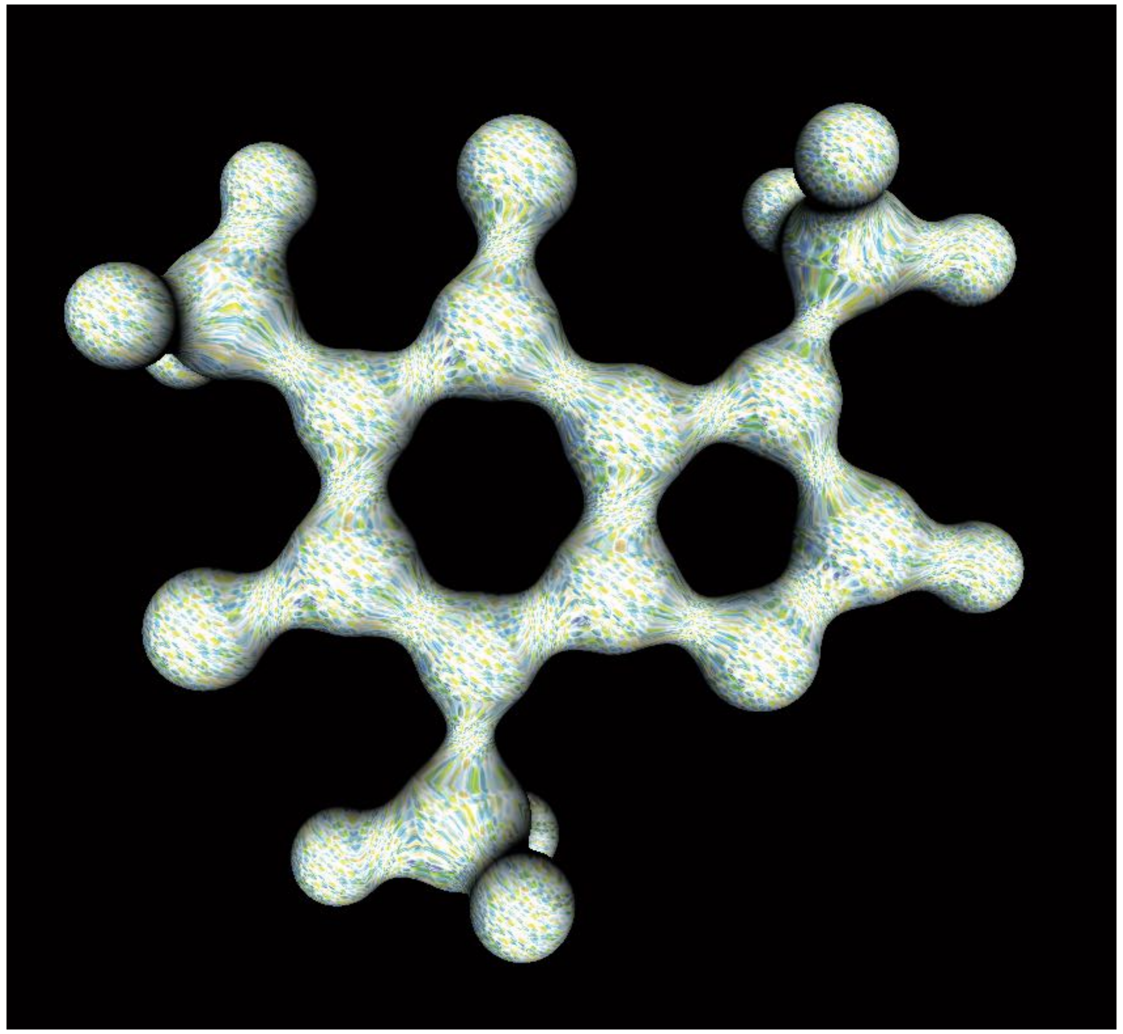

HYDROPHILIE

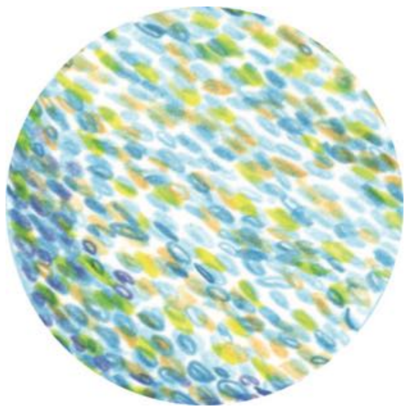

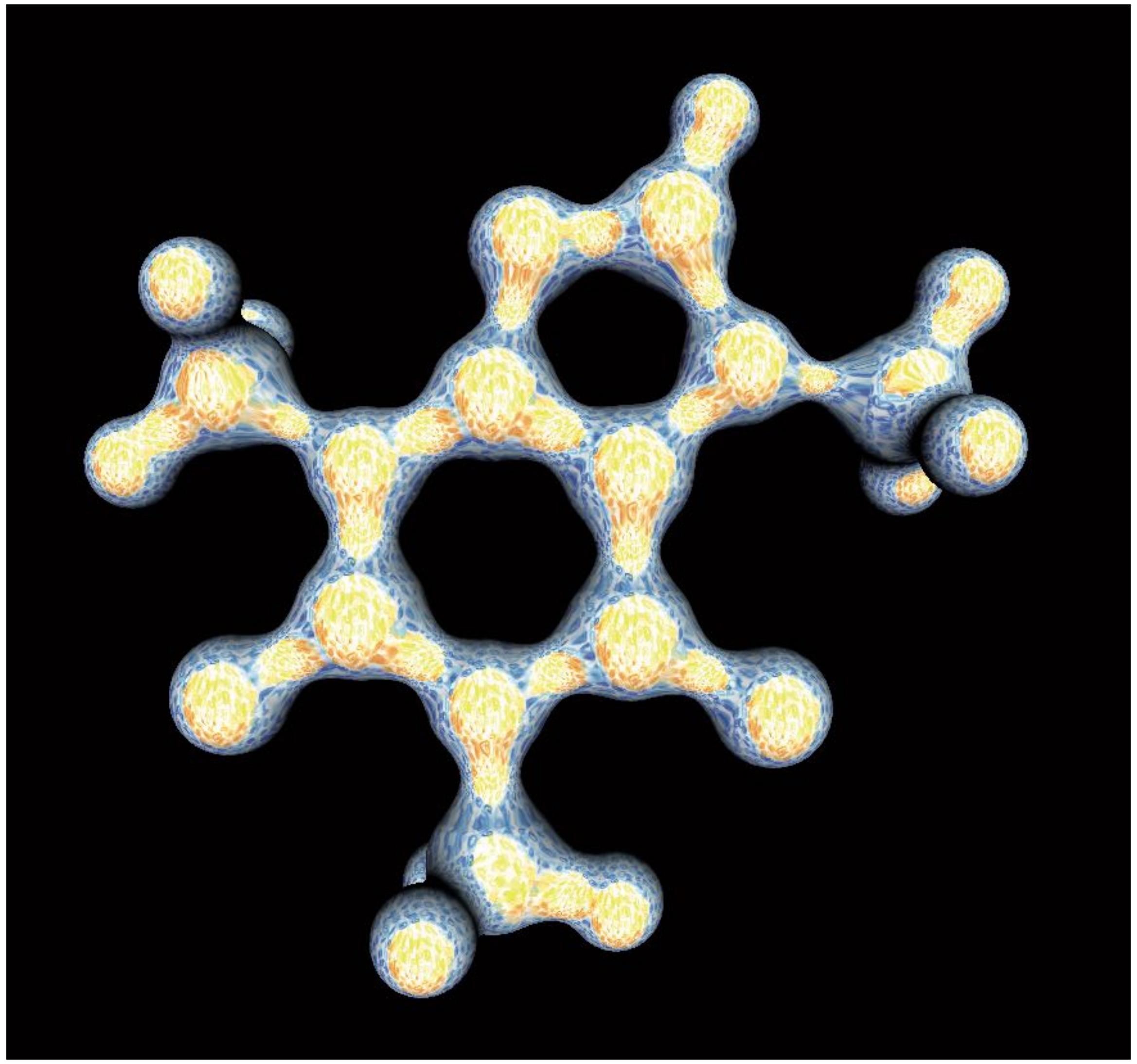

HYDROPHOBIE

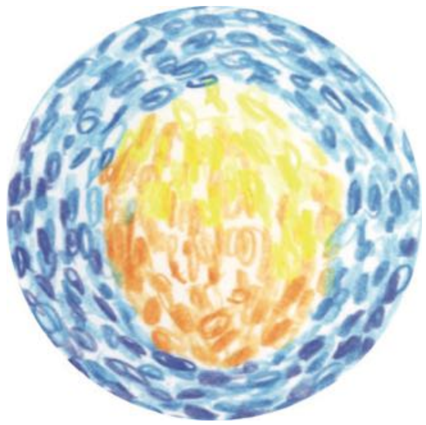

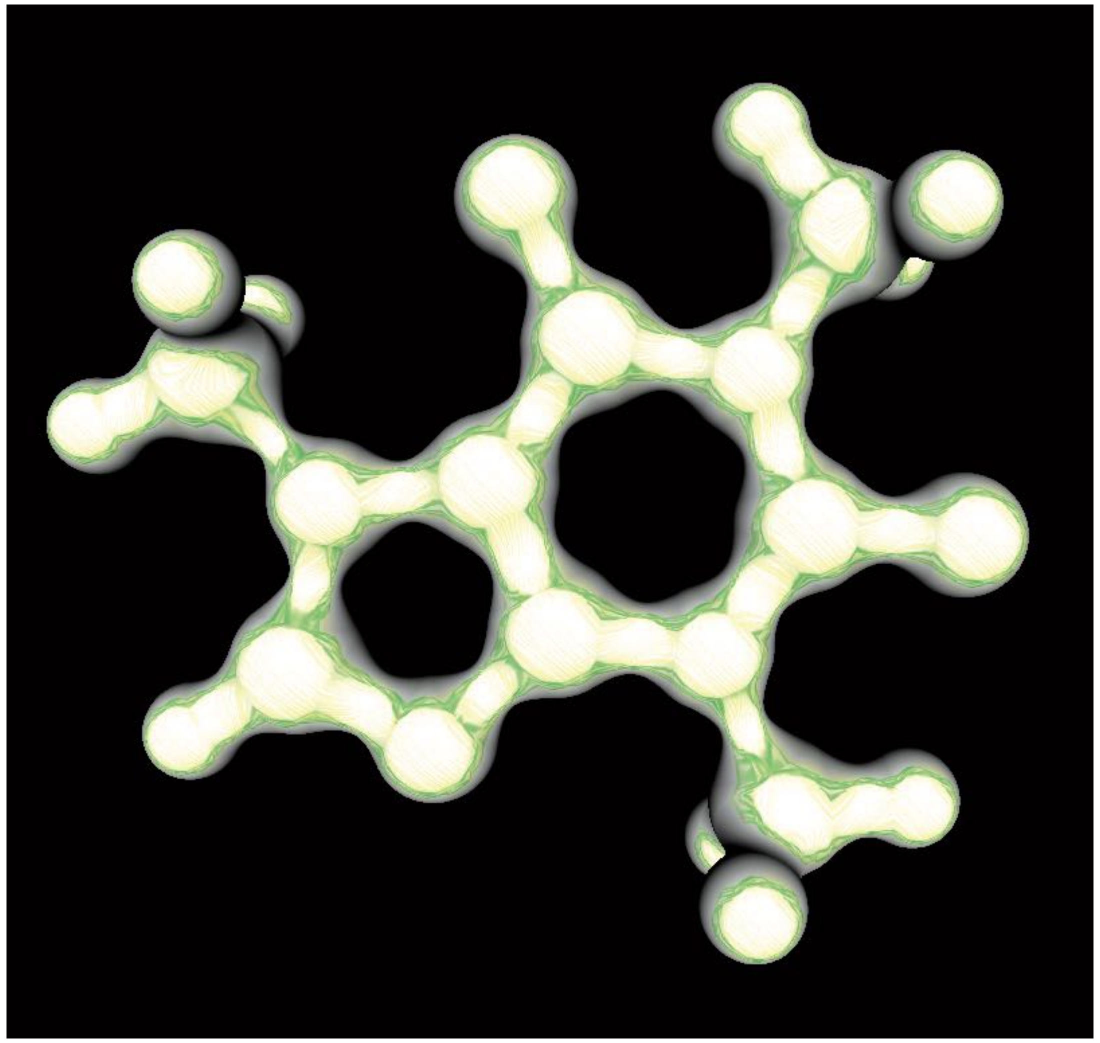

BONNE
QUALITÉ

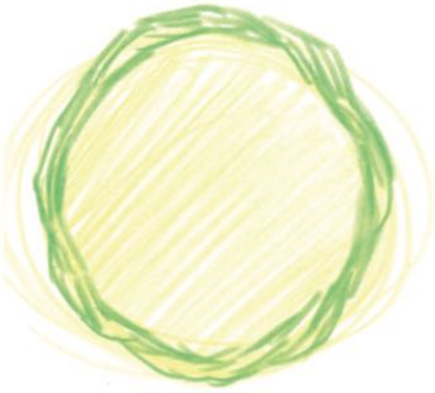

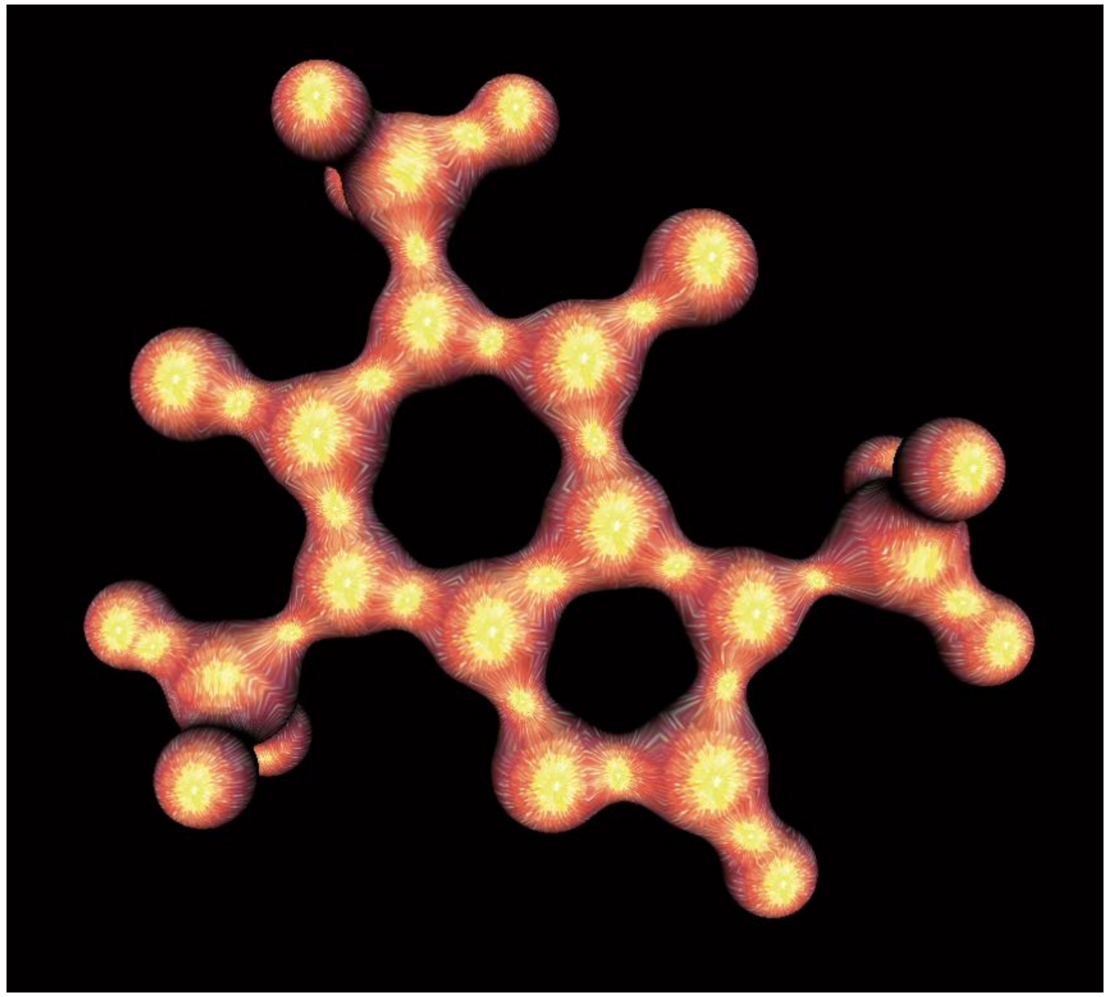

MAUVAISE
QUALITÉ

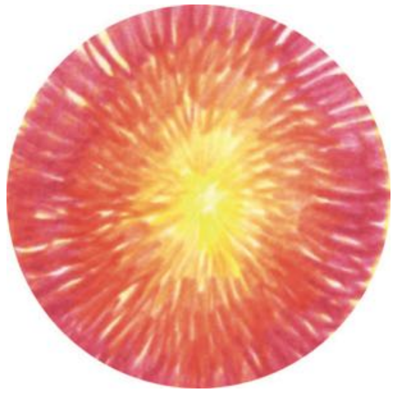

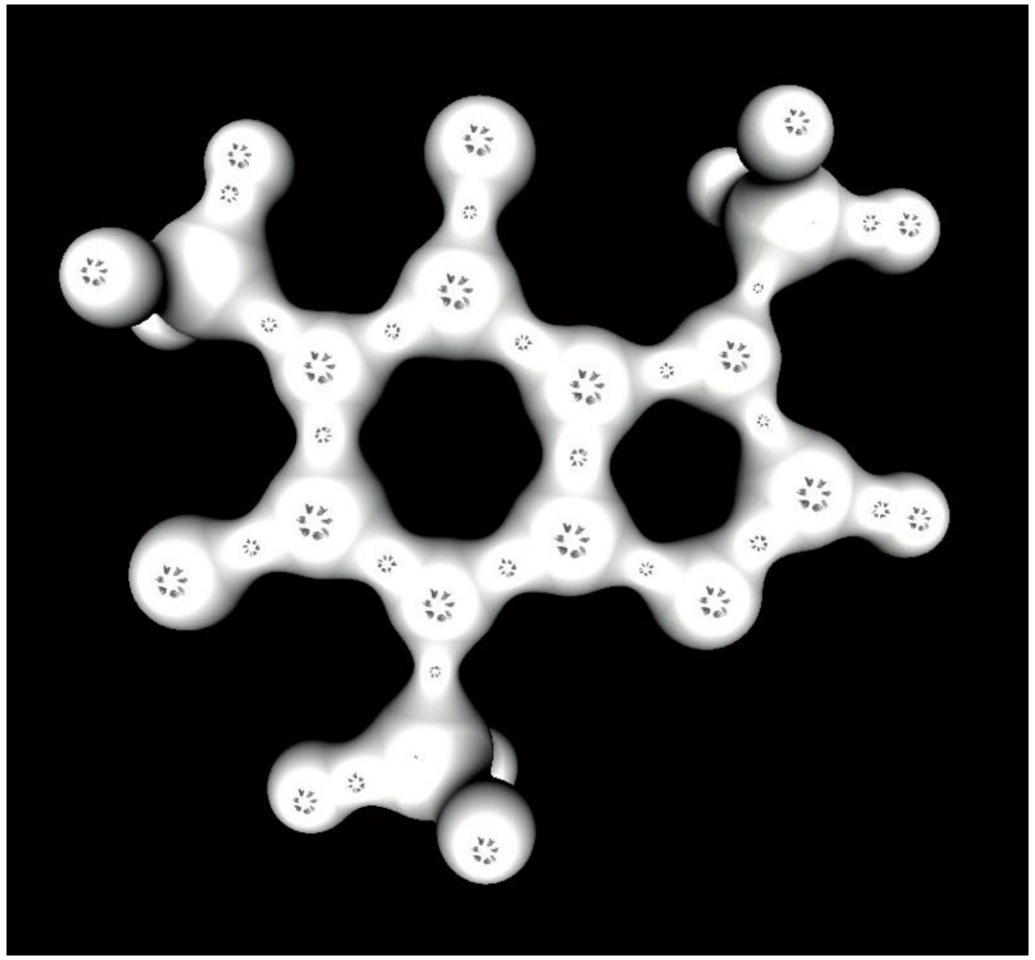

POLARITÉ

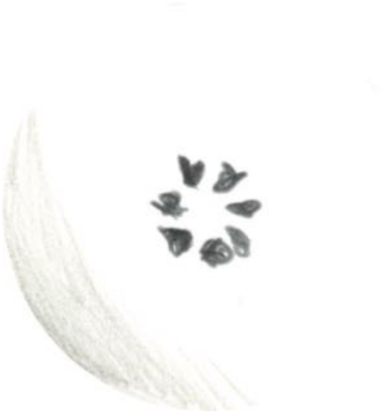

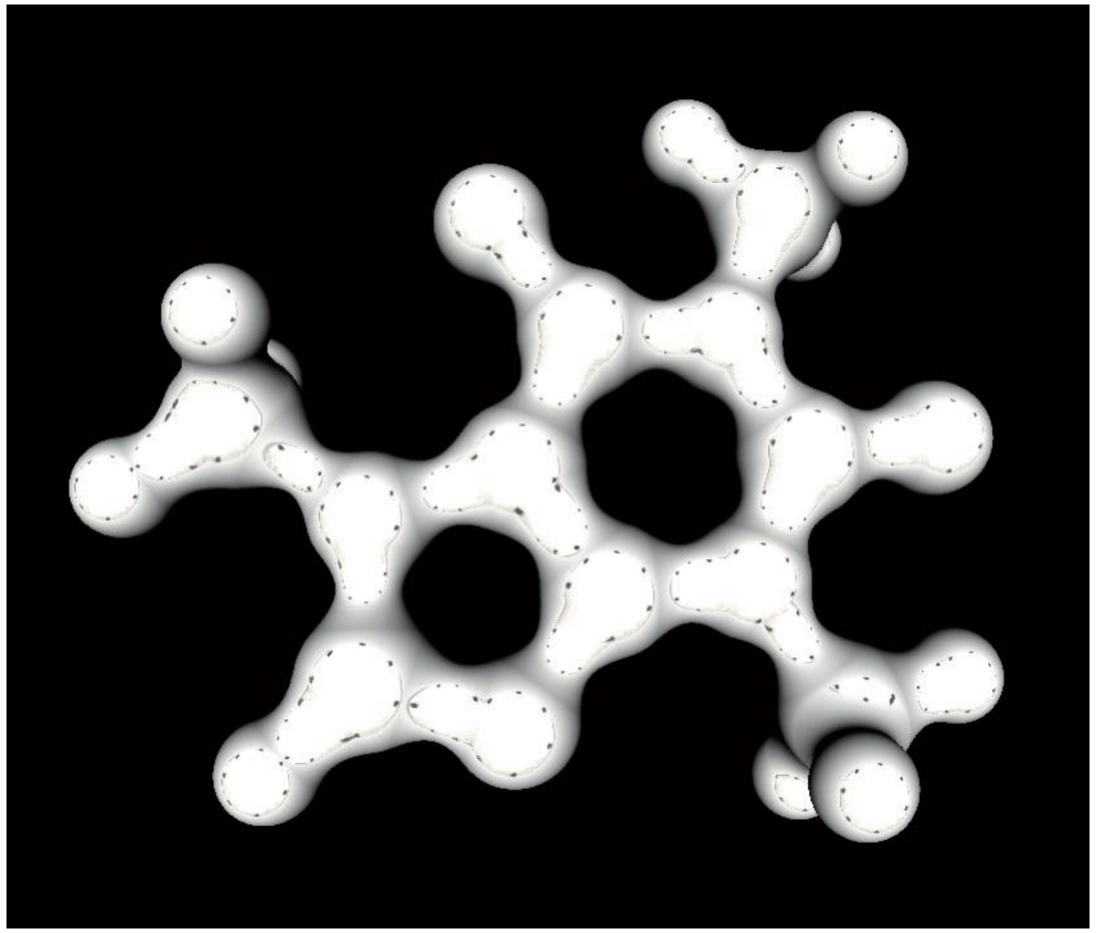

POLARITÉ

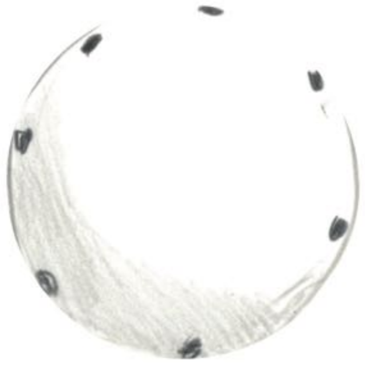

EVE NAGY

LIT-SPHÈRES

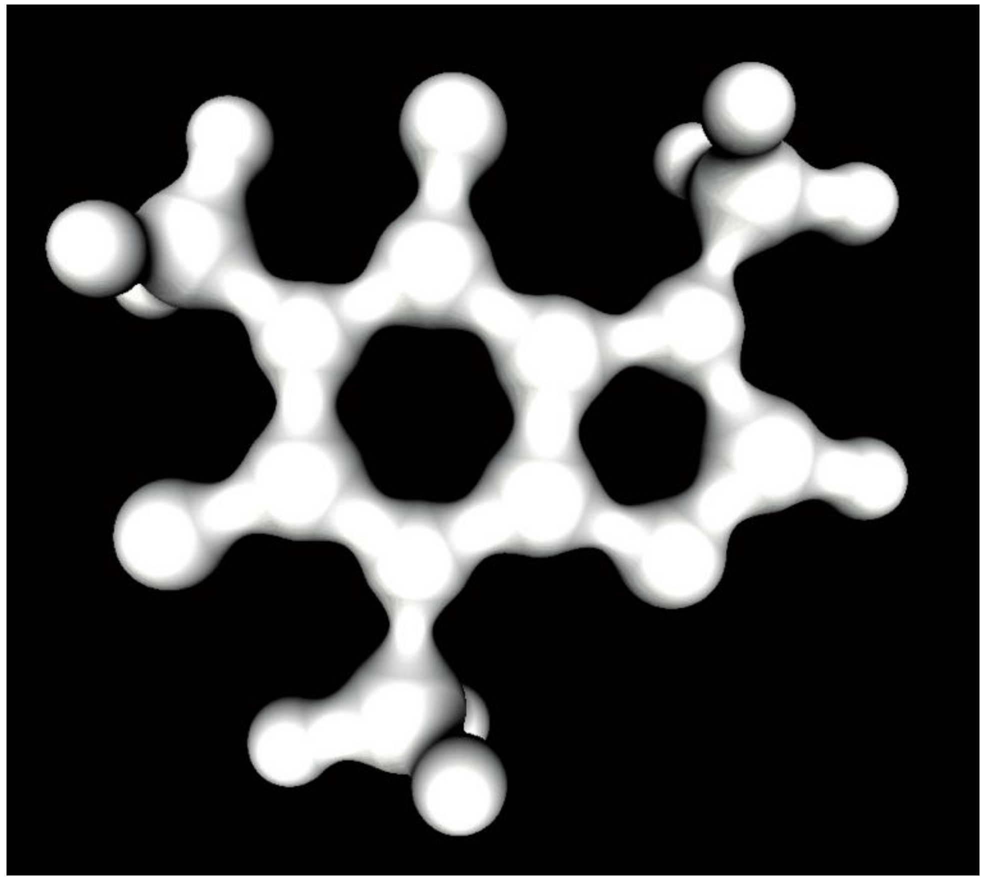

CHARGE

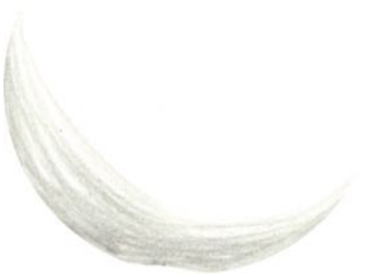

EVE NAGY

LIT-SPHÈRES

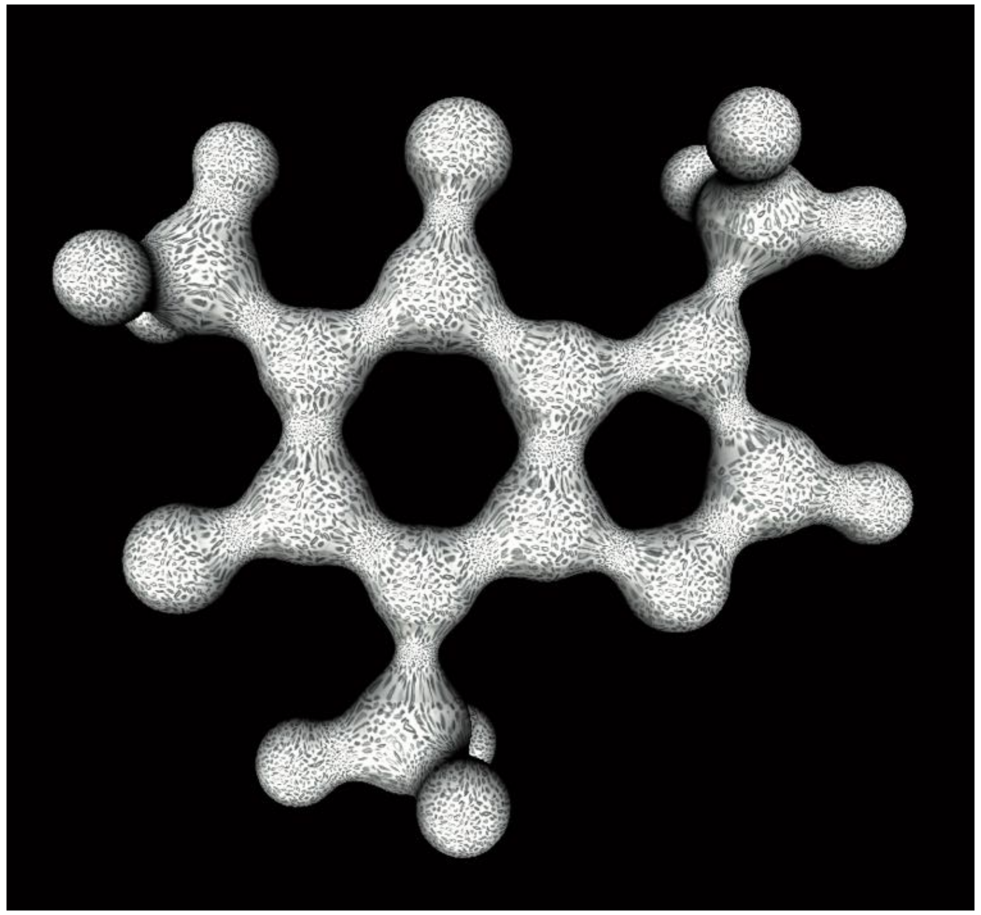

CHARGE

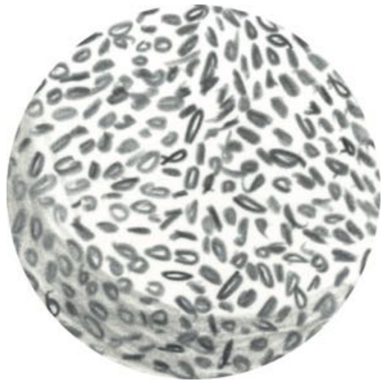

EVE NAGY

LIT-SPHÈRES

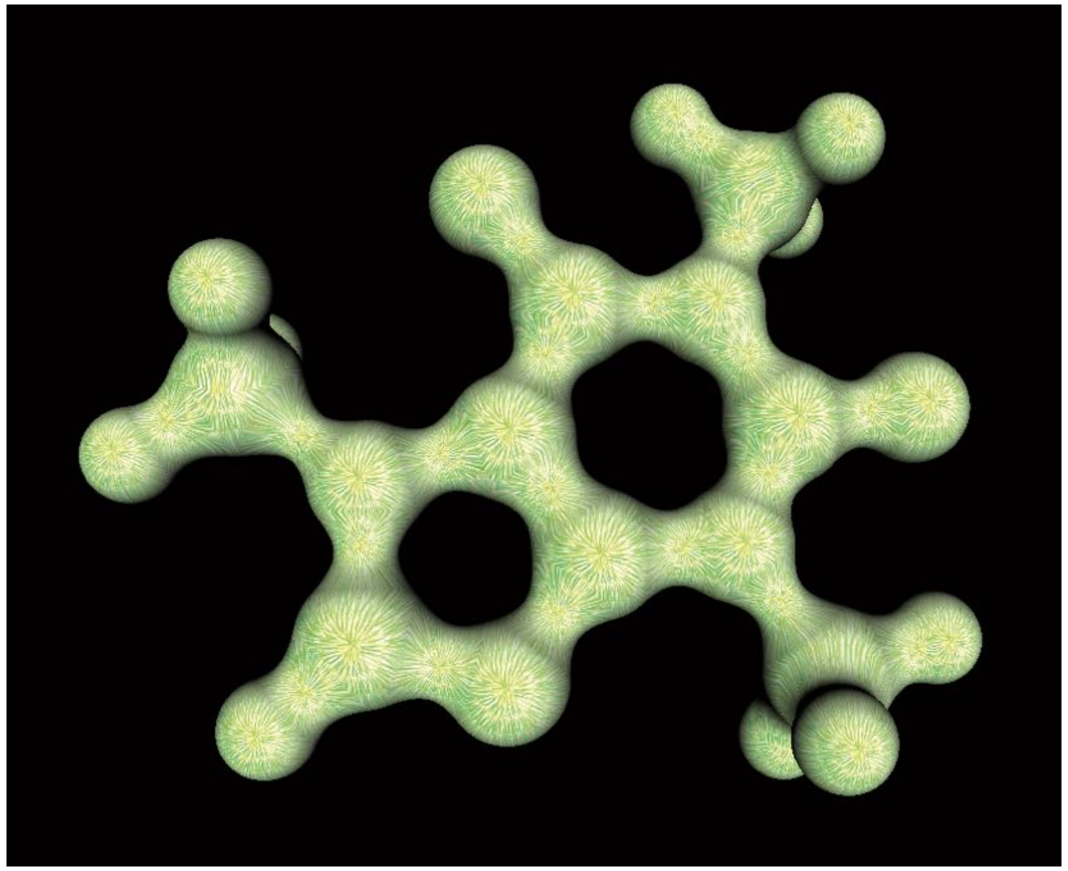

SAIN

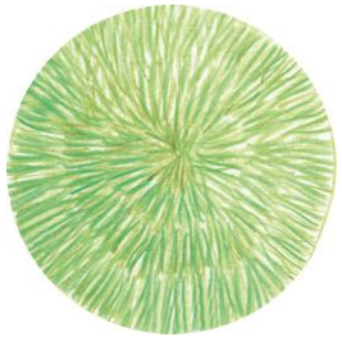

EVE NAGY

LIT-SPHÈRES

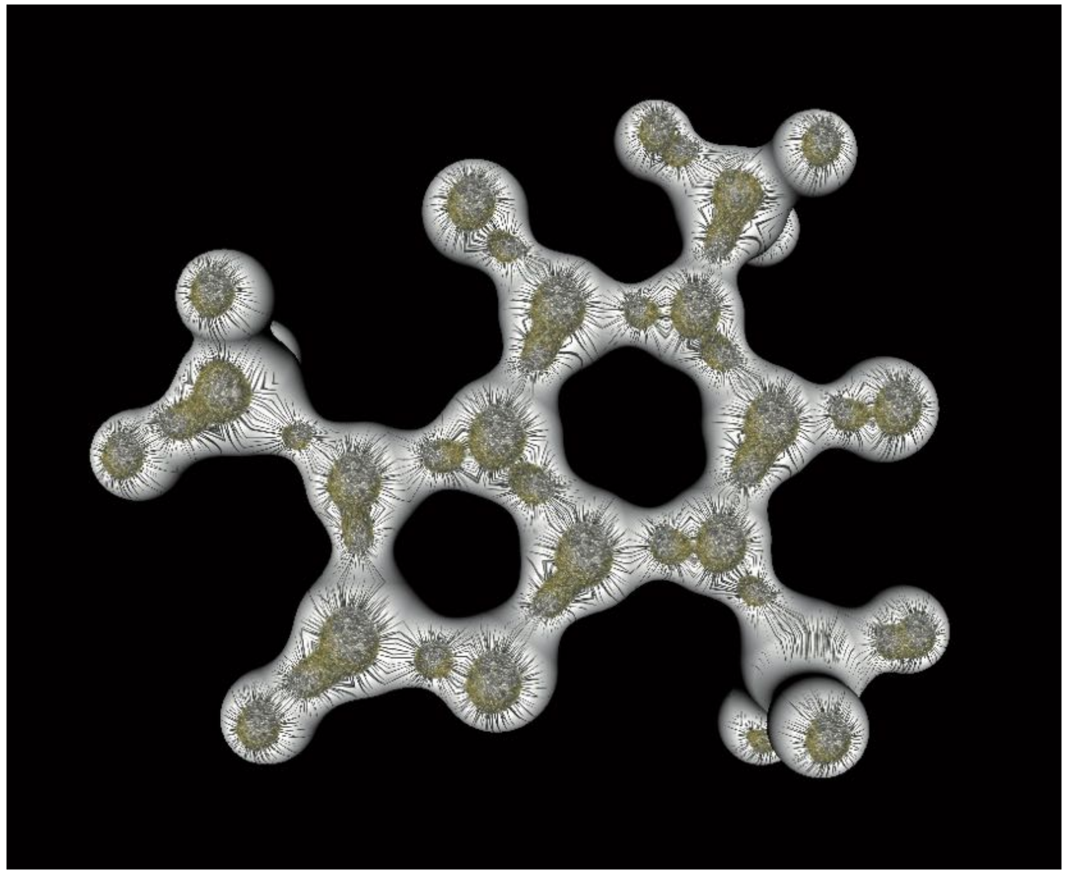

TOXIQUE

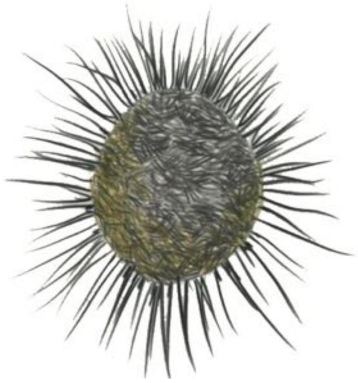

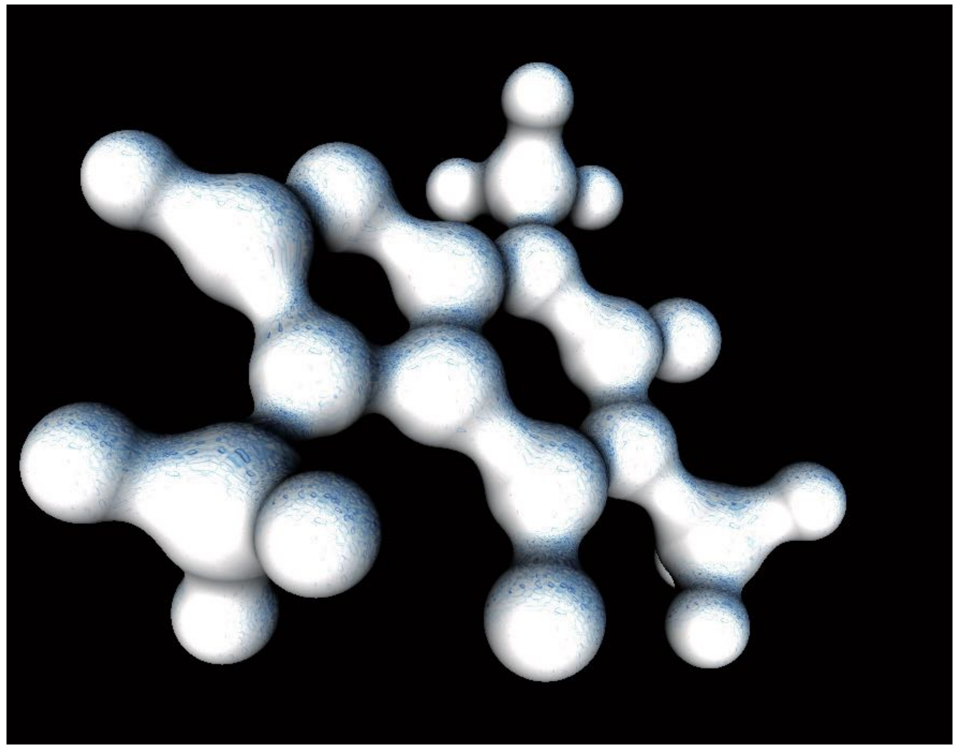

OXYGÈNE

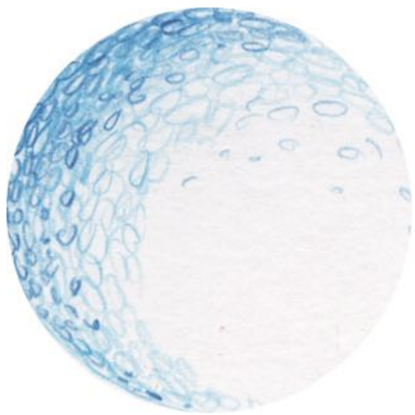

EVE NAGY

LIT-SPHÈRES

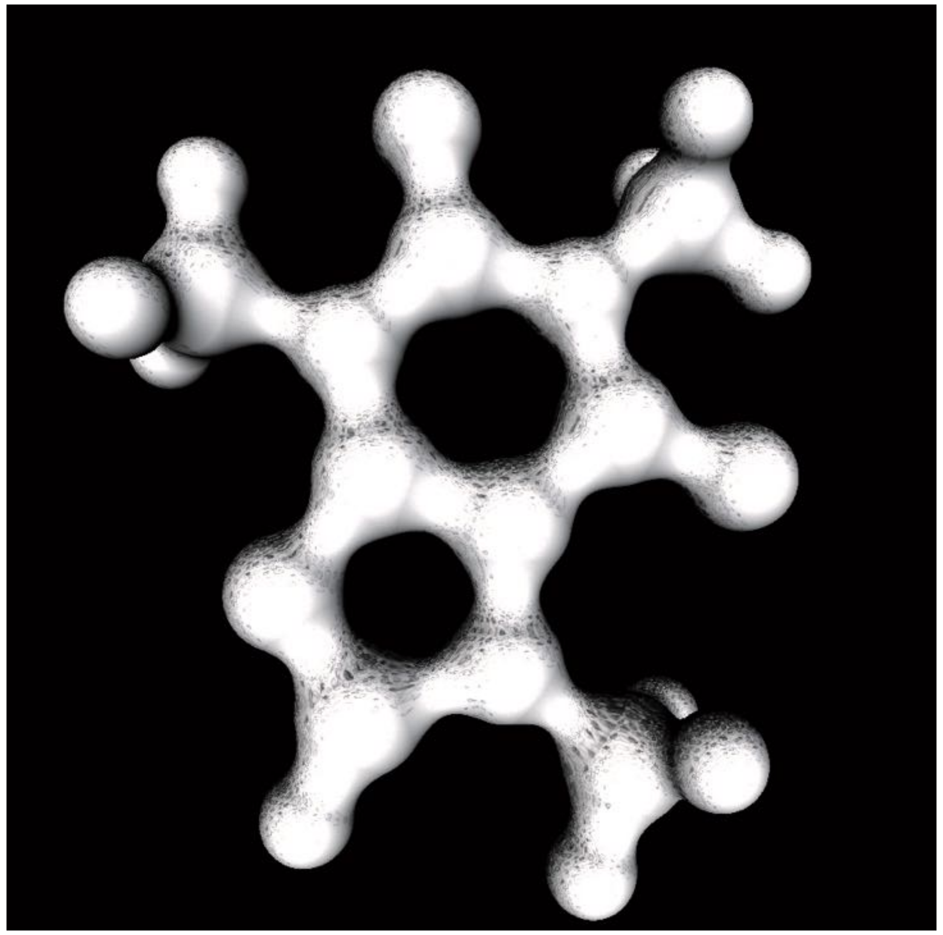

CARBONE

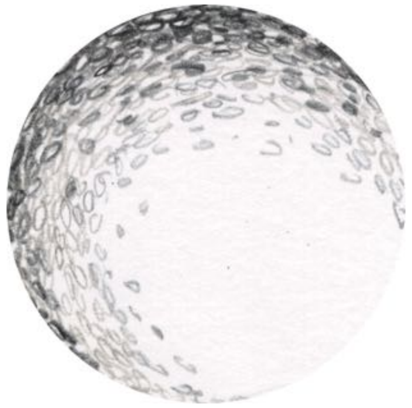

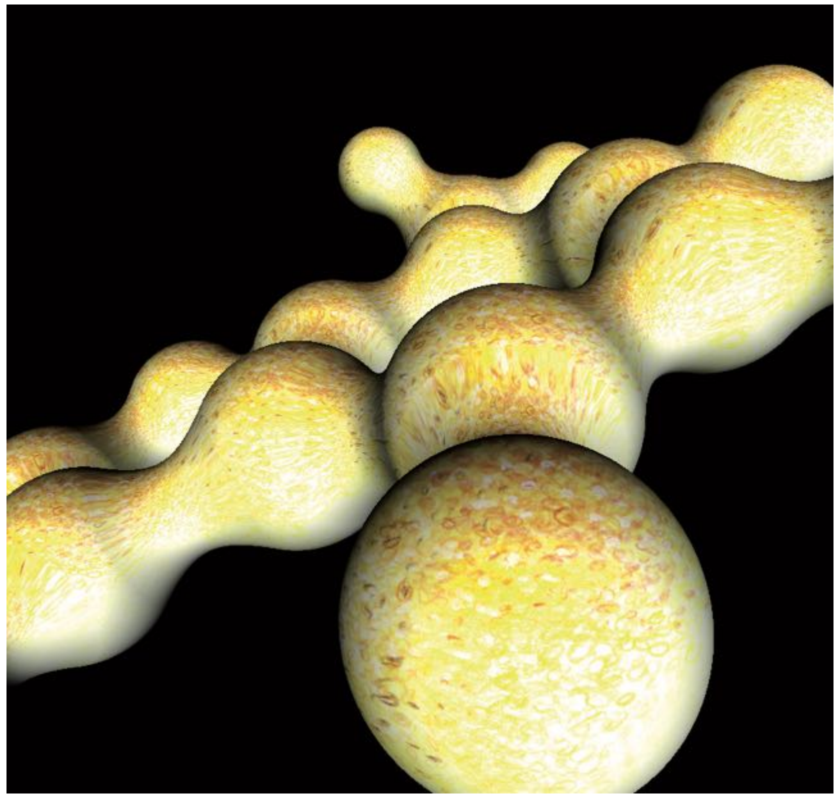

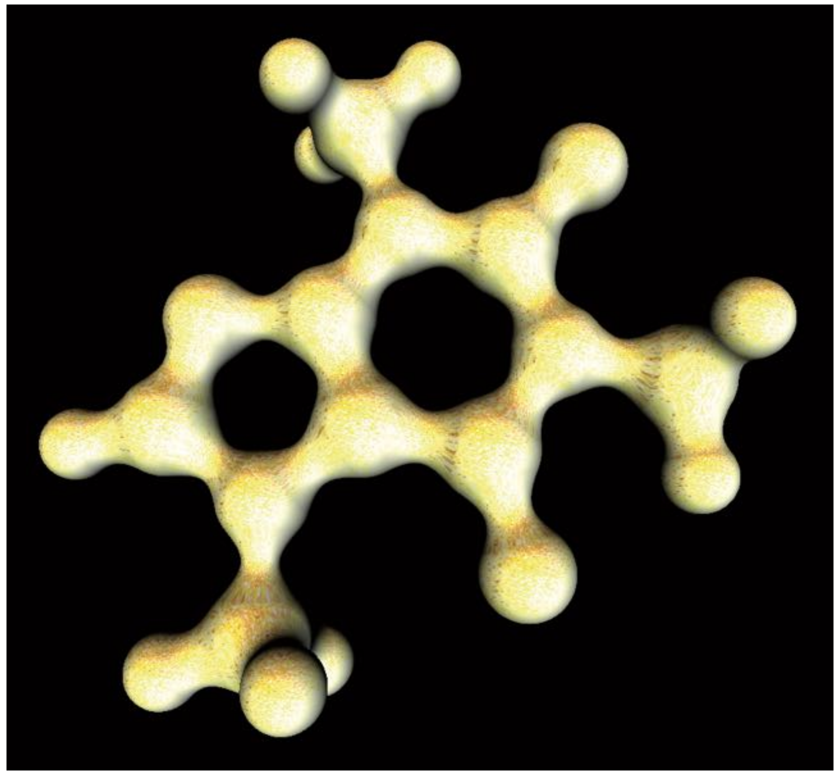

SUCRE

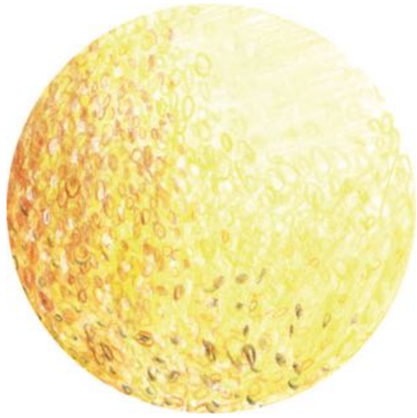

EVE NAGY

LIT-SPHÈRES

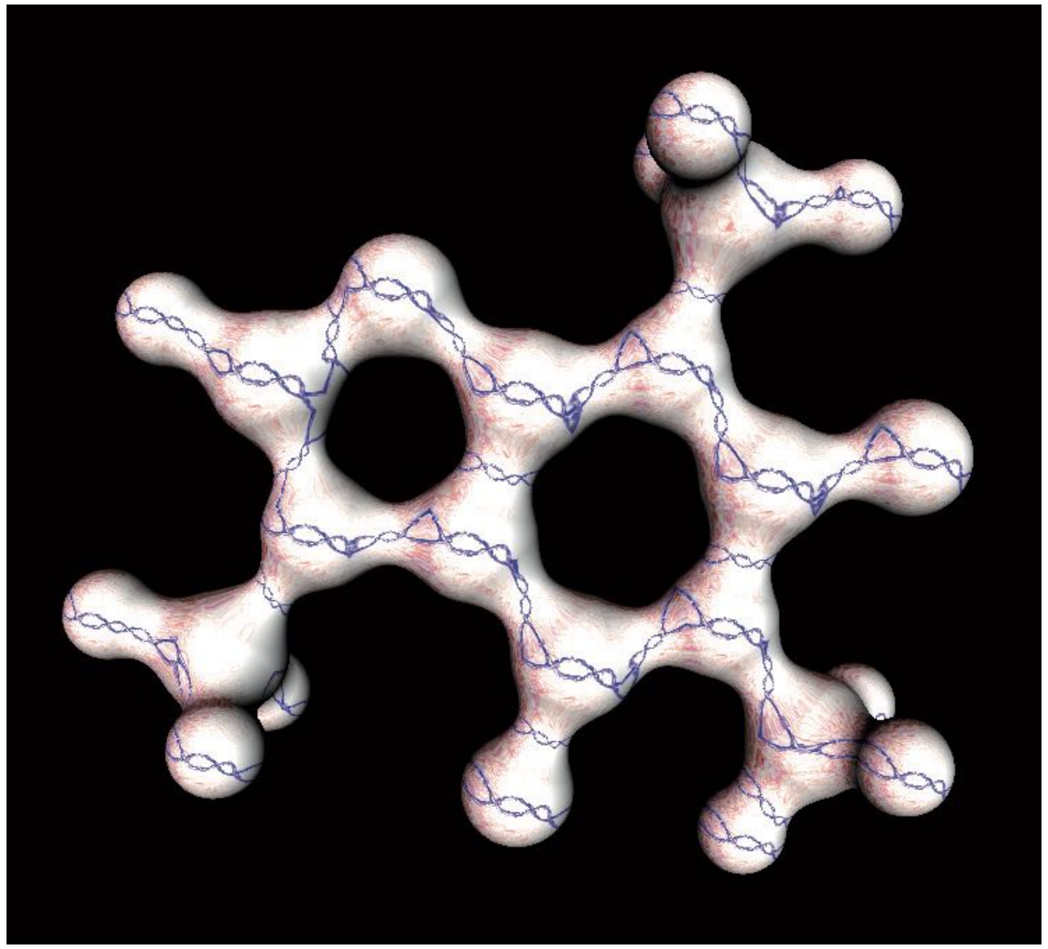

ADN

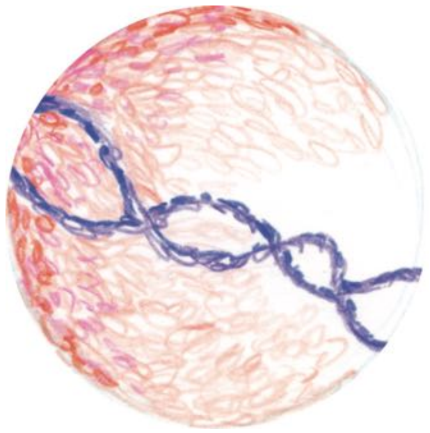

EVE NAGY

LIT-SPHÈRES

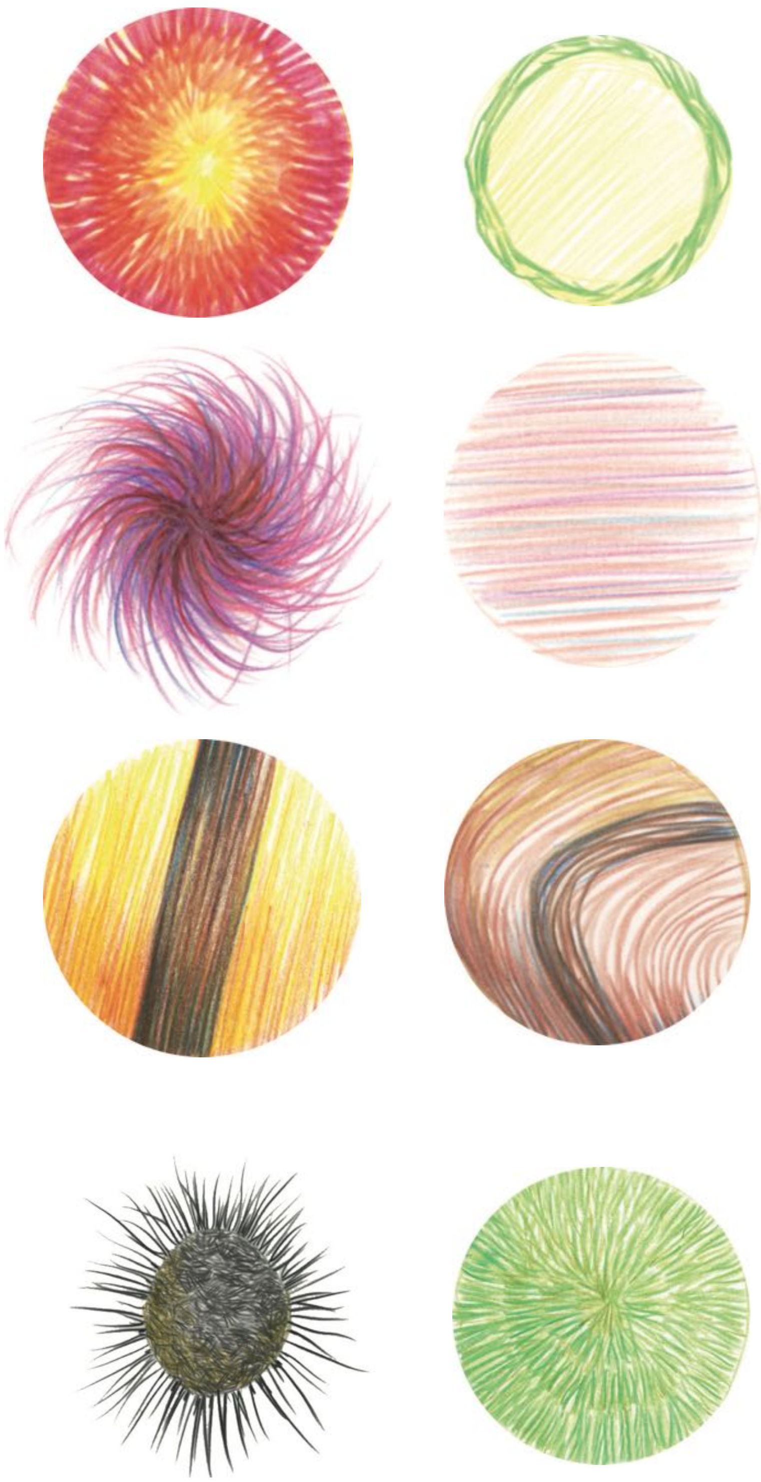

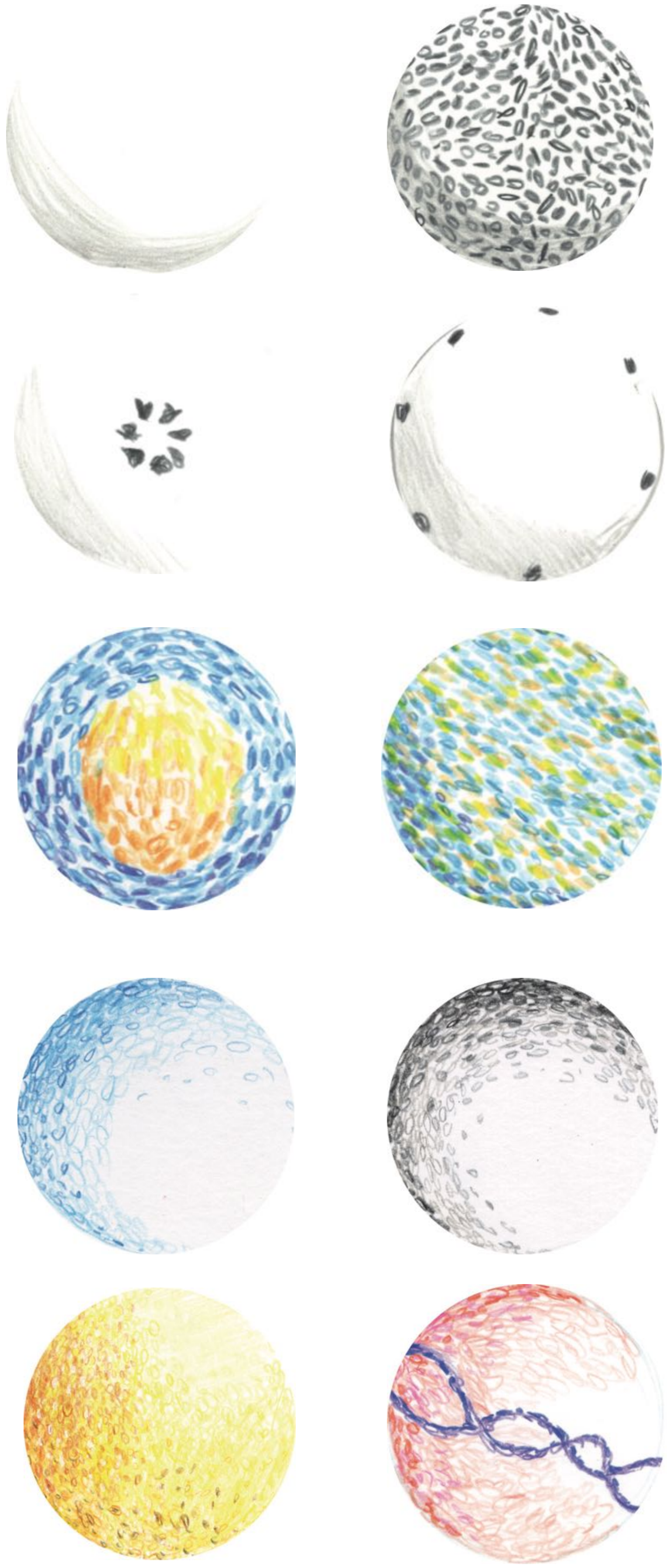

«LIT-SPHÈRE»

AXE SUR LE PRINCIPE DE L'ILLUSION PERSPECTIVE.
(RESTITUTION DES VOLUMES PAR
LES OMBRES PORTÉES ET LES OMBRES PROPRES)
REPRÉSENTATION DE L'INVISIBLE.
PETITS ÉLÉMENTS GÉOMÉTRIQUE.

CLAIRE THIBON

ÉCOCOMPATIBLE / TOXIQUE

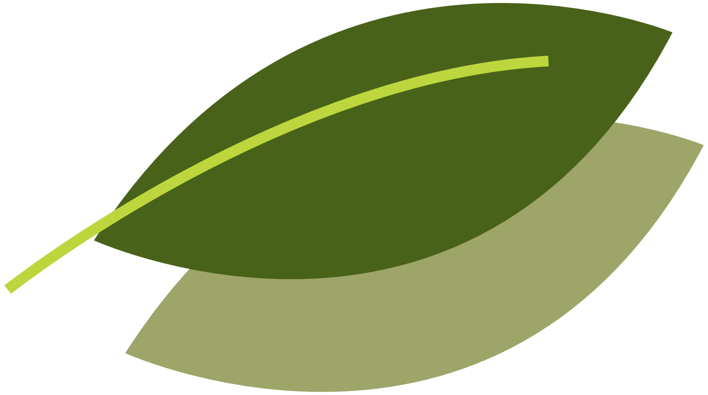

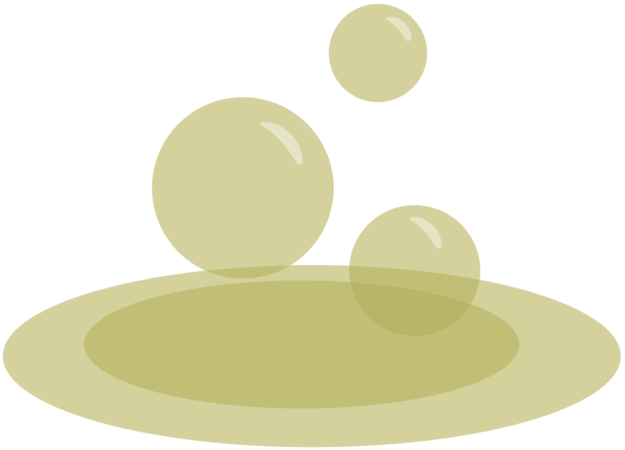

CLAIRE THIBON

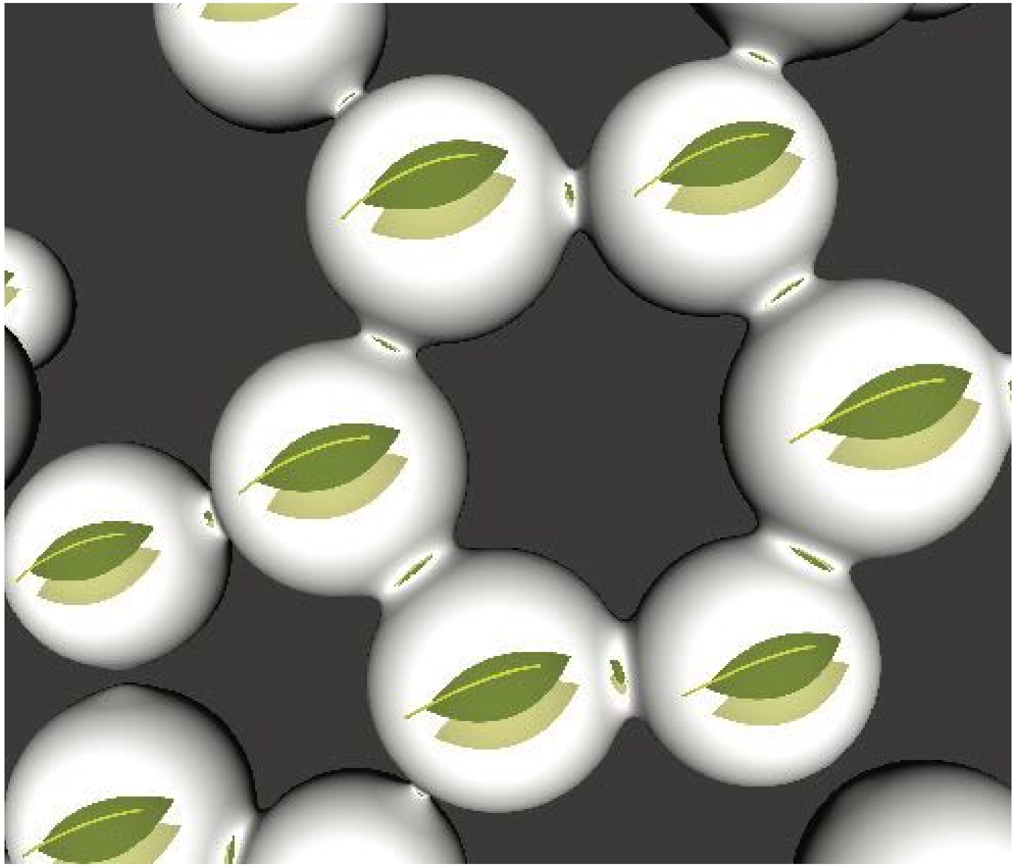

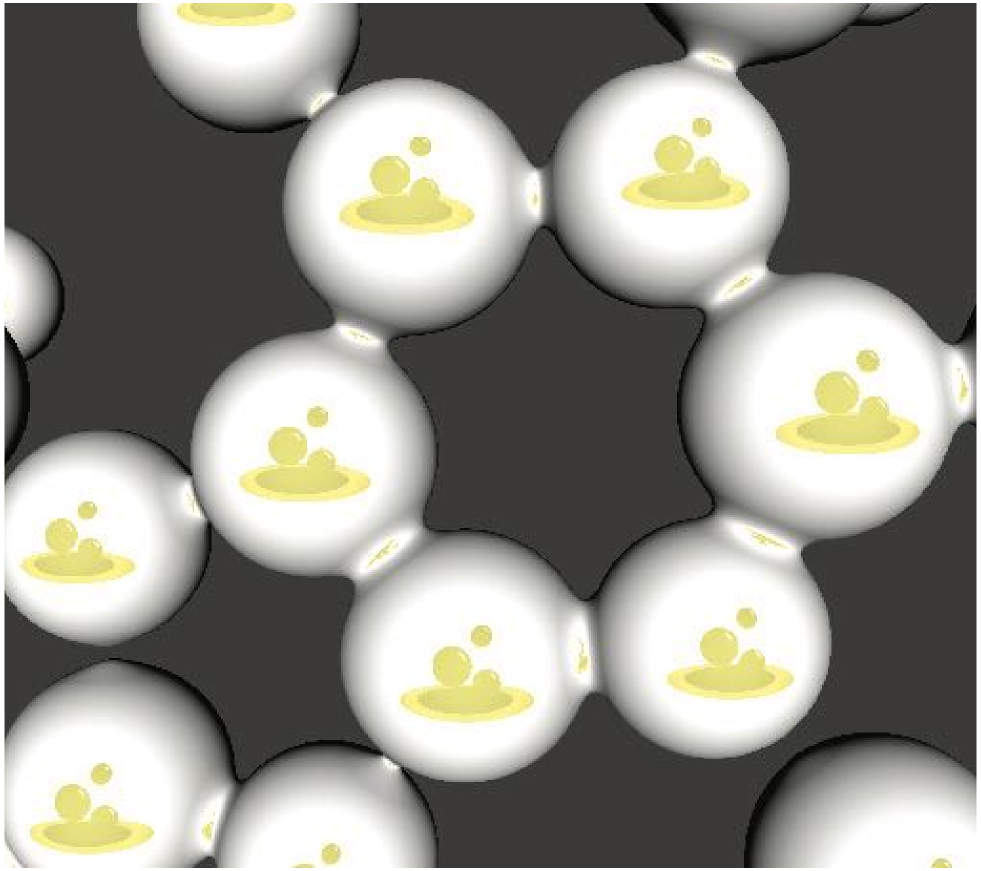

CLAIRE THIBON

ACTIF

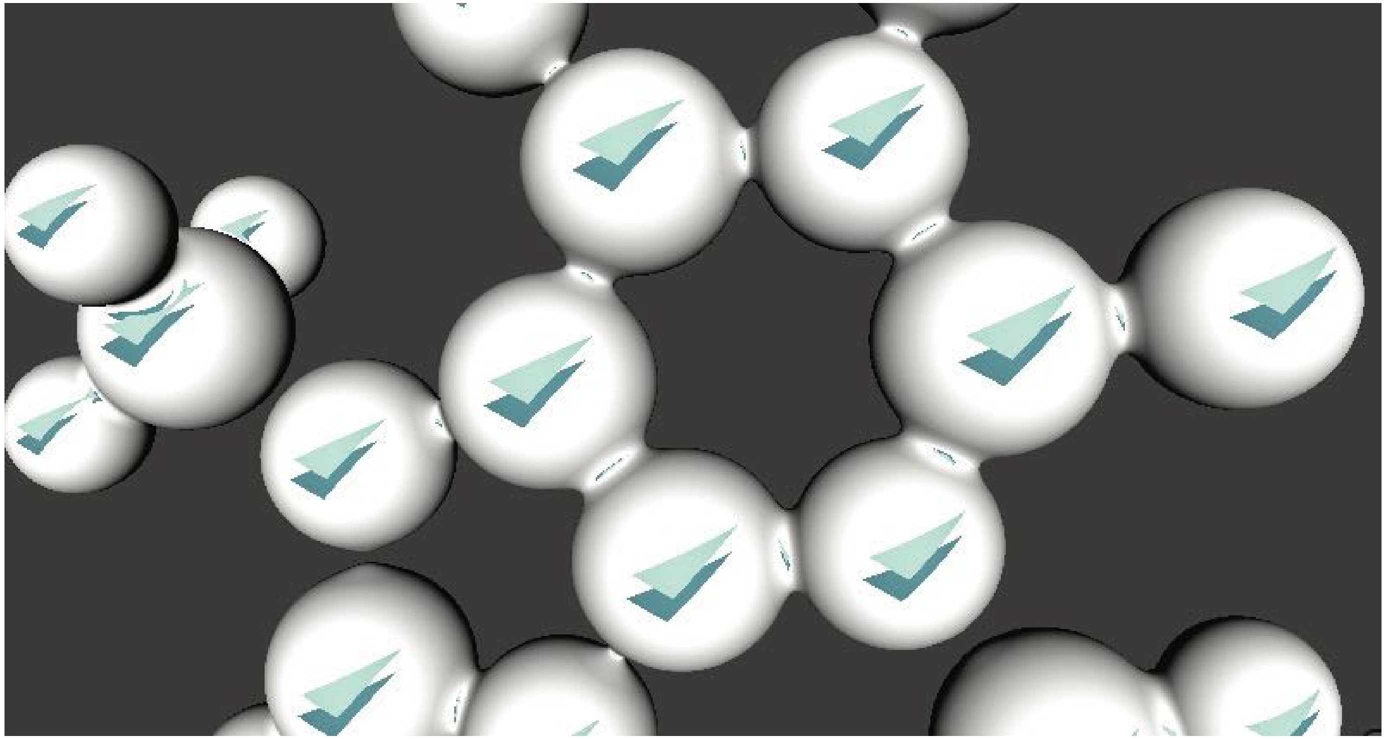

CLAIRE THIBON

BON / MAUVAIS

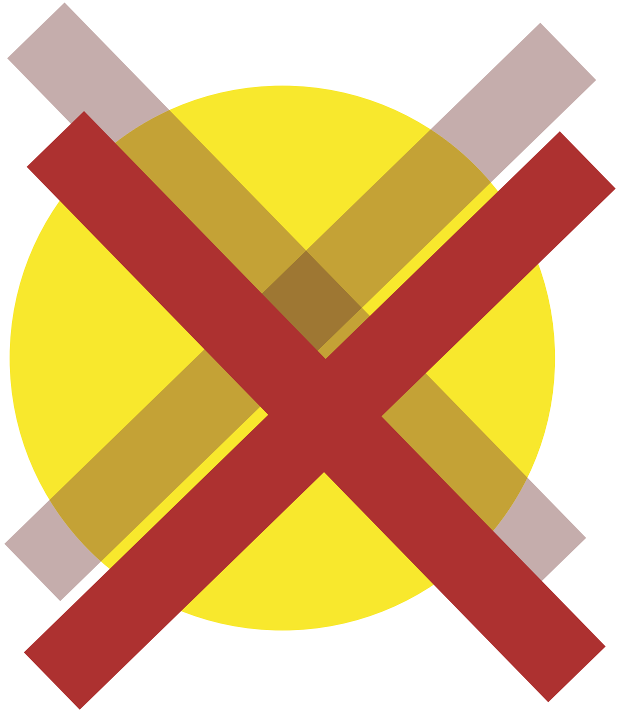

CLAIRE THIBON

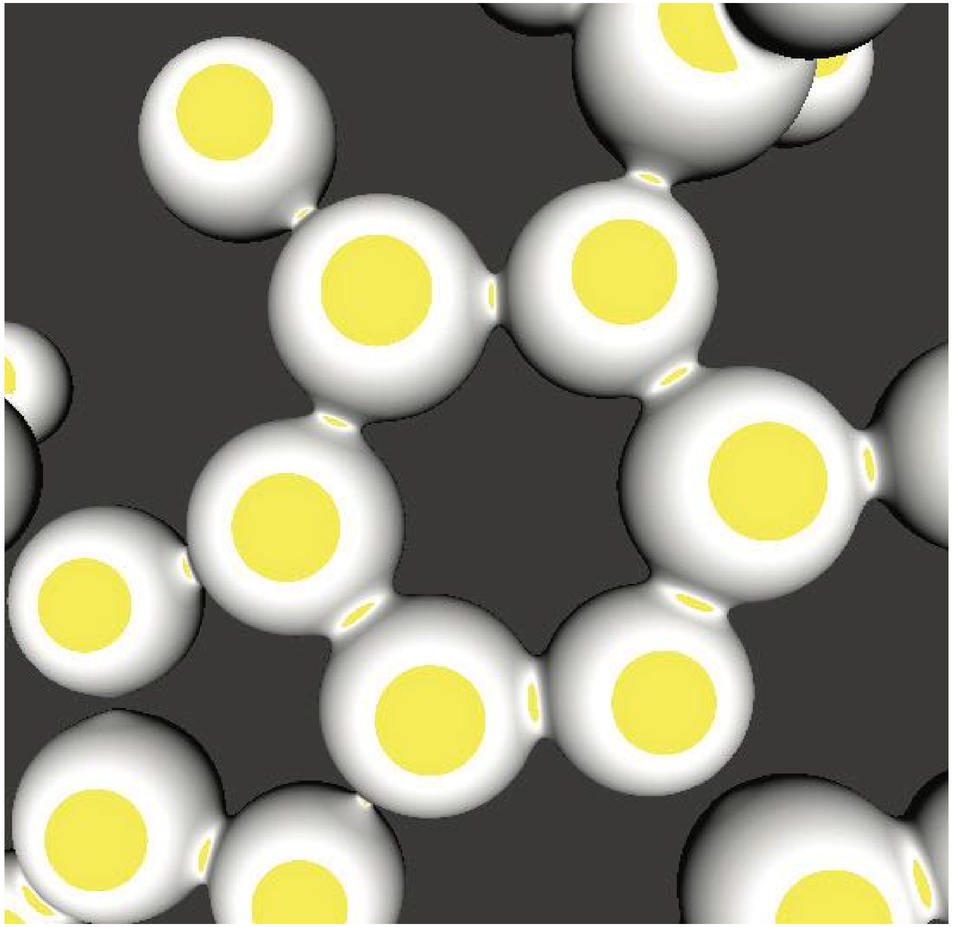

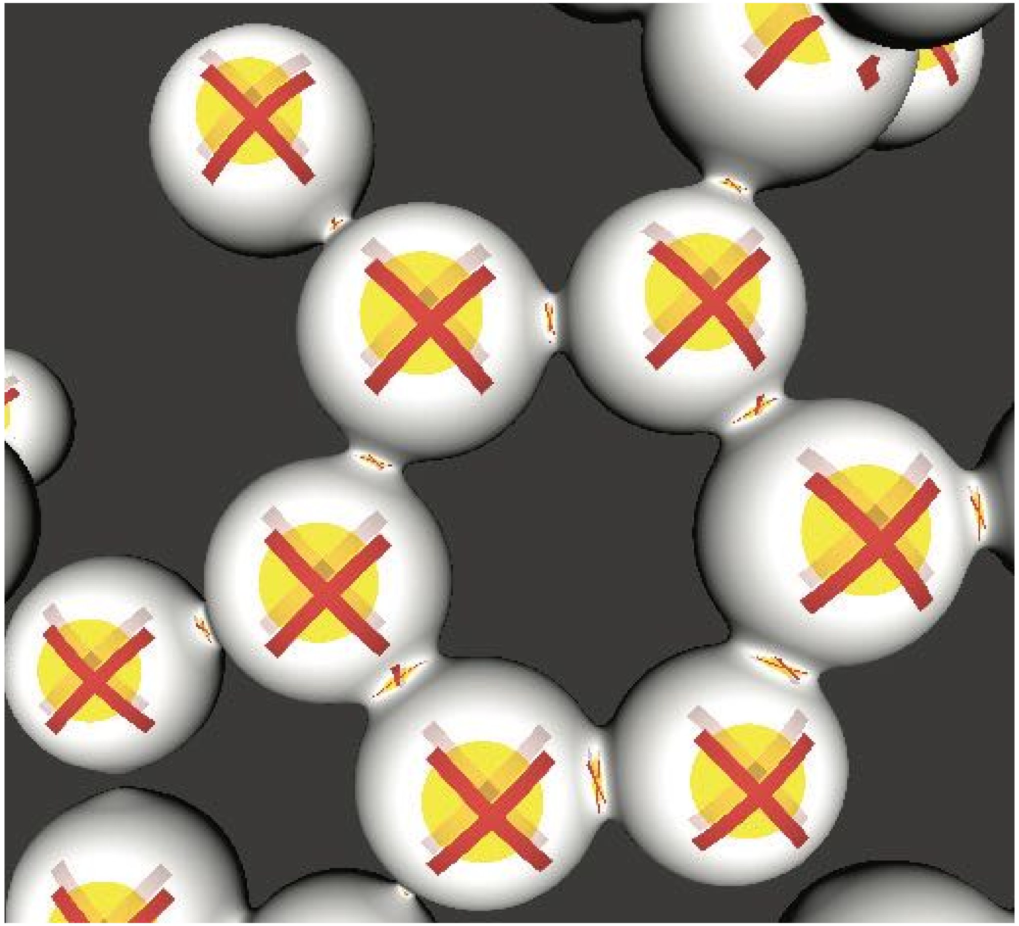

CLAIRE THIBON

POSITIF / NÉGATIF

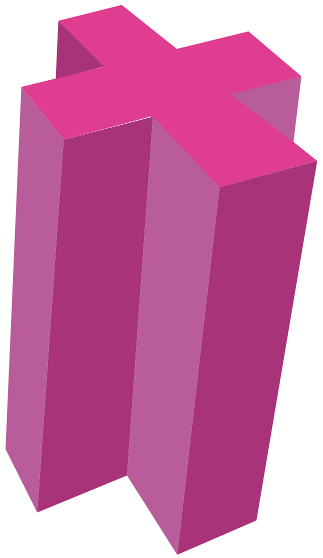

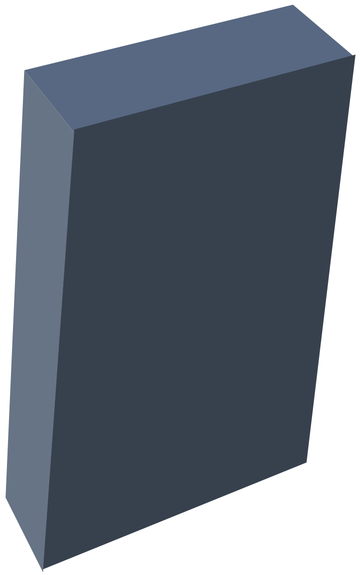

CLAIRE THIBON

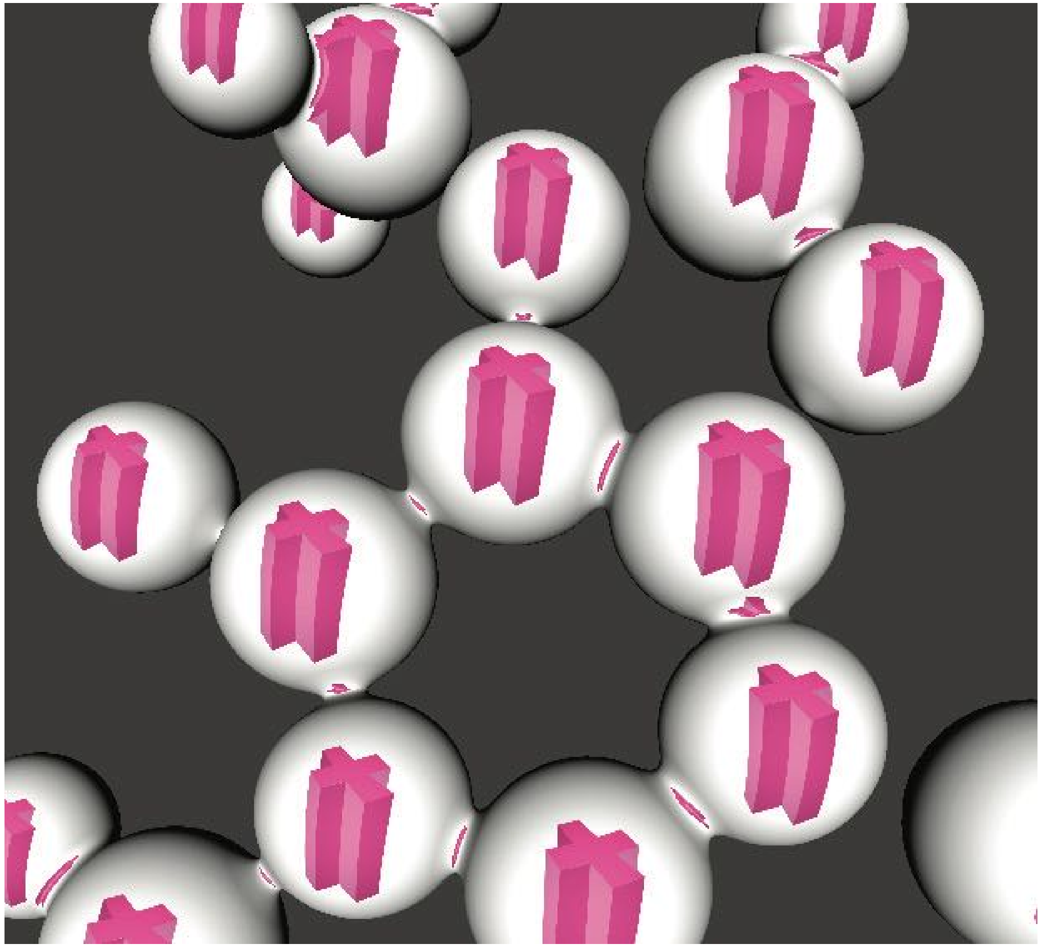

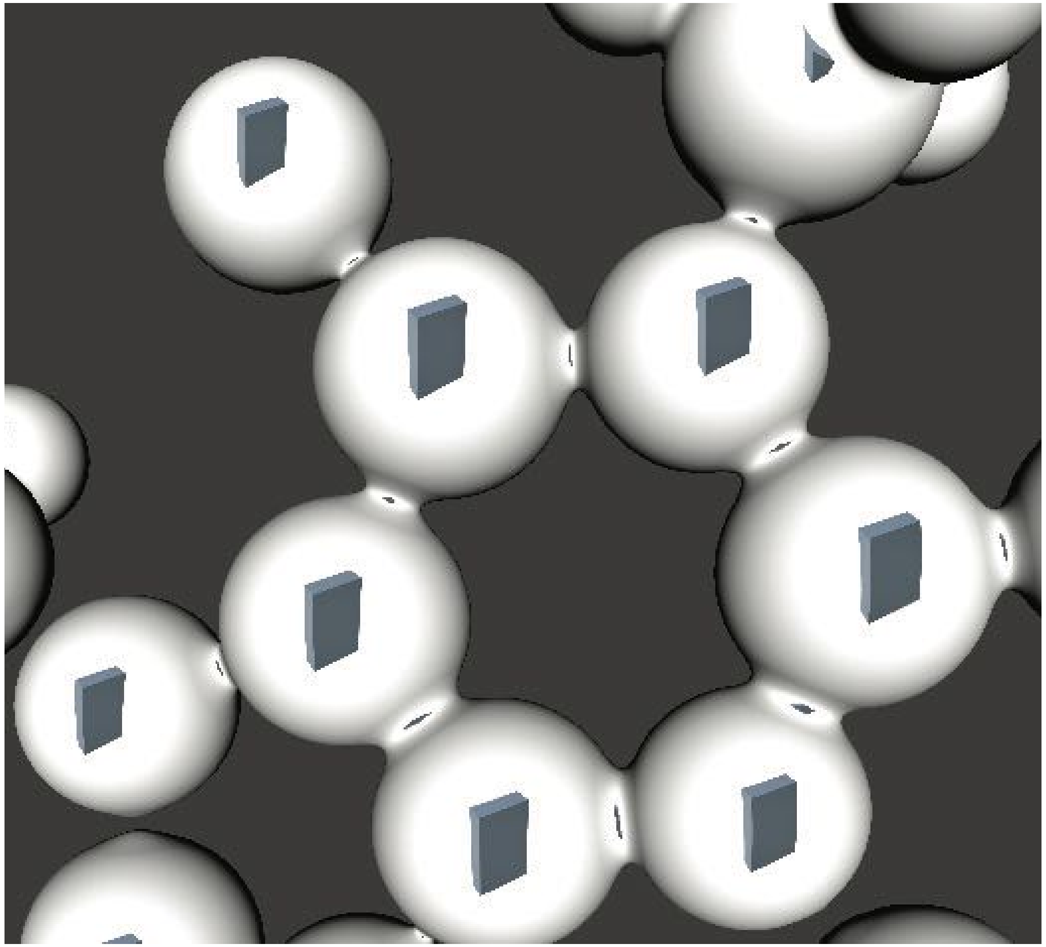

CLAIRE THIBON

RIGIDE / FLEXIBLE

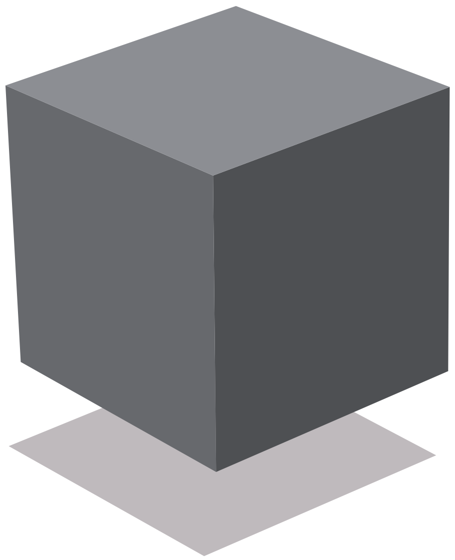

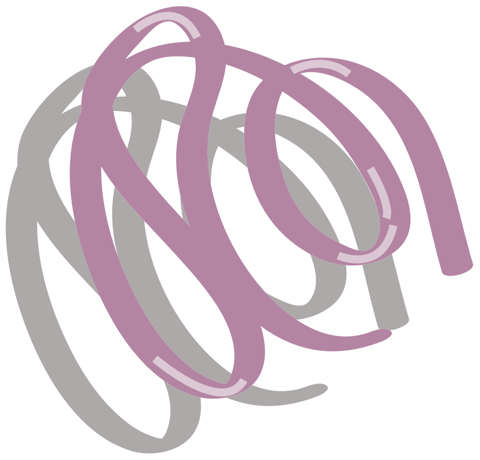

CLAIRE THIBON

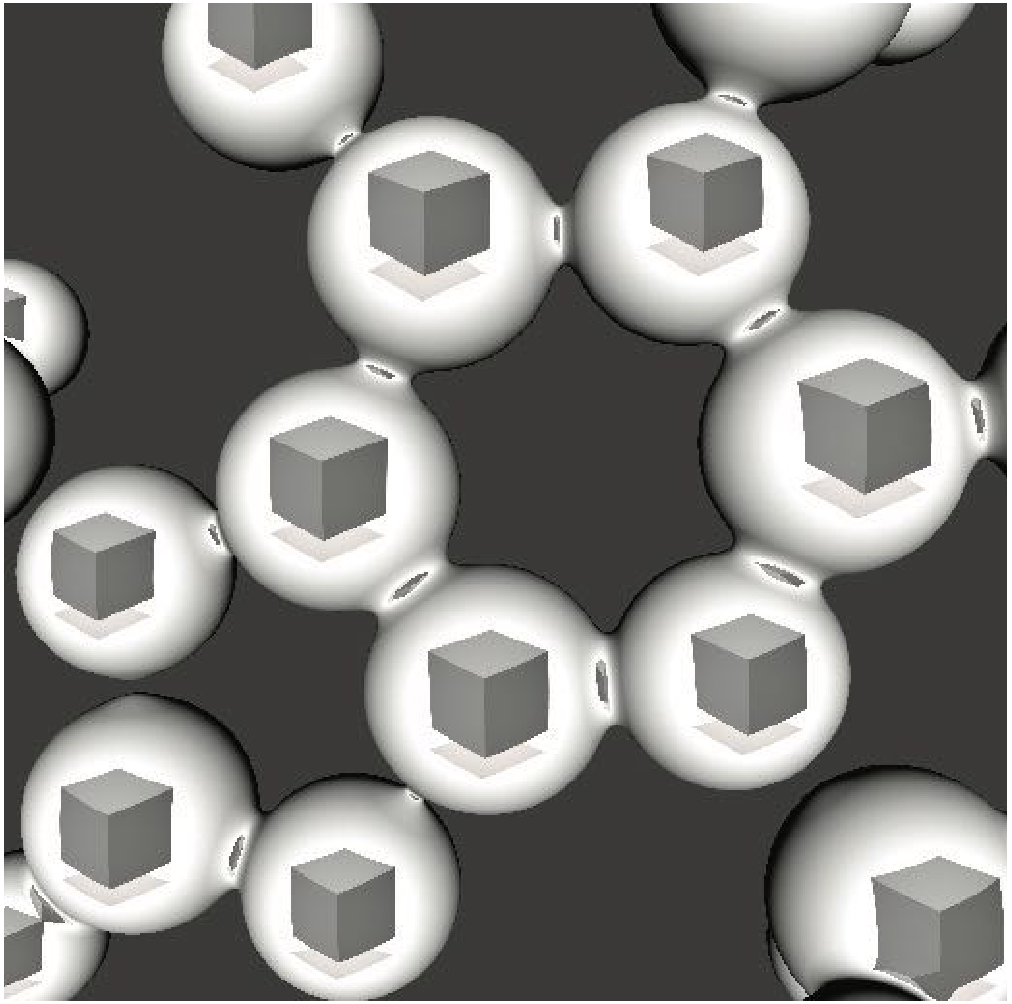

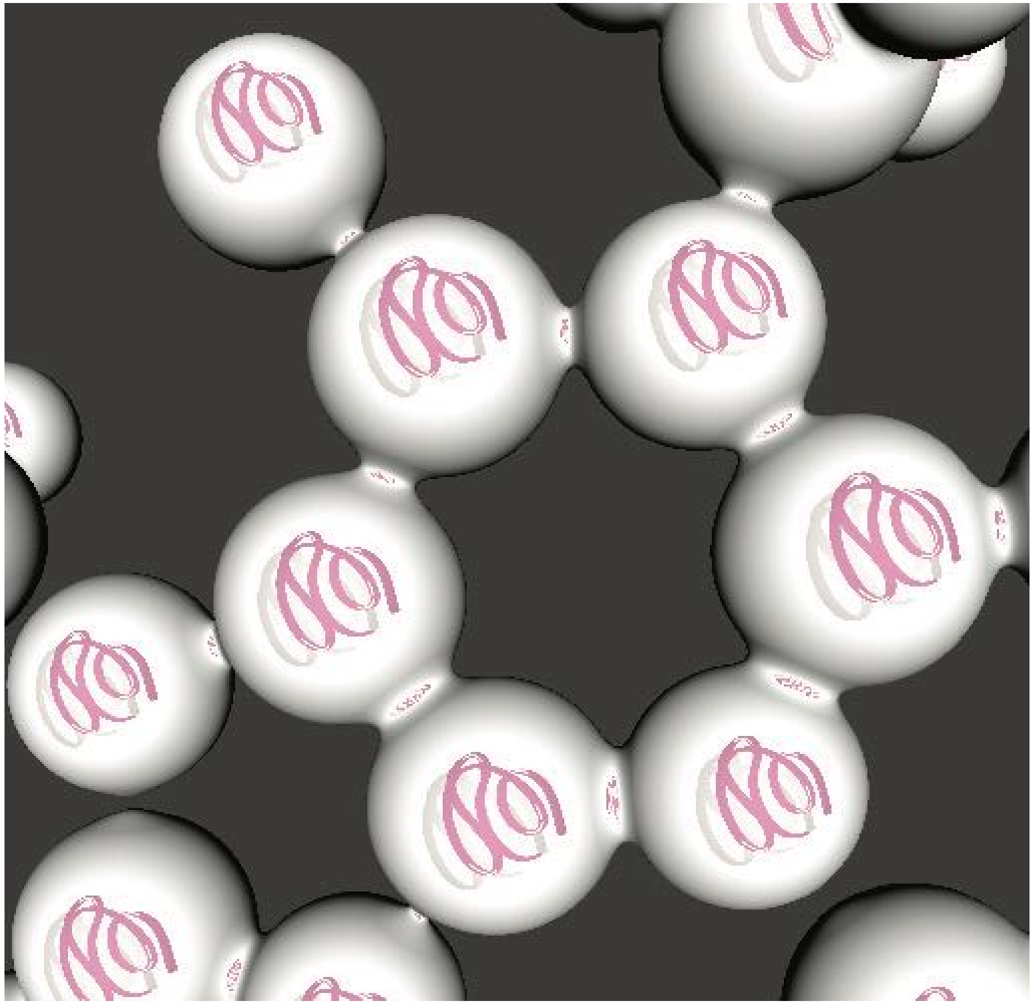

CLAIRE THIBON

HYDROPHOBE / HYDROPHILE

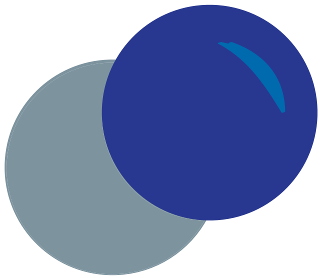

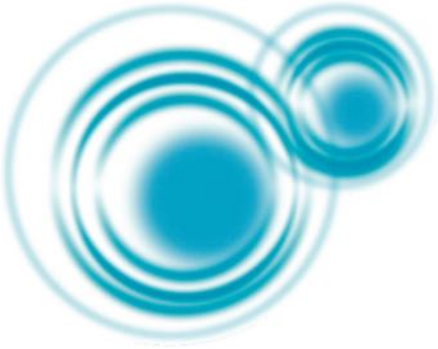

CLAIRE THIBON

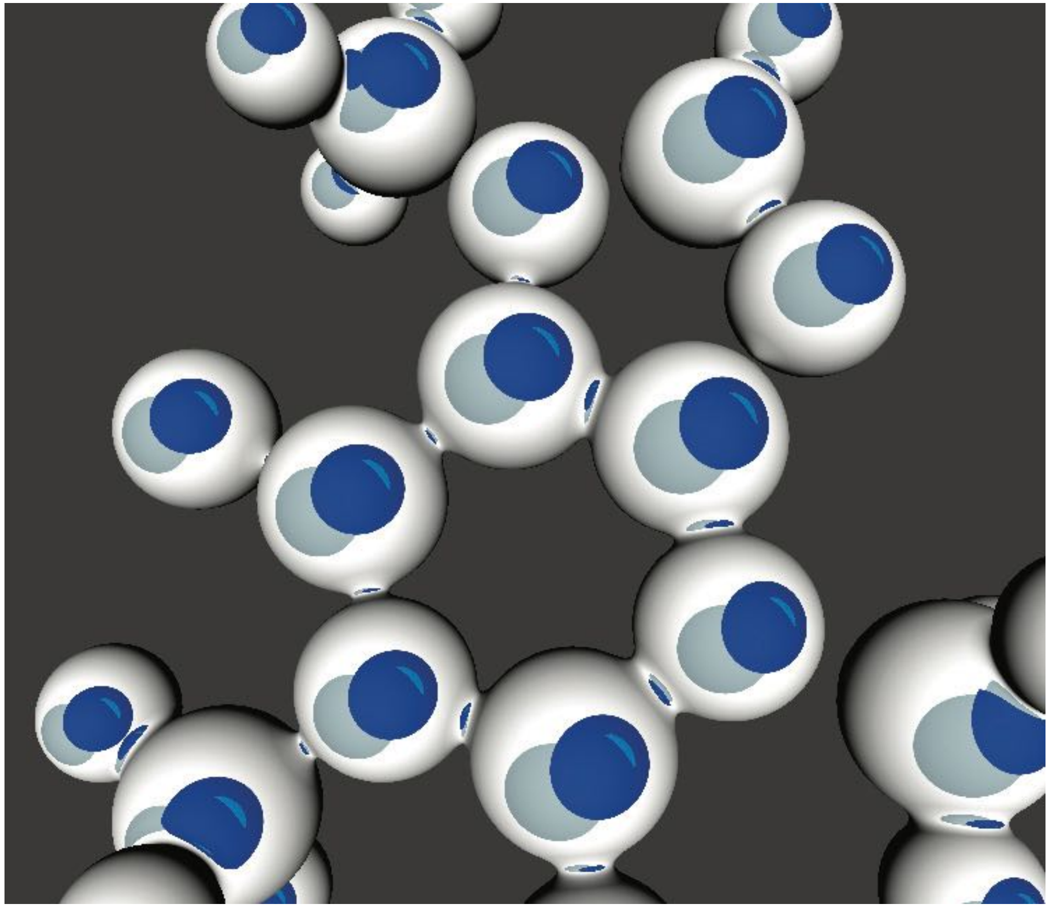

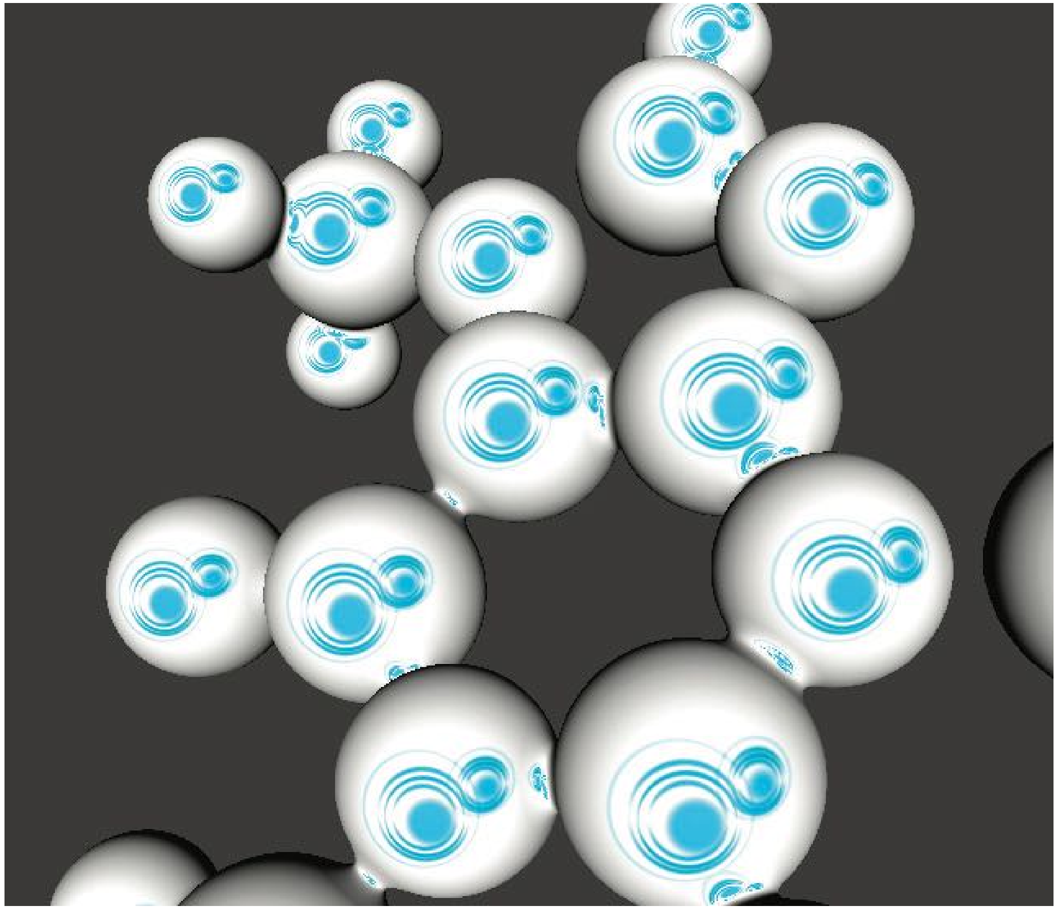

CLAIRE THIBON

NATURE DE LA MOLÉCULE
GRAISSE / SUCRE

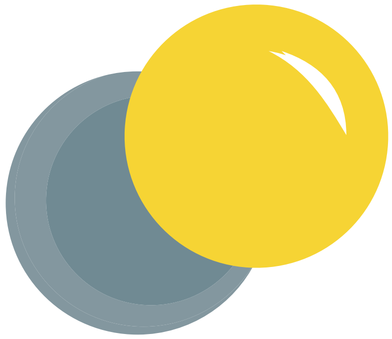

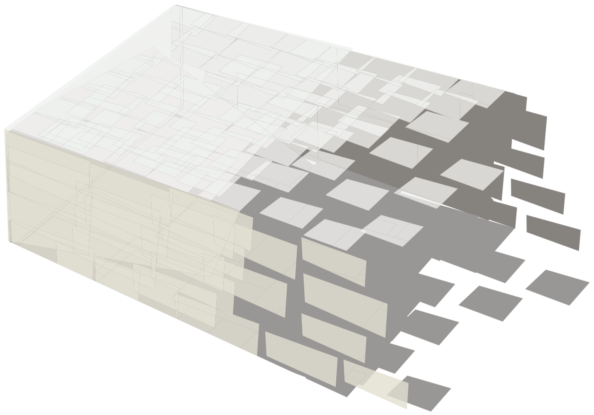

CLAIRE THIBON

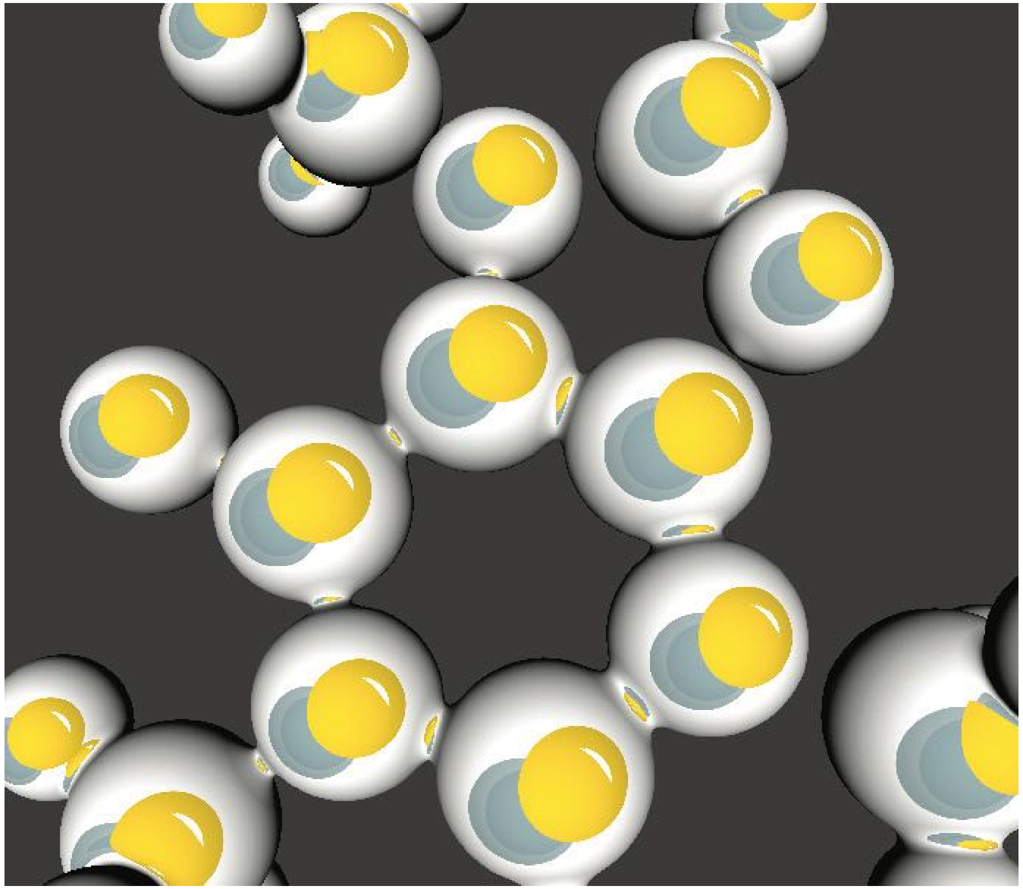

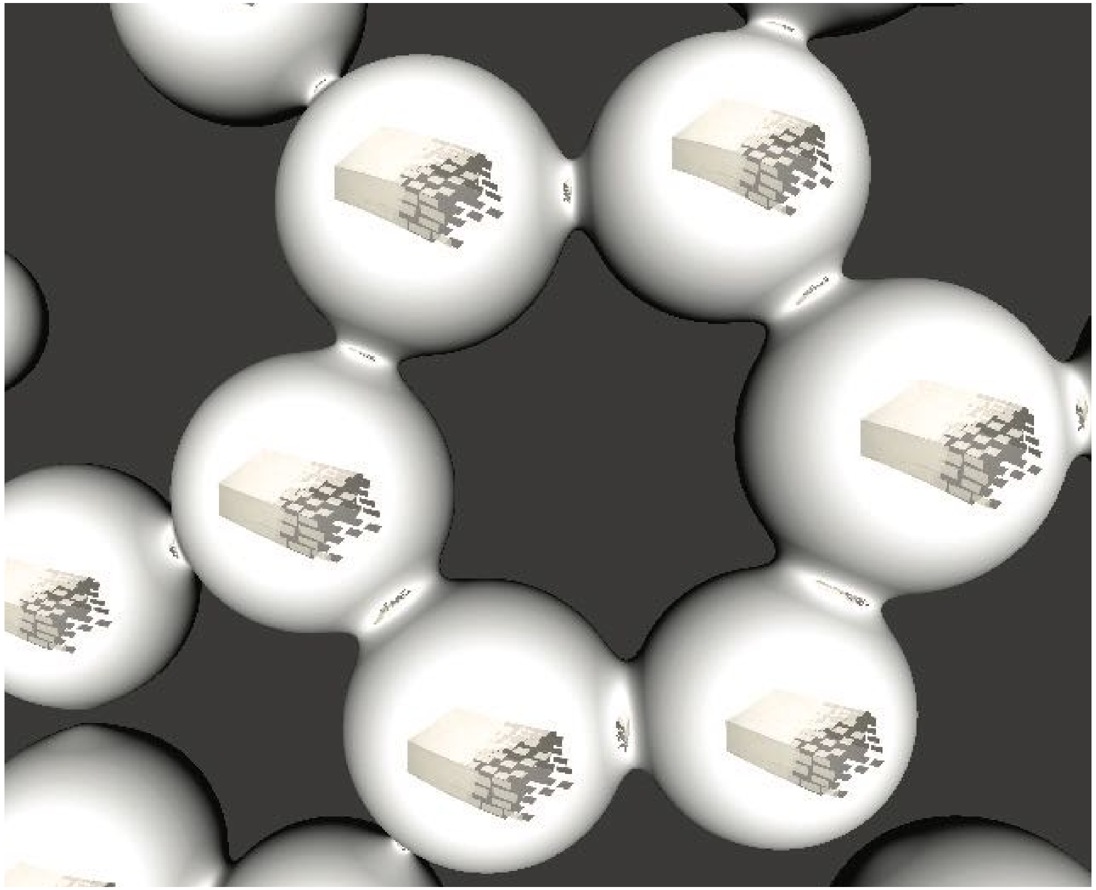

CLAIRE THIBON

NATURE DE L'ATOME
CARBONE / OXYGÈNE

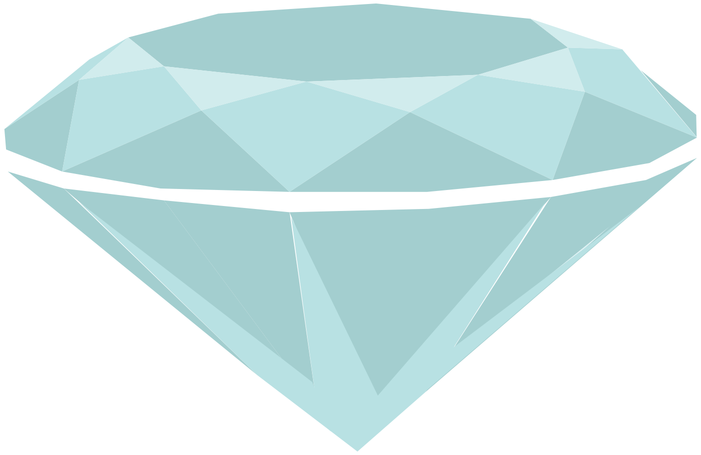

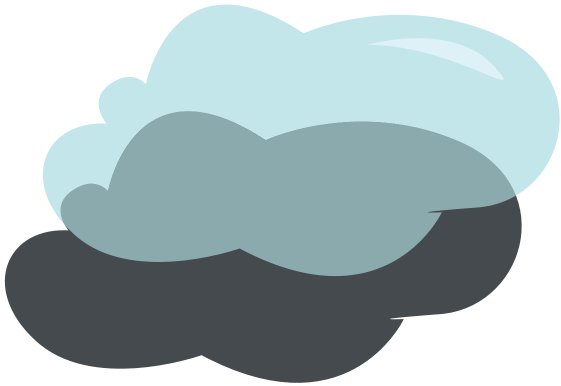

CLAIRE THIBON

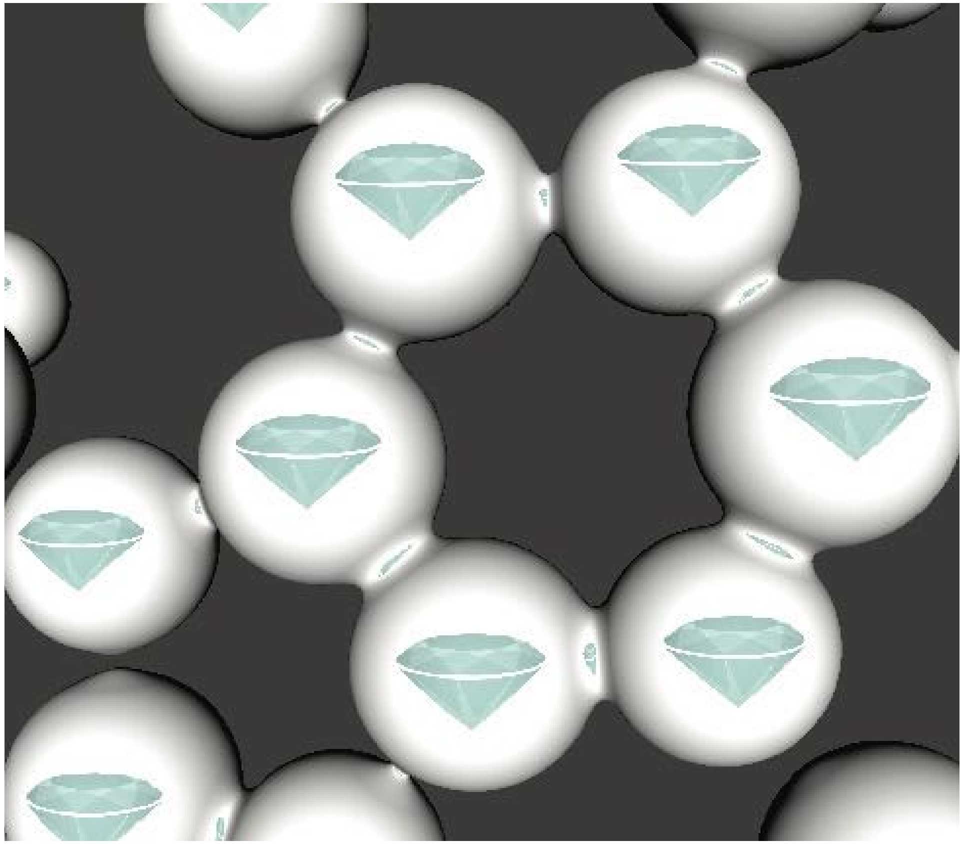

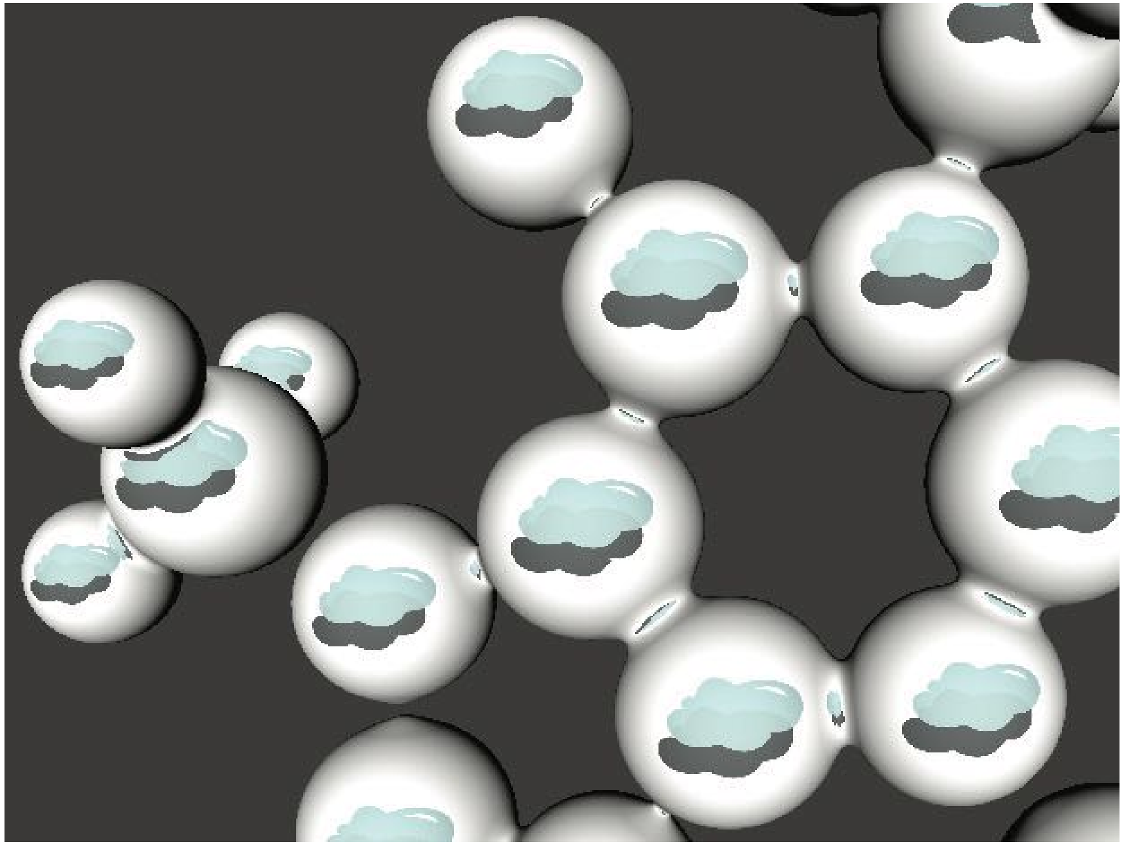

CLAIRE THIBON

QUANTITÉ
RÉPÉTITION

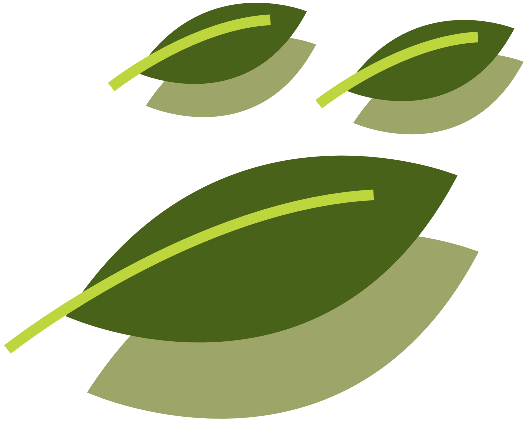

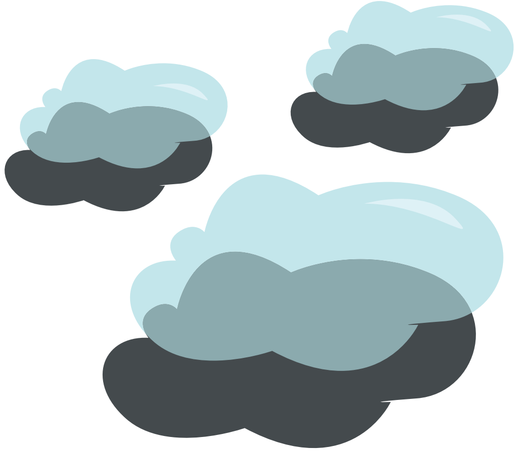

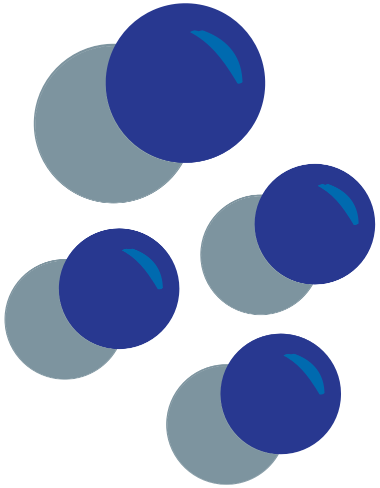

CLAIRE THIBON

CHANGEMENT DE TAILLE

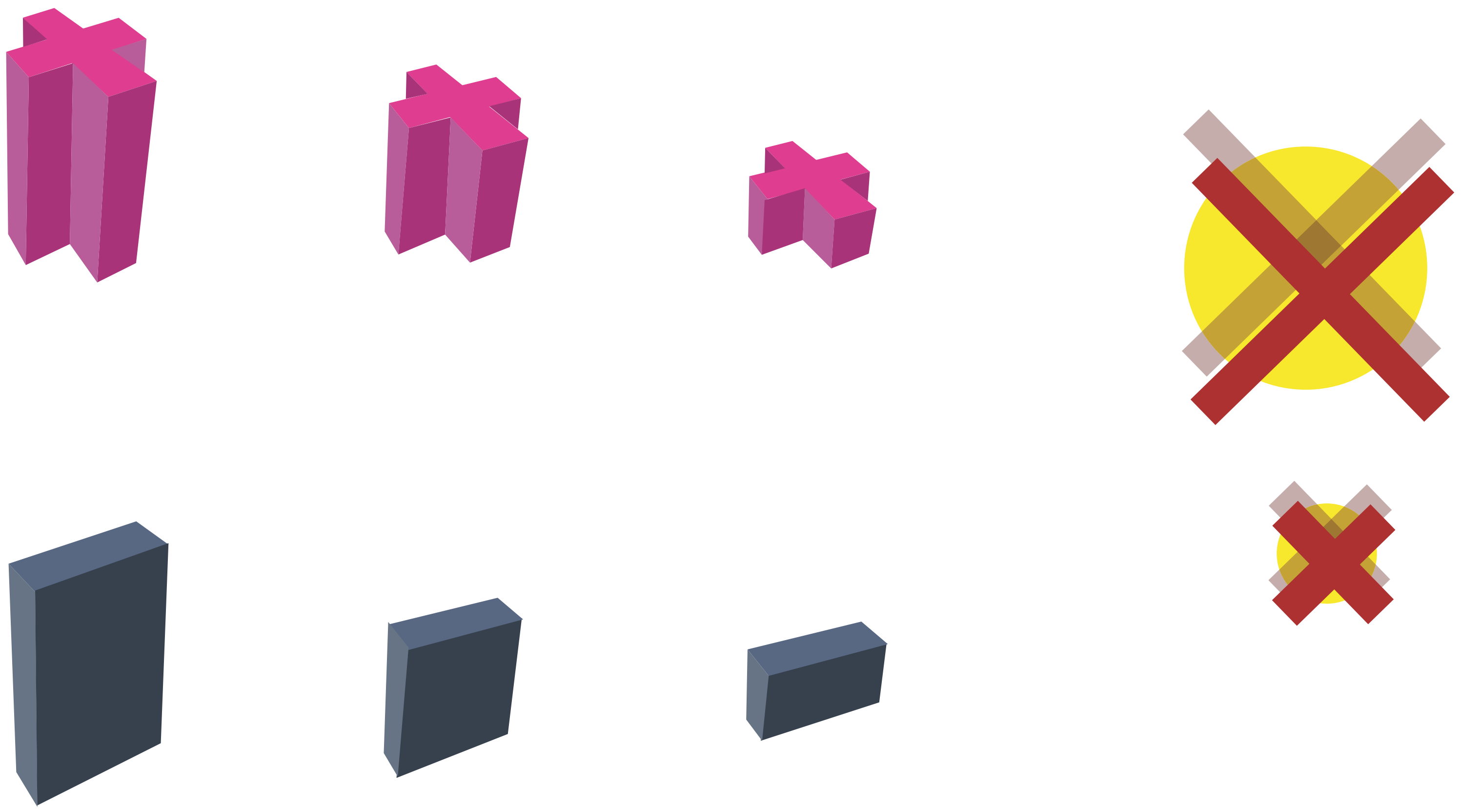

CLAIRE THIBON

CHANGEMENT
DANS LE VISUEL

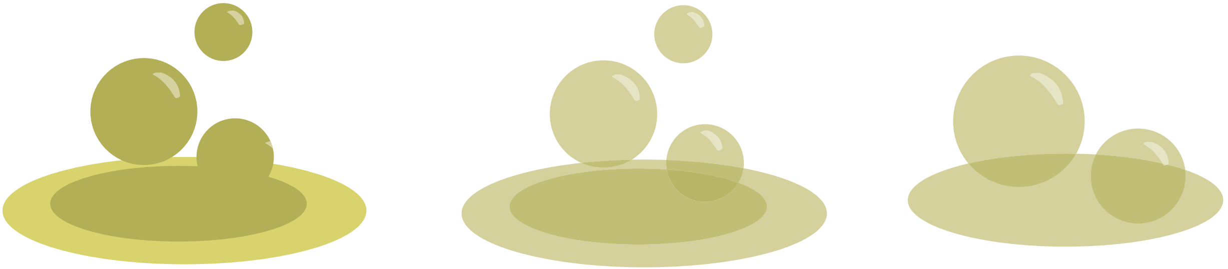

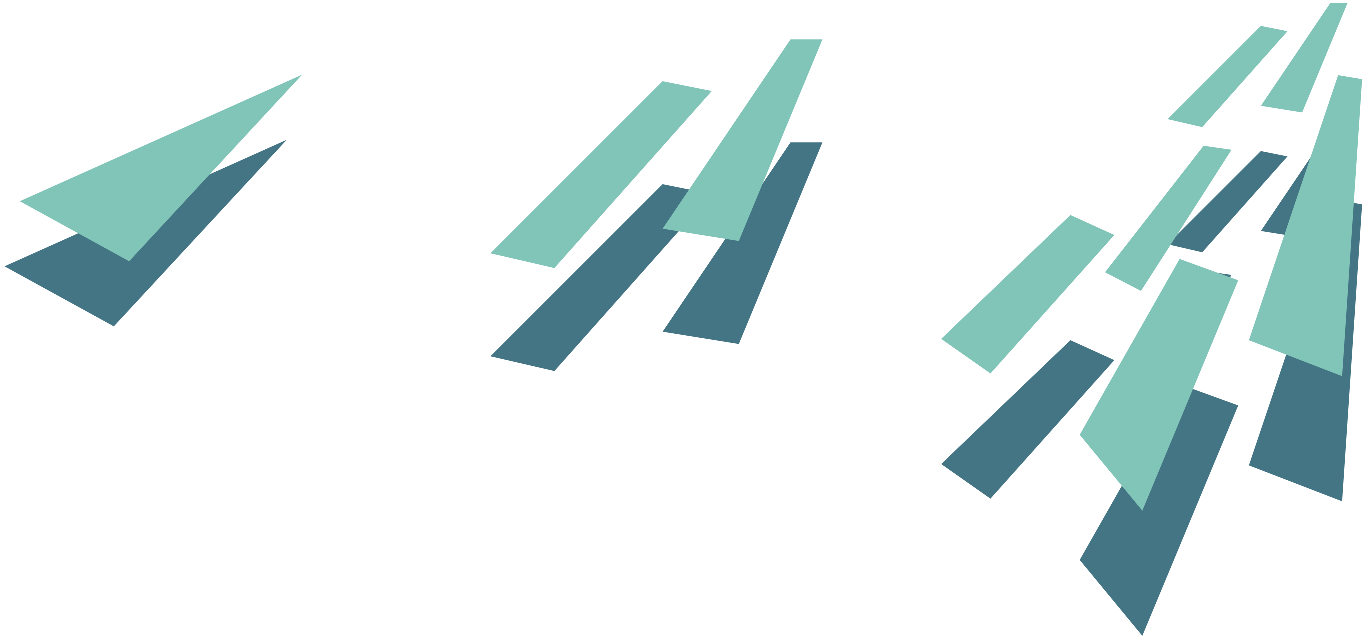

LA DERNIÈRE FOIS...

ESTELLE VILLEMIN

LIT-SPHÈRES

LA DERNIÈRE FOIS...

Rappel des recherches précédentes dans l'axe choisi : principe de strates en couches de papier découpé

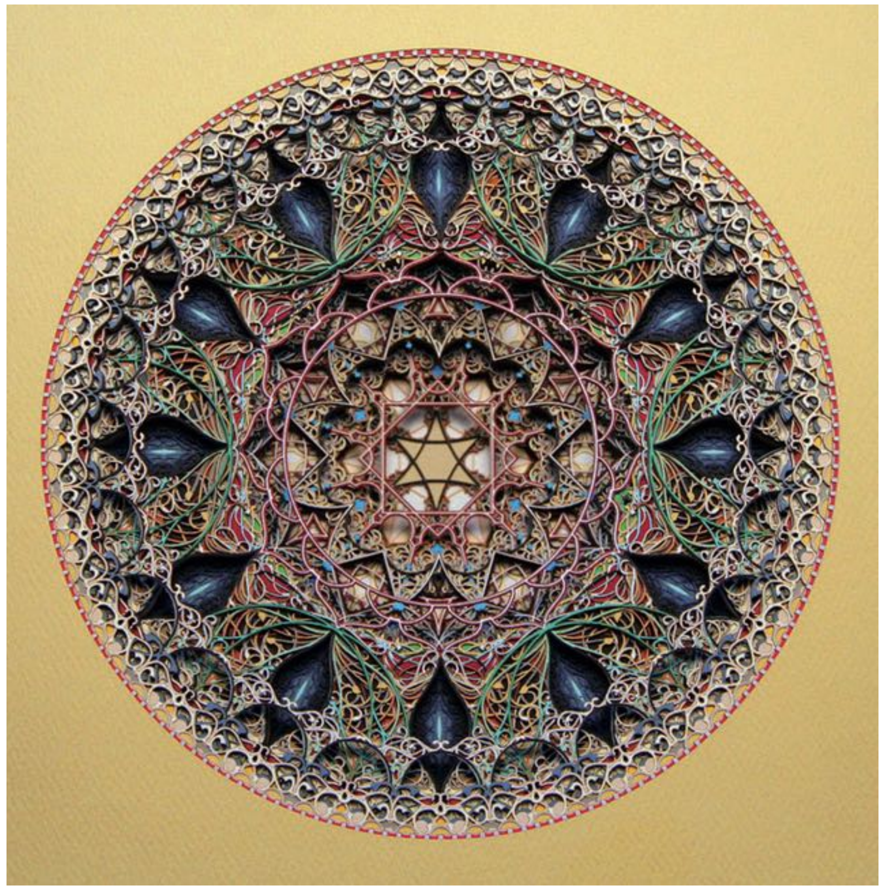

Eric Standley

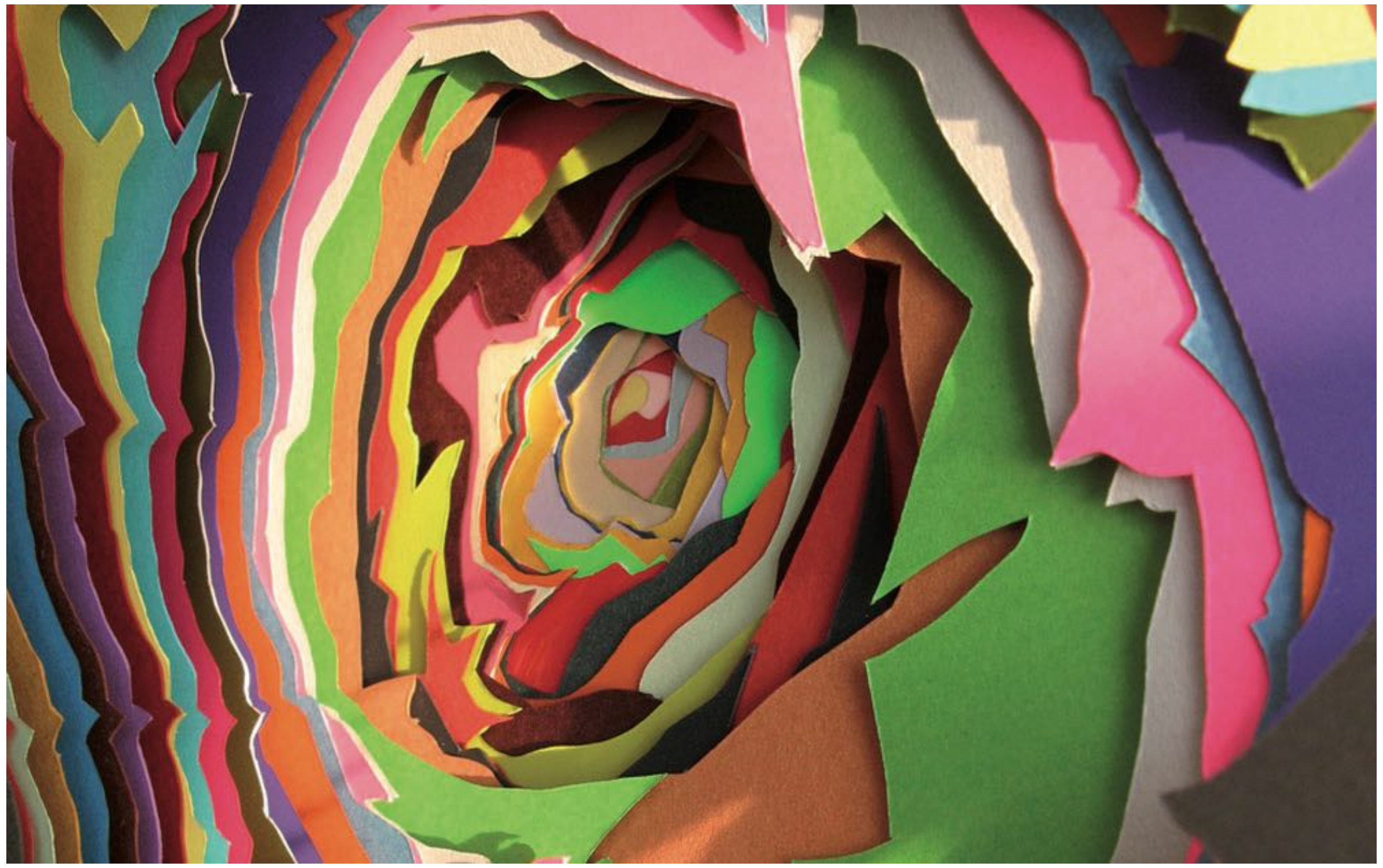

Maud Vantours

LA DERNIÈRE FOIS...

Rappel des recherches précédentes dans l'axe choisi : principe de strates en couches de papier découpé

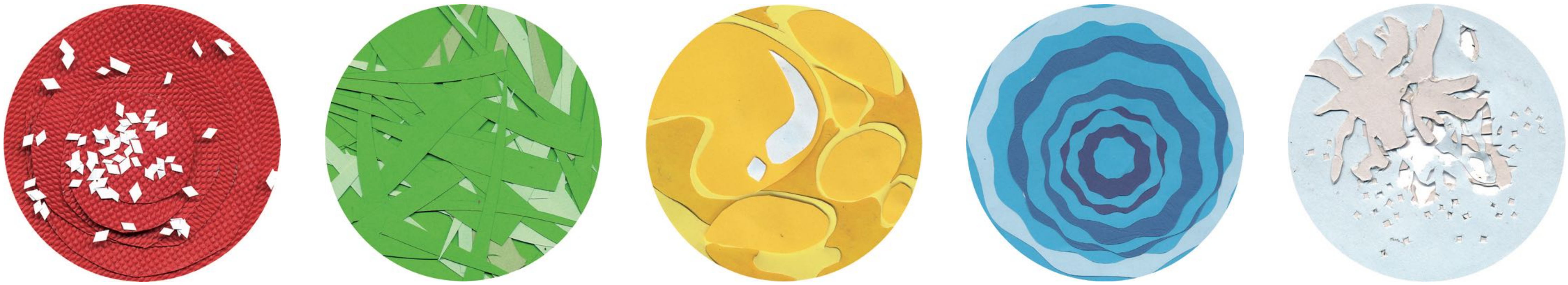

ESTELLE VILLEMIN

LIT-SPHÈRES

PROPOSITIONS FINALES :

Développement des items, choix stratégiques de motifs, de couleurs et de volume :

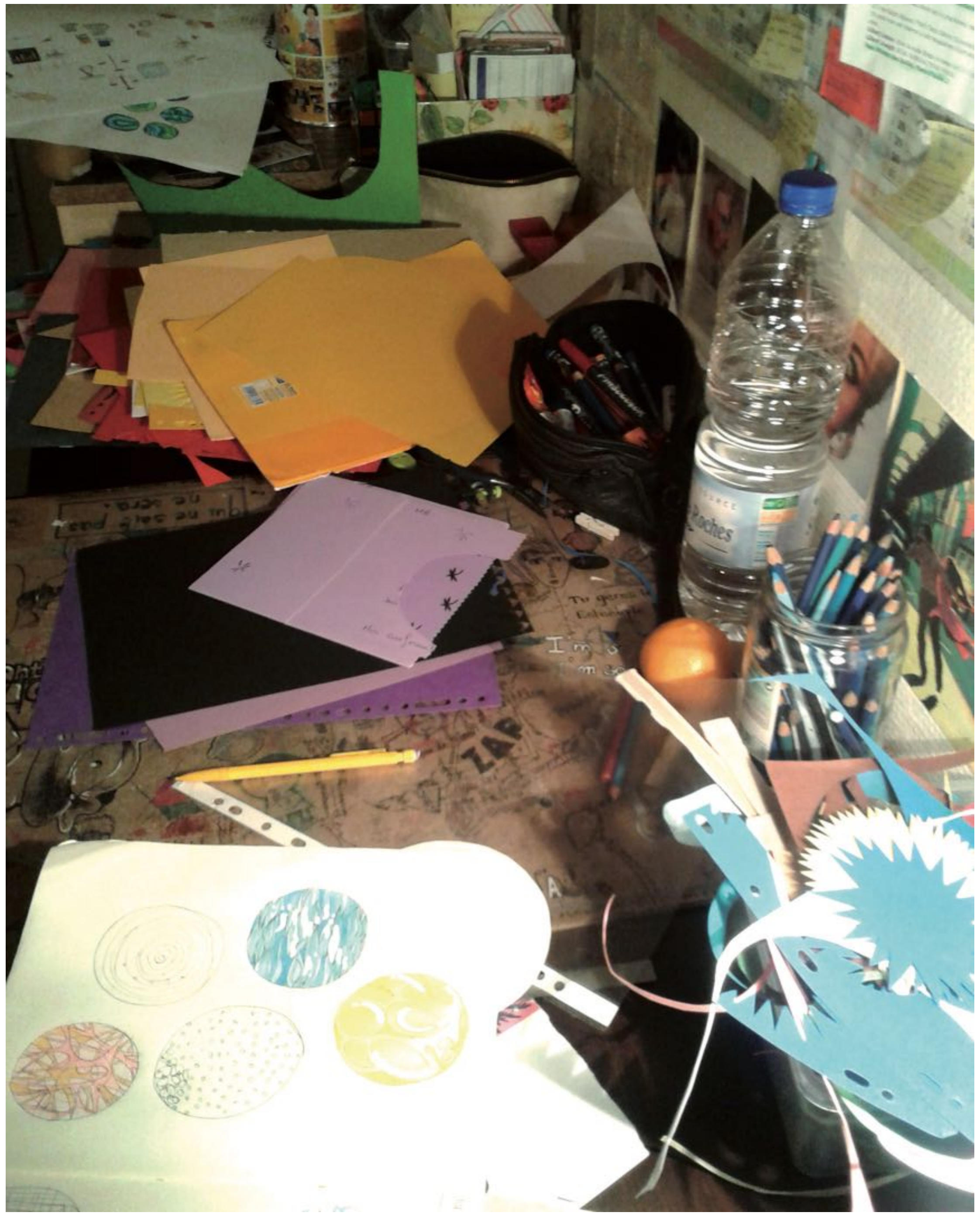

PROPOSITIONS FINALES :

Développement des items, choix stratégiques de motifs, de couleurs et de volume :

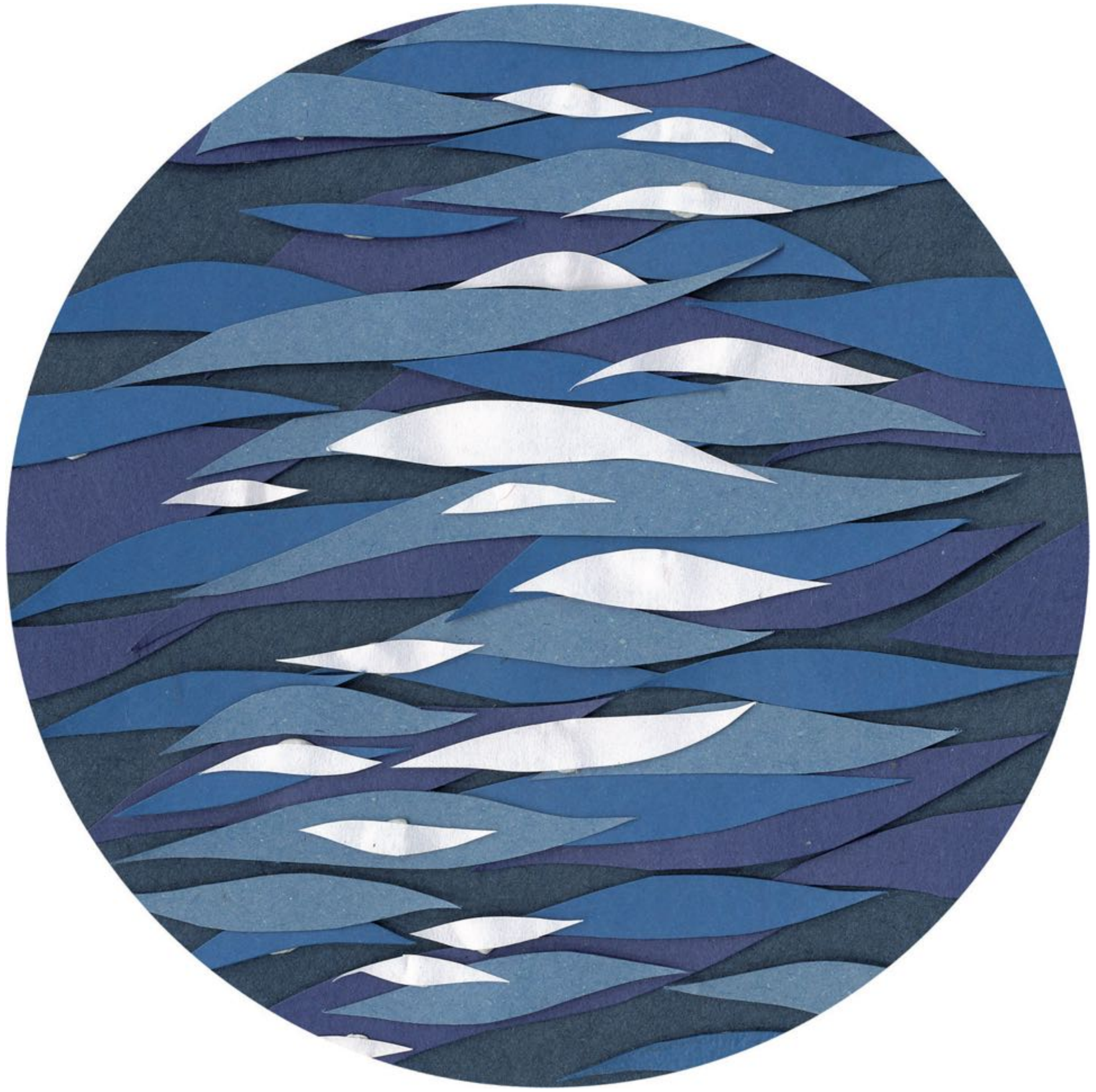

HYDROPHILE

PROPOSITIONS FINALES :

Développement des items, choix stratégiques de motifs, de couleurs et de volume :

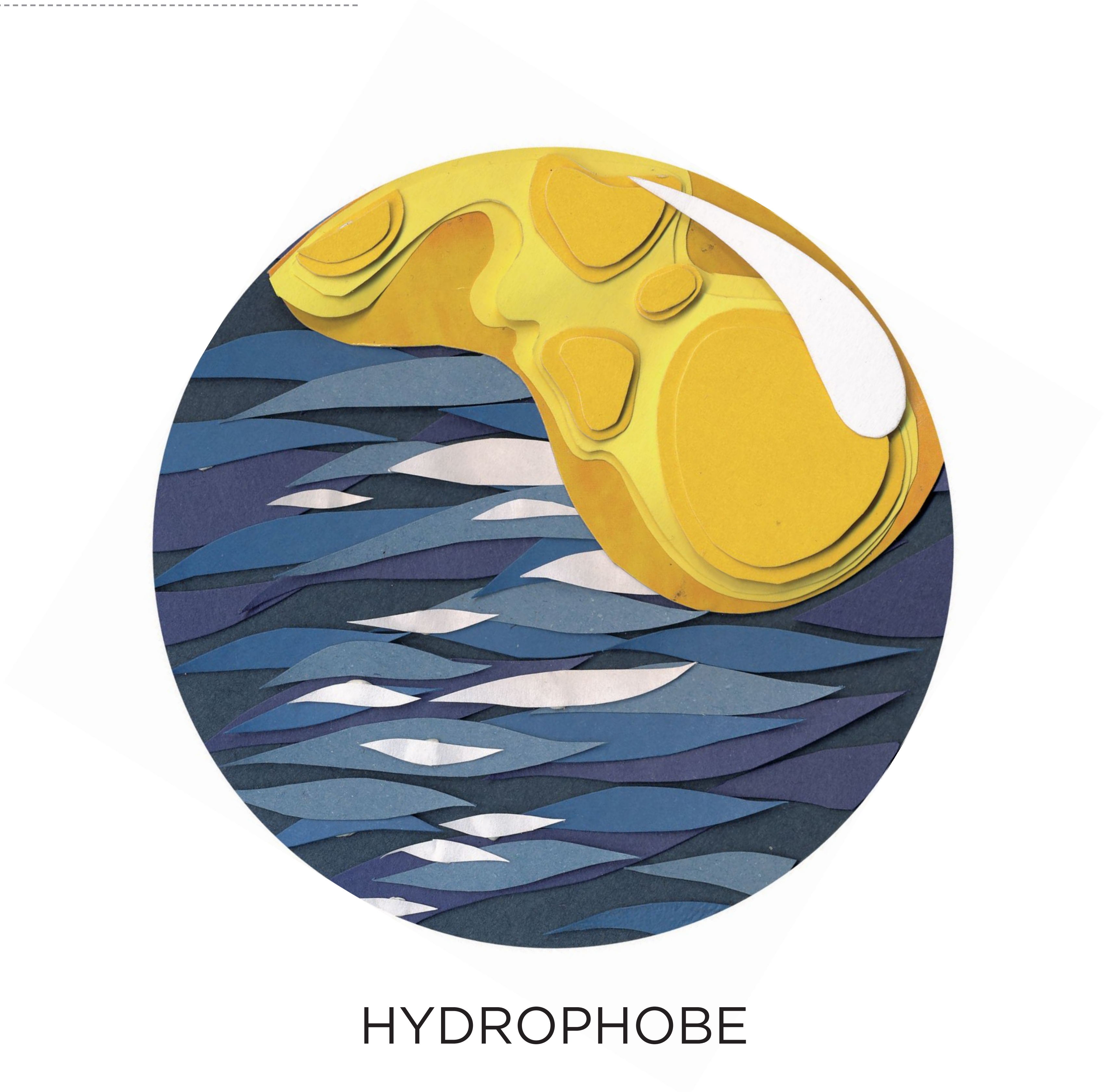

HYDROPHOBE

PROPOSITIONS FINALES :

Développement des items, choix stratégiques de motifs, de couleurs et de volume :

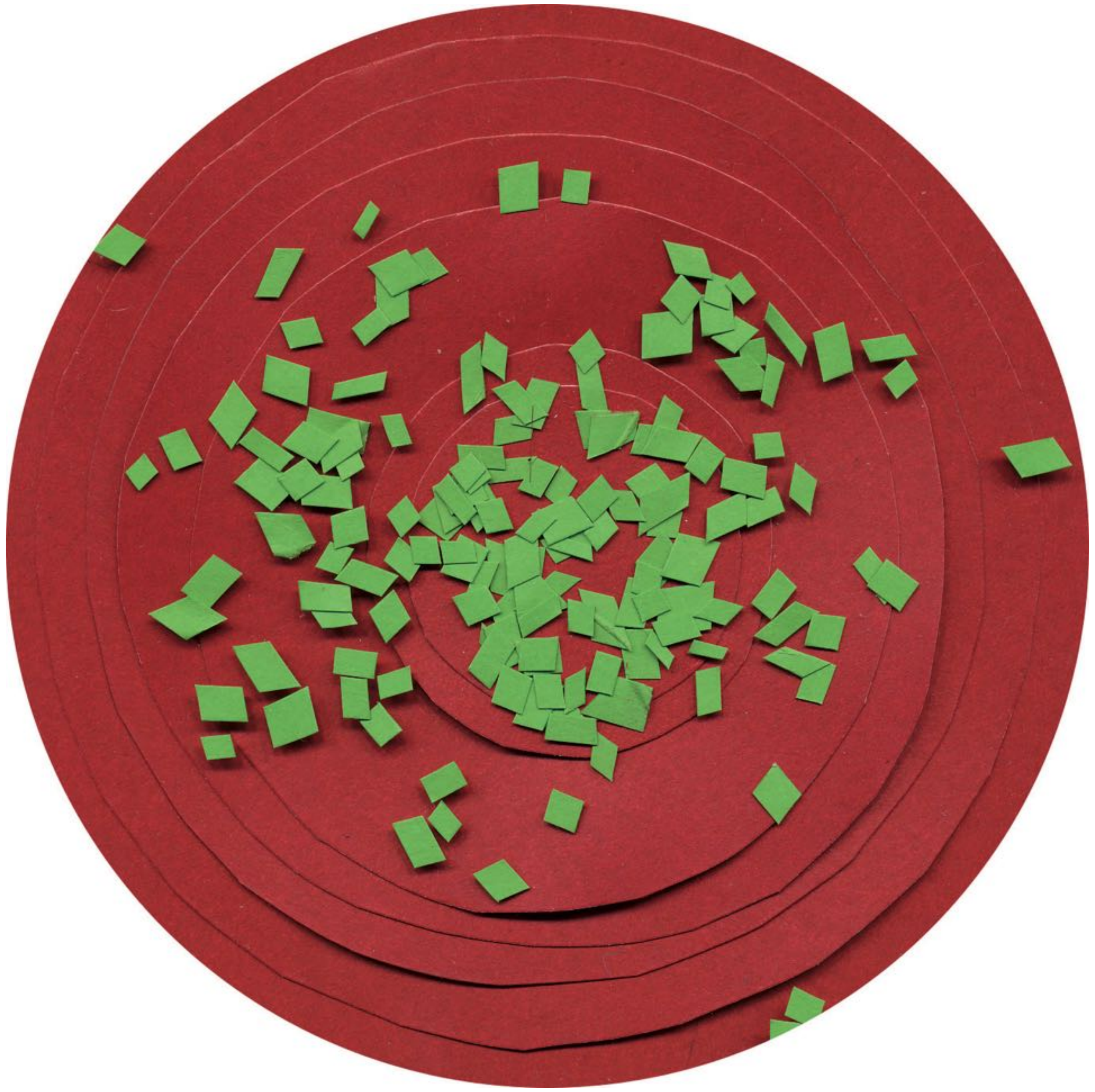

TOXIQUE

PROPOSITIONS FINALES :

Développement des items, choix stratégiques de motifs, de couleurs et de volume :

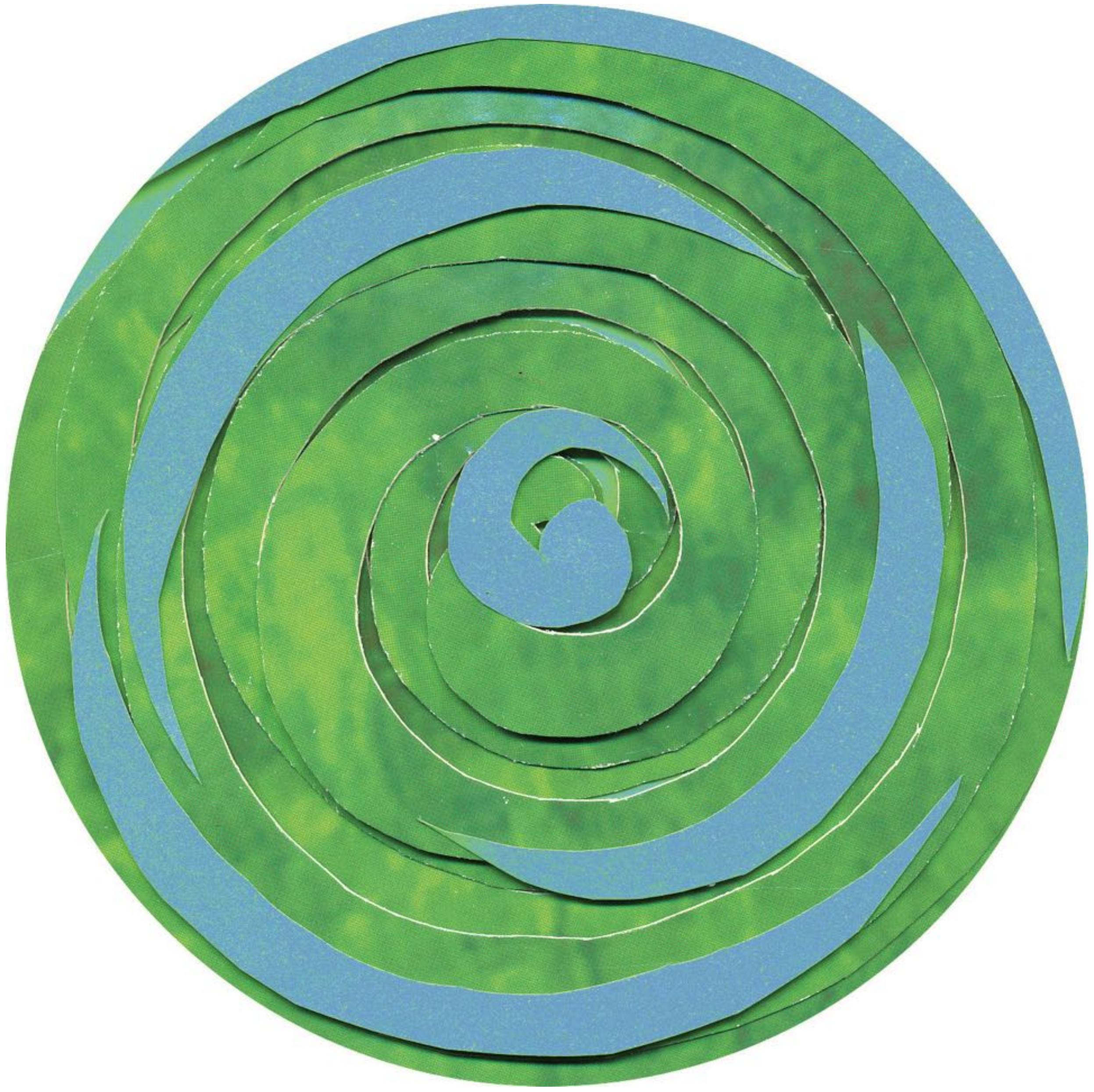

ÉCOCOMPATIBLE

PROPOSITIONS FINALES :

Développement des items, choix stratégiques de motifs, de couleurs et de volume :

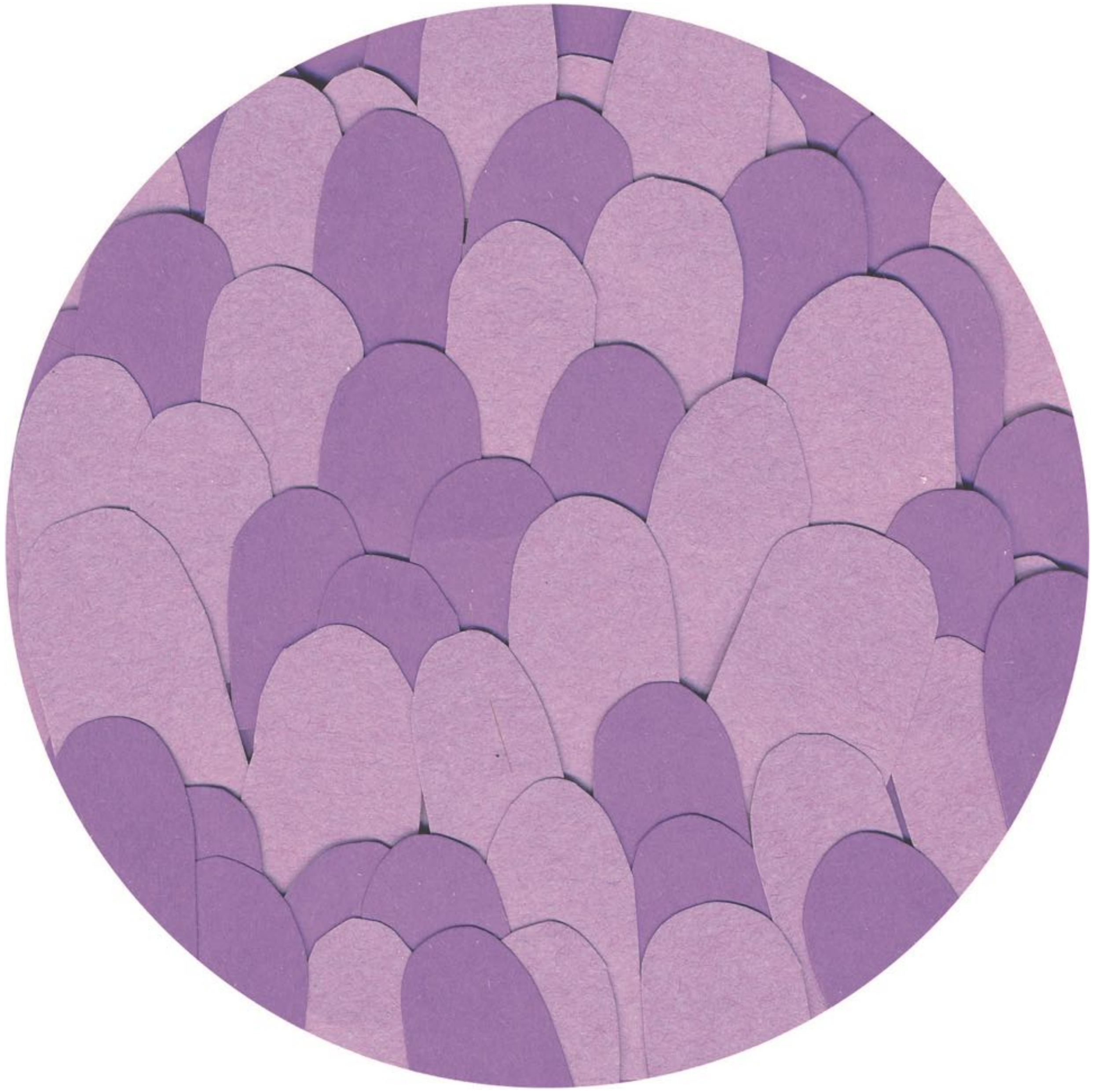

BON

ESTELLE VILLEMIN

LIT-SPHÈRES

PROPOSITIONS FINALES :

Développement des items, choix stratégiques de motifs, de couleurs et de volume :

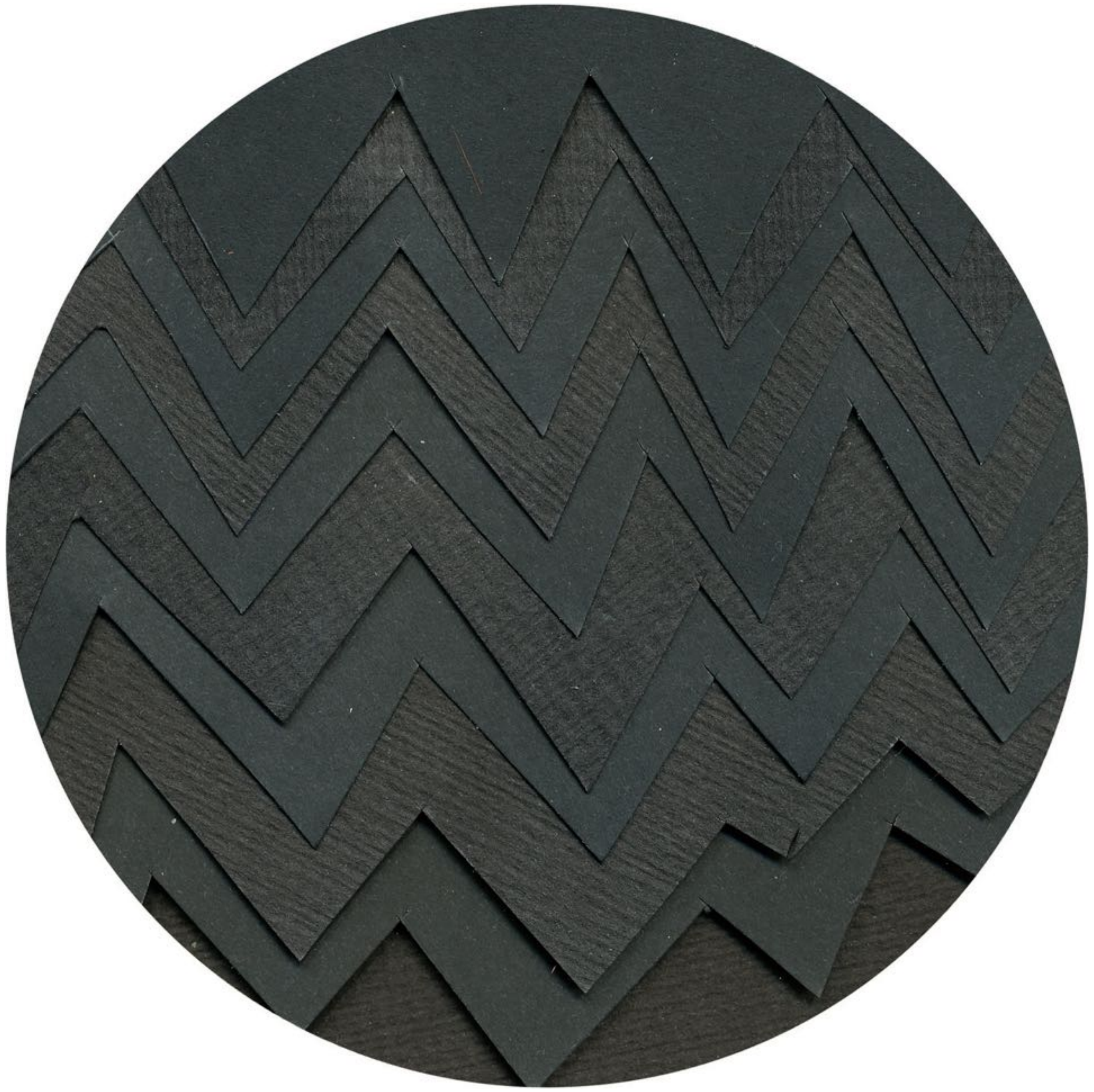

MAUVAIS

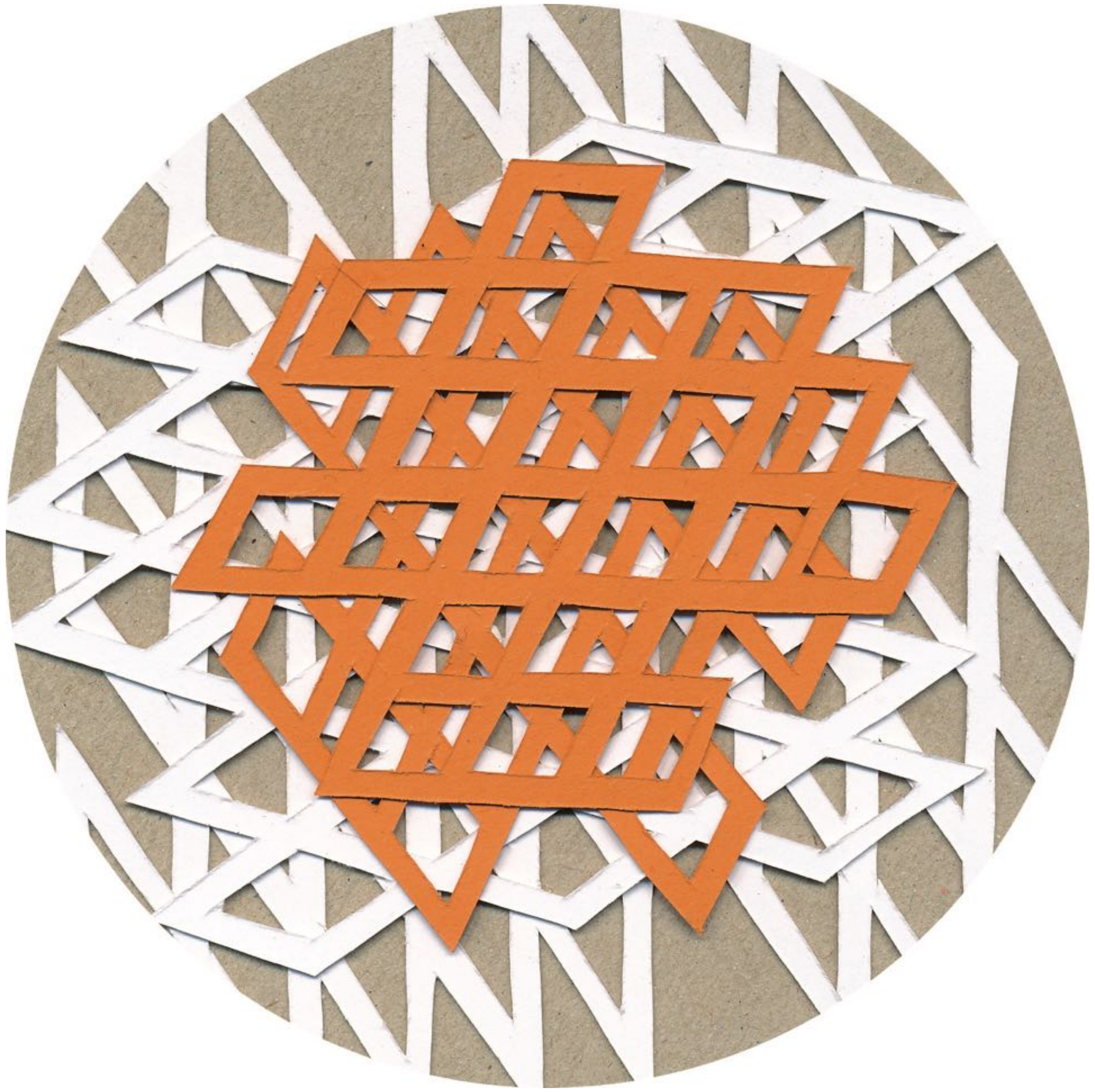

SITE ACTIF (ENZYME)

PROPOSITIONS FINALES :

Développement des items, choix stratégiques de motifs, de couleurs et de volume :

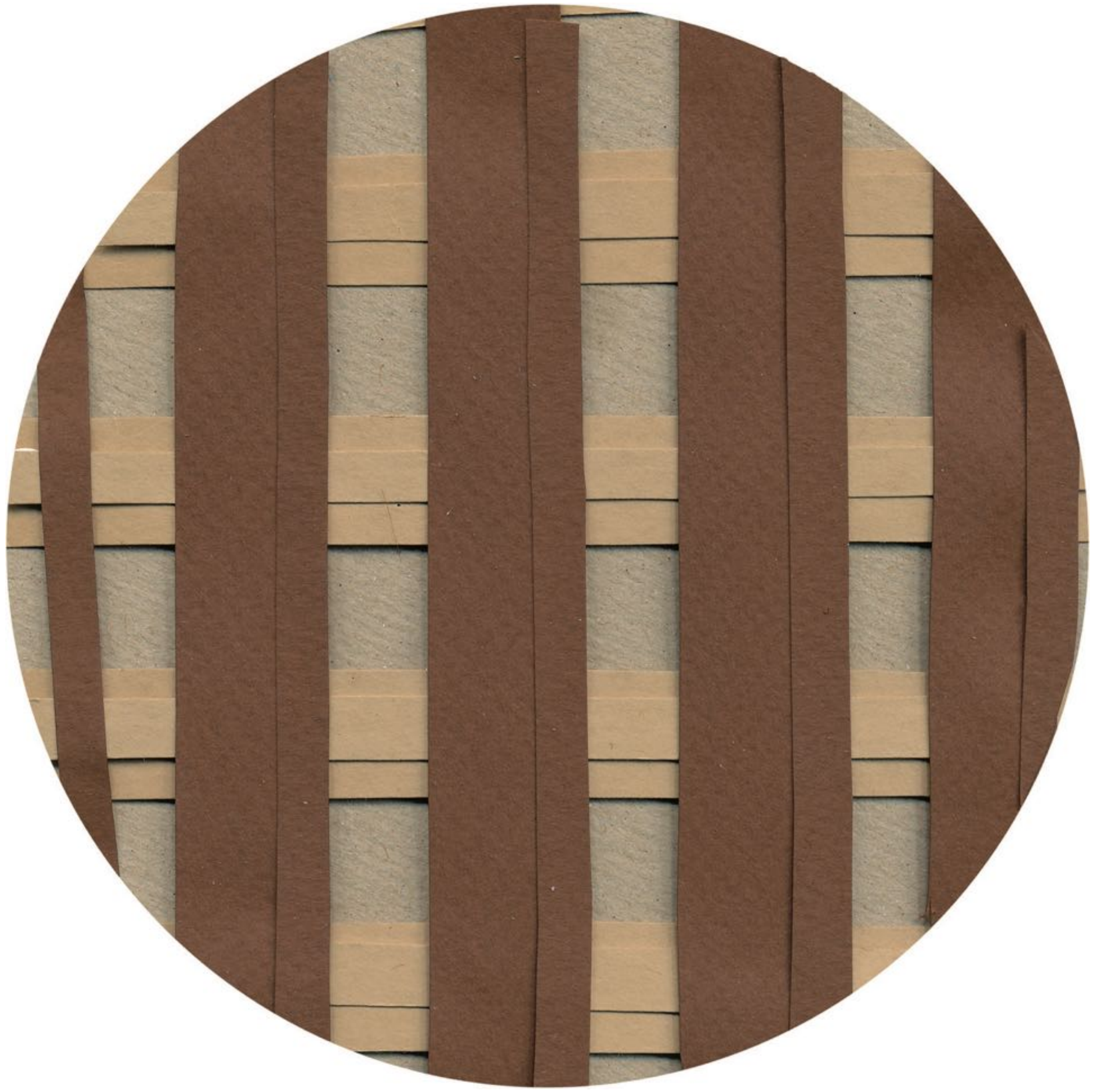

RIGIDE

PROPOSITIONS FINALES :

Développement des items, choix stratégiques de motifs, de couleurs et de volume :

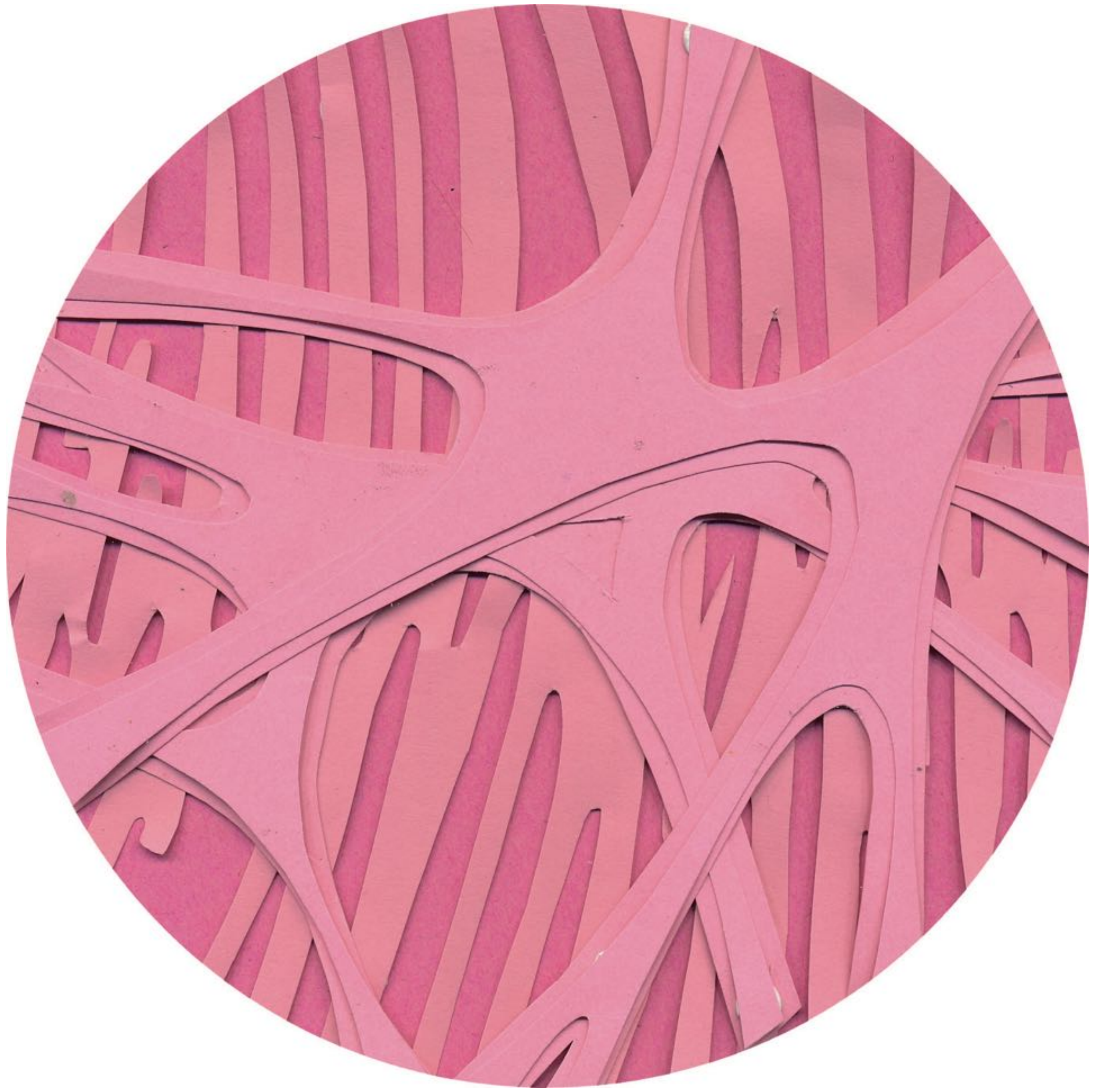

FLEXIBLE

PROPOSITIONS FINALES :

Développement des items, choix stratégiques de motifs, de couleurs et de volume :

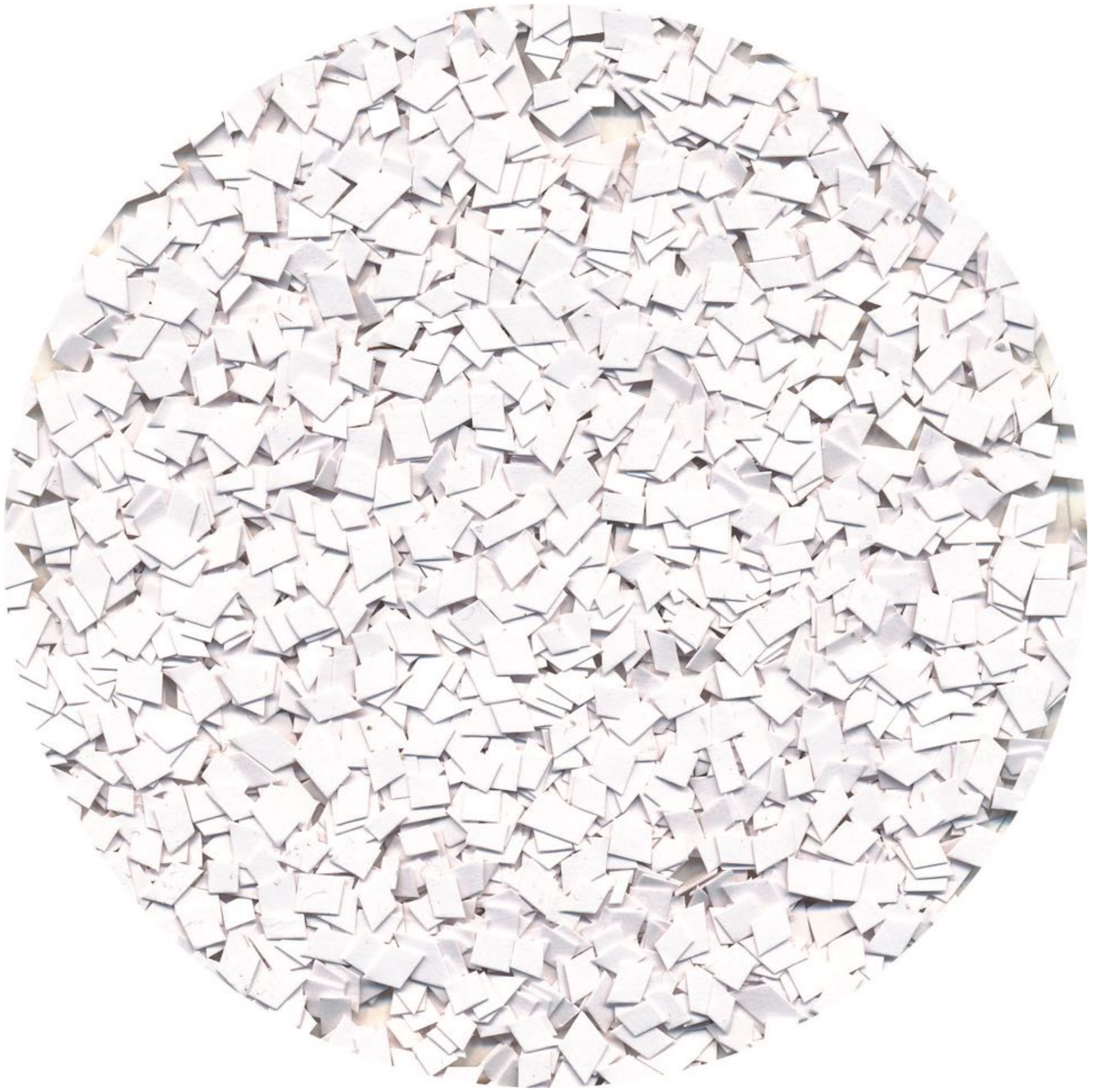

SUCRE

PROPOSITIONS FINALES :

Développement des items, choix stratégiques de motifs, de couleurs et de volume :

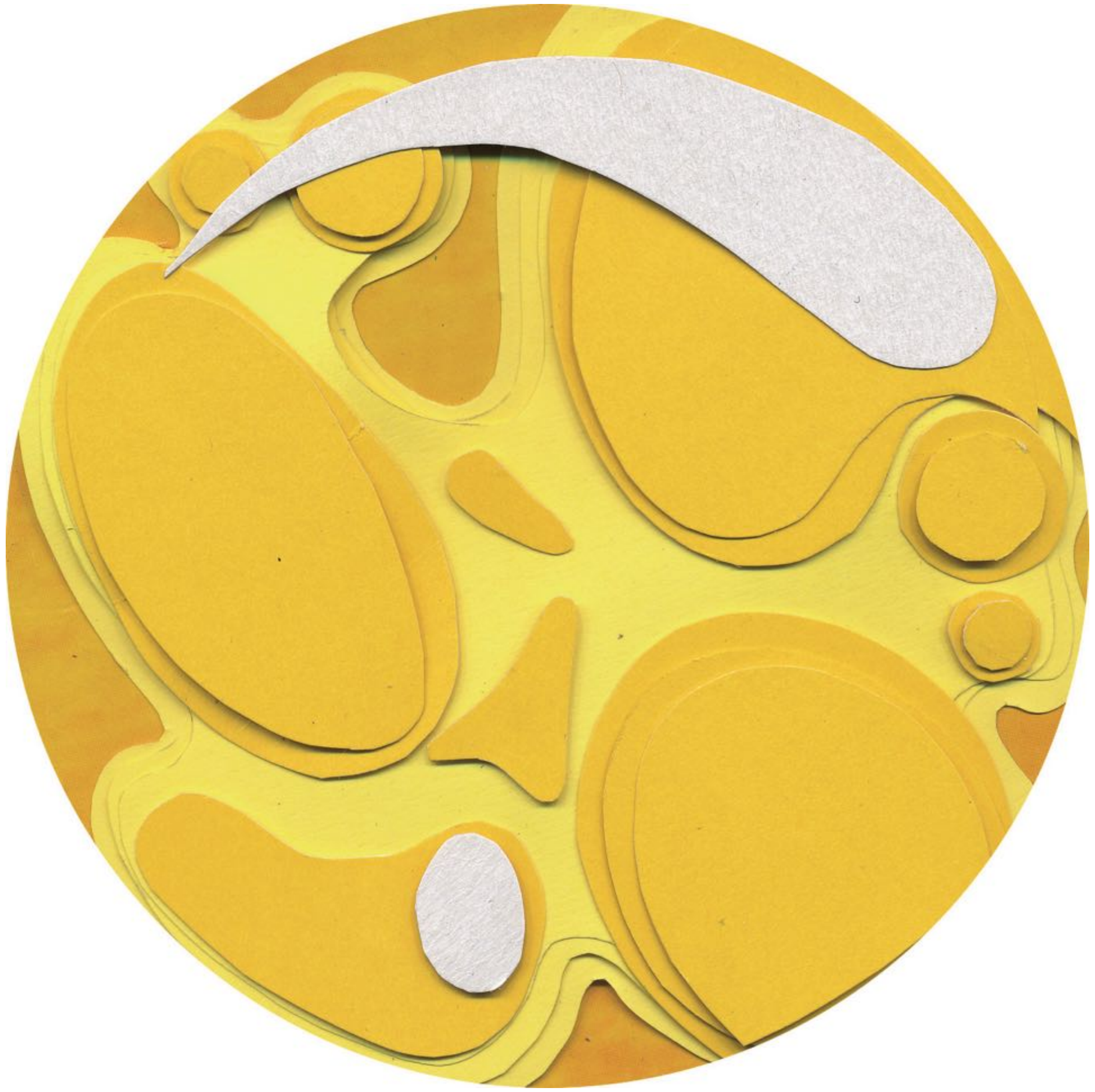

GRAISSE

PROPOSITIONS FINALES :

Développement des items, choix stratégiques de motifs, de couleurs et de volume :

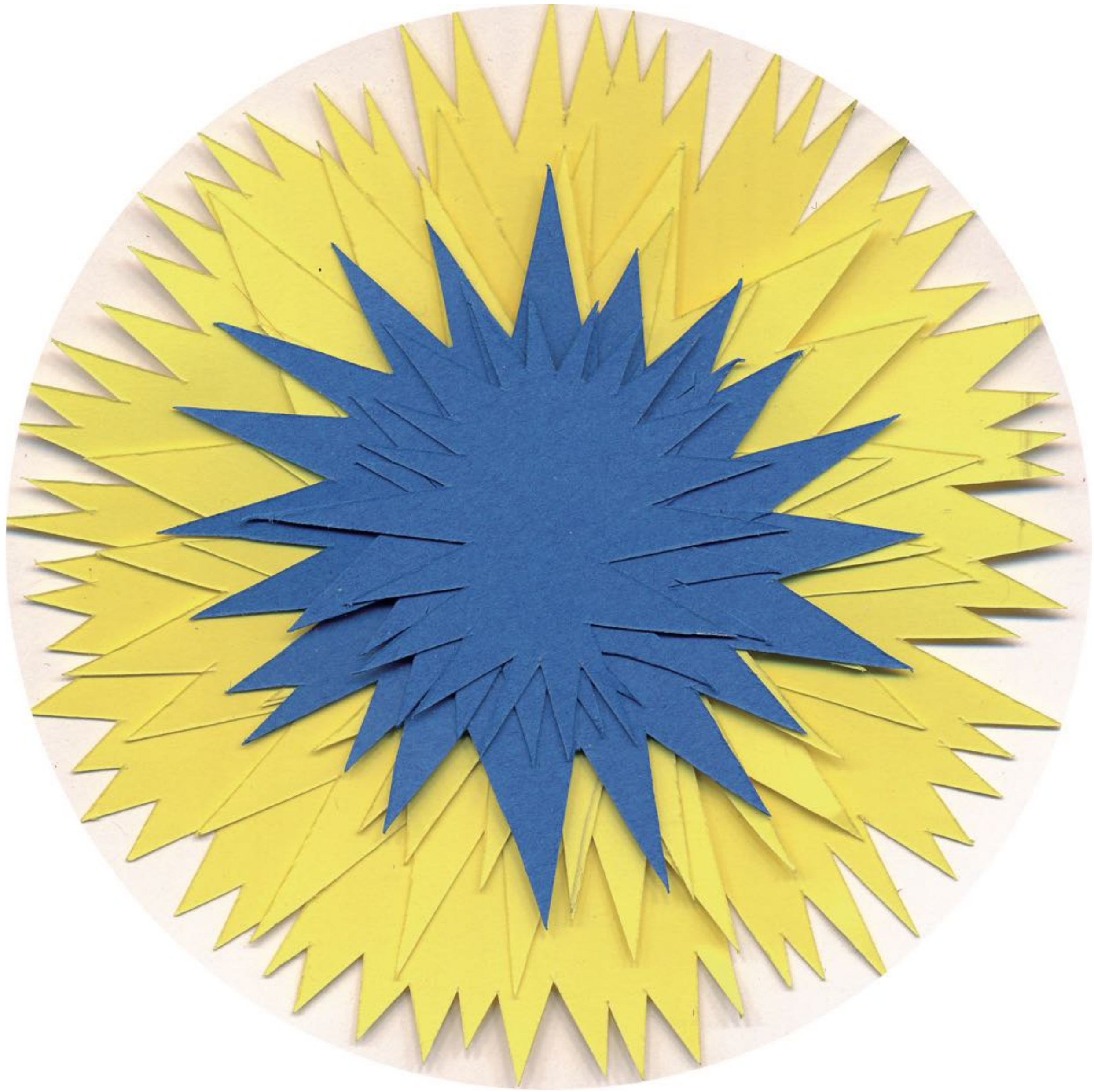

POSITIF

PROPOSITIONS FINALES :

Développement des items, choix stratégiques de motifs, de couleurs et de volume :

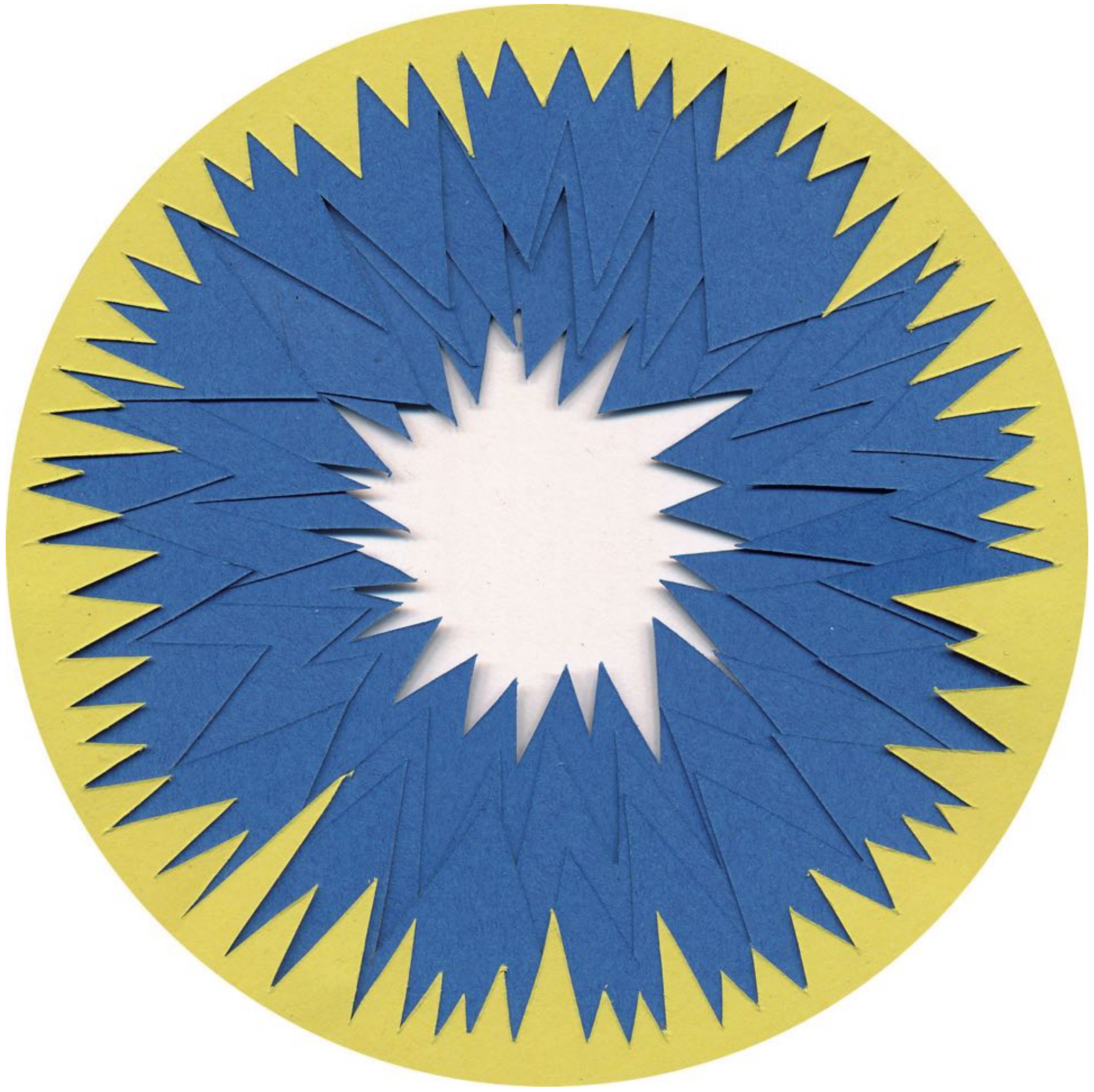

NÉGATIF

PROPOSITIONS FINALES :

Développement des items, choix stratégiques de motifs, de couleurs et de volume :

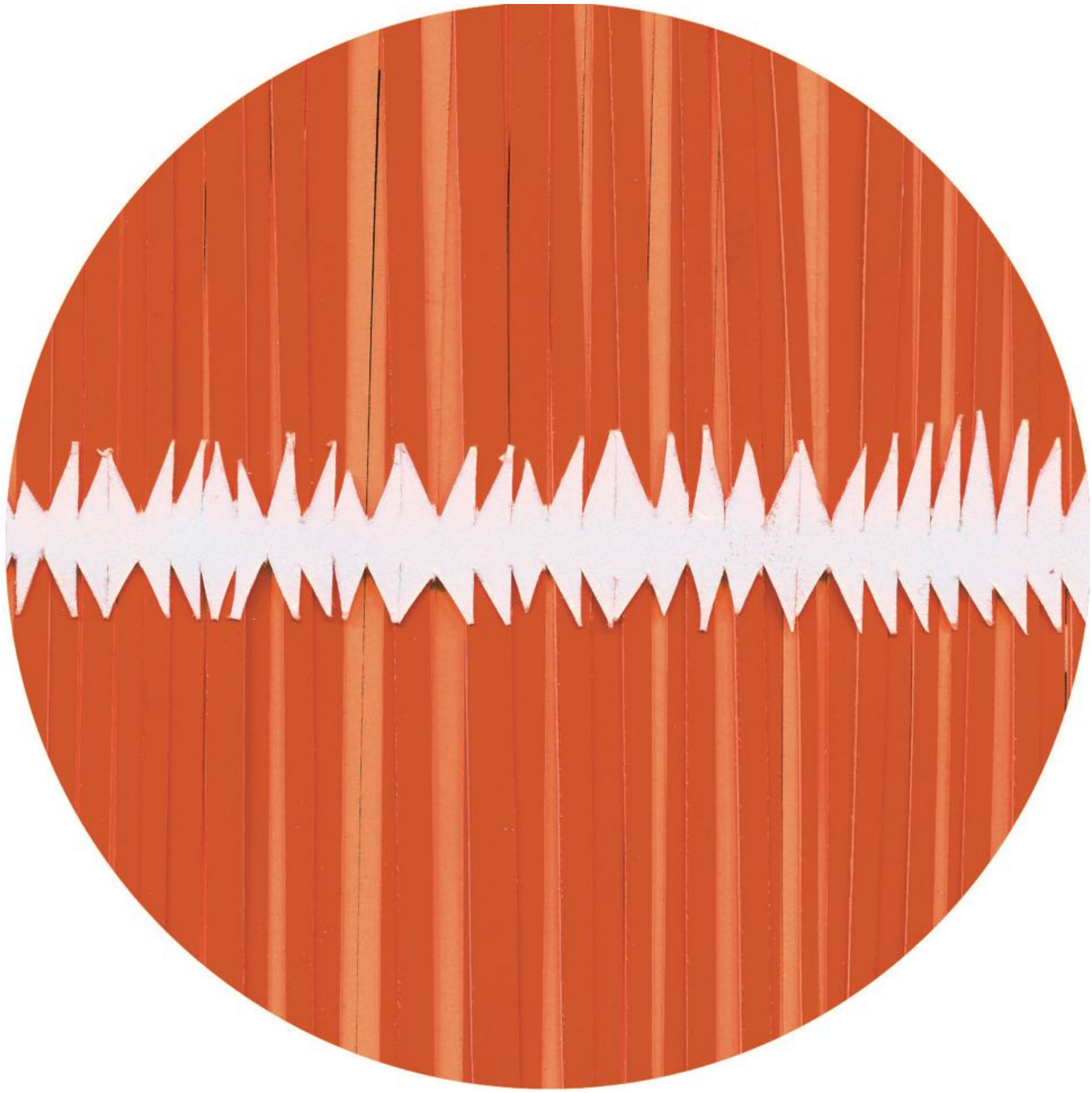

CUIVRE

PROPOSITIONS FINALES :

Développement des items, choix stratégiques de motifs, de couleurs et de volume :

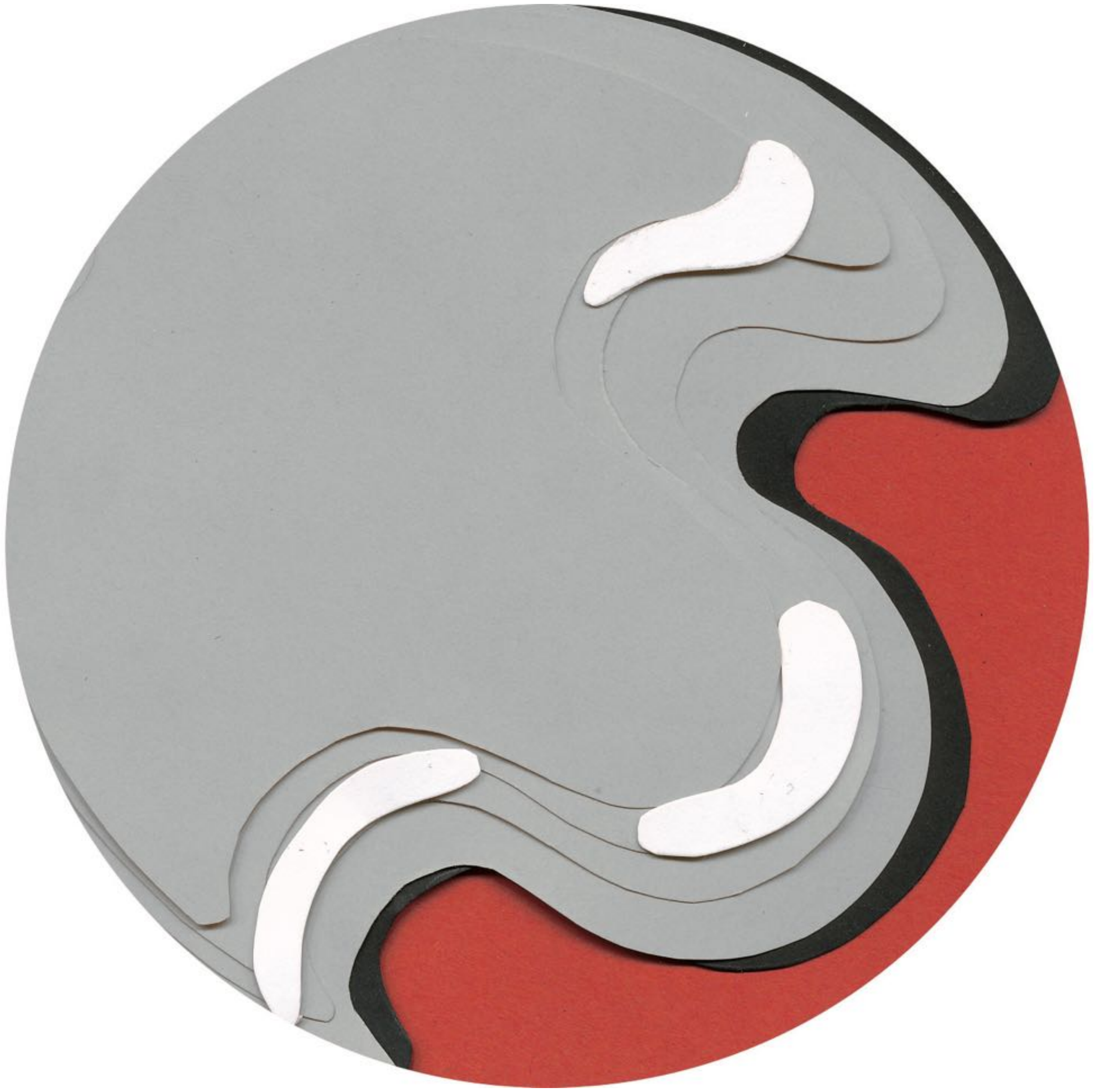

MERCURE

ESTELLE VILLEMIN

LIT-SPHÈRES

PROPOSITIONS FINALES :

Développement des items, choix stratégiques de motifs, de couleurs et de volume :

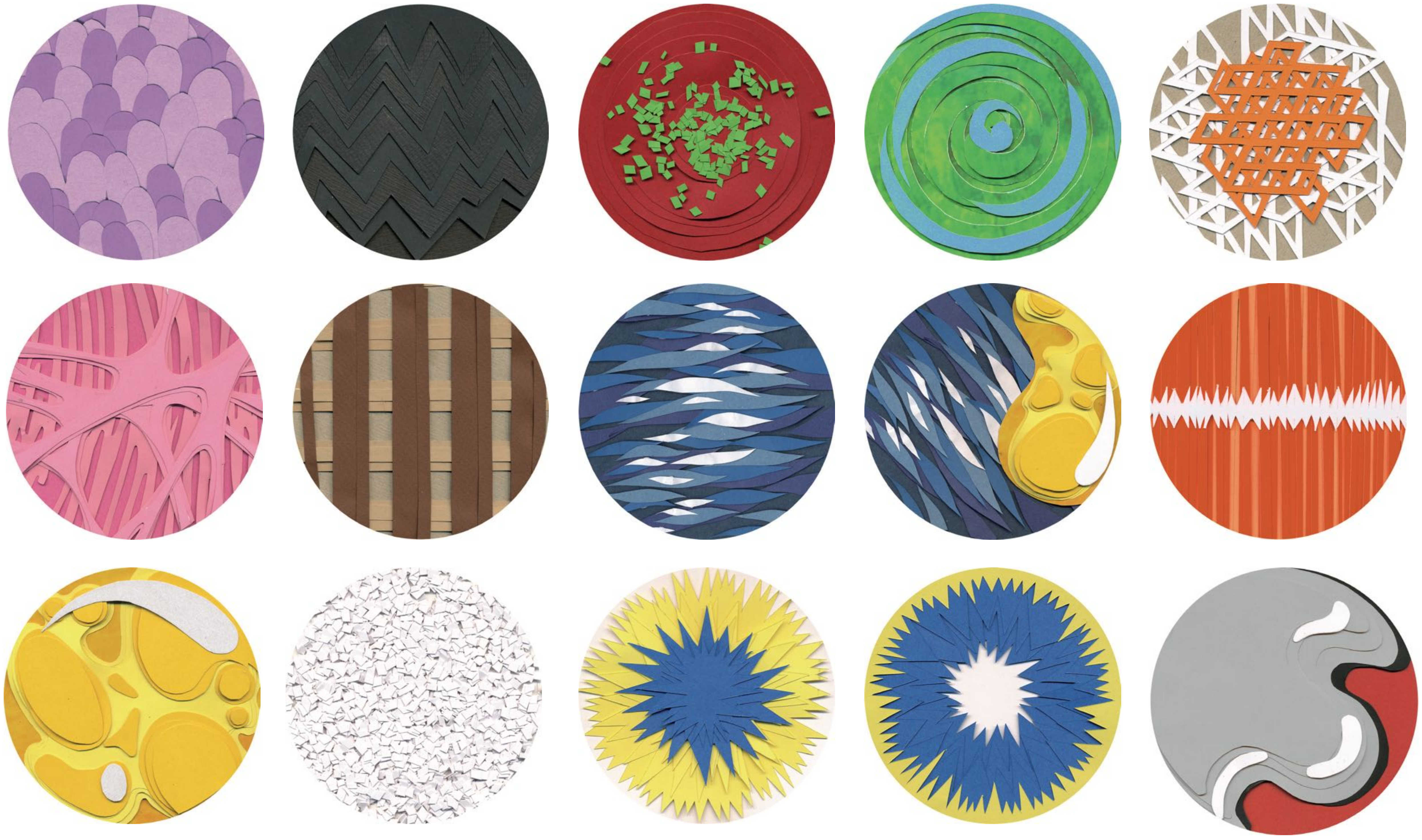

INTÉGRATION AU LOGICIEL

PROPOSITIONS FINALES :

Intégration au logiciel unitymol, (molécule de caféine)

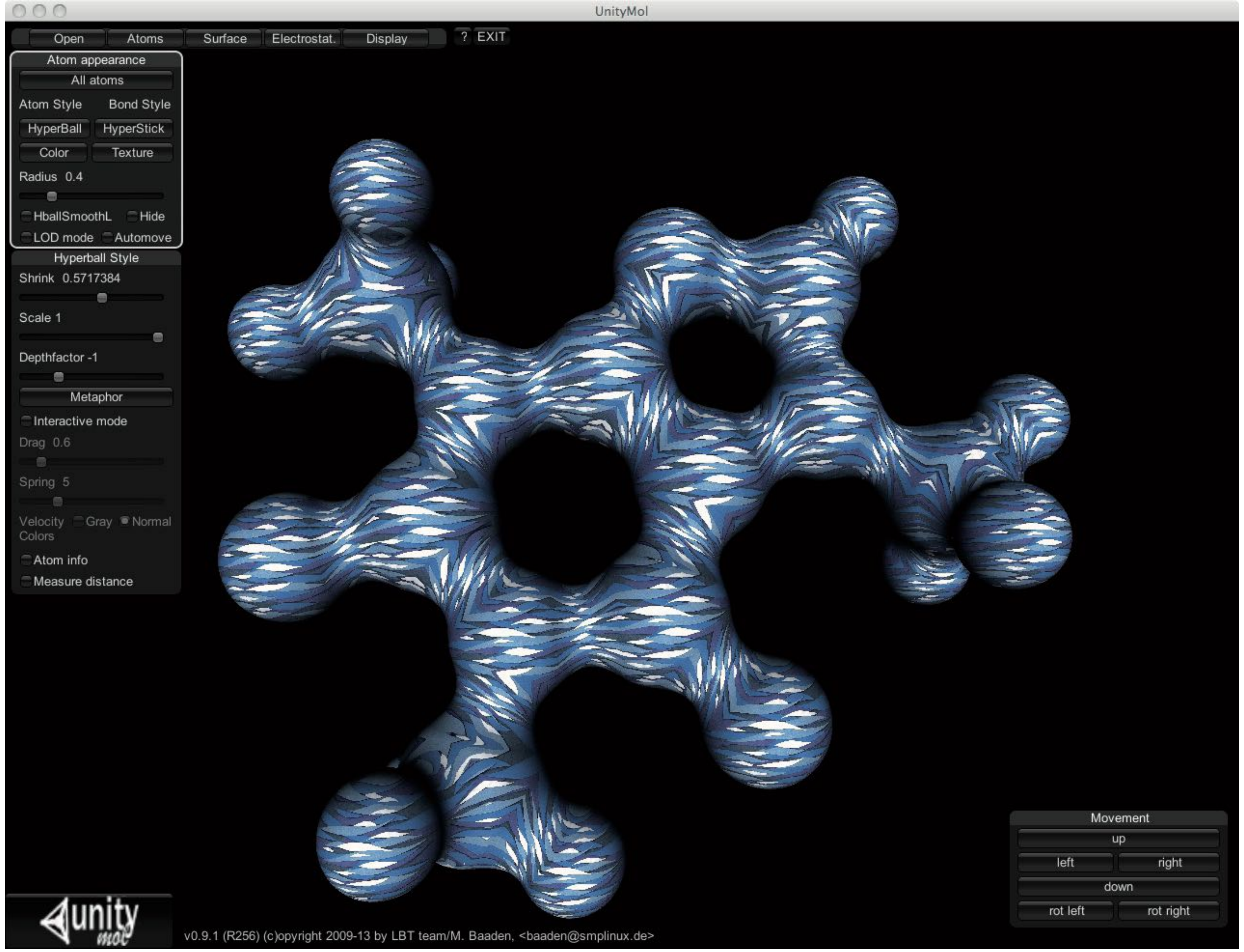

HYDROPHILE

PROPOSITIONS FINALES :

Intégration au logiciel unitymol,
(molécule de caféine)

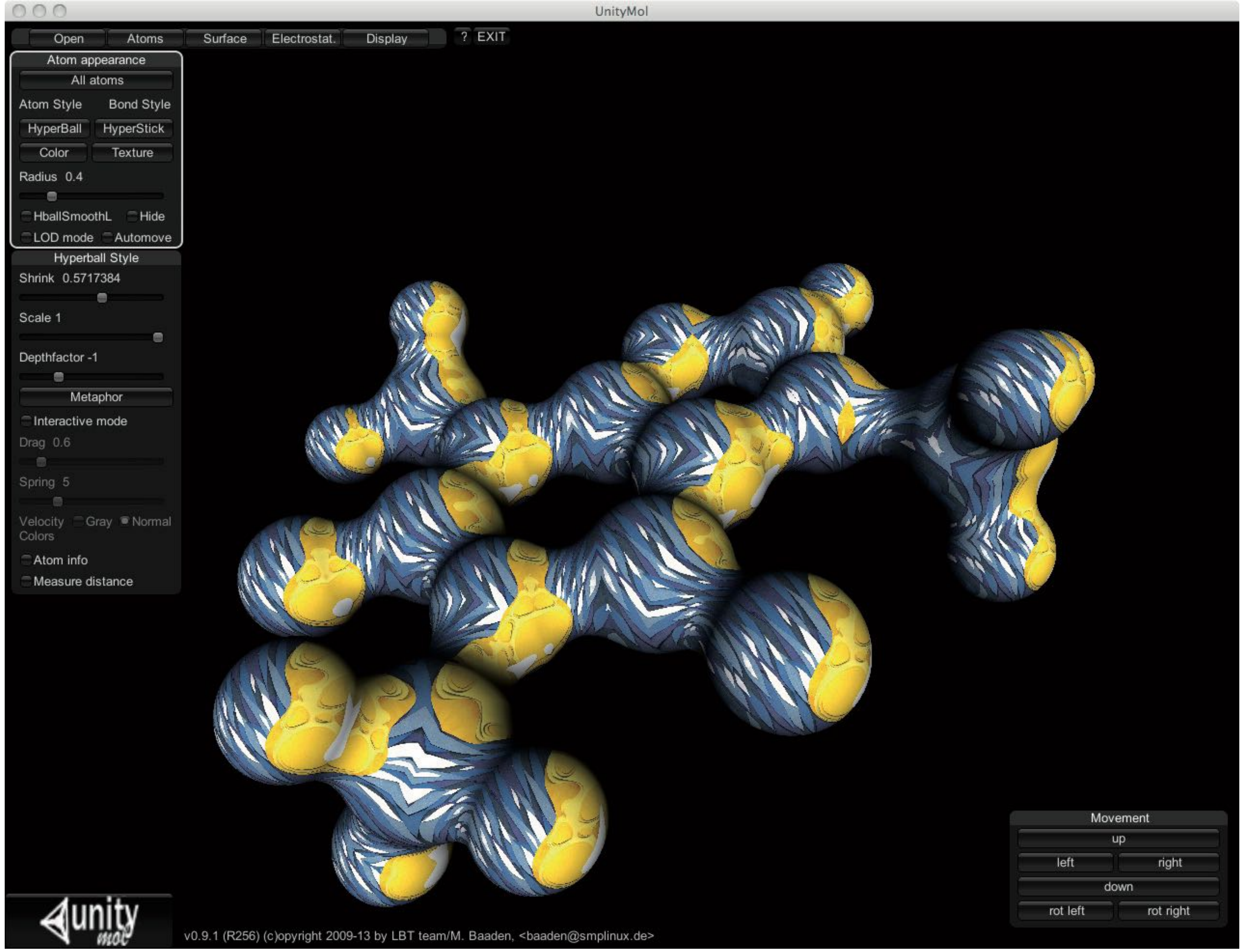

HYDROPHOBE

ESTELLE VILLEMIN

LIT-SPHÈRES

PROPOSITIONS FINALES :

Intégration au logiciel unitymol,
(molécule de caféine)

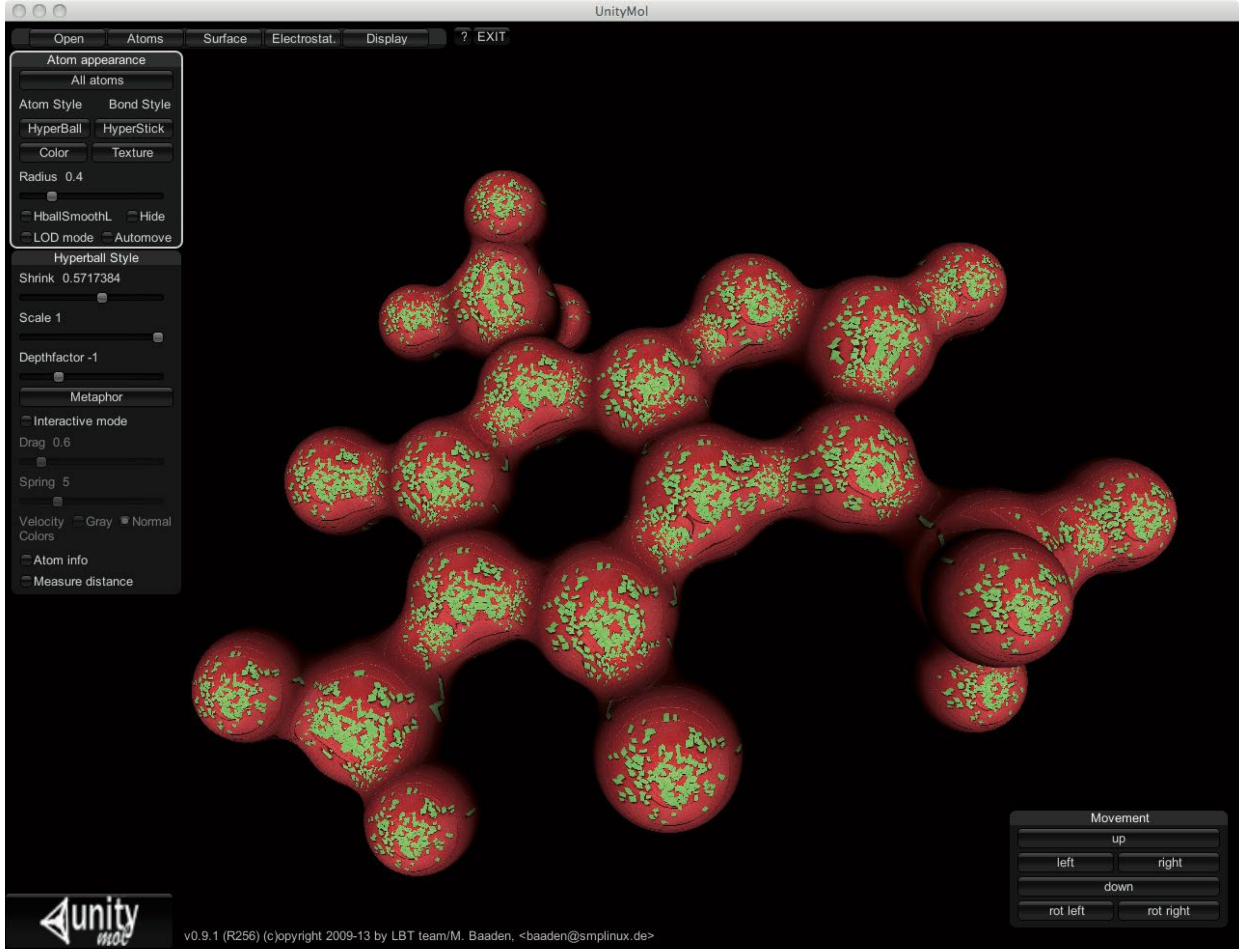

TOXIQUE

PROPOSITIONS FINALES :

Intégration au logiciel unitymol,
(molécule de caféine)

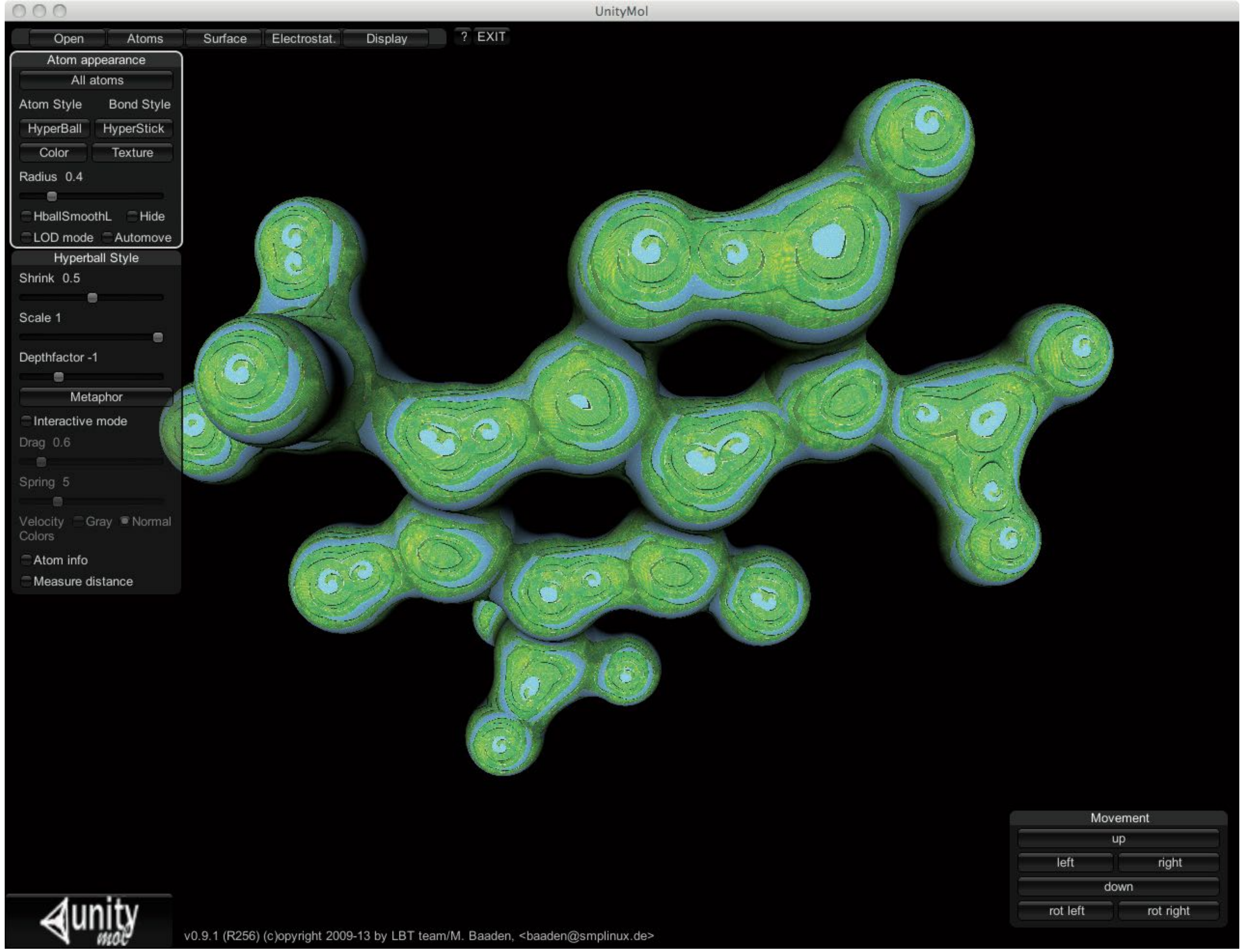

ÉCOCOMPATIBLE

PROPOSITIONS FINALES :

Intégration au logiciel unitymol, (molécule de caféine)

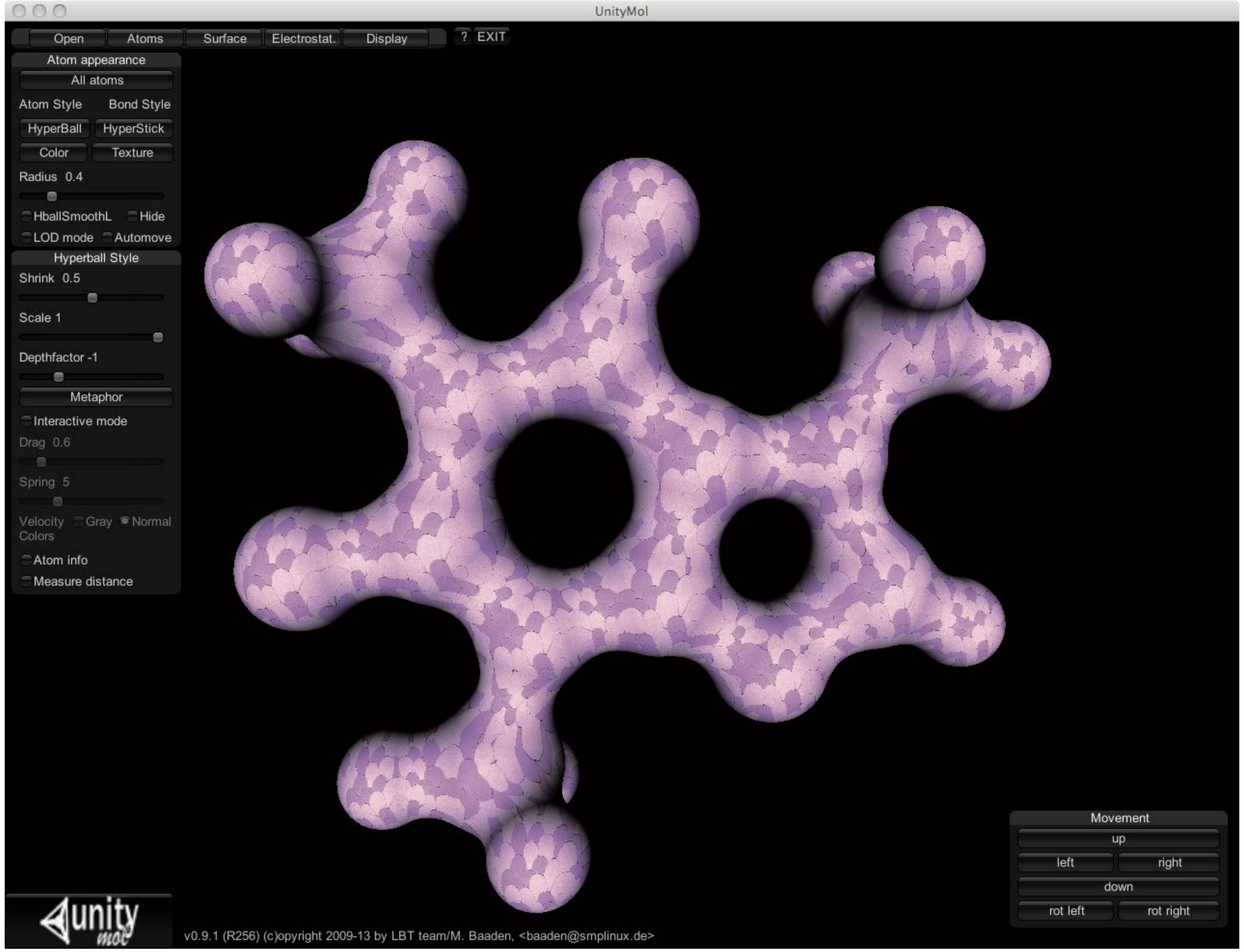

BON

ESTELLE VILLEMIN

LIT-SPHÈRES

PROPOSITIONS FINALES :

Intégration au logiciel unitymol,
(molécule de caféine)

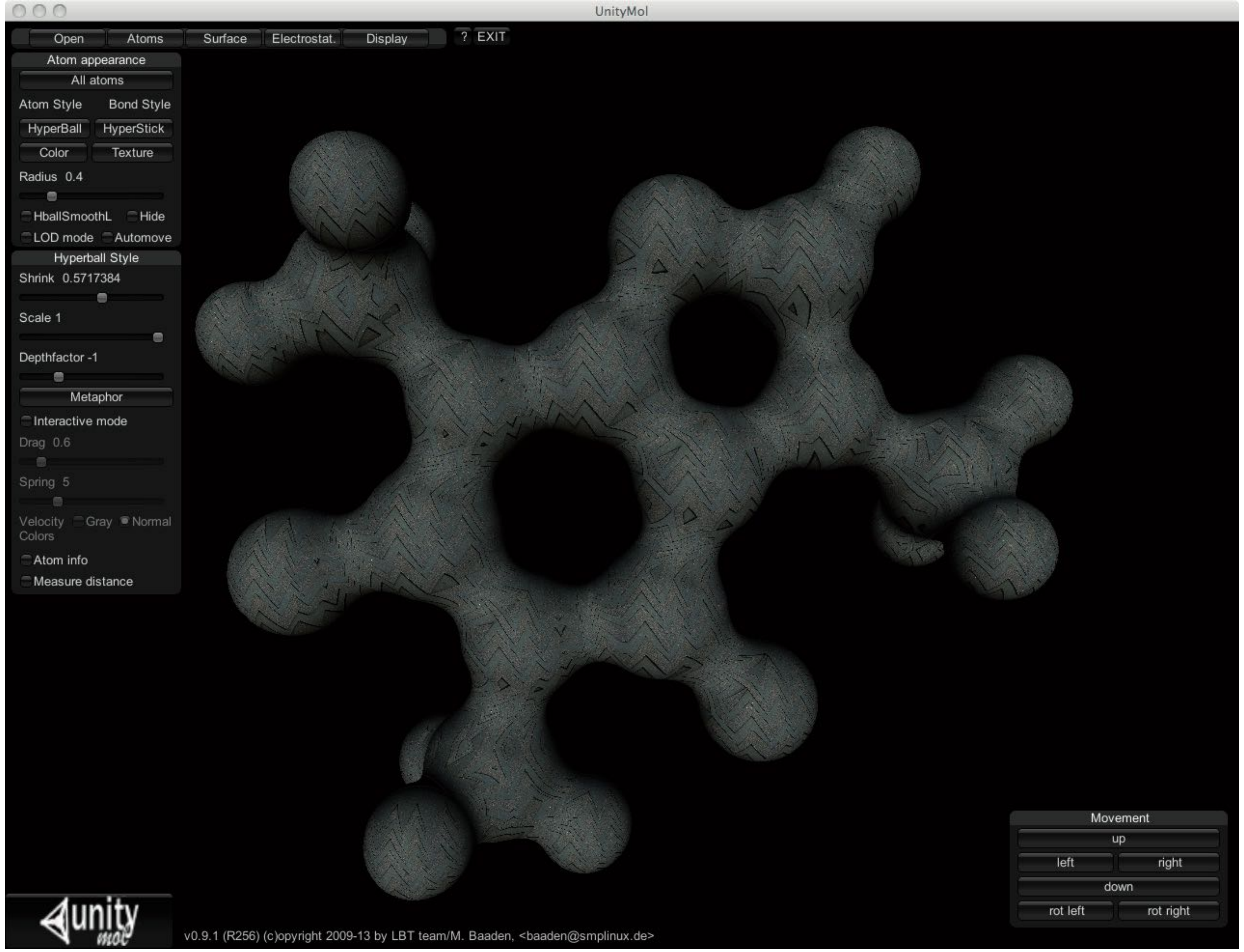

MAUVAIS

ESTELLE VILLEMIN

LIT-SPHÈRES

PROPOSITIONS FINALES :

Intégration au logiciel unitymol,
(molécule de caféine)

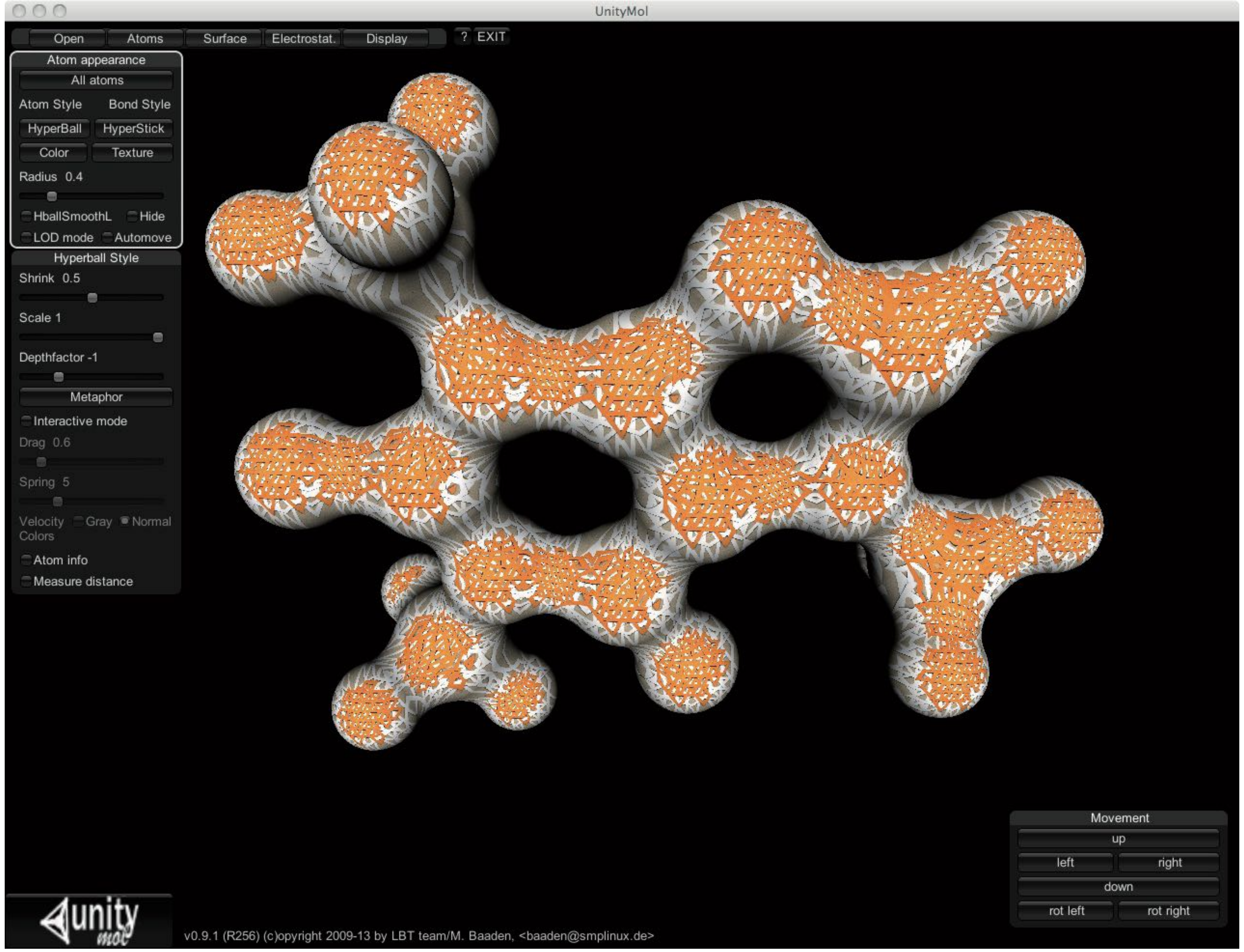

SITE ACTIF (ENZYME)

ESTELLE VILLEMIN

LIT-SPHÈRES

PROPOSITIONS FINALES :

Intégration au logiciel unitymol,
(molécule de caféine)

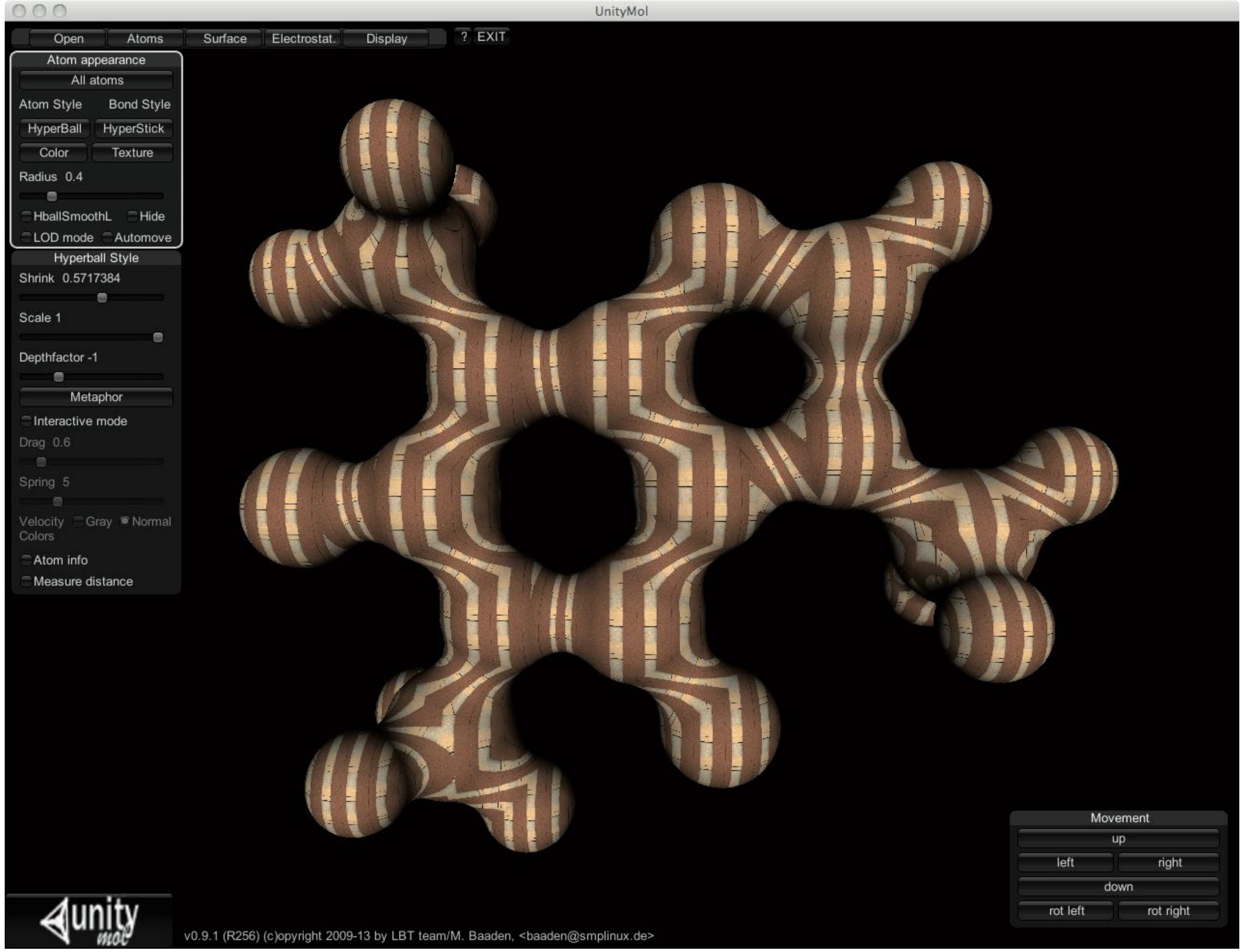

RIGIDE

ESTELLE VILLEMIN

LIT-SPHÈRES

PROPOSITIONS FINALES :

**Intégration au logiciel unitymol,
(molécule de caféine)**

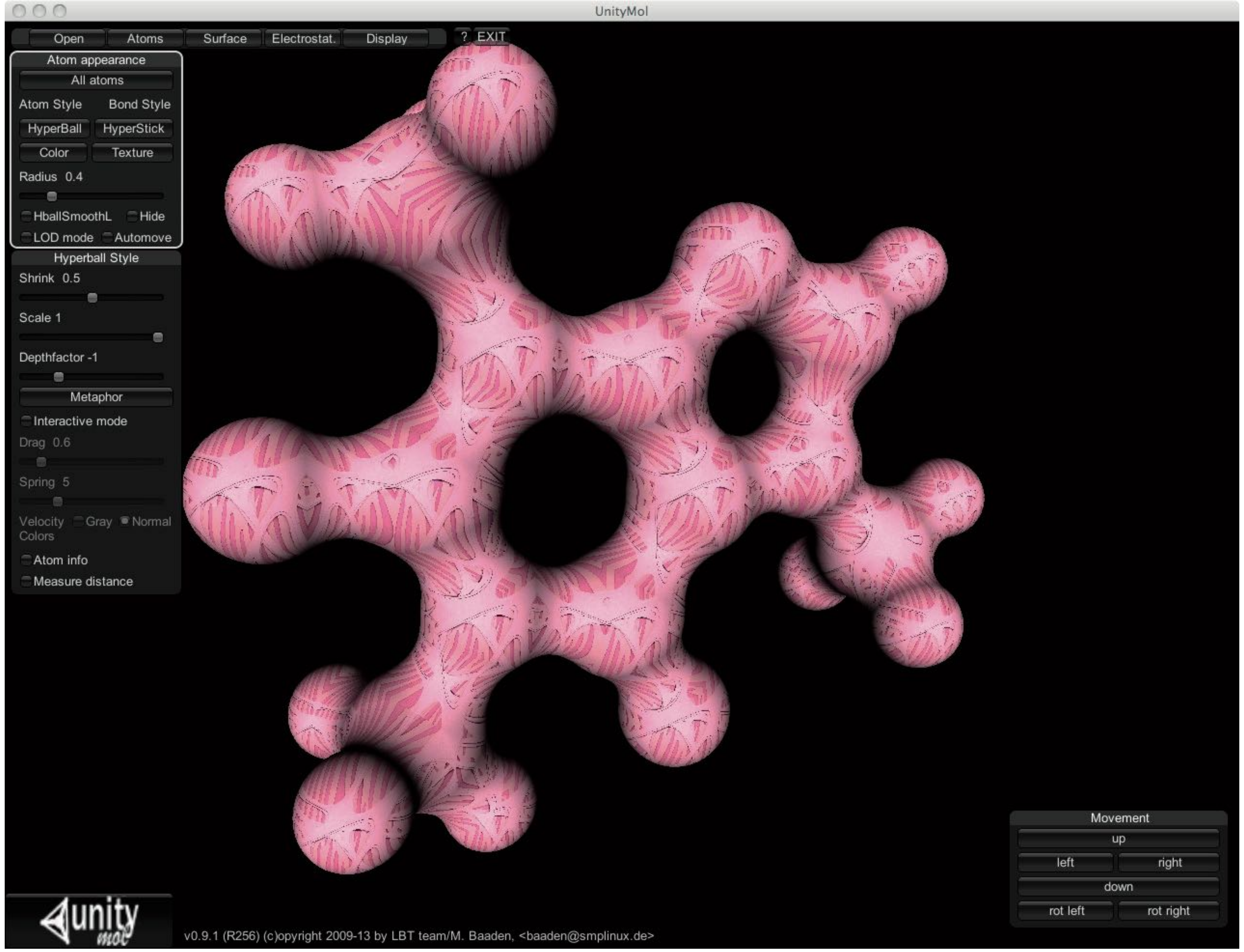

FLEXIBLE

ESTELLE VILLEMIN

LIT-SPHÈRES

PROPOSITIONS FINALES :

**Intégration au logiciel unitymol,
(molécule de caféine)**

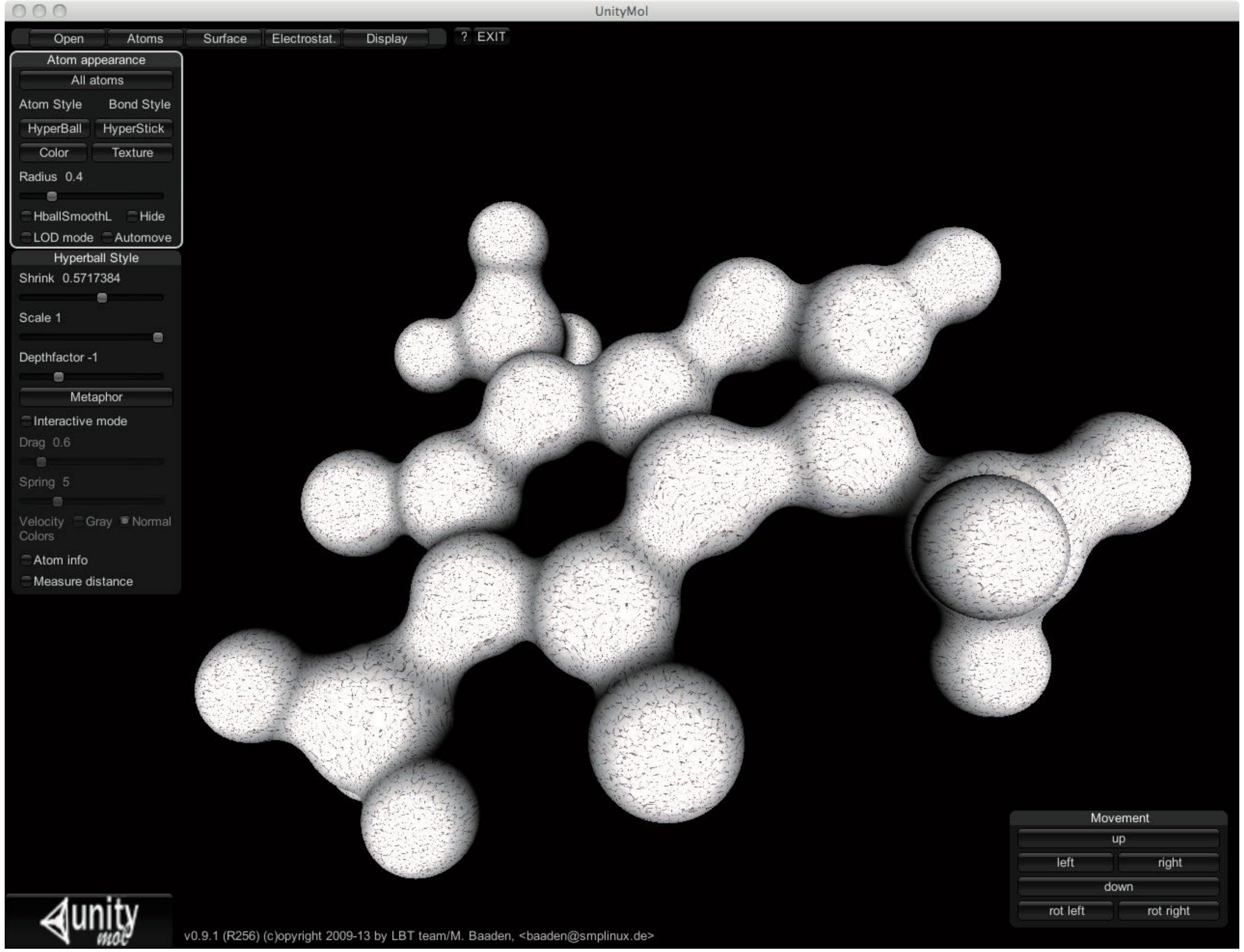

SUCRE

ESTELLE VILLEMIN

LIT-SPHÈRES

PROPOSITIONS FINALES :

**Intégration au logiciel unitymol,
(molécule de caféine)**

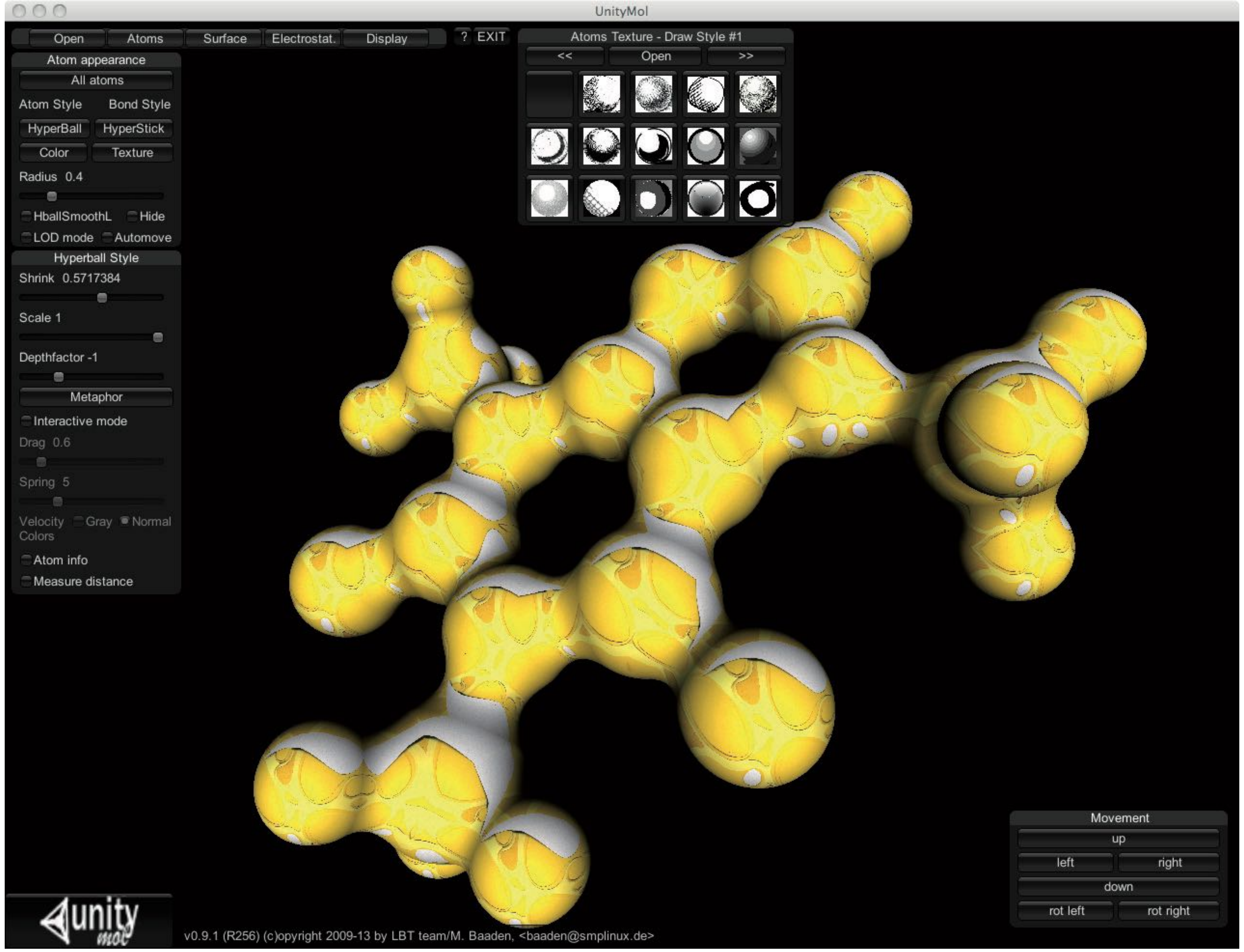

GRAISSE

PROPOSITIONS FINALES :

Intégration au logiciel unitymol, (molécule de caféine)

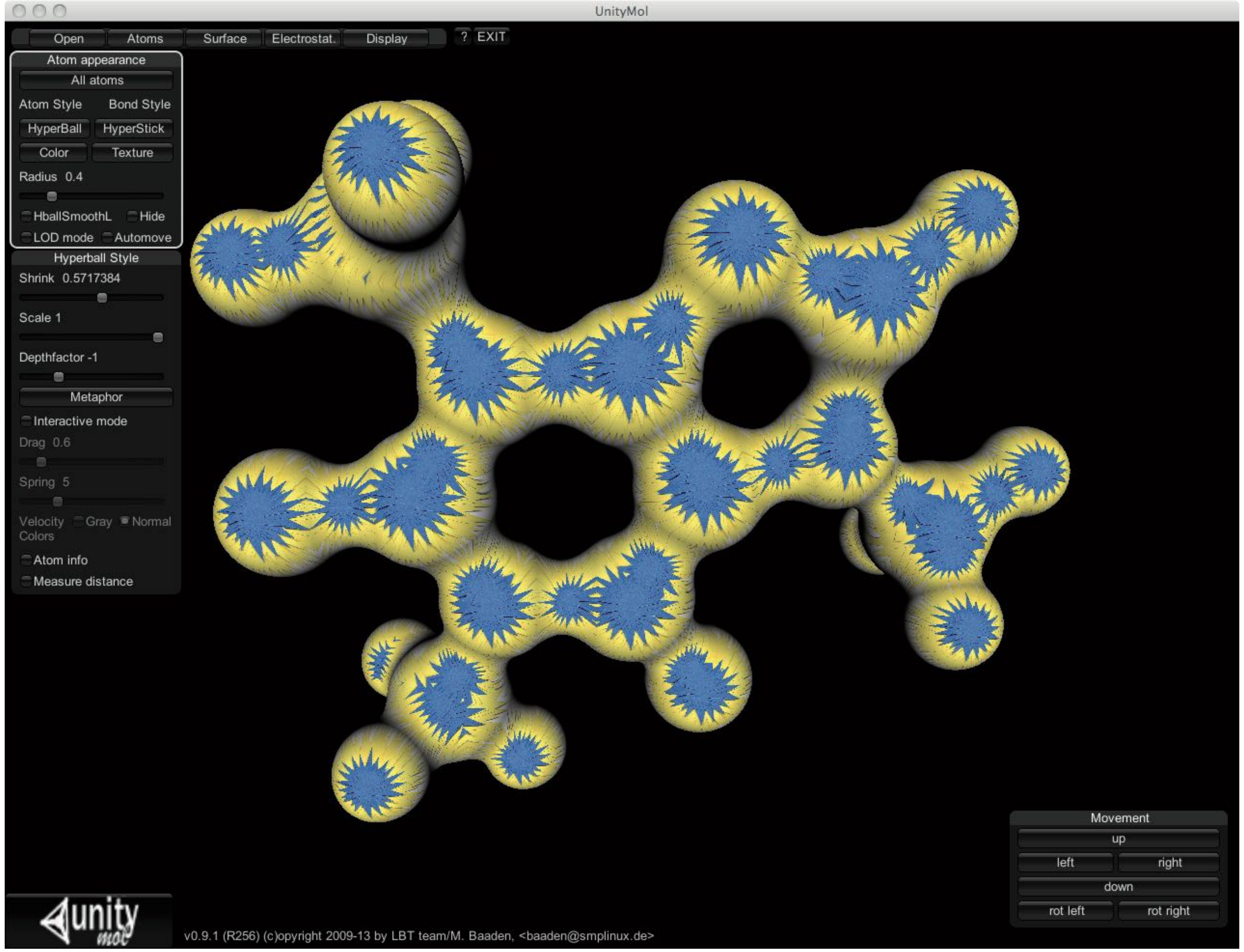

POSITIF

LIT-SPHÈRES

PROPOSITIONS FINALES :

Intégration au logiciel unitymol,
(molécule de caféine)

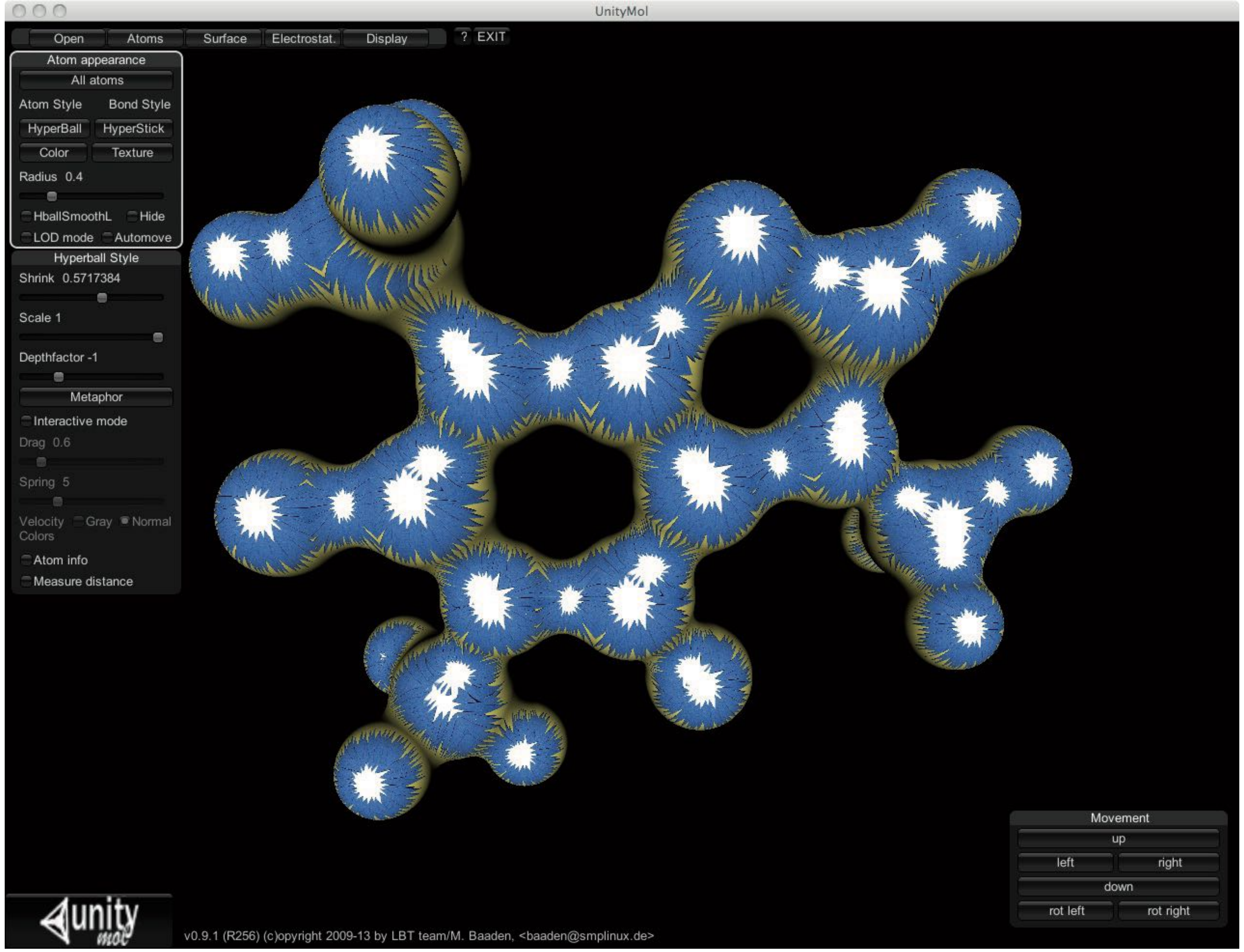

NÉGATIF

ESTELLE VILLEMIN

LIT-SPHÈRES

PROPOSITIONS FINALES :

Intégration au logiciel unitymol,
(molécule de caféine)

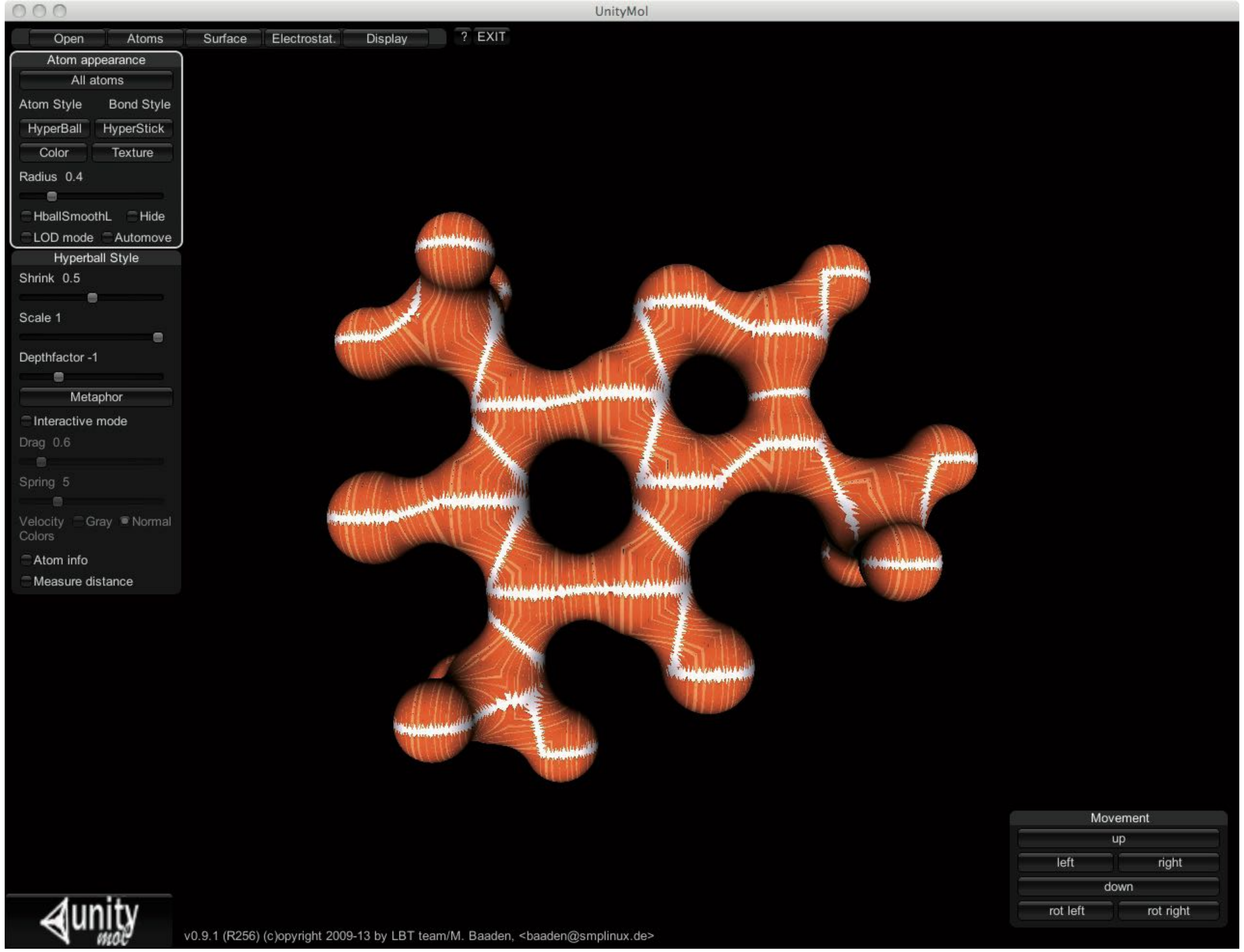

CUIVRE

PROPOSITIONS FINALES :

**Intégration au logiciel unitymol,
(molécule de caféine)**

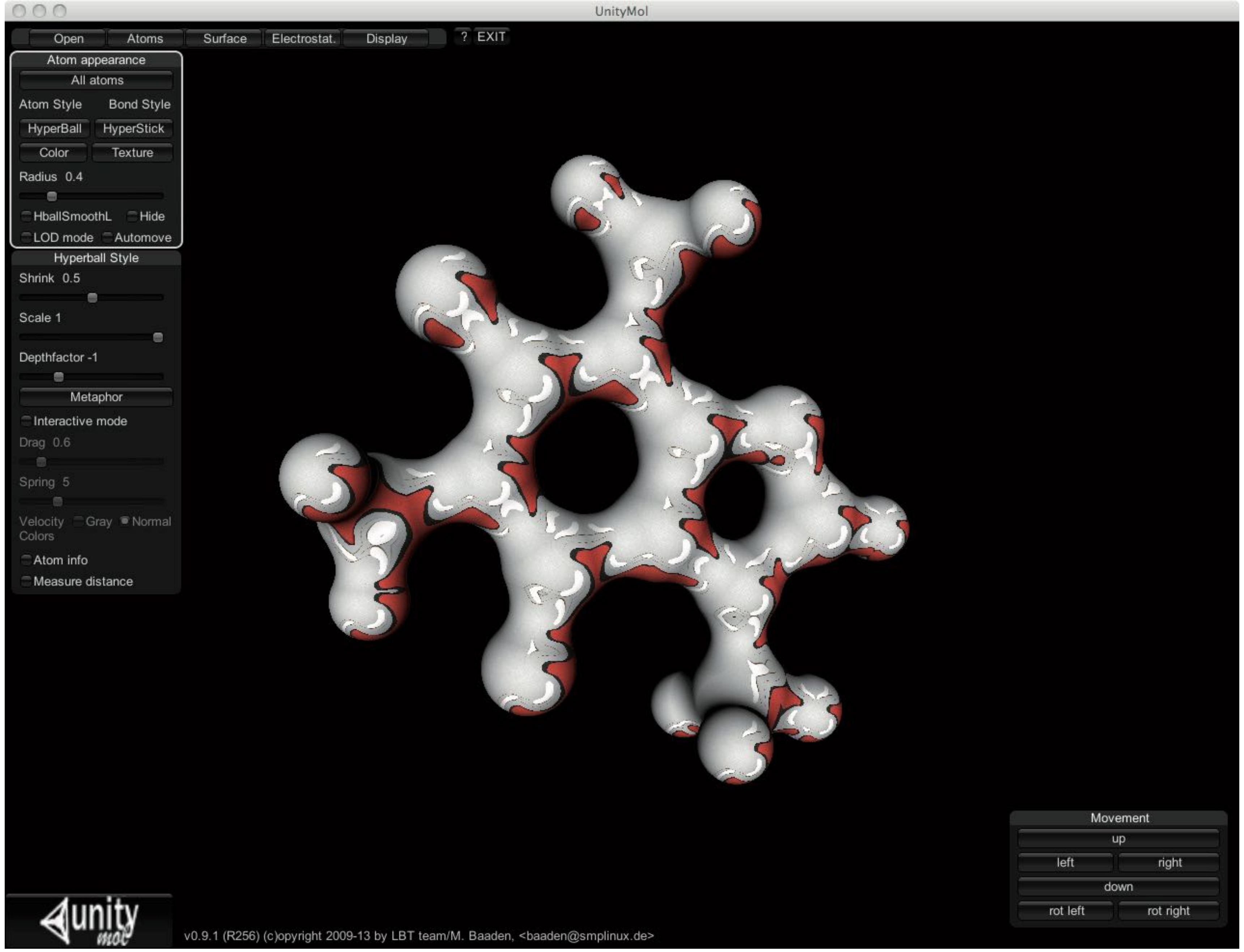

MERCURE

NOIR & BLANC

ESTELLE VILLEMIN

LIT-SPHÈRES

PROPOSITIONS FINALES :

Possibilités de passage au noir et blanc
(d'où l'usage des motifs)

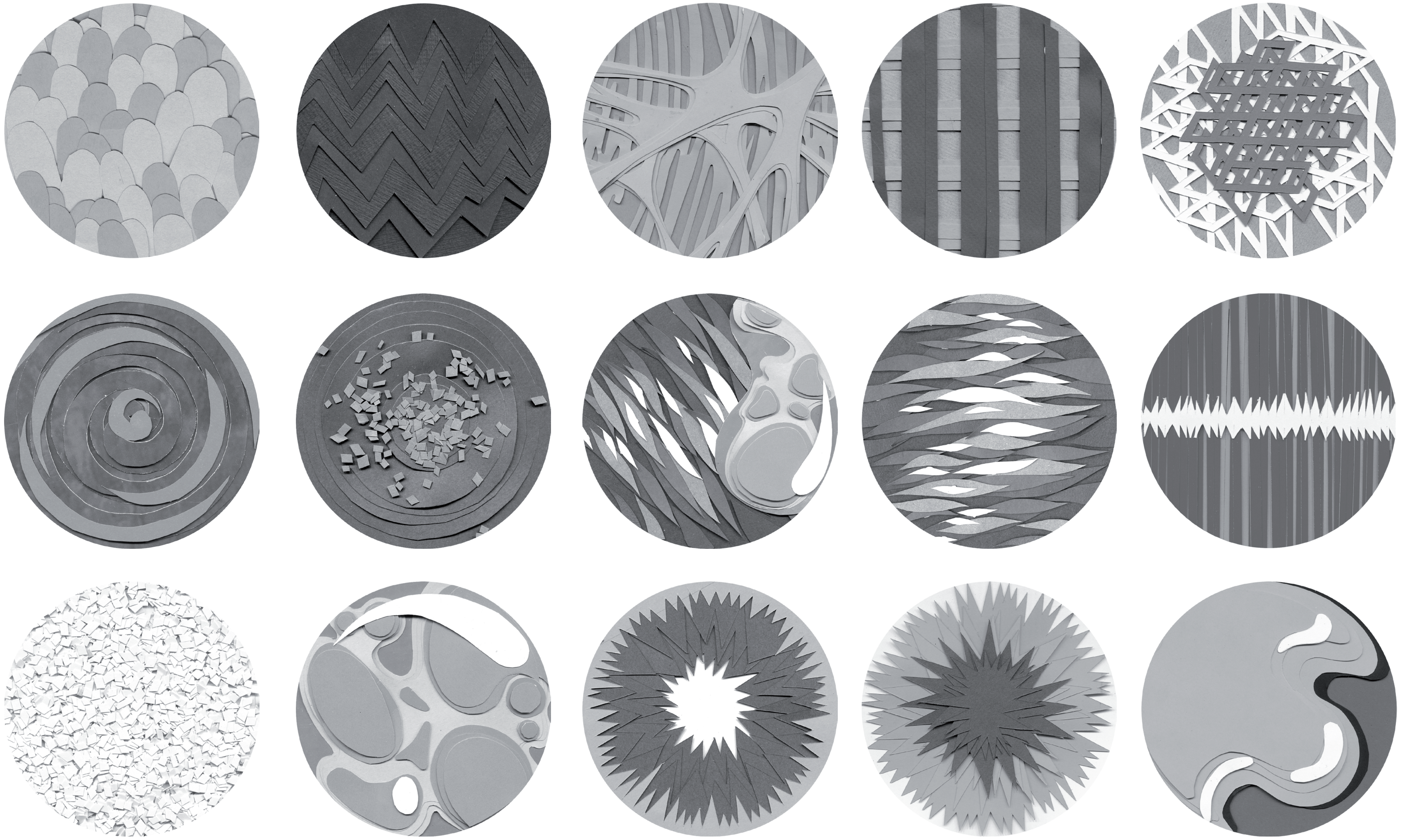

MERCI

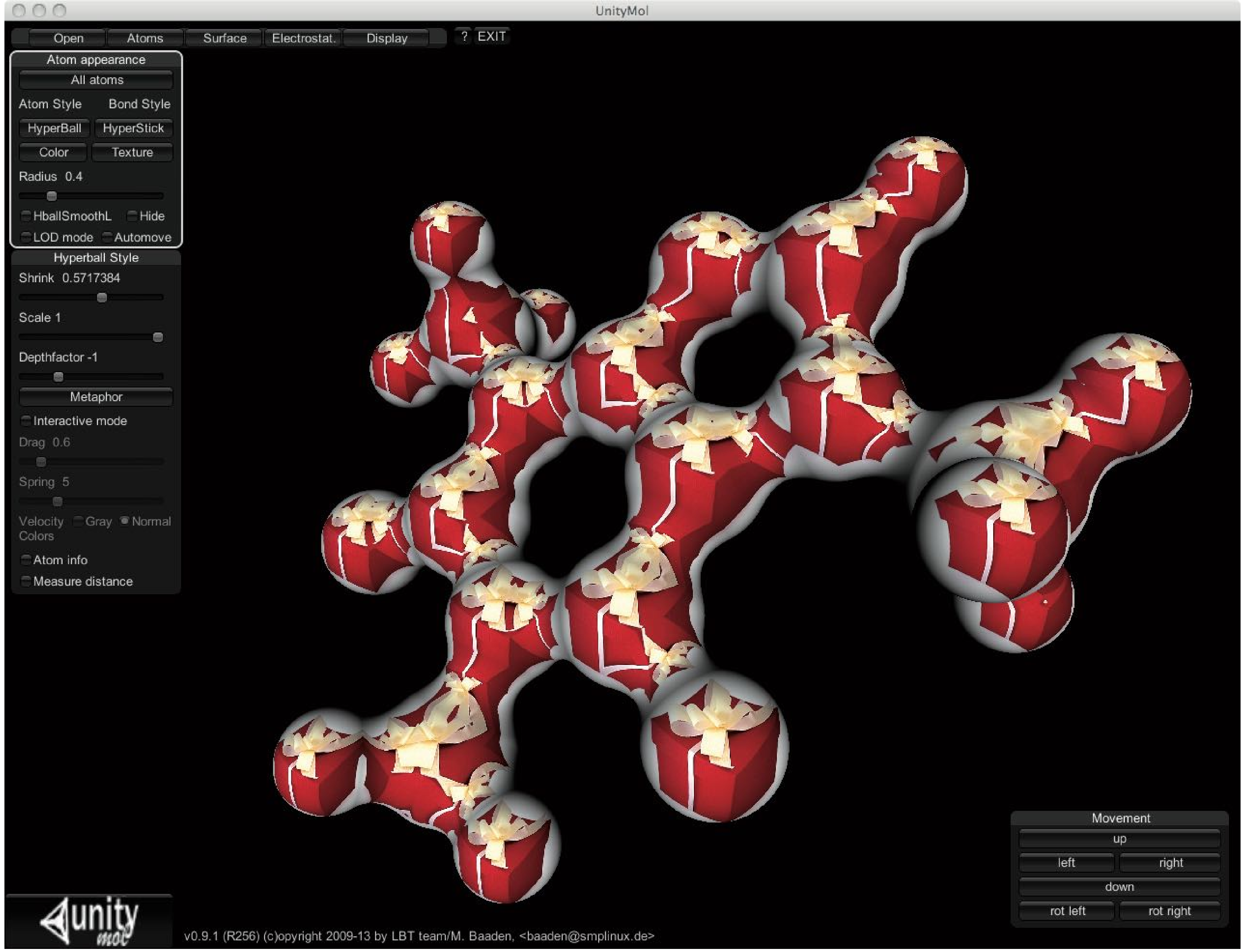
UnityMol
Open
Atoms
Surface
Electrostat.
Display
? EXIT
Atom appearance
All atoms
Atom Style
Bond Style
HyperBall
HyperStick
Color
Texture
Radius 0.4
HballSmoothL
Hide
LOD mode
Automove
Hyperball Style
Shrink 0.5717384
Scale 1
Depthfactor -1
Metaphor
Interactive mode
Drag 0.6
Spring 5
Velocity Colors
Gray
Normal
Atom info
Measure distance
Movement
up
left
right
down
rot left
rot right
unity mol
v0.9.1 (R256) (c)opyright 2009-13 by LBT team/M. Baaden, <baaden@smplinux.de>
