# Supplementary material for: Design – a new way to look at old molecules
Source: J Integr Bioinform. 2022 Jul 1;19(2):20220020. doi: 10.1515/jib-2022-0020 (PMC9377703; doi:10.1515/jib-2022-0020)
Supplement: Supplementary file 2 — Supplementary Material Details [file j_jib-2022-0020_suppl_002.pdf]

SUPPLEMENTARY MATERIAL FOR

Design - a new way to look at old molecules

Davide Spalvieri[2,3], Anne-Marine Mauviel[2,3], Matthieu Lambert[4], Nicolas Férey[2,3,5], Sophie Sacquin-Mora[2,3], Matthieu Chavent[6], Marc Baaden[2,3,1]

[2]CNRS, Université Paris Cité, UPR 9080, Laboratoire de Biochimie Théorique, 13 rue Pierre et Marie Curie, F-75005, Paris, France

[3]Institut de Biologie Physico-Chimique - Fondation Edmond de Rothschild, Paris, France

[4]Ecole Estienne, Paris, France

[5]Université Paris-Saclay, CNRS, Laboratoire interdisciplinaire des sciences du numérique, 91405, Orsay, France.

[6]Institut de Pharmacologie et de Biologie Structurale, Université de Toulouse, CNRS, Université Paul Sabatier, 31400 Toulouse, France

[1] To whom correspondence should be addressed. Email: baaden@smplinux.de

1 Table of contents

The supplementary material contains the following parts:

1) Testimonies from design practicioners

2) Supplementary Figures

3) Projet sketches from Ecole Estienne project

2 The designer in the lab - testimonies from design practitioners

Only in a few scientific disciplines is it common to employ an illustrator or designer in the laboratory. In bioinformatics, and more generally in biology, this is unfortunately a rare opportunity. Nevertheless, there are some bridges, for example through internships in collaboration with design schools, when preparing an illustrated book for public relations, or when a designer decides to work in an academic environment for a while. Here are some testimonials from practitioners in these circles who have had the opportunity to observe or be directly involved in such interactions.

Matthieu Lambert is a lecturer at the Ecole Estienne for Applied Arts in Paris and regularly advises students on their internship projects in academic laboratories. He contributed the following testimony: *"As an instructor of scientific illustration design at the Estienne School, and thus an instructor of future expert illustrators in the design of visual representations useful for communicating a complex message, I would say that the key to the success of an information design project in this field is collaboration. Scientific illustration is a deeply transdisciplinary activity. It is part of a*

creative approach in which the connection between the different actors of the project - designer, scientific sender and receiving audience - is fundamental to ensure the effectiveness of the resulting mediation system. The creation of a space of exchange with dedicated tools or platforms is therefore one of the prerequisites for a project of this type. In this shared space, you can exchange all the elements that are useful for the management of the project, be it language data, knowledge to be acquired or integrated, tests with the people involved, feedback … This is something that I have experienced in my professional experience and that we as a team want to set up and promote in every phase of our educational project. As in the field of scientific research, design research consists in the articulation between hypotheses from collected data or formulated considerations and concrete experiments with different tools. The scientific illustrator must also know how to adapt to different contexts, taking care at each step to enter into a dialogue with his interlocutors, to present his progress in design in a didactic way and to justify each of his choices".

Alain Bade is pedagogical director of EPSAA, the School of Visual Communication of the City of Paris. He commented on the subject of science and visual creation: *"The pedagogical interest in the collaboration between scientific research laboratories and young students of EPSAA of visual communication. The collaboration of knowledge leads to professional skills. EPSAA regularly offers its students concrete projects that confront them with the realities of their future profession and that strengthen their vocation to train talents in visual communication. By exposing them to the constraints and goals of real life, the school helps students develop their future skills. The so-called "scientific popularization" approach that is part of these concrete projects is a complex task, since the goal is to make science*

accessible to the greatest number of people without betraying its meaning, an ideal challenge for a visual communication student. They require a synthetic mind, the ability to understand complex processes, analyze them and translate them into a visual language that can be understood by all audiences. The student learns here his future creative and relational skills. Managing and producing a project (from briefing and meetings to creative design, producing images and changes, and managing the schedule) while discovering the professional and personal skills required for a specific assignment is a unique educational challenge for young people. Indeed, it is an opportunity to put into practice what will be the meaning of their future profession: the talent to communicate through form without betraying substance. This experience is an opportunity for students to discover what constitutes a mission, the progress of a mission, the commitment to their future responsibilities as artistic directors. It is also an opportunity for the lab to see the fruits of its research through a different prism, the opportunity to tackle a communication project whose result will be produced by creative young people and which is a good support to promote the lab's activities.

Davide Spalvieri is an art director, graphic designer, videomaker, and photographer (https://www.davidespalvieri.com/) who has experience with scientific illustrations and has led a project on the family of pentameric ligand-gated ion channels, which we discuss in detail in this article. Davide provided the following comments: "*It's interesting how many scientists are involved in art or simply art lovers. This means that art needs understanding and cognition. In a world where cg, imagery and scan technology are extremely advanced, a creative mind still adds value with his work to science. We can differentiate the illustrator and the designer: an illustrator is asked to realise images by more or less traditional techniques such as hand and digital*

drawing, 3d modeling and rendering. He could employ imagery as part of his work. A designer is asked to find a creative solution for a specific need. Scientific visualization is a domain where both illustration and design skills are required. The solution would likely be a series of visual artifacts that not simply represent the reality for what it is, but explain what we are seeing by itself or alongside a speech, a text or other information. In fact, imagery only translates datas into faithful images. Imagery can't take a picture of a phenomenon (unless we "break" it in multiple moments in time), neither can it represent something that is only supposed to exist, or purely theoretical. A faithful image is not always what we need to understand something: too much information (detail), extremely big or small scales in space (such as in astronomy opposed to microbiology) and the lack of codes lead the viewer to confusion. The illustrator/designer understands what is to hide and what is to visually highlight to make an artifact clear, to make it speak. Simplification is also something that machines can do, but they need parameters set by a human and they are not able to work it through to find a path, a scheme, a structure and links between the elements in the picture. An illustrator/designer can use the conventional visual codes to speak through images, and can even conceive new visual codes. If in the past illustration was the only way to represent reality, today creatives aim to represent what is to tell in the fastest and clearest way our brain can digest".

Carsten Janke is a biologist at Institut Curie in Orsay. He shared his experience as follows: *"As a researcher in Biology, representation of scientific concepts across size and time scales is a difficult task for me, and requires collaboration with graphic designers or artists. My lab has over many years collaborated with artists from different backgrounds, and we even hosted a PhD project at the frontier of art and*

science. The collaboration between scientists and artists can have different types of output: illustrations for scientific publications, in particular review papers, illustrations for scientific presentations, and public outreach. All these forms of communication have in common that our research work, scientific concepts and their greater impact are communicated in a way that is comprehensible to a large public - both scientific and lay. In the past, I have organised several scientific meetings in which artists were documenting the science presented in the talks by drawings, which built the groundwork for future scientific communication and outreach. In a current project, we have integrated an art-science interface into the teaching program of the EPSAA, the École Professionnelle Supérieure d'Arts graphiques of the city of Paris, which sends all their students into different research laboratories of the Paris region to directly interact with researchers and to present their scientific work in a graphic format that is then presented to the lay public in exhibitions around Paris. This project is a great success as it stimulates both, scientists and graphic artists, to go beyond their comfort zones. The interactions with scientists and artists is a fruitful way of fostering scientific thinking and goes beyond the pure illustration of science - it also helps the scientists to think further about their own work, and discover greater concepts that crystallise during their work with the artist".

Renaud Chabrier is a writer, designer, film director and researcher at Ecole Polytechnique - IP Paris. He focuses on how drawing and animation can help in science education, using both traditional and digital techniques. He contributed the following testimony: *"In my career, I had three major opportunities to create links between the world of Cell and Molecular Biology, and the world of Design. The first one was creating animated movies and installations for the general public in the main*

French Science Museum (Cité des Sciences et de l'Industrie). The second was creating scientific drawings for researchers of Institut Curie. The third was being embedded at Institut Curie as an artist-researcher. Each of those experiences confirmed the benefit of a close collaboration between designers and researchers. Hand drawing proves to play a key role in this dialog. It is the most natural way to create and share representations that integrate different views of the same question. Thanks to this tool, the work of a scientific illustrator can be very close to the work of a consultant: through addressing the question "how can we visualize this idea?", it brings researchers the occasion to identify problems like blindspots, contradictions or confusion in the proportions and scales. This is particularly important in the field of Cell and Molecular Biology because those recent sciences did not stabilize the way they cope with representation (unlike Compared Anatomy, for instance). In my experience, most researchers are a bit confused facing the epistemological possibilities and limits of 3D, 2D, drawing, and animation techniques. The designer is here to orient the team towards the best approach for the problem to be addressed, and the kind of public in view. "Design" has recently become a generic word that can gather many activities beyond its classical meaning: author, film director, illustrator, and so on. Today the collaboration between designers and scientific researchers is probably mandatory for a good coordination between science and society. An important caveat is the idealized way researcher see artists and designers, most of the time, as if they were "independent and free" (as suggested by the word "freelance"), flying from one project to the other. Efficient scientific design is more like a teamwork that develops on the long term. It can be very comparable to science laboratories, with less expensive equipment and more diverse collaborations, and I think it should be funded and organized as such".

3 Additional details for sample design implementations

Concerning the **enhanced exploded view** prototype, we first created a geometric representation of the molecule, then an exploded view. The target molecules were proteins with symmetry elements which served to establish criteria for the partitioning of the molecule. The first criterion is to find the symmetry(s) of the molecule. For the GLIC example, we could naturally define a symmetry axis corresponding to the first separation level we were aiming for, as shown in the second sub-panel in Figure 2c. For other levels of separation, as shown in other sub-panels of Figure 2c, we alternatively thought of biological criteria, e.g., the partitioning of a membrane protein into the part inside the cell, the membrane, and the part outside. For each isolated element of the molecule, an enclosing shape had to be defined, as shown in Figure 7c. To this end, we performed projections of horizontal and vertical points and various calculations to define this enclosing shape. Once it was constructed, we determined the transition matrix for the symmetry-related copies. With the molecule fully modeled and divided into different meshes according to its subunits, the motion of each mesh is defined by the vector between its barycenter and a point on the symmetry axis, orthogonal to the symmetry axis.

Supplementary Figures

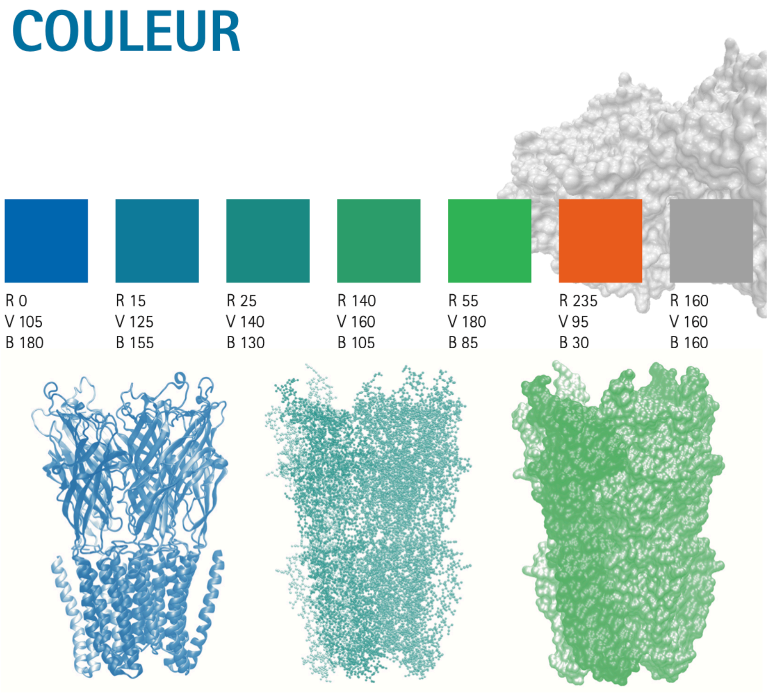

Supplementary Figure S1: Design of a color palette for the experiments with ion channel illustrations.

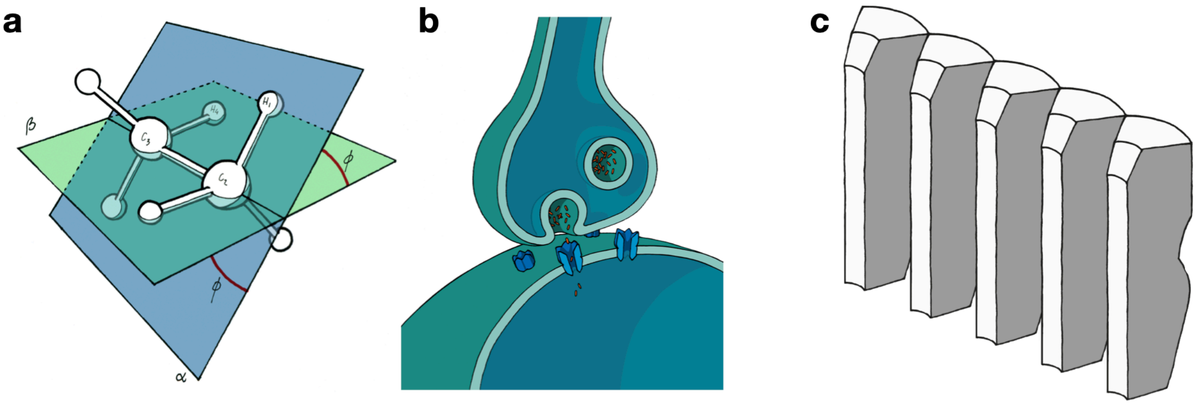

Supplementary Figure S2: First finalized drawings: a) dihedral angle concept, b) ion channels at the synapse, c) unfolding ion channel subunits.

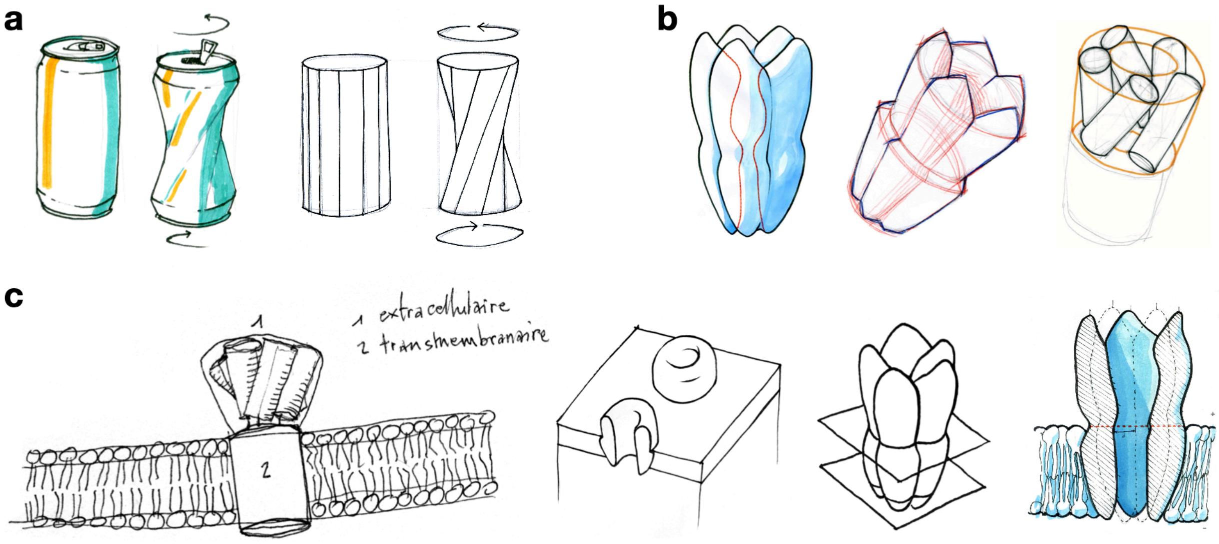

Supplementary Figure S3: Sketches. a) Illustration of twist as a motion representing the gating of an ion channel, based on the analogy of a metal can. b) Overall shape of the ion channel. c) Addition of the membrane to the image.

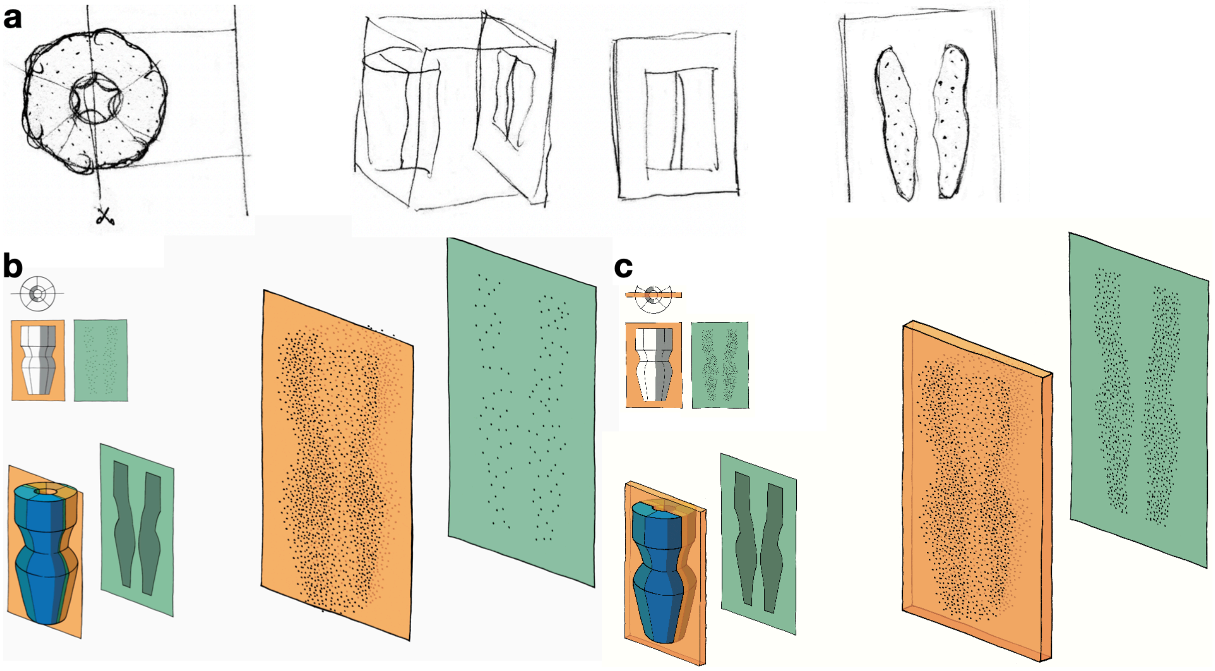

Supplementary Figure S4: Slicing through an ion channel, a) first sketches, b) thin slices, c) slices of defined thickness.

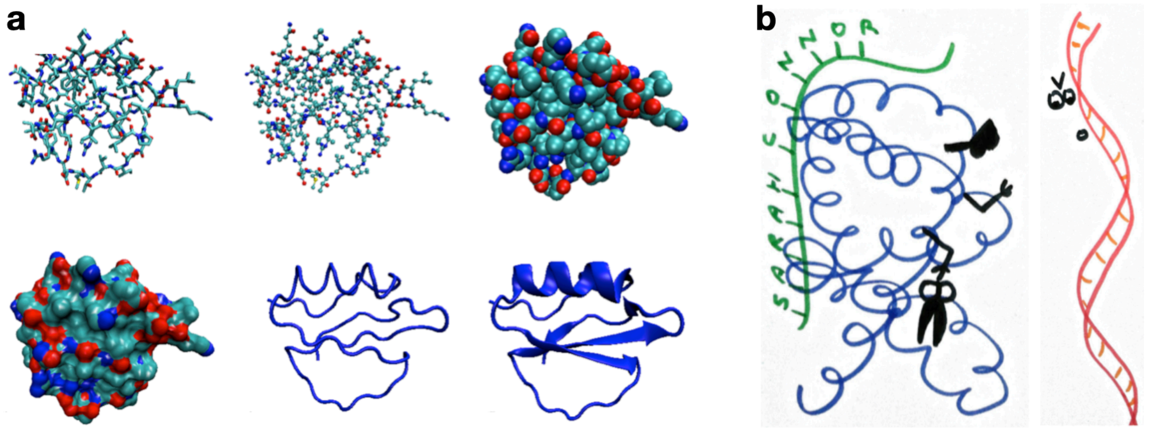

Supplementary Figure S5: Two examples of illustrations originally found on the Top of the Prots blog. a) Chimotrypsin inhibitor (PDB 2ci2) shown with six different display styles of VMD (licorice, cpk, van der Waals, surface, tube, and new cartoon) and the default colors. b) CRISPR-Cas9 (left) talking to the DNA strand it is supposed to cut into pieces (drawing by S. Sacquin-Mora).

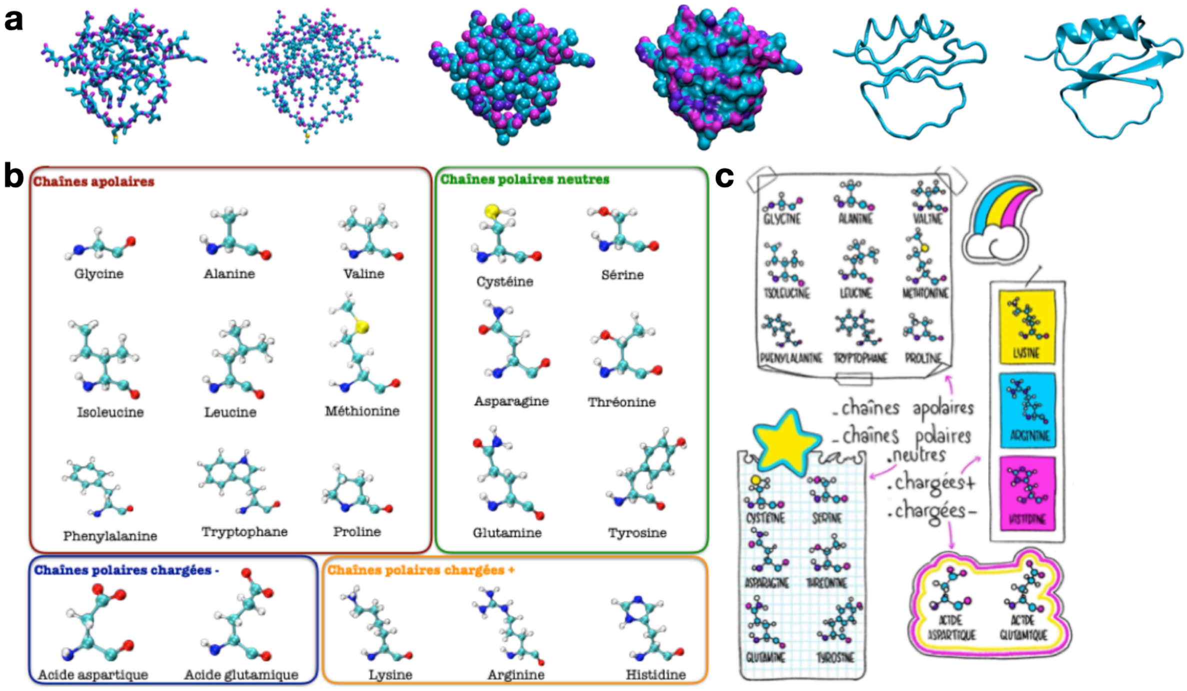

Supplementary Figure S6: a) Six representations of the chimotrypsin inhibitor with Anmryn's new color palette. b) Original table of natural amino acids as found on the Top of the Prots blog. c) Published version of Anmryn's amino acid table (with permission from EDP-Sciences).

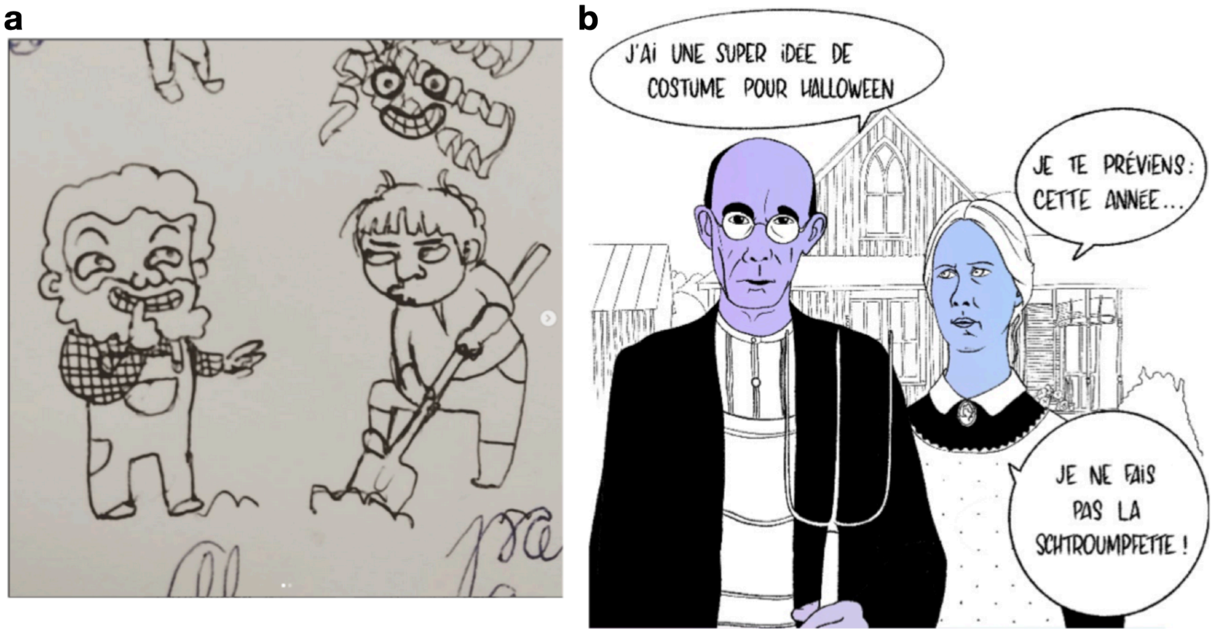

Supplementary Figure S7: The "Blue Fulgates" were an American family known to inherit a disordered hemoglobin that resulted in a blue skin color. a) Early draft by Anmryn. b) Published version of the illustration (with permission from EDP-Science).

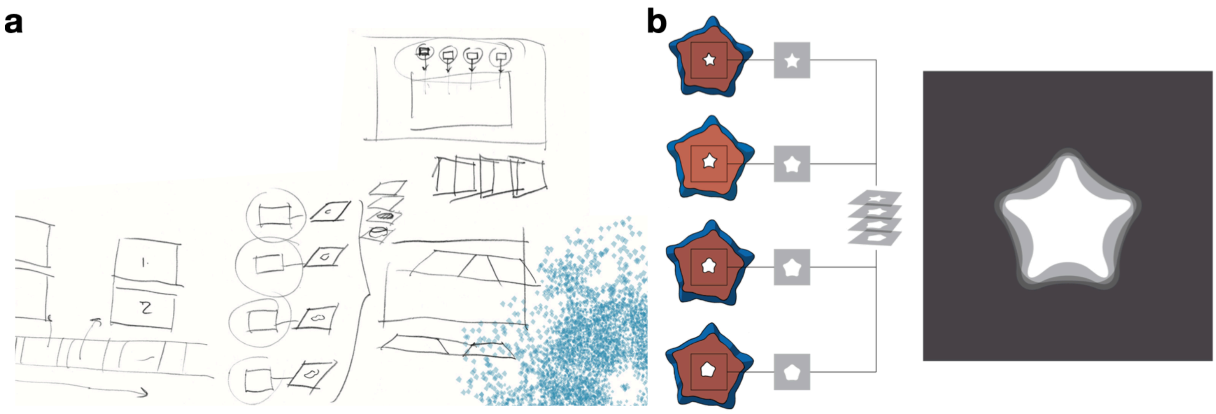

Supplementary Figure S8: Time series of slices through the channel contour, a) first sketches, b) first drawing of time averaging.

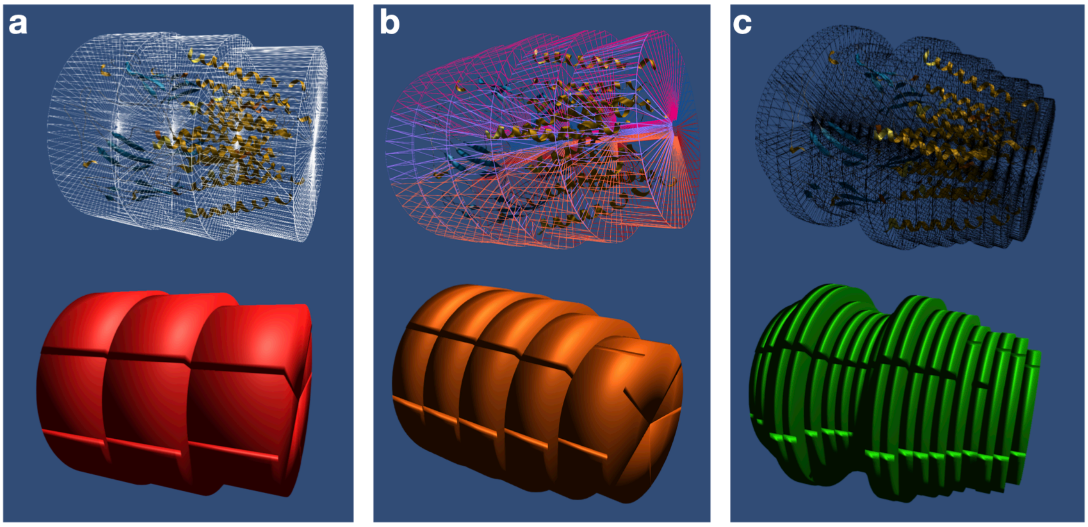

Supplementary Figure S9: Shape abstraction and explosion revisited, a) few horizontal subdivisions, b) refining the shape by adding geometrical elements, c) very fine subdivision.

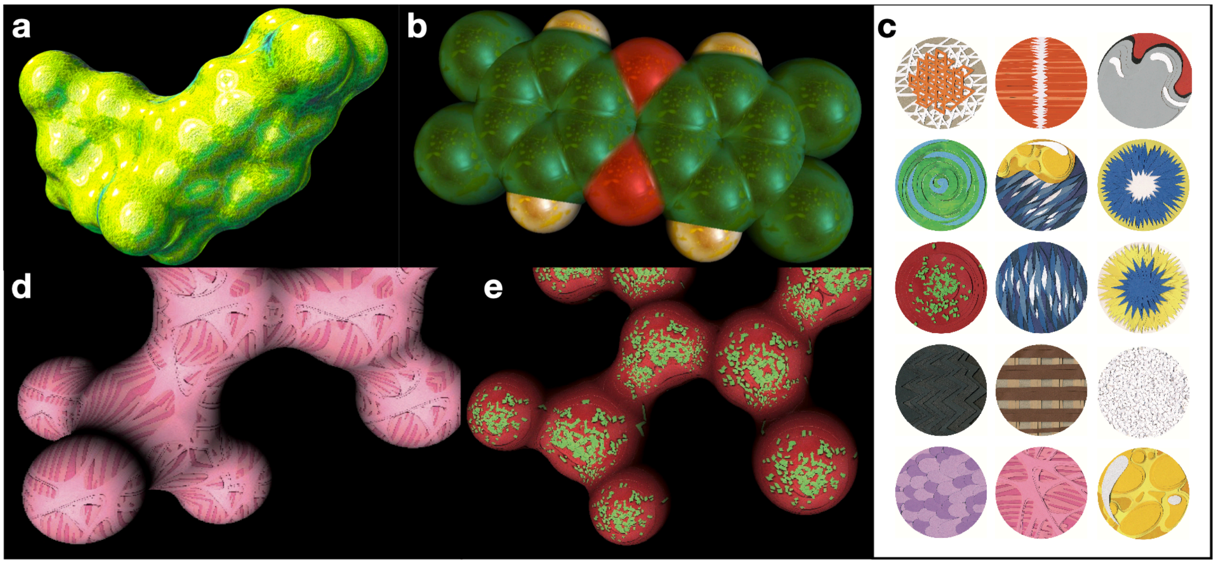

Supplementary Figure S10: Lit sphere lighting experiments. a) Hand-drawn surface example shown to students for inspiration. b) Example of molecule mediating toxicity through lit sphere lighting. c) Set of spheres created by student Estelle Villemin to illustrate different concepts with lit spheres. d) Experiment illustrating flexibility. e) Experiment illustrating toxicity.

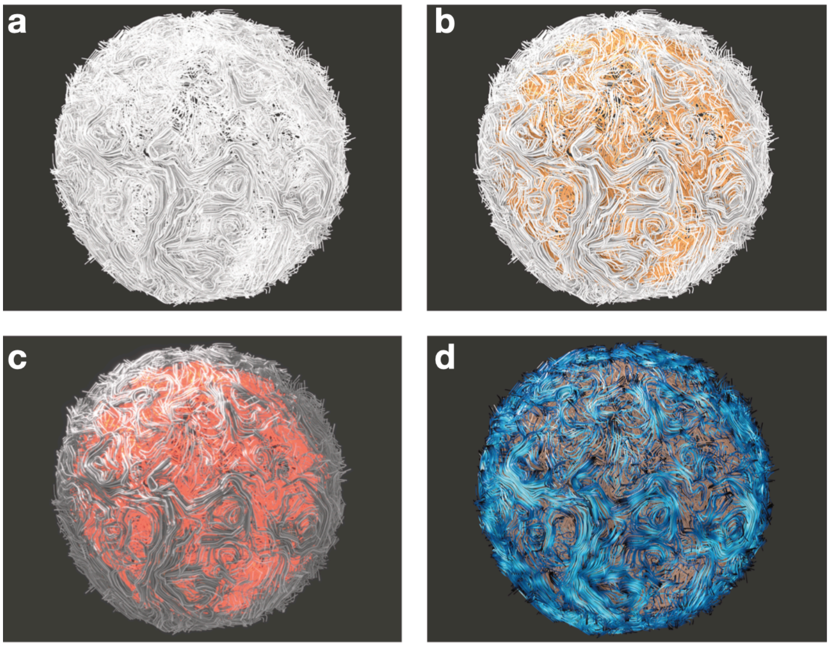

Supplementary Figure S11: Use of Blender to render lipid flows at the surface of a vesicle model (51). a) Ambient Occlusion lighting to highlight dense areas. b) Adding orange color to better visualise inner flows. c) Adding a *glow* lighting effect to further highlight the inner lipid flows but limiting the perception of outer lipid flows. d) Assigning adequate colors and lighting to both perceive outer and inner flows.

Project sketches from Ecole Estienne project

We set up a student project to experiment with the added value of artistic renderings of molecular objects through lit sphere shading. This approach aims to intuitively visualize molecular properties. Here, we provide some details on these experiments related to **lit sphere lighting**, sometimes referred to as matcap texturing. The rendering technique is inspired by artistic methods for rendering light and shadow on a three-dimensional object drawn on a two-dimensional surface. Based on the fact that a 2D representation of a sphere encodes all possible light values for a 3D object, we can use a 2D texture representing a sphere and its lighting, called a lit sphere, as the basis for lighting any 3D object. This process ensures consistent shading and transfers all the artistic value put into the lit sphere to the 3D object. For example, a near-photorealistic rendering is possible when you use spheres extracted from a photograph of a material such as metal, stone, glass, etc. Using a drawn sphere as a reference texture results in an illustrative rendering that approximates the style of an illustrator. This principle can be used in UnityMol to illuminate 3D meshes: The orientation of a pixel relative to the camera is determined by its normals. Then all visible normals can be retrieved for a viewport on a theoretical hemisphere in the centre of the screen. So if you map the pixel normals to a 2D texture representing a sphere, you can achieve the corresponding lit sphere shading. This is an easy way to render complex lighting effects in real time with simple square textures.

We carried out a collaborative experiment on lit sphere lighting with the Ecole Estienne art school. A group of 8 students was asked to design lit spheres prototypes: Julie Borgese, Elsa Depont, Joana Gouin, Margaux Khalil, Quentin Lebeau, Eve Nagy, Claire Thibon and Estelle Villemin. Their goal was to illustrate molecular structures and properties using original and intuitive approaches. The idea

is to perceive both the shapes and complementarities of molecular objects and the distribution of their physical properties, such as the charges carried by atoms or their affinity for water (hydrophilicity) or fats (lipophilicity). Since these are invisible objects, the role of an image is both ambiguous and essential to our understanding of these structures. Students were asked to design a visual nomenclature that explicitly and directly describes the various properties (charge, flexibility, affinity...) that can be attributed to molecules. This set of visual properties should enrich the visual repertoire that can be directly applied to the analysis and dissemination of modeling results by researchers. This graphical task referred only to the properties and not to the structures of the molecules. The visual system with which the students were to deal with evolved around the following features: Polarity, charge and electronegativity, hydrophilicity and hydrophobicity, toxicity or environmental compatibility, flexibility, rigidity, activity (active site of an enzyme), qualitative evaluation (good, bad), type of molecule (protein, fat, water, solute, sugar), type of atom (carbon, sufficiency, oxygen,...). In this investigation, each student had to determine the conceptual tools (signs, images, symbols...) as much as possible (traditional or digital tools, use of colors...) to create a system of images that is both consistent and communicative. The experiment was supervised by three instructors from the art school: Antoine Barnaud, Annaïck Péron and Matthieu Lambert. Supplementary Figure S10a,b show two examples that were shown to the students for inspiration. In the first, a special hand-drawing style was used to illustrate the shape of molecular objects; in the second, tetrachlorodibenzodioxin is shown in a Van der Waals representation with texturing to emphasize its toxicity. One solution was based on paper-cut and assembled spheres (Supplementary Figure S10c), with the example of representing the flexibility concept through a sort of elastomeric pattern (Supplementary Figure S10d), or the toxicity property through a combination

of color and texturing (Supplementary Figure S10e). Many more original propositions were produced by the students, which are all provided as further supplementary material in a compiled document.
